# Supplementary material for: Hemocytes facilitate interclonal cooperation-induced tumor malignancy by hijacking the innate immune system in Drosophila
Source: EMBO J. 2025 Aug 22;44(19):5394–428. doi: 10.1038/s44318-025-00547-5 (PMC12489090; doi:10.1038/s44318-025-00547-5)
Supplement: Supplementary file 1 — Table EV1 [file 44318_2025_547_MOESM1_ESM.docx]

**Table EV1 Oligonucleotides and Drosophila strains used in this study.**

1. Oligonucleotides used in this study.

| **Oligonucleotides** | **Sequence** | **IDENTIFIER** |
| --- | --- | --- |
| qPCR-*AttB*-F: | 5-GCAACATGCAGAAGACAAGCA | N/A |
| qPCR-*AttB*-R: | 5-AAGGAACCTCCAAGCACCTG | N/A |
| qPCR-*AttC*-F: | 5-TCCGTATACCCAGCCACTGA | N/A |
| qPCR-*AttC*-R: | 5-TTAGGTCCAATCGGGCATCG | N/A |
| qPCR-*AttD*-F: | 5-GGTCGGTGATGATCTTGCCA | N/A |
| qPCR-*AttD*-R: | 5- ATGCTGCAGTGAGAGTGCAT | N/A |
| qPCR-*Def*-F: | 5-TTATTGCAGAAACGGGCTCT | N/A |
| qPCR-*Def*-R: | 5-ATGGTAAGTCGCTAACGCTAATG | N/A |
| qPCR-*Mtk*-F: | 5- GCATCAATCAATTCCCGCCA | N/A |
| qPCR-*Mtk*-R: | 5-GCTCTGCCAGCACTGATGTA | N/A |
| qPCR-*Dro-*F: | 5-ACCTGACTCAAGCTGCCATC | N/A |
| qPCR-*Dro-*R: | 5-CGATGGGAACCCCTCATTGT | N/A |
| qPCR-*Drs*-F: | 5-CCAAGCTCCGTGAGAACCTT | N/A |
| qPCR-*Drs*-R: | 5-CAGGTCTCGTTGTCCCAGAC | N/A |
| qPCR-*Myc*-F: | 5-AGCCAGAGATCCGCAACATC | N/A |
| qPCR-*Myc*-R: | 5-CGCGCTGTAGAGATTCGTAGAG | N/A |
| qPCR-*ex-*F: | 5-AGAGTGCACGAAGAGTGAGC | N/A |
| qPCR-*ex-*R: | 5-AATCGCGAGCCTGGTGATAG | N/A |
| qPCR-*Diap1*-F: | 5-ATAGCCAACGAAAGTGCGGA | N/A |
| qPCR- *Diap1*-R: | 5-CTCATCTCCAGCGTCGAGTC | N/A |
| qPCR-*wg*-F: | 5-GACCCAGCGATCCACTCTAC | N/A |
| qPCR-*wg*-R: | 5-CGGCGATTTCTGAACTGGTGT | N/A |
| qPCR-*ban*-F: | 5-ATTTGACTACGAAACCGGTTTTCG | N/A |
| qPCR-*ban*-R: | 5-CAGCTTTCAAAATGATCTCACTTG | N/A |
| qPCR-*Mmp1*-F: | 5-GAAGGCTCGGACAACGAGT | N/A |
| qPCR-*Mmp1*-R: | GTCGTTGGACTGGTGATCG | N/A |
| qPCR-*Ets21C*-F: | 5-CAACGACGACGAACCAAAT | N/A |
| qPCR-*Ets21C*-R: | 5-GTTCGCGTTGGACGAATC | N/A |
| qPCR-*Pvf1*-F: | 5-AAGCCGGAACACCATTGAC | N/A |
| qPCR-*Pvf1*-R: | 5-CATGATGCTGCGCTTAAAGT | N/A |
| qPCR-*Pvf2*-F: | 5-CCTATGCCCCAAGCACTTCA | N/A |
| qPCR-*Pvf2*-R: | 5-CCCTCAACGCCGTTTTTCAG | N/A |
| qPCR-*rp49*-F: | 5-TCCTACCAGCTTCAAGATGACC | N/A |
| qPCR-*rp49*-R: | 5-CACGTTGTGCACCAGGAACT | N/A |

2. Drosophila strains used in this study.

| **REAGENT or RESOURCE** | **SOURCE** | **IDENTIFIER** |
| --- | --- | --- |
| w^1118^ | Bloomington Drosophila Stock Center | Cat# 5905 |
| UAS-p35 | Bloomington Drosophila Stock Center | Cat# 5072 |
| w^67c23^, myc-LacZ^G0354^ | Bloomington Drosophila Stock Center | Cat# 11981 |
| ban-LacZ | Bloomington Drosophila Stock Center | Cat# 10154 |
| spz^2^ | Bloomington Drosophila Stock Center | Cat# 3115 |
| alphaTub84B-QF2 | Bloomington Drosophila Stock Center | Cat# 51958 |
| QUAS-mCD8-GFP | Bloomington Drosophila Stock Center | Cat# 30002 |
| UAS-Dorsal-H | Bloomington Drosophila Stock Center | Cat# 9319 |
| ex^e1^ (ex-lacZ) | Bloomington Drosophila Stock Center | Cat# 44249 |
| FRT82B,tub-QS.P | Bloomington Drosophila Stock Center | Cat# 30034 |
| UAS-Dif | Bloomington Drosophila Stock Center | Cat# 22201 |
| UAS-hpo-Flag | Bloomington Drosophila Stock Center | Cat# 44254 |
| UAS-wts(Ⅲ) | Bloomington Drosophila Stock Center | Cat# 44258 |
| UAS-Tl^RNAi^ | Vienna Drosophila Resource Center | Cat# 330125 |
| UAS-dl^RNAi#1^ | Vienna Drosophila Resource Center | Cat# 45996 |
| UAS-dl^RNAi#2^ | Vienna Drosophila Resource Center | Cat# 45998 |
| UAS-Dif^RNAi#1^ | Vienna Drosophila Resource Center | Cat# 30578 |
| UAS-Dif^RNAi#2^ | Vienna Drosophila Resource Center | Cat# 30579 |
| UAS-Ras^V12^ | Gift from Tian Xu (Westlake University) | N/A |
| UAS-Spz* | Gift from Tian Xu (Westlake University) | (Mishra-Gorur, Li et al., 2019) |
| FRT82B | Gift from Tian Xu (Westlake University) | N/A |
| FRT79E | Gift from Tian Xu (Westlake University) | N/A |
| M6^-/-^, FRT 79E | Gift from Tian Xu (Westlake University) | (Dunn, Rush et al., 2018) |
| ey-Flp; act>y+>Gal4, UAS-GFP; tub-Gal80, ri FRT 79E（79E tester） | Gift from Tian Xu (Westlake University) | (Ma, Lu et al., 2020) |
| ey-Flp5, act>y+>Gal4, UAS-GFP; Tub-Gal80, FRT 79E/S-T（79E tester） | Gift from Tian Xu (Westlake University) | (Ma et al., 2020) |
| UAS-Toll[10B] | Gift from Lei Xue (Tongji University) | (Wu, Chen et al., 2015) |
| Cg-Gal4 | Gift from Lei Xue (Tongji University) | N/A |
| UAS-Tub^RNAi^ | Gift from Lei Xue (Tongji University) | (Wu et al., 2015) |
| UAS-wts | Gift from Shian Wu (Nankai University) | (Wu, Huang et al., 2003) |
| QUAS-Ras^V12^ | Gift from Tatsushi Igaki(Kyoto University) | (Enomoto M., et al., 2021, Dev Cell) |
| Drosophila melanogaster: yki^B5^ | Gift from Duojia Pan (Howard Hughes Medical Institute) | FBal0194179 |
| Drosophila melanogaster: UAS-yki^S168A^ | Gift from Duojia Pan (Howard Hughes Medical Institute) | (Huang, et al., 2005, Cell) |
| fj*^9-II^* (fj-lacZ) | Bloomington Drosophila Stock Center | Cat# 6370 |
| Hml-Gal4 | Gift from Lei Xue (Tongji University) | N/A |
| UAS-spz^RNAi^ | Bloomington Drosophila Stock Center | Cat# 28538 |
| UAS-rprC^M147^ | Gift from Lei Xue (Tongji University) | N/A |
| UAS-hid | Gift from Lei Xue (Tongji University) | N/A |
| UAS-Ets21C^RNAi#1^ | Vienna Drosophila Resource Center | Cat# 51225 |
| UAS-Ets21C^RNAi#2^ | Tsinghua Fly Center | THU3974 |
| He-Gal4 | Gift from Lei Xue (Tongji University) | N/A |
| yw,eyFlp; act>y+>Gal4, UAS-RFP/CyO; TG80, ri FRT79E | Gift from Tian Xu (Westlake University) | N/A |
| UAS-Pvf1^RNAi#1^ | Vienna Drosophila Resource Center | Cat# 6175 |
| UAS-Pvf2^RNAi#1^ | Vienna Drosophila Resource Center | Cat# 7629 |
| UAS-pll-3xHA | Fly ORF | Cat# 1257 |
| UAS-pelle^RNAi^ | Gift from Lei Xue (Tongji University) | (Wu et al., 2015) |
| UAS-Pvr.DN | Bloomington Drosophila Stock Center | Cat# 58430 |
| UAS-Relish^RNAi#^ | Vienna Drosophila Resource Center | Cat# 49414 |
| UAS-Gli^RNAi^ | Bloomington Drosophila Stock Center | Cat# 58115 |
| UAS-aka^RNAi^ | Bloomington Drosophila Stock Center | Cat# 67014 |
| tub-QS | Bloomington Drosophila Stock Center | Cat# 30021 |
| Tl^[MI01254]^ | Bloomington Drosophila Stock Center | Cat# 36134 |
| UAS-bsk^DN^ | Bloomington Drosophila Stock Center | Cat# 6409 |
| UAS-M6 | Gift from Tian Xu (Westlake University) | N/A |
| Def^SK3^ | Gift from zongzhao zhai (Hunan Normal University) | (Hanson, Dostalova et al., 2019) |
| Def^SK3^,AttC^MI^,Dro-Att^SK2^,Dpt^SK1^ | Gift from zongzhao zhai (Hunan Normal University) | (Hanson et al., 2019) |
| rpr-lacZ | Bloomington Drosophila Stock Center | Cat# 58793 |
| puc^[E69]^ | Gift from Tian Xu (Westlake University) | N/A |
| UAS-spz^RNAi^ | Bloomington Drosophila Stock Center | Cat# 58499 |
| UAS-Pvf1^RNAi#2^ | Bloomington Drosophila Stock Center | Cat# 39038 |
| UAS-Pvf2^RNAi#2^ | Bloomington Drosophila Stock Center | Cat# 61955 |
