# Supplementary material for: Hemocytes facilitate interclonal cooperation-induced tumor malignancy by hijacking the innate immune system in Drosophila
Source: EMBO J. 2025 Aug 22;44(19):5394–428. doi: 10.1038/s44318-025-00547-5 (PMC12489090; doi:10.1038/s44318-025-00547-5)
Supplement: Supplementary file 2 — Table EV2 [file 44318_2025_547_MOESM2_ESM.docx]

**Table EV2: Detailed genotypes for each figure.**

A complete list of detailed genotypes used in this study is provided below, corresponding to Figures 1 through 7 and Figures EV1 through EV8. The detailed genotypes for each experiment are as follows:

| **Fig 1**. |  |
| --- | --- |
| **Fig 1C** | *y, w/+; eyFLP5, Act5C>y+>Gal4, UAS-GFP/+; Tub-Gal80, FRT79E/FRT79E* |
|  | *y, w/+; eyFLP5, Act5C>y+>Gal4, UAS-GFP/UAS-Ras^V12^; Tub-Gal80, FRT79E/FRT79E* |
|  | *y, w/+; eyFLP5, Act5C>y+>Gal4, UAS-GFP/UAS-Ras^V12^; Tub-Gal80, M6^W186*^, FRT79E/FRT79E* |
|  |  |
| **Fig 1E** | *y, w/+; eyFLP5, Act5C>y+>Gal4, UAS-GFP/+; Tub-Gal80, FRT79E/FRT79E* |
|  | *y, w/+; eyFLP5, Act5C>y+>Gal4, UAS-GFP/UAS-Ras^V12^; Tub-Gal80, FRT79E/FRT79E* |
|  | *y, w/+; eyFLP5, Act5C>y+>Gal4, UAS-GFP/UAS-Ras^V12^; Tub-Gal80, M6^W186*^, FRT79E/FRT79E* |
|  |  |
| **Fig 1G** | *y, w, eyFLP1/w or Y; Tub-QF, QUAS-mCD8-GFP, Act5C>y+>Gal4/+; FRT82B, Tub-QS/QUAS-Ras^V12^, FRT82B, Tub-Gal80* |
|  | *y, w, eyFLP1/w or Y; Tub-QF, QUAS-mCD8-GFP, Act5C>y+>Gal4/UAS-hid; FRT82B, Tub-QS/QUAS-Ras^V12^, FRT82B, Tub-Gal80* |
|  | *y, w, eyFLP1/w or Y; Tub-QF, QUAS-mCD8-GFP, Act5C>y+>Gal4/UAS-Gli^RNAi^; FRT82B, Tub-QS/QUAS-Ras^V12^, FRT82B, Tub-Gal80* |
|  | *y, w, eyFLP1/w or Y; Tub-QF, QUAS-mCD8-GFP, Act5C>y+>Gal4/UAS-aka^RNAi^; FRT82B, Tub-QS/QUAS-Ras^V12^, FRT82B, Tub-Gal80* |
|  | *y, w, eyFLP1/w or Y; Tub-QF, QUAS-mCD8-GFP, Act5C>y+>Gal4/+; Tub-QS, M6^W186*^, FRT79E/QUAS-Ras^V12^, FRT79E* |
|  |  |
| **Fig 2**. |  |
| **Fig 2D** | *y, w/+; eyFLP5, Act5C>y+>Gal4, UAS-GFP/UAS-Ras^V12^; Tub-Gal80, FRT79E/FRT79E* |
|  | *y, w/+; eyFLP5, Act5C>y+>Gal4, UAS-GFP/UAS-Ras^V12^; Tub-Gal80, M6^W186*^, FRT79E/FRT79E* |
|  |  |
| **Fig 2E** | *y, w/+; eyFLP5, Act5C>y+>Gal4, UAS-RFP/UAS-Ras^V12^; Tub-Gal80, FRT79E/FRT79E, Toll-GFP* |
|  | *y, w/+; eyFLP5, Act5C>y+>Gal4, UAS-RFP/UAS-Ras^V12^; Tub-Gal80, M6^W186*^, FRT79E/FRT79E, Toll-GFP* |
|  |  |
| **Fig 2G** | *y, w/+; eyFLP5, Act5C>y+>Gal4, UAS-GFP/UAS-Ras^V12^; Tub-Gal80, FRT79E/FRT79E* |
|  | *y, w/+; eyFLP5, Act5C>y+>Gal4, UAS-GFP/UAS-Ras^V12^, UAS-Tl^RNAi^; Tub-Gal80, FRT79E/FRT79E* |
|  | *y, w/+; eyFLP5, Act5C>y+>Gal4, UAS-GFP/UAS-Ras^V12^; Tub-Gal80, FRT79E/UAS-tub^RNAi^, FRT79E* |
|  | *y, w/+; eyFLP5, Act5C>y+>Gal4, UAS-GFP/UAS-Ras^V12^, UAS-pll^RNAi^; Tub-Gal80, FRT79E/ FRT79E* |
|  | *y, w/+; eyFLP5, Act5C>y+>Gal4, UAS-GFP/UAS-Ras^V12^, UAS-dl^RNAi#1^; Tub-Gal80, FRT79E /FRT79E* |
|  | *y, w/+; eyFLP5, Act5C>y+>Gal4, UAS-GFP/UAS-Ras^V12^, UAS-Dif^RNAi#1^; Tub-Gal80, FRT79E/FRT79E* |
|  | *y, w/+; eyFLP5, Act5C>y+>Gal4, UAS-GFP/UAS-Ras^V12^; Tub-Gal80, M6^W186*^, FRT79E /FRT79E* |
|  | *y, w/+; eyFLP5, Act5C>y+>Gal4, UAS-GFP/UAS-Ras^V12^, UAS-Tl^RNAi^; Tub-Gal80, M6^W186*^, FRT79E/FRT79E* |
|  | *y, w/+; eyFLP5, Act5C>y+>Gal4, UAS-GFP/UAS-Ras^V12^; M6^W186*^, Tub-Gal80, FRT79E/UAS-tub^RNAi^, FRT79E* |
|  | *y, w/+; eyFLP5, Act5C>y+>Gal4, UAS-GFP/UAS-Ras^V12^, UAS-pll^RNAi^; M6^W186*^, Tub-Gal80, FRT79E/ FRT79E* |
|  | *y, w/+; eyFLP5, Act5C>y+>Gal4, UAS-GFP/UAS-Ras^V12^, UAS-dl^RNAi#1^; M6^W186*^, Tub-Gal80, FRT79E/FRT79E* |
|  | *y, w/+; eyFLP5, Act5C>y+>Gal4, UAS-GFP/UAS-Ras^V12^, UAS-Dif^RNAi#1^; Tub-Gal80, M6^W186*^, FRT79E/FRT79E* |
|  |  |
| **Fig 2I** | *y, w, eyFLP1/w or Y; Act5C>y+>Gal4, UAS-GFP/UAS-Ras^V12^; Tub-Gal80, FRT79E/FRT79E* |
|  | *y, w, eyFLP1/w or Y; Act5C>y+>Gal4, UAS-GFP/UAS-Ras^V12^, UAS-Tl^10B^; Tub-Gal80, FRT79E/FRT79E* |
|  | *y, w, eyFLP1/w or Y; Act5C>y+>Gal4, UAS-GFP/UAS-Ras^V12^; Tub-Gal80, FRT79E/FRT79E, UAS-pll* |
|  | *y, w, eyFLP1/w or Y; Act5C>y+>Gal4, UAS-GFP/UAS-Ras^V12^, UAS-dl-H; Tub-Gal80, FRT79E/FRT79E* |
|  | *y, w, eyFLP1/w or Y; Act5C>y+>Gal4, UAS-GFP/UAS-Ras^V12^, UAS-Dif; Tub-Gal80, FRT79E/FRT79E* |
|  |  |
| **Fig 3**. |  |
| **Fig 3H** | *y, w/+; eyFLP5, Act5C>y+>Gal4, UAS-GFP/ex^e1^, FRT 40A, UAS-Ras^V12^; Tub-Gal80, FRT79E/FRT79E* |
|  | *y, w/+; eyFLP5, Act5C>y+>Gal4, UAS-GFP/ex^e1^, FRT 40A, UAS-Ras^V12^; Tub-Gal80, M6^W186*^, FRT79E/FRT79E* |
|  |  |
| **Fig 3I** | *w67c23, Myc-LacZ^G0354^/w; eyFLP5, Act5C>y+>Gal4, UAS-GFP/UAS-Ras^V12^; Tub-Gal80, FRT79E/FRT79E* |
|  | *w67c23, Myc-LacZ^G0354^/w; eyFLP5, Act5C>y+>Gal4, UAS-GFP/UAS-Ras^V12^; Tub-Gal80, M6^W186*^, FRT79E/FRT79E* |
|  | *y, w/+; eyFLP5, Act5C>y+>Gal4, UAS-GFP/UAS-Ras^V12^; Tub-Gal80, FRT79E/FRT79E* |
|  | *y, w/+; eyFLP5, Act5C>y+>Gal4, UAS-GFP/UAS-Ras^V12^; Tub-Gal80, M6^W186*^, FRT79E/FRT79E* |
|  | *y, w, eyFLP1/w or Y; Act5C>y+>Gal4, UAS-GFP/UAS-Ras^V12^; Tub-Gal80, FRT79E/FRT79E, ban-lacZ,* |
|  | *y, w, eyFLP1/w or Y; Act5C>y+>Gal4, UAS-GFP/UAS-Ras^V12^; Tub-Gal80, M6^W186*^, FRT79E/FRT79E, ban-lacZ* |
|  |  |
| **Fig 3J** | *y, w/+; eyFLP5, Act5C>y+>Gal4, UAS-GFP/UAS-Ras^V12^; Tub-Gal80, FRT79E/FRT79E* |
|  | *y, w/+; eyFLP5, Act5C>y+>Gal4, UAS-GFP/UAS-Ras^V12^, yki^B5^; Tub-Gal80, FRT79E/FRT79E* |
|  | *y, w/+; eyFLP5, Act5C>y+>Gal4, UAS-GFP/UAS-Ras^V12^, UAS-wts; Tub-Gal80, FRT79E/FRT79E* |
|  | *y, w/+; eyFLP5, Act5C>y+>Gal4, UAS-GFP/UAS-Ras^V12^; Tub-Gal80, M6^W186*^, FRT79E/FRT79E* |
|  | *y, w/+; eyFLP5, Act5C>y+>Gal4, UAS-GFP/UAS-Ras^V12^, yki^B5^; Tub-Gal80, M6^W186*^, FRT79E/FRT79E* |
|  | *y, w/+; eyFLP5, Act5C>y+>Gal4, UAS-GFP/UAS-Ras^V12^, UAS-wts; Tub-Gal80, M6^W186*^, FRT79E/FRT79E* |
|  |  |
| **Fig 4**. |  |
| **Fig 4A** | *y, w, eyFLP1/w or Y; Act5C>y+>Gal4, UAS-GFP/UAS-Ras^V12^; Tub-Gal80, FRT79E/FRT79E* |
|  | *y, w, eyFLP1/w or Y; Act5C>y+>Gal4, UAS-GFP/UAS-Ras^V12^; Tub-Gal80, FRT79E/ FRT79E, ban-lacZ* |
|  | *y, w, eyFLP1/w or Y; Act5C>y+>Gal4, UAS-GFP/UAS-Ras^V12^, UAS-Tl^10B^; Tub-Gal80, FRT79E/FRT79E* |
|  | *y, w, eyFLP1/w or Y; Act5C>y+>Gal4, UAS-GFP/UAS-Ras^V12^, UAS-Tl^10B^; Tub-Gal80, FRT79E/FRT79E, ban-lacZ* |
|  | *y, w, eyFLP1/w or Y; Act5C>y+>Gal4, UAS-GFP/UAS-Ras^V12^, UAS-dl-H; Tub-Gal80, FRT79E/FRT79E* |
|  | *y, w, eyFLP1/w or Y; Act5C>y+>Gal4, UAS-GFP/UAS-Ras^V12^, UAS-dl-H; Tub-Gal80, FRT79E/FRT79E, ban-lacZ* |
|  | *y, w, eyFLP1/w or Y; Act5C>y+>Gal4, UAS-GFP/UAS-Ras^V12^, UAS-Dif; Tub-Gal80, FRT79E/FRT79E* |
|  | *y, w, eyFLP1/w or Y; Act5C>y+>Gal4, UAS-GFP/UAS-Ras^V12^, UAS-Dif; Tub-Gal80, FRT79E/FRT79E, ban-lacZ* |
|  |  |
| **Fig 4C** | *y, w, eyFLP1/w or Y; Act5C>y+>Gal4, UAS-GFP/UAS-Ras^V12^, UAS-Tl^10B^; Tub-Gal80, FRT79E/FRT79E* |
|  | *y, w, eyFLP1/w or Y; Act5C>y+>Gal4, UAS-GFP/UAS-Ras^V12^, UAS-Tl^10B^; Tub-Gal80, FRT79E/UAS-wts, FRT79E* |
|  | *y, w, eyFLP1/w or Y; Act5C>y+>Gal4, UAS-GFP/UAS-Ras^V12^, UAS-Tl^10B^; Tub-Gal80, FRT79E/UAS-hpo-Flag, FRT79E* |
|  | *y, w, eyFLP1/w or Y; Act5C>y+>Gal4, UAS-GFP/UAS-Ras^V12^, UAS-dl-H; Tub-Gal80, FRT79E/FRT79E* |
|  | *y, w, eyFLP1/w or Y; Act5C>y+>Gal4, UAS-GFP/UAS-Ras^V12^, UAS-dl-H; Tub-Gal80, FRT79E/UAS-wts, FRT79E* |
|  | *y, w, eyFLP1/w or Y; Act5C>y+>Gal4, UAS-GFP/UAS-Ras^V12^, UAS-dl-H; Tub-Gal80, FRT79E/UAS-hpo-Flag, FRT79E* |
|  | *y, w, eyFLP1/w or Y; Act5C>y+>Gal4, UAS-GFP/UAS-Ras^V12^, UAS-Dif; Tub-Gal80, FRT79E/FRT79E* |
|  | *y, w, eyFLP1/w or Y; Act5C>y+>Gal4, UAS-GFP/UAS-Ras^V12^, UAS-Dif; Tub-Gal80, FRT79E/UAS-wts, FRT79E* |
|  | *y, w, eyFLP1/w or Y; Act5C>y+>Gal4, UAS-GFP/UAS-Ras^V12^, UAS-Dif; Tub-Gal80, FRT79E/UAS-hpo-Flag, FRT79E* |
|  |  |
| **Fig 4F** | *y, w/+; eyFLP5, Act5C>y+>Gal4, UAS-GFP/UAS-Ras^V12^; Tub-Gal80, FRT79E/UAS-yki^S168A^, FRT79E* |
|  | *y, w/+; eyFLP5, Act5C>y+>Gal4, UAS-GFP/UAS-Ras^V12^, UAS-Tl^RNAi^; Tub-Gal80, FRT79E /UAS-yki^S168A^, FRT79E* |
|  | *y, w/+; eyFLP5, Act5C>y+>Gal4, UAS-GFP/UAS-Ras^V12^, UAS-dl^RNAi#1^; Tub-Gal80, FRT79E /UAS-yki^S168A^, FRT79E* |
|  | *y, w/+; eyFLP5, Act5C>y+>Gal4, UAS-GFP/UAS-Ras^V12^, UAS-Dif^RNAi#1^; Tub-Gal80, FRT79E/UAS-yki^S168A^, FRT79E* |
|  |  |
| **Fig 4H** | *y, w/+; eyFLP5, Act5C>y+>Gal4, UAS-GFP/UAS-Ras^V12^; Tub-Gal80, FRT79E/FRT79E* |
|  | *y, w/+; eyFLP5, Act5C>y+>Gal4, UAS-GFP/UAS-Ras^V12^; Tub-Gal80, M6^W186*^, FRT79E/FRT79E* |
|  | *UAS-bsk^DN^/y, w; eyFLP5, Act5C>y+>Gal4, UAS-GFP/UAS-Ras^V12^; Tub-Gal80, M6^W186*^, FRT79E/FRT79E* |
|  |  |
| **Fig 4J** | *y, w/+; eyFLP5, Act5C>y+>Gal4, UAS-GFP/ex^e1^, FRT 40A, UAS-Ras^V12^; Tub-Gal80, M6^W186*^, FRT79E/FRT79E* |
|  | *UAS-bsk^DN^/y, w; eyFLP5, Act5C>y+>Gal4, UAS-GFP/ex^e1^, FRT 40A, UAS-RasV12; Tub-Gal80, M6^W186*^, FRT79E/FRT79E* |
|  |  |
| **Fig 5**. |  |
| **Fig 5A** | *y, w/+; eyFLP5, Act5C>y+>Gal4, UAS-GFP/UAS-Ras^V12^; Tub-Gal80, FRT79E/FRT79E* |
|  | *y, w/+; eyFLP5, Act5C>y+>Gal4, UAS-GFP/UAS-Ras^V12^; Tub-Gal80, M6^W186*^, FRT79E/FRT79E* |
|  |  |
| **Fig 5B** | *y, w/+; eyFLP5, Act5C>y+>Gal4, UAS-GFP/UAS-Ras^V12^; Tub-Gal80, FRT79E/FRT79E* |
|  | *y, w/+; eyFLP5, Act5C>y+>Gal4, UAS-GFP/UAS-Ras^V12^; Tub-Gal80, M6^W186*^, FRT79E/FRT79E* |
|  | *y, w/+; eyFLP5, Act5C>y+>Gal4, UAS-GFP/UAS-Ras^V12^; Tub-Gal80, M6^W186*^, FRT79E, spz^2^/FRT79E, spz^2^* |
|  | *y, w/+; eyFLP5, Act5C>y+>Gal4, UAS-GFP/UAS-Ras^V12^, UAS-Tl^10B^; Tub-Gal80, M6^W186*^, FRT79E, spz^2^/FRT79E, spz^2^* |
|  | *y, w/+; eyFLP5, Act5C>y+>Gal4, UAS-GFP/UAS-Ras^V12^, UAS-dl-H; Tub-Gal80, M6^W186*^, FRT79E, spz^2^/FRT79E, spz^2^* |
|  | *y, w/+; eyFLP5, Act5C>y+>Gal4, UAS-GFP/UAS-Ras^V12^ UAS-Dif; Tub-Gal80, M6^W186*^, FRT79E, spz^2^/FRT79E, spz^2^* |
|  |  |
| **Fig 5D** | *y, w/+; eyFLP5, Act5C>y+>Gal4, UAS-GFP/UAS-Ras^V12^; Tub-Gal80, M6^W186*^, FRT79E /FRT79E* |
|  | *y, w/+; eyFLP5, Act5C>y+>Gal4, UAS-GFP/UAS-Ras^V12^; Tub-Gal80, M6^W186*^, FRT79E, spz^2^/FRT79E, spz^2^* |
|  |  |
| **Fig 5E** | *y, w/+; eyFLP5, Act5C>y+>Gal4, UAS-GFP/UAS-Ras^V12^; Tub-Gal80, FRT79E/FRT79E* |
|  | *y, w/+; eyFLP5, Act5C>y+>Gal4, UAS-GFP/UAS-Ras^V12^; Tub-Gal80, M6^W186*^, FRT79E/FRT79E* |
|  |  |
| **Fig 5H** | *y, w/+; eyFLP5, Act5C>y+>Gal4, UAS-GFP/UAS-Ras^V12^; Tub-Gal80, M6^W186*^, FRT79E/FRT79E* |
|  |  |
| **Fig 6**. |  |
| **Fig 6B** | *y, w, eyFLP1/w or Y; Tub-QF, QUAS-mCD8-GFP/+; Tub-QS, FRT79E, He-Gal4/QUAS-Ras^V12^, FRT79E* |
|  | *y, w, eyFLP1/w or Y; Tub-QF, QUAS-mCD8-GFP/+; Tub-QS, M6^W186*^, FRT79E, He-Gal4/QUAS-Ras^V12^, FRT79E* |
|  | *y, w, eyFLP1/w or Y; Tub-QF, QUAS-mCD8-GFP/UAS-spz^RNAi^; Tub-QS, M6^W186*^, FRT79E, He-Gal4/QUAS-Ras^V12^, FRT79E* |
|  |  |
| **Fig 6E** | *y, w, eyFLP1/w or Y; Tub-QF, QUAS-mCD8-GFP/+; FRT82B, Tub-QS, He-Gal4/QUAS-Ras^V12^, FRT82B* |
|  | *y, w, eyFLP1/w or Y; Tub-QF, QUAS-mCD8-GFP/+; FRT82B, Tub-QS, He-Gal4/QUAS-Ras^V12^, FRT82B, UAS-spz^ACT^* |
| **Fig 6G** |  |
| **Donor** | *y, w/+; eyFLP5, Act5C>y+>Gal4, UAS-GFP/UAS-Ras^V12^; Tub-Gal80, FRT79E/FRT79E* |
|  | *y, w/+; eyFLP5, Act5C>y+>Gal4, UAS-GFP/UAS-Ras^V12^; Tub-Gal80, M6^W186*^, FRT79E/FRT79E* |
| **Host** | *y, w/+; +/+; Hml-Gal4/+* |
|  | *y, w/+; +/+; Hml-Gal4/UAS-spz^RNAi^* |
|  | *y, w/+; +/+; Hml-Gal4/UAS-rpr* |
|  | *y, w/+; UAS-hid/+; Hml-Gal4/+* |
|  |  |
| **Fig 7**. |  |
| **Fig 7H** |  |
|  | *y, w/+; eyFLP5, Act5C>y+>Gal4, UAS-GFP/UAS-Ras^V12^; Tub-Gal80, M6^W186*^, FRT79E /FRT79E* |
|  | *y, w/+; eyFLP5, Act5C>y+>Gal4, UAS-GFP/UAS-Ras^V12^, UAS-Ets21C^RNAi#1^; Tub-Gal80, M6^W186*^, FRT79E/FRT79E* |
|  | *y, w/+; eyFLP5, Act5C>y+>Gal4, UAS-GFP/UAS-Ras^V12^; Tub-Gal80, M6^W186*^, FRT79E/UAS-Ets21C^RNAi#2^, FRT79E* |
|  | *y, w/+; eyFLP5, Act5C>y+>Gal4, UAS-GFP/UAS-Ras^V12^; Tub-Gal80, M6^W186*^, FRT79E/UAS-Pvf1^RNAi#1^, FRT79E* |
|  | *y, w/+; eyFLP5, Act5C>y+>Gal4, UAS-GFP/UAS-Ras^V12^; Tub-Gal80, M6^W186*^, FRT79E/UAS-Pvf2^RNAi#1^, FRT79E* |
| **Fig 7I** |  |
|  | *y, w, eyFLP1/w or Y; Tub-QF, QUAS-mCD8-GFP/+; Tub-QS, FRT79E, He-Gal4/QUAS-Ras^V12^, FRT79E* |
|  | *y, w, eyFLP1/w or Y; Tub-QF, QUAS-mCD8-GFP/UAS-Pvr^DN^; Tub-QS, FRT79E, He-Gal4/QUAS-Ras^V12^, FRT79E* |
|  | *y, w, eyFLP1/w or Y; Tub-QF, QUAS-mCD8-GFP/+; Tub-QS, M6^W186*^, FRT79E, He-Gal4/QUAS-Ras^V12^, FRT79E* |
|  | *y, w, eyFLP1/w or Y; Tub-QF, QUAS-mCD8-GFP/UAS-Pvr^DN^; Tub-QS, M6^W186*^, FRT79E, He-Gal4/QUAS-Ras^V12^, FRT79E* |
|  |  |
| **Fig EV1.** |  |
| **Fig EV1A** | *y, w/+; eyFLP5, Act5C>y+>Gal4, UAS-GFP/+; Tub-Gal80, FRT79E/FRT79E* |
|  | *y, w/+; eyFLP5, Act5C>y+>Gal4, UAS-GFP/+; Tub-Gal80, FRT79E/M6^W186*^, FRT79E* |
|  |  |
| **Fig EV1B** | *y, w/+; eyFLP5, Act5C>y+>Gal4, UAS-GFP/+; Tub-Gal80, FRT79E/FRT79E* |
|  | *y, w/+; eyFLP5, Act5C>y+>Gal4, UAS-GFP/+; Tub-Gal80, FRT79E/M6^W186*^, FRT79E* |
|  | *y, w/+; eyFLP5, Act5C>y+>Gal4, UAS-GFP/UAS-p35; Tub-Gal80, FRT79E/M6^W186*^, FRT79E* |
|  |  |
| **Fig EV1C** | *y, w, eyFLP1/w or Y; Tub-QF, QUAS-mCD8-GFP, Act5C>y+>Gal4/+; Tub-QS, FRT79E/QUAS-Ras^V12^, Tub-Gal80, FRT79E* |
|  | *y, w, eyFLP1/w or Y; Tub-QF, QUAS-mCD8-GFP, Act5C>y+>Gal4/+; Tub-QS, M6^W186*^, FRT79E/QUAS-Ras^V12^, Tub-Gal80, FRT79E* |
|  | *y, w, eyFLP1/w or Y; Tub-QF, QUAS-mCD8-GFP, Act5C>y+>Gal4/UAS-M6; Tub-QS, M6^W186*^, FRT79E/QUAS-Ras^V12^, Tub-Gal80, FRT79E* |
|  |  |
| **Fig EV1F** | *y, w/+; eyFLP5, Act5C>y+>Gal4, UAS-GFP/UAS-Ras^V12^; Tub-Gal80, FRT79E/FRT79E* |
|  | *y, w/+; eyFLP5, Act5C>y+>Gal4, UAS-GFP/UAS-Ras^V12^; Tub-Gal80, M6^W186*^, FRT79E/FRT79E* |
|  |  |
| **Fig EV1H** | *y, w/+; eyFLP5, Act5C>y+>Gal4, UAS-GFP/UAS-Ras^V12^; Tub-Gal80, FRT79E/FRT79E* |
|  | *y, w/+; eyFLP5, Act5C>y+>Gal4, UAS-GFP/UAS-Ras^V12^; Tub-Gal80, M6^W186*^, FRT79E/FRT79E* |
|  |  |
| **Fig EV1K** | *y, w/+; eyFLP5, Act5C>y+>Gal4, UAS-GFP/UAS-Ras^V12^; Tub-Gal80, M6^W186*^, FRT79E/FRT79E* |
|  | *y, w/+; eyFLP5, Act5C>y+>Gal4, UAS-GFP/Def^SK3^; Tub-Gal80, M6^W186*^, FRT79E/FRT79E, UAS-Ras^V12^* |
|  | *y, w/+; eyFLP5, Act5C>y+>Gal4, UAS-GFP/Def^SK3^, AttC^MI^, Dro-Att^SK2^, Dpt^SK1^; Tub-Gal80, M6^W186*^, FRT79E/FRT79E, UAS-Ras^V12^* |
|  |  |
| **Fig EV2.** |  |
| **Fig EV2A** | *y, w/+; eyFLP5, Act5C>y+>Gal4, UAS-GFP/+; Tub-Gal80, FRT79E/FRT79E* |
|  | *y, w/+; eyFLP5, Act5C>y+>Gal4, UAS-GFP/UAS-dl^RNAi#1^; Tub-Gal80, FRT79E /FRT79E* |
|  | *y, w/+; eyFLP5, Act5C>y+>Gal4, UAS-GFP/+; Tub-Gal80, FRT79E /FRT79E, UAS-dl^RNAi#2^* |
|  |  |
| **Fig EV2C** | *y, w/+; eyFLP5, Act5C>y+>Gal4, UAS-GFP/+; Tub-Gal80, FRT79E/FRT79E* |
|  | *y, w/+; eyFLP5, Act5C>y+>Gal4, UAS-GFP/UAS-Tl; Tub-Gal80, FRT79E/FRT79E* |
|  | *y, w/+; eyFLP5, Act5C>y+>Gal4, UAS-GFP/+; Tub-Gal80, FRT79E/FRT79E, UAS-pll* |
|  | *y, w/+; eyFLP5, Act5C>y+>Gal4, UAS-GFP/UAS-dl; Tub-Gal80, FRT79E /FRT79E* |
|  | *y, w/+; eyFLP5, Act5C>y+>Gal4, UAS-GFP/ UAS-Dif; Tub-Gal80, FRT79E/FRT79E* |
|  | *y, w/+; eyFLP5, Act5C>y+>Gal4, UAS-GFP/UAS-Tl^RNAi^; Tub-Gal80, FRT79E/FRT79E* |
|  | *y, w/+; eyFLP5, Act5C>y+>Gal4, UAS-GFP/+; Tub-Gal80, FRT79E/UAS-tub^RNAi^, FRT79E* |
|  | *y, w/+; eyFLP5, Act5C>y+>Gal4, UAS-GFP/UAS-pll^RNAi^; Tub-Gal80, FRT79E/FRT79E* |
|  | *y, w/+; eyFLP5, Act5C>y+>Gal4, UAS-GFP/UAS-dl^RNAi#1^; Tub-Gal80, FRT79E /FRT79E* |
|  | *y, w/+; eyFLP5, Act5C>y+>Gal4, UAS-GFP/UAS-Dif^RNAi#1^; Tub-Gal80, FRT79E/FRT79E* |
|  |  |
| **Fig EV2E** | *y, w/+; eyFLP5, Act5C>y+>Gal4, UAS-GFP/+; Tub-Gal80, M6^W186*^, FRT79E/FRT79E* |
|  | *y, w/+; eyFLP5, Act5C>y+>Gal4, UAS-GFP/UAS-Tl; Tub-Gal80, M6^W186^*, FRT79E/FRT79E* |
|  | *y, w/+; eyFLP5, Act5C>y+>Gal4, UAS-GFP/+; Tub-Gal80, M6^W186*^, FRT79E/FRT79E, UAS-pll* |
|  | *y, w/+; eyFLP5, Act5C>y+>Gal4, UAS-GFP/UAS-dl; Tub-Gal80, M6^W186^*, FRT79E/FRT79E* |
|  | *y, w/+; eyFLP5, Act5C>y+>Gal4, UAS-GFP/ UAS-Dif; Tub-Gal80, M6^W186*^, FRT79E/FRT79E* |
|  | *y, w/+; eyFLP5, Act5C>y+>Gal4, UAS-GFP/UAS-Tl^RNAi^; Tub-Gal80, M6^W186*^, FRT79E/FRT79E* |
|  | *y, w/+; eyFLP5, Act5C>y+>Gal4, UAS-GFP/+; Tub-Gal80, M6^W186^*, FRT79E/UAS-tub^RNAi^, FRT79E* |
|  | *y, w/+; eyFLP5, Act5C>y+>Gal4, UAS-GFP/ UAS-pll^RNAi^; Tub-Gal80, M6^W186^*, FRT79E/ FRT79E* |
|  | *y, w/+; eyFLP5, Act5C>y+>Gal4, UAS-GFP/UAS-dl^RNAi#1^; Tub-Gal80, M6^W186*^, FRT79E/FRT79E* |
|  | *y, w/+; eyFLP5, Act5C>y+>Gal4, UAS-GFP/ UAS-Dif^RNAi#1^; Tub-Gal80, M6^W186*^, FRT79E/FRT79E* |
|  |  |
| **Fig EV2G** | *y, w, eyFLP1/w or Y; Act5C>y+>Gal4, UAS-GFP/UAS-Ras^V12^; Tub-Gal80, FRT79E/FRT79E* |
|  | *y, w, eyFLP1/w or Y; Act5C>y+>Gal4, UAS-GFP/UAS-Ras^V12^, UAS-Tl^10B^; Tub-Gal80, FRT79E/FRT79E* |
|  | *y, w, eyFLP1/w or Y; Act5C>y+>Gal4, UAS-GFP/UAS-Ras^V12^, UAS-dl-H; Tub-Gal80, FRT79E/FRT79E* |
|  | *y, w, eyFLP1/w or Y; Act5C>y+>Gal4, UAS-GFP/UAS-Ras^V12^, UAS-Dif; Tub-Gal80, FRT79E/FRT79E* |
|  |  |
| **Fig EV2I** | *y, w/+; eyFLP5, Act5C>y+>Gal4, UAS-GFP/UAS-Ras^V12^; Tub-Gal80, FRT79E/FRT79E* |
|  | *y, w/+; eyFLP5, Act5C>y+>Gal4, UAS-GFP/UAS-Ras^V12^; Tub-Gal80, M6^W186*^, FRT79E/FRT79E* |
|  | *y, w/+; eyFLP5, Act5C>y+>Gal4, UAS-GFP/UAS-Ras^V12^, UAS-Rel^RNAi^; Tub-Gal80, FRT79E/FRT79E* |
|  | *y, w/+; eyFLP5, Act5C>y+>Gal4, UAS-GFP/UAS-Ras^V12^, UAS-Rel^RNAi^; Tub-Gal80, M6^W186*^, FRT79E/FRT79E* |
|  |  |
| **Fig EV3.** |  |
| **Fig EV3C** | *y, w/+; eyFLP5, Act5C>y+>Gal4, UAS-GFP/UAS-Ras^V12^; Tub-Gal80, FRT79E/FRT79E* |
|  | *y, w/+; eyFLP5, Act5C>y+>Gal4, UAS-GFP/UAS-Ras^V12^; Tub-Gal80, M6^W186*^, FRT79E/FRT79E* |
|  | *y, w/+; eyFLP5, Act5C>y+>Gal4, UAS-GFP/UAS-Ras^V12^; Tub-Gal80, FRT79E/FRT79E* |
|  | *y, w/+; eyFLP5, Act5C>y+>Gal4, UAS-GFP/UAS-Ras^V12^; Tub-Gal80, M6^W186*^, FRT79E/FRT79E* |
|  | *y, w/+; eyFLP5, Act5C>y+>Gal4, UAS-GFP/fj-lacZ, UAS-Ras^V12^; Tub-Gal80, FRT79E/FRT79E* |
|  | *y, w/+; eyFLP5, Act5C>y+>Gal4, UAS-GFP/fj-lacZ, UAS-Ras^V12^; Tub-Gal80, M6^W186*^, FRT79E/FRT79E* |
|  |  |
| **Fig EV3F** | *y, w/+; eyFLP5, Act5C>y+>Gal4, UAS-GFP/+; Tub-Gal80, FRT79E/rpr-lacZ, FRT79E* |
|  | *y, w/+; eyFLP5, Act5C>y+>Gal4, UAS-GFP/UAS-Ras^V12^; Tub-Gal80, M6^W186*^, FRT79E/rpr-lacZ, FRT79E* |
|  |  |
| **Fig EV4.** |  |
| **Fig EV4A** | *y, w, eyFLP1/w or Y; Act5C>y+>Gal4, UAS-GFP/UAS-Ras^V12^; Tub-Gal80, FRT79E/FRT79E* |
|  | *y, w, eyFLP1/w or Y; Act5C>y+>Gal4, UAS-GFP/UAS-Ras^V12^, UAS-Tl^10B^; Tub-Gal80, FRT79E/FRT79E* |
|  | *y, w, eyFLP1/w or Y; Act5C>y+>Gal4, UAS-GFP/UAS-Ras^V12^, UAS-dl-H; Tub-Gal80, FRT79E/FRT79E* |
|  | *y, w, eyFLP1/w or Y; Act5C>y+>Gal4, UAS-GFP/UAS-Ras^V12^, UAS-Dif; Tub-Gal80, FRT79E/FRT79E* |
|  |  |
| **Fig EV4B** | *y, w, eyFLP1/w or Y; Act5C>y+>Gal4, UAS-GFP/+; Tub-Gal80, FRT79E/FRT79E* |
|  | *y, w, eyFLP1/w or Y; Act5C>y+>Gal4, UAS-GFP/UAS-dl-H; Tub-Gal80, FRT79E/FRT79E* |
|  | *y, w, eyFLP1/w or Y; Act5C>y+>Gal4, UAS-GFP/UAS-Dif; Tub-Gal80, FRT79E/FRT79E* |
|  |  |
| **Fig EV4D** | *y, w, eyFLP1/w or Y; Act5C>y+>Gal4, UAS-GFP/+; Tub-Gal80, FRT79E, puc^[E69]^/FRT79E* |
|  | *y, w, eyFLP1/w or Y; Act5C>y+>Gal4, UAS-GFP/UAS-dl-H; Tub-Gal80, FRT79E, puc^[E69]^/FRT79E* |
|  | *y, w, eyFLP1/w or Y; Act5C>y+>Gal4, UAS-GFP/UAS-Dif; Tub-Gal80, FRT79E, puc^[E69]^/FRT79E* |
|  | *y, w, eyFLP1/w or Y; Act5C>y+>Gal4, UAS-GFP/UAS-Ras^V12^; Tub-Gal80, FRT79E, puc^[E69]^/FRT79E* |
|  | *y, w, eyFLP1/w or Y; Act5C>y+>Gal4, UAS-GFP/UAS-Ras^V12^, UAS-dl-H; Tub-Gal80, FRT79E, puc^[E69]^/FRT79E* |
|  | *y, w, eyFLP1/w or Y; Act5C>y+>Gal4, UAS-GFP/UAS-Ras^V12^, UAS-Dif; Tub-Gal80, FRT79E, puc^[E69]^/FRT79E* |
|  | *y, w, eyFLP1/w or Y; Act5C>y+>Gal4, UAS-GFP/+; Tub-Gal80, FRT79E/FRT79E* |
|  | *y, w, eyFLP1/w or Y; Act5C>y+>Gal4, UAS-GFP/UAS-dl-H; Tub-Gal80, FRT79E/FRT79E* |
|  | *y, w, eyFLP1/w or Y; Act5C>y+>Gal4, UAS-GFP/UAS-Dif; Tub-Gal80, FRT79E/FRT79E* |
|  | *y, w, eyFLP1/w or Y; Act5C>y+>Gal4, UAS-GFP/UAS-Ras^V12^; Tub-Gal80, FRT79E/FRT79E* |
|  | *y, w, eyFLP1/w or Y; Act5C>y+>Gal4, UAS-GFP/UAS-Ras^V12^, UAS-dl-H; Tub-Gal80, FRT79E/FRT79E* |
|  | *y, w, eyFLP1/w or Y; Act5C>y+>Gal4, UAS-GFP/UAS-Ras^V12^, UAS-Dif; Tub-Gal80, FRT79E/FRT79E* |
|  |  |
|  |  |
| **Fig EV5.** |  |
| **Fig EV5A** | *y, w/+; eyFLP5, Act5C>y+>Gal4, UAS-GFP/+; Tub-Gal80, FRT79E/FRT79E* |
|  | *y, w/+; eyFLP5, Act5C>y+>Gal4, UAS-GFP/+; Tub-Gal80, FRT79E/M6^W186*^, FRT79E* |
|  | *y, w/+; eyFLP5, Act5C>y+>Gal4, UAS-GFP/+; Tub-Gal80, FRT79E/FRT79E, UAS-spz^ACT^* |
|  |  |
| **Fig EV5B** | *y, w/+; eyFLP5, Act5C>y+>Gal4, UAS-GFP/UAS-Ras^V12^; Tub-Gal80, M6^W186*^, FRT79E/FRT79E* |
|  | *y, w/+; eyFLP5, Act5C>y+>Gal4, UAS-GFP/UAS-Ras^V12^; Tub-Gal80, M6^W186*^, FRT79E, spz^2^/FRT79E, spz^2^* |
|  |  |
| **Fig EV5F** | *y, w/+; eyFLP5, Act5C>y+>Gal4, UAS-GFP/+; Tub-Gal80, FRT79E/FRT79E* |
|  | *y, w/+; eyFLP5, Act5C>y+>Gal4, UAS-GFP/+; Tub-Gal80, FRT79E/M6^W186*^, FRT79E* |
|  |  |
| **Fig EV5H** | *y, w/+; eyFLP5, Act5C>y+>Gal4, UAS-GFP/UAS-Ras^V12^; Tub-Gal80, FRT79E/FRT79E* |
|  | *y, w/+; eyFLP5, Act5C>y+>Gal4, UAS-GFP/UAS-Ras^V12^; Tub-Gal80, M6^W186*^, FRT79E/FRT79E* |
|  |  |
| **Fig EV6.** |  |
| **Fig EV6B** | *y, w, eyFLP1/w or Y; Tub-QF, QUAS-mCD8-GFP, Cg-Gal4/+; FRT82B, Tub-QS/FRT82B, UAS-spz^ACT^* |
|  | *y, w, eyFLP1/w or Y; Tub-QF, QUAS-mCD8-GFP, Cg-Gal4/+; FRT82B, Tub-QS/QUAS-Ras^V12^, FRT82B* |
|  | *y, w, eyFLP1/w or Y; Tub-QF, QUAS-mCD8-GFP, Cg-Gal4/+; FRT82B, Tub-QS/QUAS-Ras^V12^, FRT82B, UAS-spz^ACT^* |
|  |  |
| **Fig EV6E** | *y, w, eyFLP1/w or Y; Tub-QF, QUAS-mCD8-GFP, Cg-Gal4/+; FRT82B, Tub-QS/QUAS-Ras^V12^, FRT82B* |
|  | *y, w, eyFLP1/w or Y; Tub-QF, QUAS-mCD8-GFP, Cg-Gal4/+; FRT82B, Tub-QS/QUAS-Ras^V12^, FRT82B, UAS-spz^ACT^* |
|  |  |
| **Fig EV6F** | *y, w, eyFLP1/w or Y; Tub-QF, QUAS-mCD8-GFP, Cg-Gal4/+; FRT82B, Tub-QS/QUAS-Ras^V12^, FRT82B* |
|  | *y, w, eyFLP1/w or Y; Tub-QF, QUAS-mCD8-GFP, Cg-Gal4/+; FRT82B, Tub-QS/QUAS-Ras^V12^, FRT82B, UAS-spz^ACT^* |
|  |  |
| **Fig EV6J** | *y, w, eyFLP1/w or Y; Tub-QF, QUAS-mCD8-GFP/+; FRT82B, Tub-QS, He-Gal4/QUAS-Ras^V12^, FRT82B* |
|  | *y, w, eyFLP1/w or Y; Tub-QF, QUAS-mCD8-GFP/+; FRT82B, Tub-QS, He-Gal4/QUAS-Ras^V12^, FRT82B, UAS-spz^ACT^* |
|  |  |
| **Fig EV7.** |  |
| **Fig EV7H** | *y, w/+; eyFLP5, Act5C>y+>Gal4, UAS-GFP/+; Tub-Gal80, FRT79E/FRT79E, UAS-Ras^V12^* |
|  | *y, w/+; eyFLP5, Act5C>y+>Gal4, UAS-GFP/UAS-Pvf1^RNAi#2^; Tub-Gal80, M6^W186*^, FRT79E/FRT79E, UAS-Ras^V12^* |
|  | *y, w/+; eyFLP5, Act5C>y+>Gal4, UAS-GFP/UAS-Pvf2^RNAi#2^; Tub-Gal80, M6^W186*^, FRT79E/FRT79E, UAS-Ras^V12^* |
|  |  |
| **Fig EV7J** | *y, w/+; eyFLP5, Act5C>y+>Gal4, UAS-GFP/+; Tub-Gal80, FRT79E/FRT79E* |
|  | *y, w/+; eyFLP5, Act5C>y+>Gal4, UAS-GFP/UAS-Ets21C^RNAi#1^; Tub-Gal80, FRT79E/FRT79E* |
|  | *y, w/+; eyFLP5, Act5C>y+>Gal4, UAS-GFP/+; Tub-Gal80, FRT79E/UAS-Ets21C^RNAi#2^, FRT79E* |
|  | *y, w/+; eyFLP5, Act5C>y+>Gal4, UAS-GFP/+; Tub-Gal80, FRT79E/UAS-Pvf1^RNAi#1^, FRT79E* |
|  | *y, w/+; eyFLP5, Act5C>y+>Gal4, UAS-GFP/+; Tub-Gal80, FRT79E/UAS-Pvf2^RNAi#2^, FRT79E* |
|  |  |
| **Fig EV8.** |  |
| **Fig EV8C** |  |
| **Donor** | *y, w/+; eyFLP5, Act5C>y+>Gal4, UAS-GFP/UAS-Ras^V12^; Tub-Gal80, FRT79E/FRT79E* |
|  | *y, w/+; eyFLP5, Act5C>y+>Gal4, UAS-GFP/UAS-Ras^V12^; Tub-Gal80, M6^W186*^, FRT79E/FRT79E* |
| **Host** | *y, w/+; +/+; Hml-Gal4/+* |
|  | *y, w/+; UAS-Pvr^DN^/+; Hml-Gal4/+* |
