# Supplementary material for: Hemocytes facilitate interclonal cooperation-induced tumor malignancy by hijacking the innate immune system in Drosophila
Source: EMBO J. 2025 Aug 22;44(19):5394–428. doi: 10.1038/s44318-025-00547-5 (PMC12489090; doi:10.1038/s44318-025-00547-5)
Supplement: Supplementary file 4 — Movie EV1 [file 44318_2025_547_MOESM4_ESM.zip › Movie EV1/EMBOJ-2025-120470_MovieEV1-movie legend.docx]

**Movie EV1: In *Ras^V12^//M6^-/-^* tumors, Spz-positive hemocytes adhere to GFP-positive *Ras^V12^* clones.**

(Related to Figure 5H).

3D imaging of *Ras^V12^//M6^-/-^* tumors: In the heterogeneous tumor composed of GFP-positive *Ras^V12^* clones and GFP-negative *M6* mutant clones, NimC1-positive hemocytes (purple) adhere to GFP-positive *Ras^V12^* clones, with NimC1 colocalizing with Spz (red).
