# Supplementary figures and images for: Hemocytes facilitate interclonal cooperation-induced tumor malignancy by hijacking the innate immune system in Drosophila (part 1 of 4)
Source: EMBO J. 2025 Aug 22;44(19):5394–428. doi: 10.1038/s44318-025-00547-5 (PMC12489090; doi:10.1038/s44318-025-00547-5)

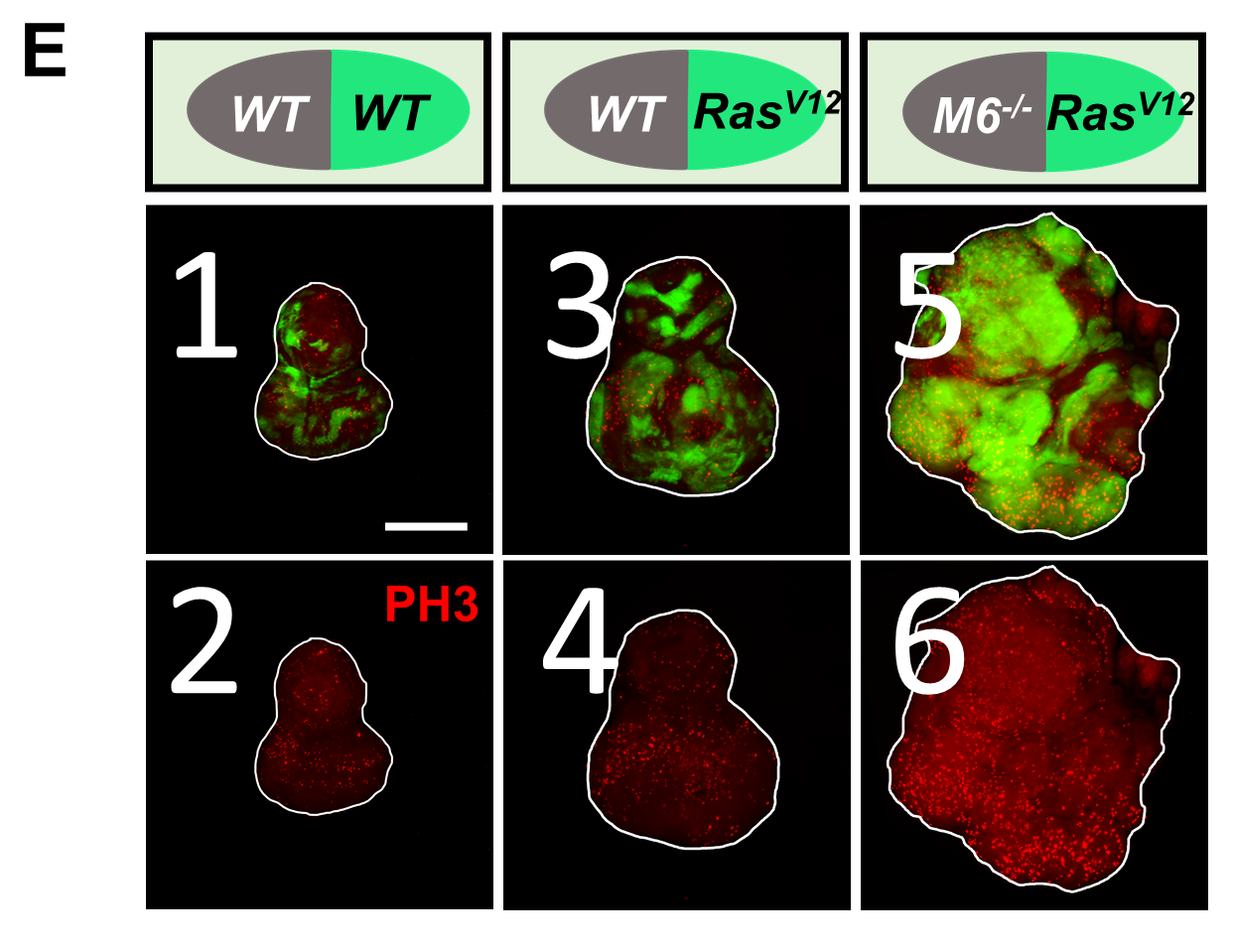

Supplement: Supplementary file 5 — Source data Fig. 1 [file 44318_2025_547_MOESM5_ESM.zip › Figure 1E/0 paper Figure 1E with provided image sequence.tif]

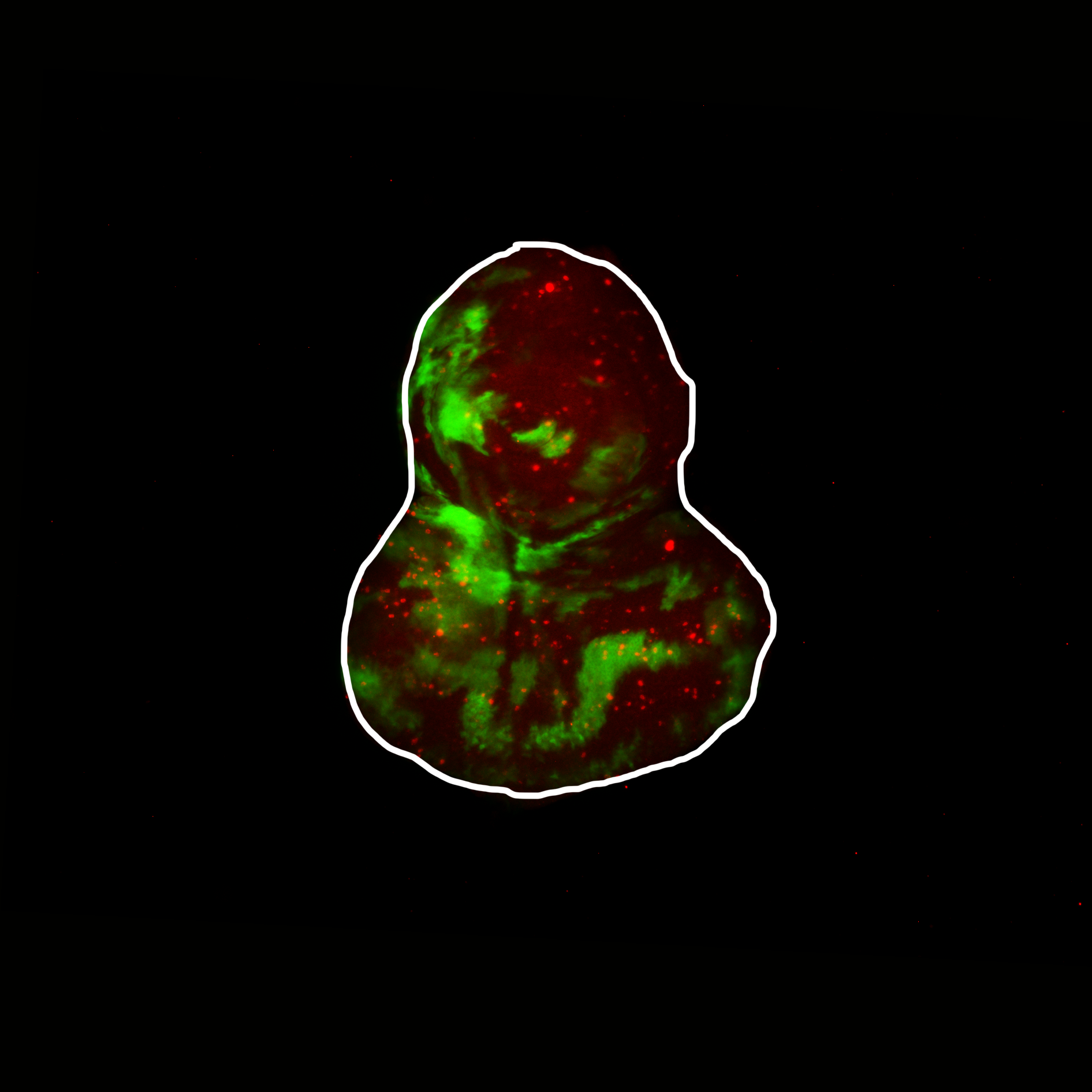

Supplement: Supplementary file 5 — Source data Fig. 1 [file 44318_2025_547_MOESM5_ESM.zip › Figure 1E/1-1 rotated and cut image with border line.tif]

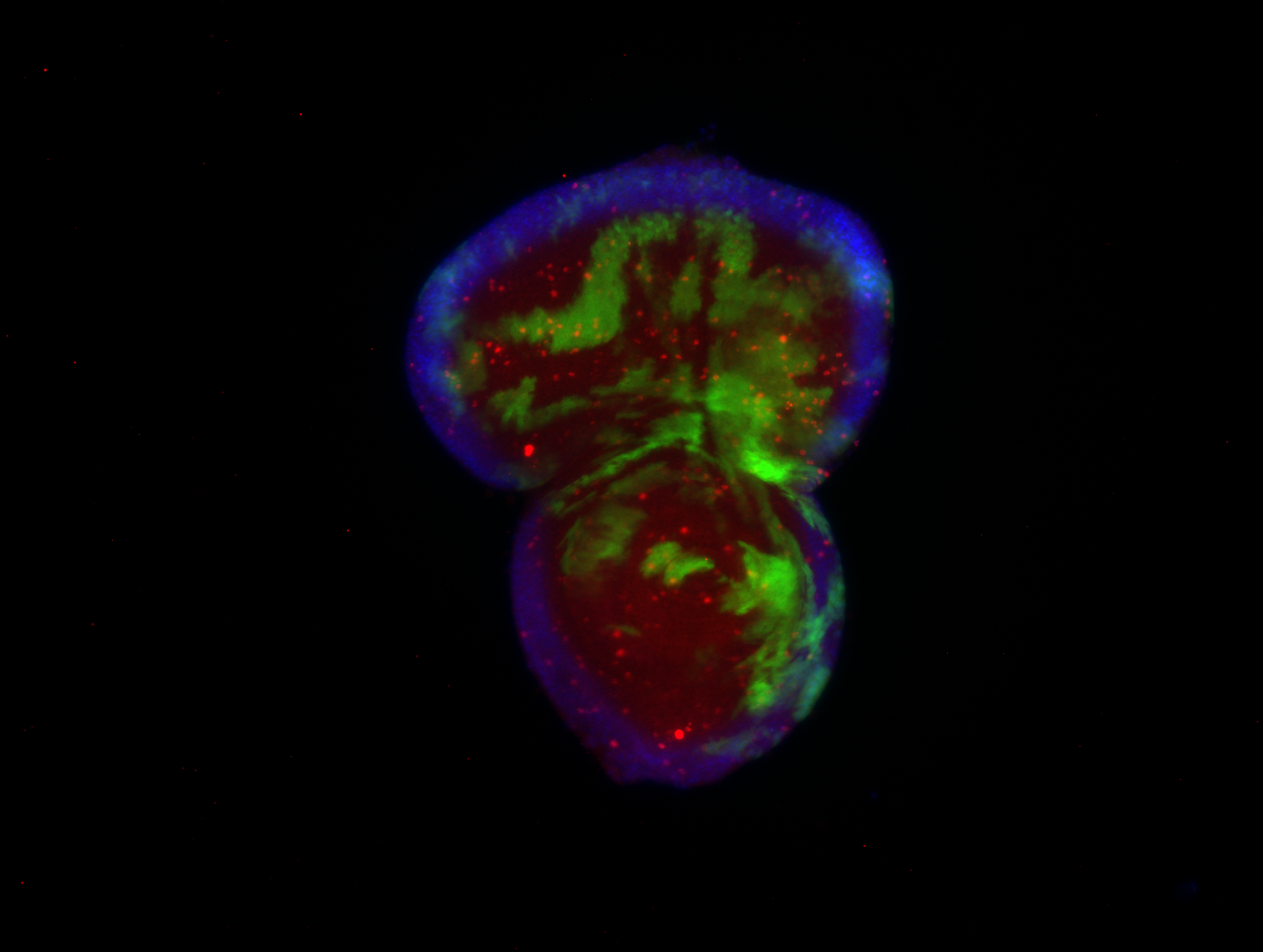

Supplement: Supplementary file 5 — Source data Fig. 1 [file 44318_2025_547_MOESM5_ESM.zip › Figure 1E/1-2 original image.tif]

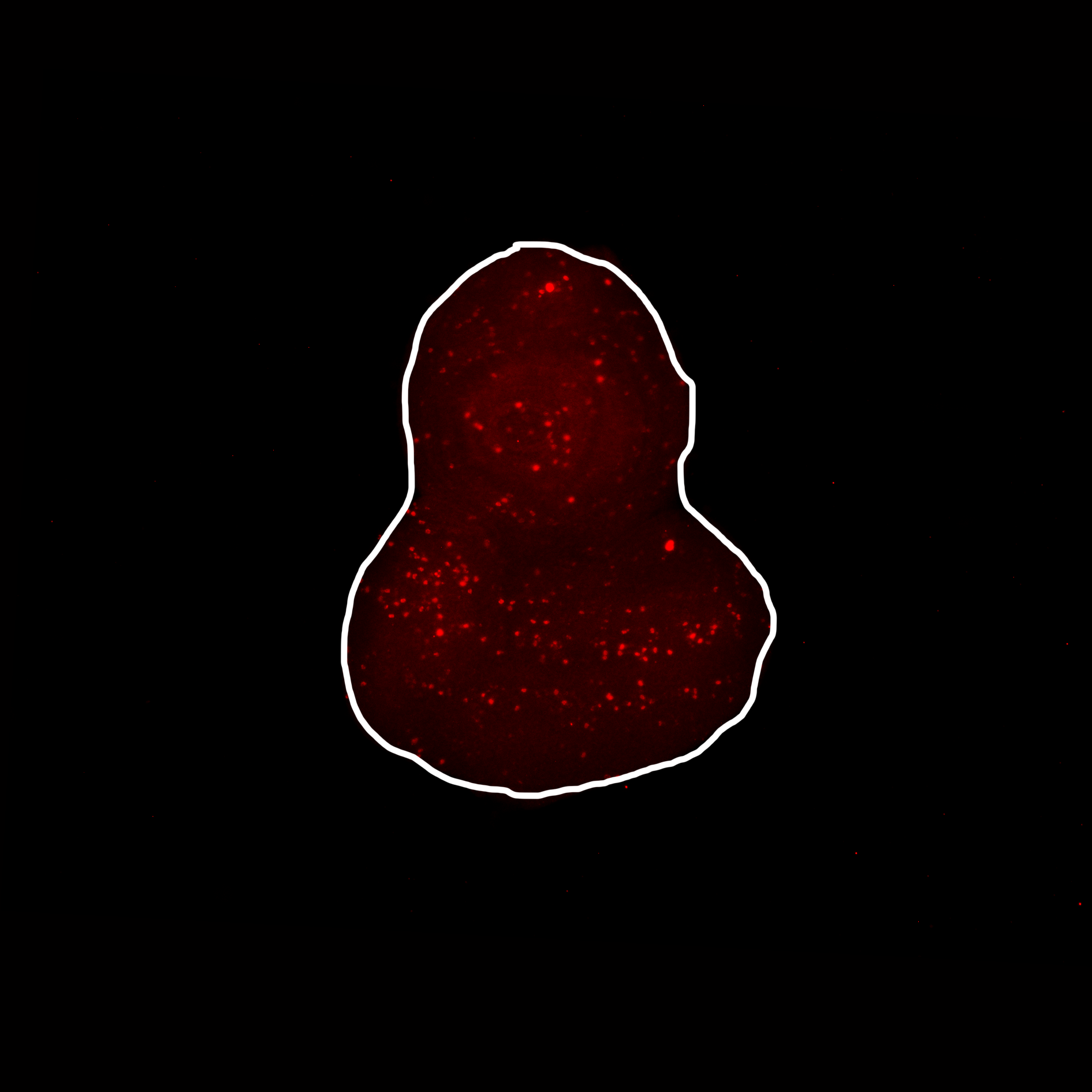

Supplement: Supplementary file 5 — Source data Fig. 1 [file 44318_2025_547_MOESM5_ESM.zip › Figure 1E/2-1 rotated and cut image with border line.tif]

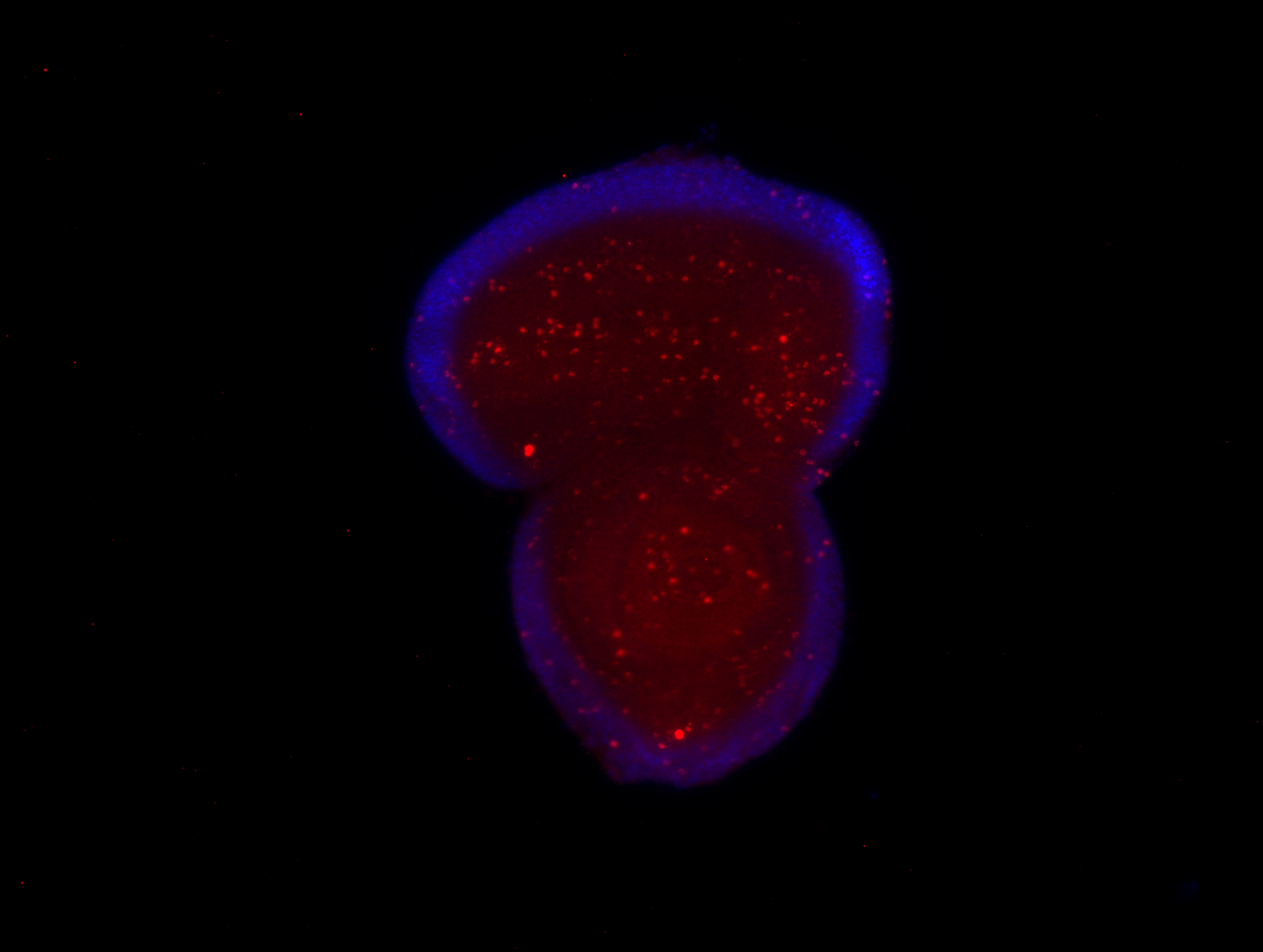

Supplement: Supplementary file 5 — Source data Fig. 1 [file 44318_2025_547_MOESM5_ESM.zip › Figure 1E/2-2 original image.tif]

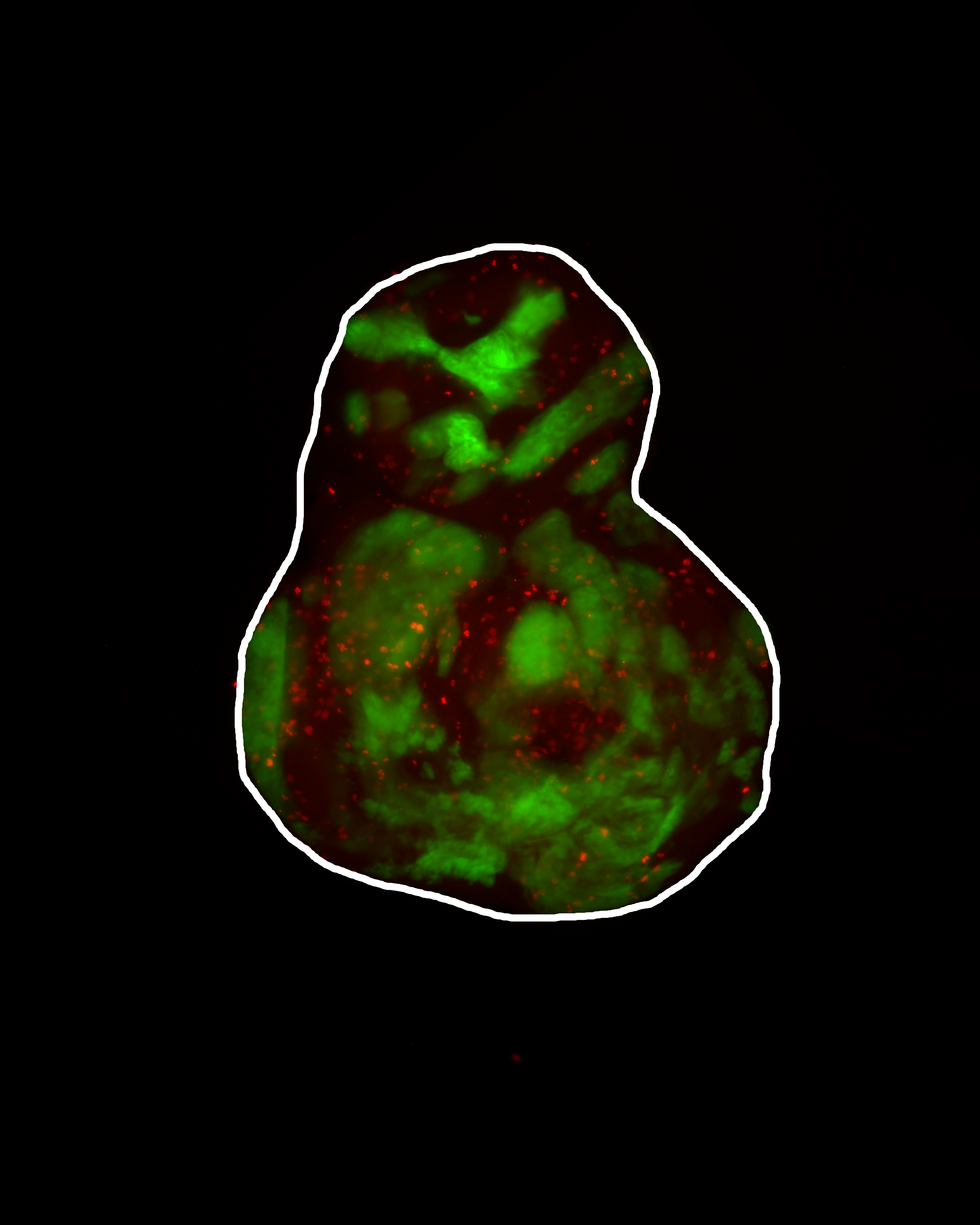

Supplement: Supplementary file 5 — Source data Fig. 1 [file 44318_2025_547_MOESM5_ESM.zip › Figure 1E/3-1 rotated and cut image with border line.tif]

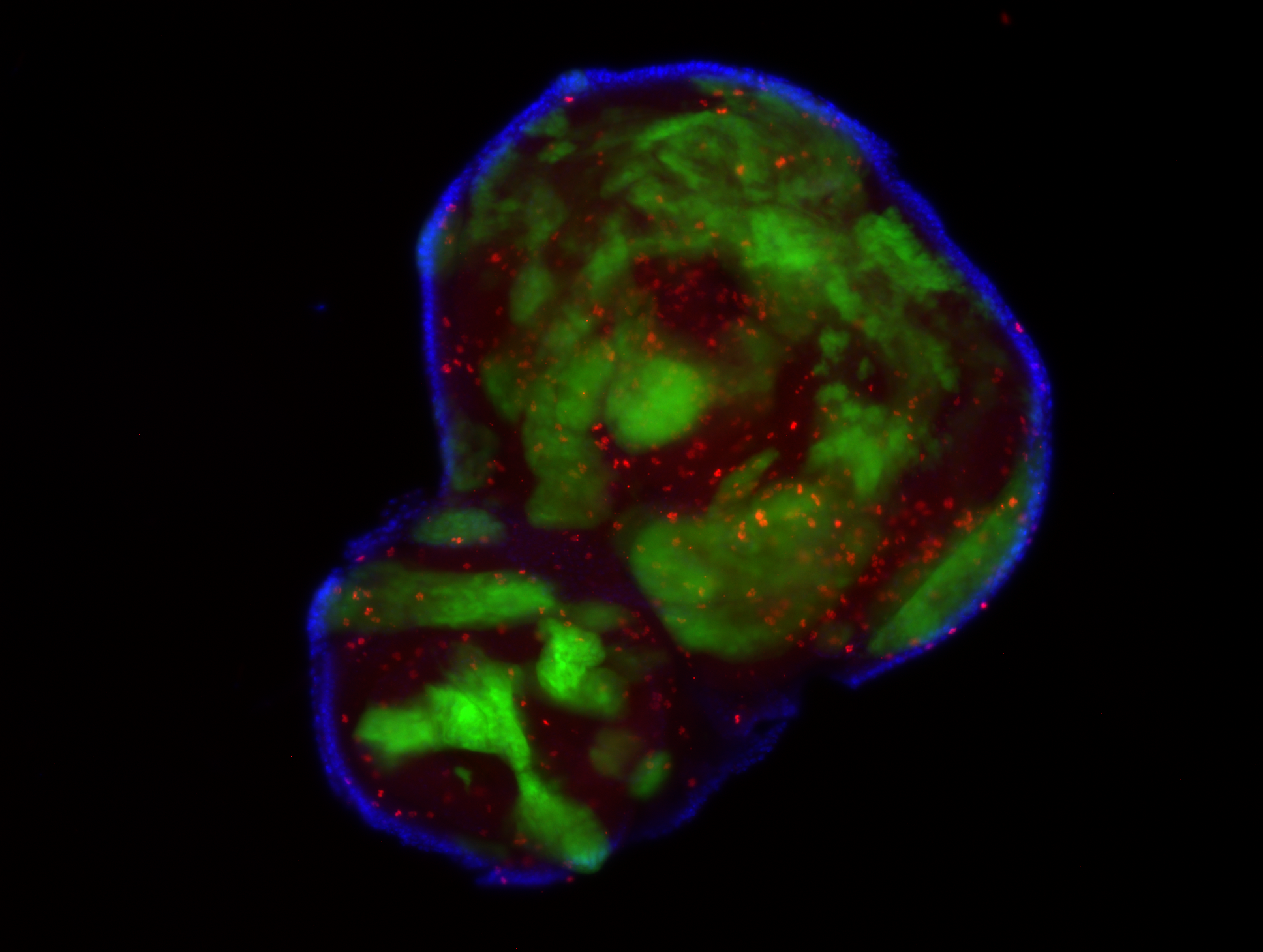

Supplement: Supplementary file 5 — Source data Fig. 1 [file 44318_2025_547_MOESM5_ESM.zip › Figure 1E/3-2 original image.tif]

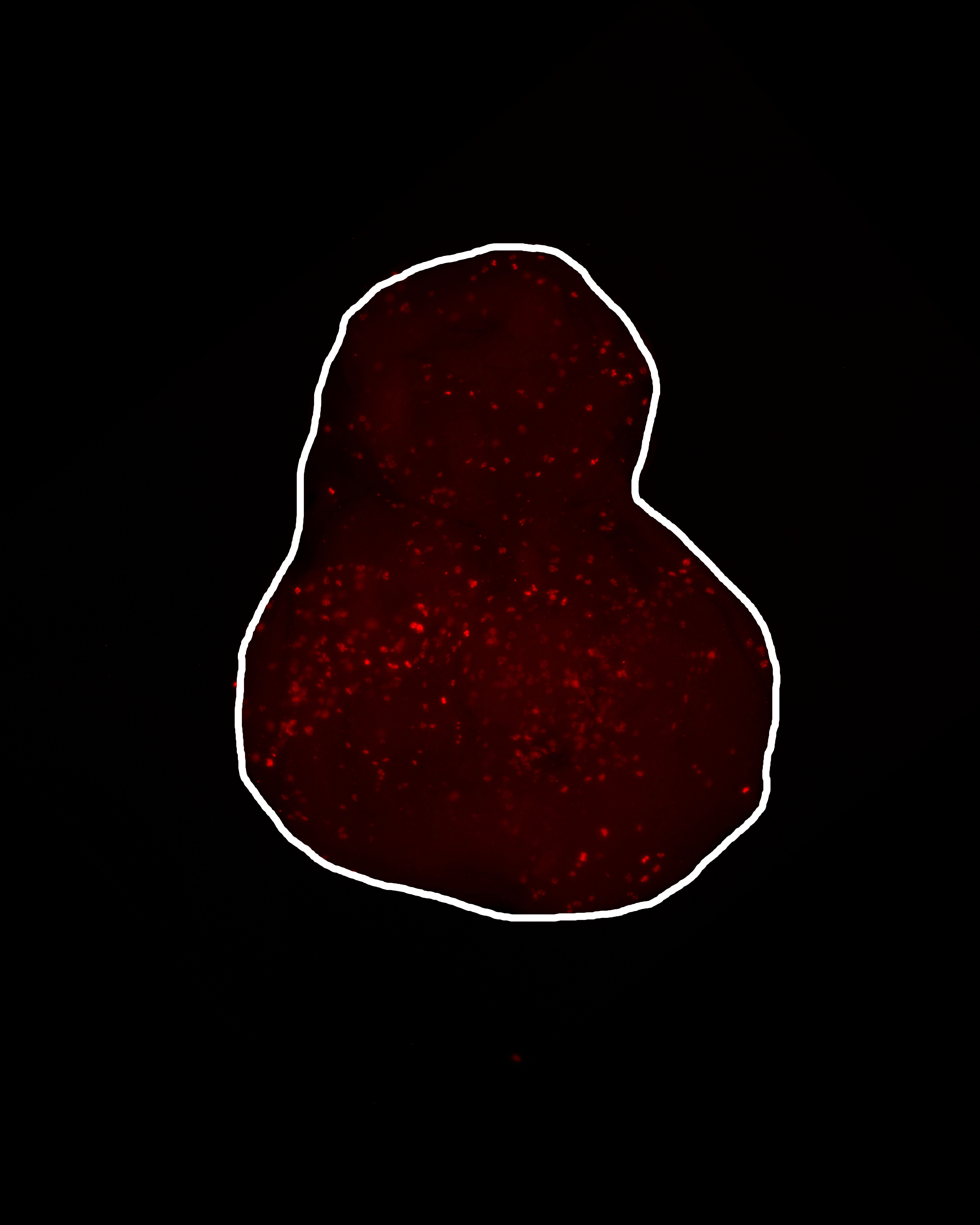

Supplement: Supplementary file 5 — Source data Fig. 1 [file 44318_2025_547_MOESM5_ESM.zip › Figure 1E/4-1 rotated and cut image with border line.tif]

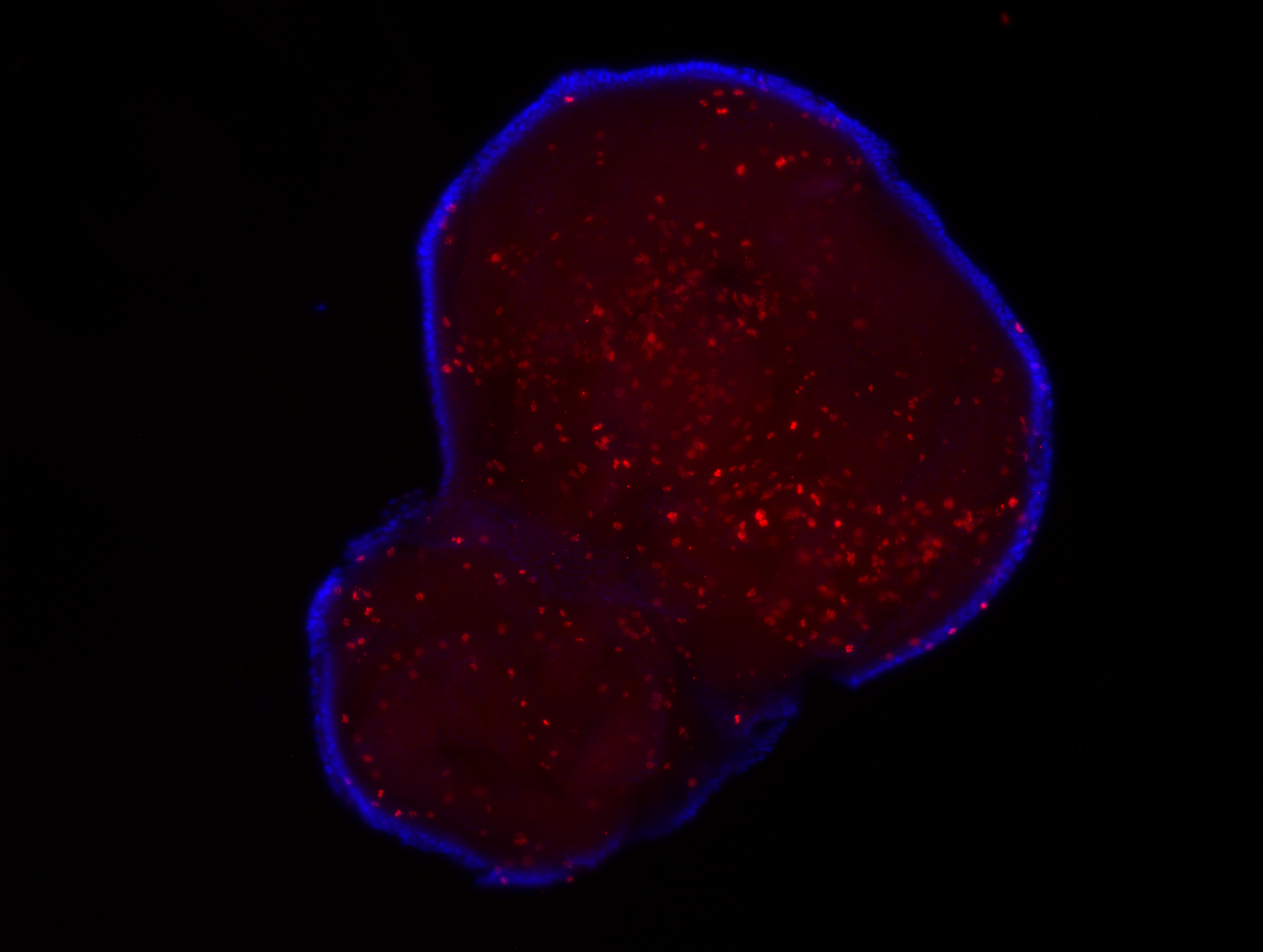

Supplement: Supplementary file 5 — Source data Fig. 1 [file 44318_2025_547_MOESM5_ESM.zip › Figure 1E/4-2 original image.tif]

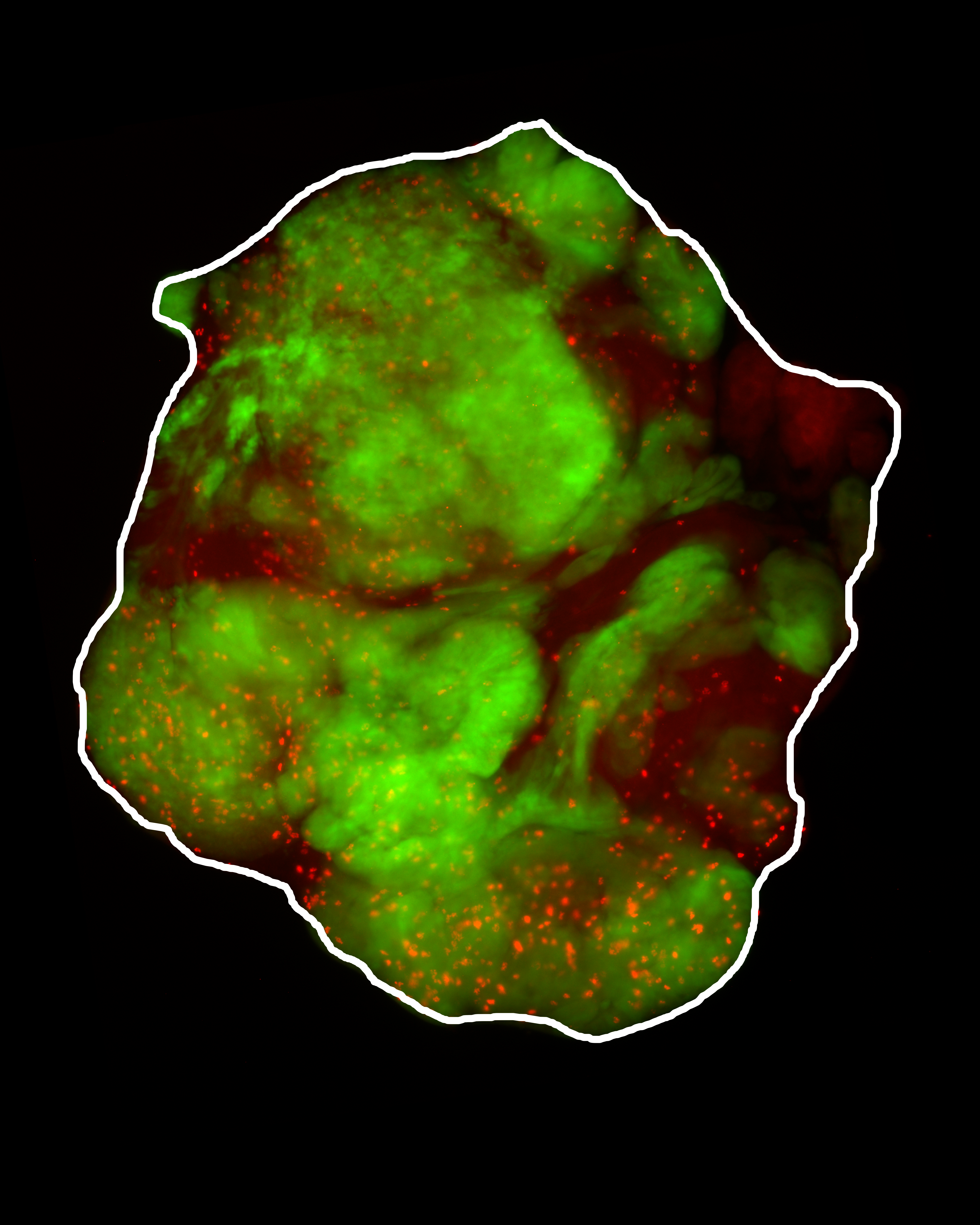

Supplement: Supplementary file 5 — Source data Fig. 1 [file 44318_2025_547_MOESM5_ESM.zip › Figure 1E/5-1 rotated and cut image with border line.tif]

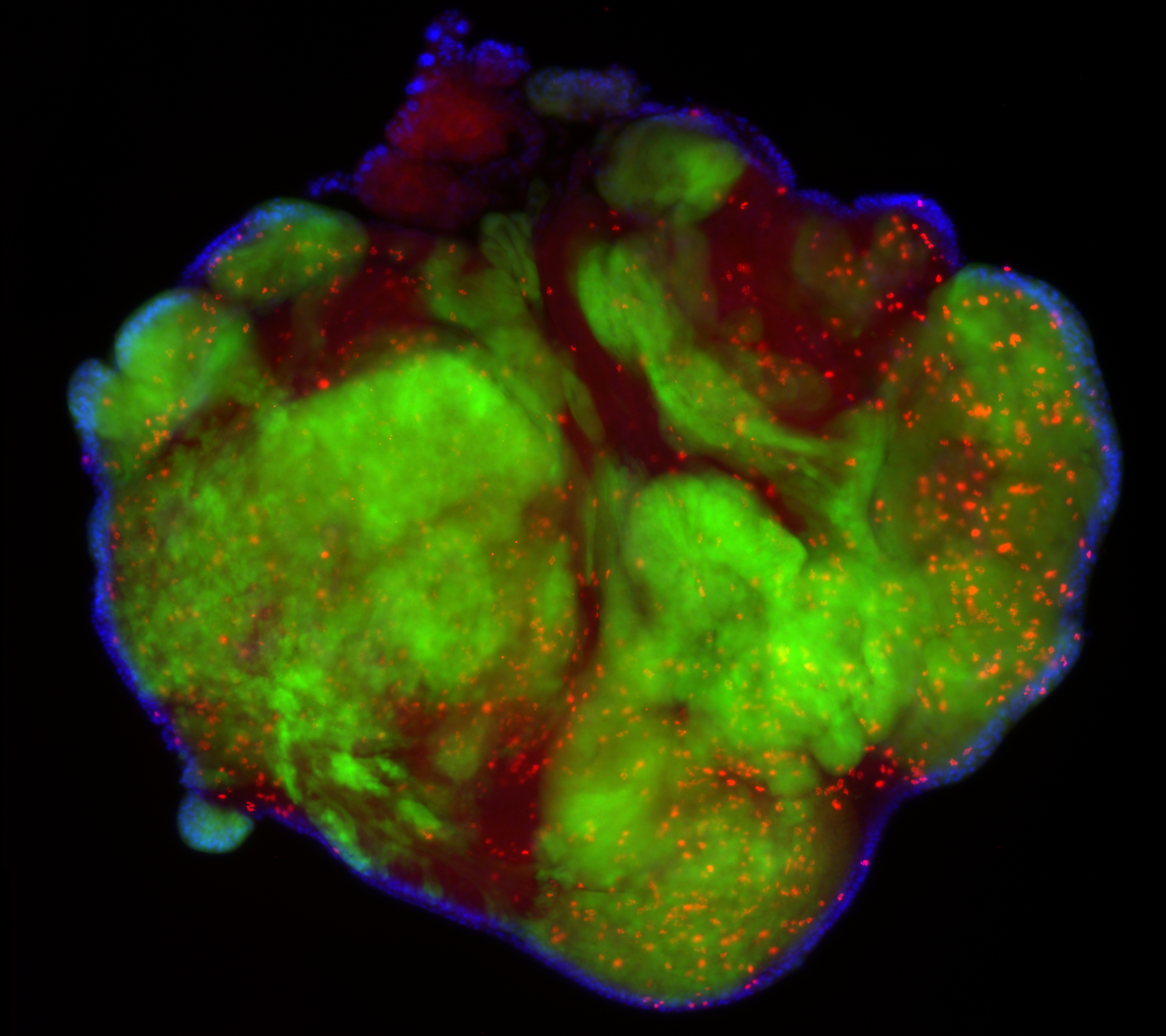

Supplement: Supplementary file 5 — Source data Fig. 1 [file 44318_2025_547_MOESM5_ESM.zip › Figure 1E/5-2 original image.tif]

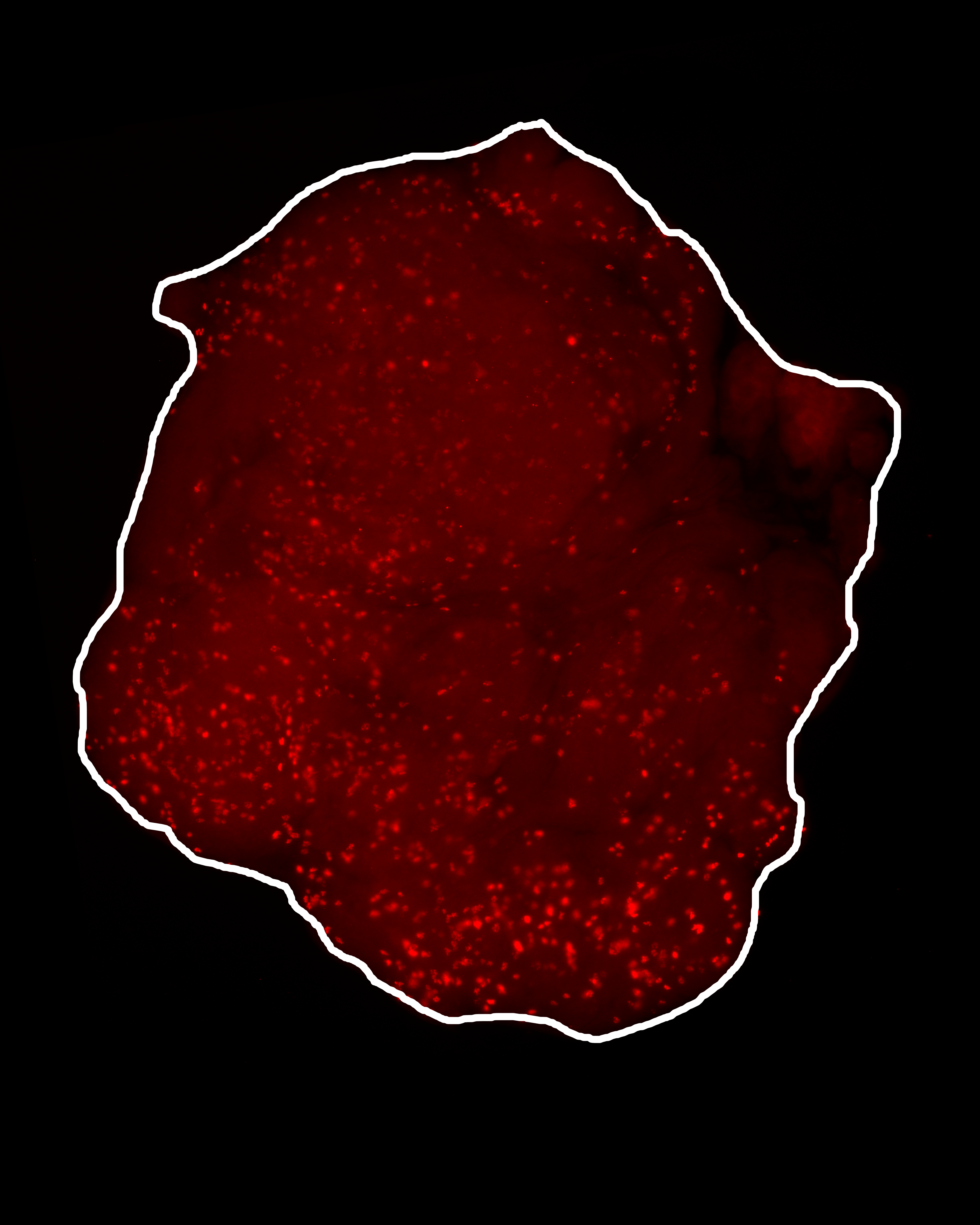

Supplement: Supplementary file 5 — Source data Fig. 1 [file 44318_2025_547_MOESM5_ESM.zip › Figure 1E/6-1 rotated and cut image with border line.tif]

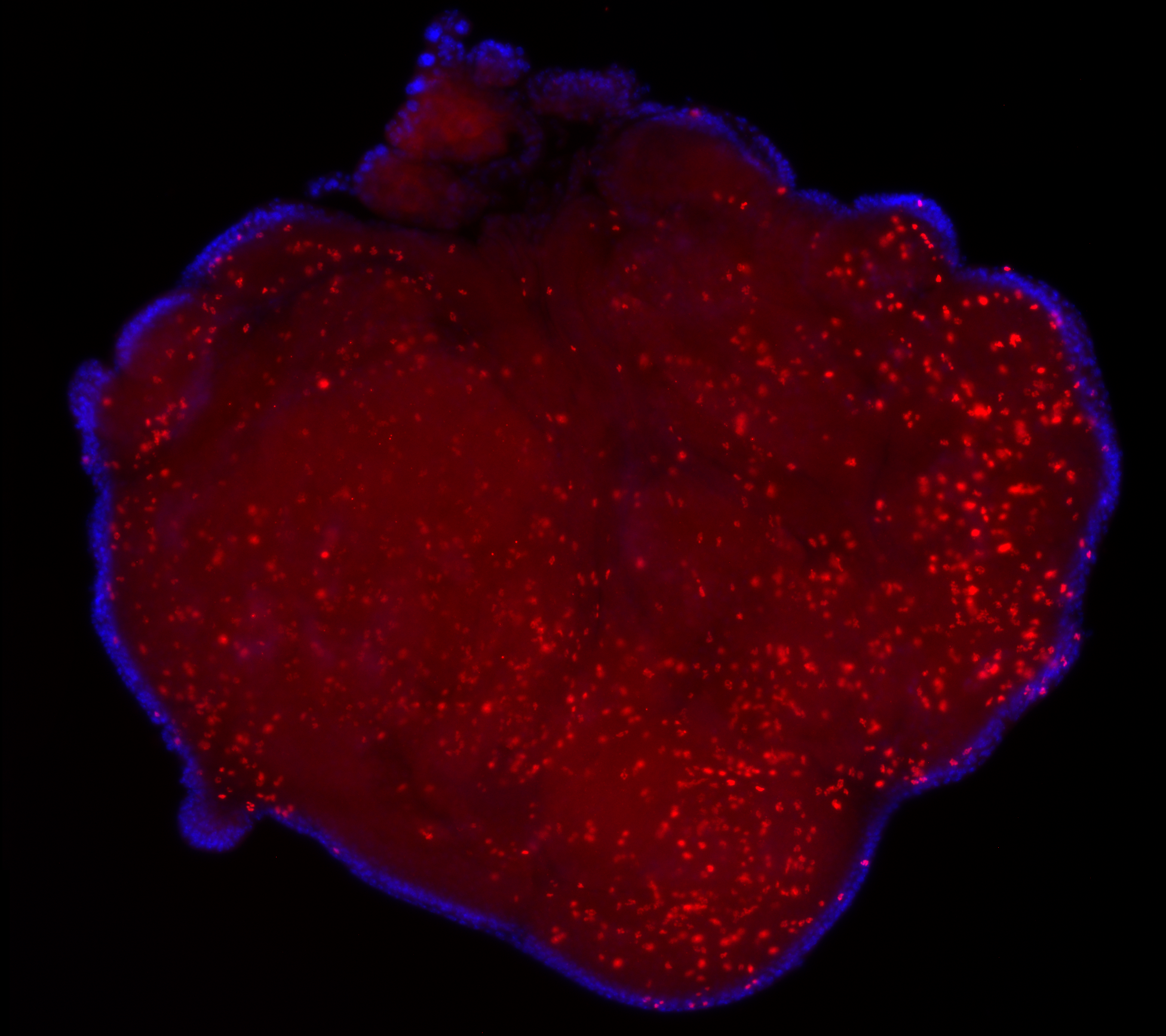

Supplement: Supplementary file 5 — Source data Fig. 1 [file 44318_2025_547_MOESM5_ESM.zip › Figure 1E/6-2 original image.tif]

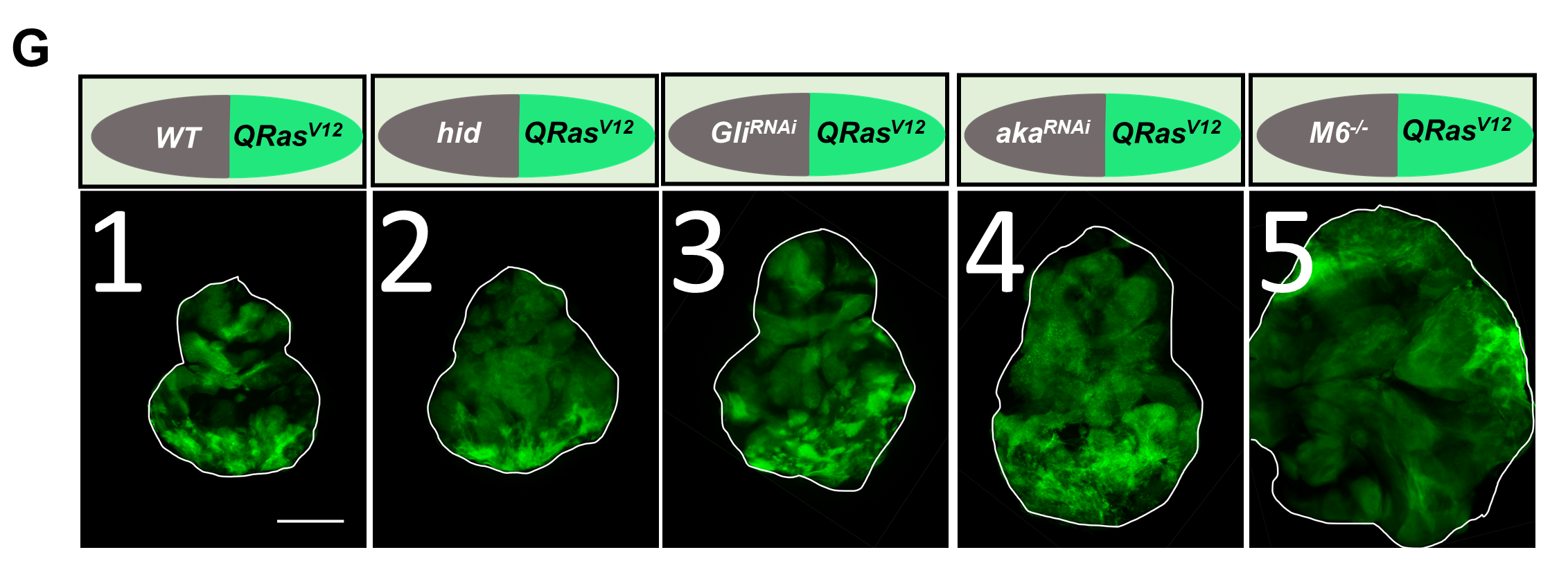

Supplement: Supplementary file 5 — Source data Fig. 1 [file 44318_2025_547_MOESM5_ESM.zip › Figure 1G/0 paper Figure 1G with provided image sequence.tif]

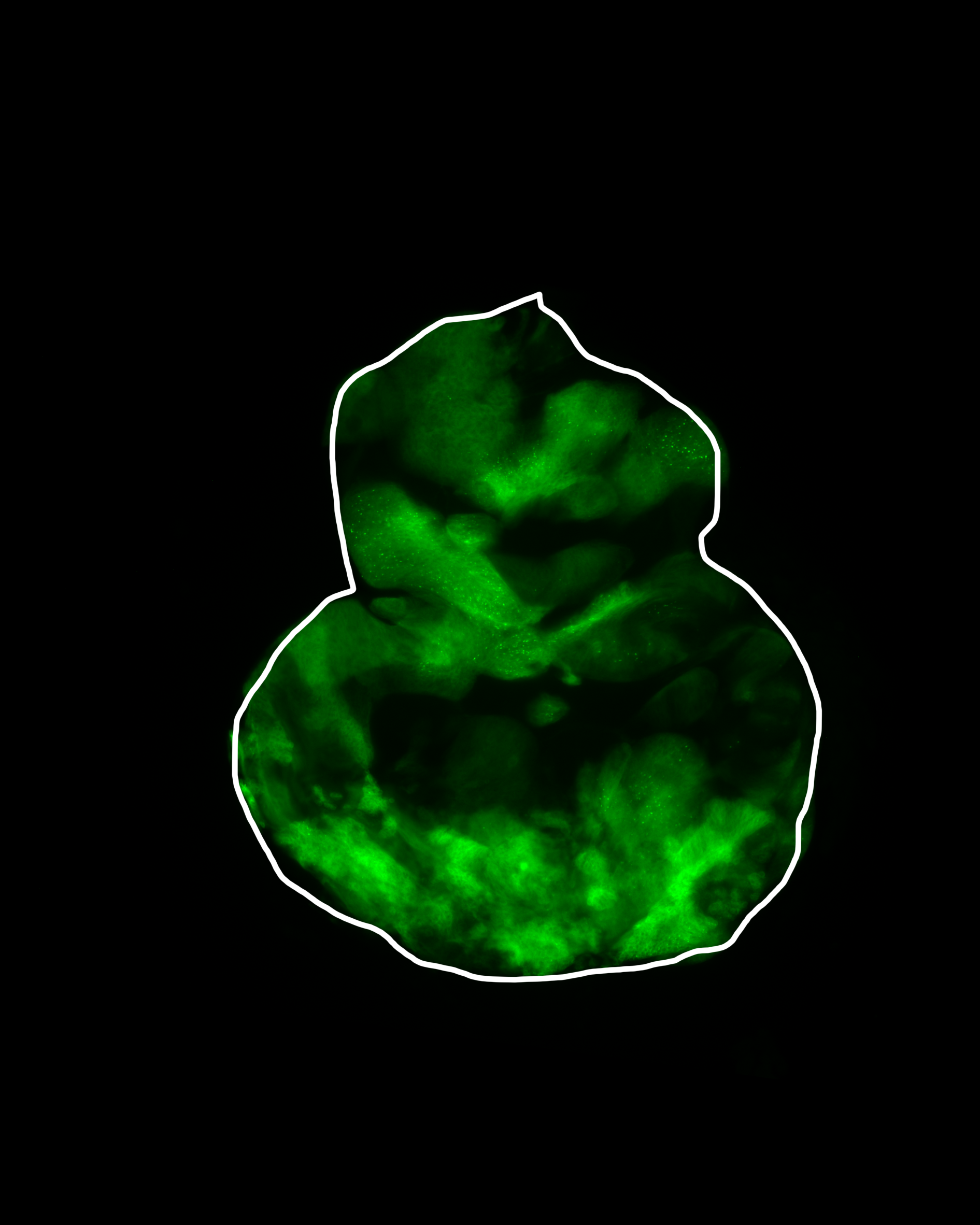

Supplement: Supplementary file 5 — Source data Fig. 1 [file 44318_2025_547_MOESM5_ESM.zip › Figure 1G/1-1 rotated and cut image with border line.tif]

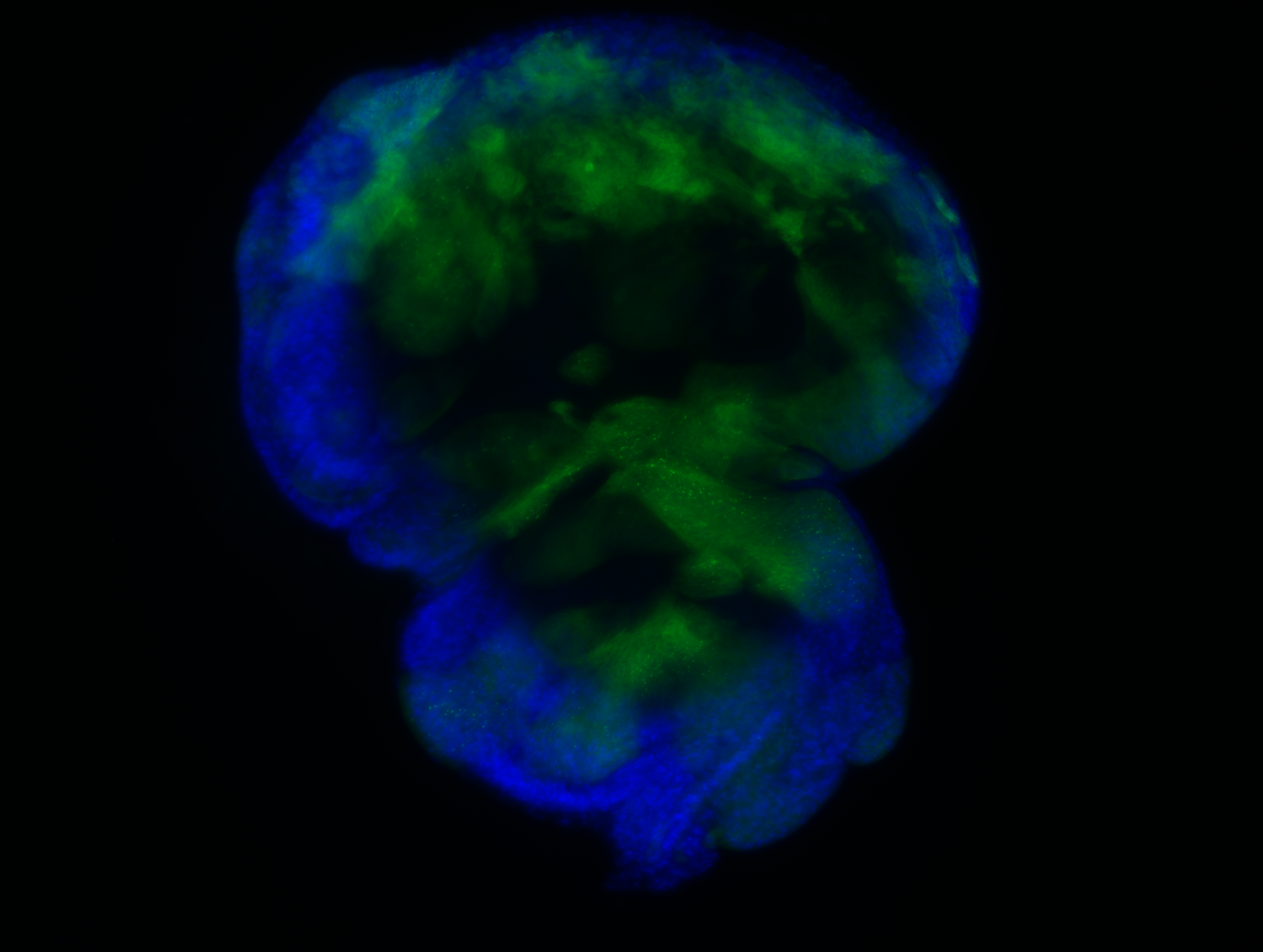

Supplement: Supplementary file 5 — Source data Fig. 1 [file 44318_2025_547_MOESM5_ESM.zip › Figure 1G/1-2 original image.tif]

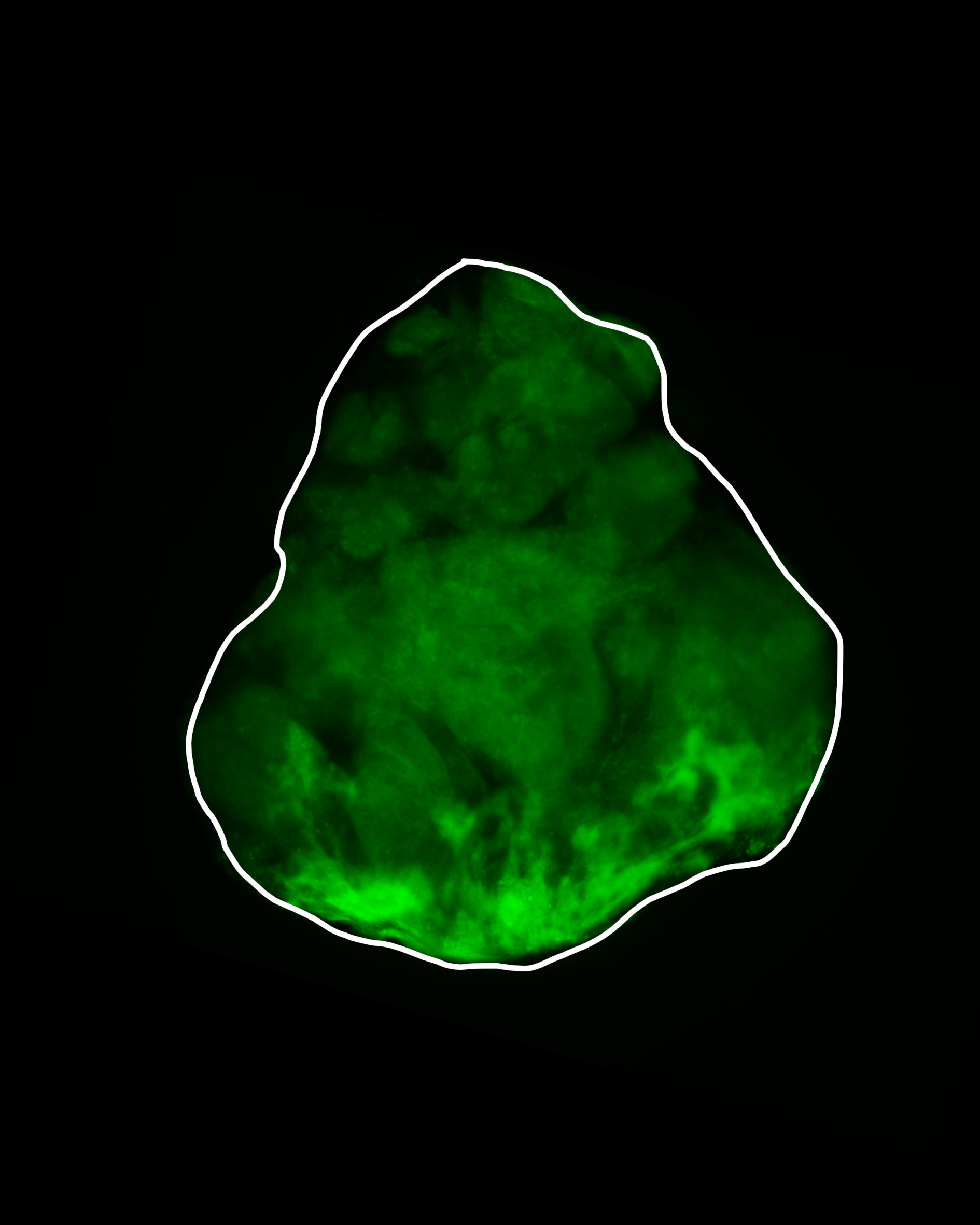

Supplement: Supplementary file 5 — Source data Fig. 1 [file 44318_2025_547_MOESM5_ESM.zip › Figure 1G/2-1 rotated and cut image with border line.tif]

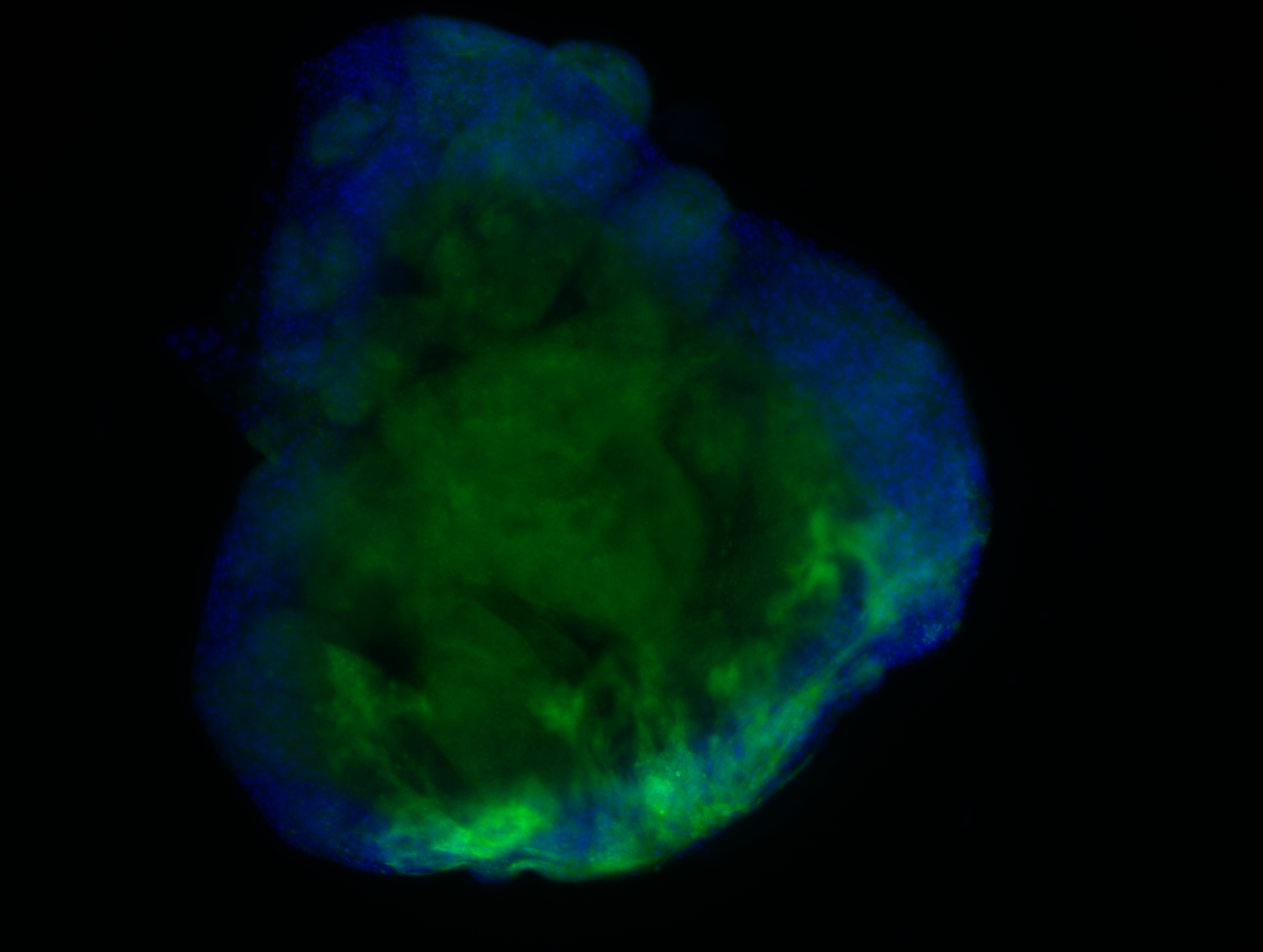

Supplement: Supplementary file 5 — Source data Fig. 1 [file 44318_2025_547_MOESM5_ESM.zip › Figure 1G/2-2 original image.tif]

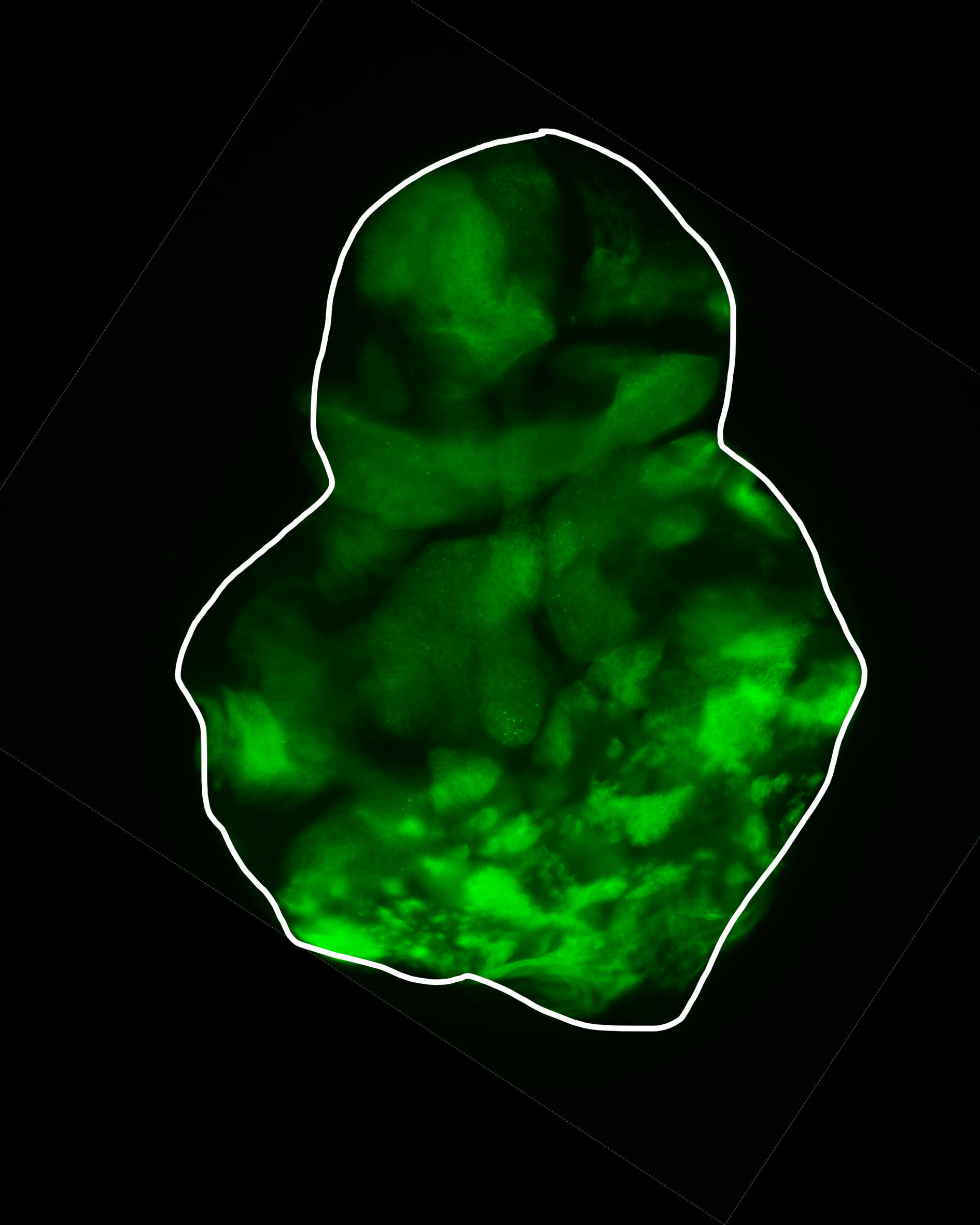

Supplement: Supplementary file 5 — Source data Fig. 1 [file 44318_2025_547_MOESM5_ESM.zip › Figure 1G/3-1 rotated and cut image with border line.tif]

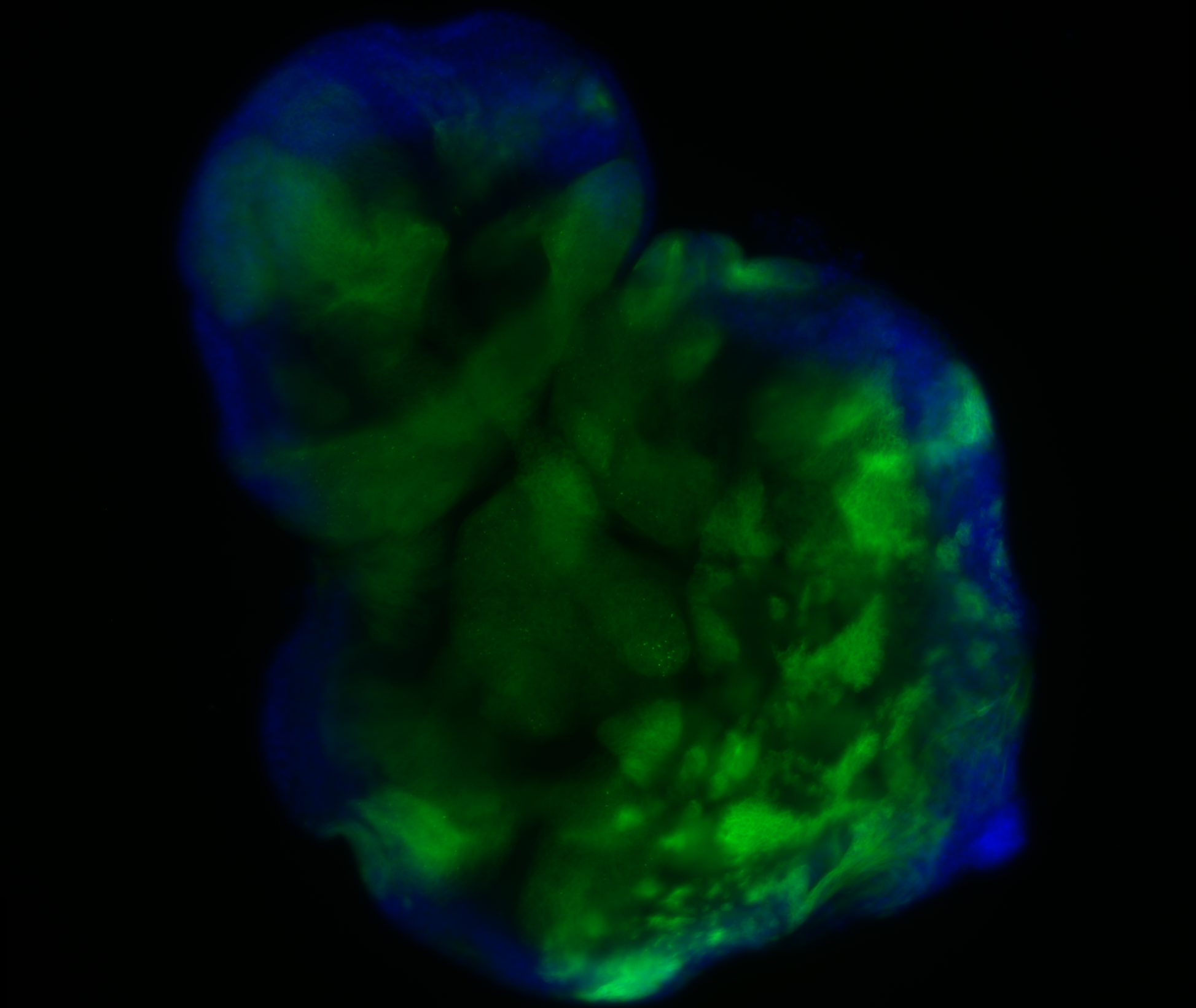

Supplement: Supplementary file 5 — Source data Fig. 1 [file 44318_2025_547_MOESM5_ESM.zip › Figure 1G/3-2 original image.tif]

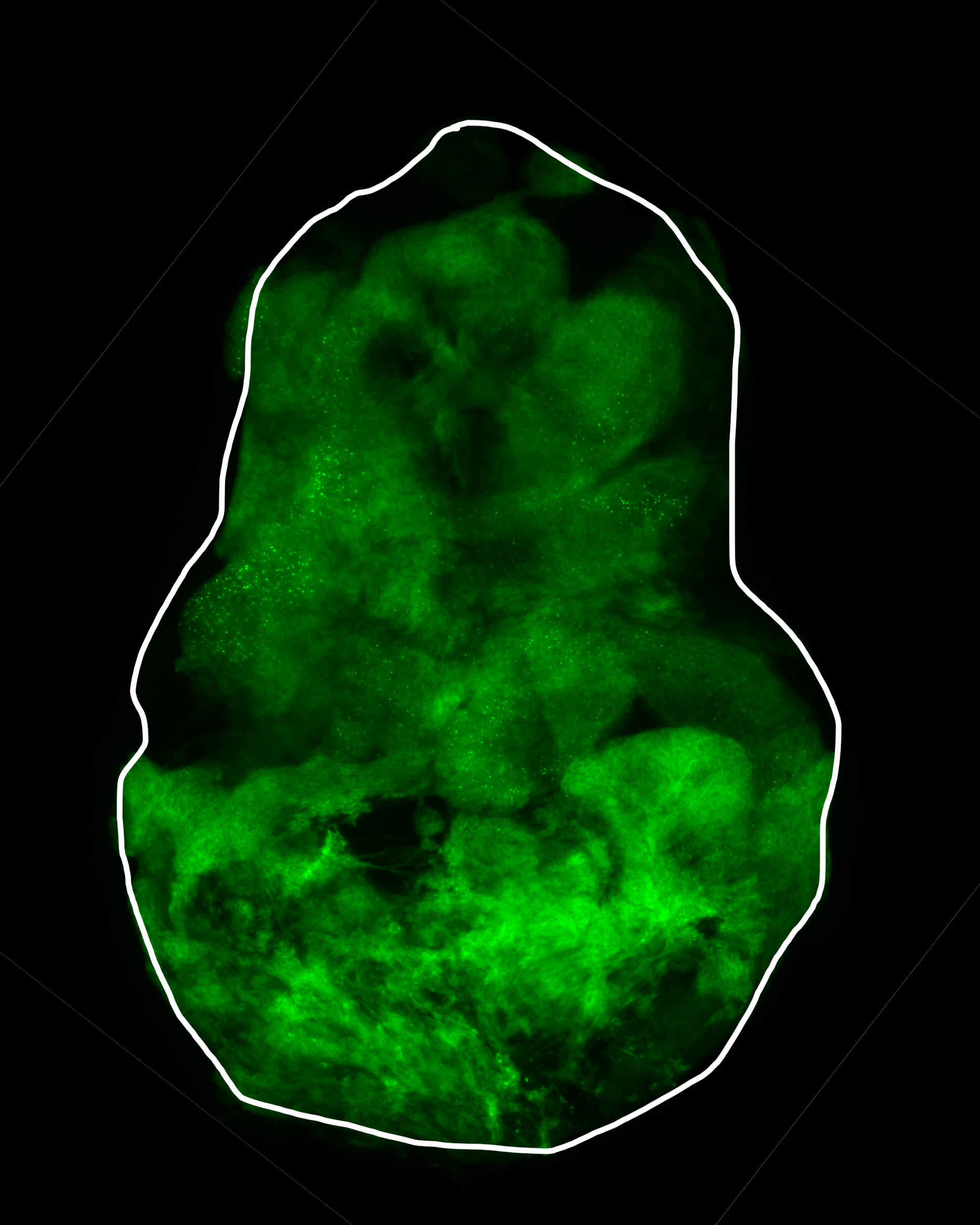

Supplement: Supplementary file 5 — Source data Fig. 1 [file 44318_2025_547_MOESM5_ESM.zip › Figure 1G/4-1 rotated and cut image with border line.tif]

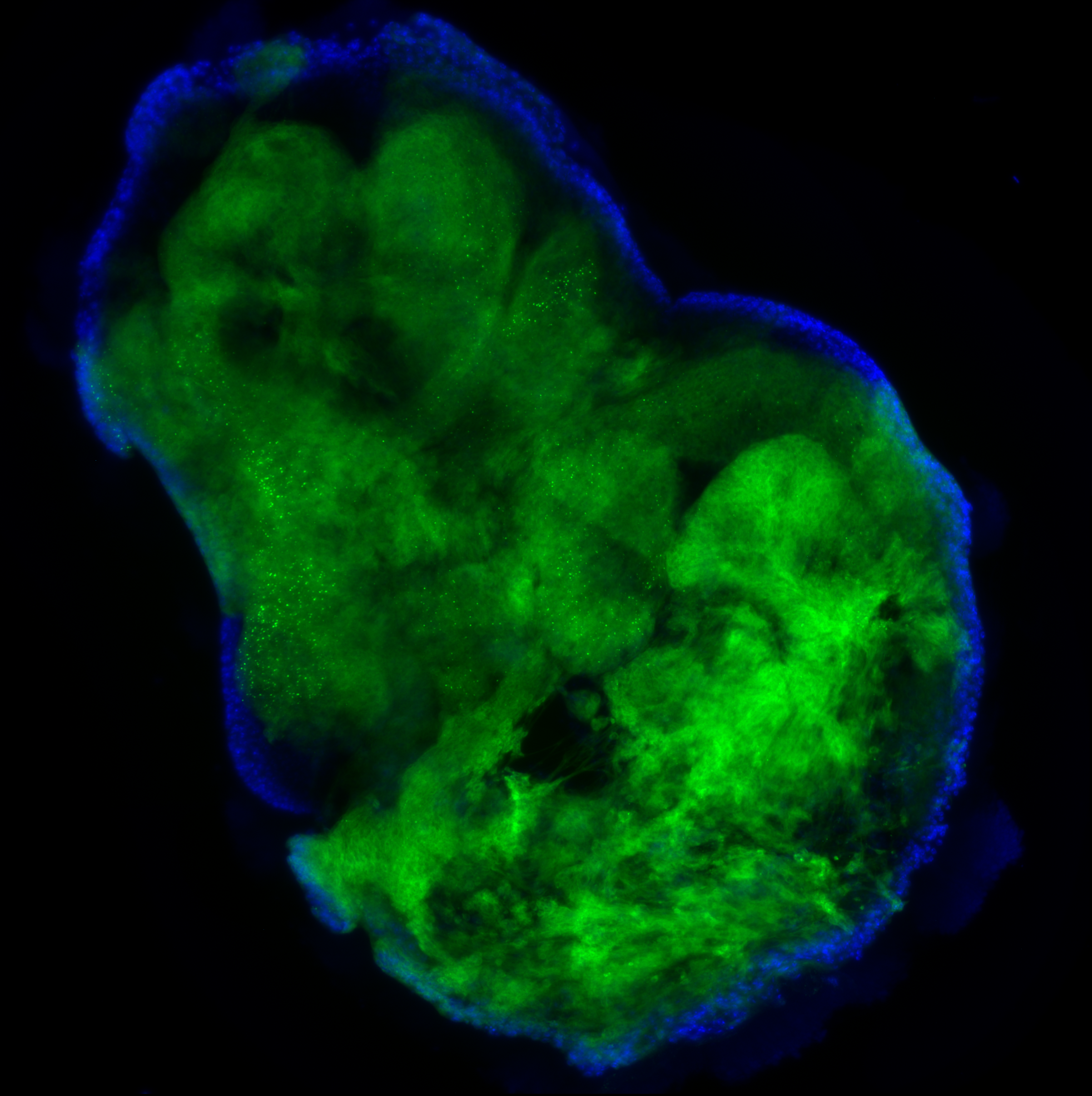

Supplement: Supplementary file 5 — Source data Fig. 1 [file 44318_2025_547_MOESM5_ESM.zip › Figure 1G/4-2 original image.tif]

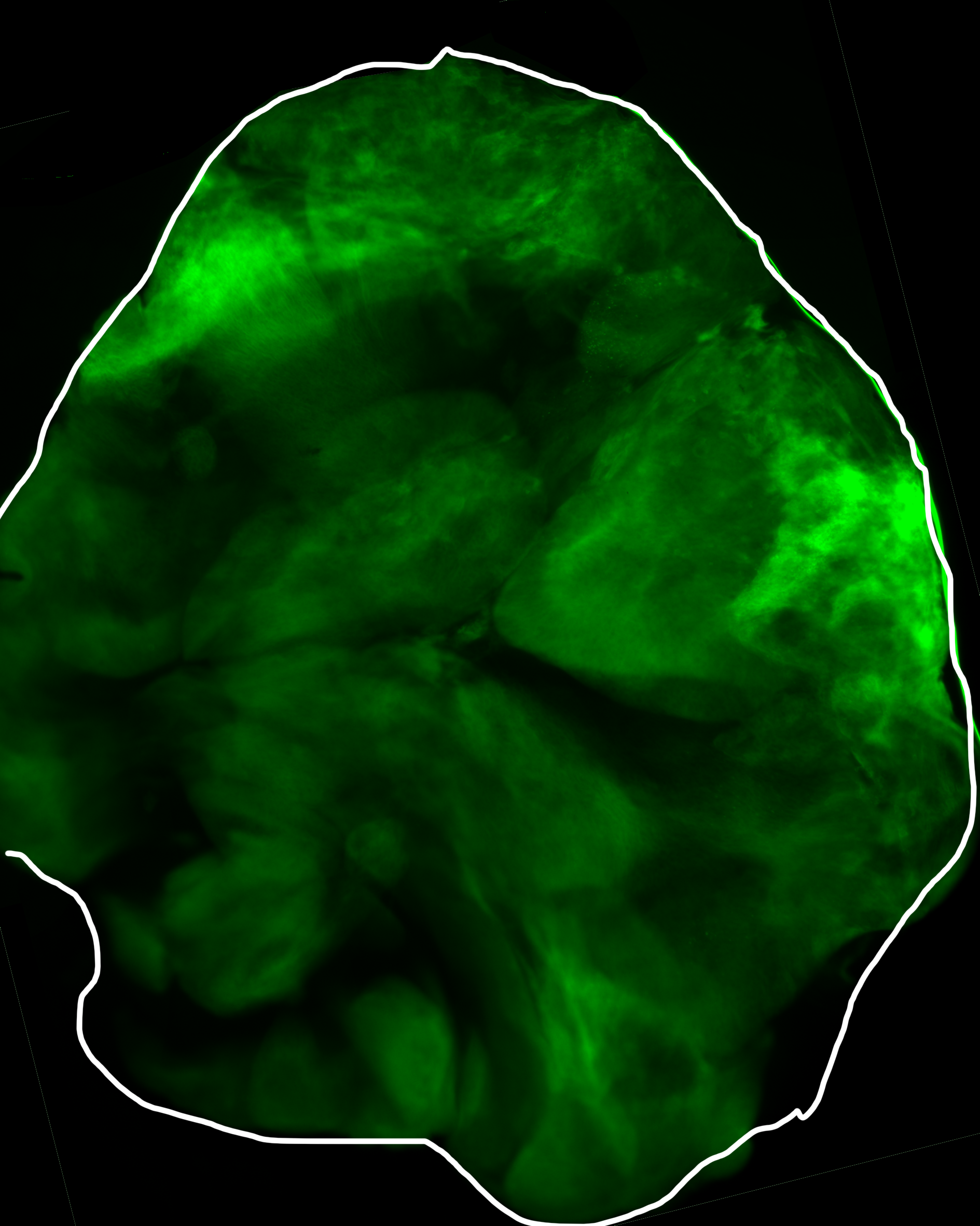

Supplement: Supplementary file 5 — Source data Fig. 1 [file 44318_2025_547_MOESM5_ESM.zip › Figure 1G/5-1 rotated and cut image with border line.tif]

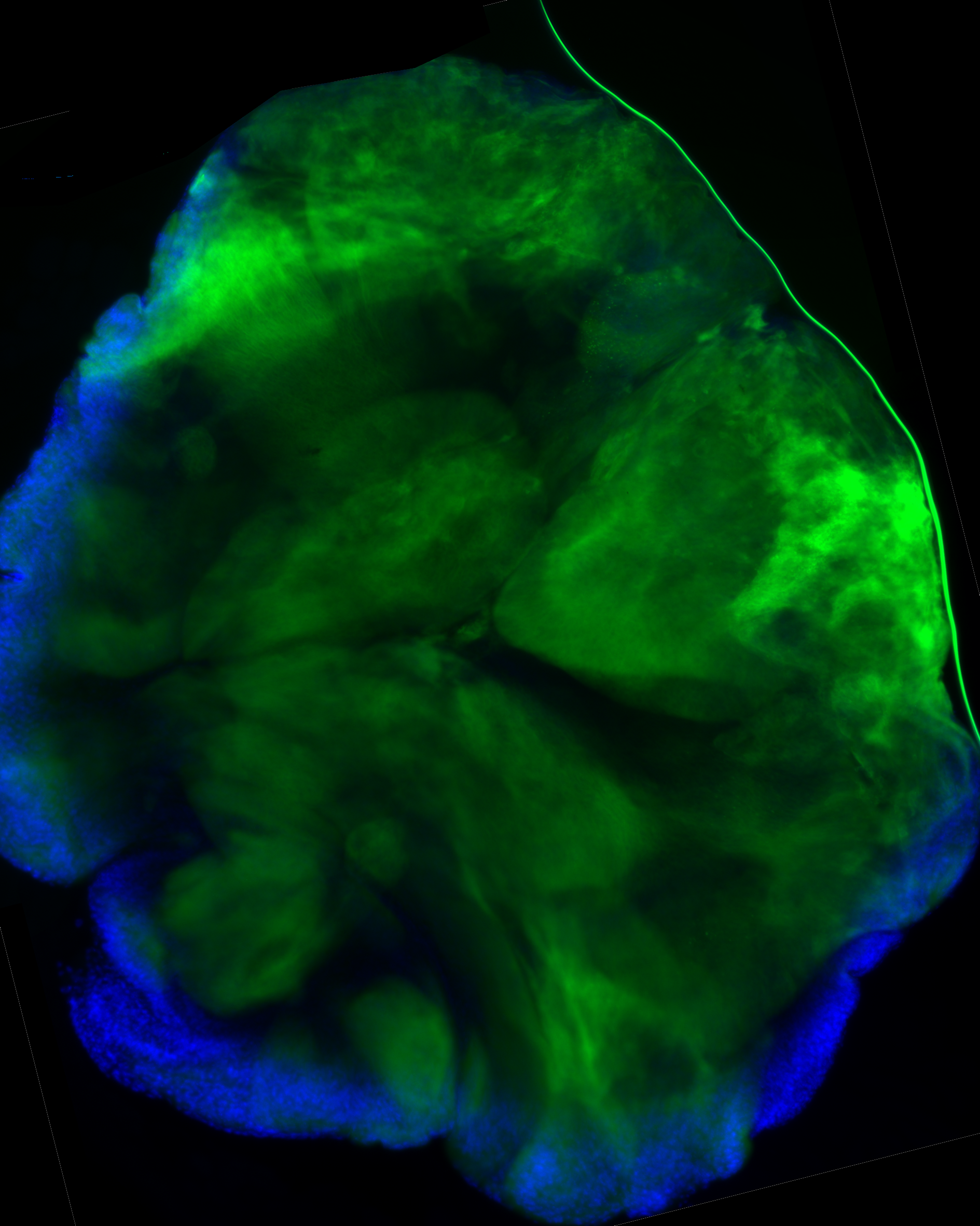

Supplement: Supplementary file 5 — Source data Fig. 1 [file 44318_2025_547_MOESM5_ESM.zip › Figure 1G/5-2 original image.tif]

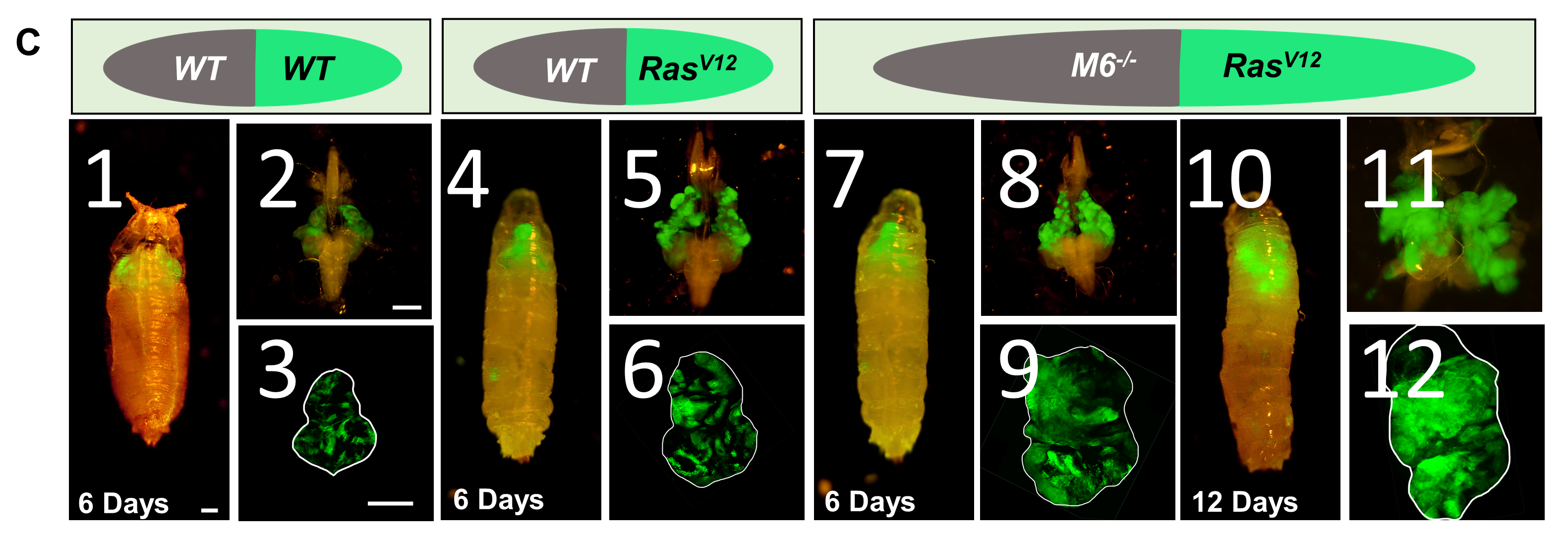

Supplement: Supplementary file 5 — Source data Fig. 1 [file 44318_2025_547_MOESM5_ESM.zip › Figure 1C/0 paper Figure 1C with provided image sequence.tif]

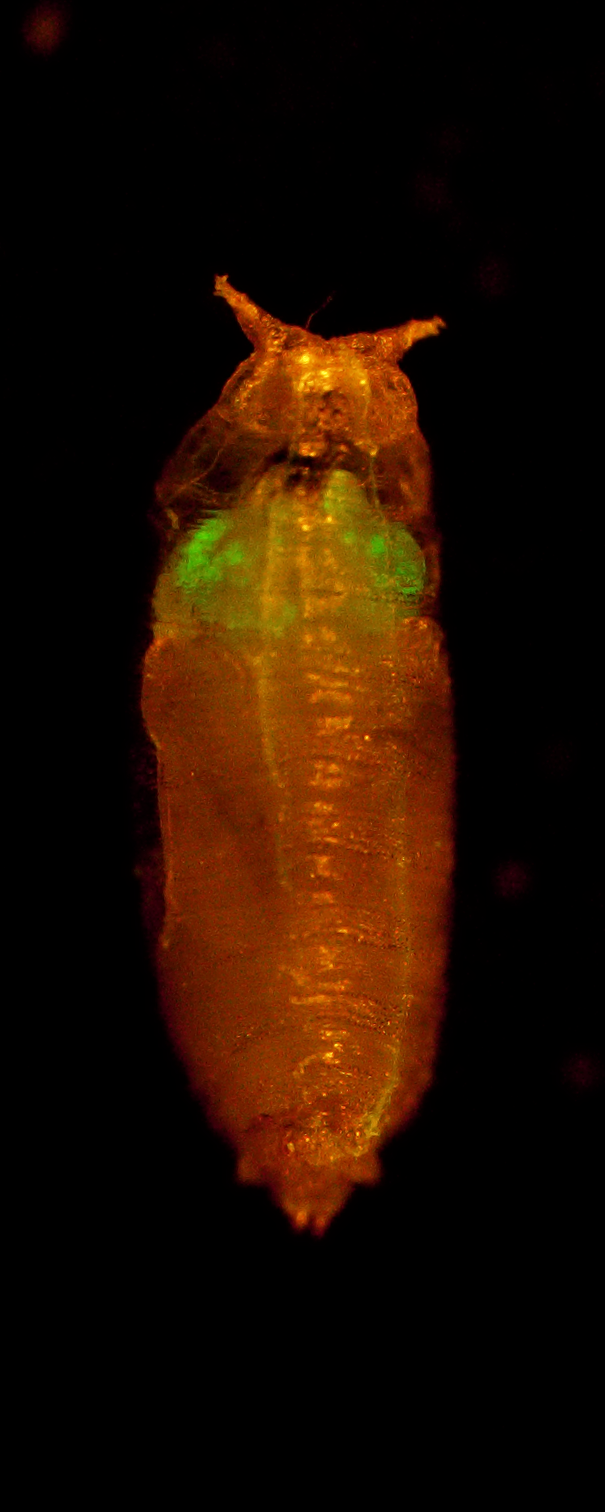

Supplement: Supplementary file 5 — Source data Fig. 1 [file 44318_2025_547_MOESM5_ESM.zip › Figure 1C/1-1 rotated and cut image.tif]

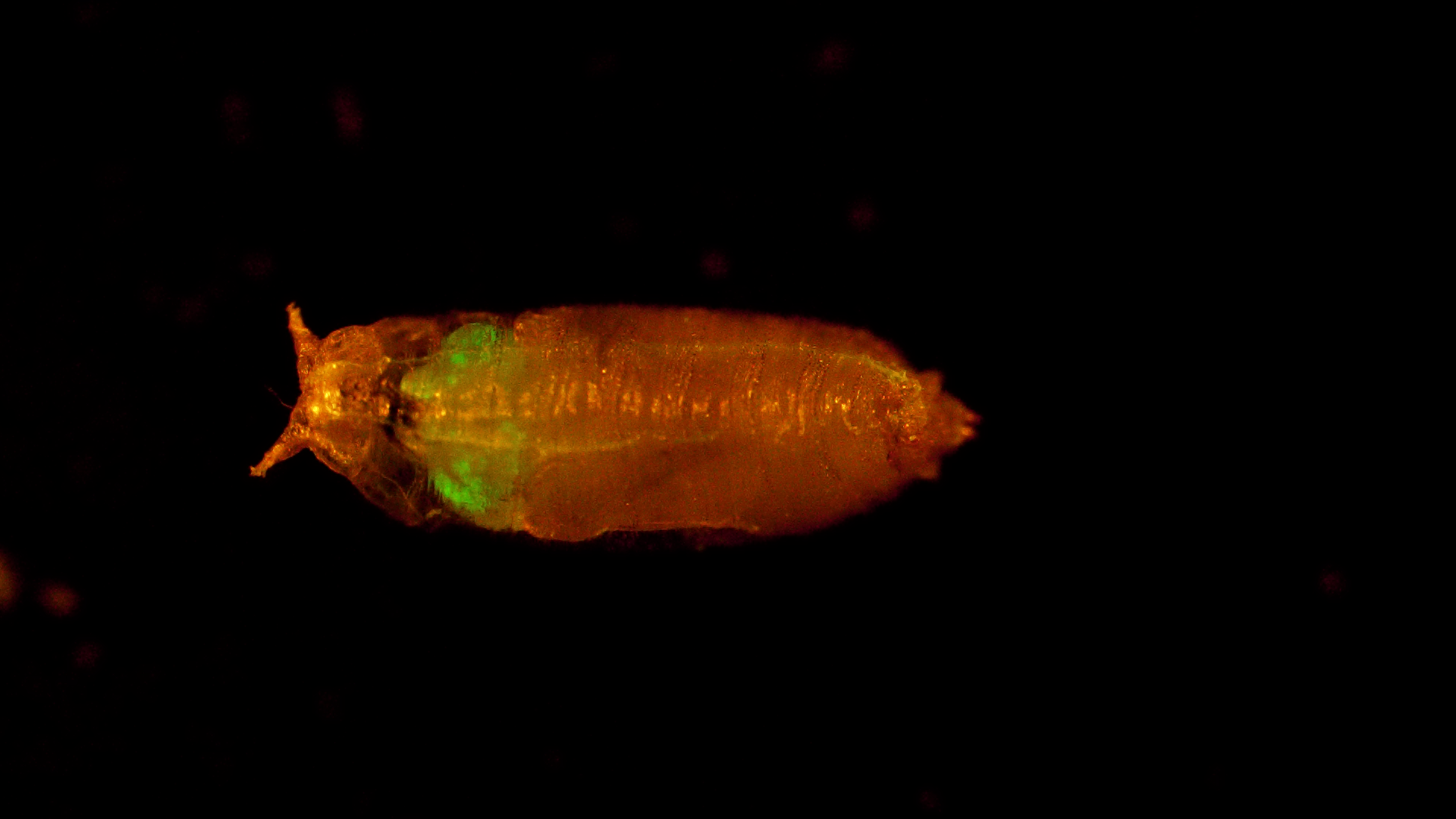

Supplement: Supplementary file 5 — Source data Fig. 1 [file 44318_2025_547_MOESM5_ESM.zip › Figure 1C/1-2 original image.tif]

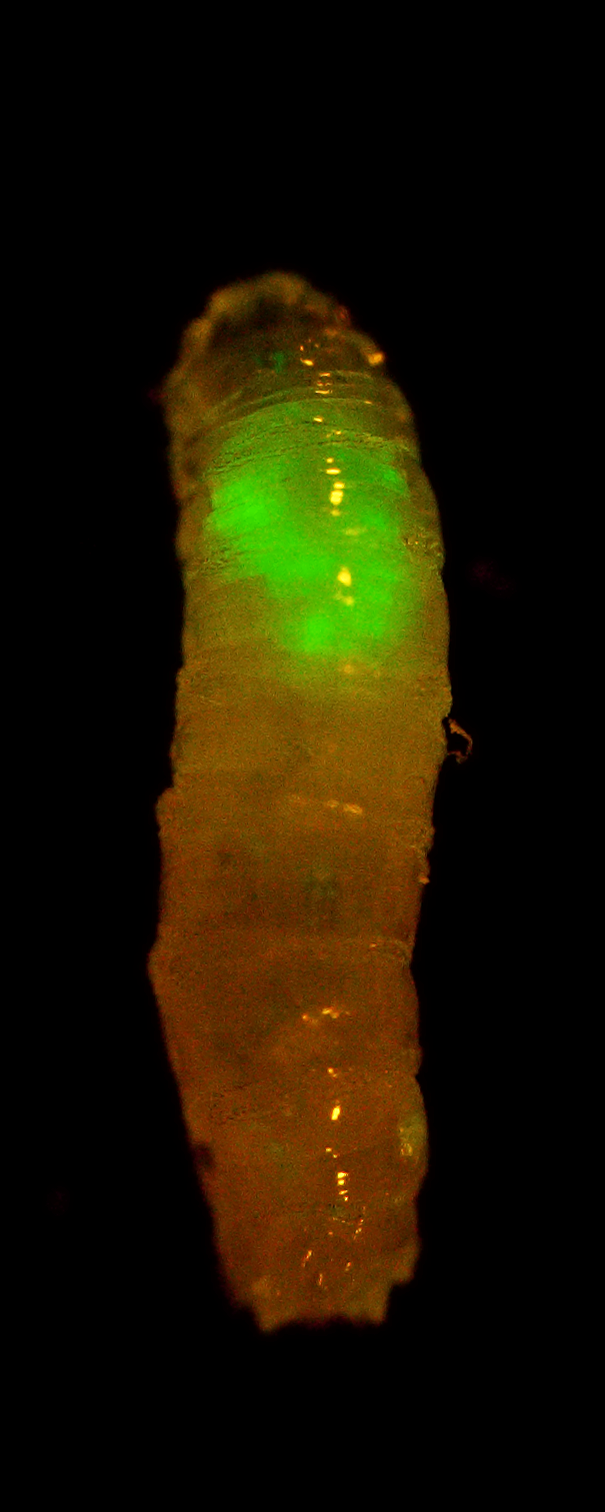

Supplement: Supplementary file 5 — Source data Fig. 1 [file 44318_2025_547_MOESM5_ESM.zip › Figure 1C/10-1 rotated and cut image.tif]

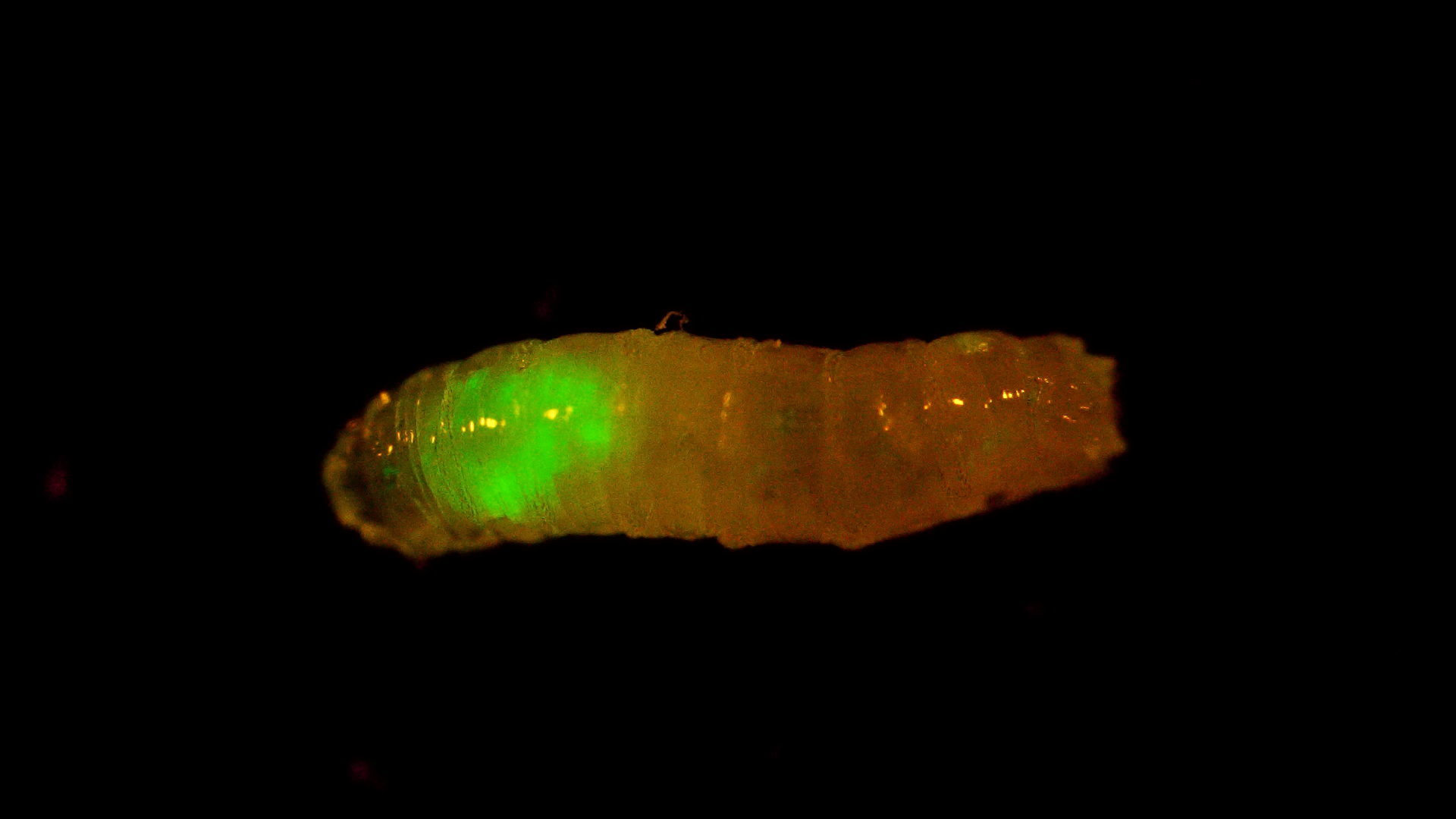

Supplement: Supplementary file 5 — Source data Fig. 1 [file 44318_2025_547_MOESM5_ESM.zip › Figure 1C/10-2 original image.tif]

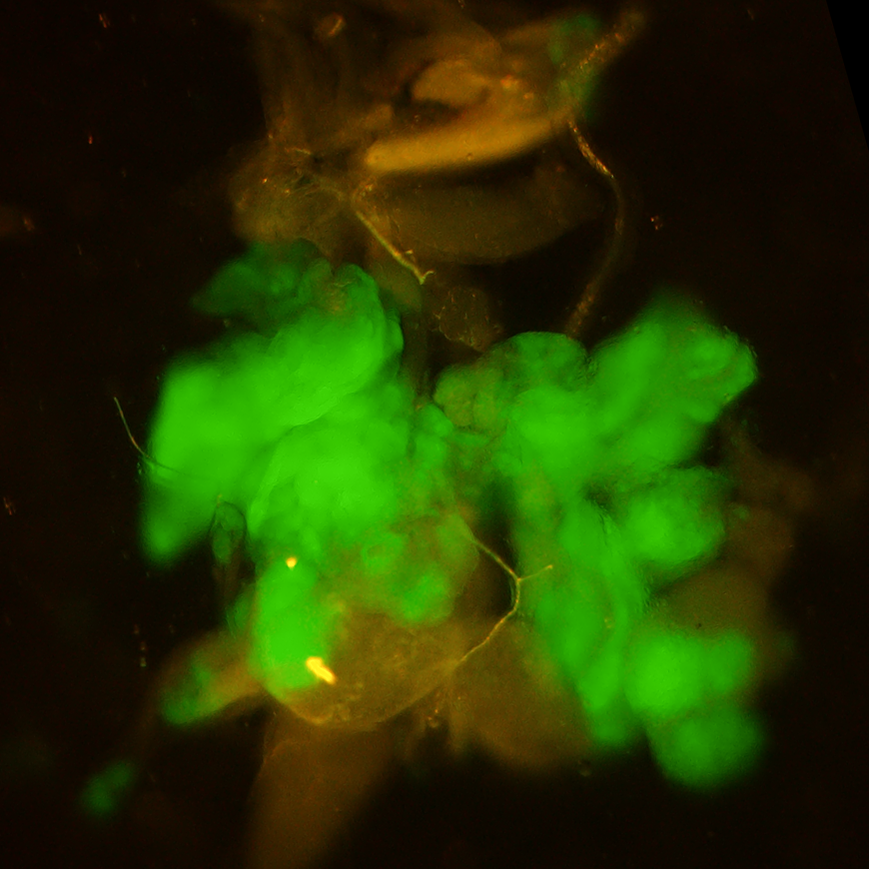

Supplement: Supplementary file 5 — Source data Fig. 1 [file 44318_2025_547_MOESM5_ESM.zip › Figure 1C/11-1 rotated and cut image.tif]

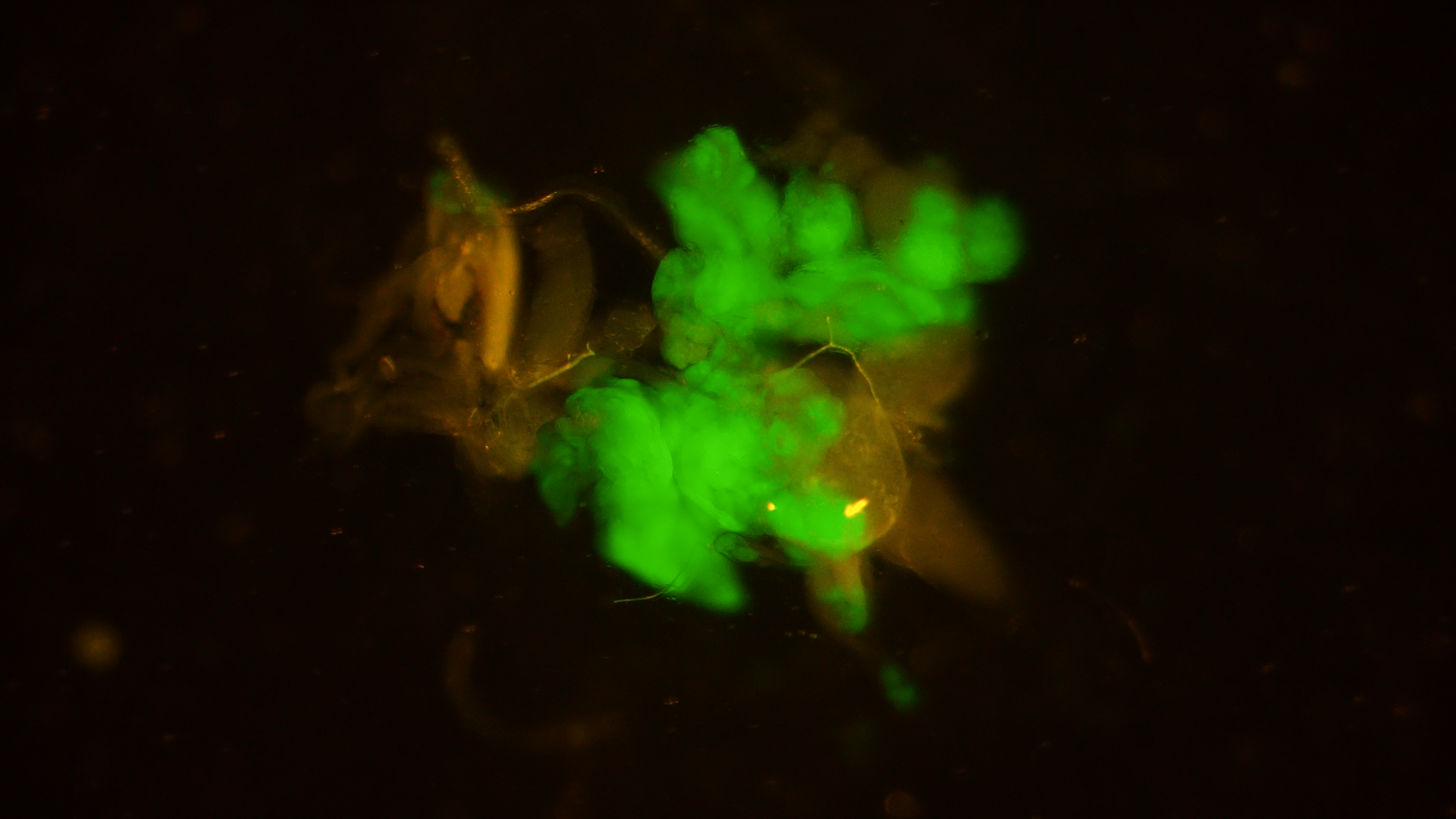

Supplement: Supplementary file 5 — Source data Fig. 1 [file 44318_2025_547_MOESM5_ESM.zip › Figure 1C/11-2 original image.tif]

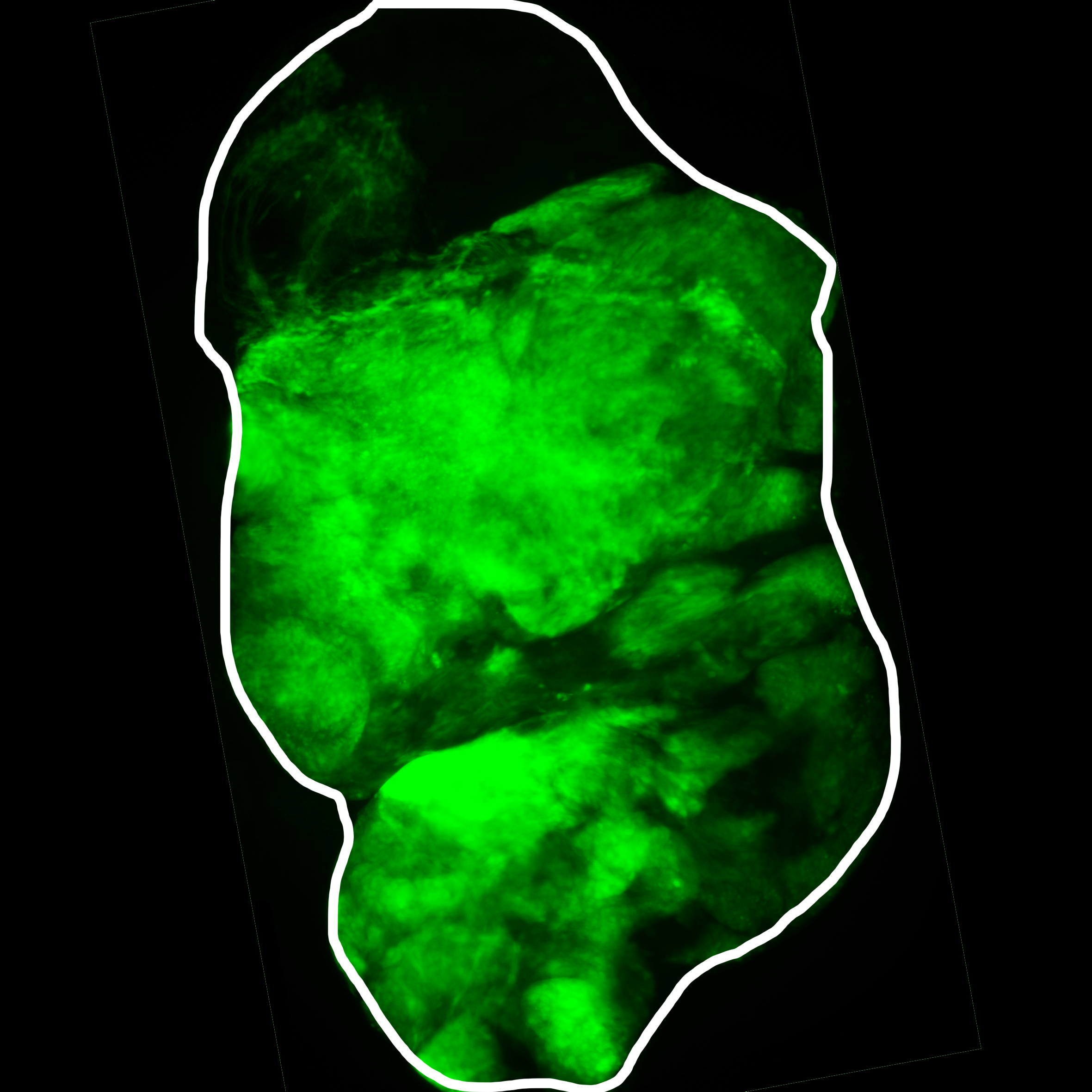

Supplement: Supplementary file 5 — Source data Fig. 1 [file 44318_2025_547_MOESM5_ESM.zip › Figure 1C/12-1 rotated and cut image with border line.tif]

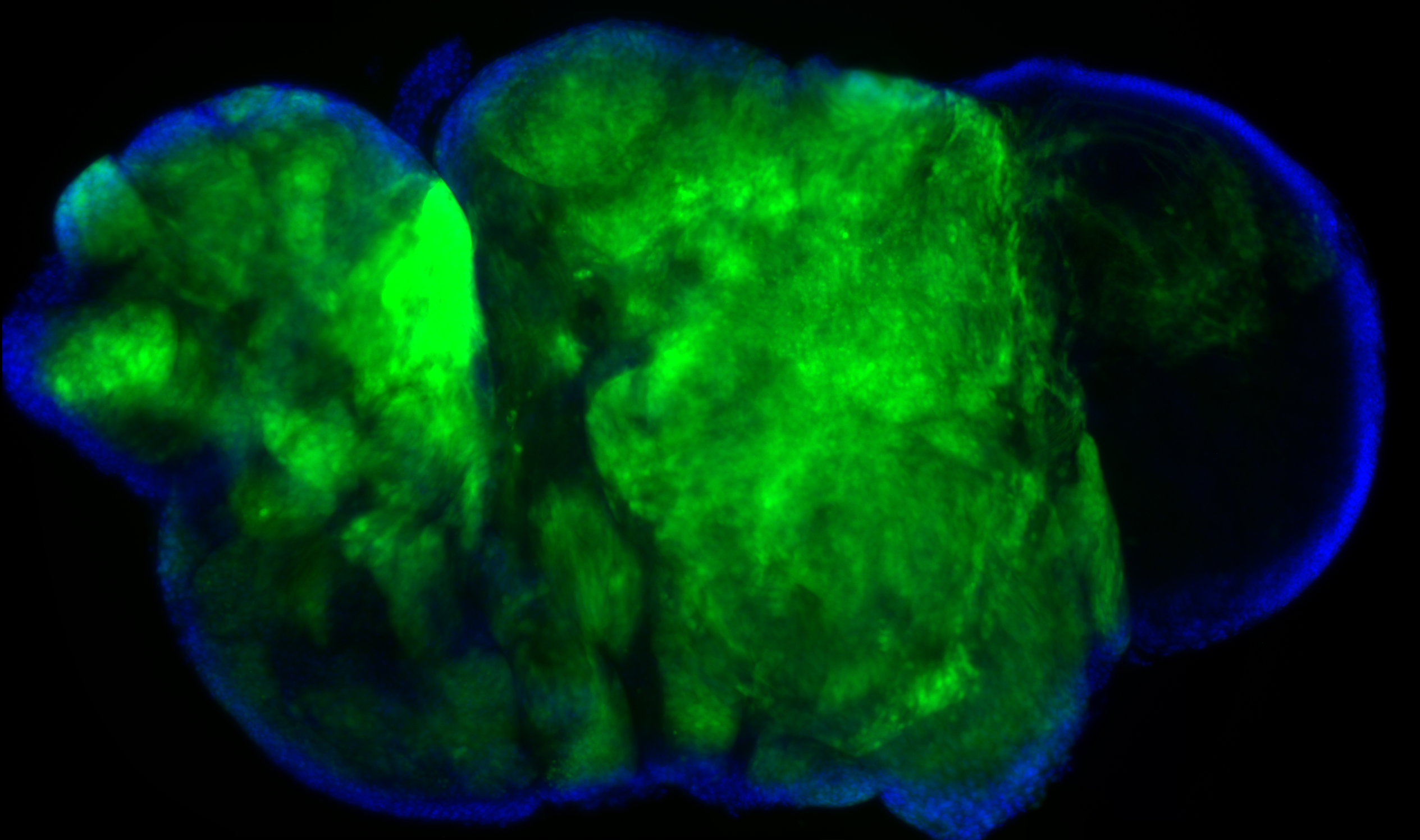

Supplement: Supplementary file 5 — Source data Fig. 1 [file 44318_2025_547_MOESM5_ESM.zip › Figure 1C/12-2 original image.tif]

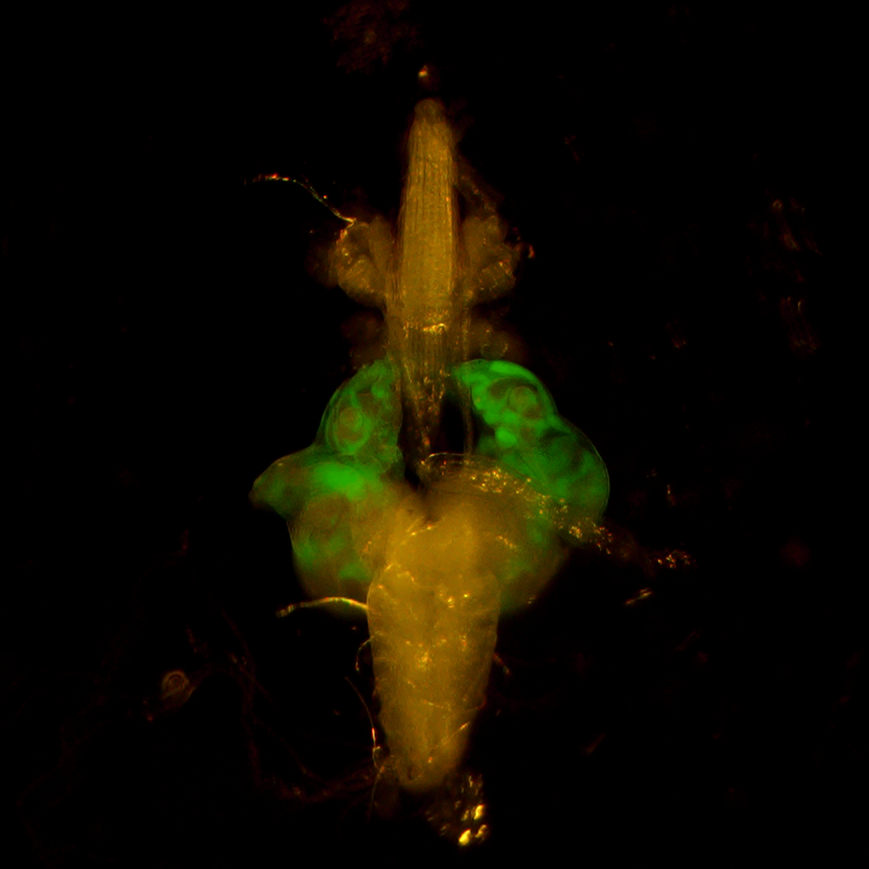

Supplement: Supplementary file 5 — Source data Fig. 1 [file 44318_2025_547_MOESM5_ESM.zip › Figure 1C/2-1 rotated and cut image.tif]

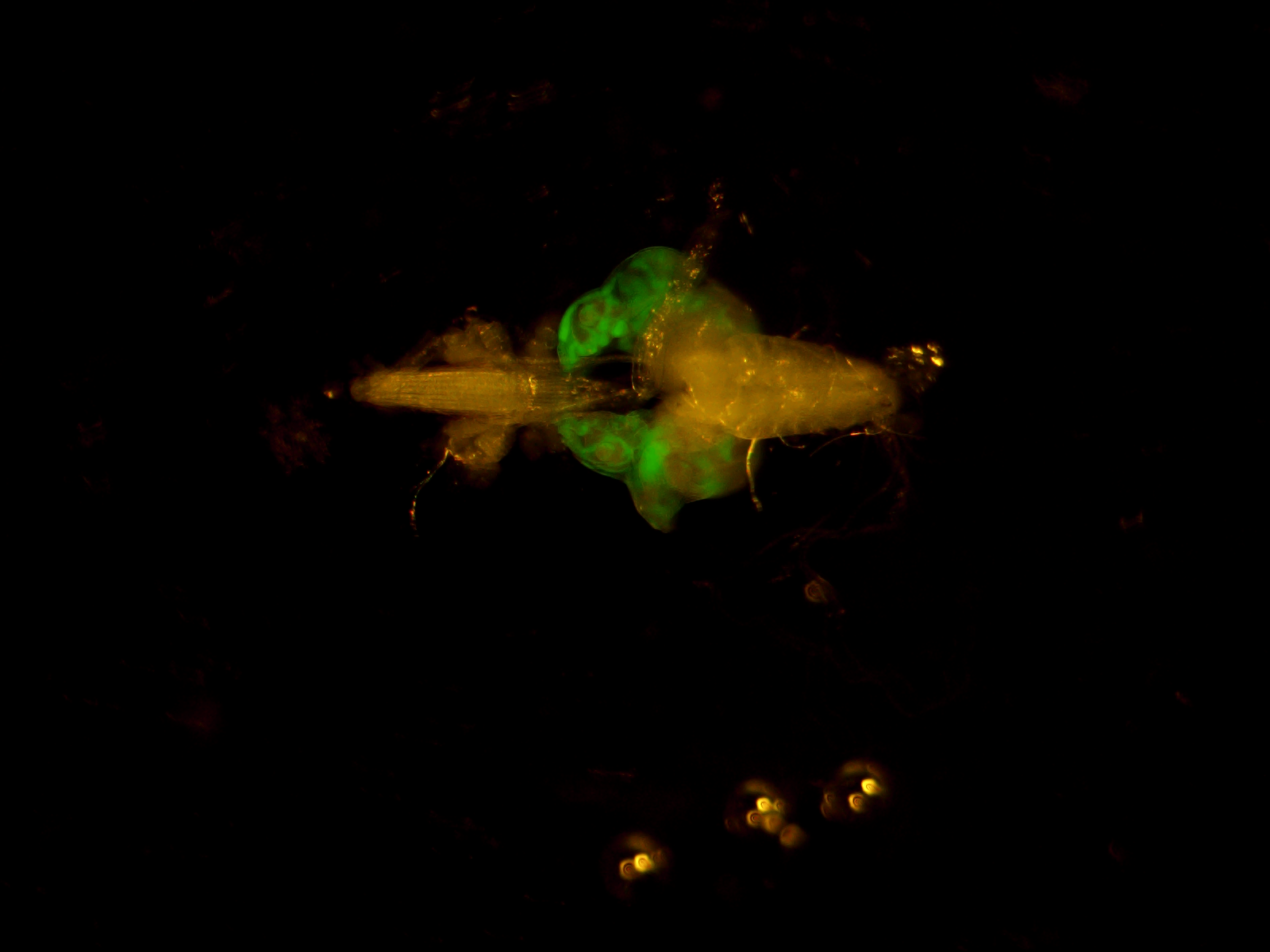

Supplement: Supplementary file 5 — Source data Fig. 1 [file 44318_2025_547_MOESM5_ESM.zip › Figure 1C/2-2 original image.tif]

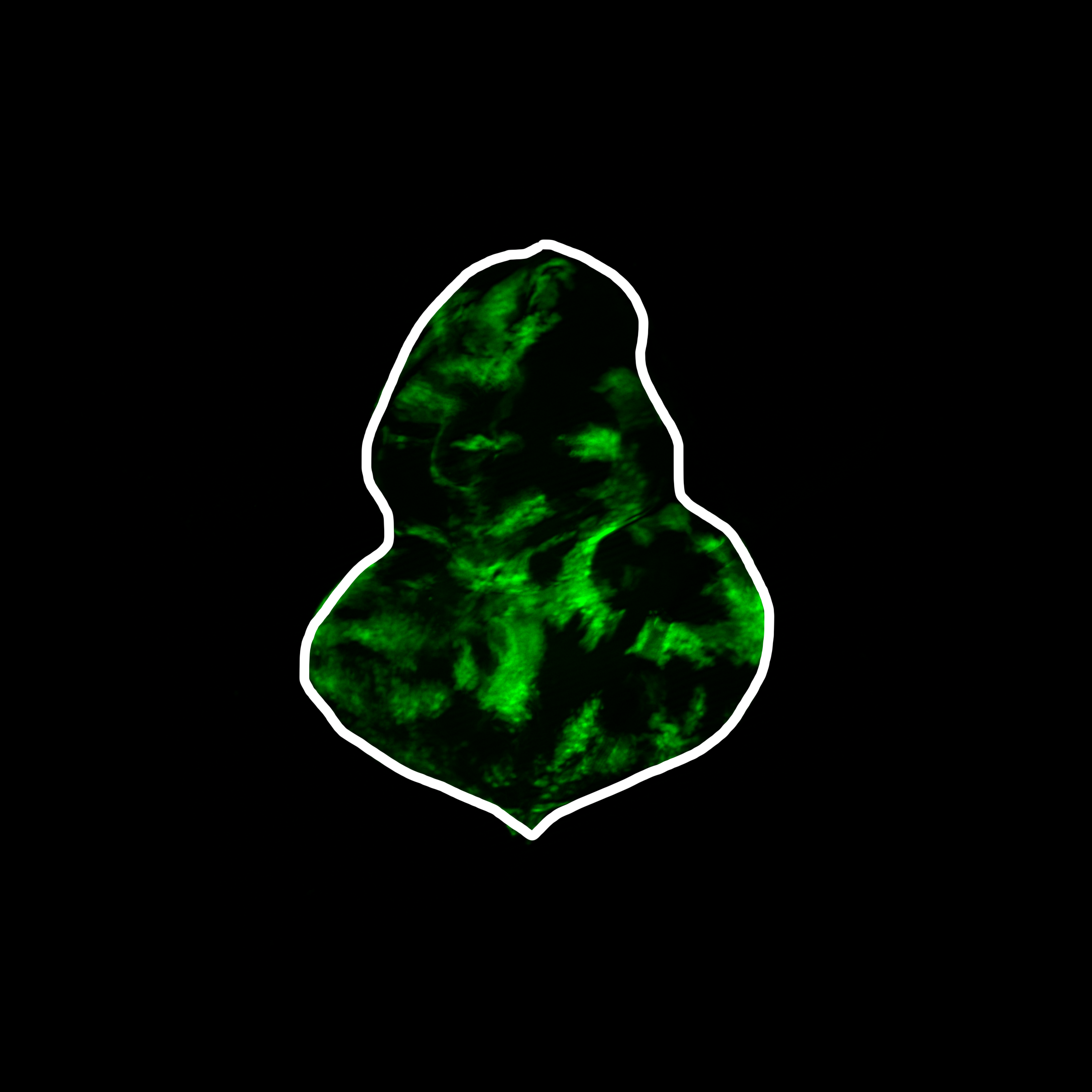

Supplement: Supplementary file 5 — Source data Fig. 1 [file 44318_2025_547_MOESM5_ESM.zip › Figure 1C/3-1 rotated and cut image with border line.tif]

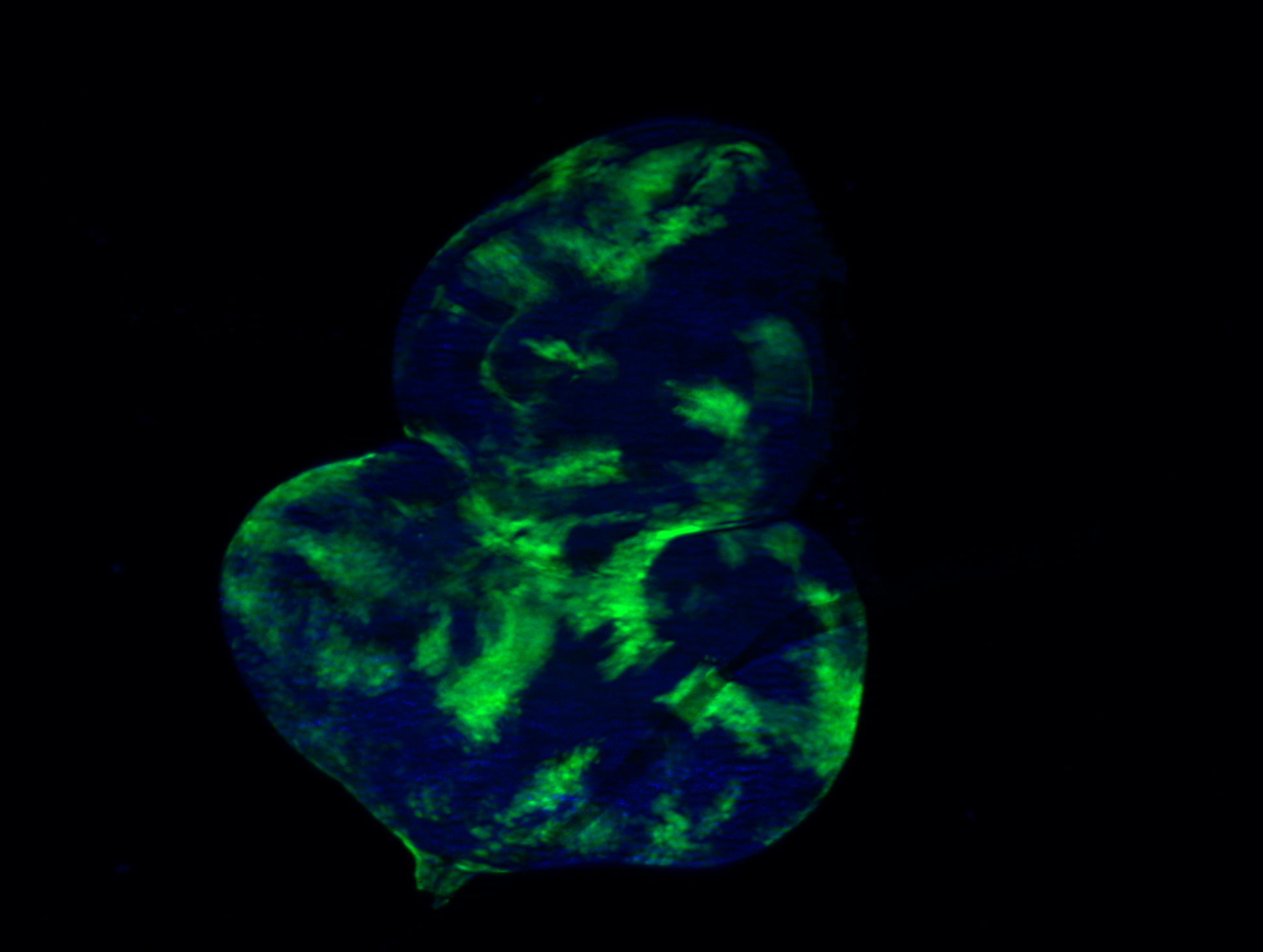

Supplement: Supplementary file 5 — Source data Fig. 1 [file 44318_2025_547_MOESM5_ESM.zip › Figure 1C/3-2 original image.tif]

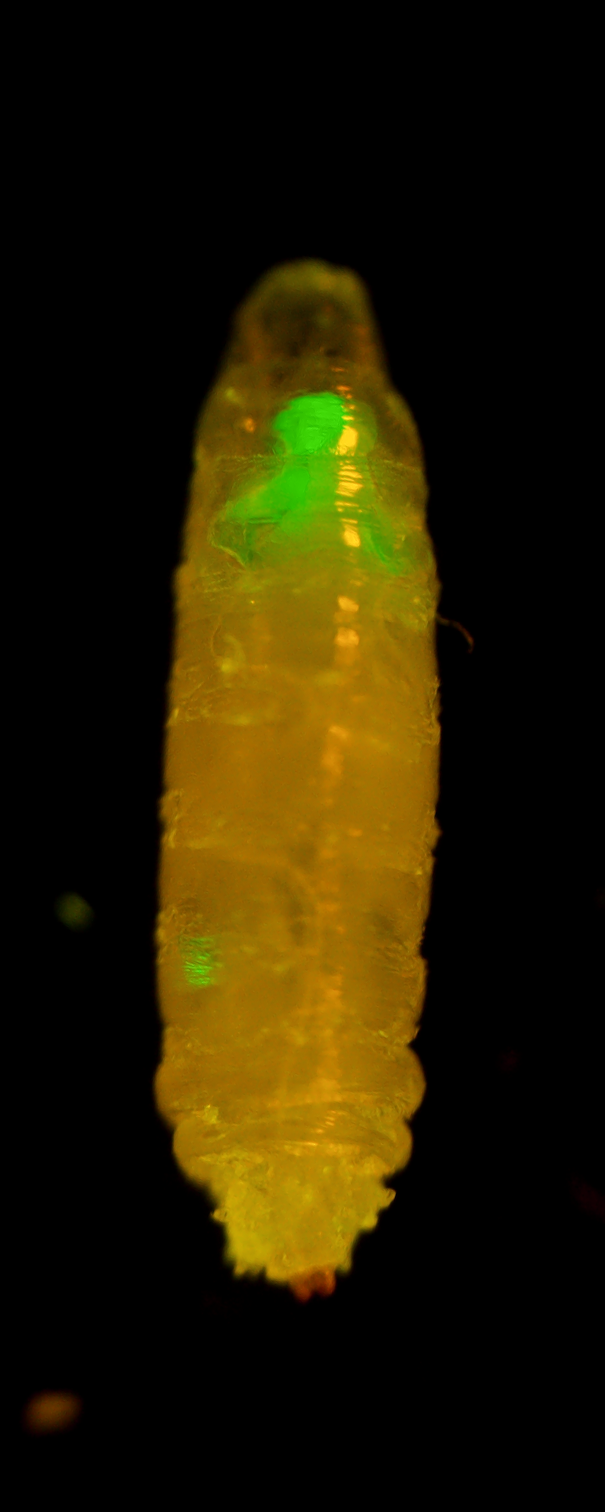

Supplement: Supplementary file 5 — Source data Fig. 1 [file 44318_2025_547_MOESM5_ESM.zip › Figure 1C/4-1 rotated and cut image.tif]

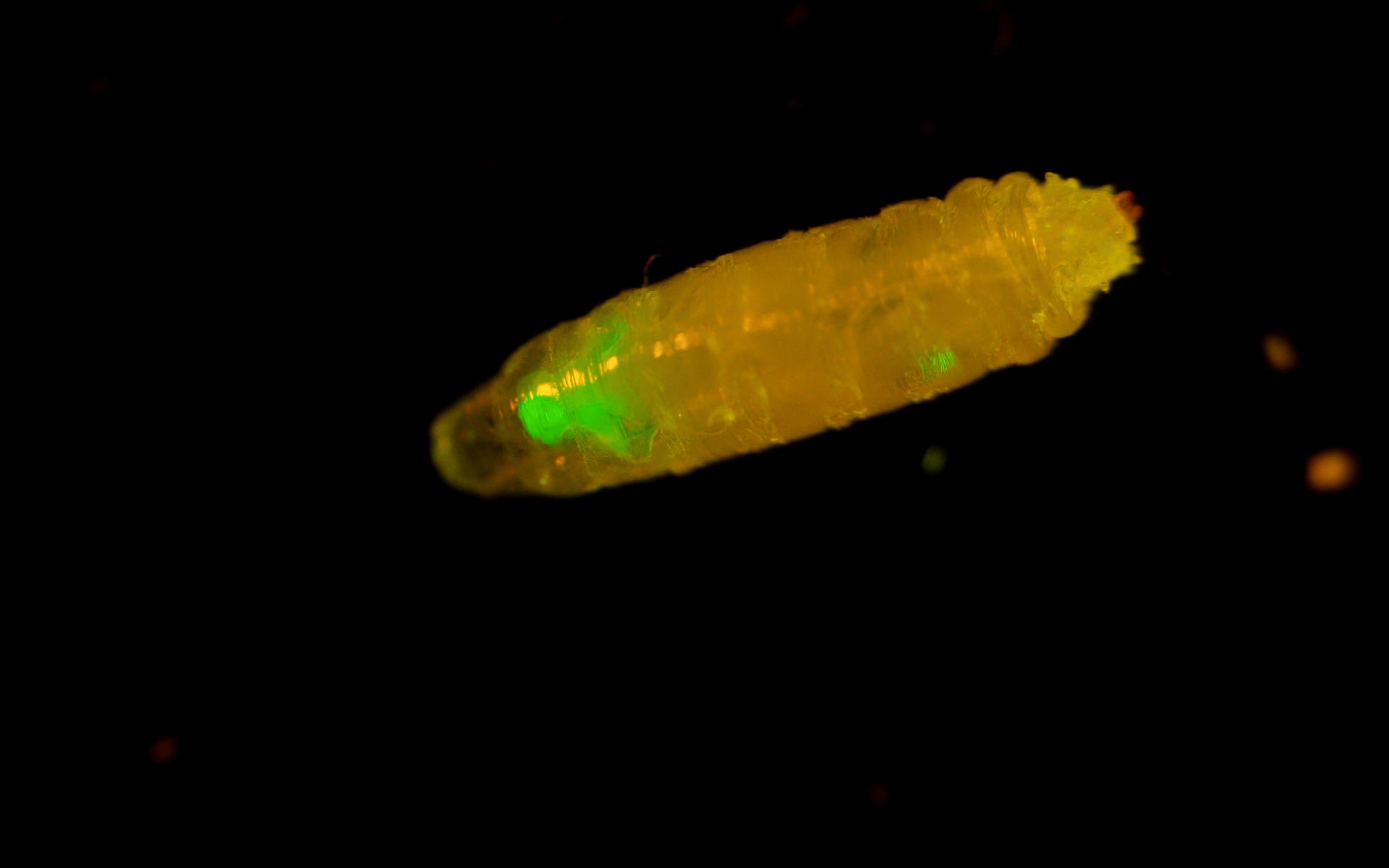

Supplement: Supplementary file 5 — Source data Fig. 1 [file 44318_2025_547_MOESM5_ESM.zip › Figure 1C/4-2 original image.tif]

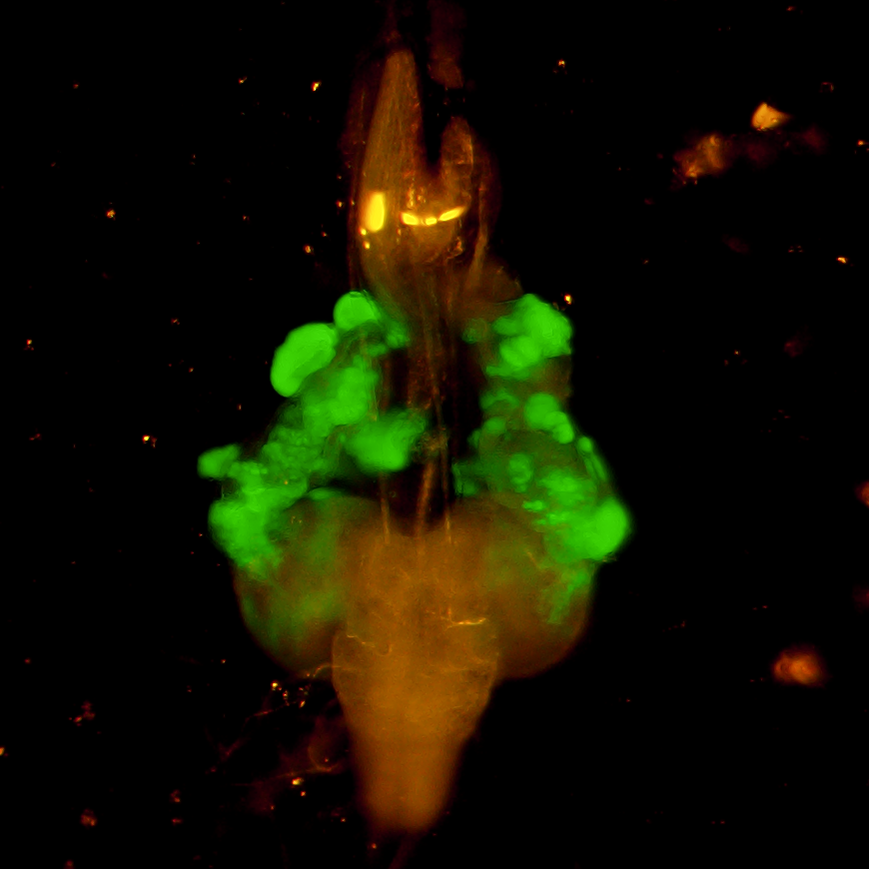

Supplement: Supplementary file 5 — Source data Fig. 1 [file 44318_2025_547_MOESM5_ESM.zip › Figure 1C/5-1 rotated and cut image.tif]

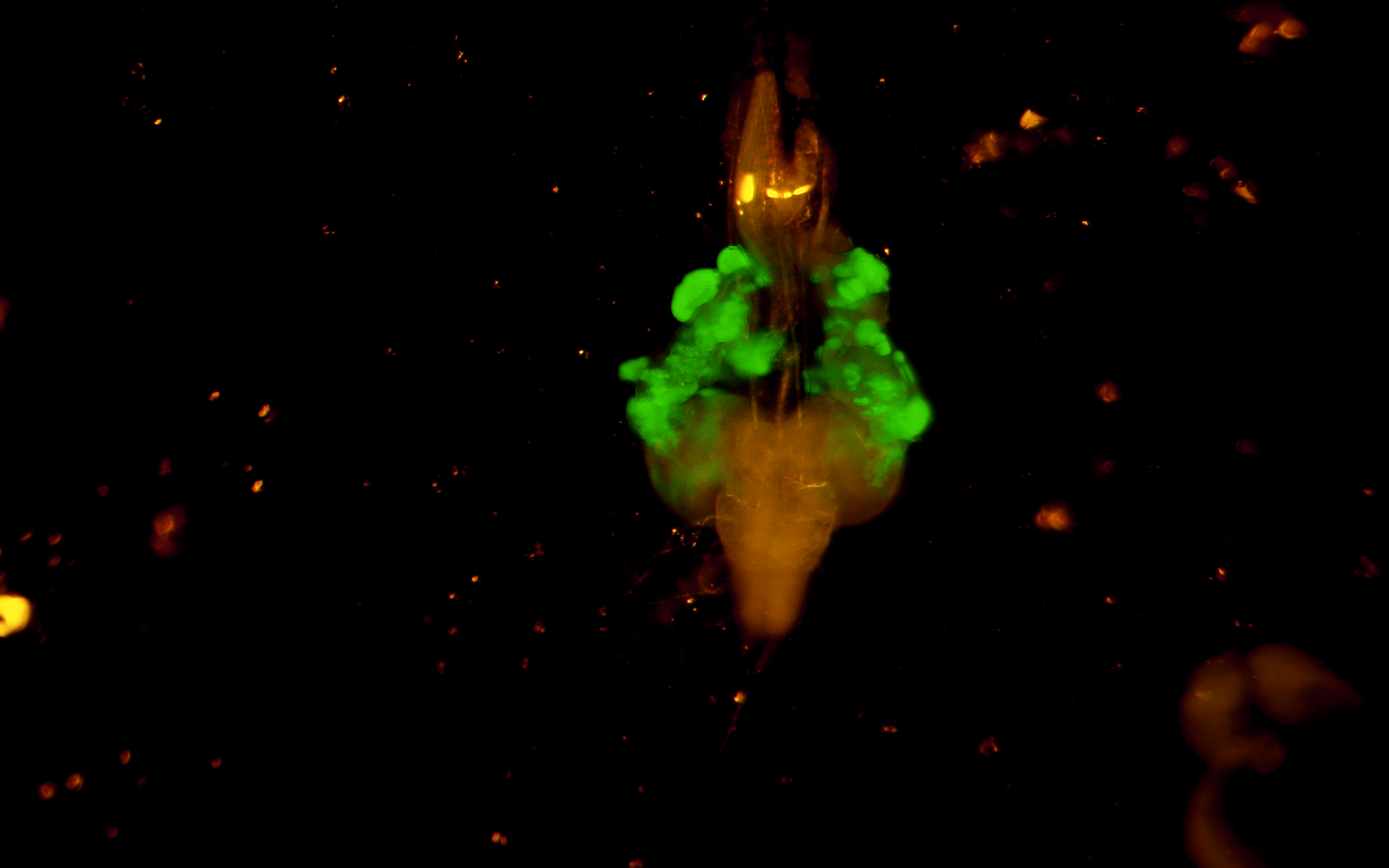

Supplement: Supplementary file 5 — Source data Fig. 1 [file 44318_2025_547_MOESM5_ESM.zip › Figure 1C/5-2 original image.tif]

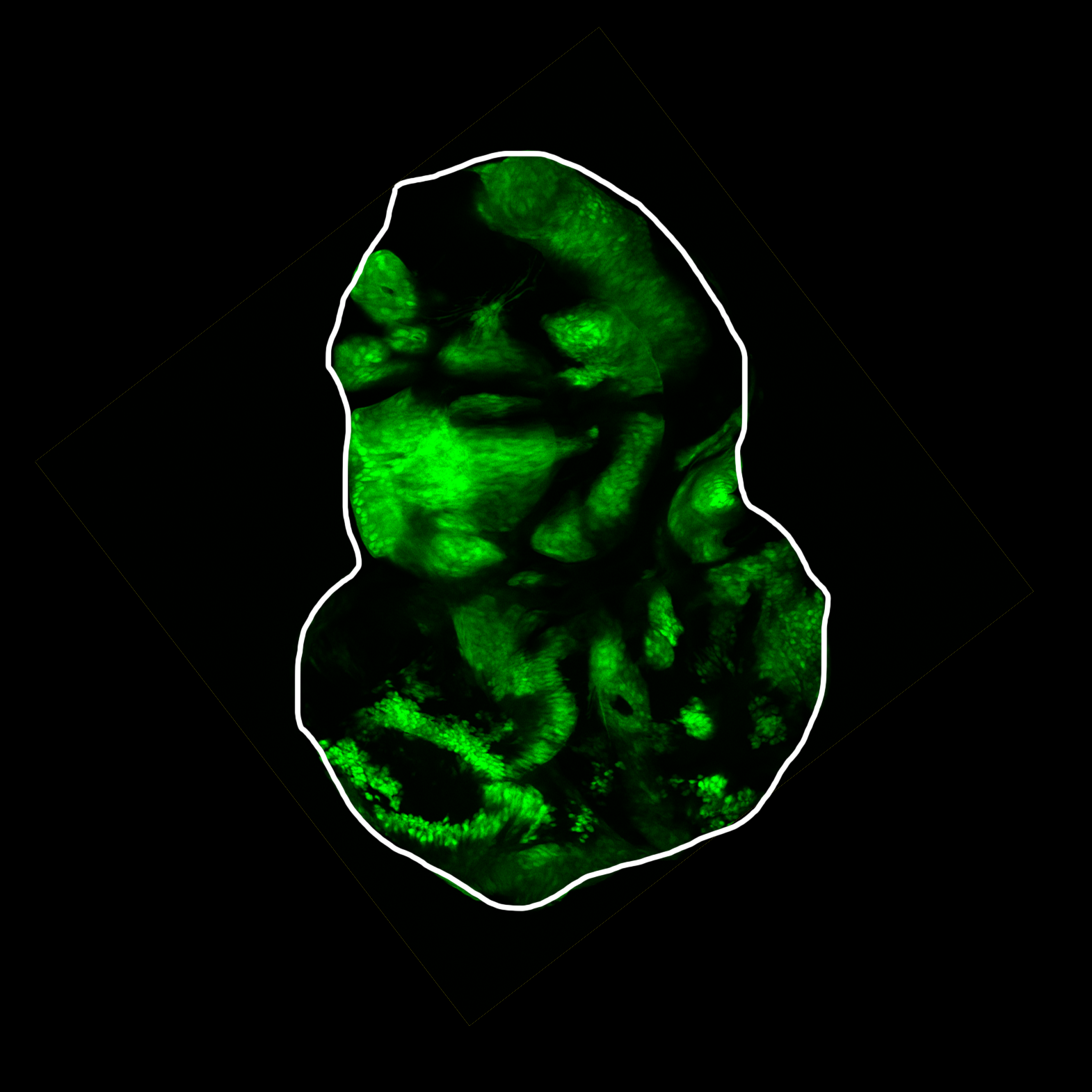

Supplement: Supplementary file 5 — Source data Fig. 1 [file 44318_2025_547_MOESM5_ESM.zip › Figure 1C/6-1 rotated and cut image with border line.tif]

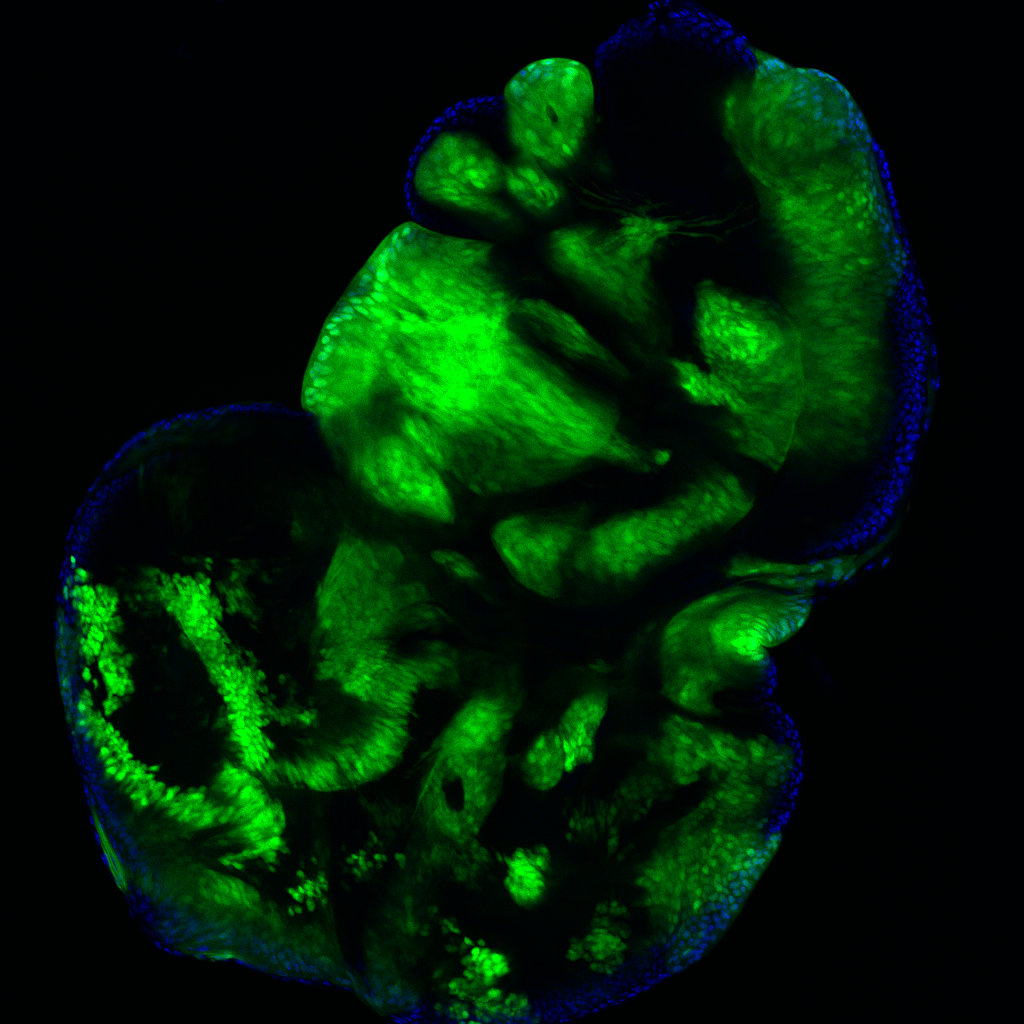

Supplement: Supplementary file 5 — Source data Fig. 1 [file 44318_2025_547_MOESM5_ESM.zip › Figure 1C/6-2 original image.tif]

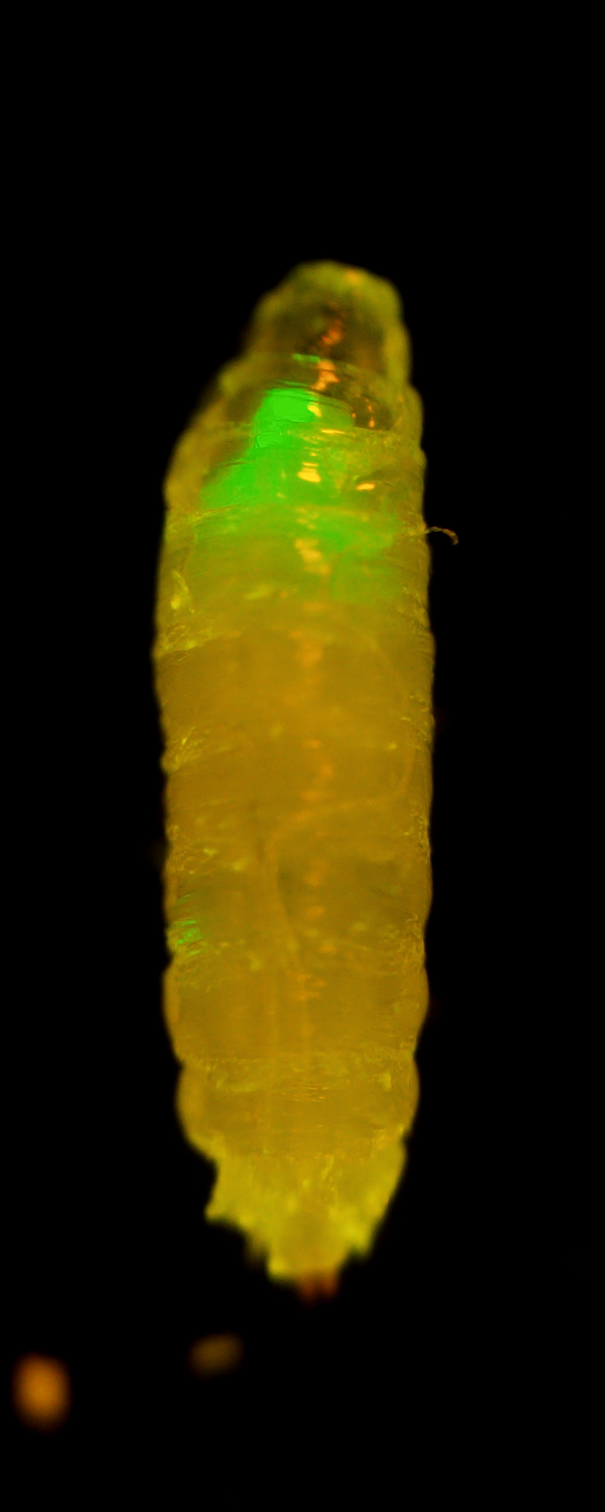

Supplement: Supplementary file 5 — Source data Fig. 1 [file 44318_2025_547_MOESM5_ESM.zip › Figure 1C/7-1 rotated and cut image.tif]

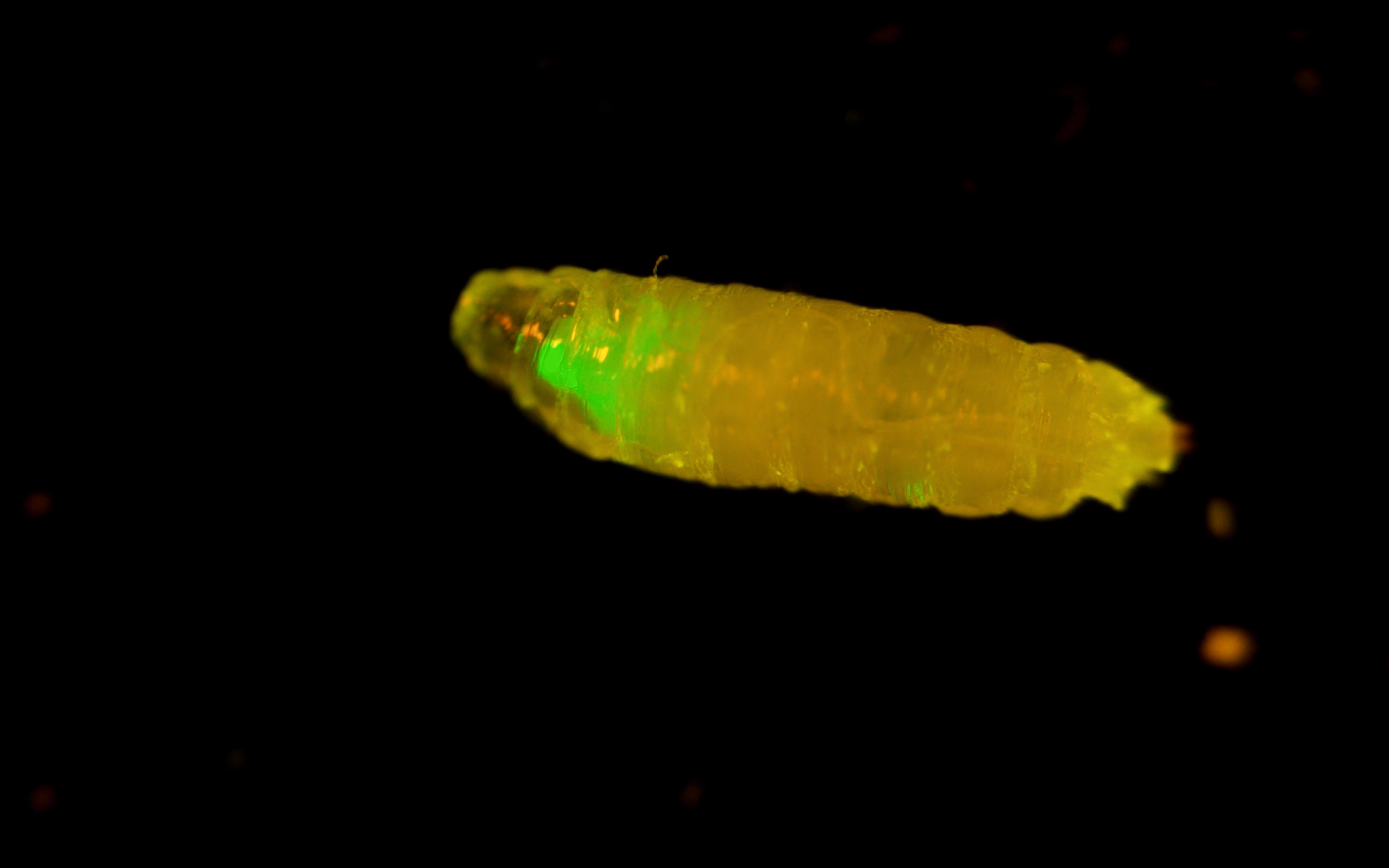

Supplement: Supplementary file 5 — Source data Fig. 1 [file 44318_2025_547_MOESM5_ESM.zip › Figure 1C/7-2 original image.tif]

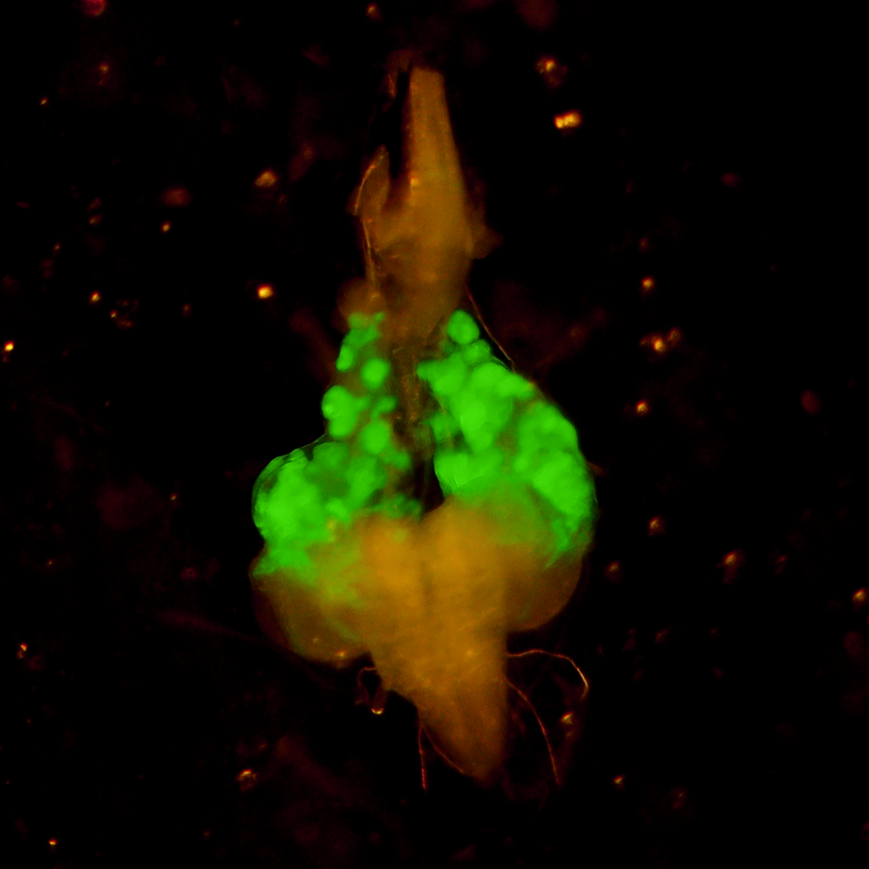

Supplement: Supplementary file 5 — Source data Fig. 1 [file 44318_2025_547_MOESM5_ESM.zip › Figure 1C/8-1 rotated and cut image.tif]

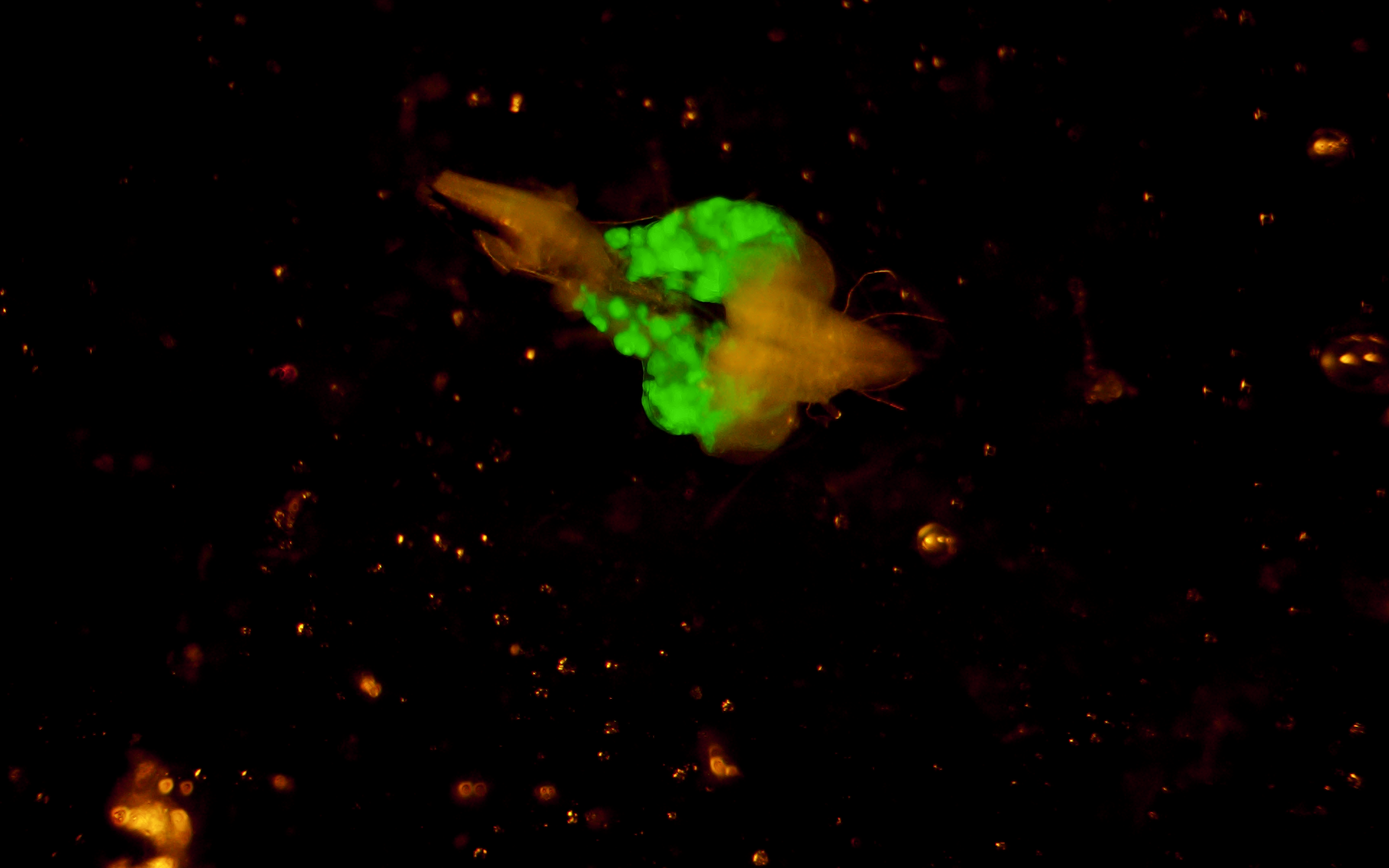

Supplement: Supplementary file 5 — Source data Fig. 1 [file 44318_2025_547_MOESM5_ESM.zip › Figure 1C/8-2 original image.tif]

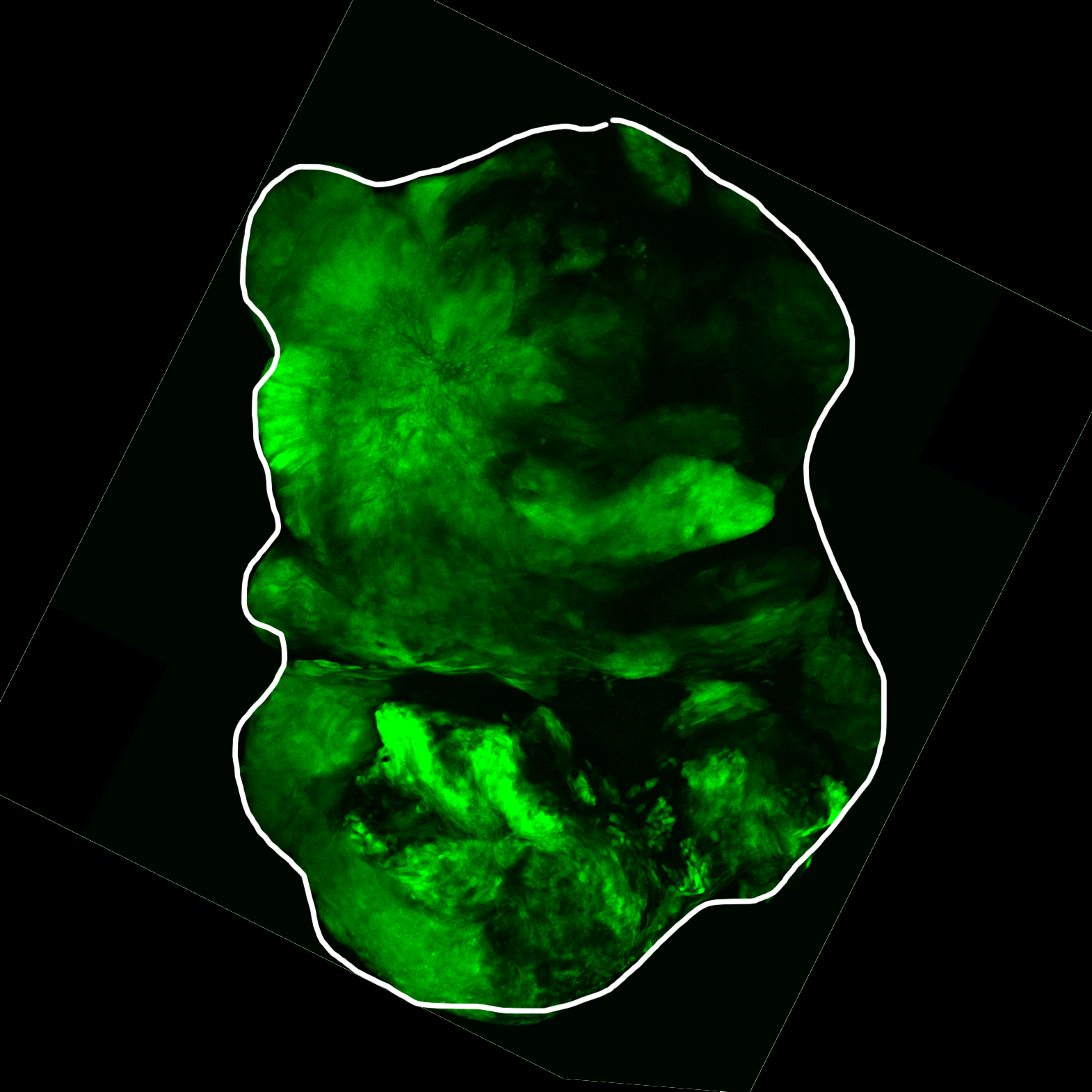

Supplement: Supplementary file 5 — Source data Fig. 1 [file 44318_2025_547_MOESM5_ESM.zip › Figure 1C/9-1 rotated and cut image with border line.tif]

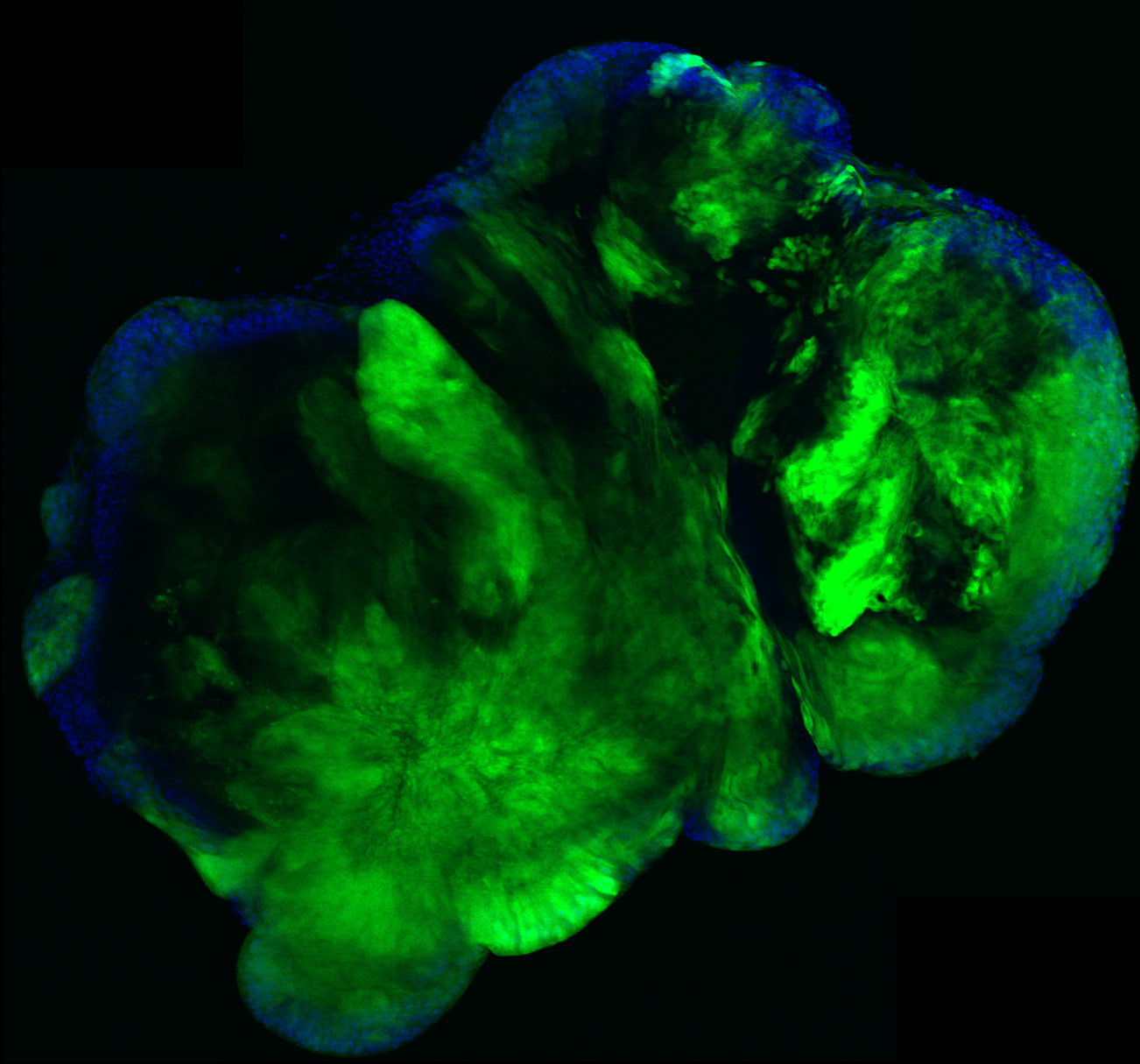

Supplement: Supplementary file 5 — Source data Fig. 1 [file 44318_2025_547_MOESM5_ESM.zip › Figure 1C/9-2 original image.tif]

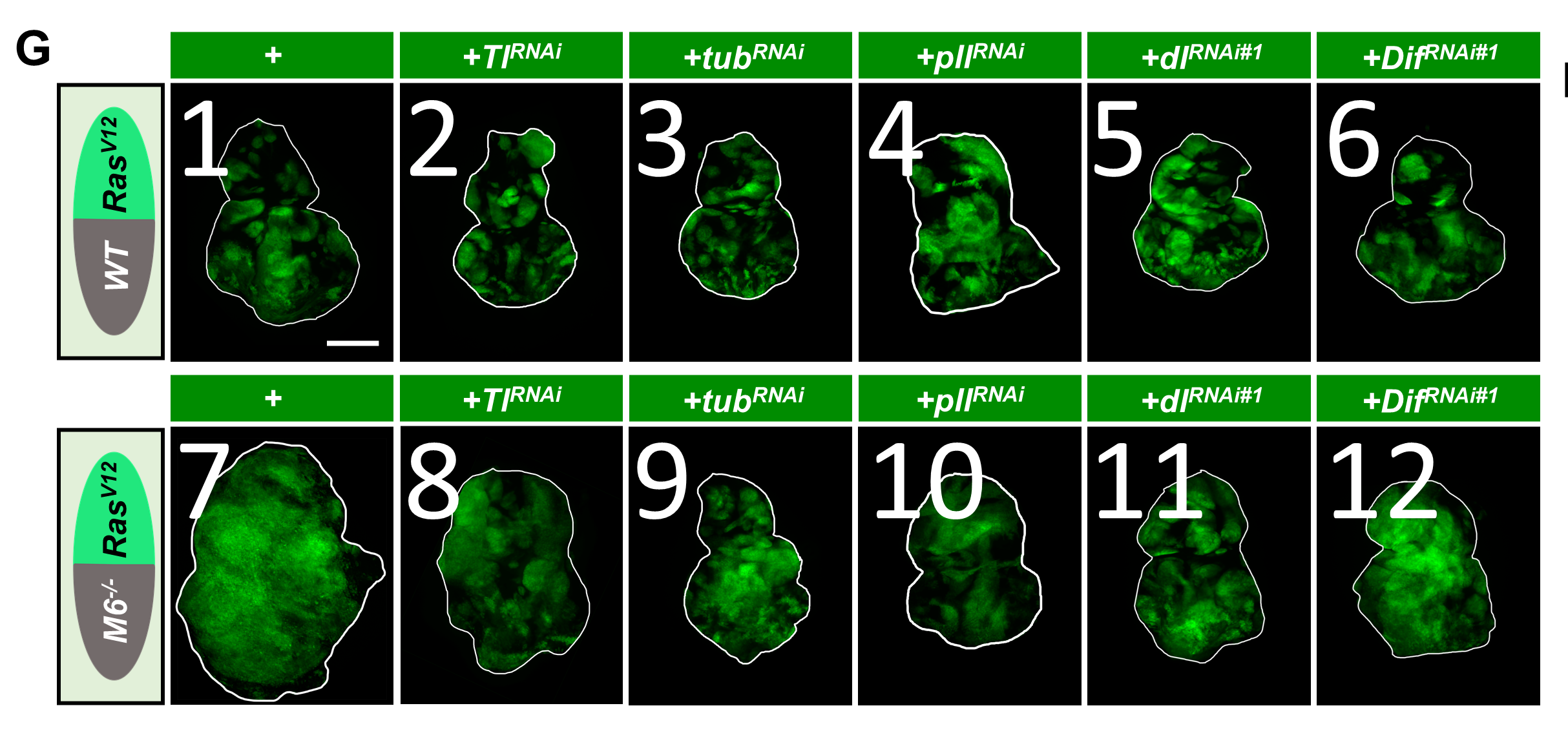

Supplement: Supplementary file 6 — Source data Fig. 2 [file 44318_2025_547_MOESM6_ESM.zip › Figure 2G/0 paper Figure 2G with provided image sequence.tif]

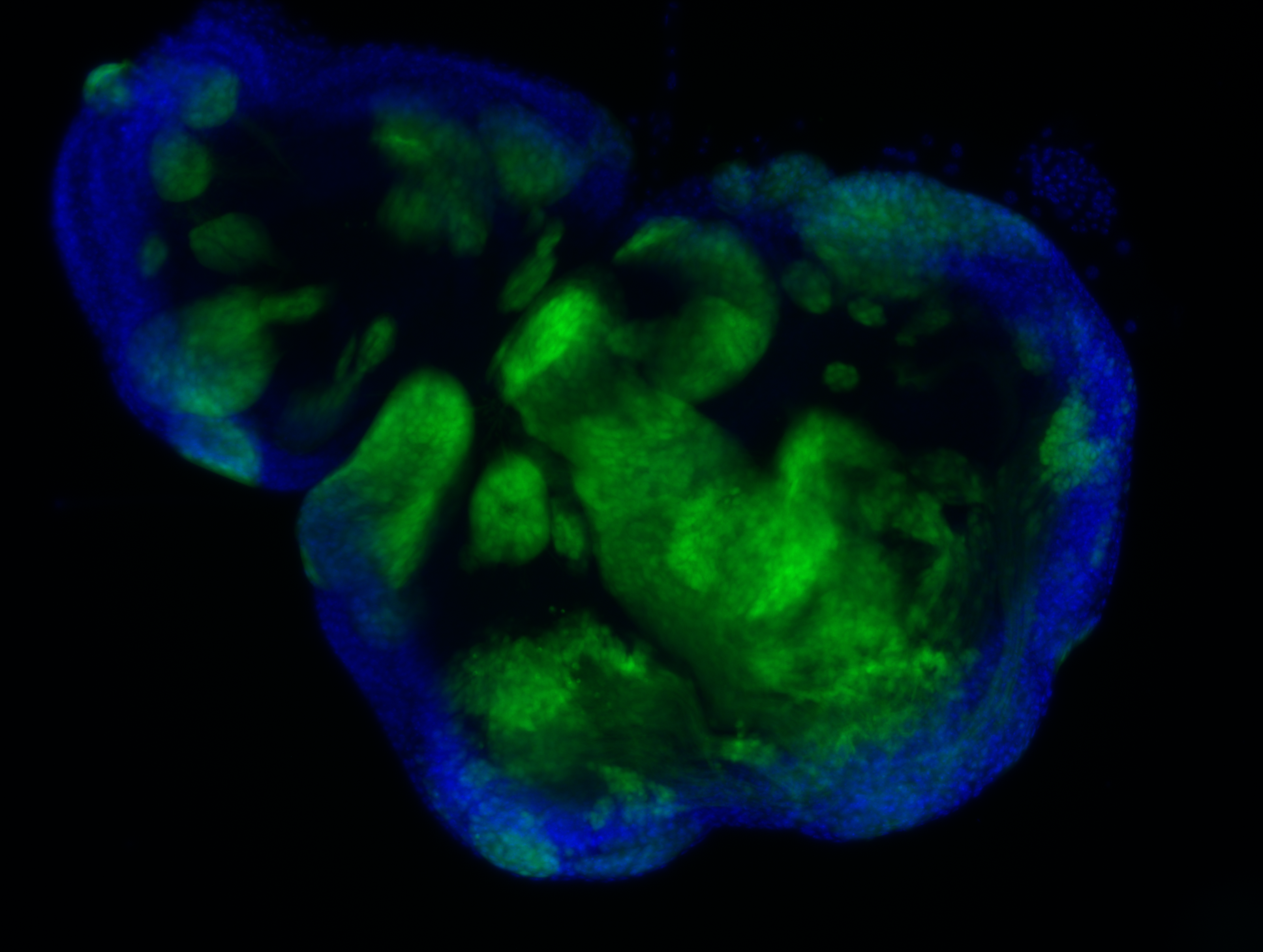

Supplement: Supplementary file 6 — Source data Fig. 2 [file 44318_2025_547_MOESM6_ESM.zip › Figure 2G/1 original image.tif]

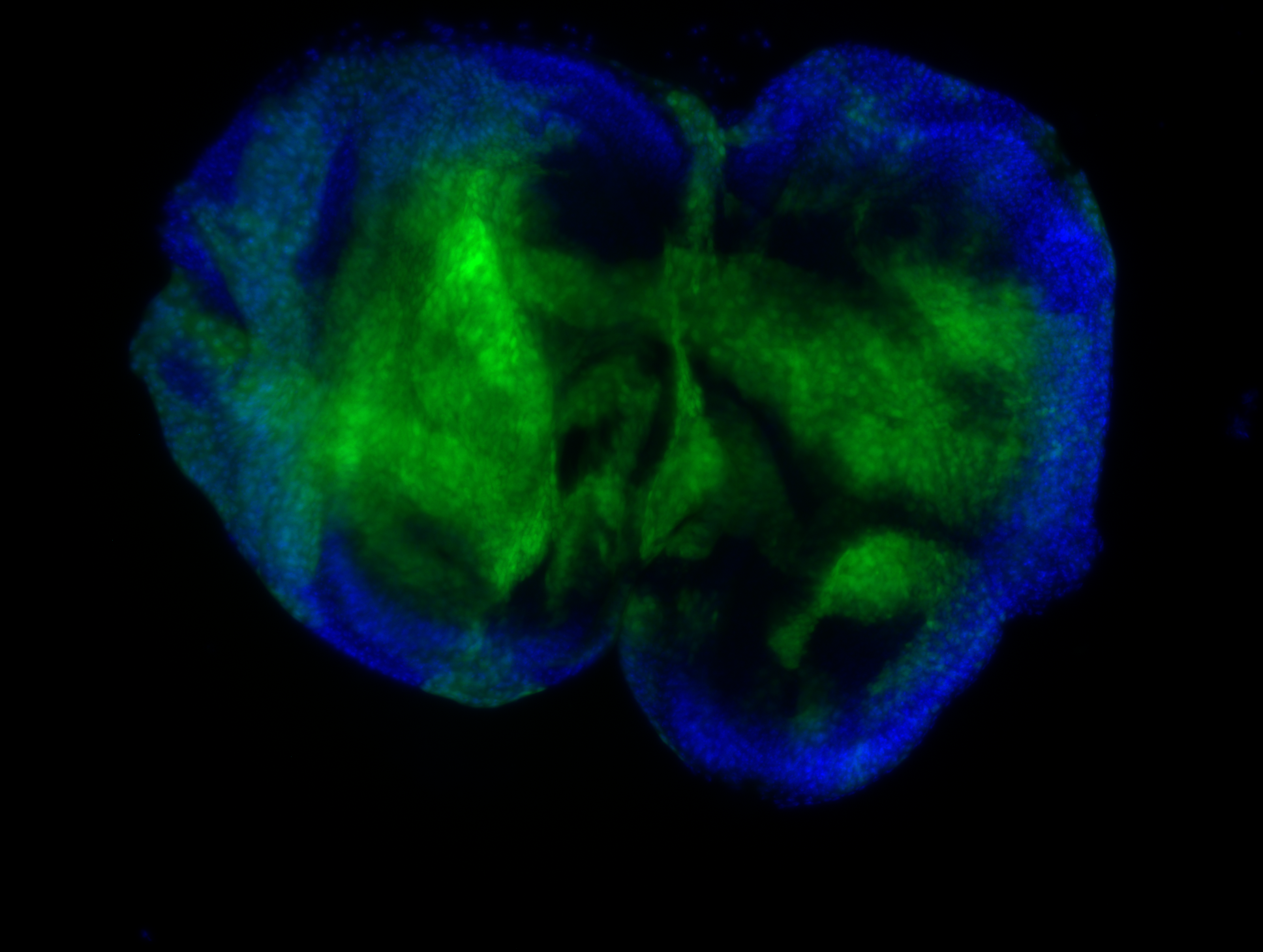

Supplement: Supplementary file 6 — Source data Fig. 2 [file 44318_2025_547_MOESM6_ESM.zip › Figure 2G/10 original image.tif]

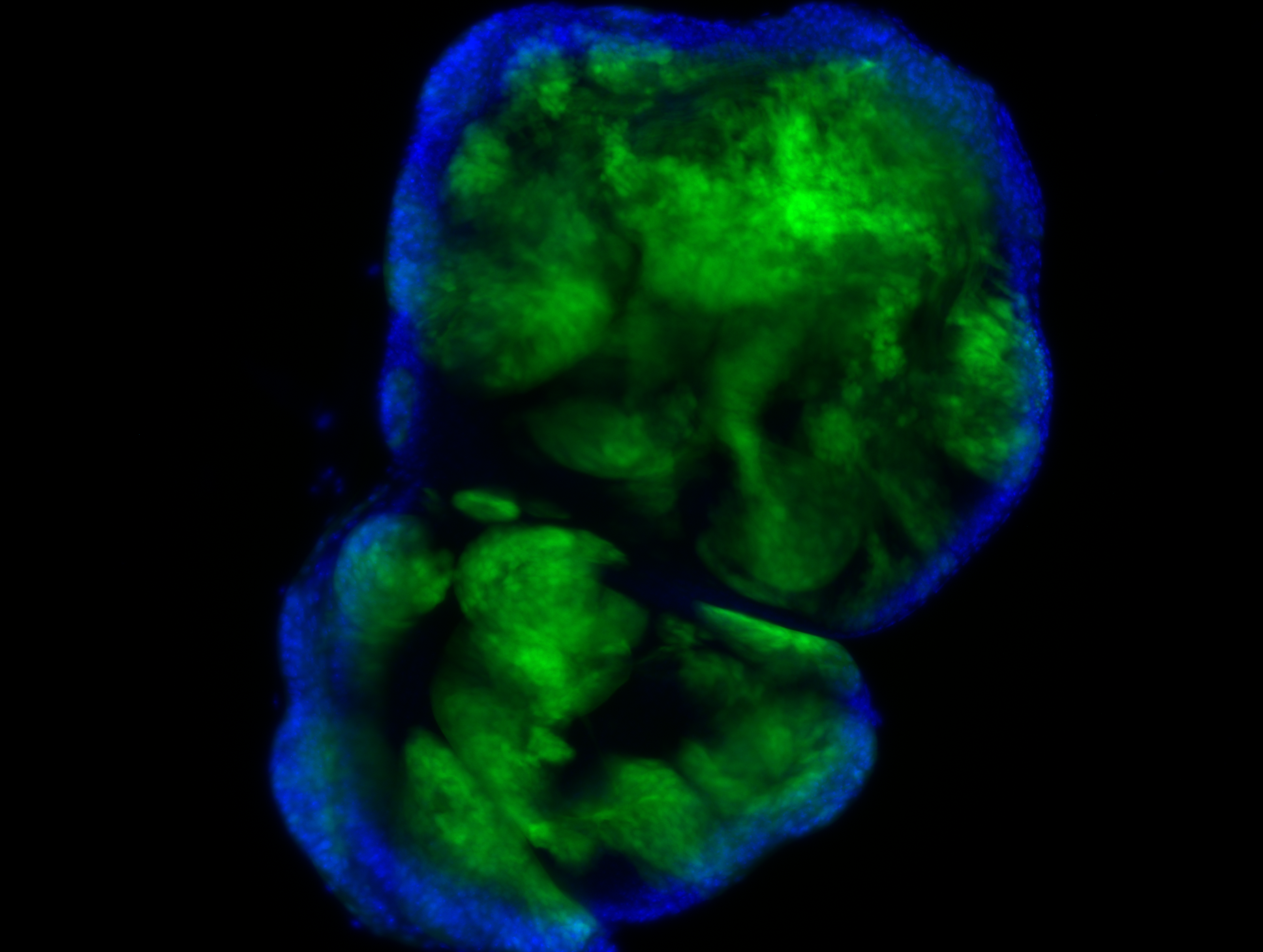

Supplement: Supplementary file 6 — Source data Fig. 2 [file 44318_2025_547_MOESM6_ESM.zip › Figure 2G/11 original image.tif]

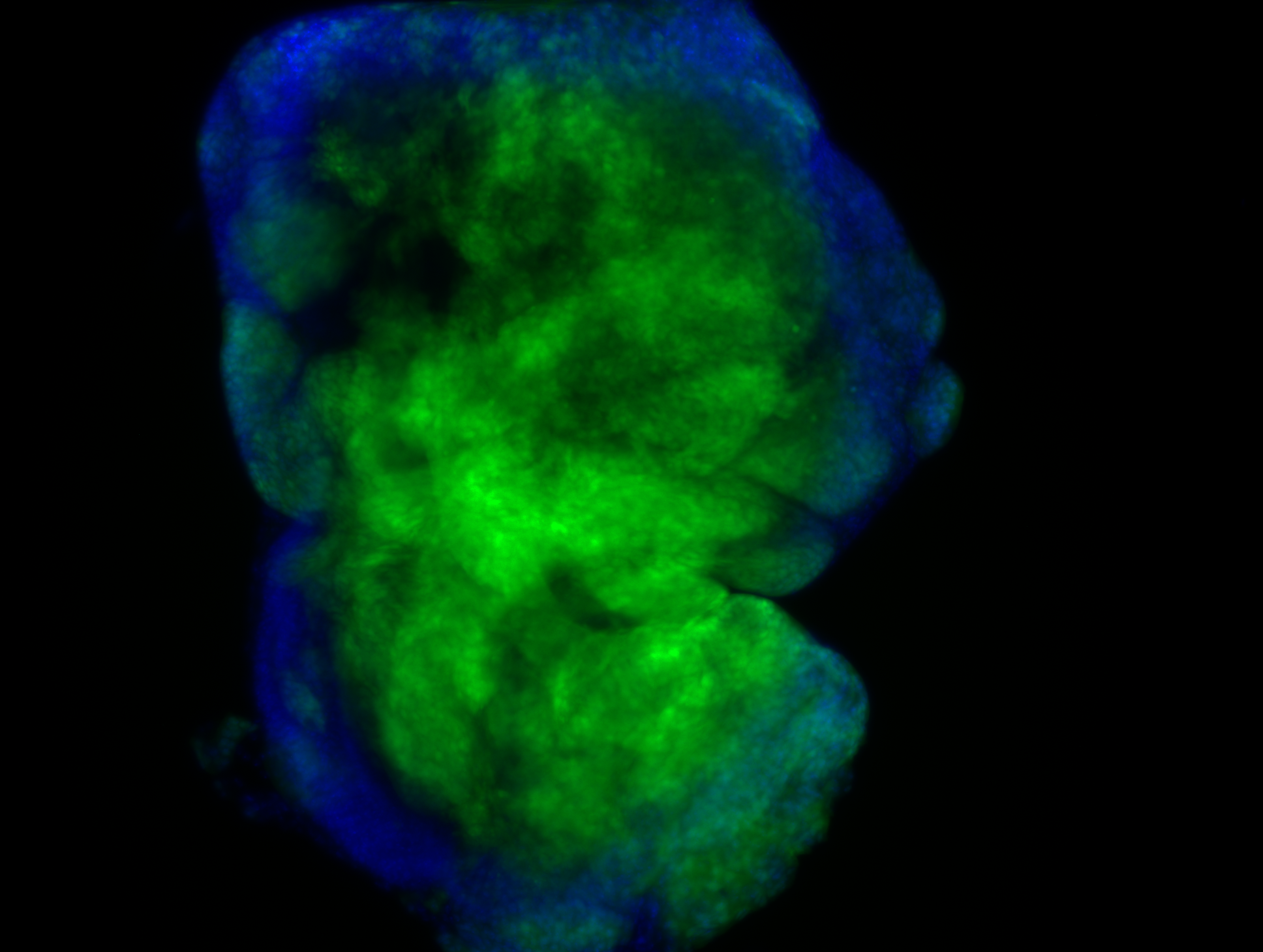

Supplement: Supplementary file 6 — Source data Fig. 2 [file 44318_2025_547_MOESM6_ESM.zip › Figure 2G/12 original image.tif]

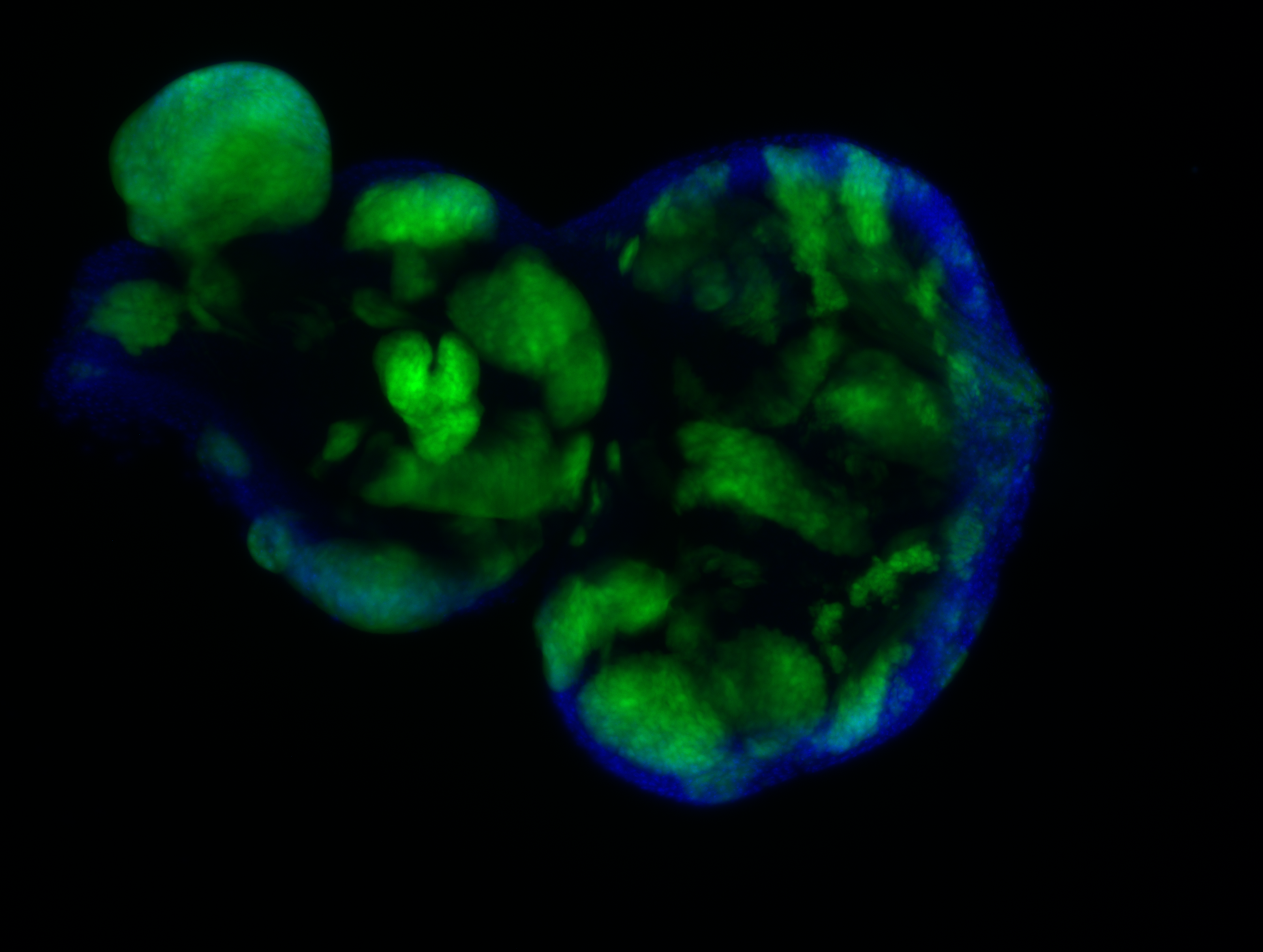

Supplement: Supplementary file 6 — Source data Fig. 2 [file 44318_2025_547_MOESM6_ESM.zip › Figure 2G/2 original image.tif]

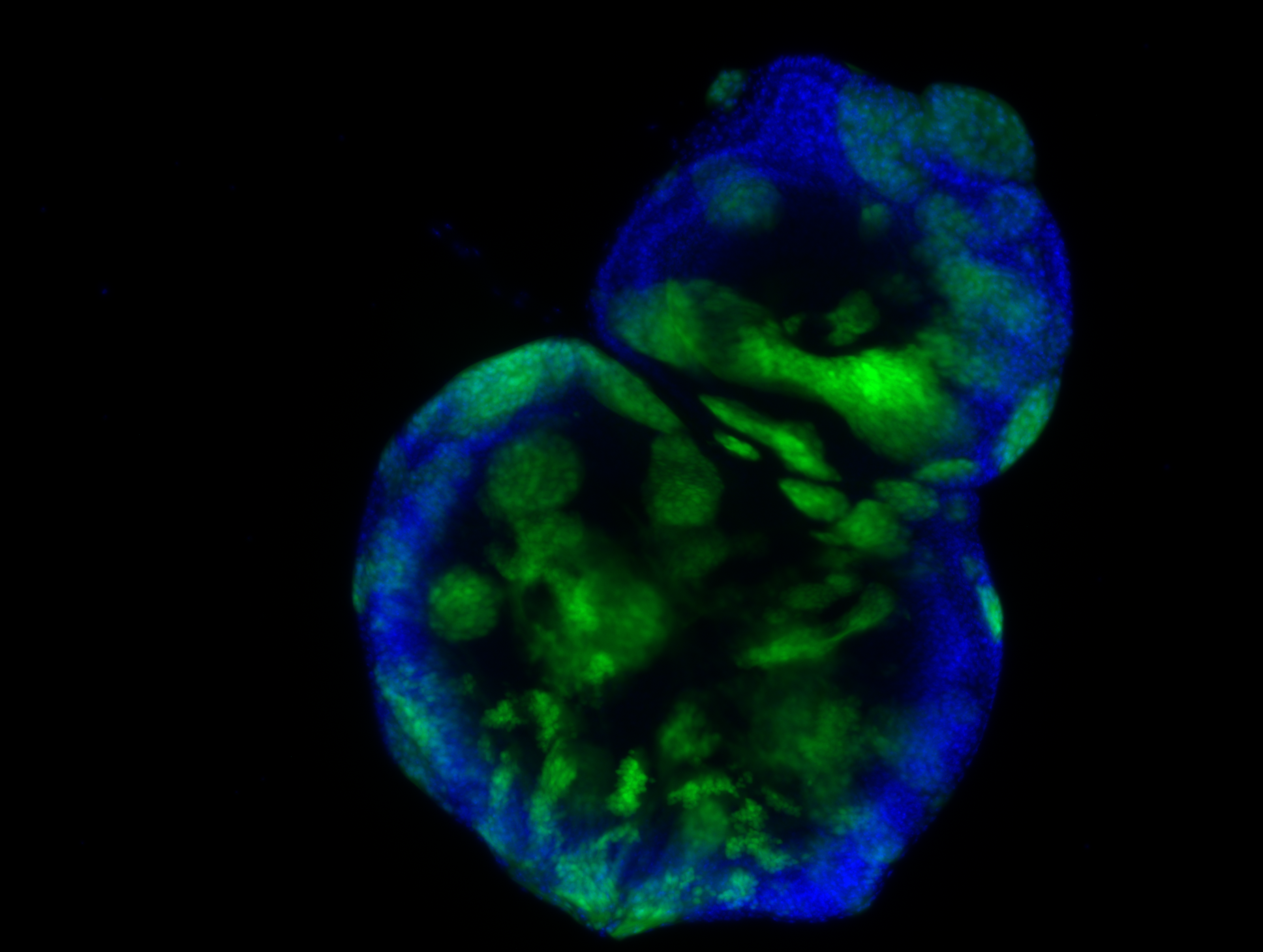

Supplement: Supplementary file 6 — Source data Fig. 2 [file 44318_2025_547_MOESM6_ESM.zip › Figure 2G/3 original image.tif]

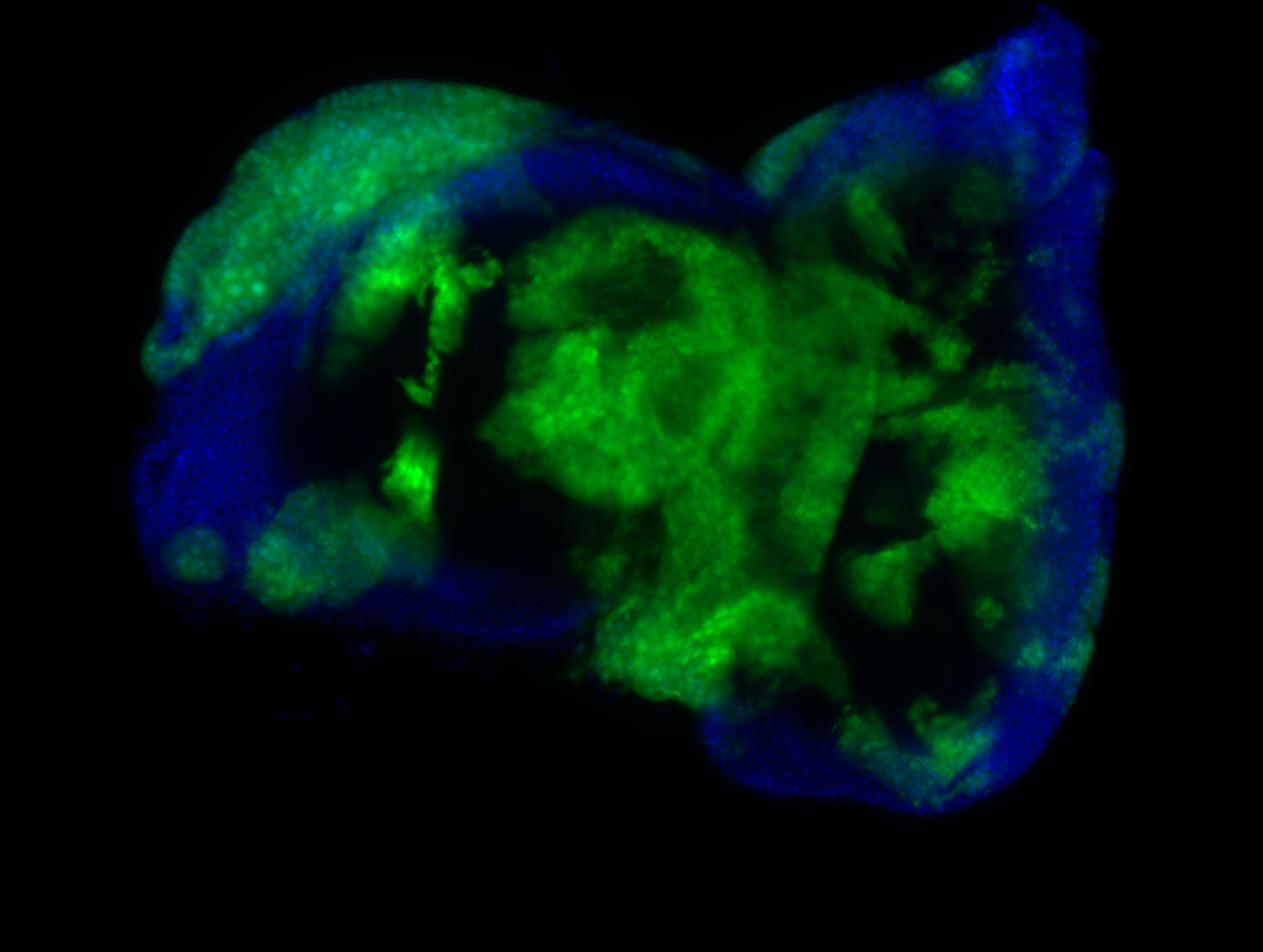

Supplement: Supplementary file 6 — Source data Fig. 2 [file 44318_2025_547_MOESM6_ESM.zip › Figure 2G/4 original image.tif]

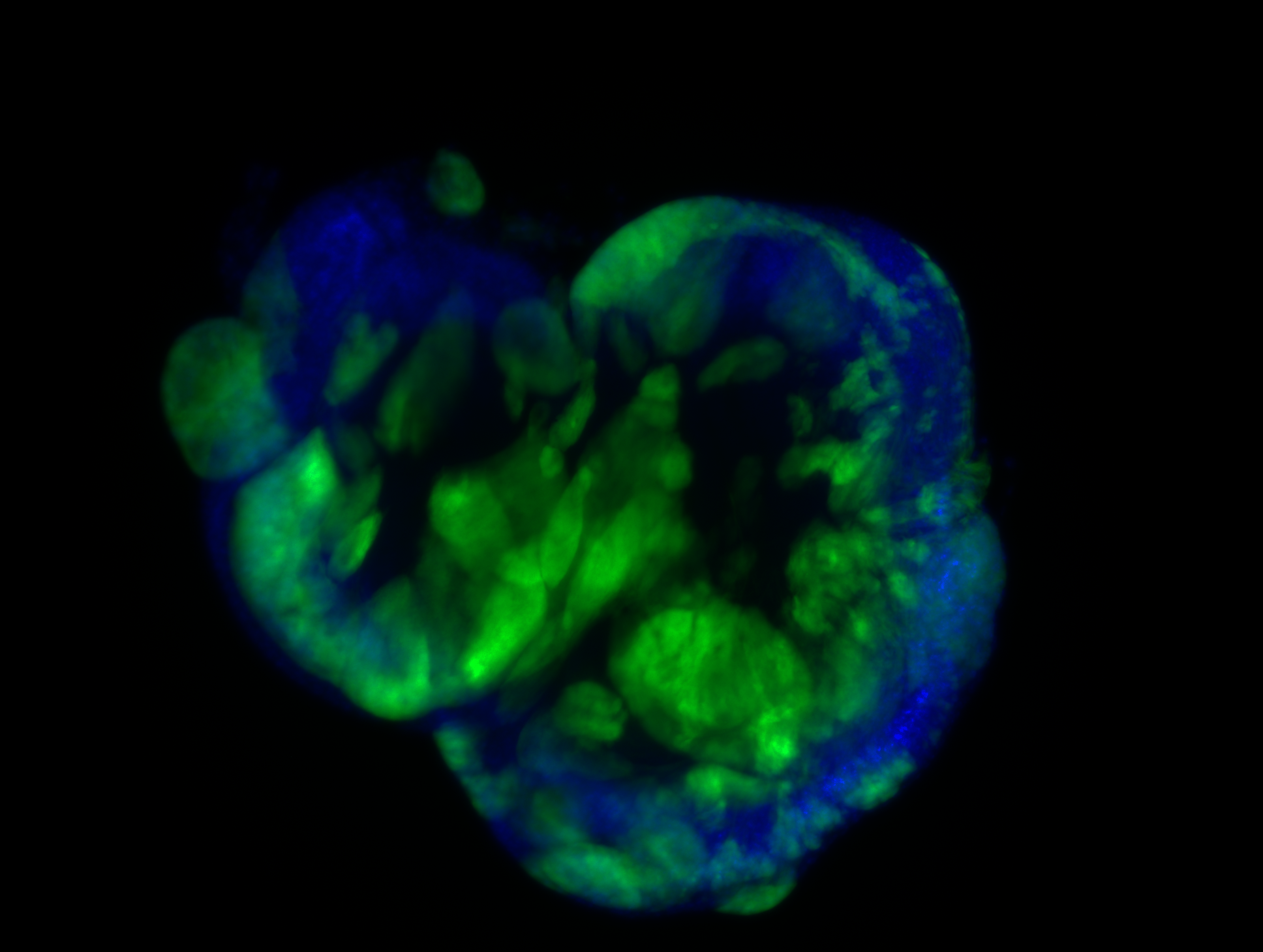

Supplement: Supplementary file 6 — Source data Fig. 2 [file 44318_2025_547_MOESM6_ESM.zip › Figure 2G/5 original image.tif]

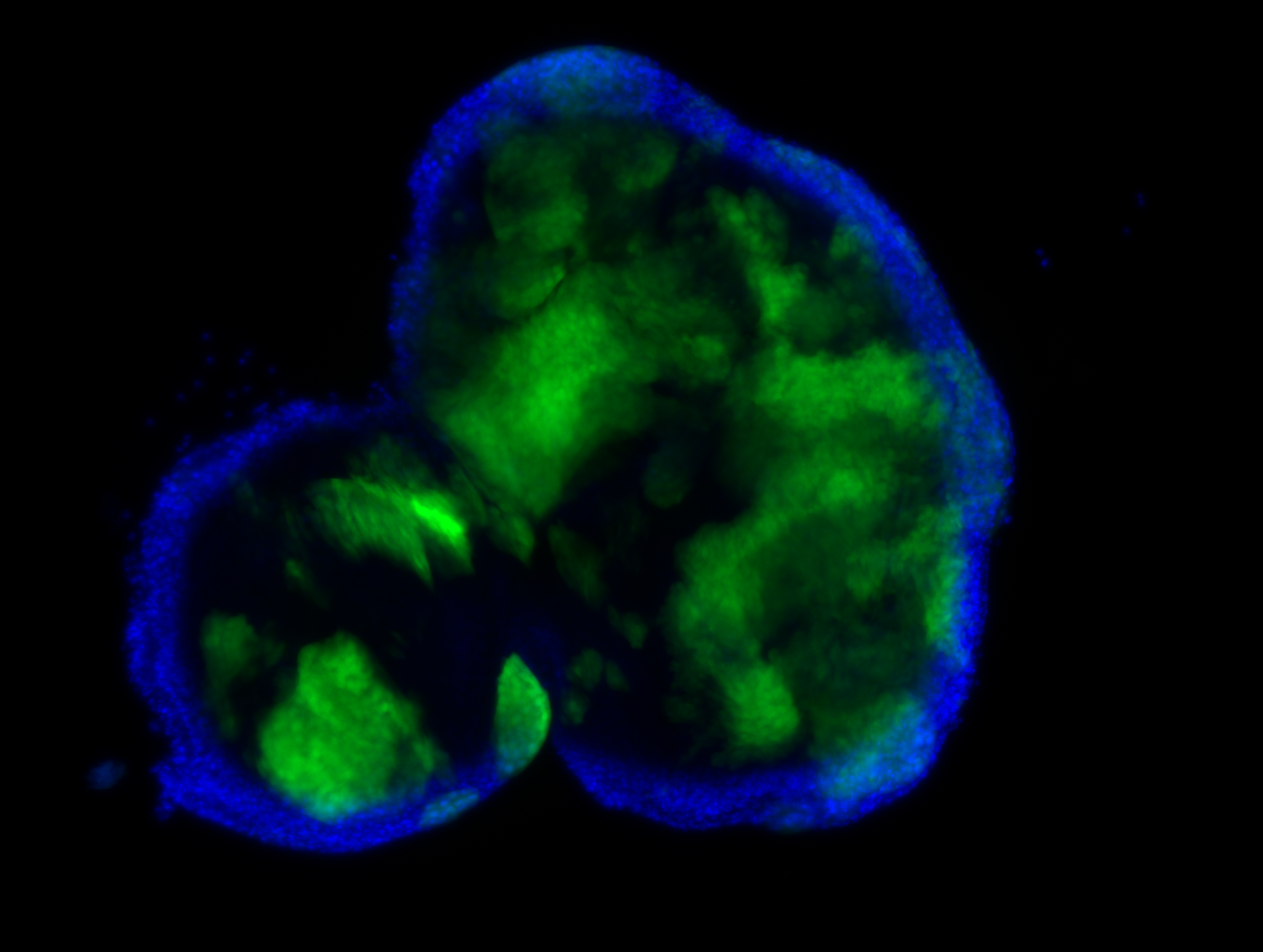

Supplement: Supplementary file 6 — Source data Fig. 2 [file 44318_2025_547_MOESM6_ESM.zip › Figure 2G/6 original image.tif]

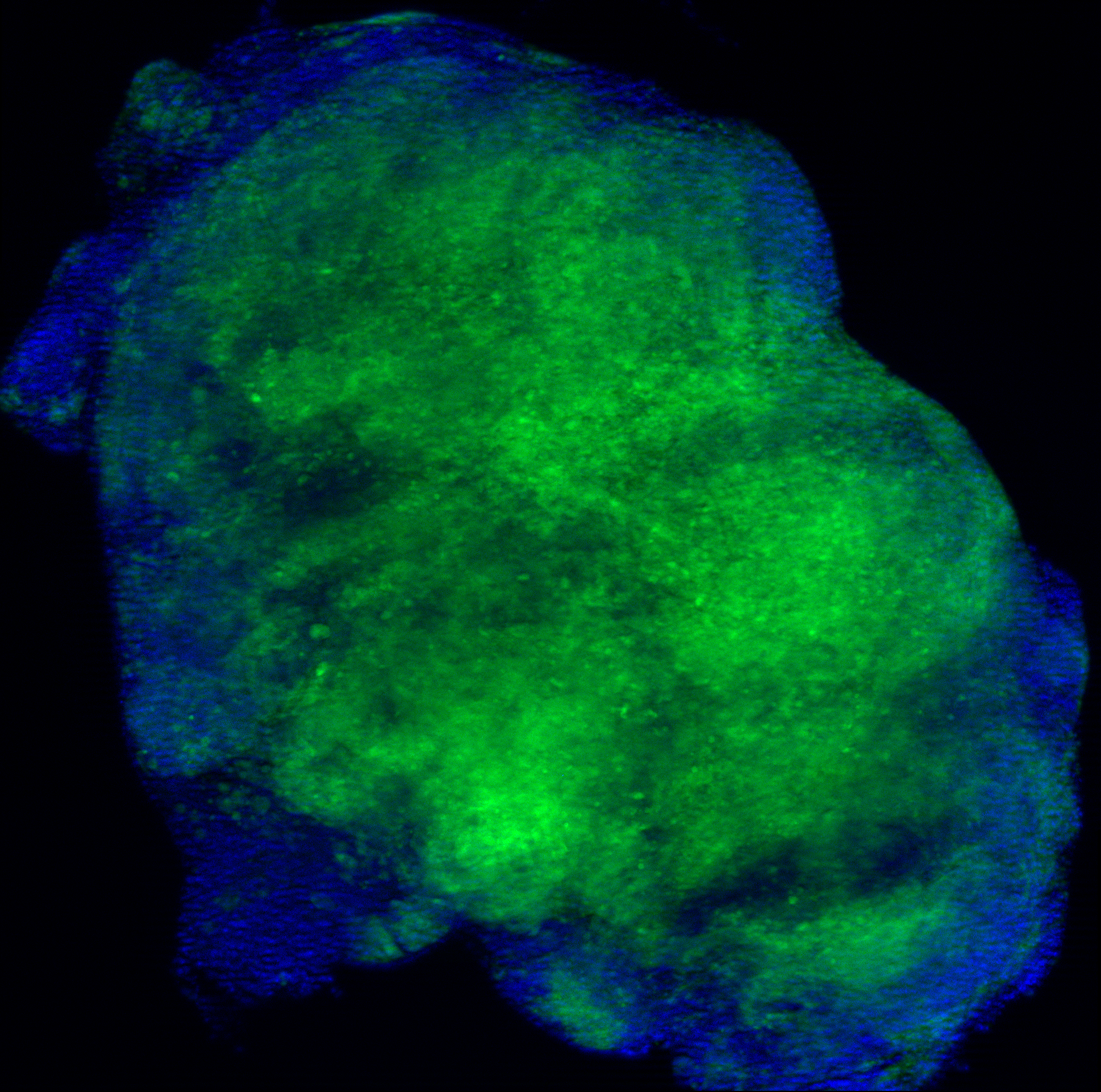

Supplement: Supplementary file 6 — Source data Fig. 2 [file 44318_2025_547_MOESM6_ESM.zip › Figure 2G/7 original image.tif]

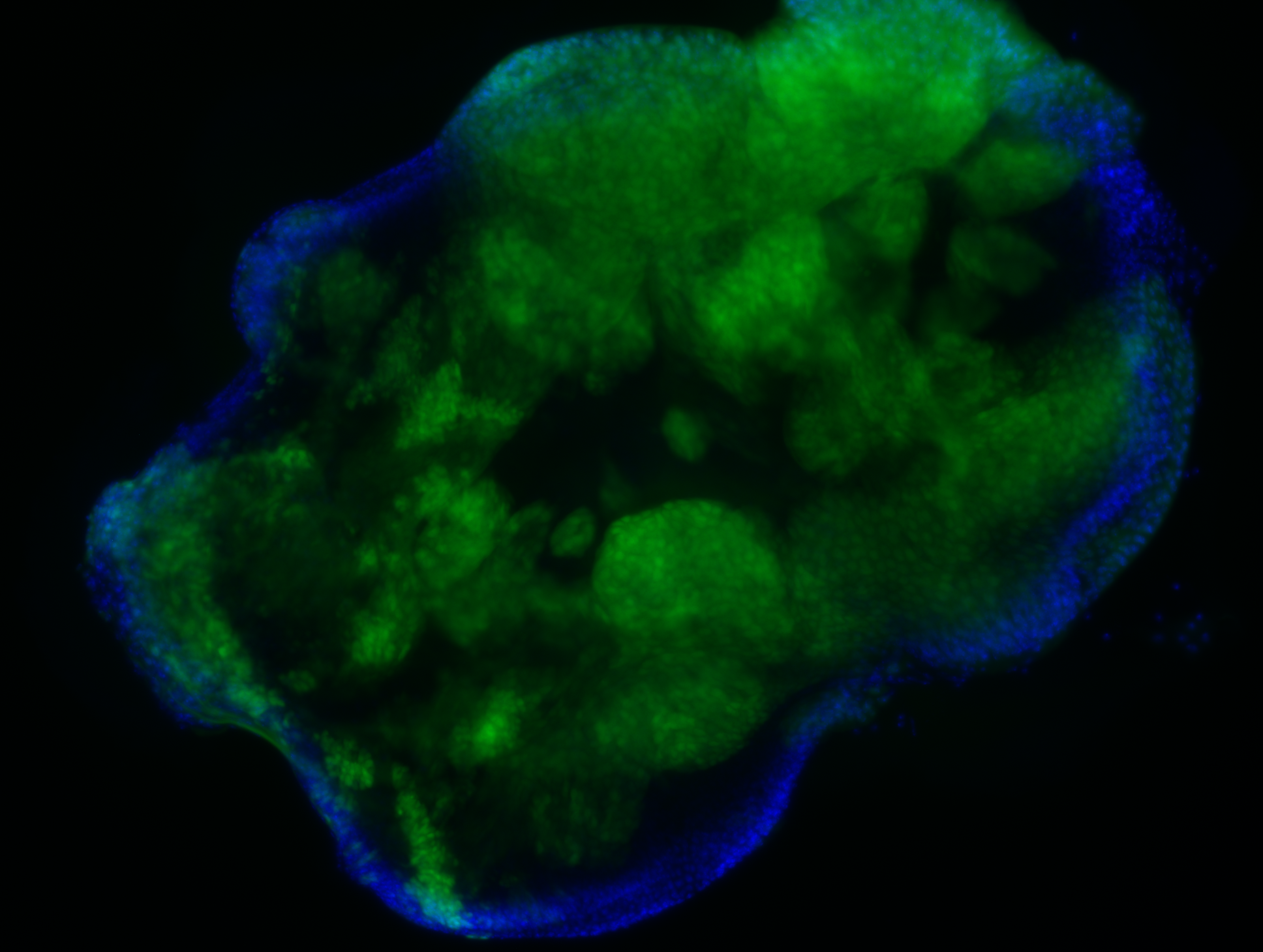

Supplement: Supplementary file 6 — Source data Fig. 2 [file 44318_2025_547_MOESM6_ESM.zip › Figure 2G/8 original image.tif]

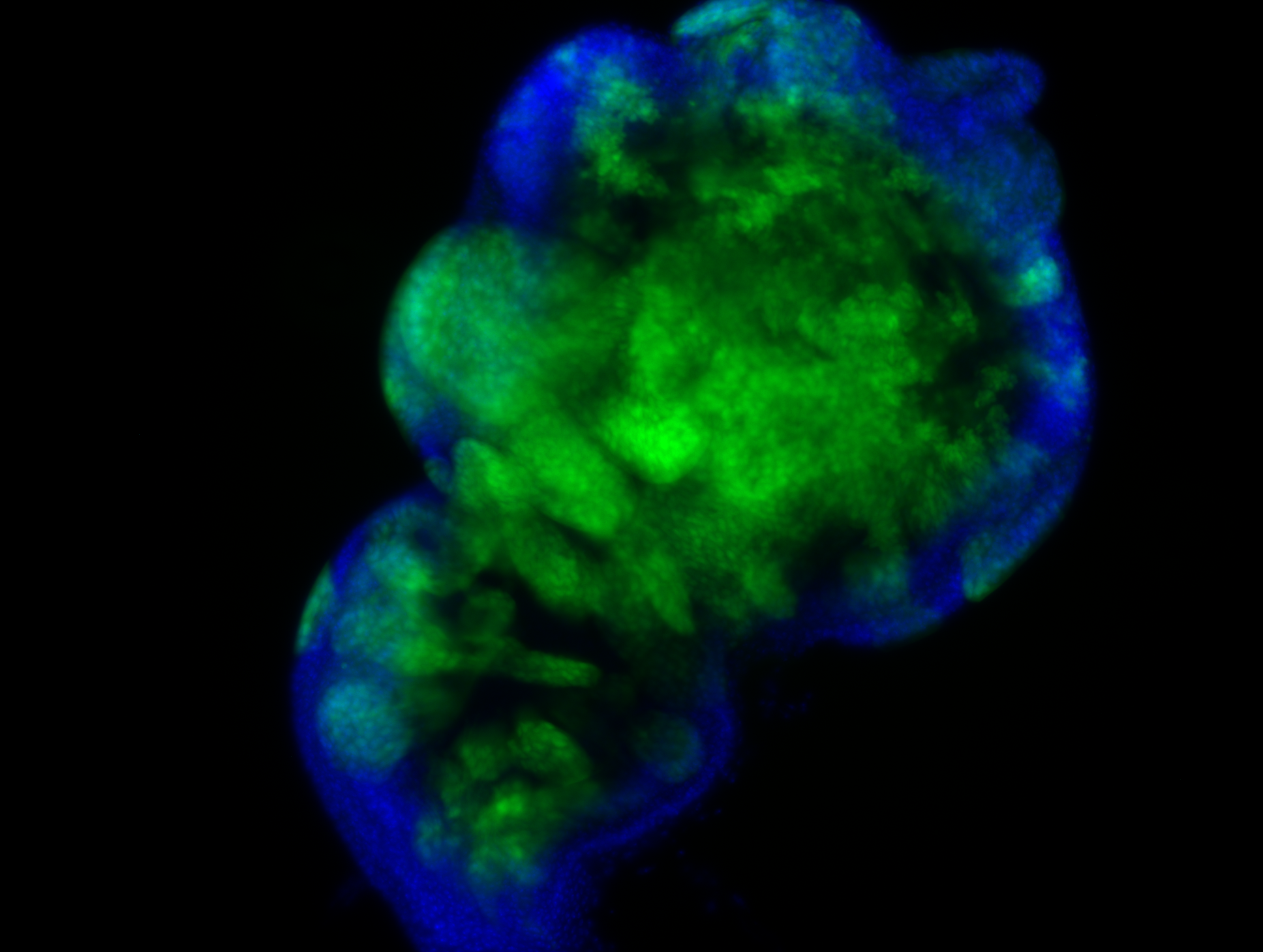

Supplement: Supplementary file 6 — Source data Fig. 2 [file 44318_2025_547_MOESM6_ESM.zip › Figure 2G/9 original image.tif]

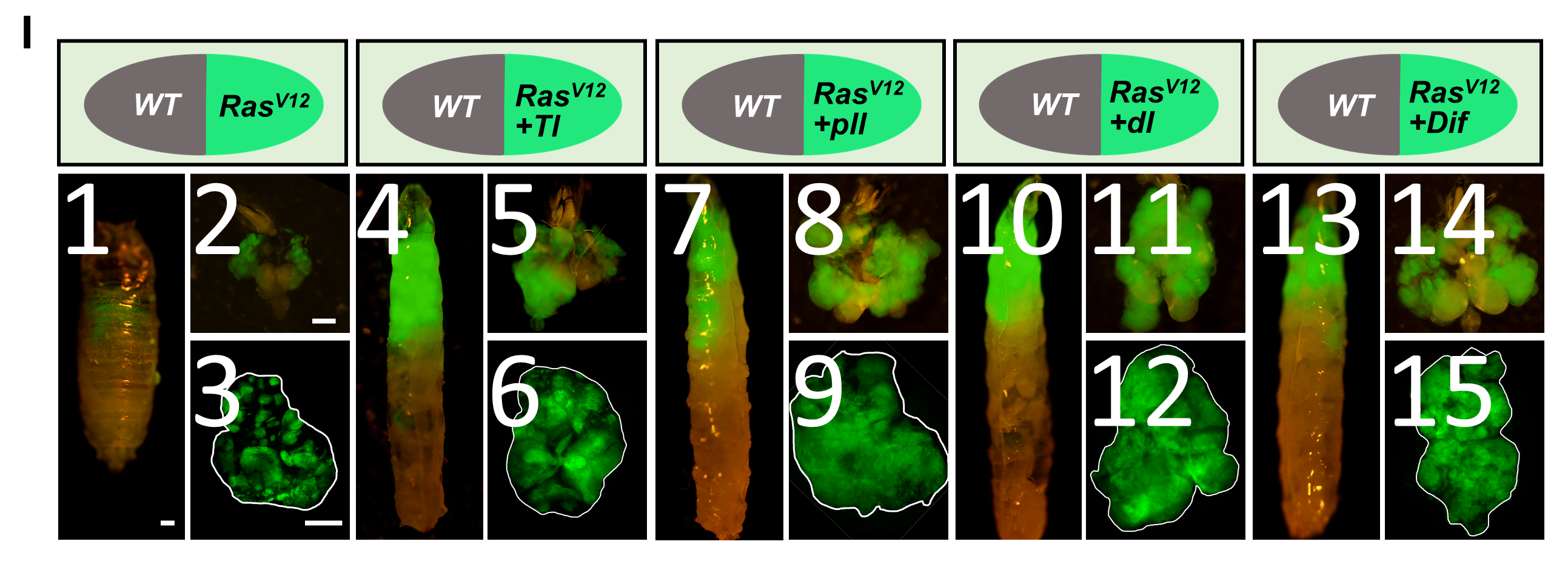

Supplement: Supplementary file 6 — Source data Fig. 2 [file 44318_2025_547_MOESM6_ESM.zip › Figure 2I/0 paper Figure 2I with provided image sequence.tif]

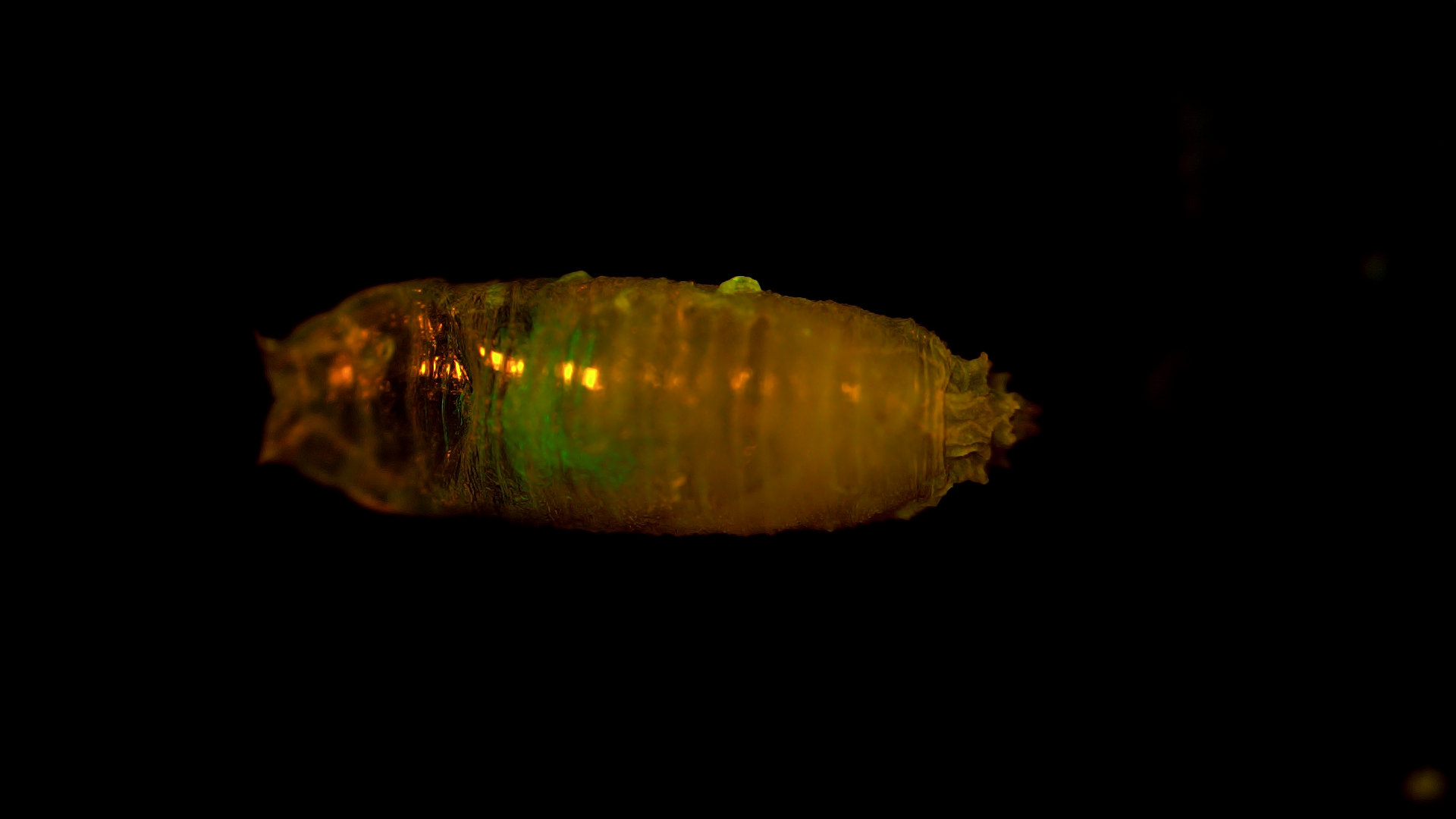

Supplement: Supplementary file 6 — Source data Fig. 2 [file 44318_2025_547_MOESM6_ESM.zip › Figure 2I/1 original image.tif]

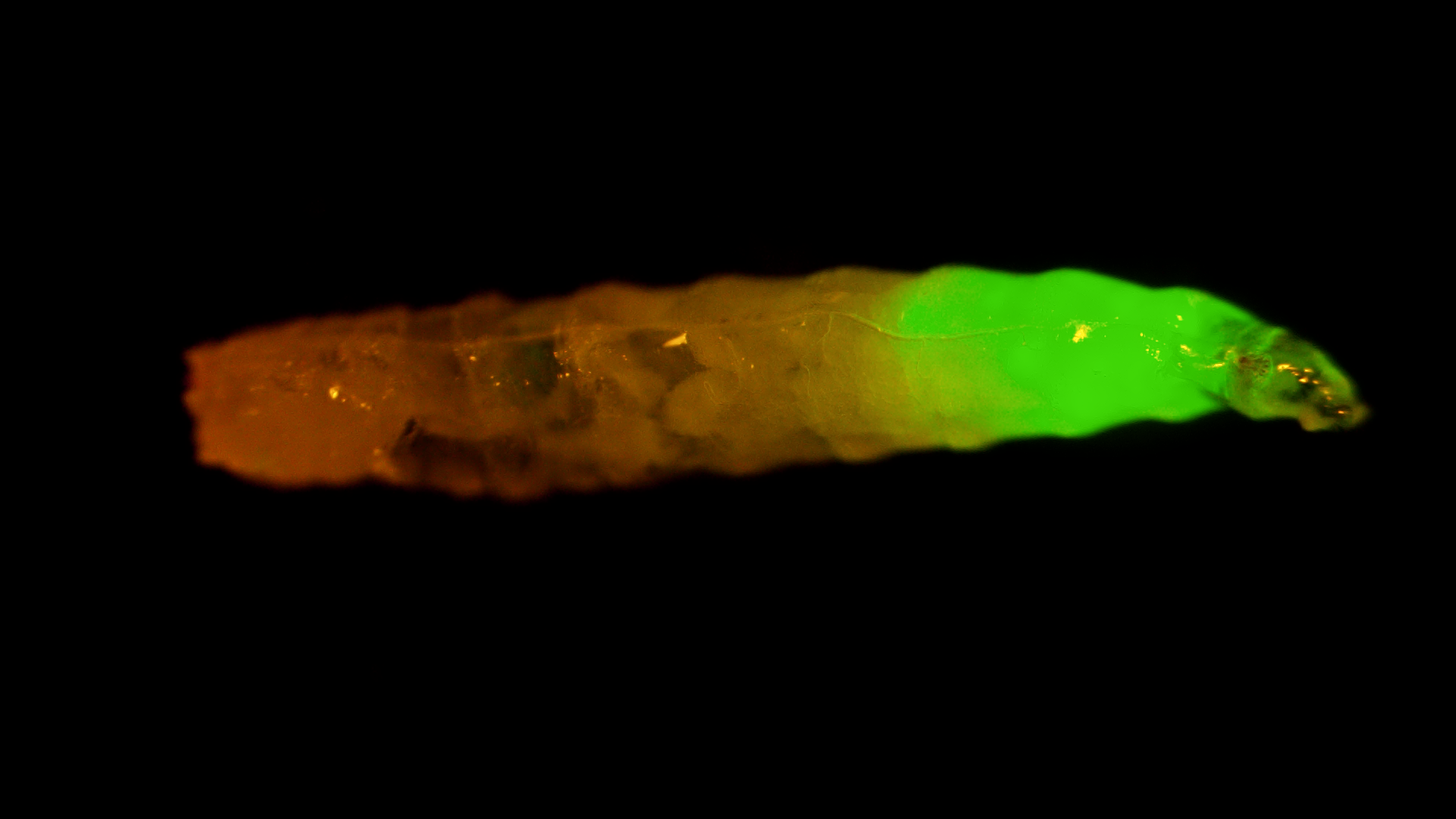

Supplement: Supplementary file 6 — Source data Fig. 2 [file 44318_2025_547_MOESM6_ESM.zip › Figure 2I/10 original image.tif]

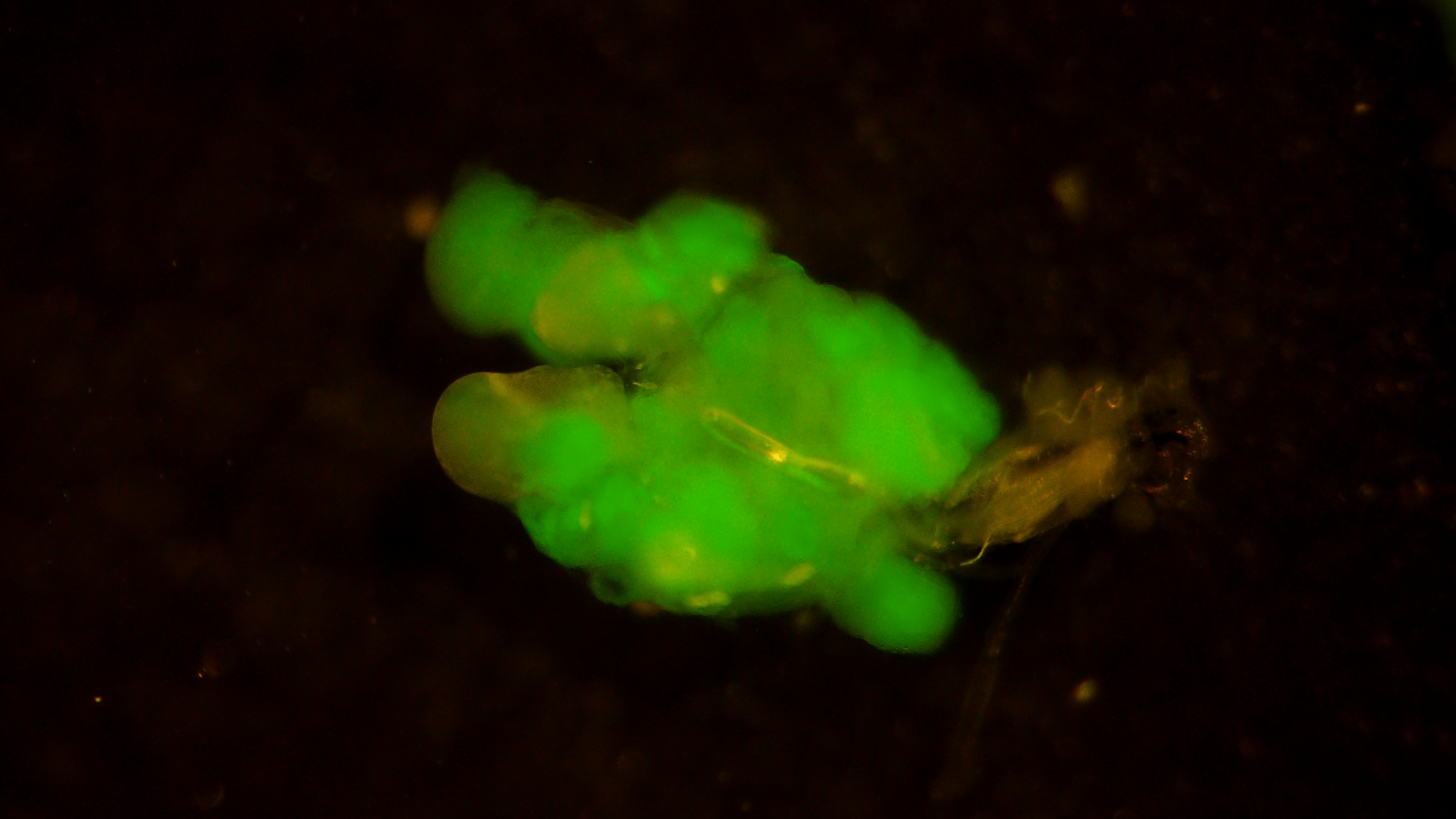

Supplement: Supplementary file 6 — Source data Fig. 2 [file 44318_2025_547_MOESM6_ESM.zip › Figure 2I/11 original image.tif]

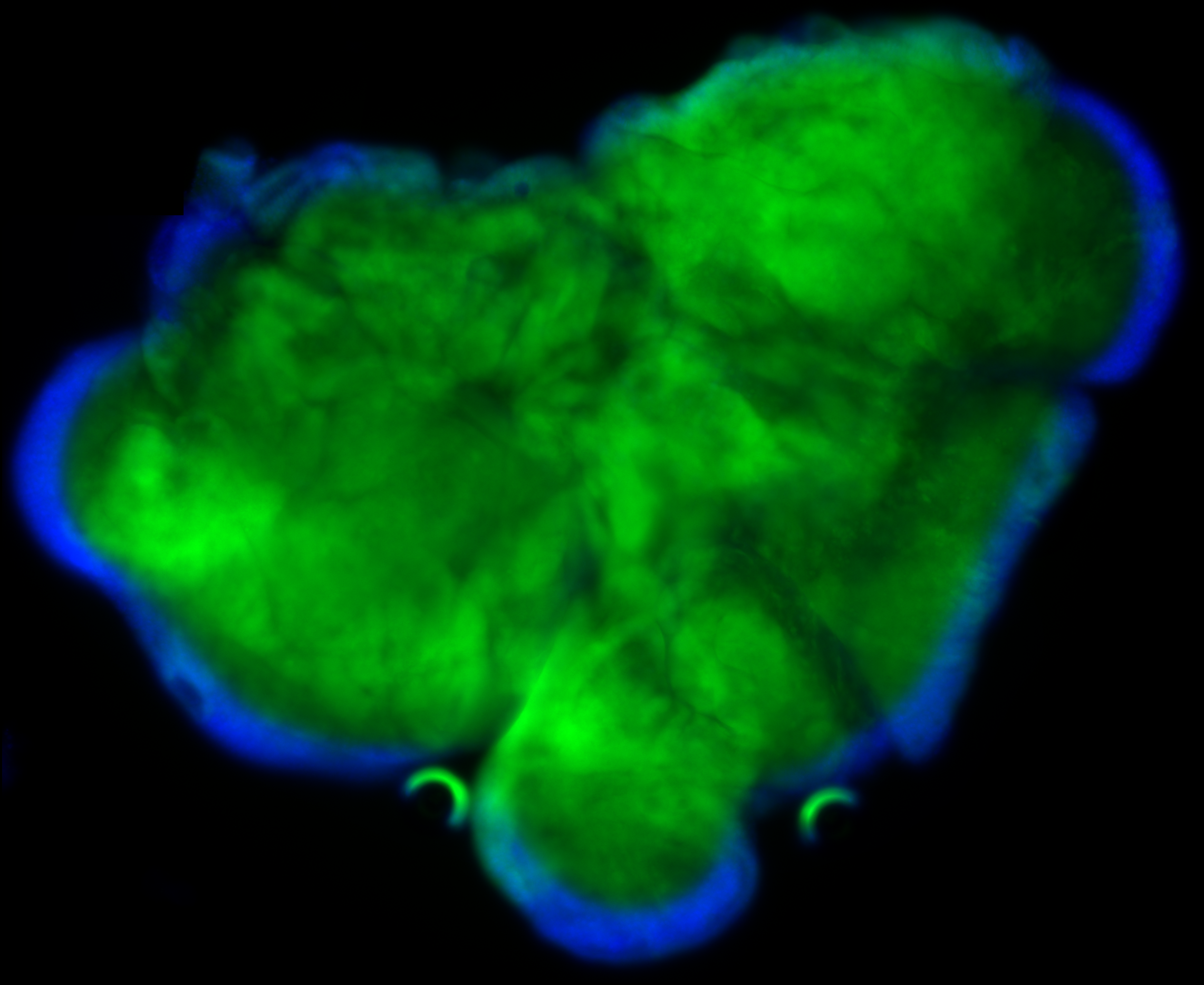

Supplement: Supplementary file 6 — Source data Fig. 2 [file 44318_2025_547_MOESM6_ESM.zip › Figure 2I/12 original image.tif]

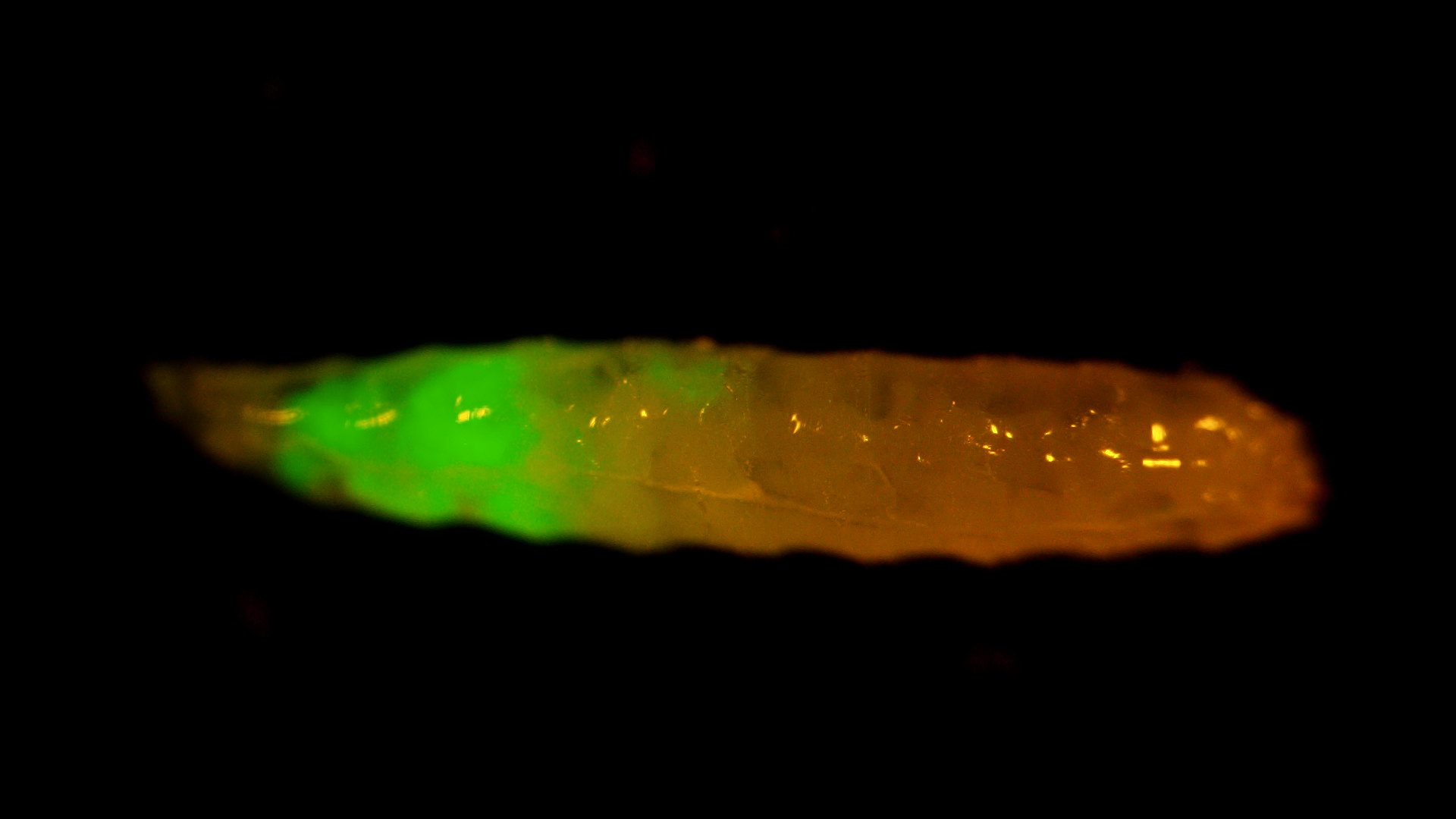

Supplement: Supplementary file 6 — Source data Fig. 2 [file 44318_2025_547_MOESM6_ESM.zip › Figure 2I/13 original image.tif]

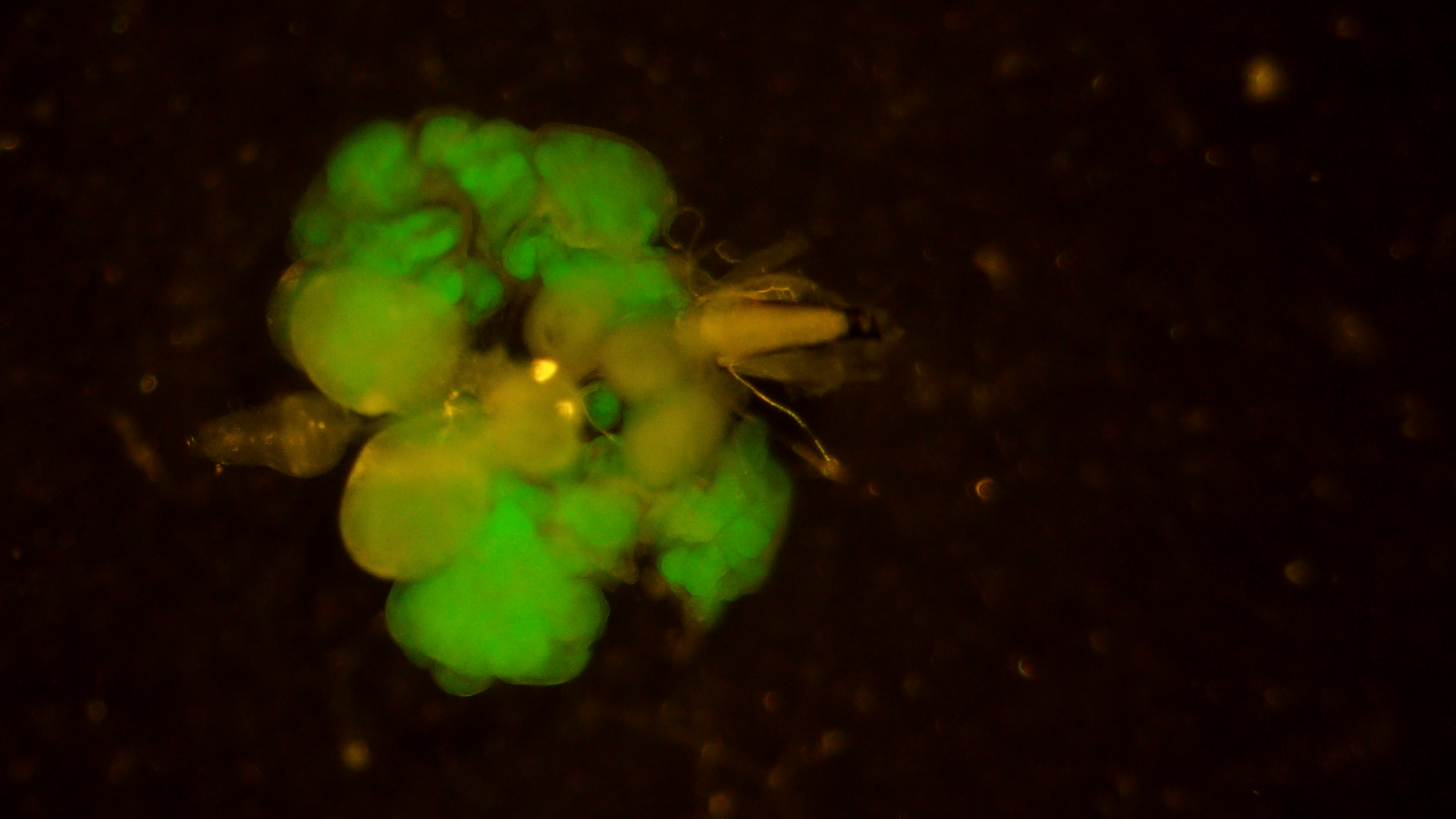

Supplement: Supplementary file 6 — Source data Fig. 2 [file 44318_2025_547_MOESM6_ESM.zip › Figure 2I/14 original image.tif]

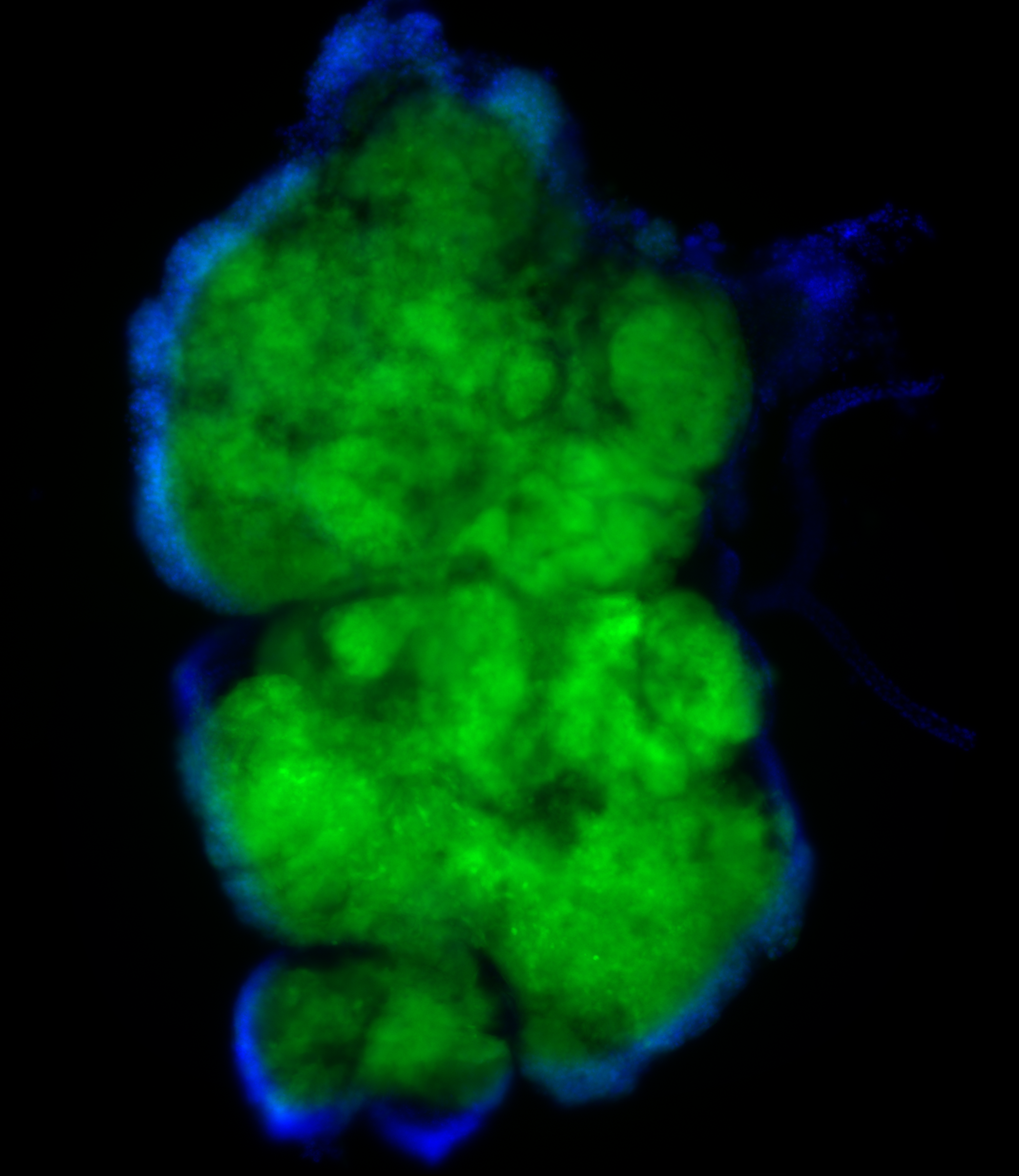

Supplement: Supplementary file 6 — Source data Fig. 2 [file 44318_2025_547_MOESM6_ESM.zip › Figure 2I/15 original image.tif]

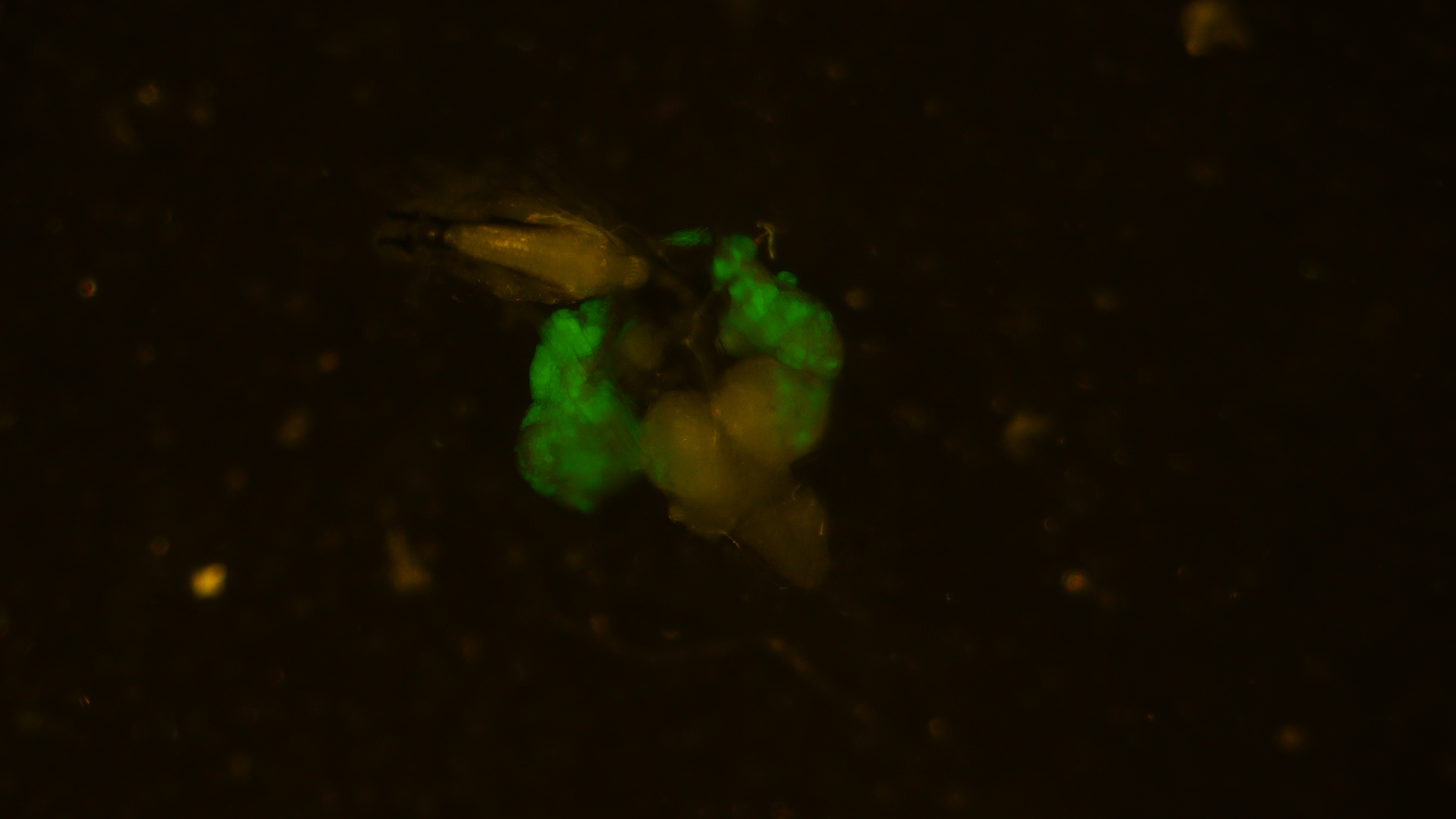

Supplement: Supplementary file 6 — Source data Fig. 2 [file 44318_2025_547_MOESM6_ESM.zip › Figure 2I/2 original image.tif]

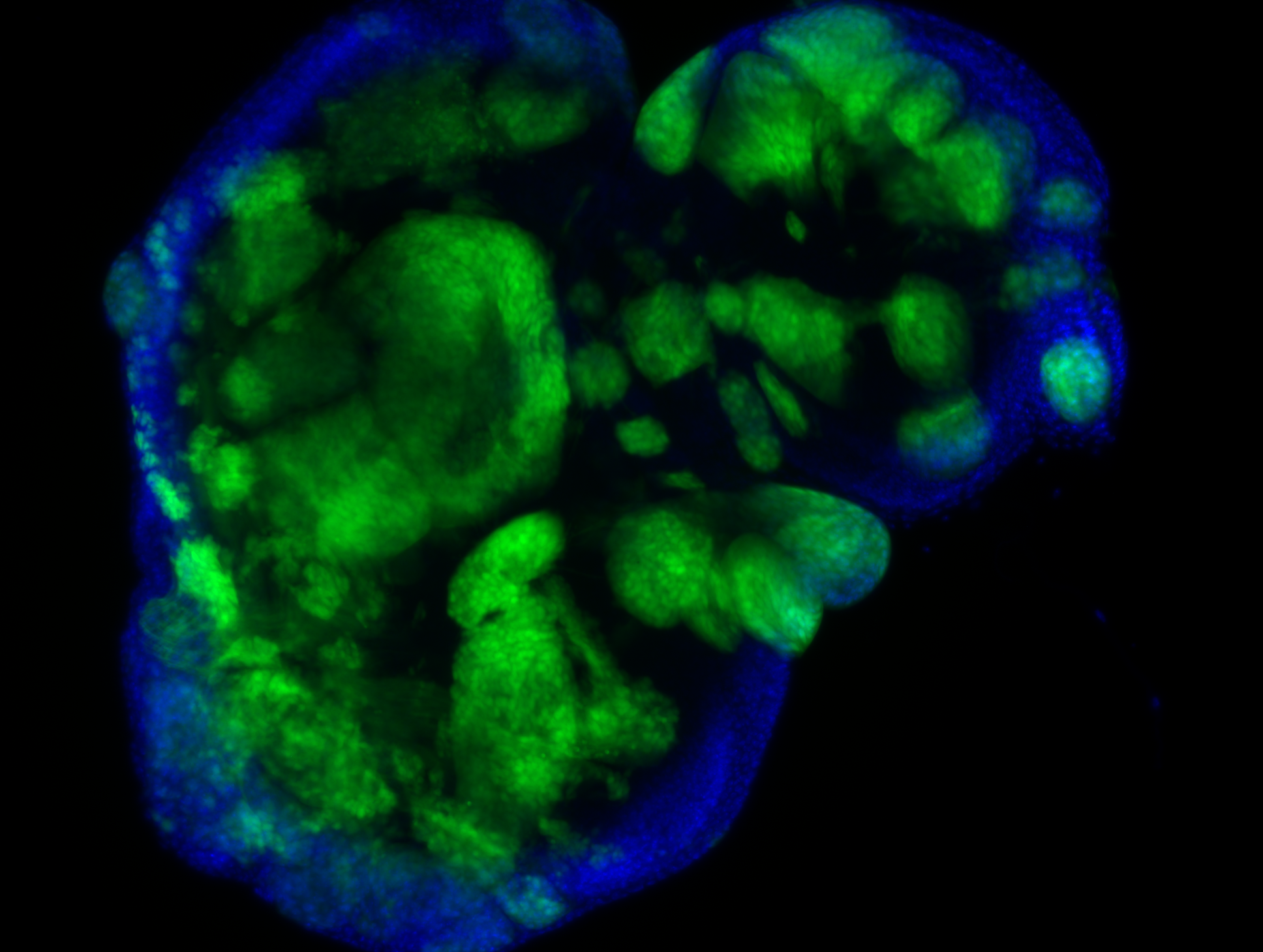

Supplement: Supplementary file 6 — Source data Fig. 2 [file 44318_2025_547_MOESM6_ESM.zip › Figure 2I/3 original image.tif]

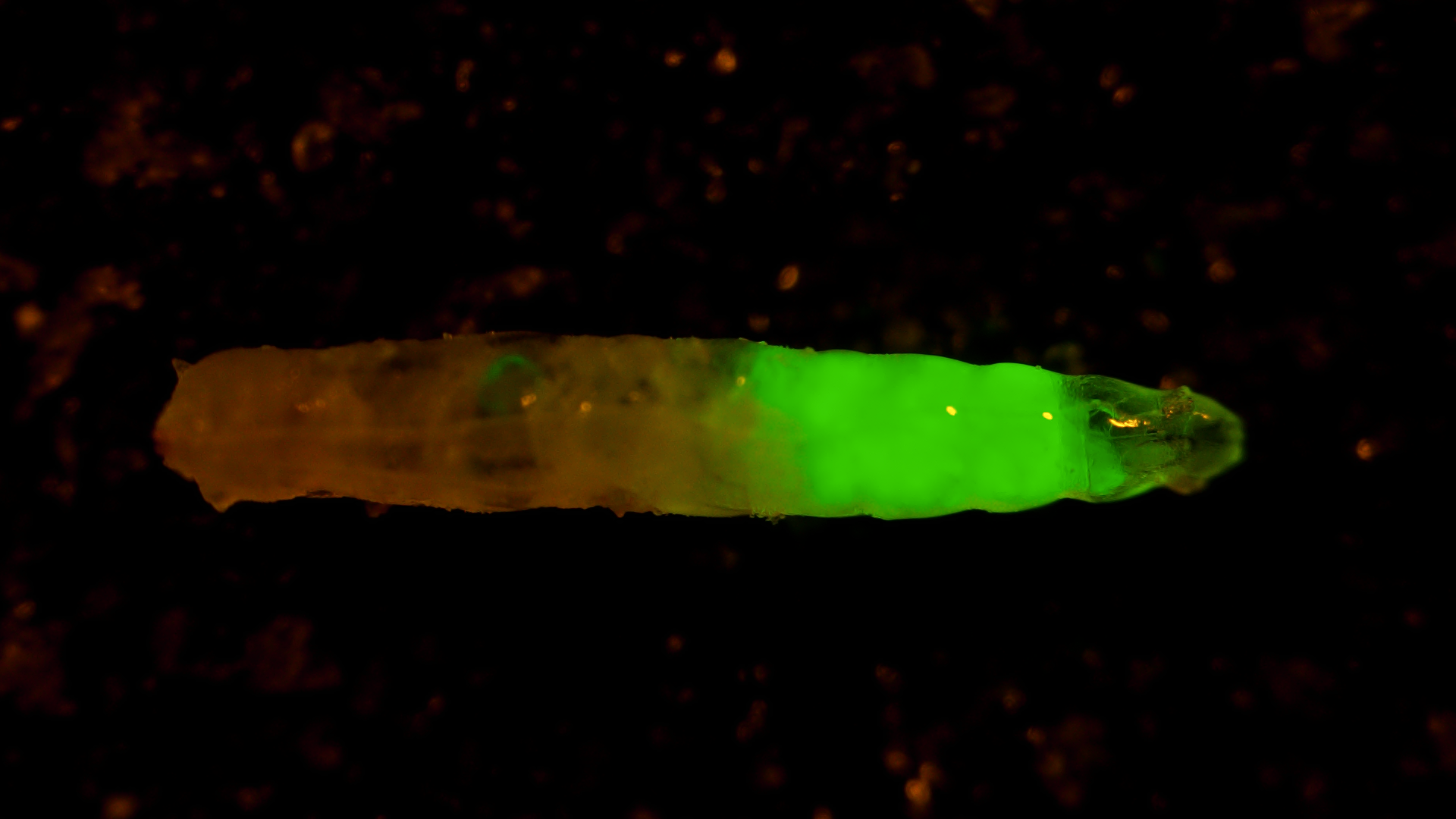

Supplement: Supplementary file 6 — Source data Fig. 2 [file 44318_2025_547_MOESM6_ESM.zip › Figure 2I/4 original image.tif]

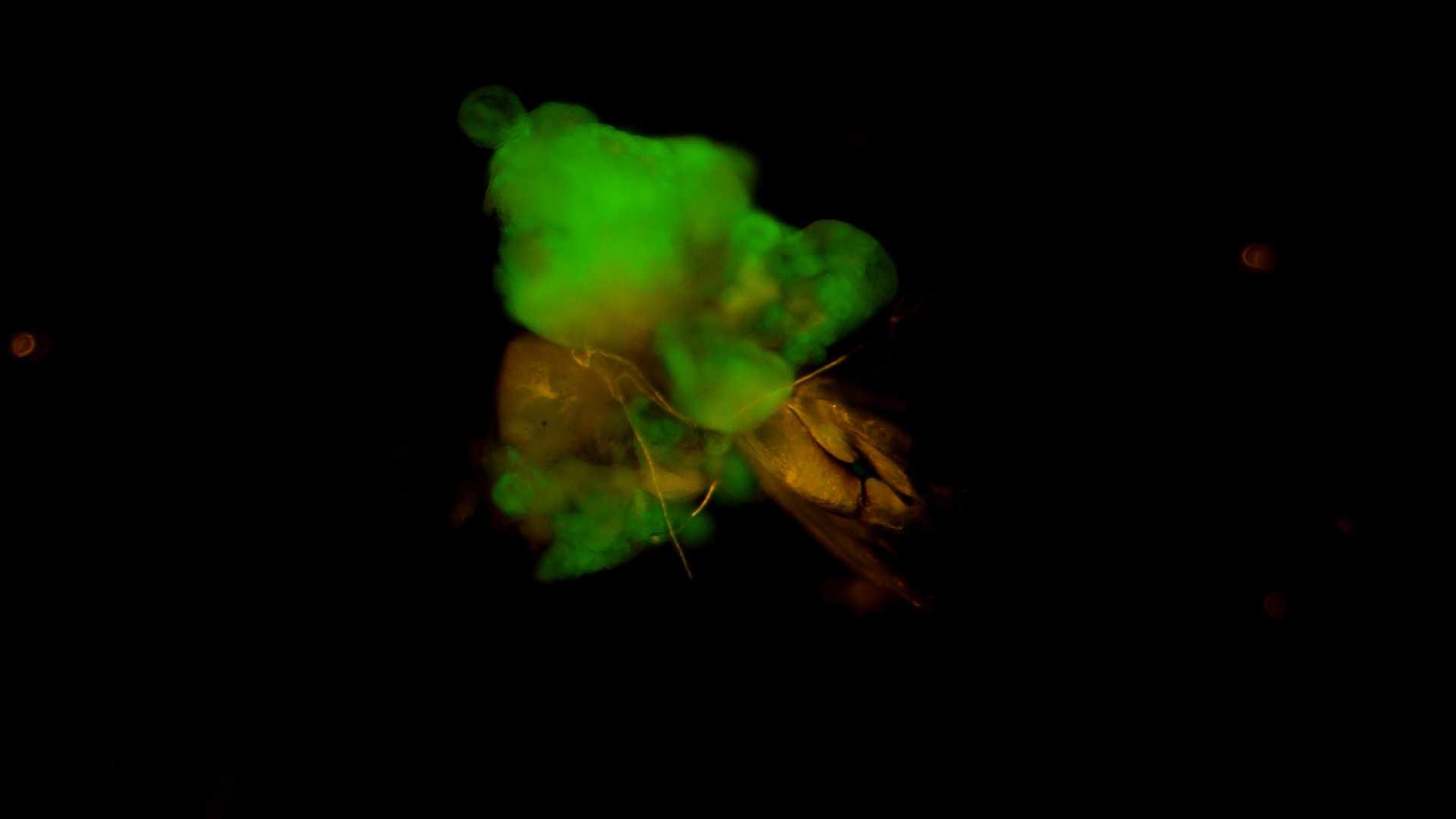

Supplement: Supplementary file 6 — Source data Fig. 2 [file 44318_2025_547_MOESM6_ESM.zip › Figure 2I/5 original image.tif]

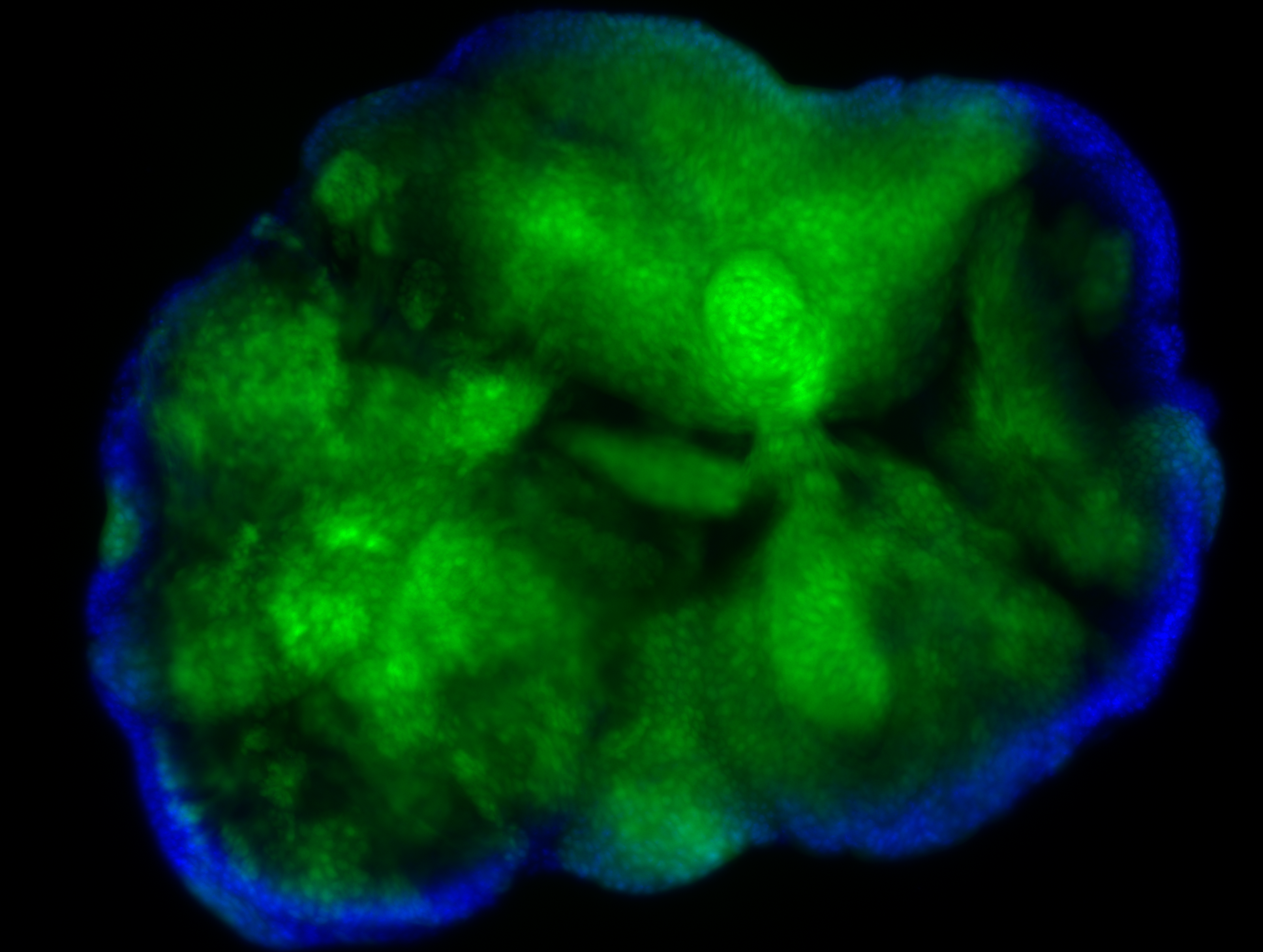

Supplement: Supplementary file 6 — Source data Fig. 2 [file 44318_2025_547_MOESM6_ESM.zip › Figure 2I/6 original image.tif]

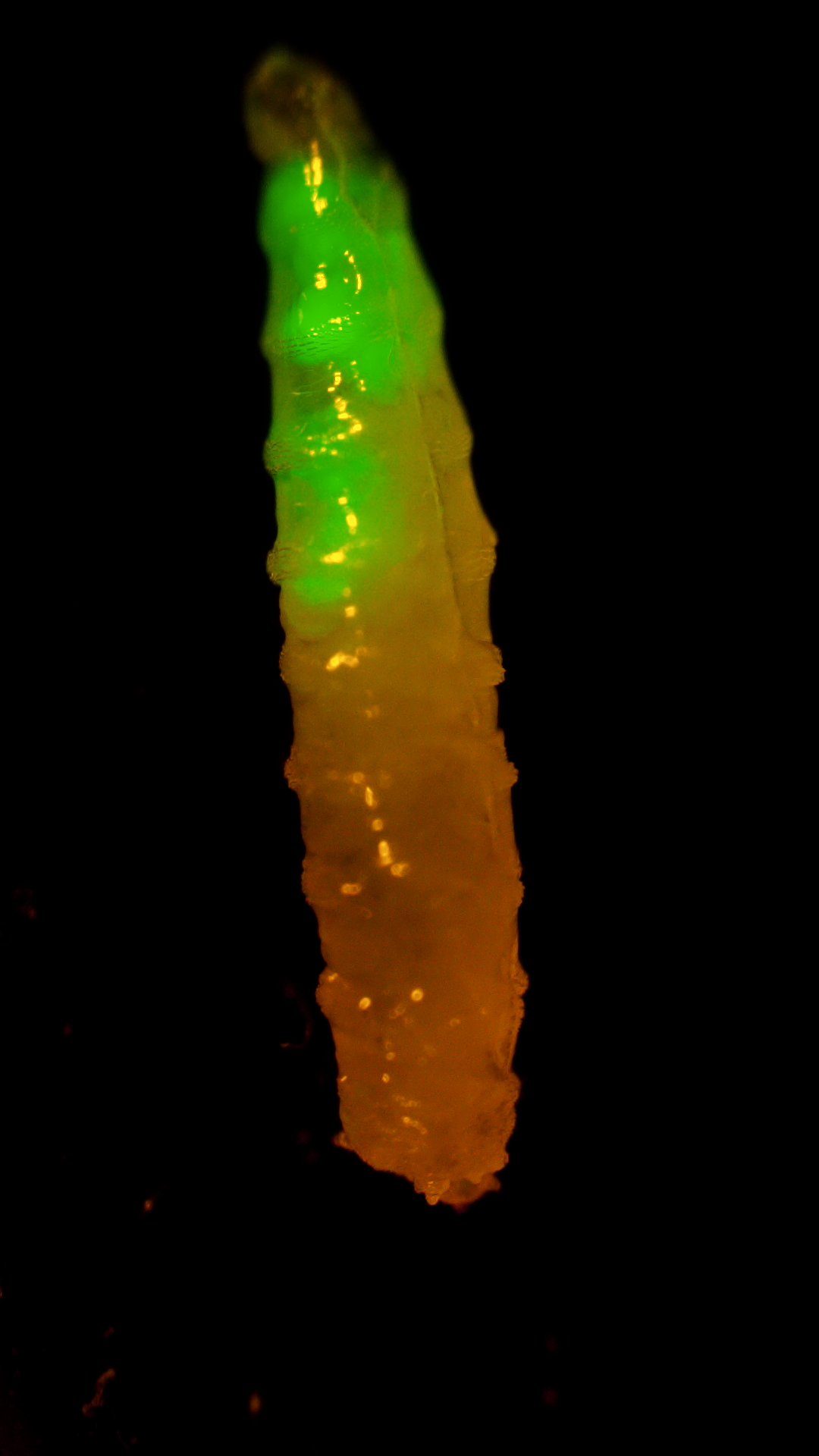

Supplement: Supplementary file 6 — Source data Fig. 2 [file 44318_2025_547_MOESM6_ESM.zip › Figure 2I/7 original image.tif]

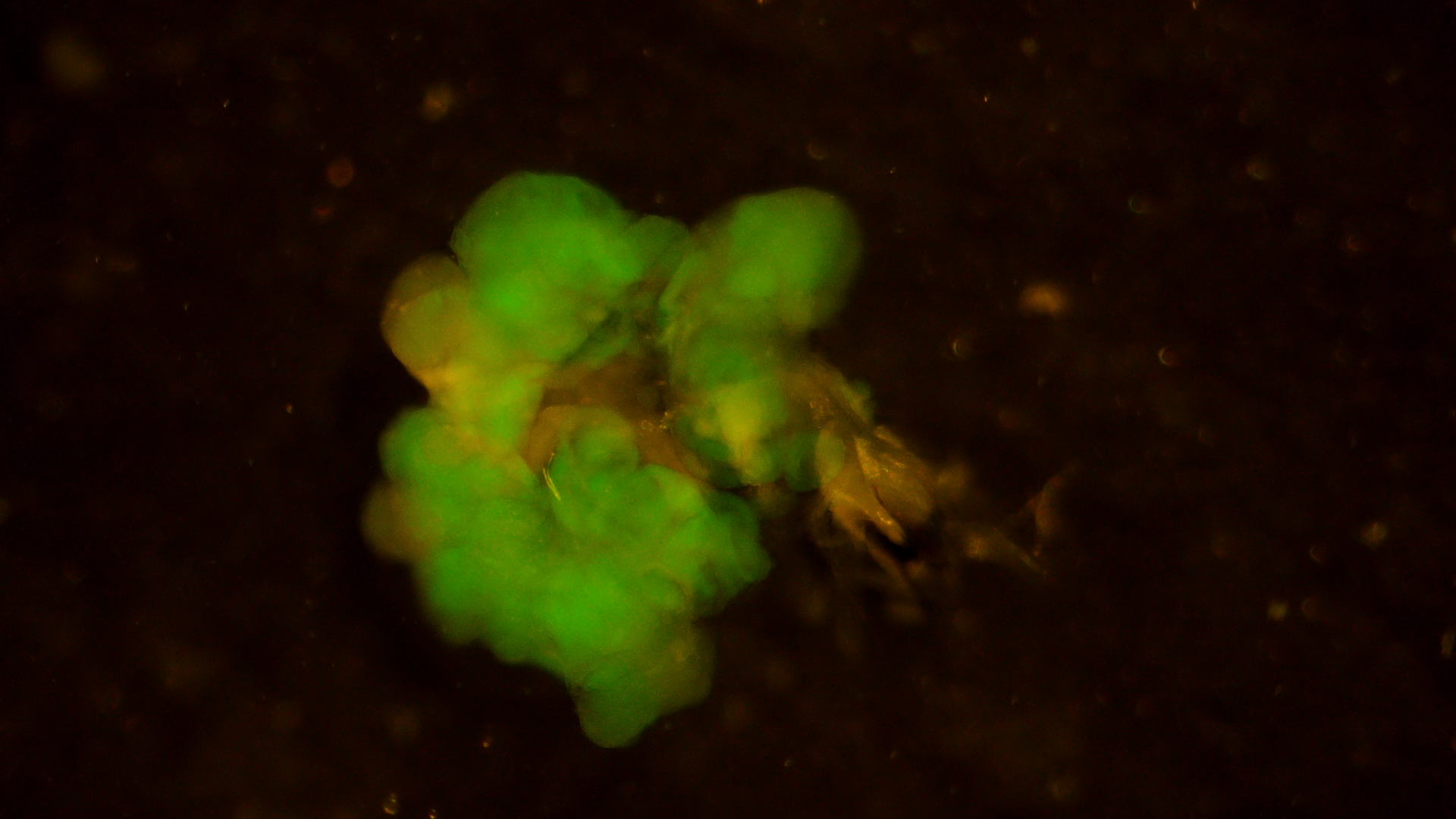

Supplement: Supplementary file 6 — Source data Fig. 2 [file 44318_2025_547_MOESM6_ESM.zip › Figure 2I/8 original image.tif]

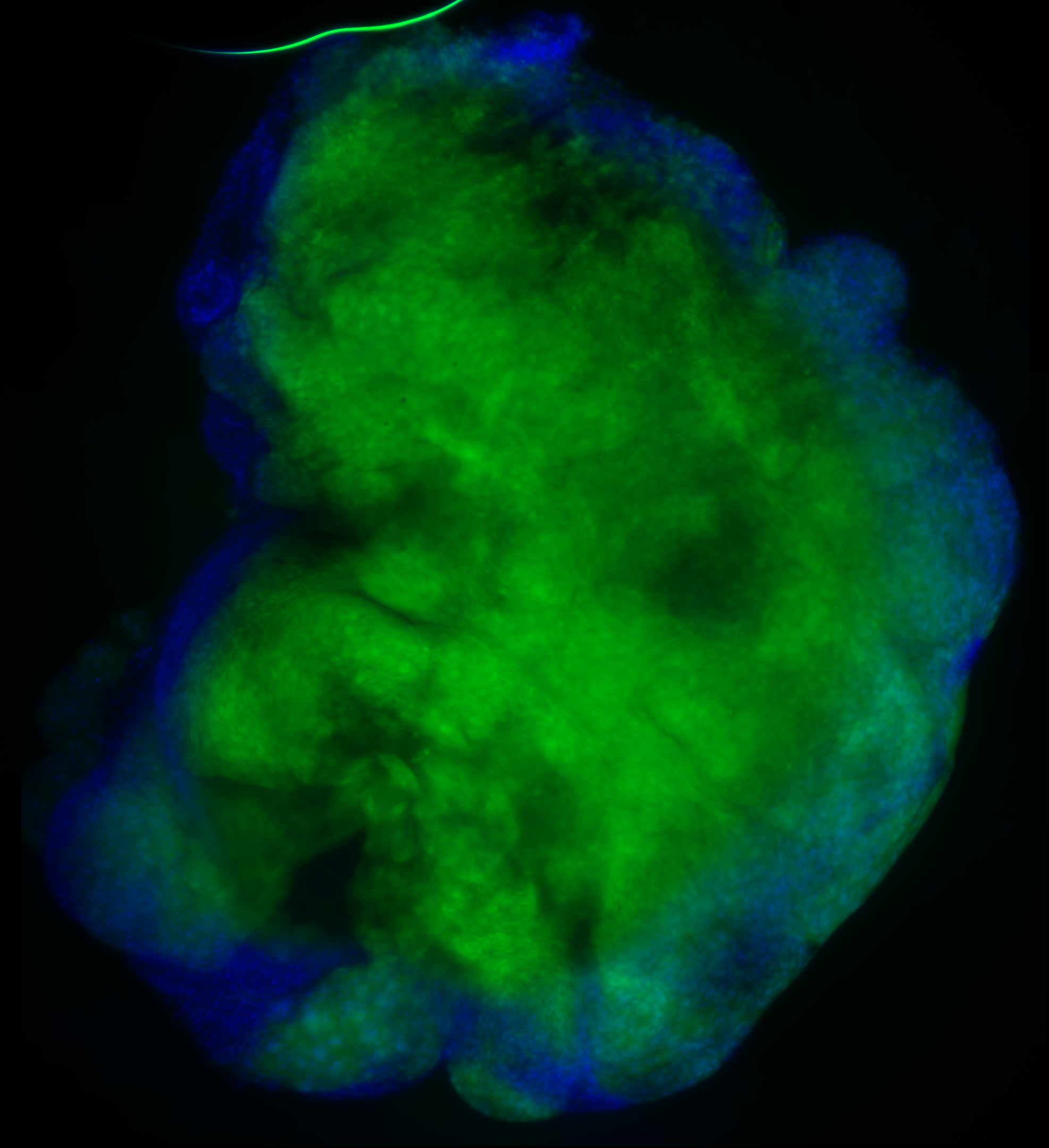

Supplement: Supplementary file 6 — Source data Fig. 2 [file 44318_2025_547_MOESM6_ESM.zip › Figure 2I/9 original image.tif]

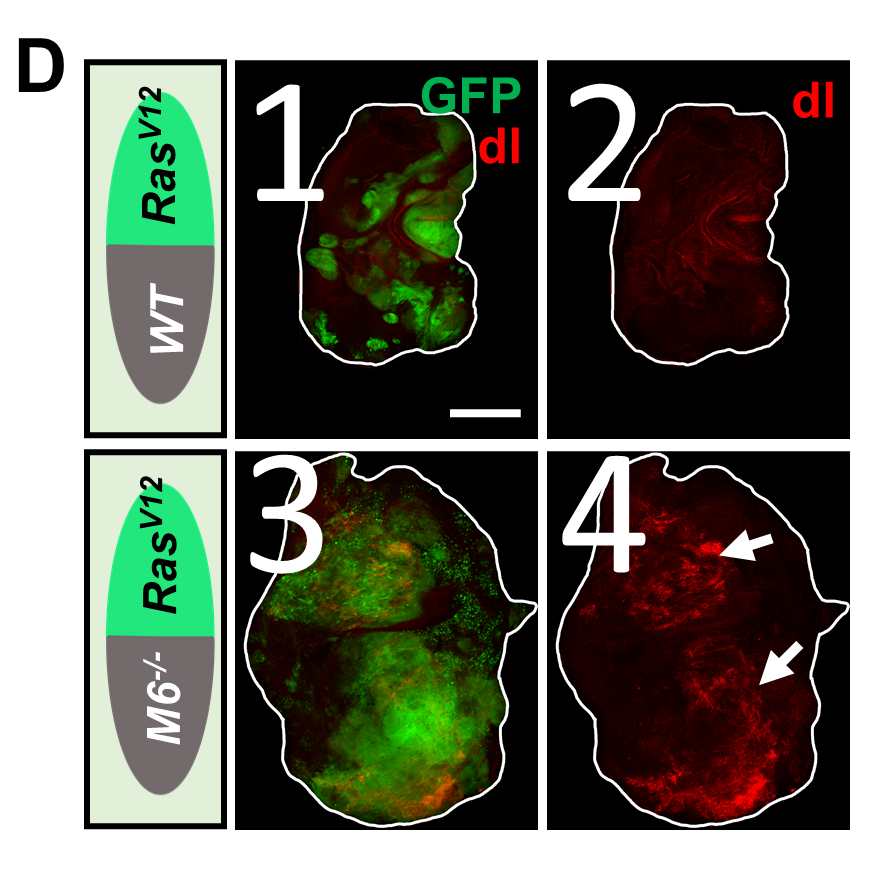

Supplement: Supplementary file 6 — Source data Fig. 2 [file 44318_2025_547_MOESM6_ESM.zip › Figure 2D/0 paper Figure 2D with provided image sequence.tif]

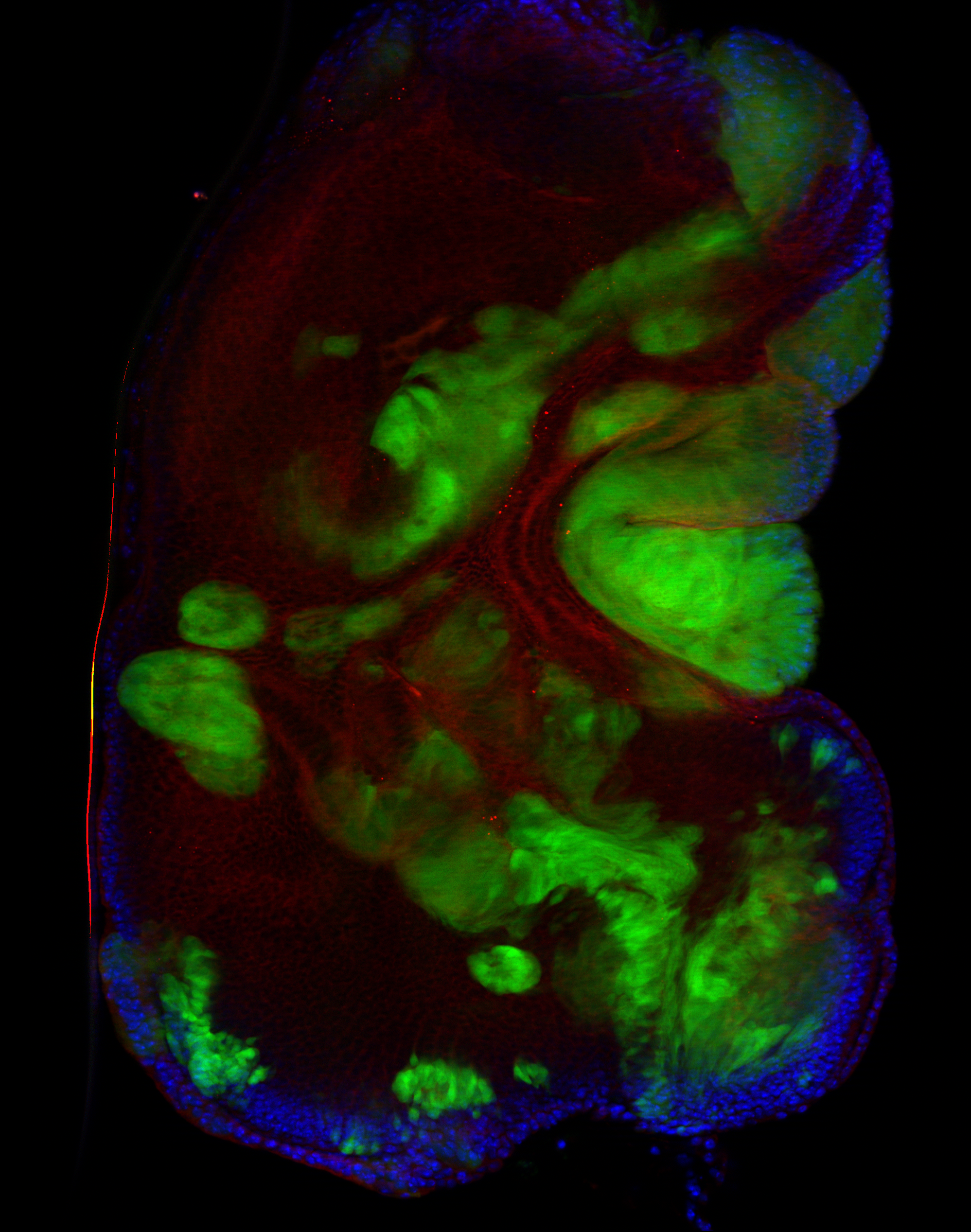

Supplement: Supplementary file 6 — Source data Fig. 2 [file 44318_2025_547_MOESM6_ESM.zip › Figure 2D/1 original image.tif]

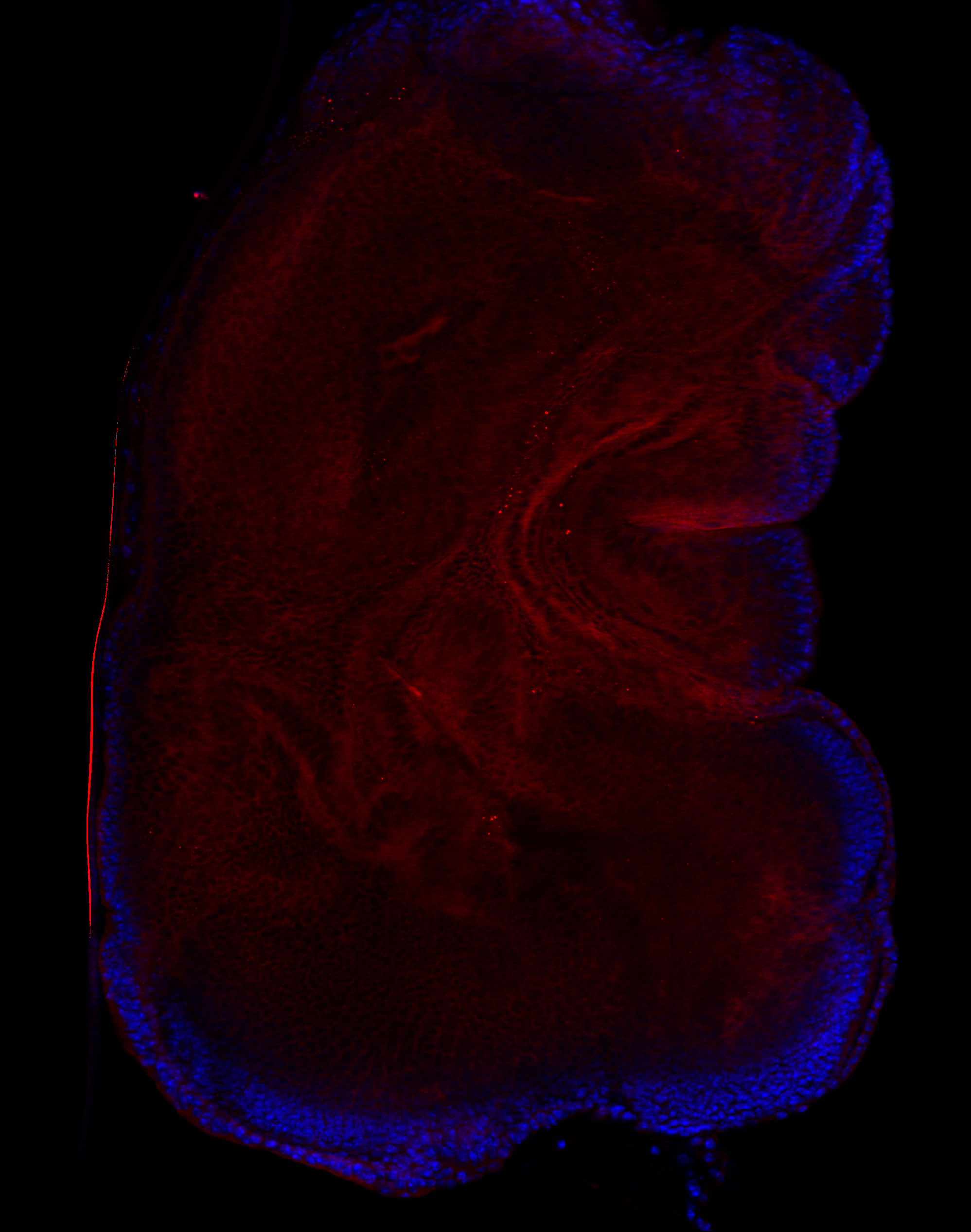

Supplement: Supplementary file 6 — Source data Fig. 2 [file 44318_2025_547_MOESM6_ESM.zip › Figure 2D/2 original image.tif]

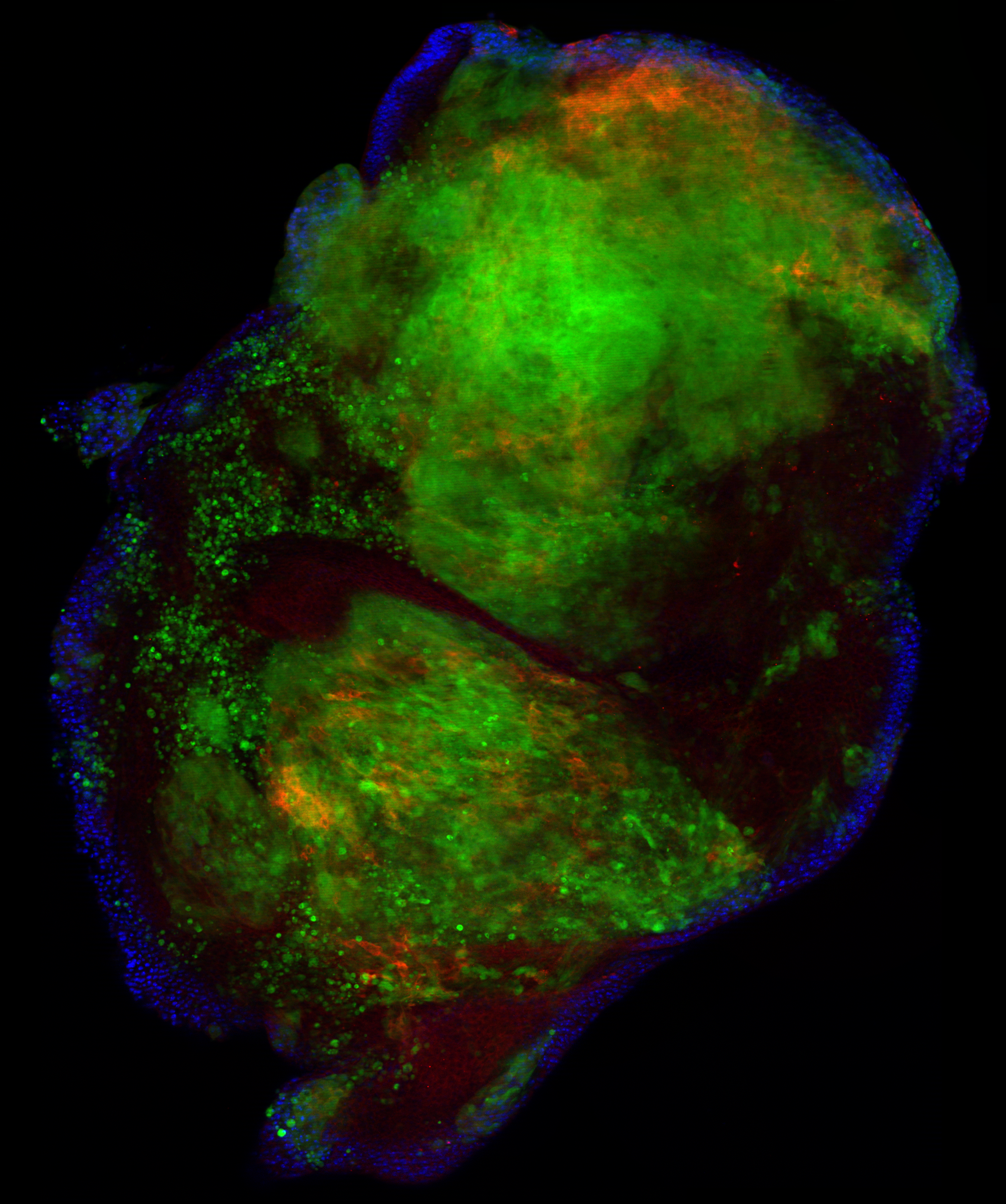

Supplement: Supplementary file 6 — Source data Fig. 2 [file 44318_2025_547_MOESM6_ESM.zip › Figure 2D/3 original image.tif]

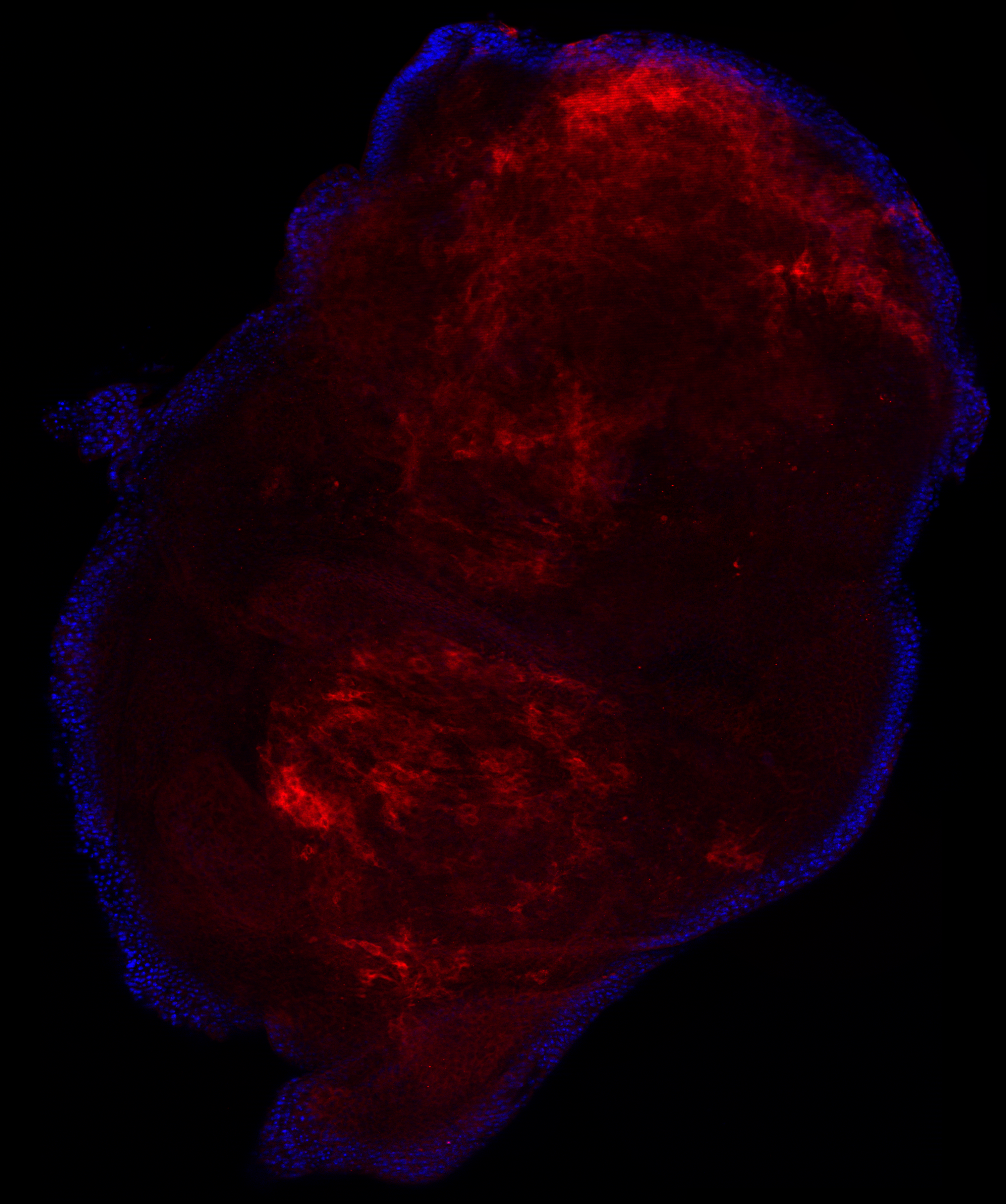

Supplement: Supplementary file 6 — Source data Fig. 2 [file 44318_2025_547_MOESM6_ESM.zip › Figure 2D/4 original image.tif]

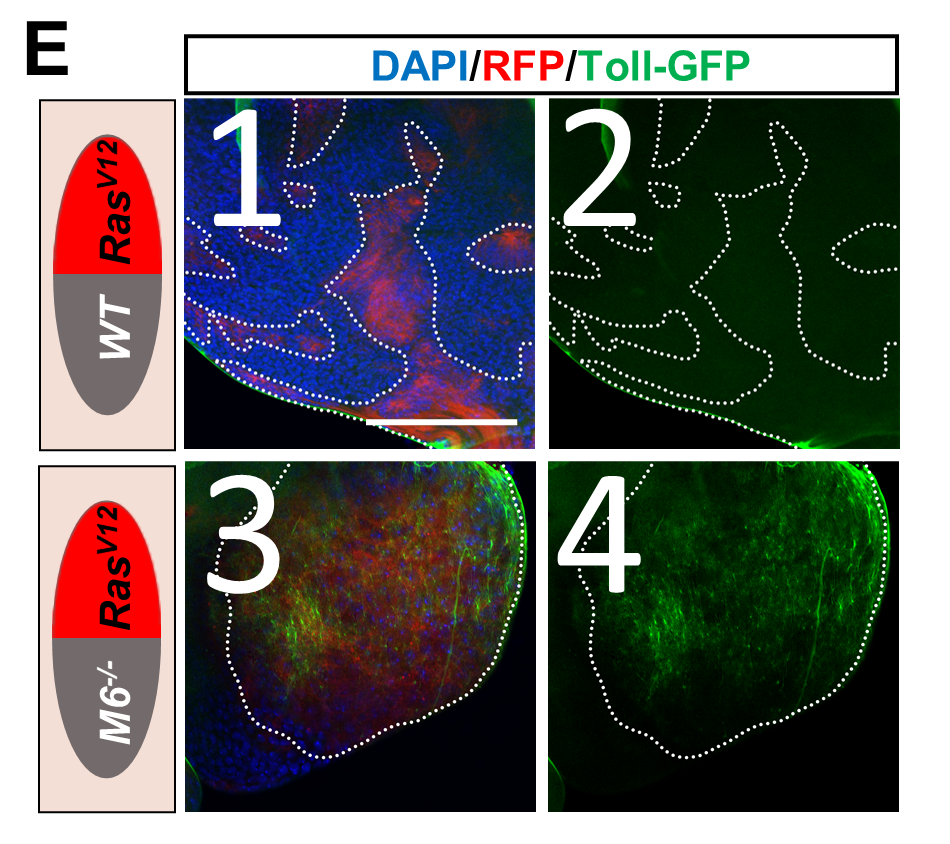

Supplement: Supplementary file 6 — Source data Fig. 2 [file 44318_2025_547_MOESM6_ESM.zip › Figure 2E/0 paper Figure 2E with provided image sequence.tif]

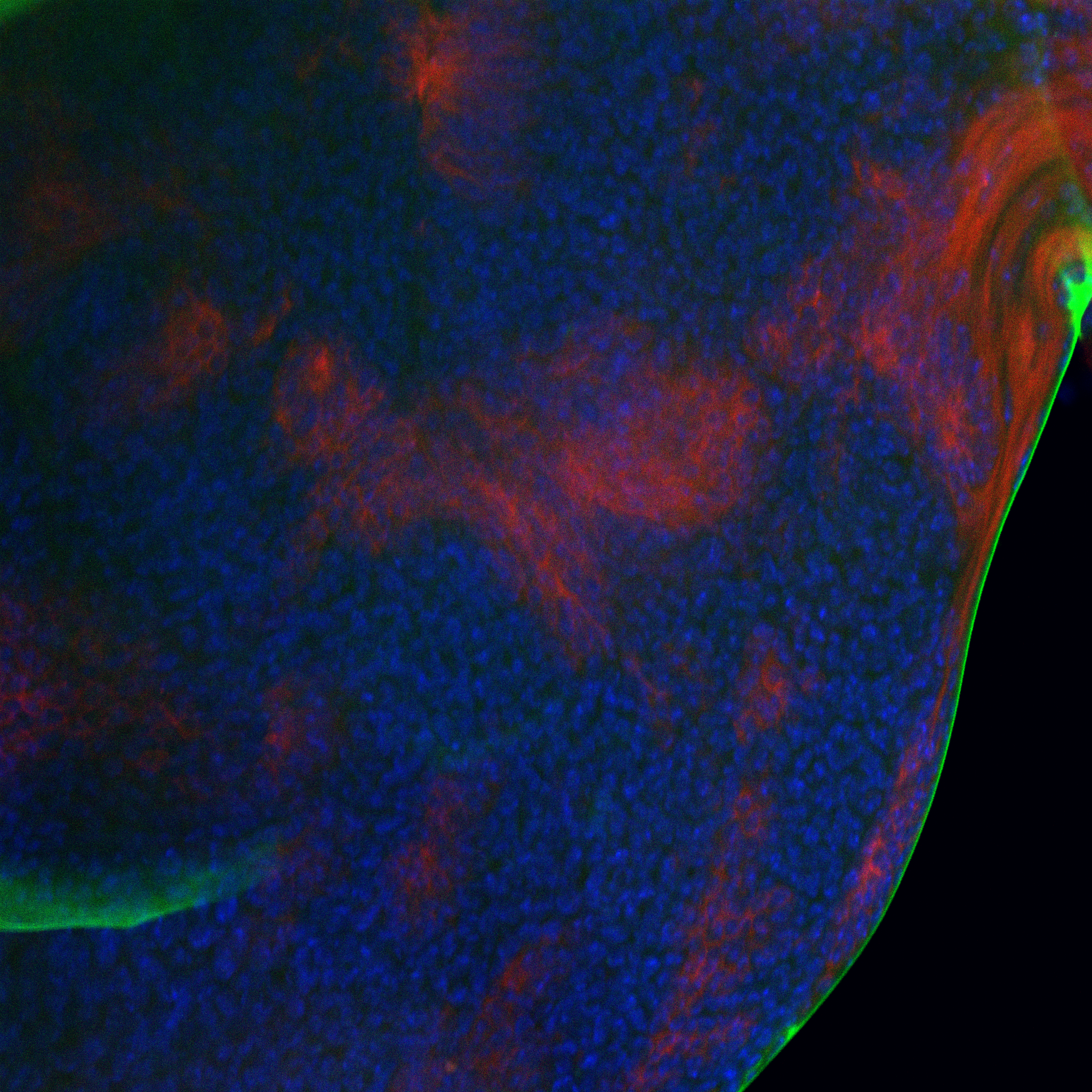

Supplement: Supplementary file 6 — Source data Fig. 2 [file 44318_2025_547_MOESM6_ESM.zip › Figure 2E/1 original image.tif]

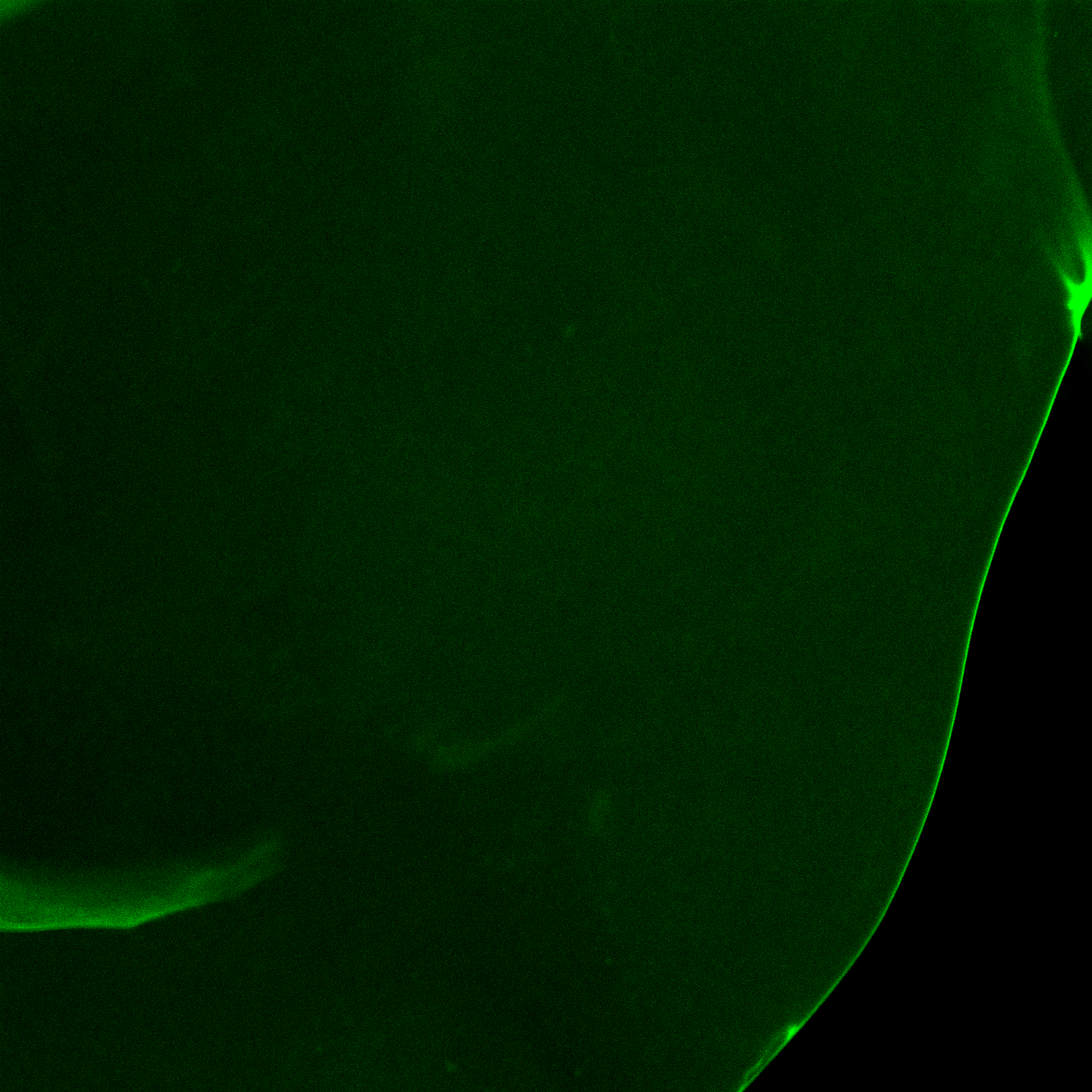

Supplement: Supplementary file 6 — Source data Fig. 2 [file 44318_2025_547_MOESM6_ESM.zip › Figure 2E/2 original image.tif]

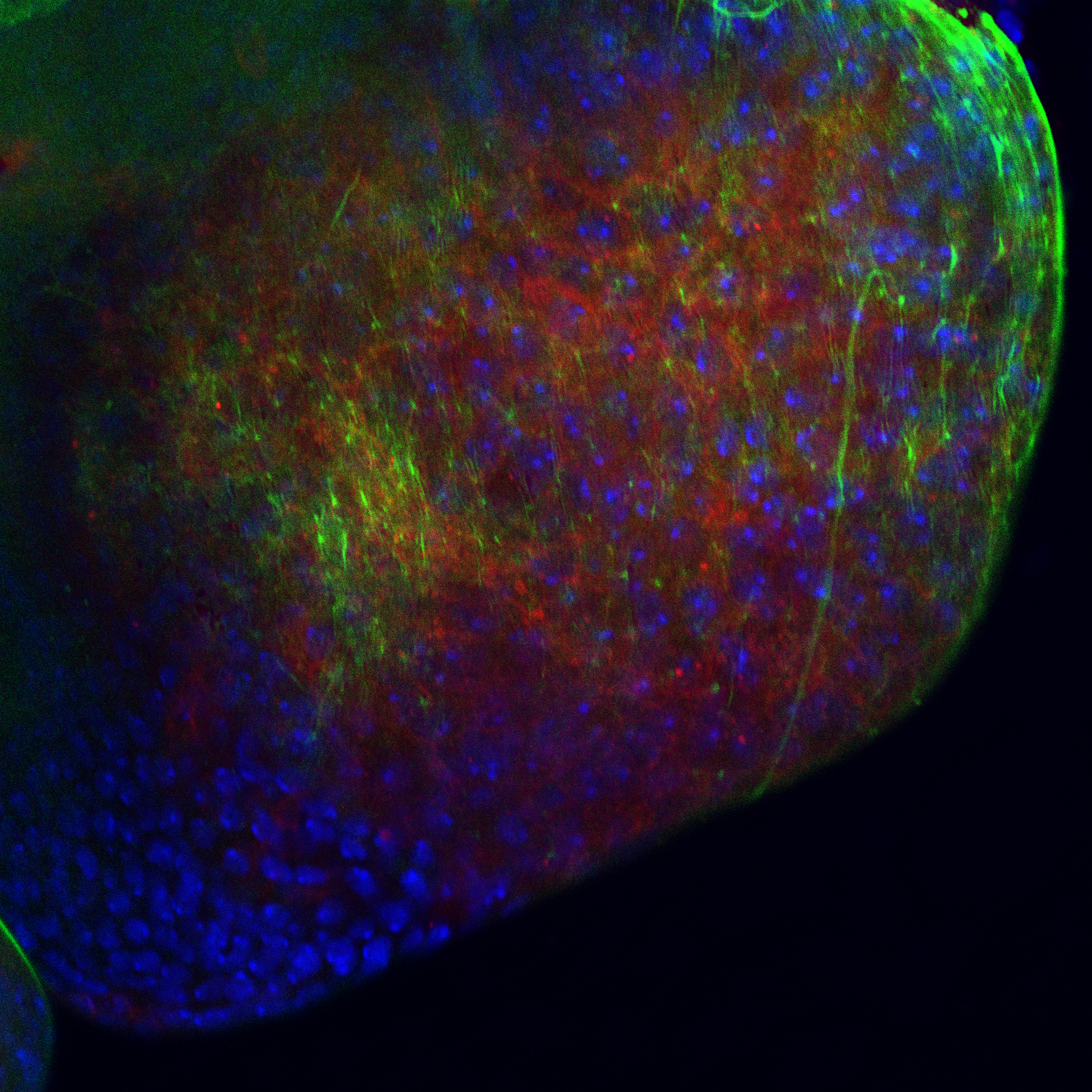

Supplement: Supplementary file 6 — Source data Fig. 2 [file 44318_2025_547_MOESM6_ESM.zip › Figure 2E/3 original image.tif]

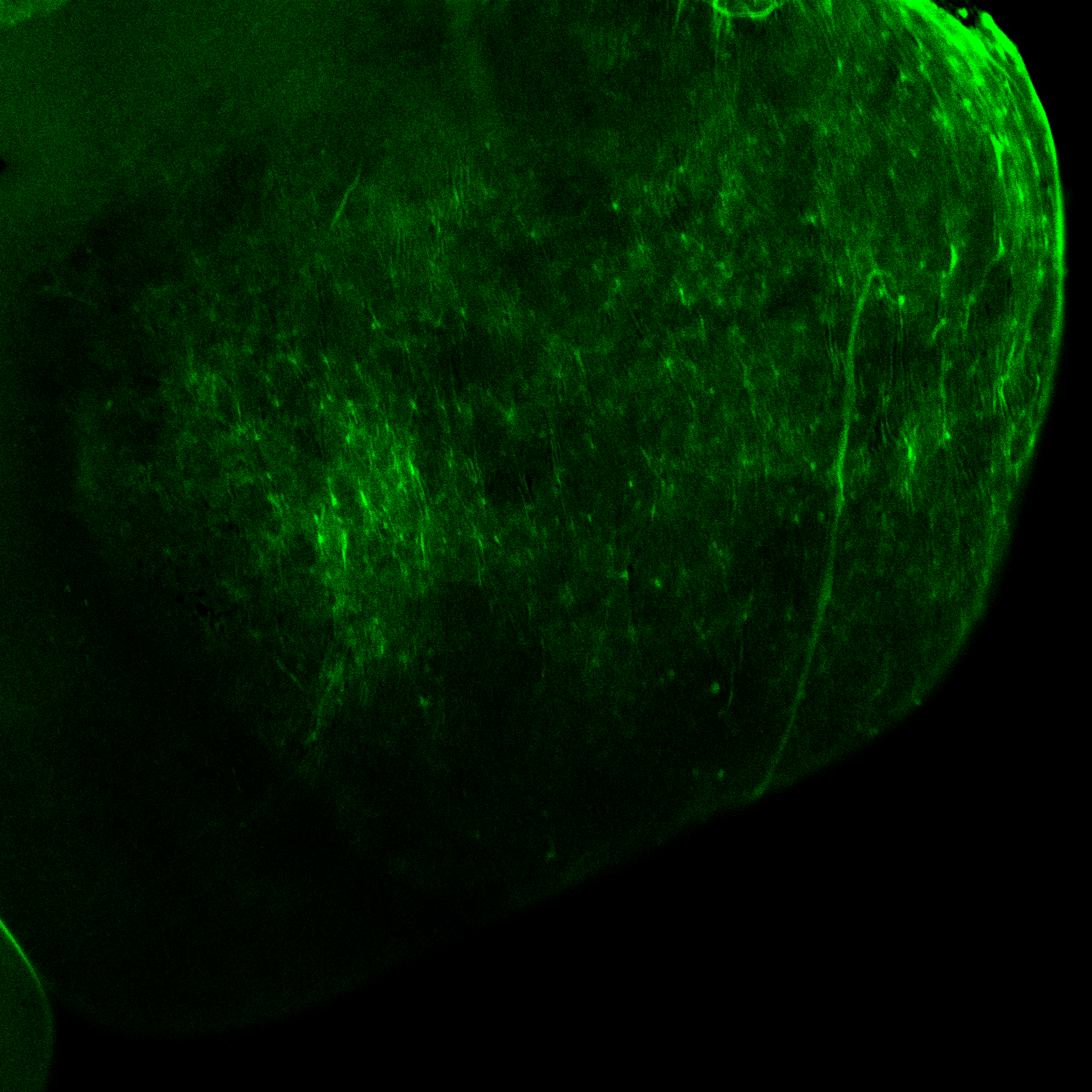

Supplement: Supplementary file 6 — Source data Fig. 2 [file 44318_2025_547_MOESM6_ESM.zip › Figure 2E/4 original image.tif]

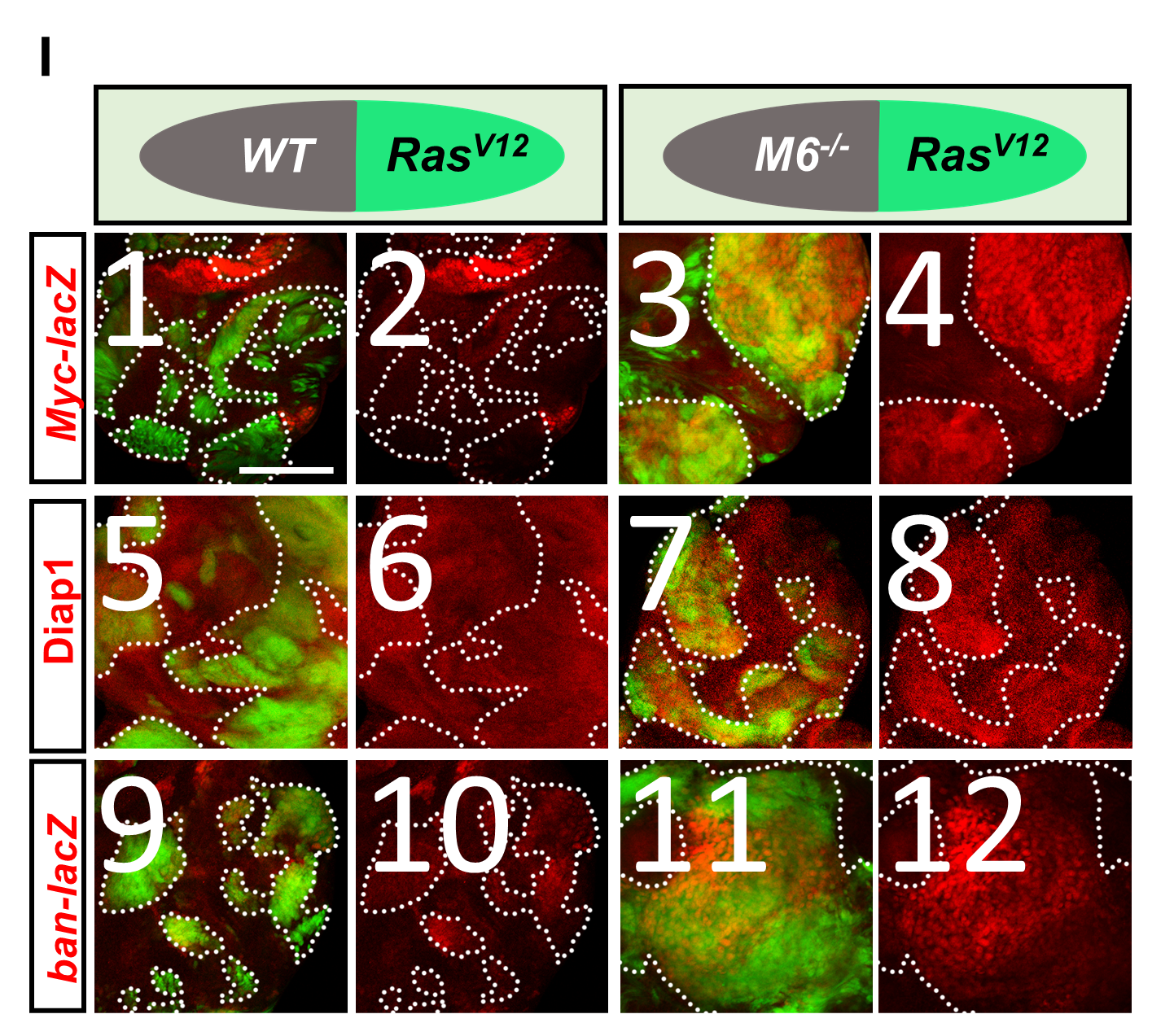

Supplement: Supplementary file 7 — Source data Fig. 3 [file 44318_2025_547_MOESM7_ESM.zip › Figure 3I/0 paper Figure 3I with provided image sequence.tif]

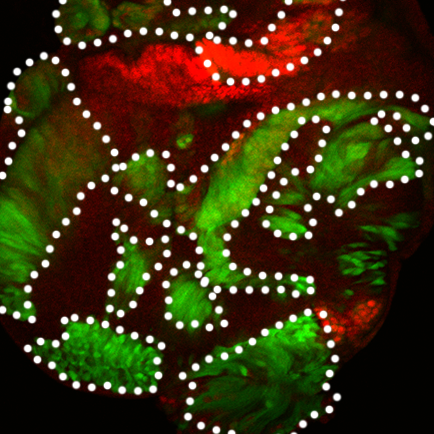

Supplement: Supplementary file 7 — Source data Fig. 3 [file 44318_2025_547_MOESM7_ESM.zip › Figure 3I/1-1 rotated and cut image with border line.tif]

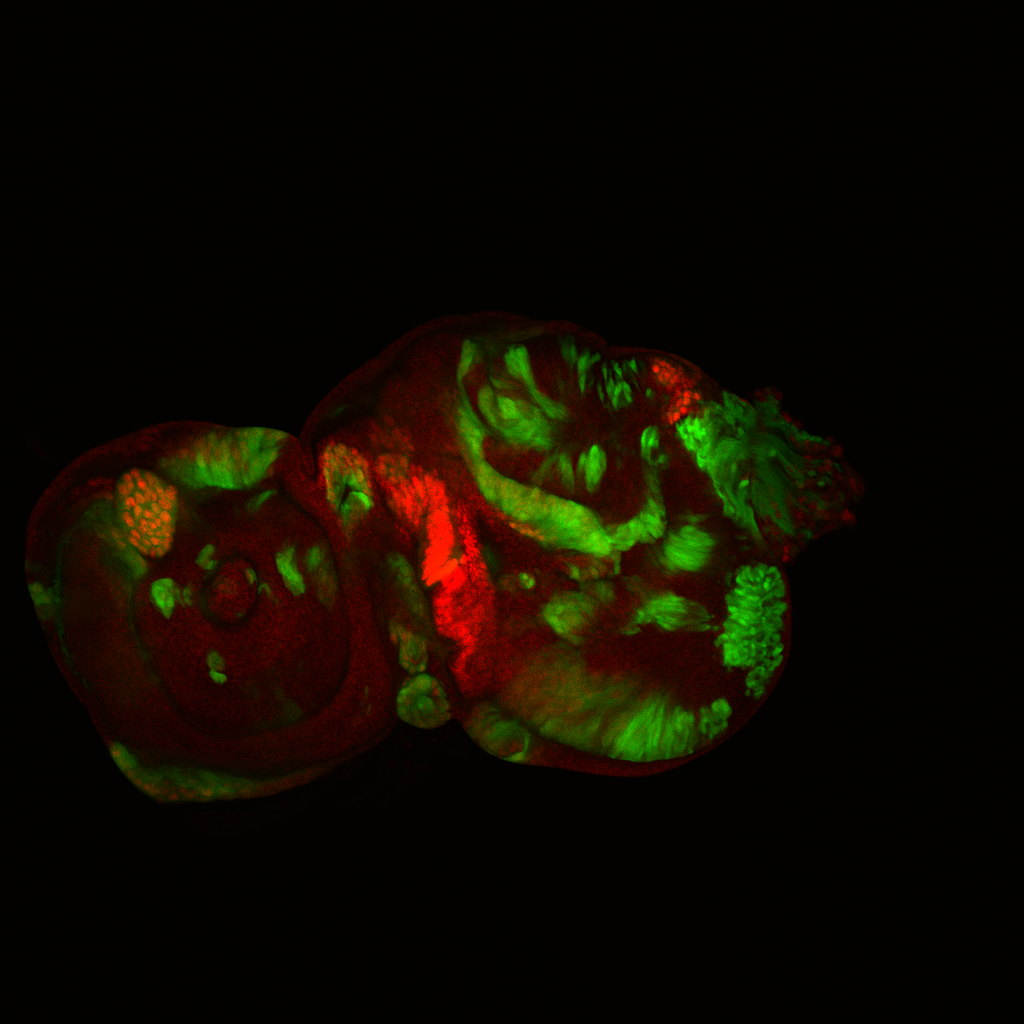

Supplement: Supplementary file 7 — Source data Fig. 3 [file 44318_2025_547_MOESM7_ESM.zip › Figure 3I/1-2 original image.tif]

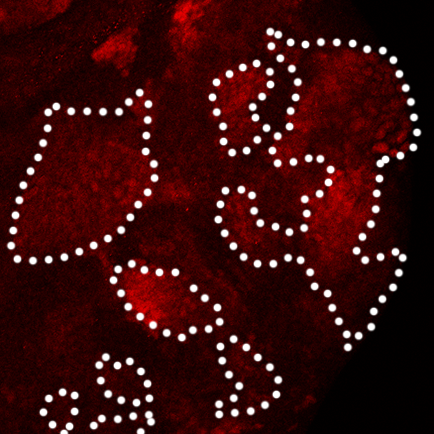

Supplement: Supplementary file 7 — Source data Fig. 3 [file 44318_2025_547_MOESM7_ESM.zip › Figure 3I/10-1 rotated and cut image with border line.tif]

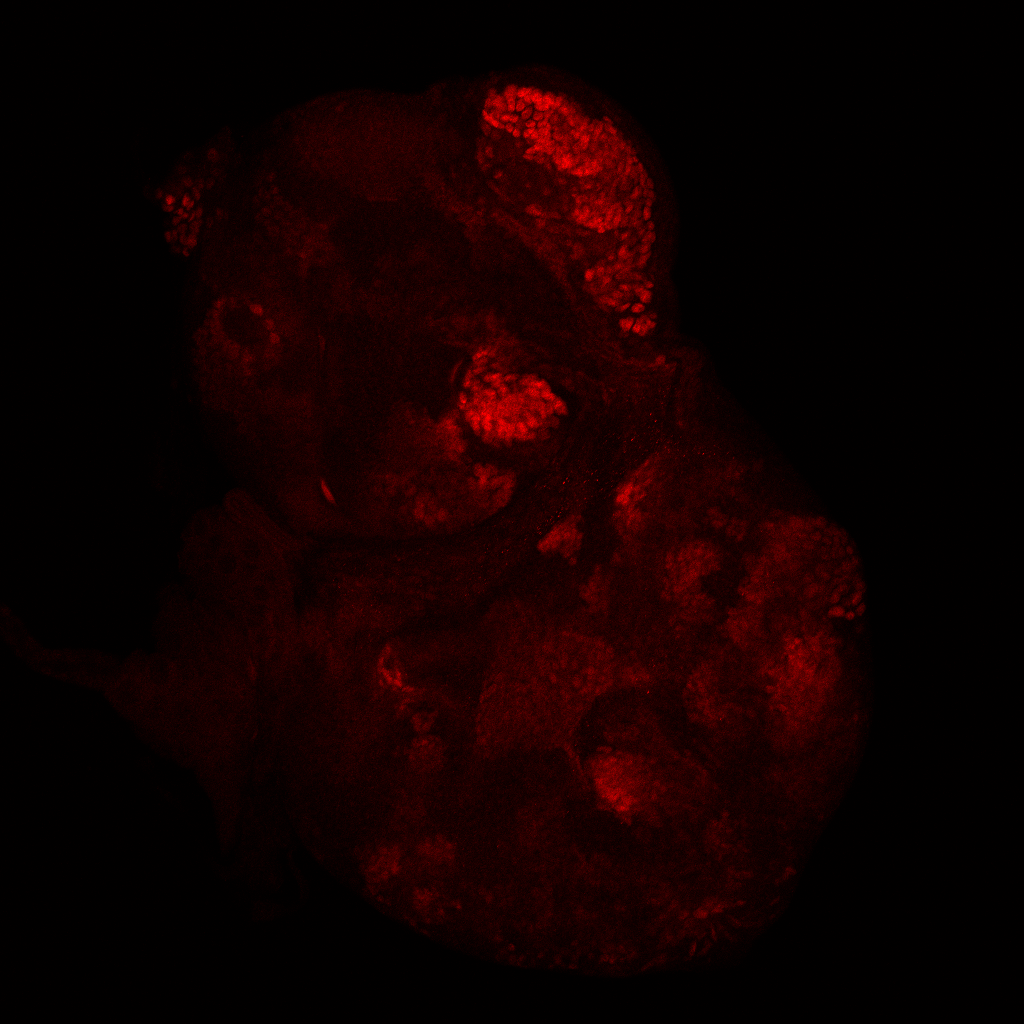

Supplement: Supplementary file 7 — Source data Fig. 3 [file 44318_2025_547_MOESM7_ESM.zip › Figure 3I/10-2 original image.tif]

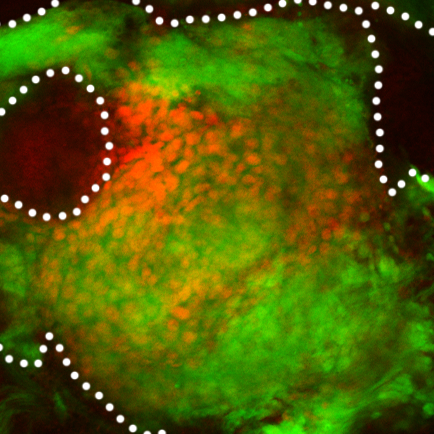

Supplement: Supplementary file 7 — Source data Fig. 3 [file 44318_2025_547_MOESM7_ESM.zip › Figure 3I/11-1 rotated and cut image with border line.tif]

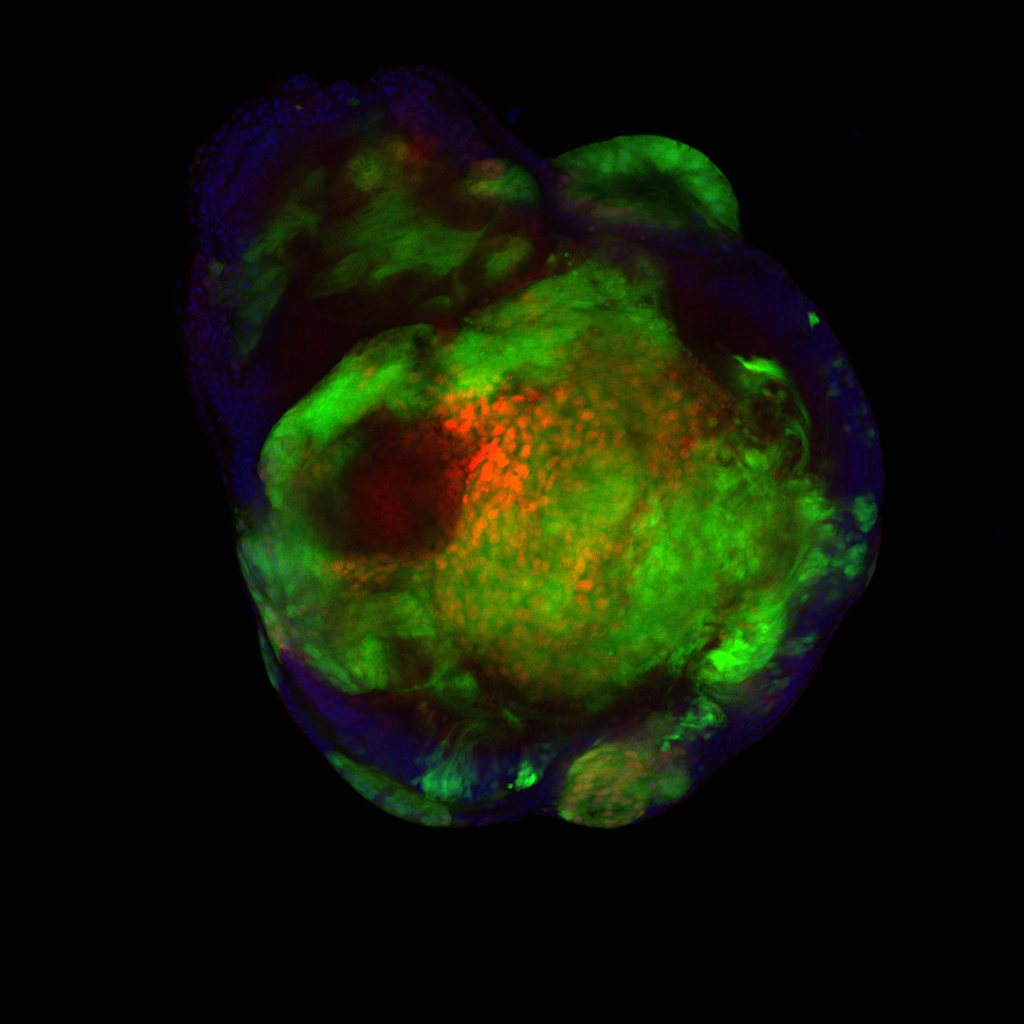

Supplement: Supplementary file 7 — Source data Fig. 3 [file 44318_2025_547_MOESM7_ESM.zip › Figure 3I/11-2 original image.tif]

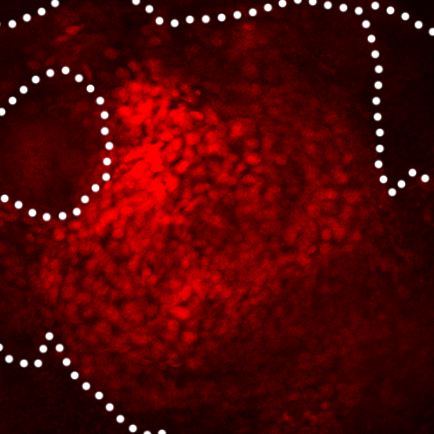

Supplement: Supplementary file 7 — Source data Fig. 3 [file 44318_2025_547_MOESM7_ESM.zip › Figure 3I/12-1 rotated and cut image with border line.tif]

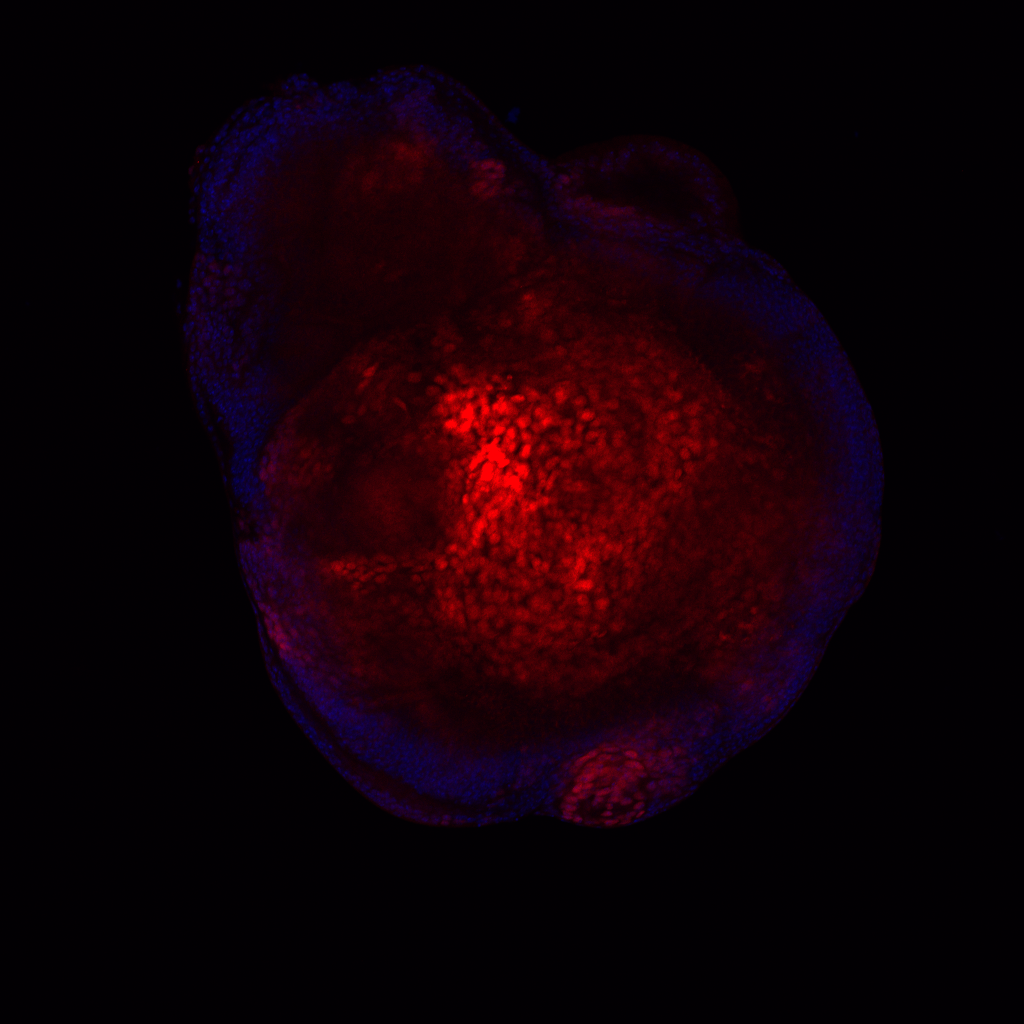

Supplement: Supplementary file 7 — Source data Fig. 3 [file 44318_2025_547_MOESM7_ESM.zip › Figure 3I/12-2 original image.tif]

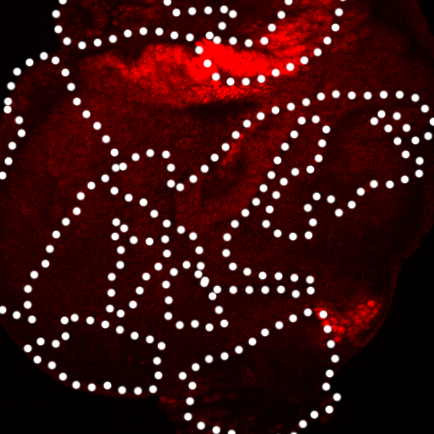

Supplement: Supplementary file 7 — Source data Fig. 3 [file 44318_2025_547_MOESM7_ESM.zip › Figure 3I/2-1 rotated and cut image with border line.tif]

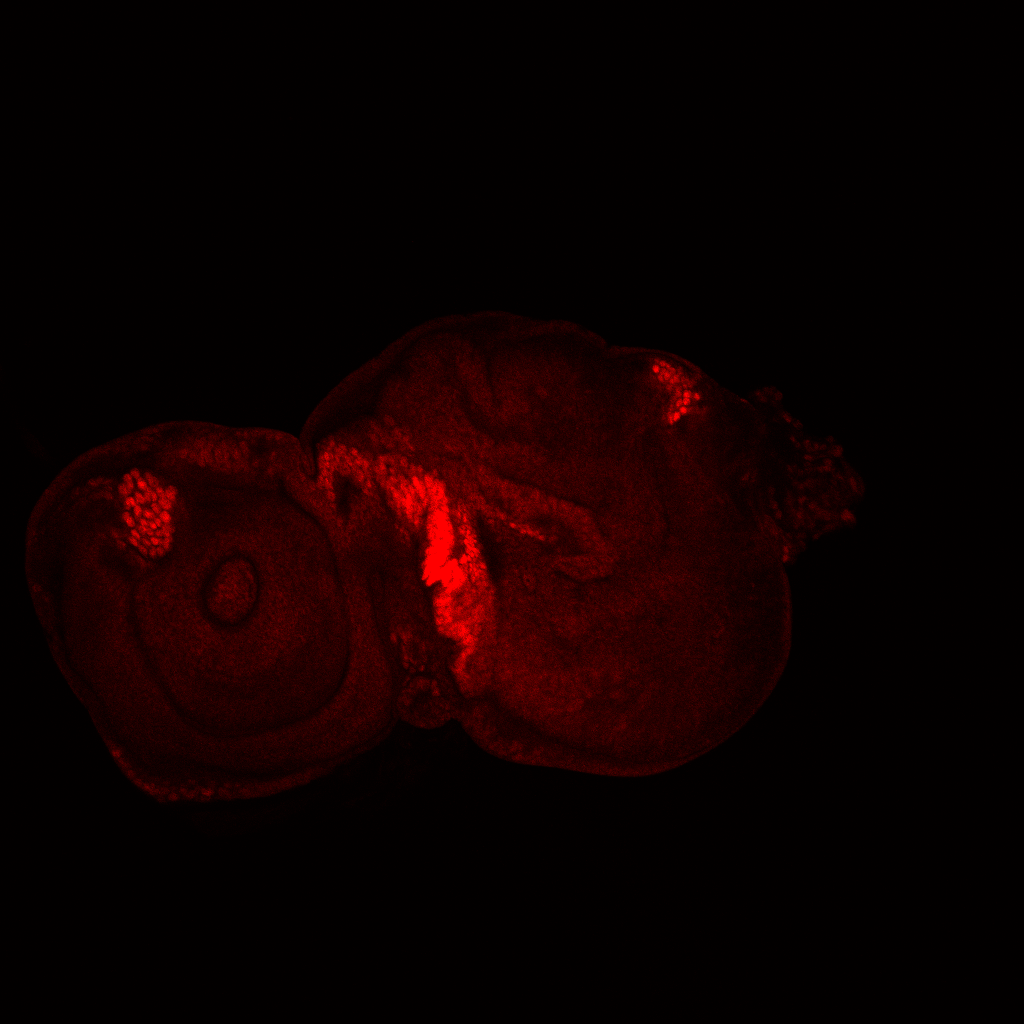

Supplement: Supplementary file 7 — Source data Fig. 3 [file 44318_2025_547_MOESM7_ESM.zip › Figure 3I/2-2 original image.tif]

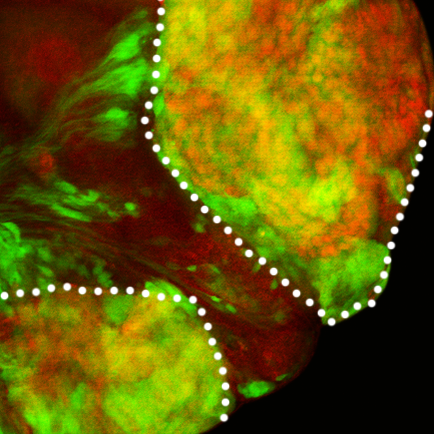

Supplement: Supplementary file 7 — Source data Fig. 3 [file 44318_2025_547_MOESM7_ESM.zip › Figure 3I/3-1 rotated and cut image with border line.tif]
