# Supplementary figures and images for: Hemocytes facilitate interclonal cooperation-induced tumor malignancy by hijacking the innate immune system in Drosophila (part 2 of 4)
Source: EMBO J. 2025 Aug 22;44(19):5394–428. doi: 10.1038/s44318-025-00547-5 (PMC12489090; doi:10.1038/s44318-025-00547-5)

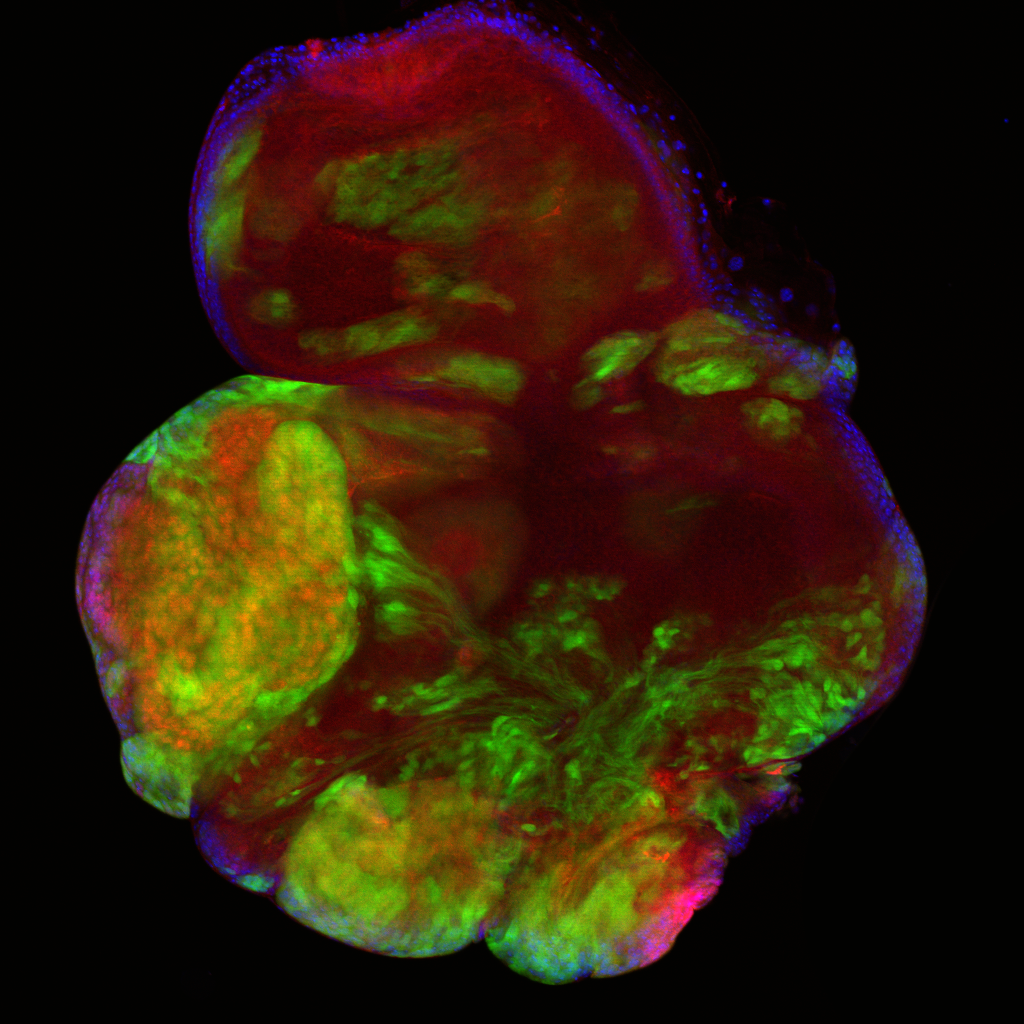

Supplement: Supplementary file 7 — Source data Fig. 3 [file 44318_2025_547_MOESM7_ESM.zip › Figure 3I/3-2 original image.tif]

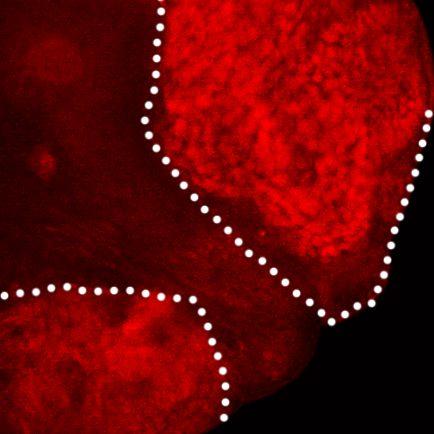

Supplement: Supplementary file 7 — Source data Fig. 3 [file 44318_2025_547_MOESM7_ESM.zip › Figure 3I/4-1 rotated and cut image with border line.tif]

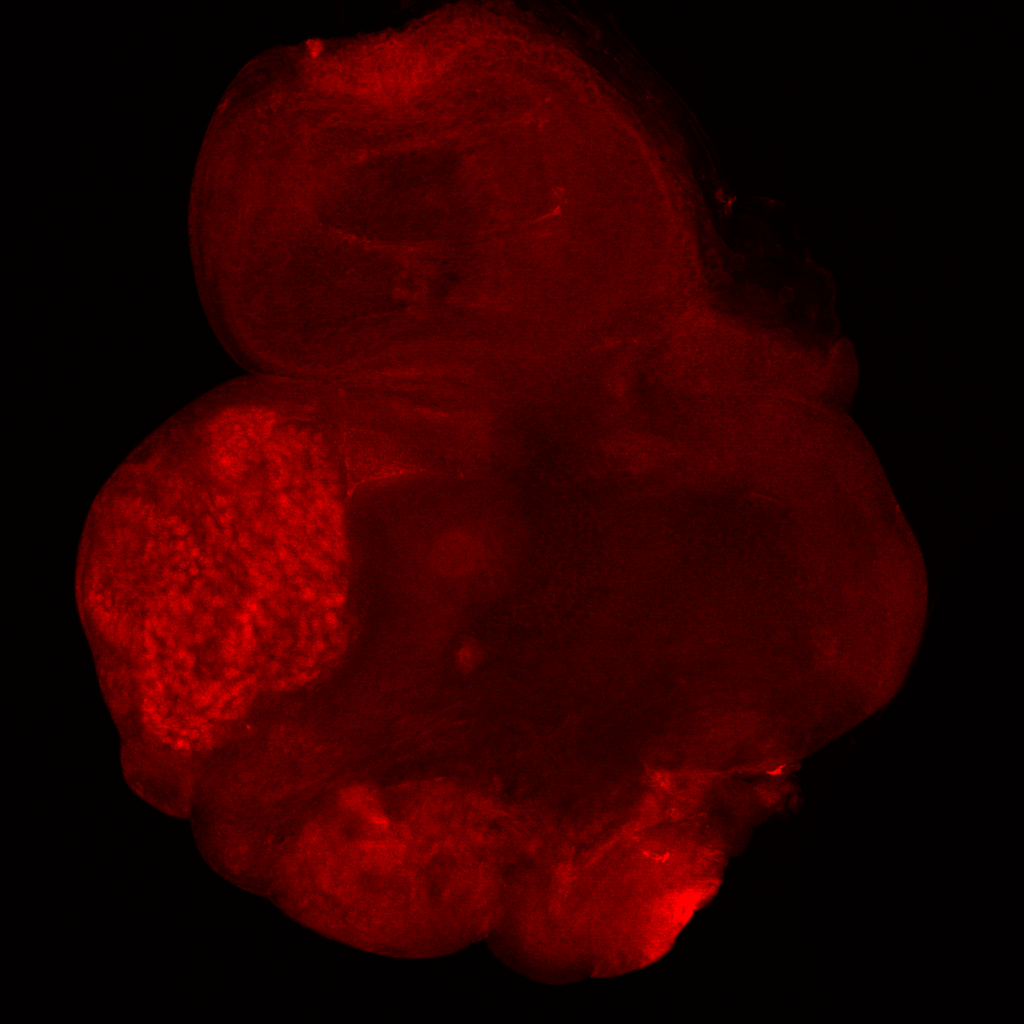

Supplement: Supplementary file 7 — Source data Fig. 3 [file 44318_2025_547_MOESM7_ESM.zip › Figure 3I/4-2 original image.tif]

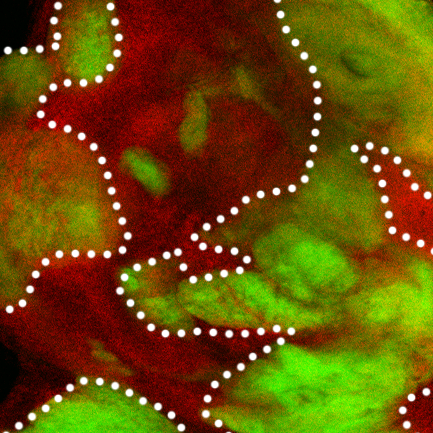

Supplement: Supplementary file 7 — Source data Fig. 3 [file 44318_2025_547_MOESM7_ESM.zip › Figure 3I/5-1 rotated and cut image with border line.tif]

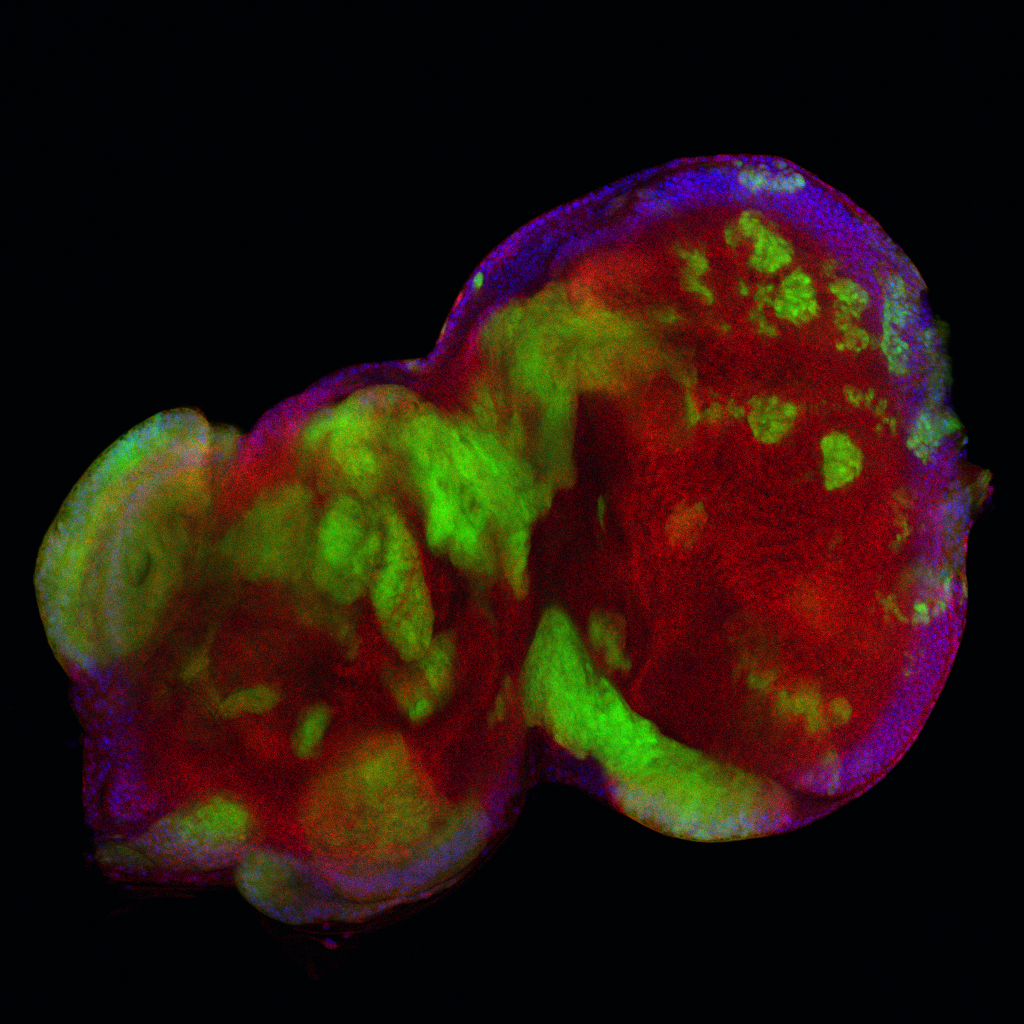

Supplement: Supplementary file 7 — Source data Fig. 3 [file 44318_2025_547_MOESM7_ESM.zip › Figure 3I/5-2 original image.tif]

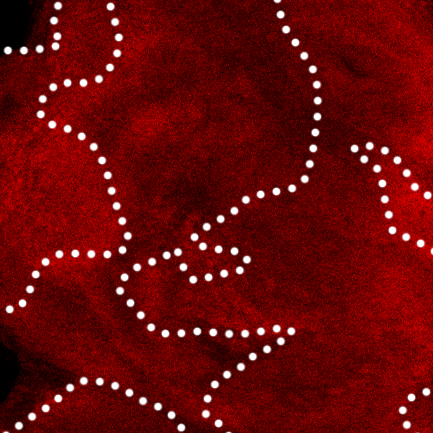

Supplement: Supplementary file 7 — Source data Fig. 3 [file 44318_2025_547_MOESM7_ESM.zip › Figure 3I/6-1 rotated and cut image with border line.tif]

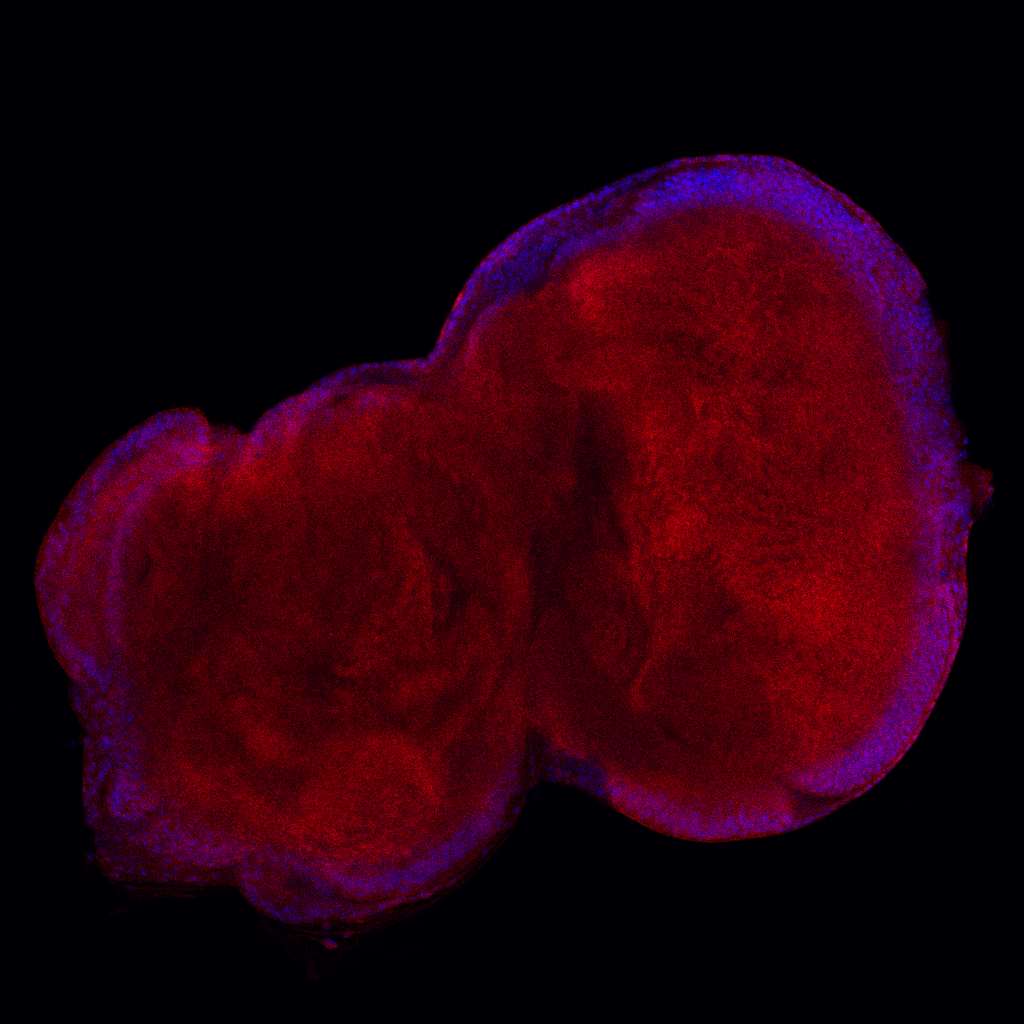

Supplement: Supplementary file 7 — Source data Fig. 3 [file 44318_2025_547_MOESM7_ESM.zip › Figure 3I/6-2 original image.tif]

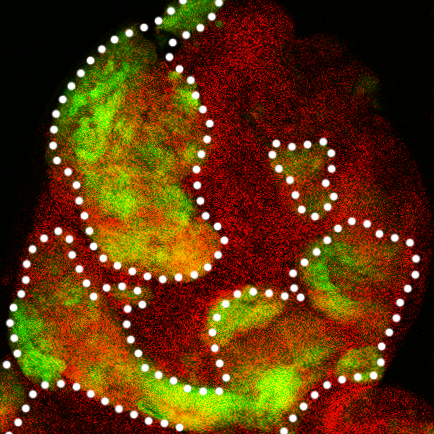

Supplement: Supplementary file 7 — Source data Fig. 3 [file 44318_2025_547_MOESM7_ESM.zip › Figure 3I/7-1 rotated and cut image with border line.tif]

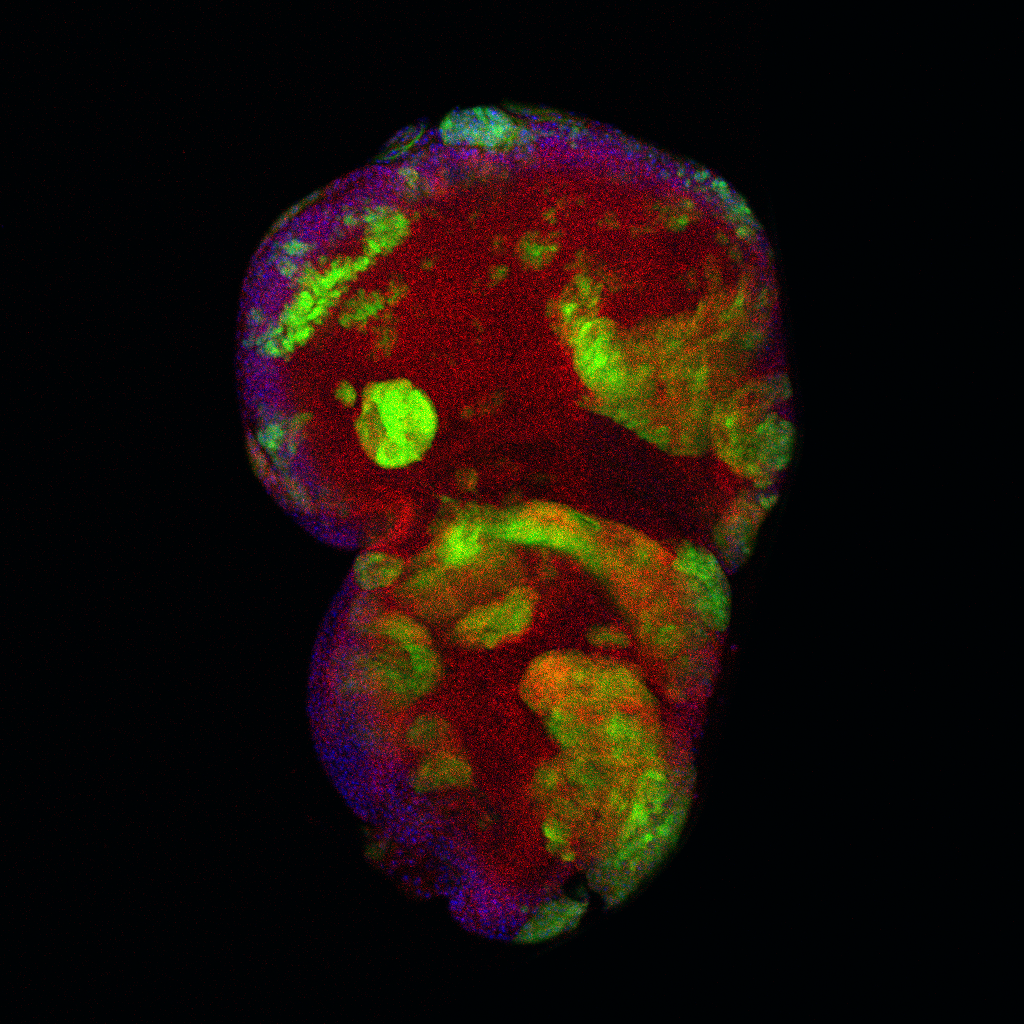

Supplement: Supplementary file 7 — Source data Fig. 3 [file 44318_2025_547_MOESM7_ESM.zip › Figure 3I/7-2 original image.tif]

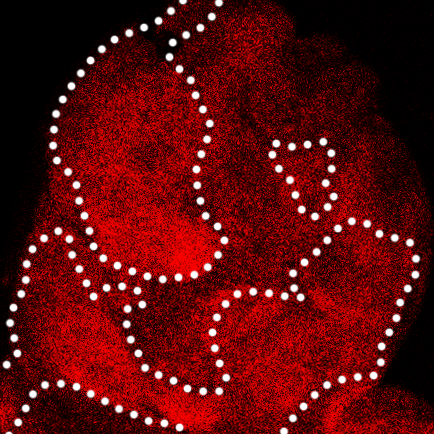

Supplement: Supplementary file 7 — Source data Fig. 3 [file 44318_2025_547_MOESM7_ESM.zip › Figure 3I/8-1 rotated and cut image with border line.tif]

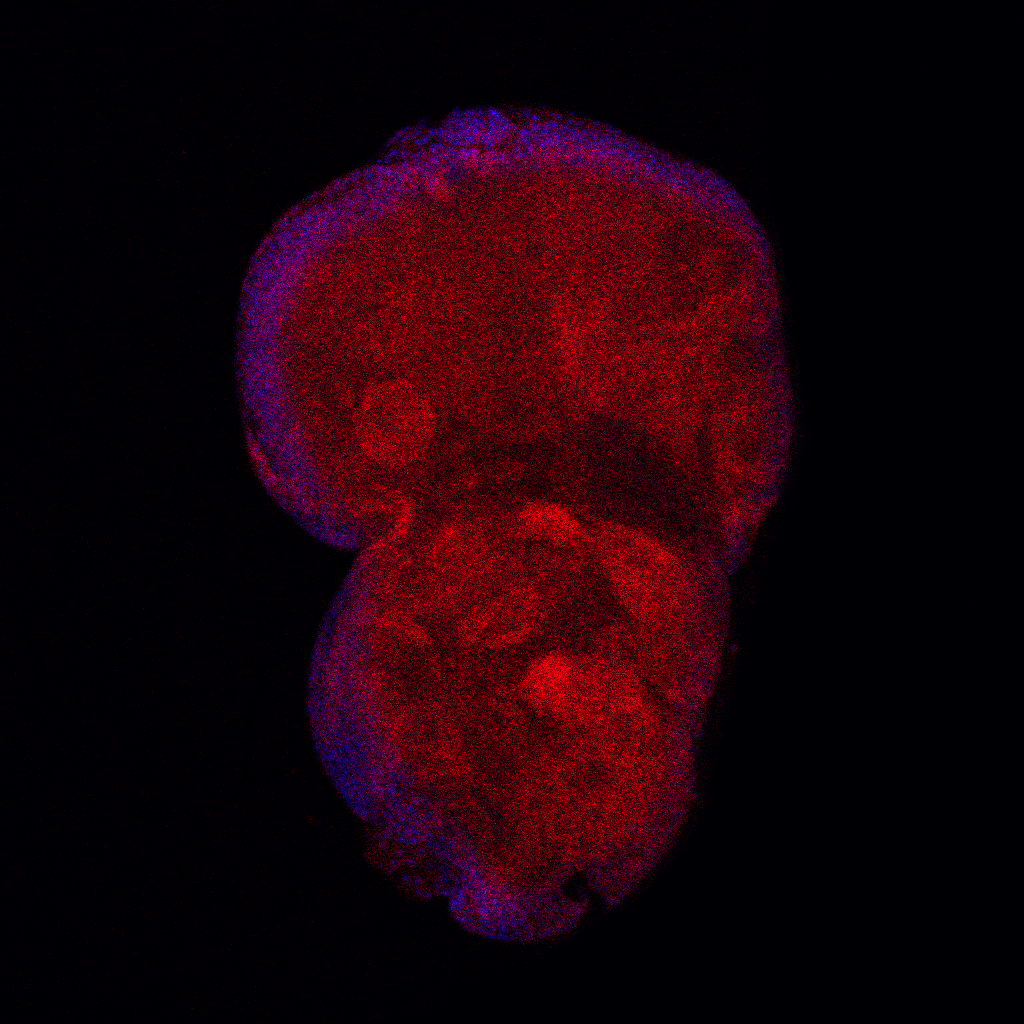

Supplement: Supplementary file 7 — Source data Fig. 3 [file 44318_2025_547_MOESM7_ESM.zip › Figure 3I/8-2 original image.tif]

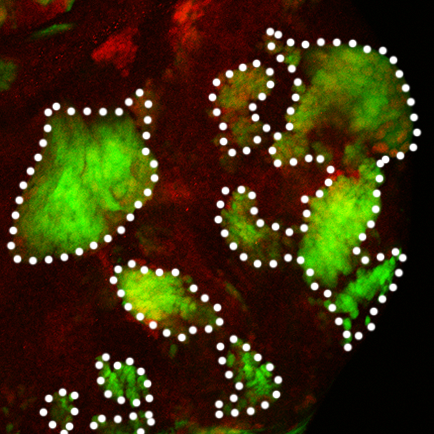

Supplement: Supplementary file 7 — Source data Fig. 3 [file 44318_2025_547_MOESM7_ESM.zip › Figure 3I/9-1 rotated and cut image with border line.tif]

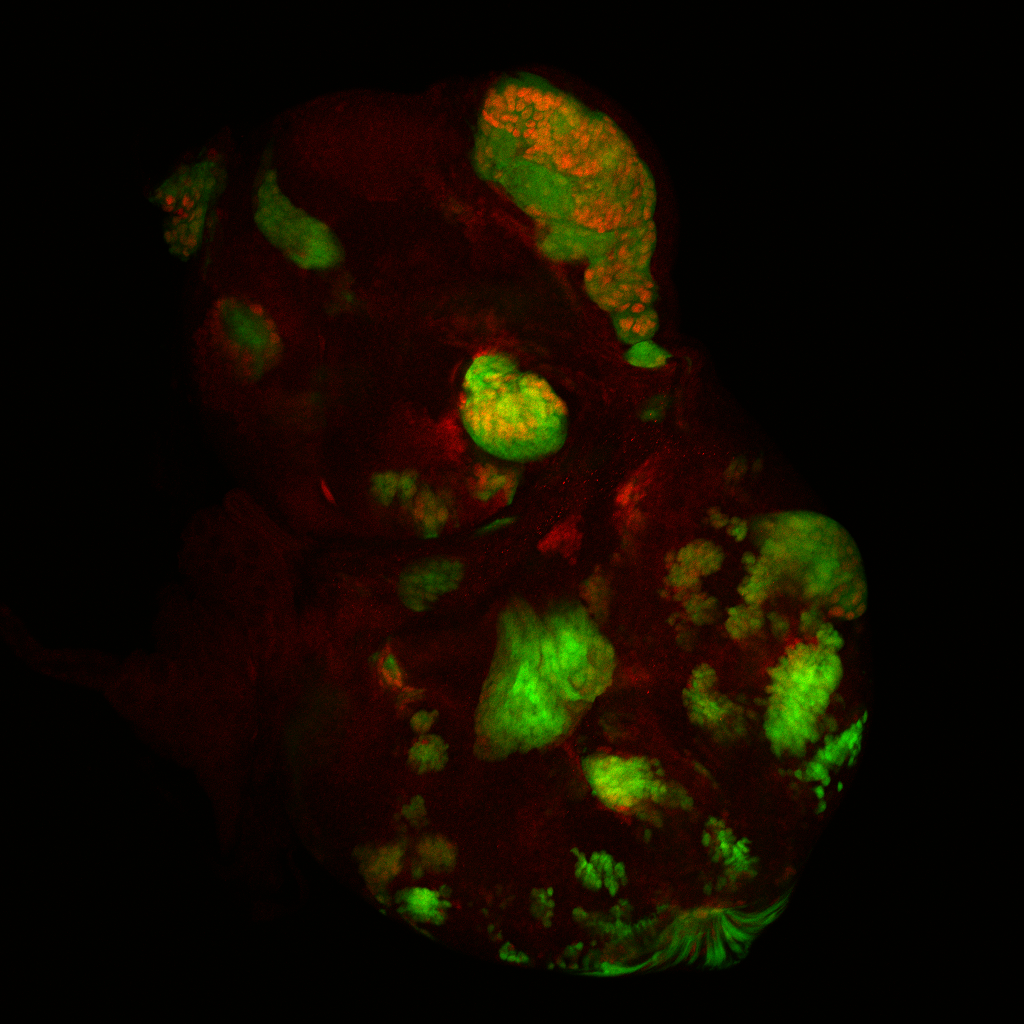

Supplement: Supplementary file 7 — Source data Fig. 3 [file 44318_2025_547_MOESM7_ESM.zip › Figure 3I/9-2 original image.tif]

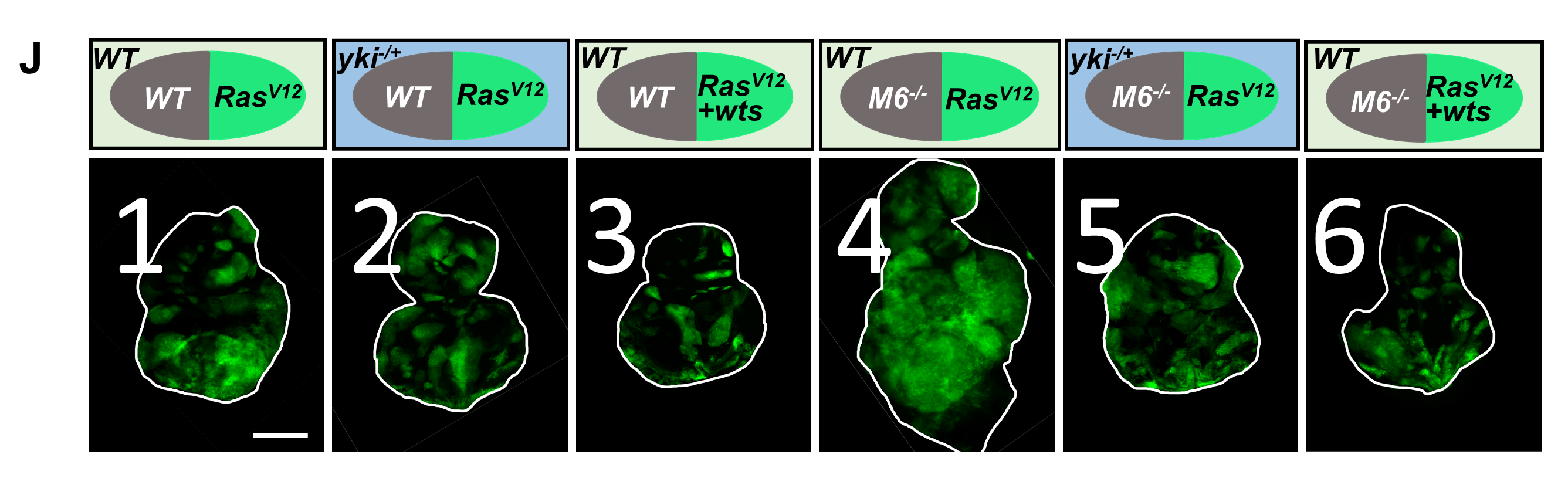

Supplement: Supplementary file 7 — Source data Fig. 3 [file 44318_2025_547_MOESM7_ESM.zip › Figure 3J/0 paper Figure 3J with provided image sequence.tif]

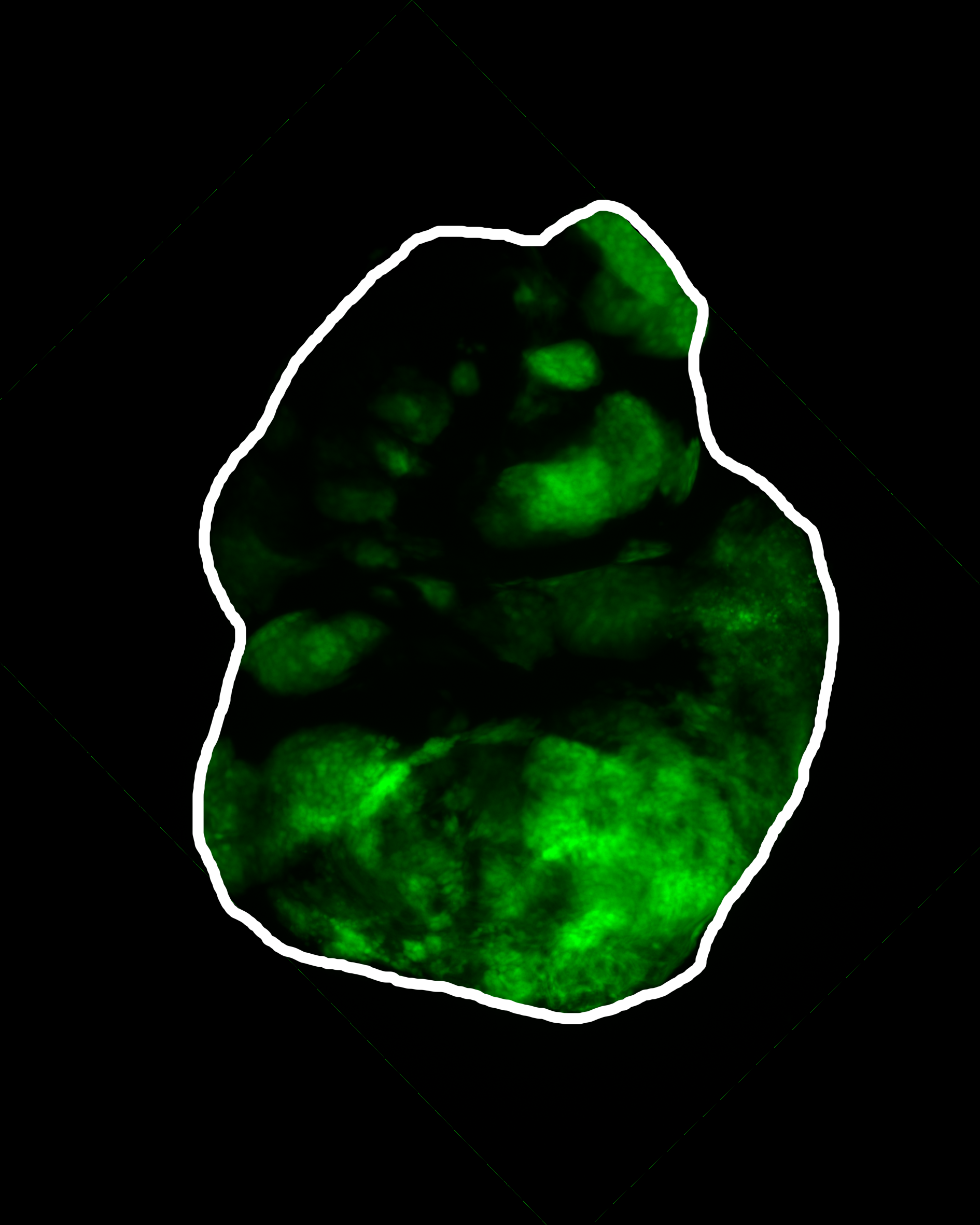

Supplement: Supplementary file 7 — Source data Fig. 3 [file 44318_2025_547_MOESM7_ESM.zip › Figure 3J/1-1 rotated and cut image with border line.tif]

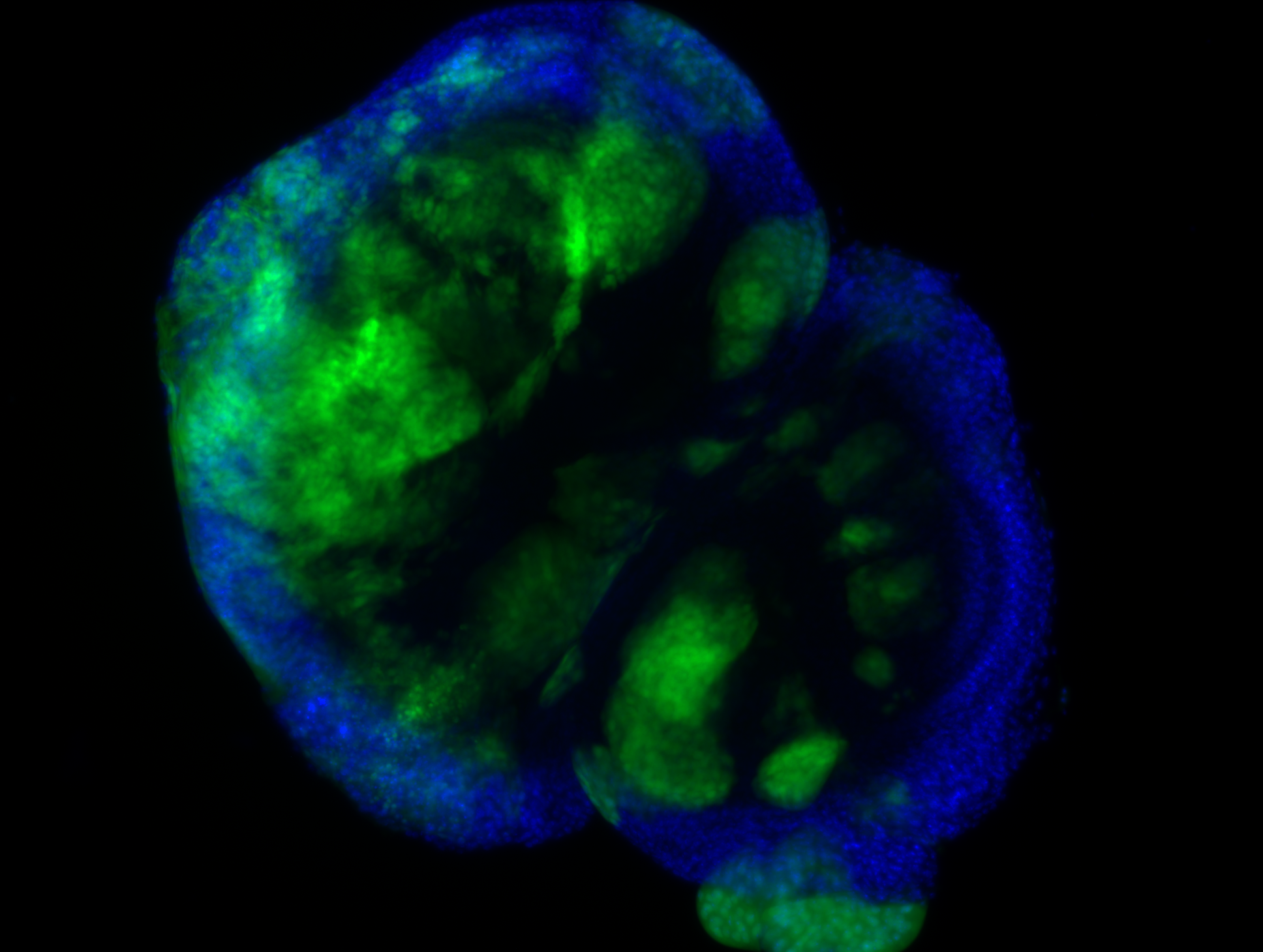

Supplement: Supplementary file 7 — Source data Fig. 3 [file 44318_2025_547_MOESM7_ESM.zip › Figure 3J/1-2 original image.tif]

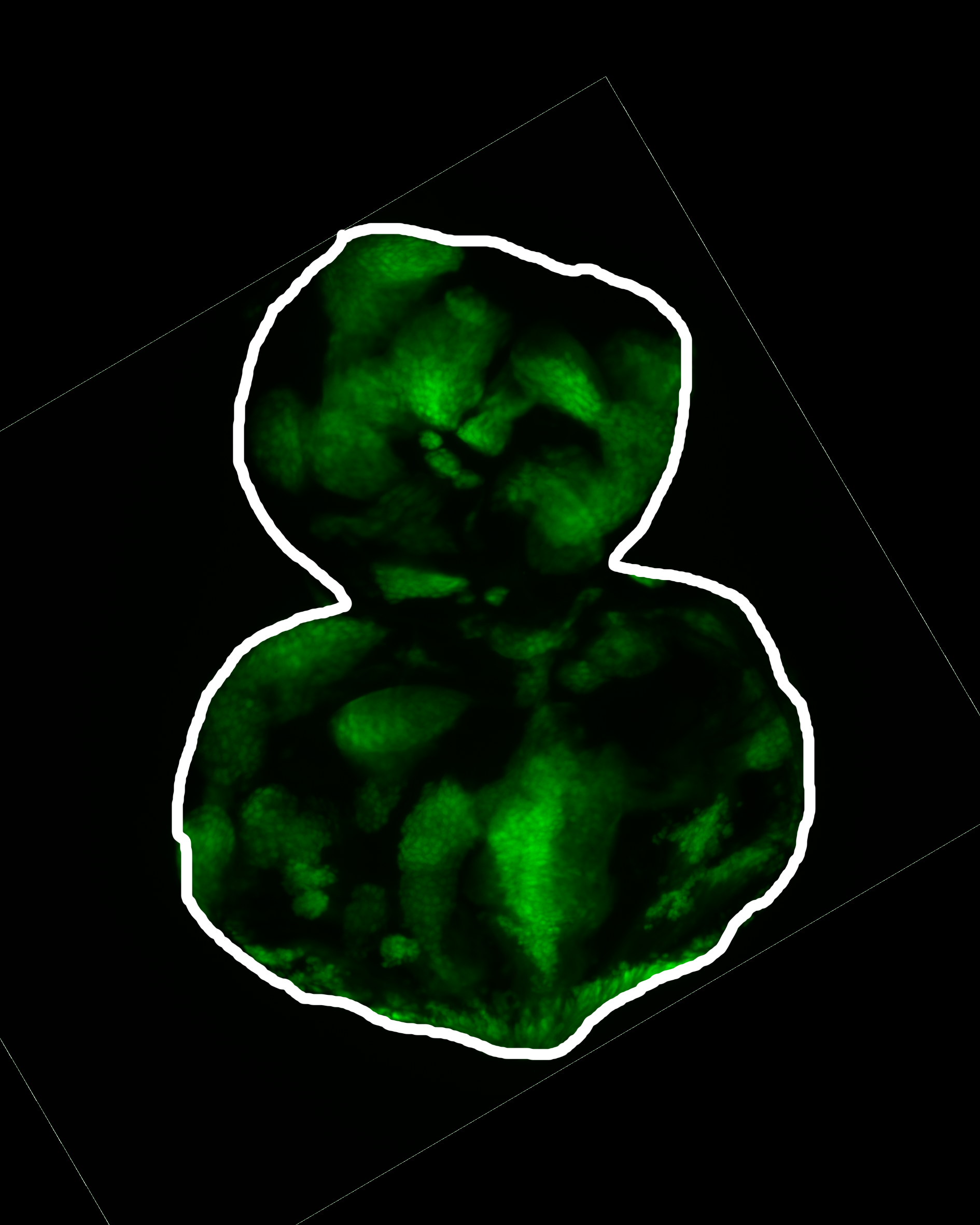

Supplement: Supplementary file 7 — Source data Fig. 3 [file 44318_2025_547_MOESM7_ESM.zip › Figure 3J/2-1 rotated and cut image with border line.tif]

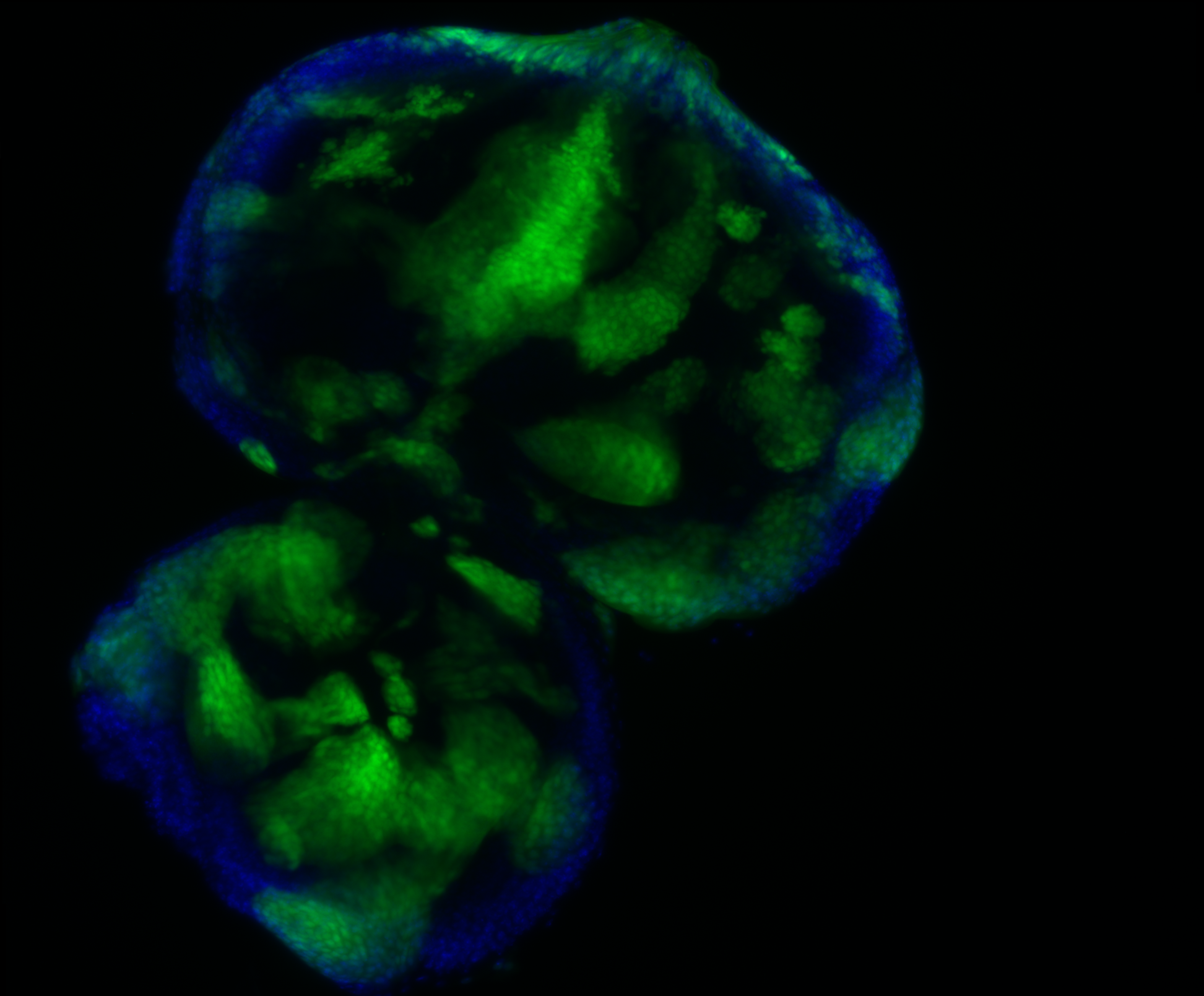

Supplement: Supplementary file 7 — Source data Fig. 3 [file 44318_2025_547_MOESM7_ESM.zip › Figure 3J/2-2 original image.tif]

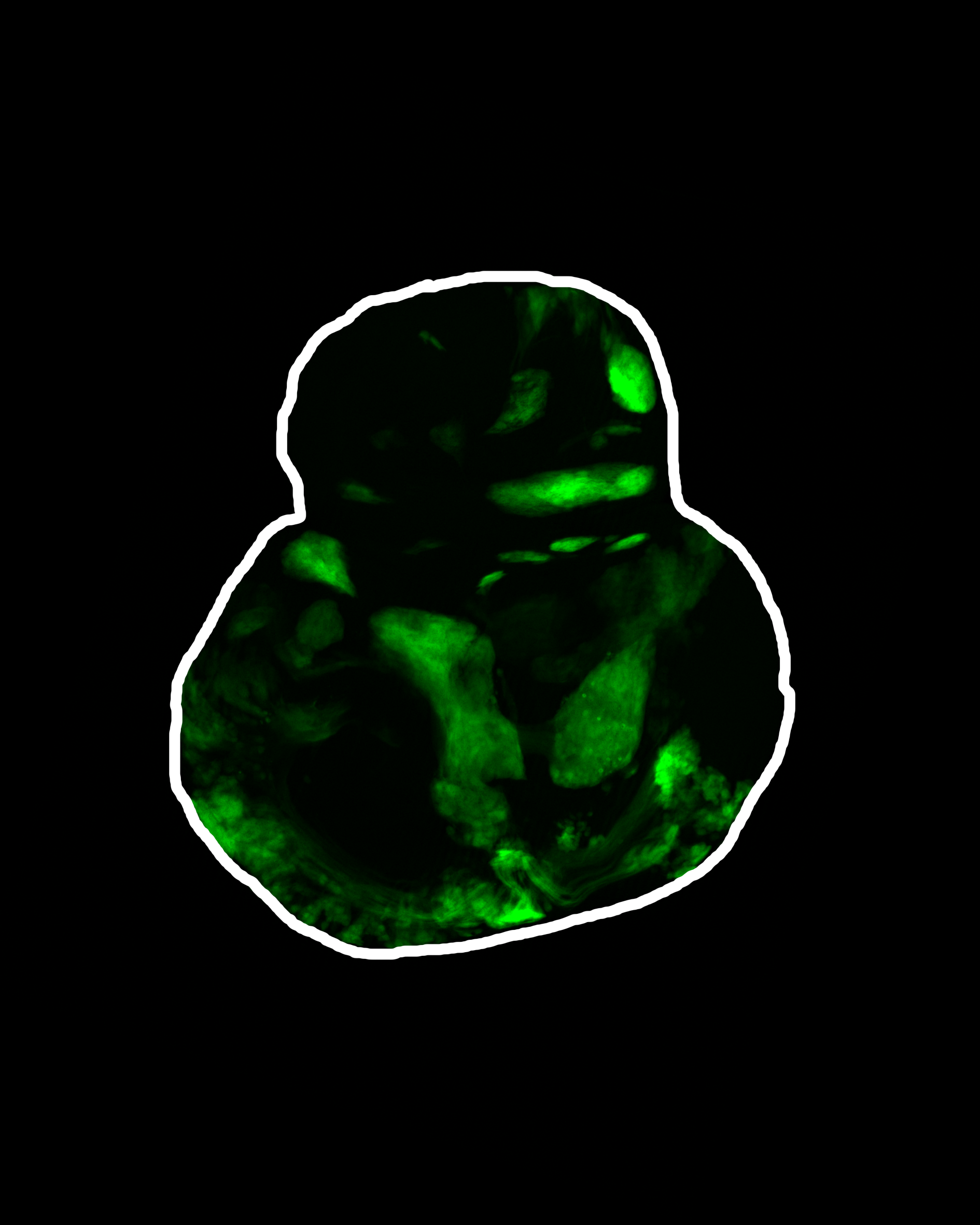

Supplement: Supplementary file 7 — Source data Fig. 3 [file 44318_2025_547_MOESM7_ESM.zip › Figure 3J/3-1 rotated and cut image with border line.tif]

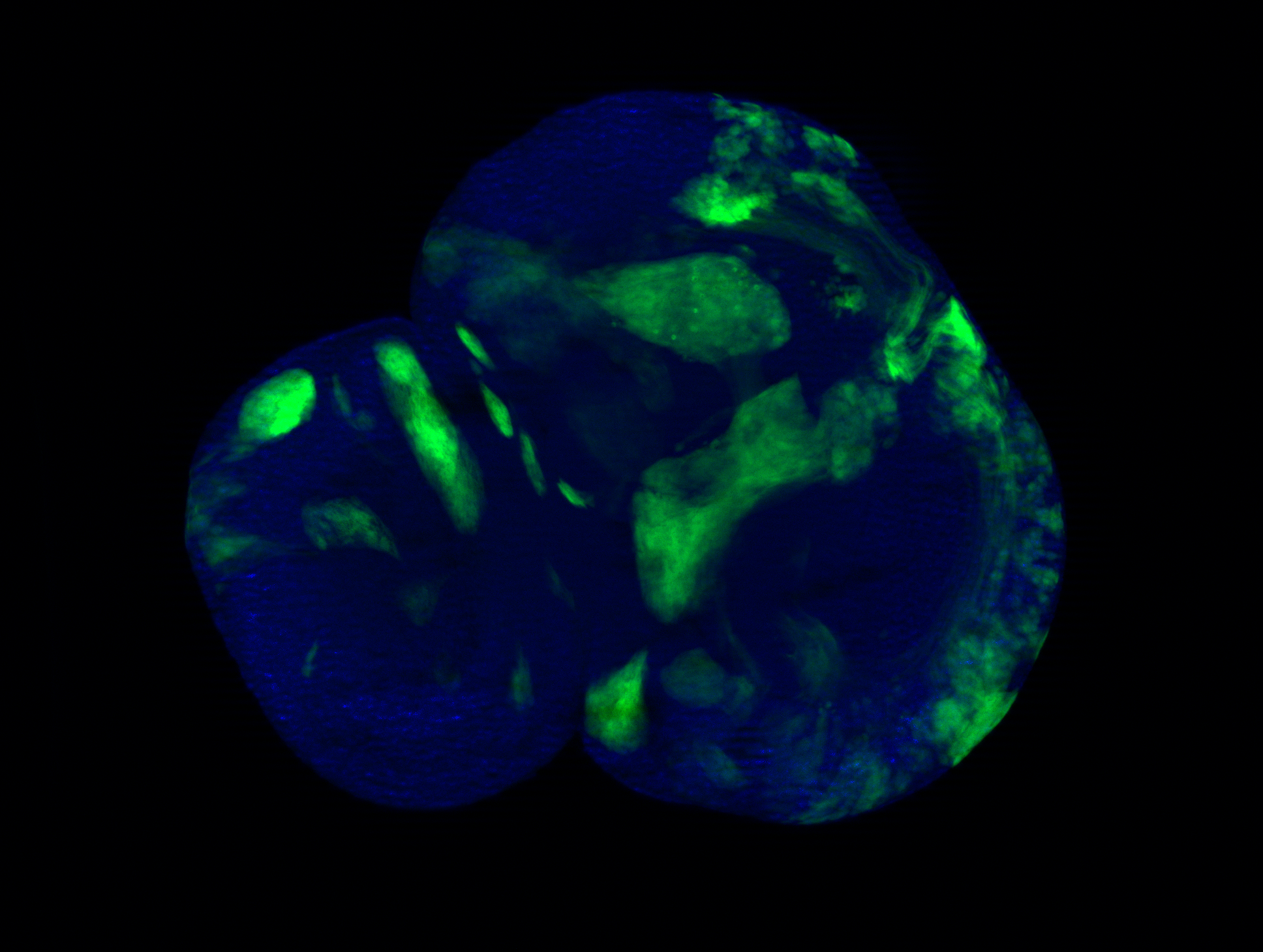

Supplement: Supplementary file 7 — Source data Fig. 3 [file 44318_2025_547_MOESM7_ESM.zip › Figure 3J/3-2 original image.tif]

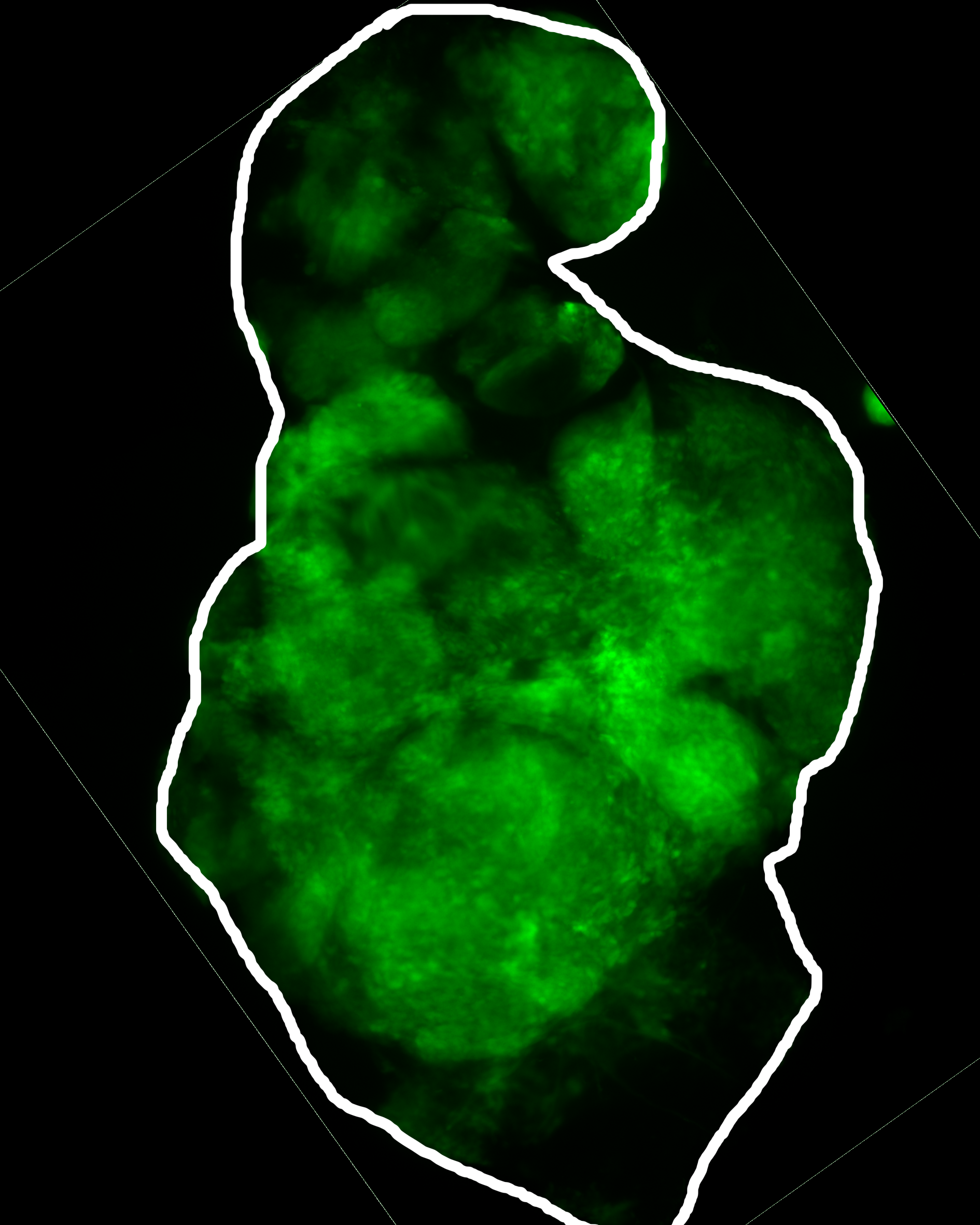

Supplement: Supplementary file 7 — Source data Fig. 3 [file 44318_2025_547_MOESM7_ESM.zip › Figure 3J/4-1 rotated and cut image with border line.tif]

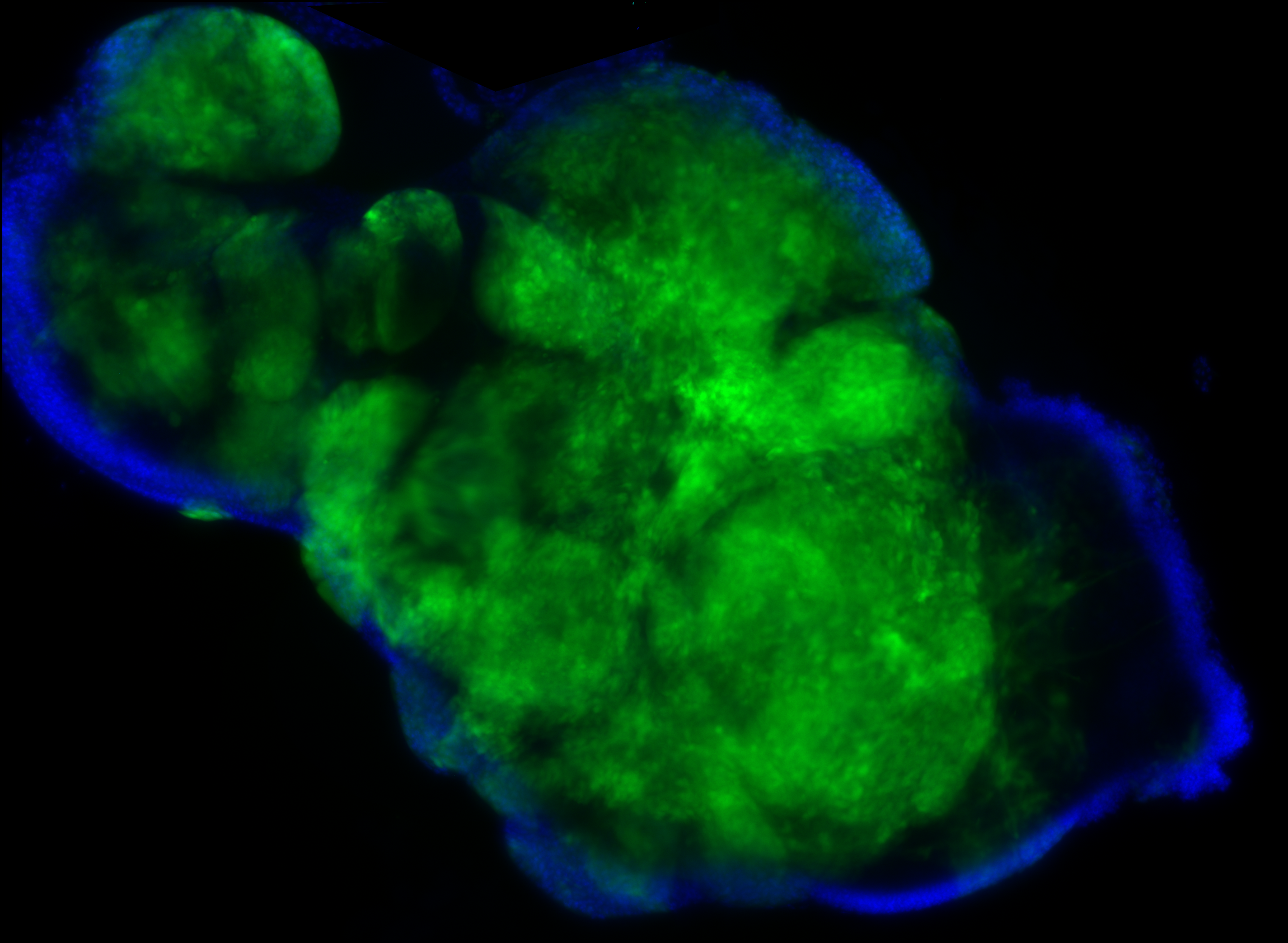

Supplement: Supplementary file 7 — Source data Fig. 3 [file 44318_2025_547_MOESM7_ESM.zip › Figure 3J/4-2 original image.tif]

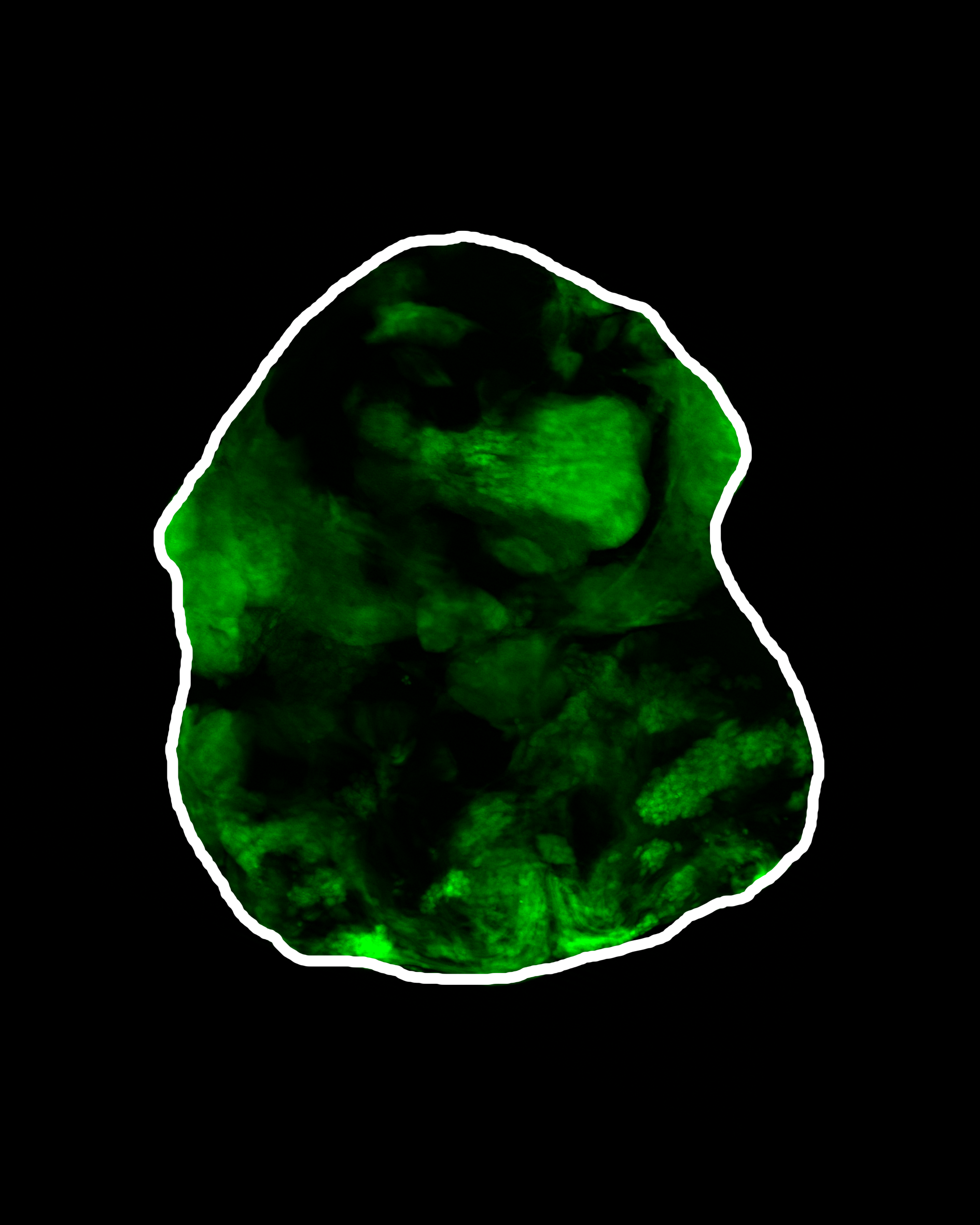

Supplement: Supplementary file 7 — Source data Fig. 3 [file 44318_2025_547_MOESM7_ESM.zip › Figure 3J/5-1 rotated and cut image with border line.tif]

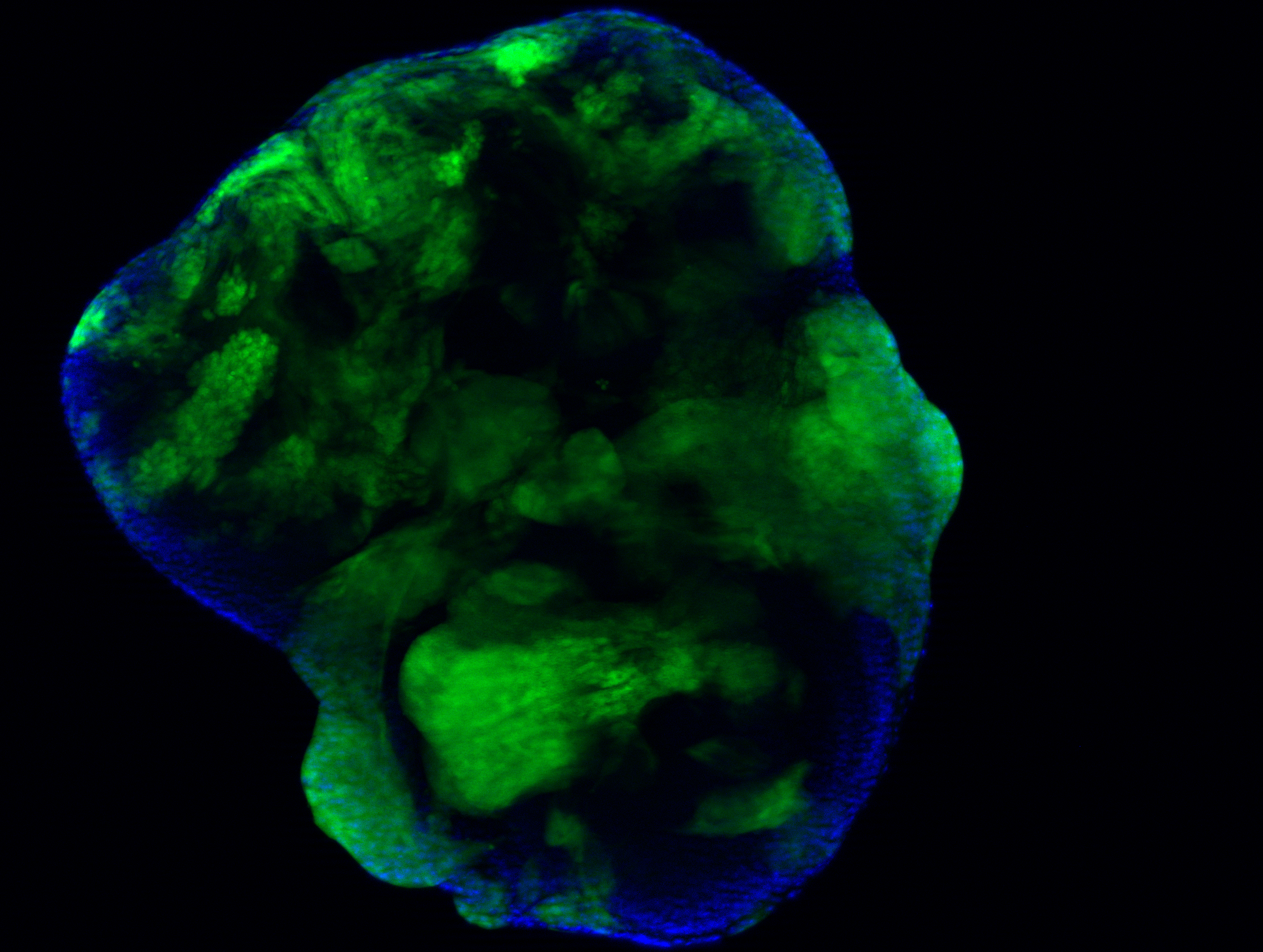

Supplement: Supplementary file 7 — Source data Fig. 3 [file 44318_2025_547_MOESM7_ESM.zip › Figure 3J/5-2 original image.tif]

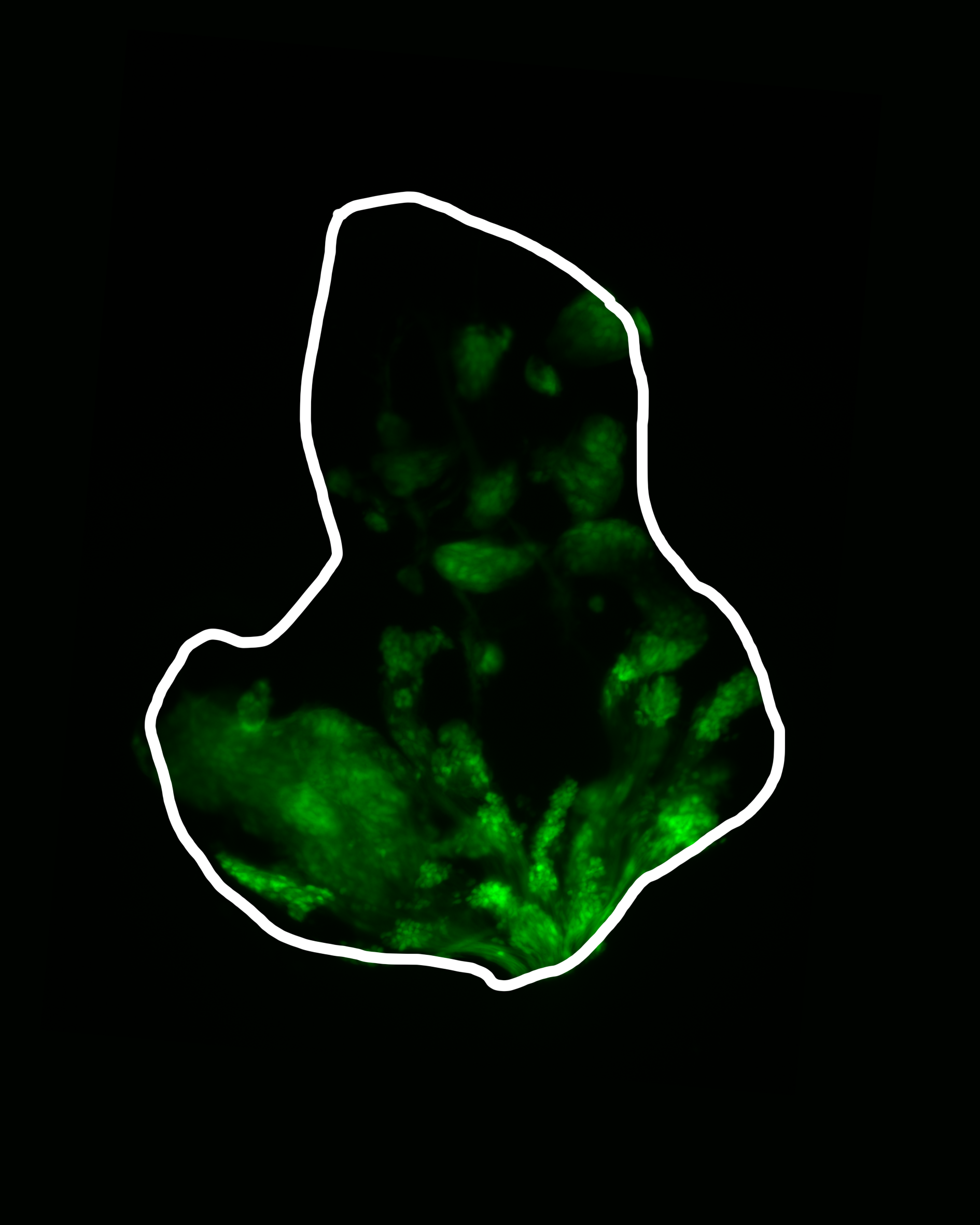

Supplement: Supplementary file 7 — Source data Fig. 3 [file 44318_2025_547_MOESM7_ESM.zip › Figure 3J/6-1 rotated and cut image with border line.tif]

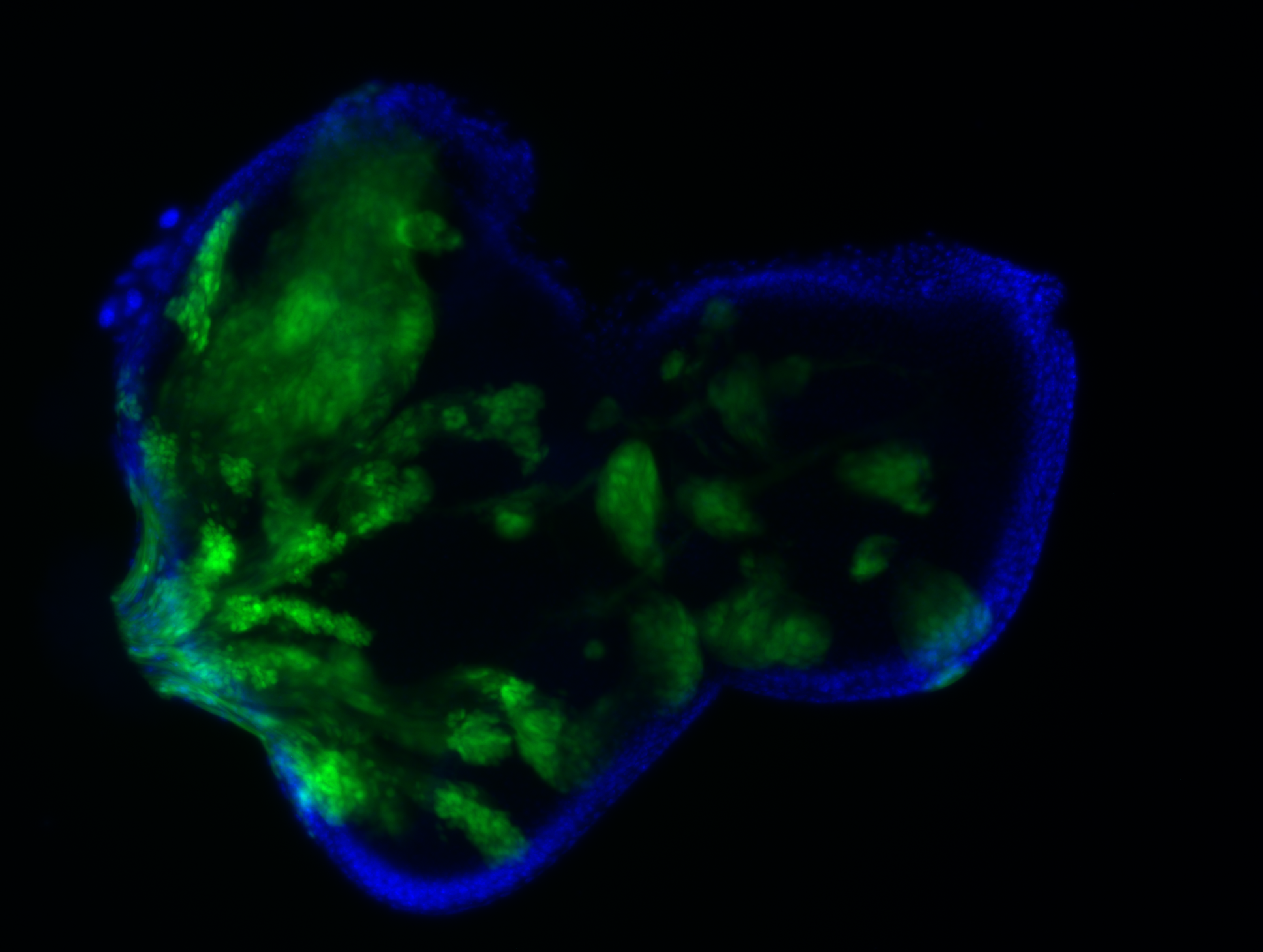

Supplement: Supplementary file 7 — Source data Fig. 3 [file 44318_2025_547_MOESM7_ESM.zip › Figure 3J/6-2 original image.tif]

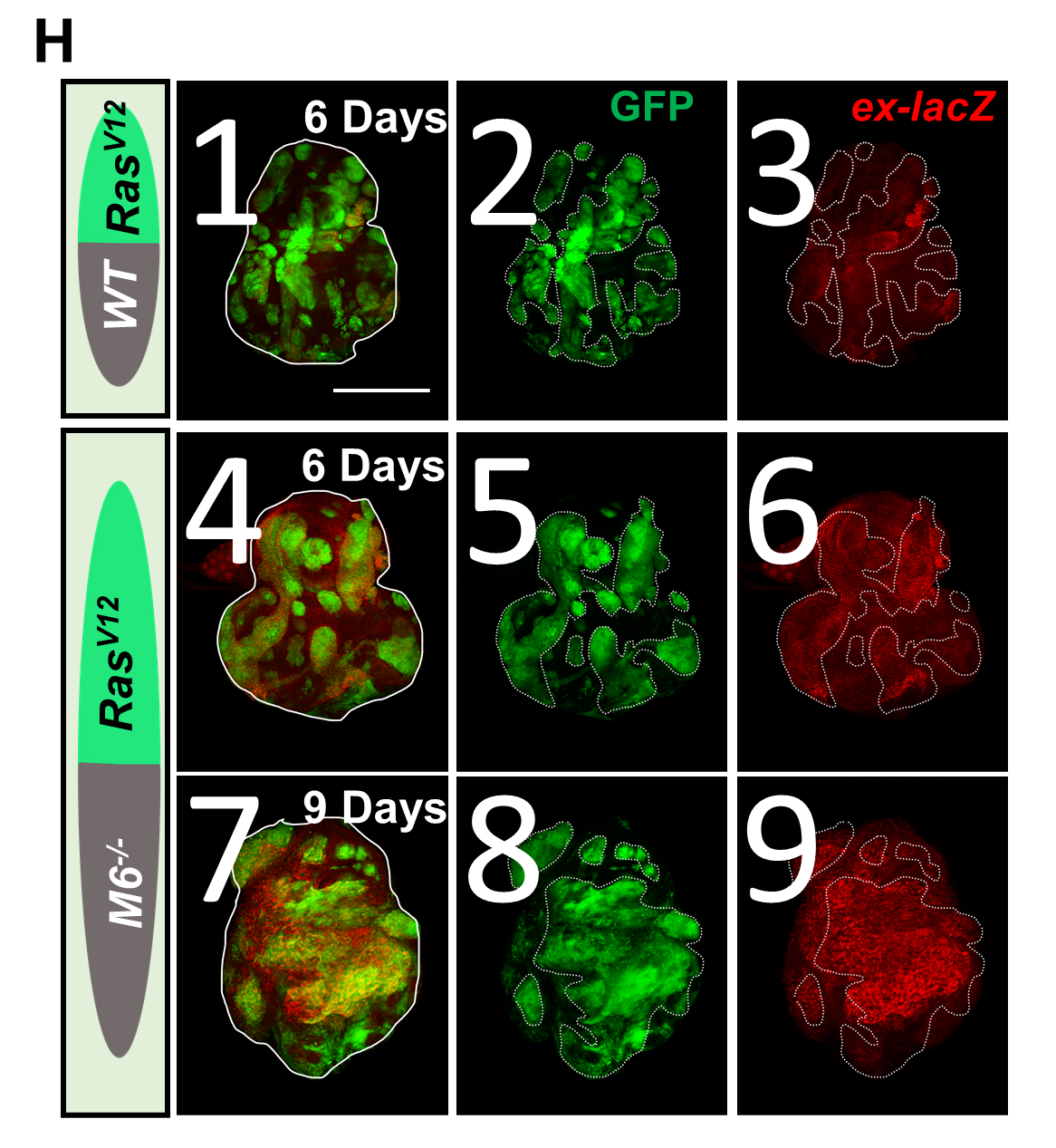

Supplement: Supplementary file 7 — Source data Fig. 3 [file 44318_2025_547_MOESM7_ESM.zip › Figure 3H/0 paper Figure 3H with provided image sequence.tif]

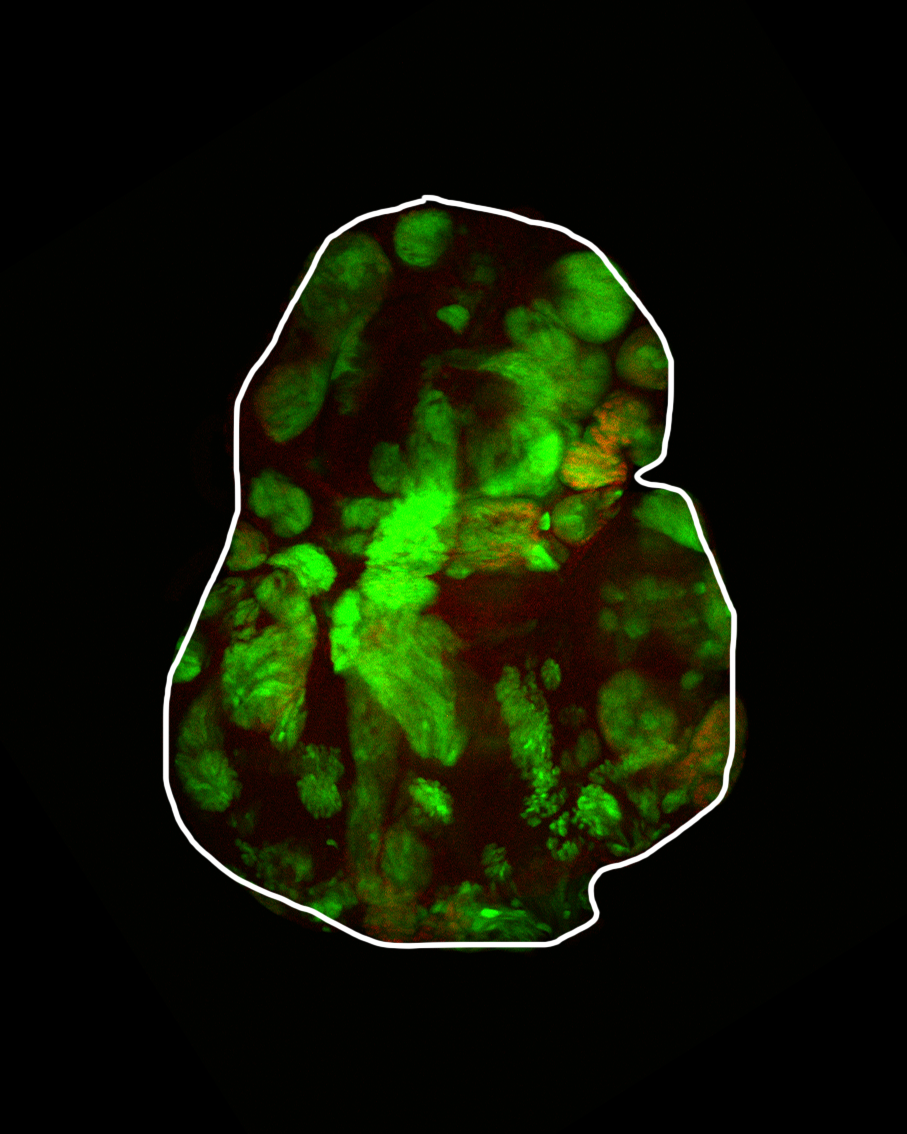

Supplement: Supplementary file 7 — Source data Fig. 3 [file 44318_2025_547_MOESM7_ESM.zip › Figure 3H/1-1 rotated and cut image with border line.tif]

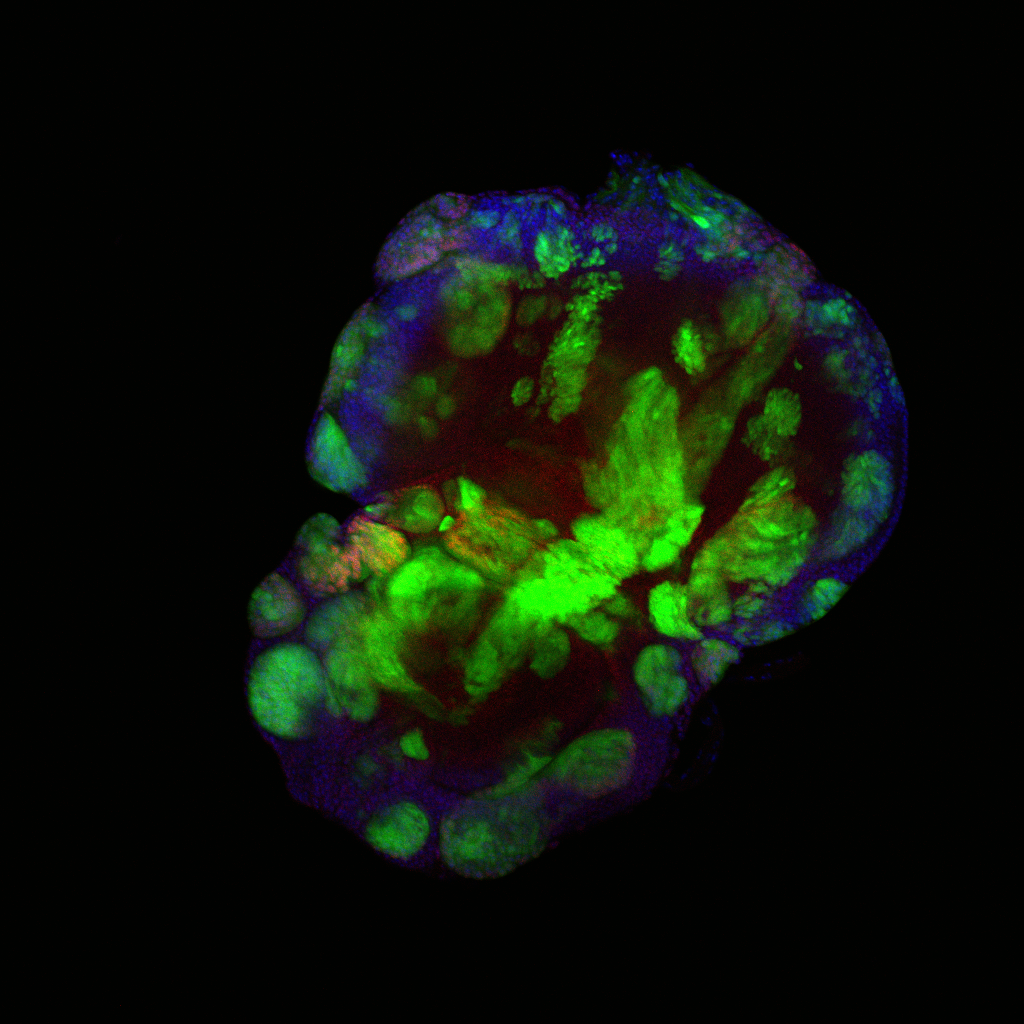

Supplement: Supplementary file 7 — Source data Fig. 3 [file 44318_2025_547_MOESM7_ESM.zip › Figure 3H/1-2 original image.tif]

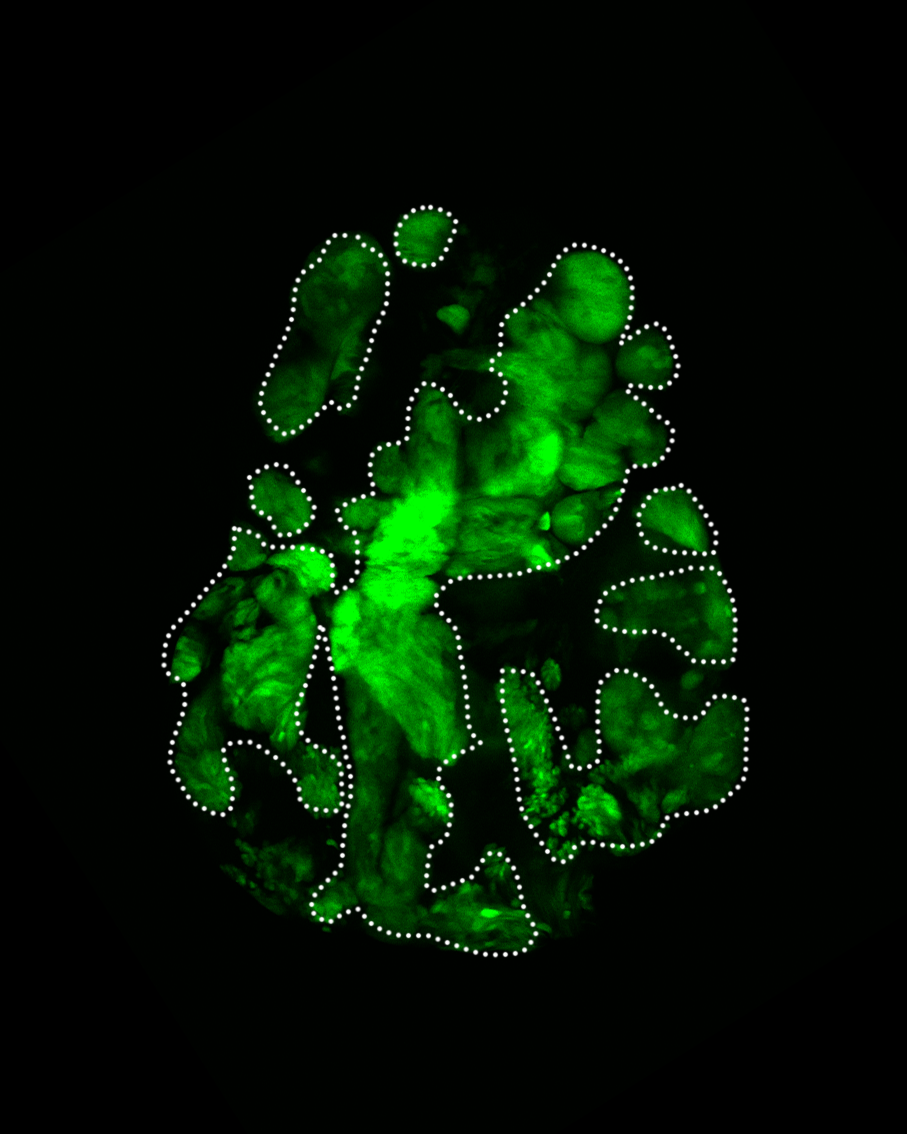

Supplement: Supplementary file 7 — Source data Fig. 3 [file 44318_2025_547_MOESM7_ESM.zip › Figure 3H/2-1 rotated and cut image with border line.tif]

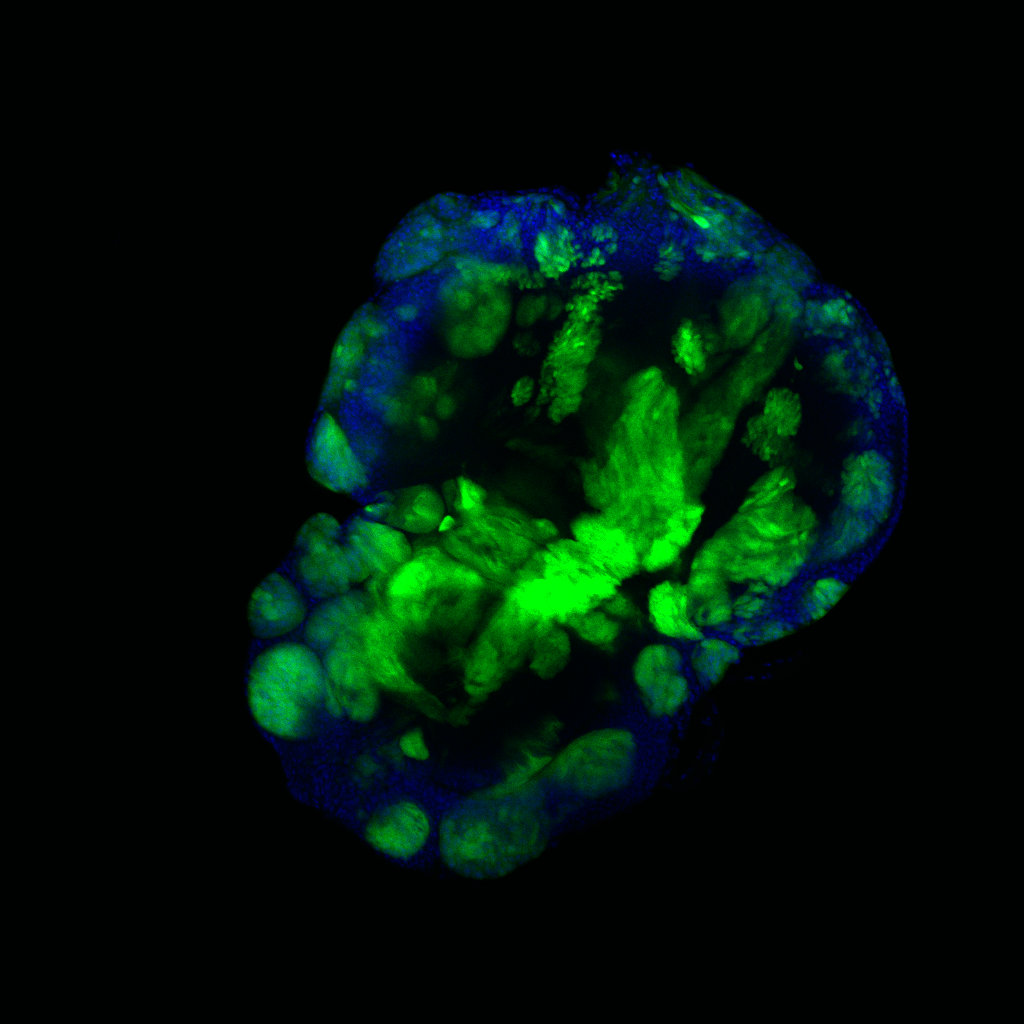

Supplement: Supplementary file 7 — Source data Fig. 3 [file 44318_2025_547_MOESM7_ESM.zip › Figure 3H/2-2 original image.tif]

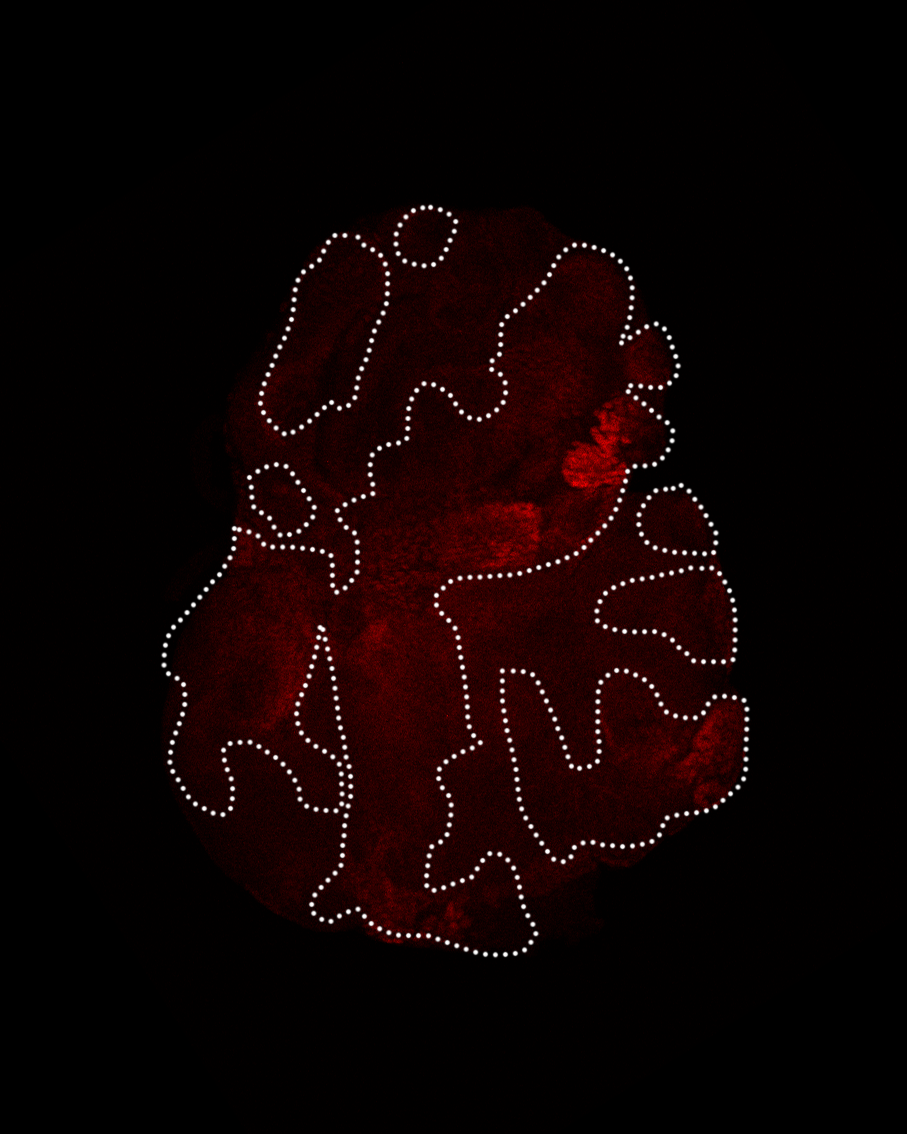

Supplement: Supplementary file 7 — Source data Fig. 3 [file 44318_2025_547_MOESM7_ESM.zip › Figure 3H/3-1 rotated and cut image with border line.tif]

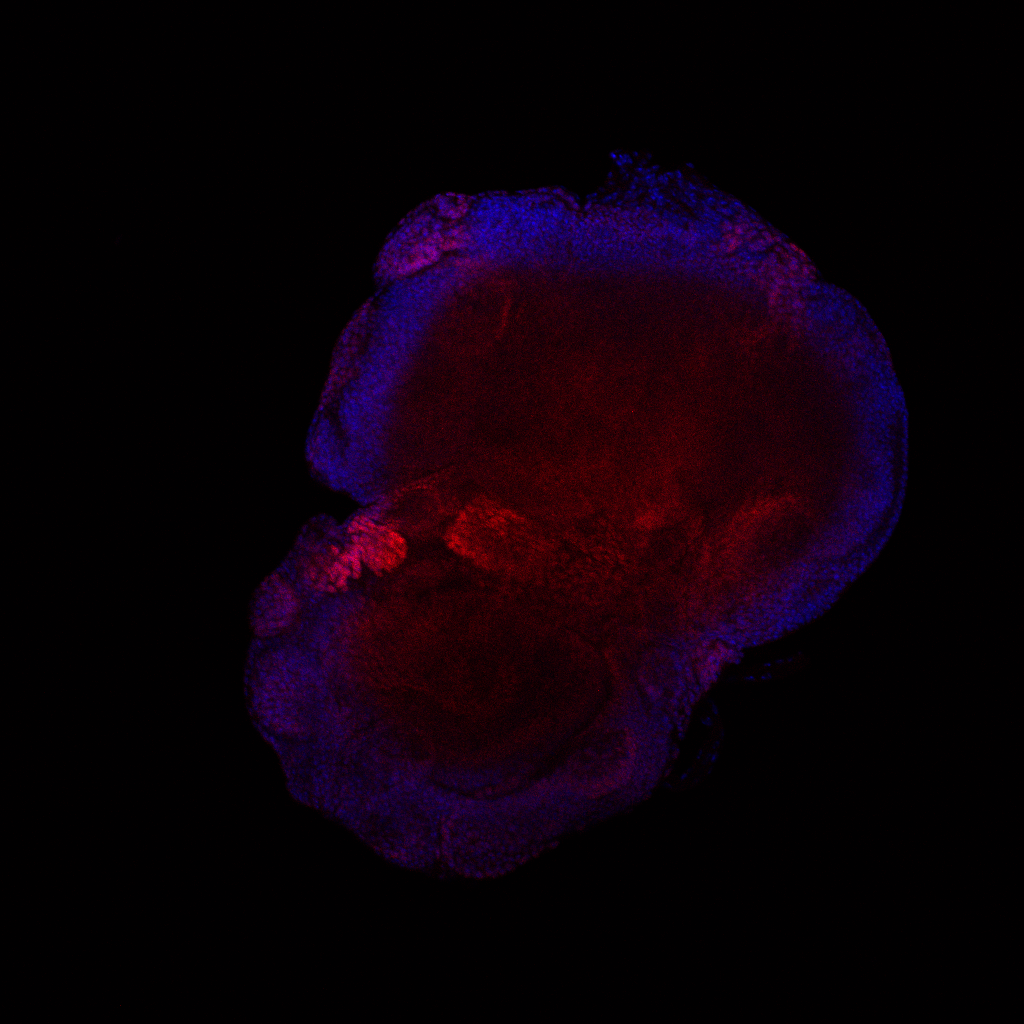

Supplement: Supplementary file 7 — Source data Fig. 3 [file 44318_2025_547_MOESM7_ESM.zip › Figure 3H/3-2 original image.tif]

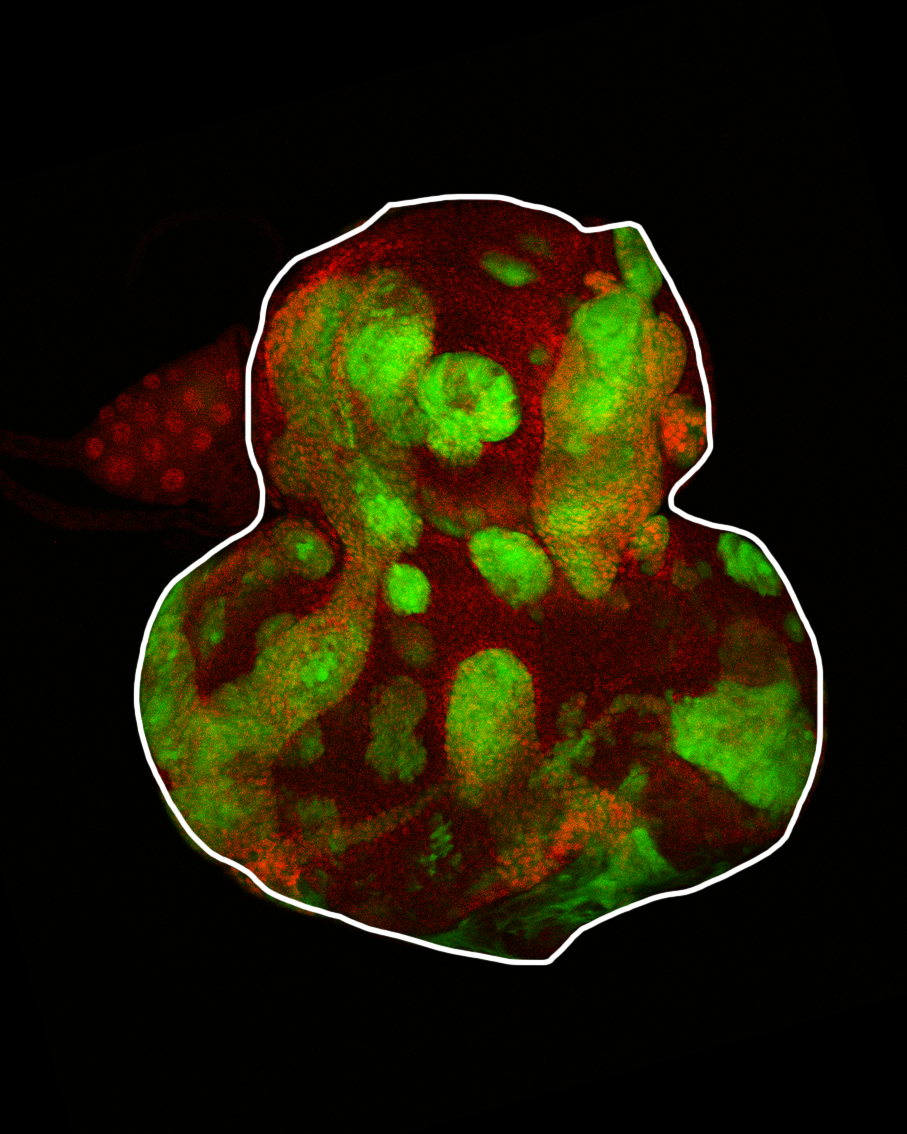

Supplement: Supplementary file 7 — Source data Fig. 3 [file 44318_2025_547_MOESM7_ESM.zip › Figure 3H/4-1 rotated and cut image with border line.tif]

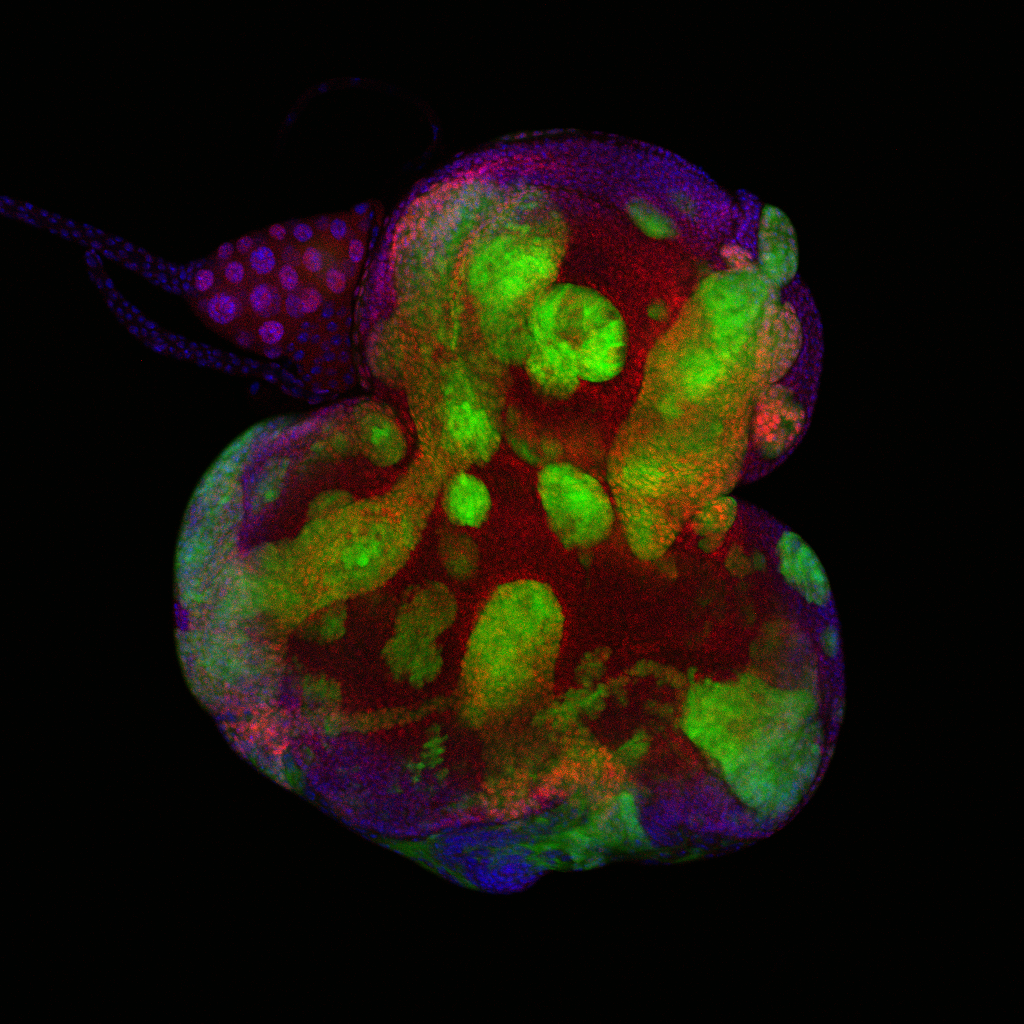

Supplement: Supplementary file 7 — Source data Fig. 3 [file 44318_2025_547_MOESM7_ESM.zip › Figure 3H/4-2 original image.tif]

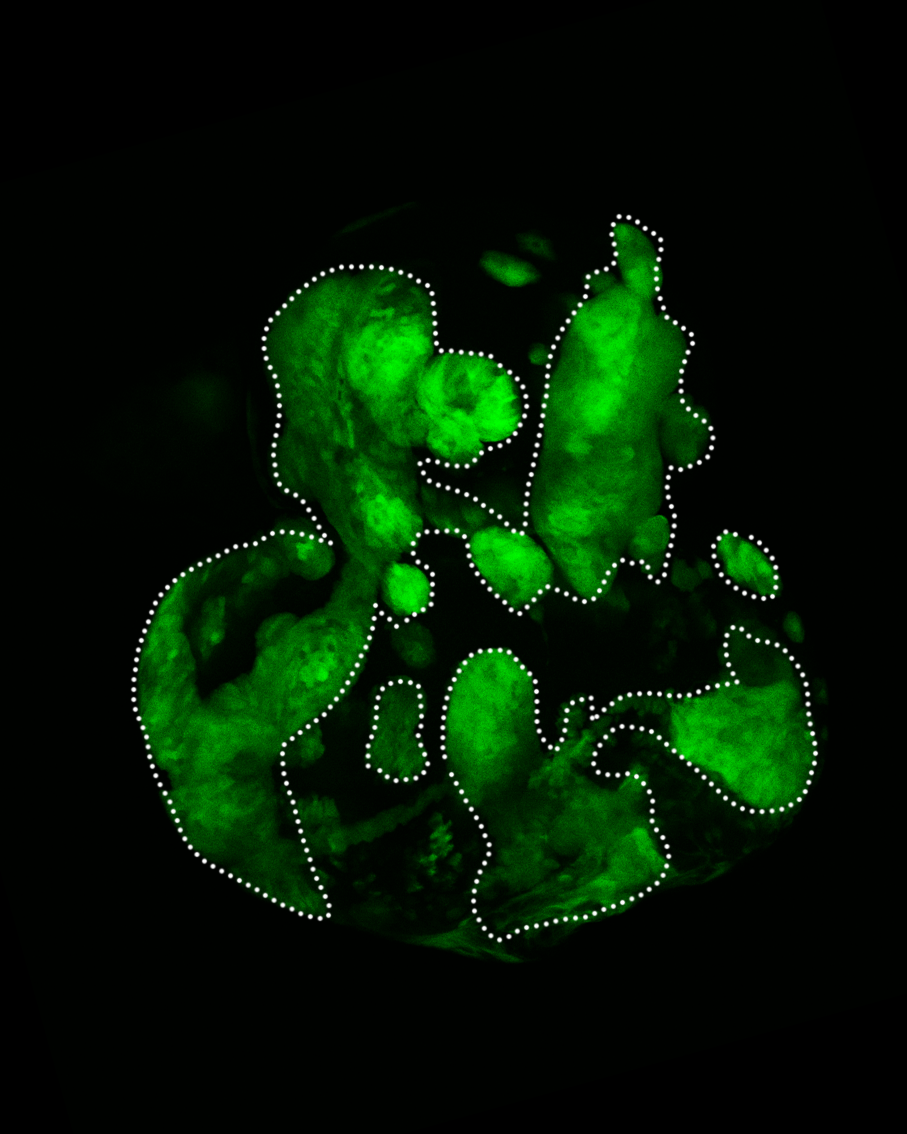

Supplement: Supplementary file 7 — Source data Fig. 3 [file 44318_2025_547_MOESM7_ESM.zip › Figure 3H/5-1 rotated and cut image with border line.tif]

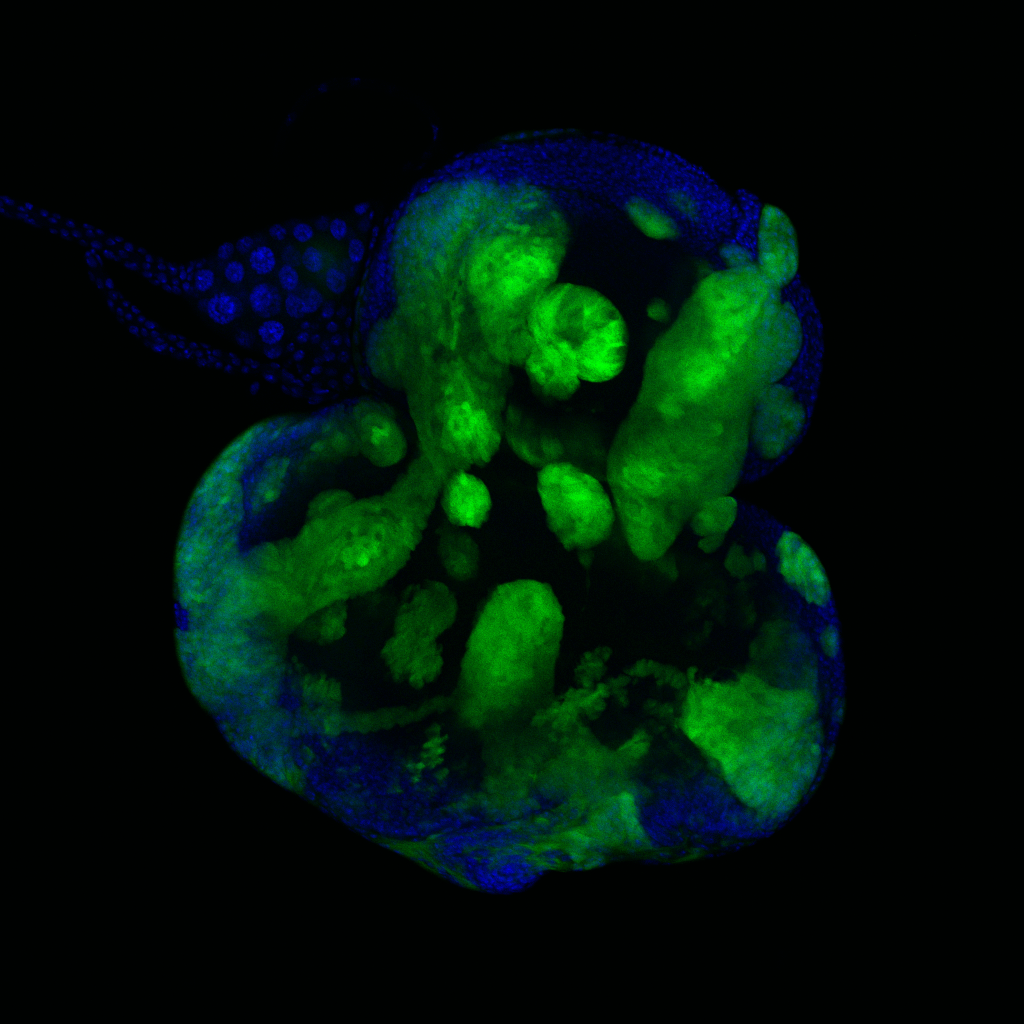

Supplement: Supplementary file 7 — Source data Fig. 3 [file 44318_2025_547_MOESM7_ESM.zip › Figure 3H/5-2 original image.tif]

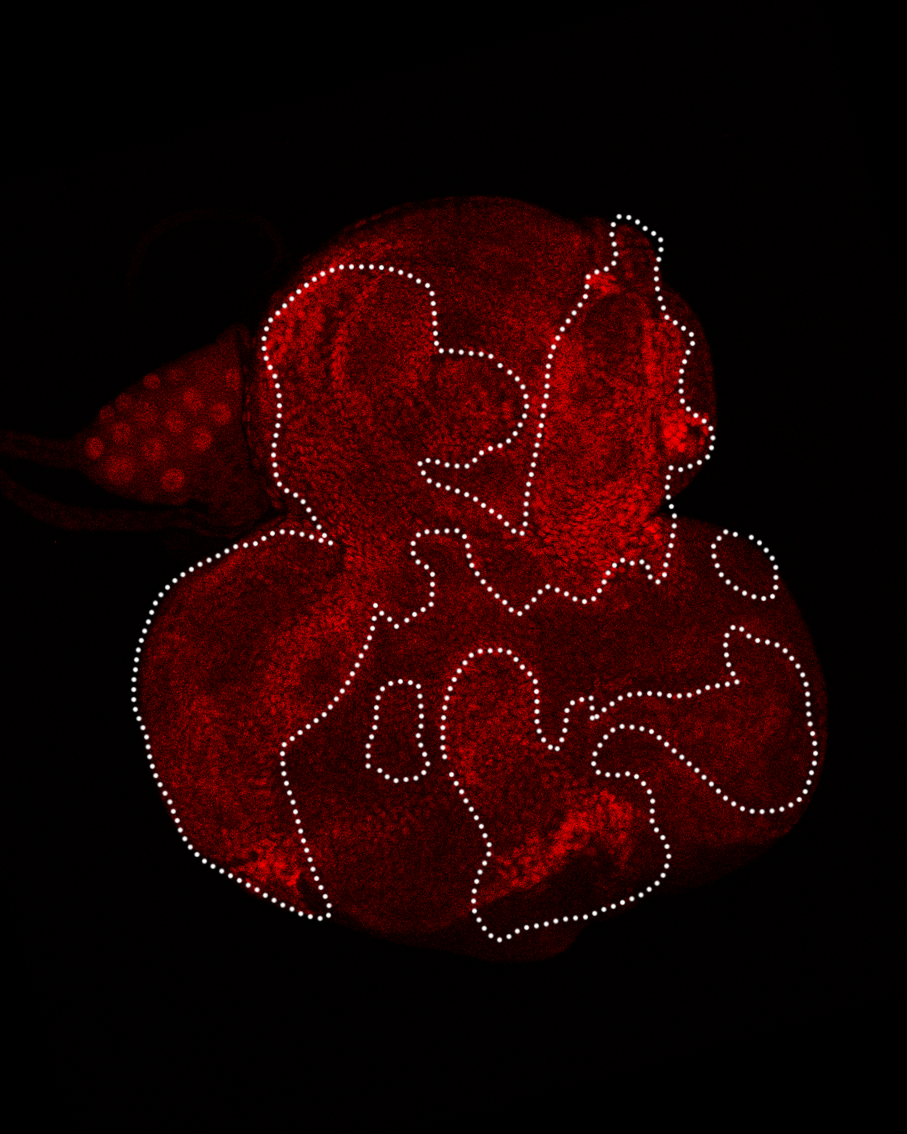

Supplement: Supplementary file 7 — Source data Fig. 3 [file 44318_2025_547_MOESM7_ESM.zip › Figure 3H/6-1 rotated and cut image with border line.tif]

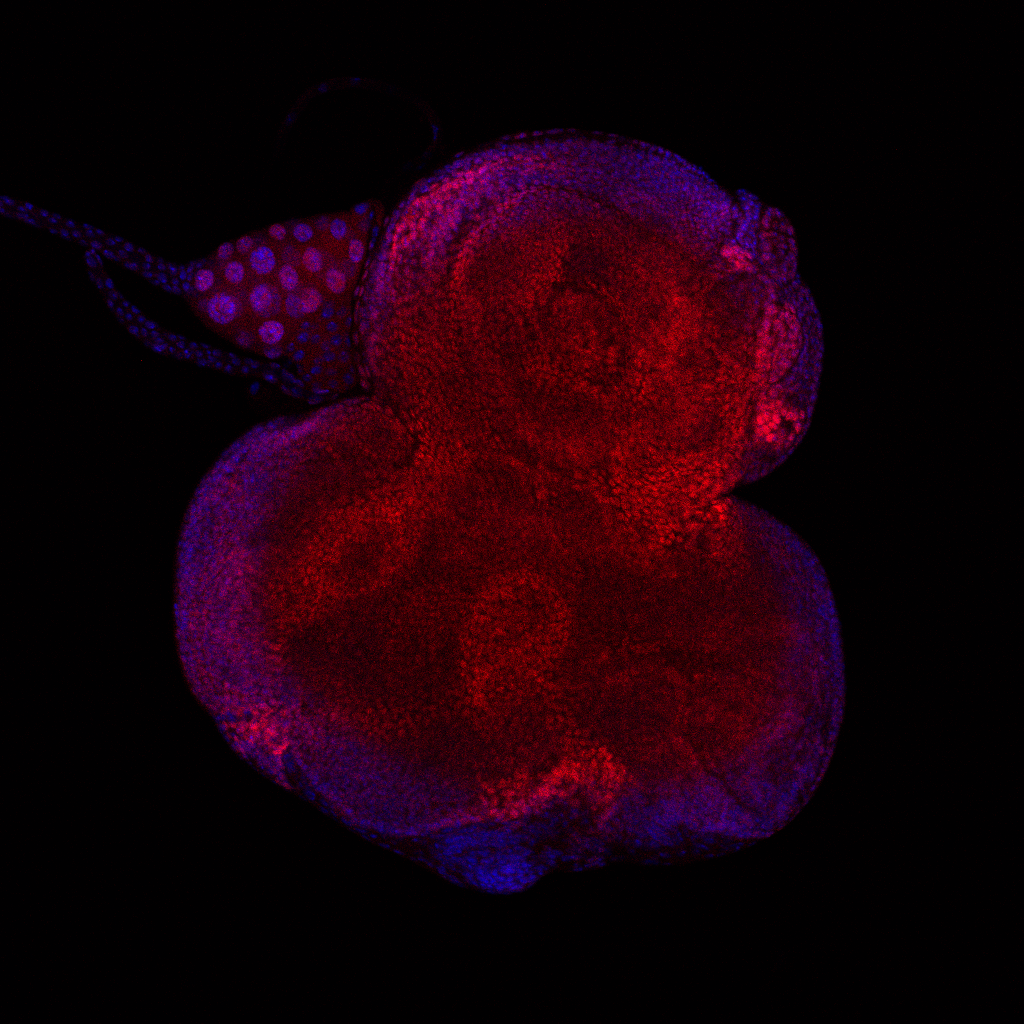

Supplement: Supplementary file 7 — Source data Fig. 3 [file 44318_2025_547_MOESM7_ESM.zip › Figure 3H/6-2 original image.tif]

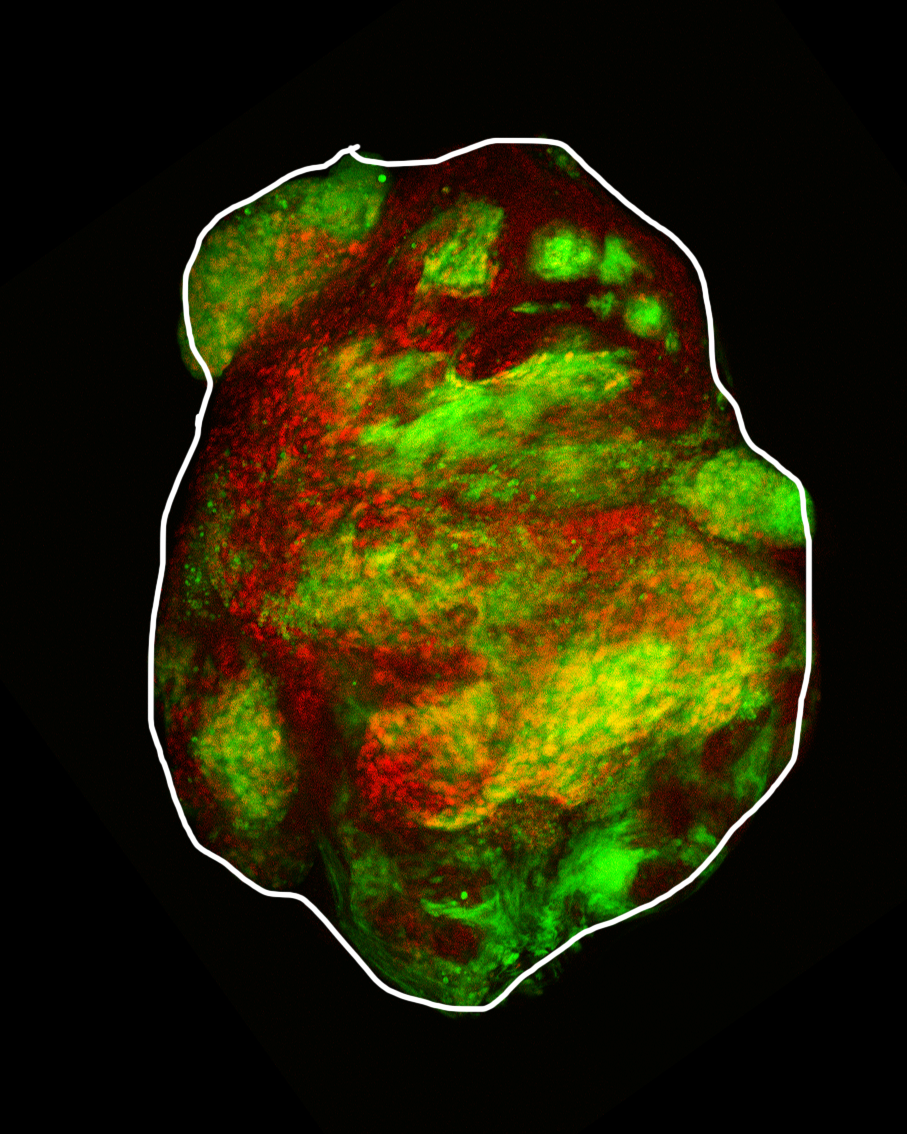

Supplement: Supplementary file 7 — Source data Fig. 3 [file 44318_2025_547_MOESM7_ESM.zip › Figure 3H/7-1 rotated and cut image with border line.tif]

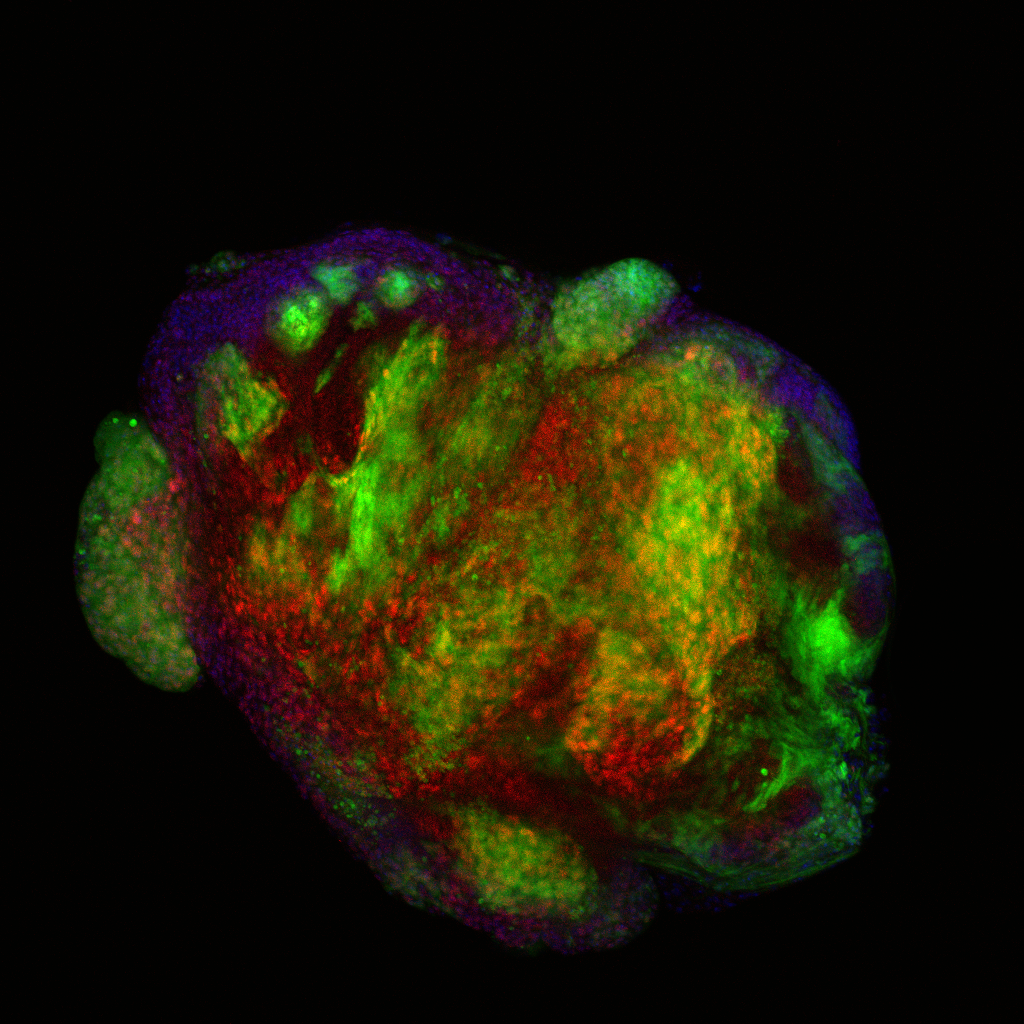

Supplement: Supplementary file 7 — Source data Fig. 3 [file 44318_2025_547_MOESM7_ESM.zip › Figure 3H/7-2 original image.tif]

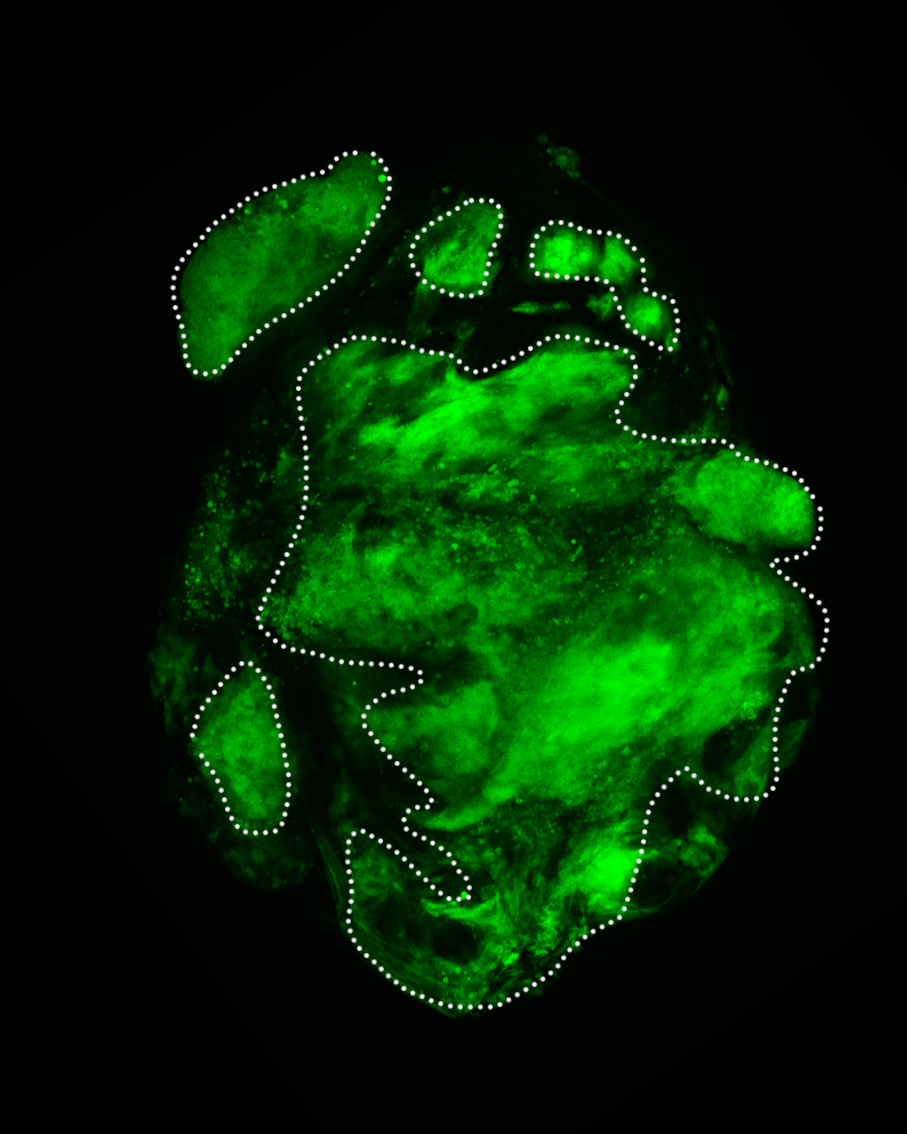

Supplement: Supplementary file 7 — Source data Fig. 3 [file 44318_2025_547_MOESM7_ESM.zip › Figure 3H/8-1 rotated and cut image with border line.tif]

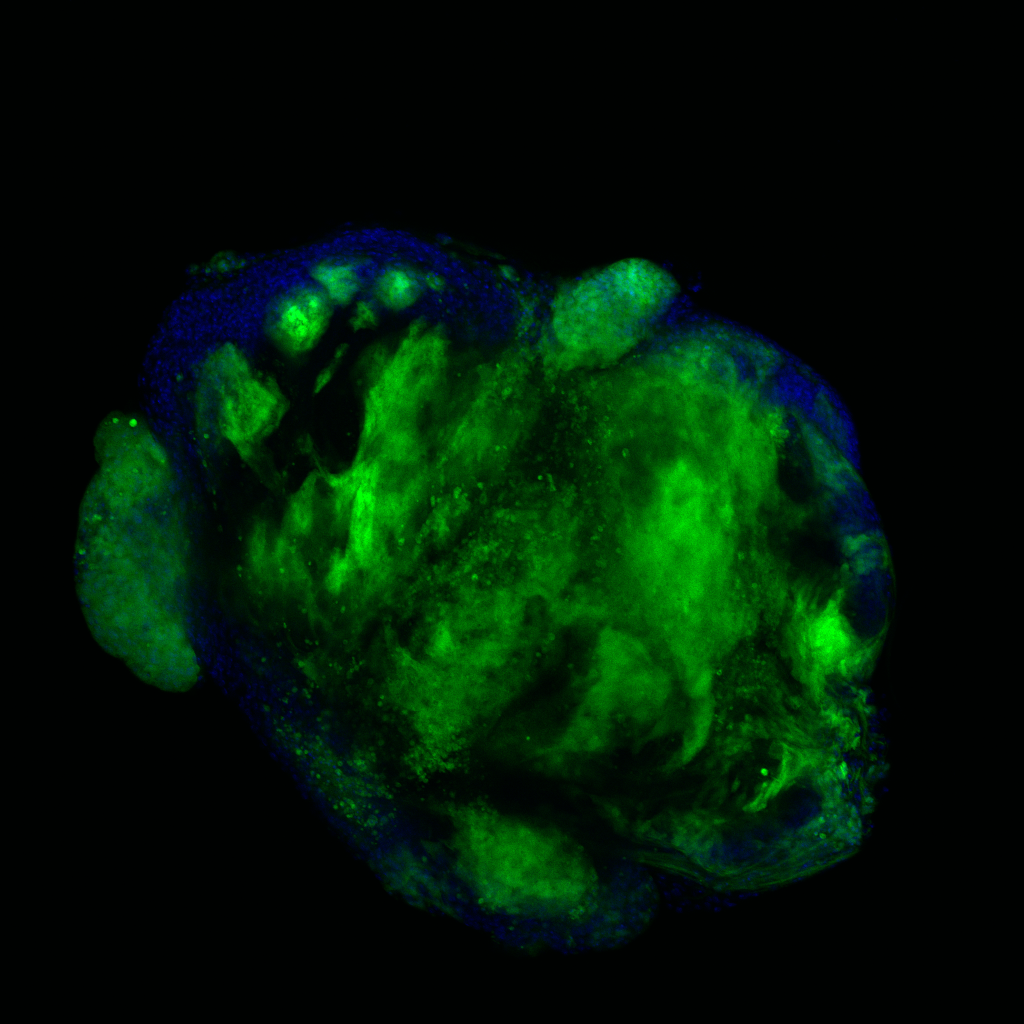

Supplement: Supplementary file 7 — Source data Fig. 3 [file 44318_2025_547_MOESM7_ESM.zip › Figure 3H/8-2 original image.tif]

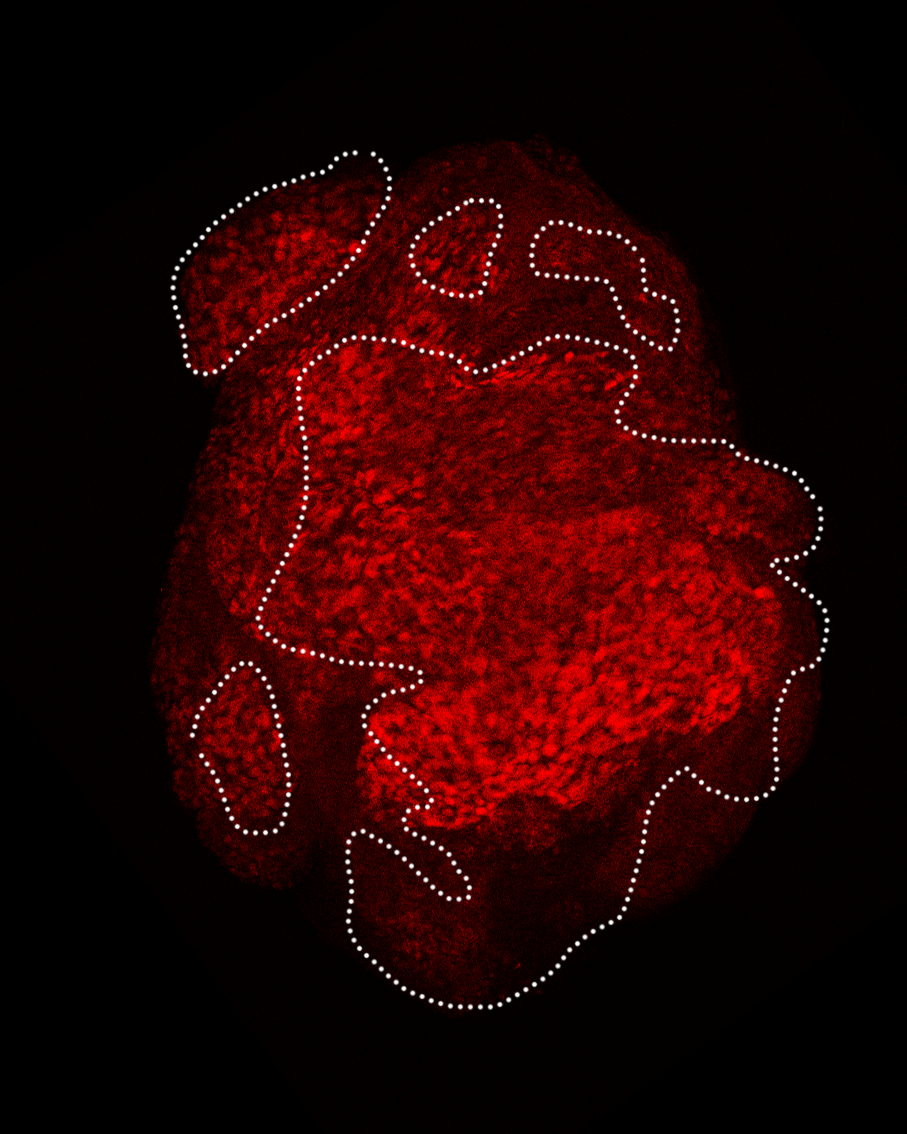

Supplement: Supplementary file 7 — Source data Fig. 3 [file 44318_2025_547_MOESM7_ESM.zip › Figure 3H/9-1 rotated and cut image with border line.tif]

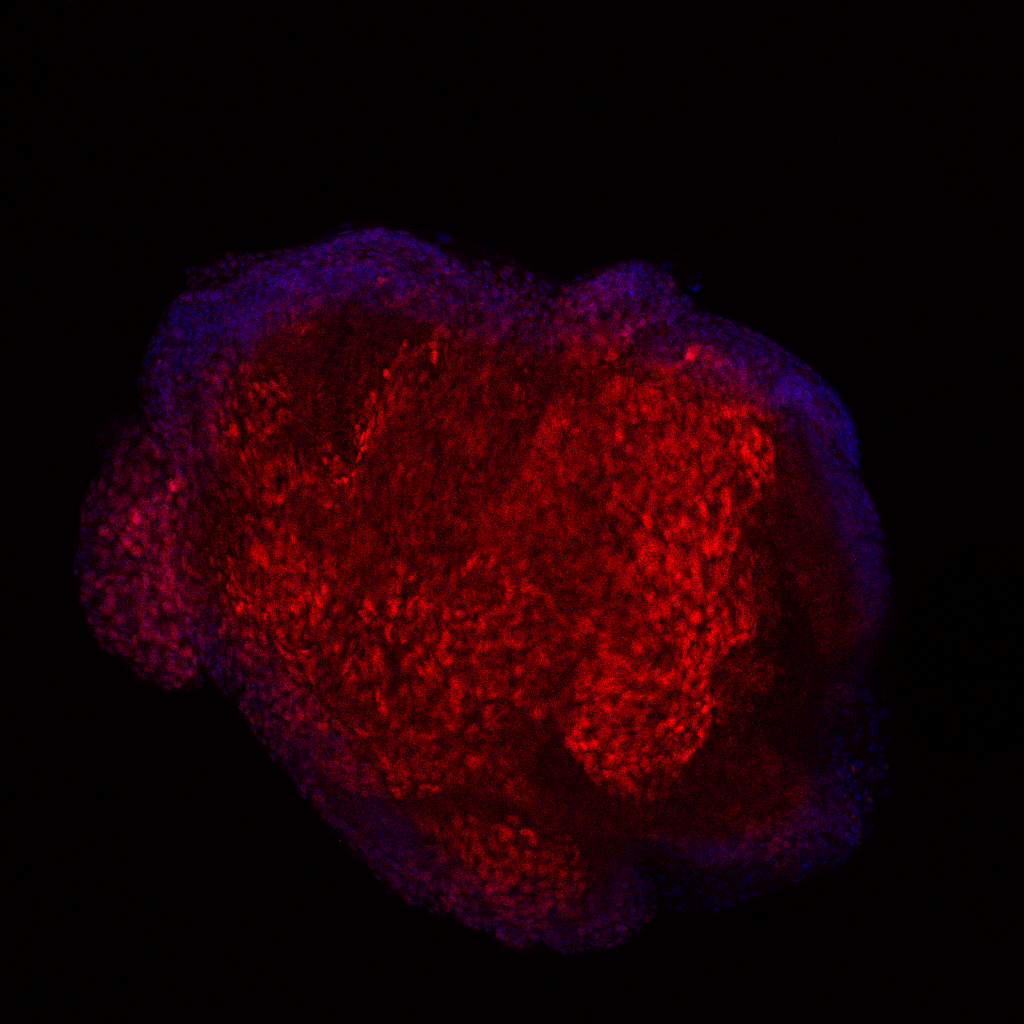

Supplement: Supplementary file 7 — Source data Fig. 3 [file 44318_2025_547_MOESM7_ESM.zip › Figure 3H/9-2 original image.tif]

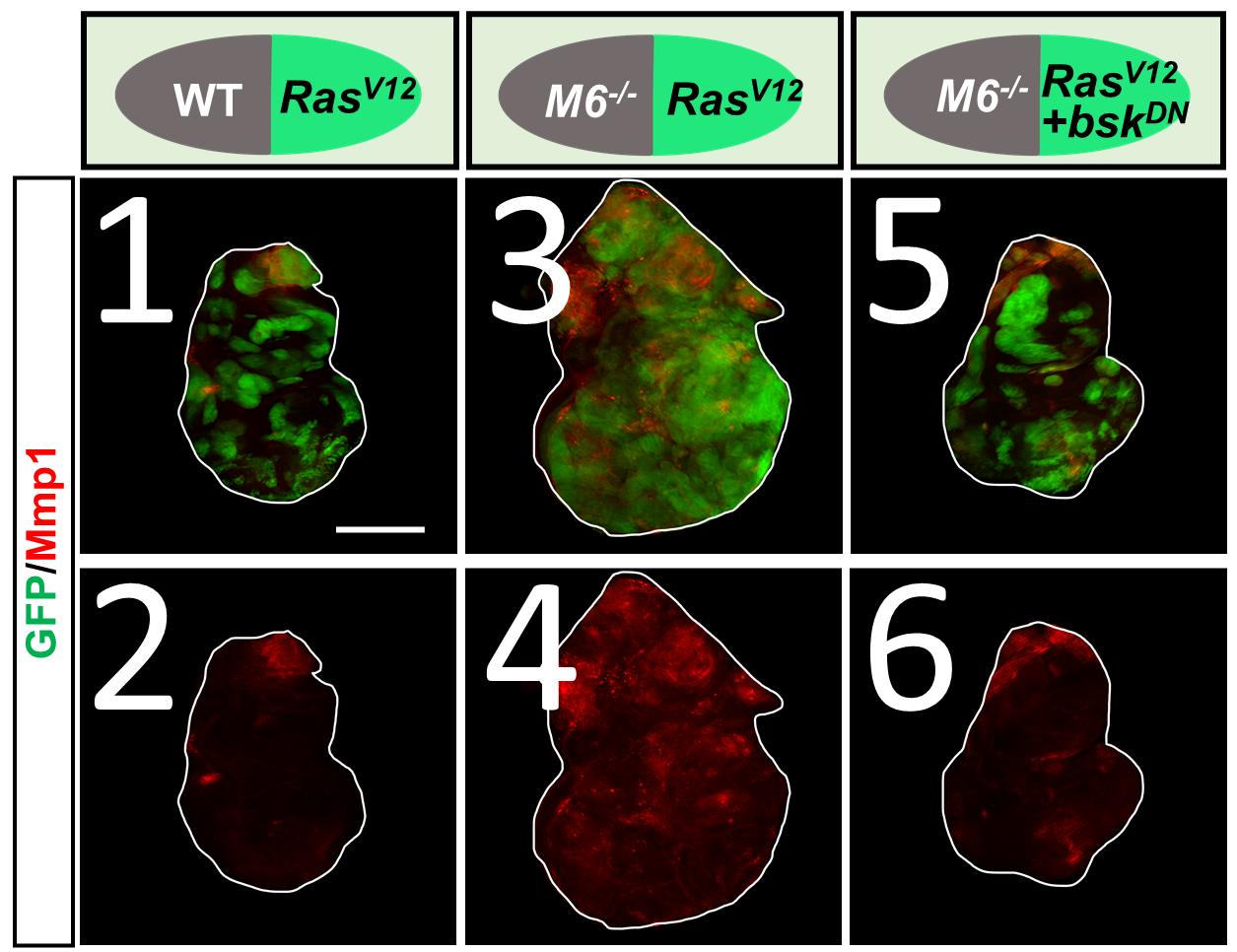

Supplement: Supplementary file 8 — Source data Fig. 4 [file 44318_2025_547_MOESM8_ESM.zip › Figure 4H/0 paper Figure 4H with provided image sequence.tif]

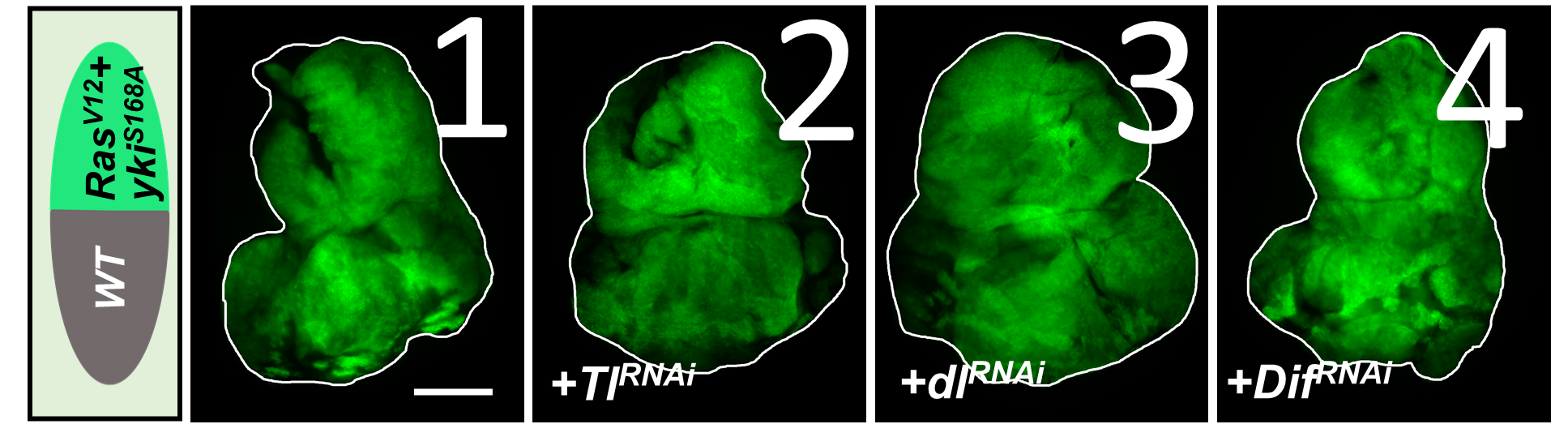

Supplement: Supplementary file 8 — Source data Fig. 4 [file 44318_2025_547_MOESM8_ESM.zip › Figure 4F/0 paper Figure 4F with provided image sequence.tif]

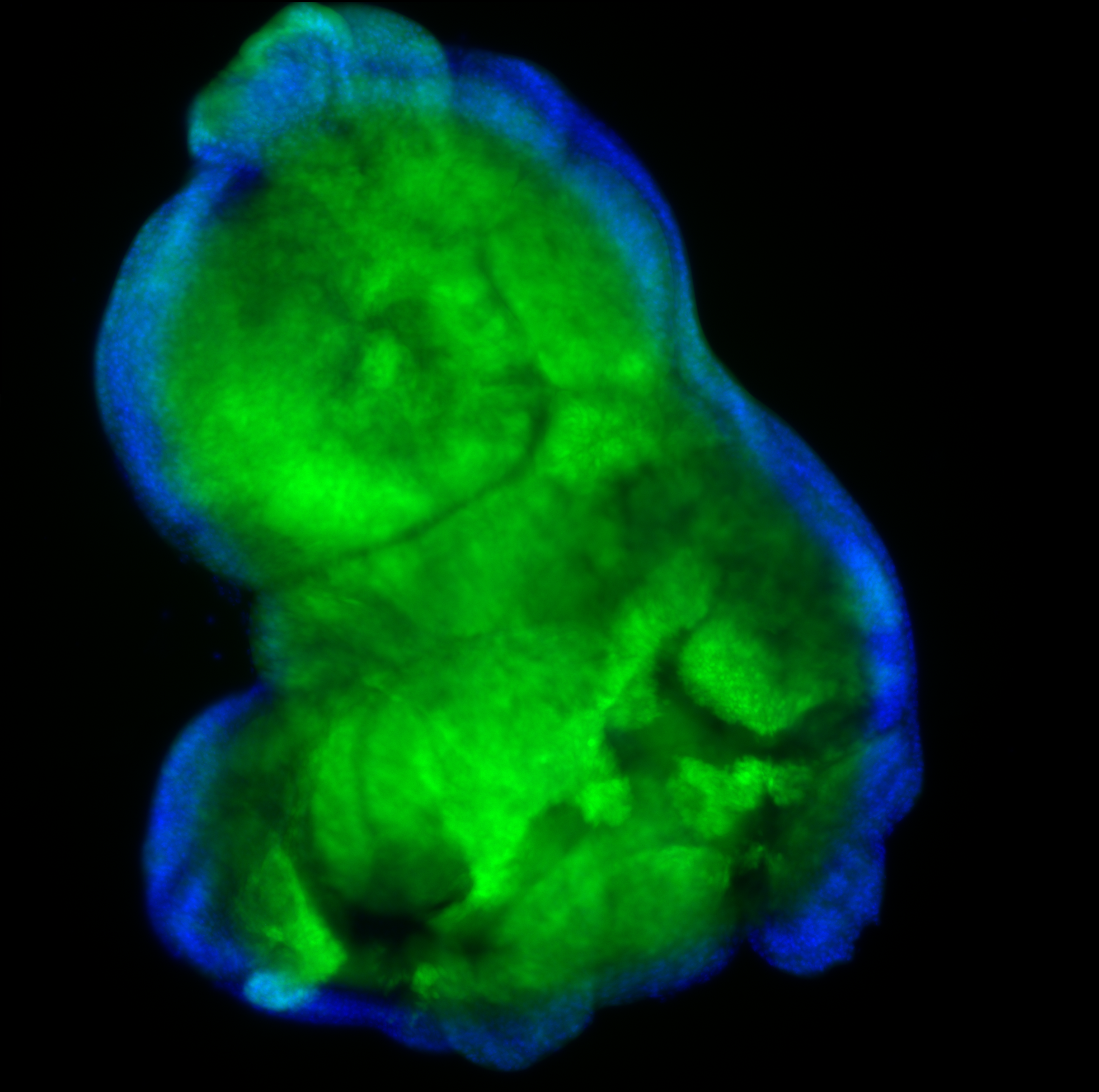

Supplement: Supplementary file 8 — Source data Fig. 4 [file 44318_2025_547_MOESM8_ESM.zip › Figure 4F/4-2 original image.tif]

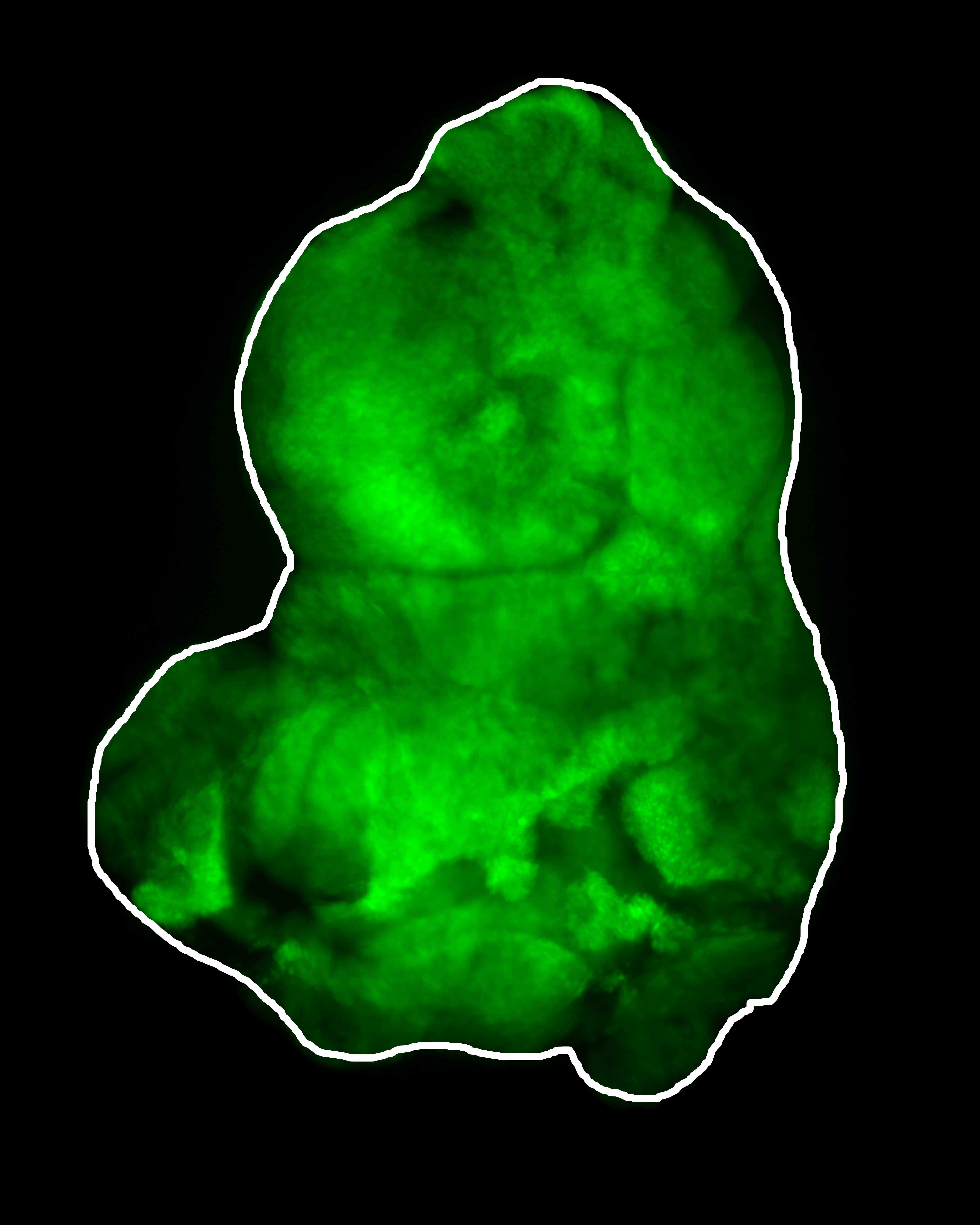

Supplement: Supplementary file 8 — Source data Fig. 4 [file 44318_2025_547_MOESM8_ESM.zip › Figure 4F/4-1 rotated and cut image with border line.tif]

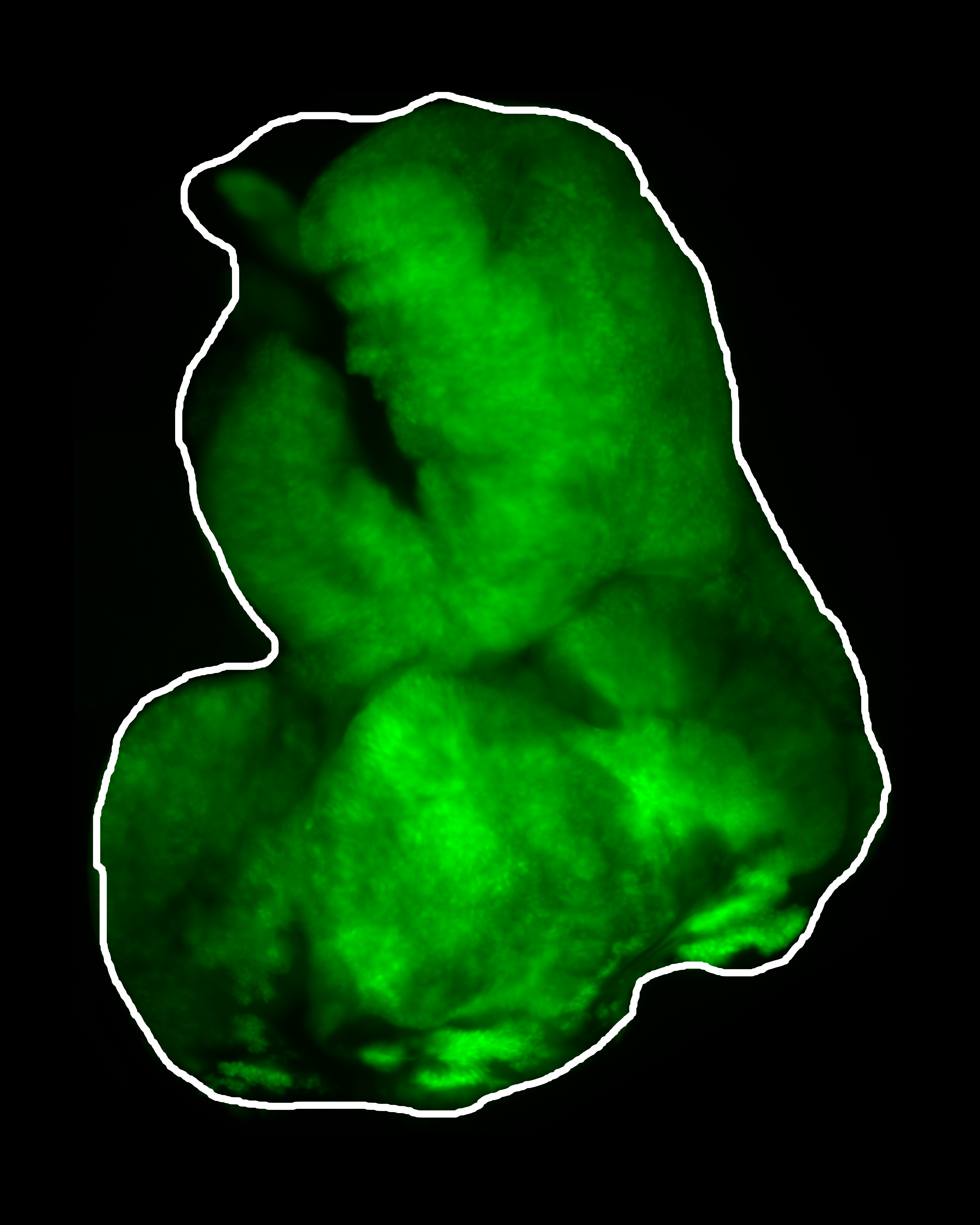

Supplement: Supplementary file 8 — Source data Fig. 4 [file 44318_2025_547_MOESM8_ESM.zip › Figure 4F/1-1 rotated and cut image with border line.tif]

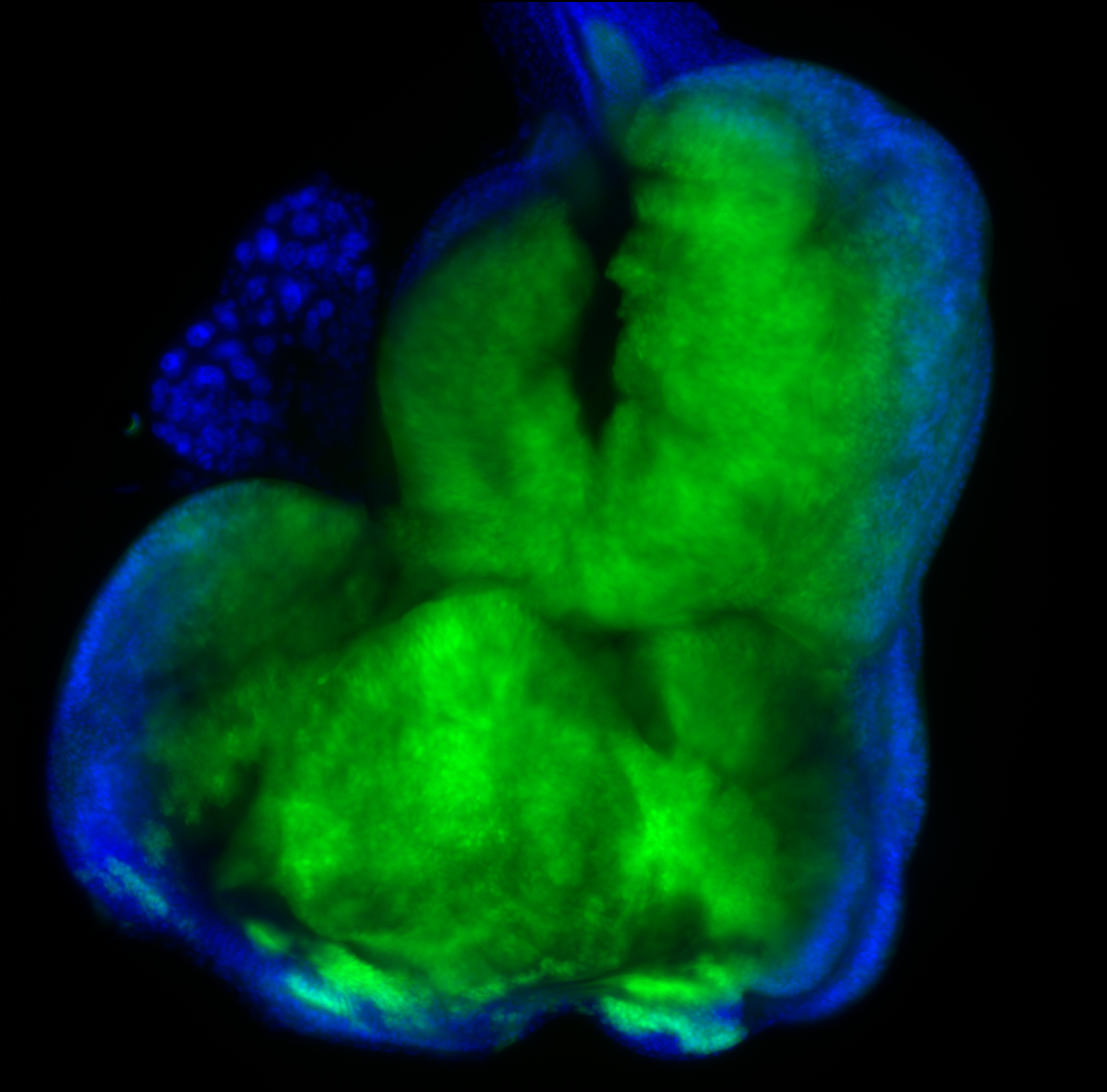

Supplement: Supplementary file 8 — Source data Fig. 4 [file 44318_2025_547_MOESM8_ESM.zip › Figure 4F/1-2 original image.tif]

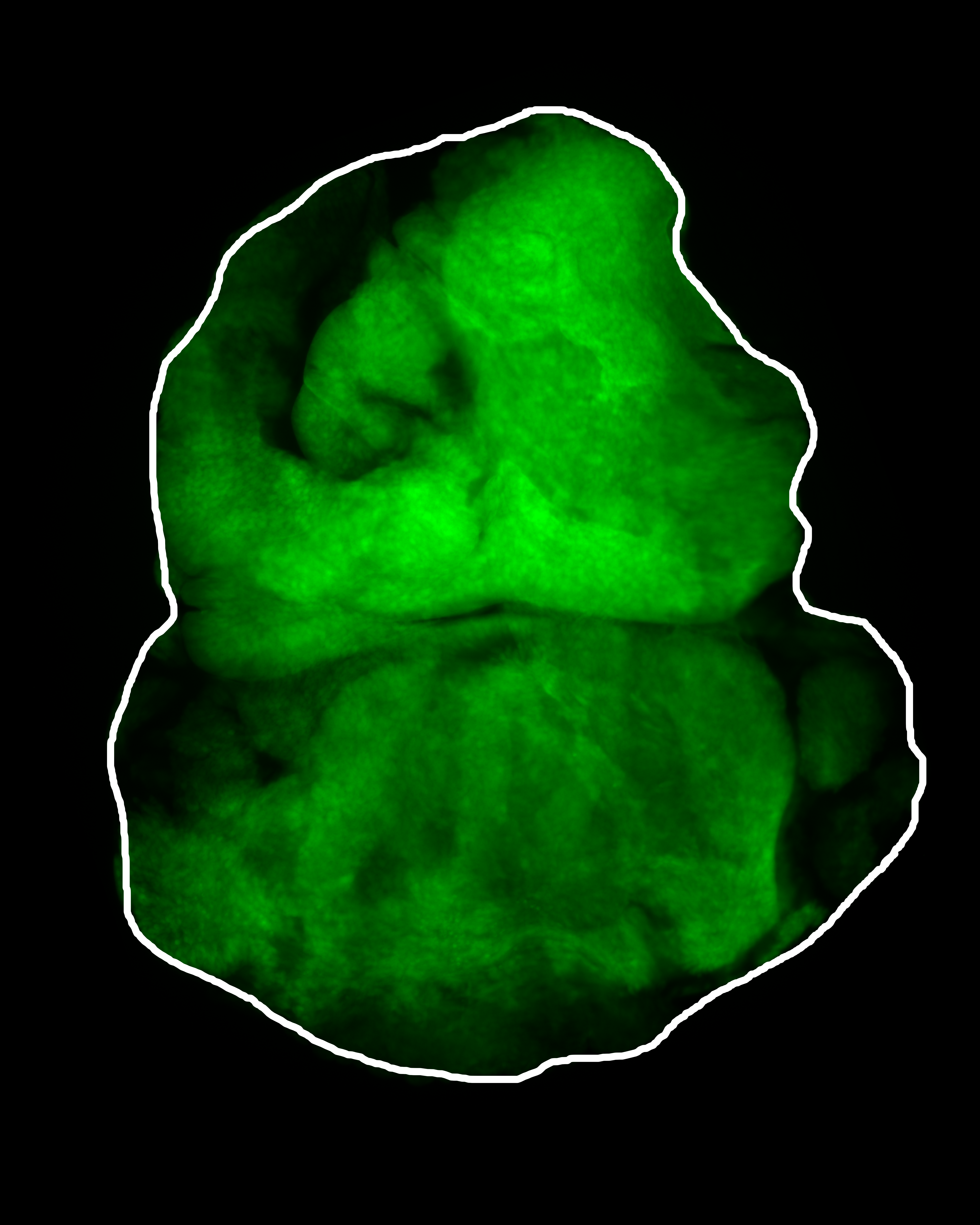

Supplement: Supplementary file 8 — Source data Fig. 4 [file 44318_2025_547_MOESM8_ESM.zip › Figure 4F/2-1 rotated and cut image with border line.tif]

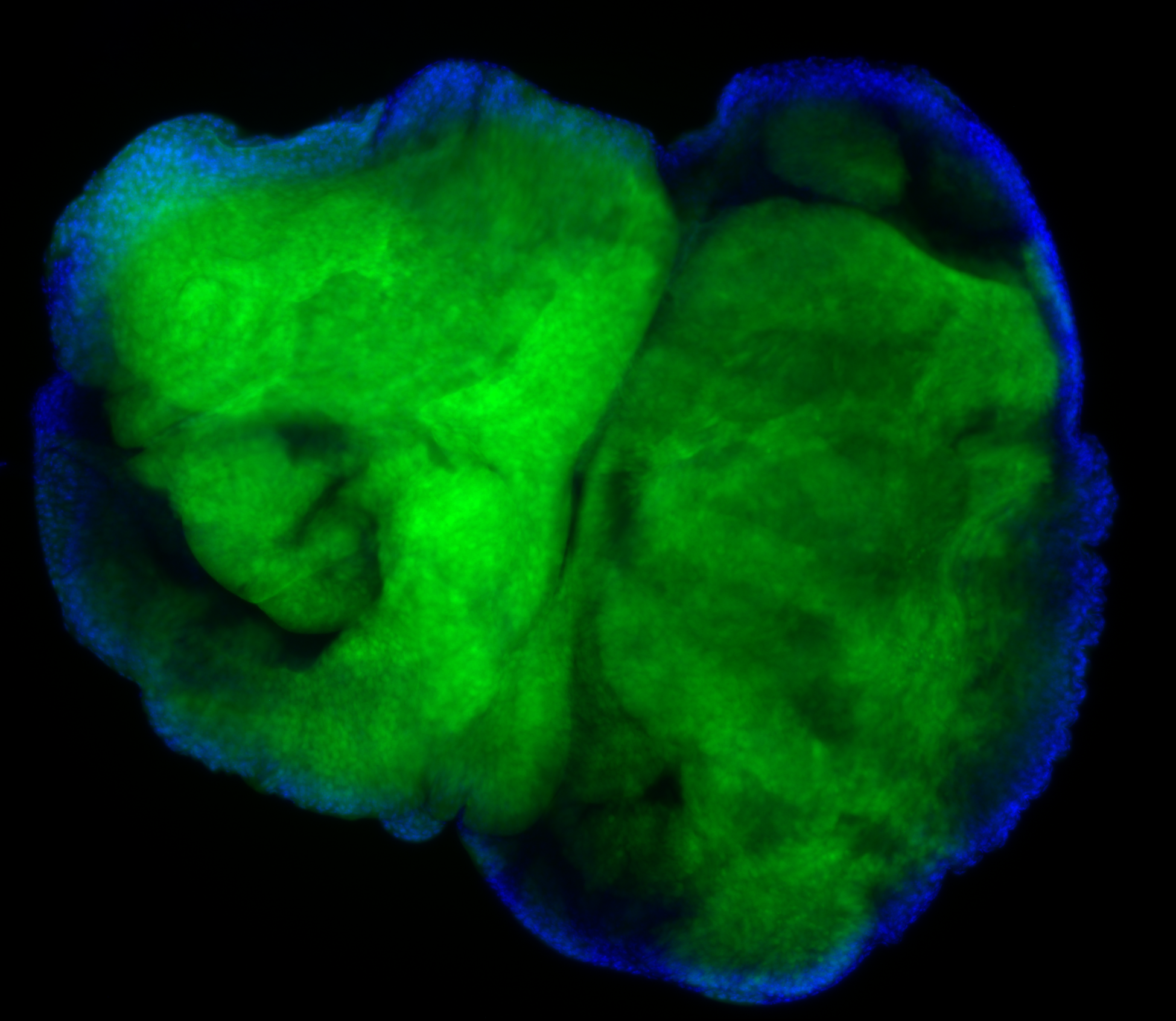

Supplement: Supplementary file 8 — Source data Fig. 4 [file 44318_2025_547_MOESM8_ESM.zip › Figure 4F/2-2 original image.tif]

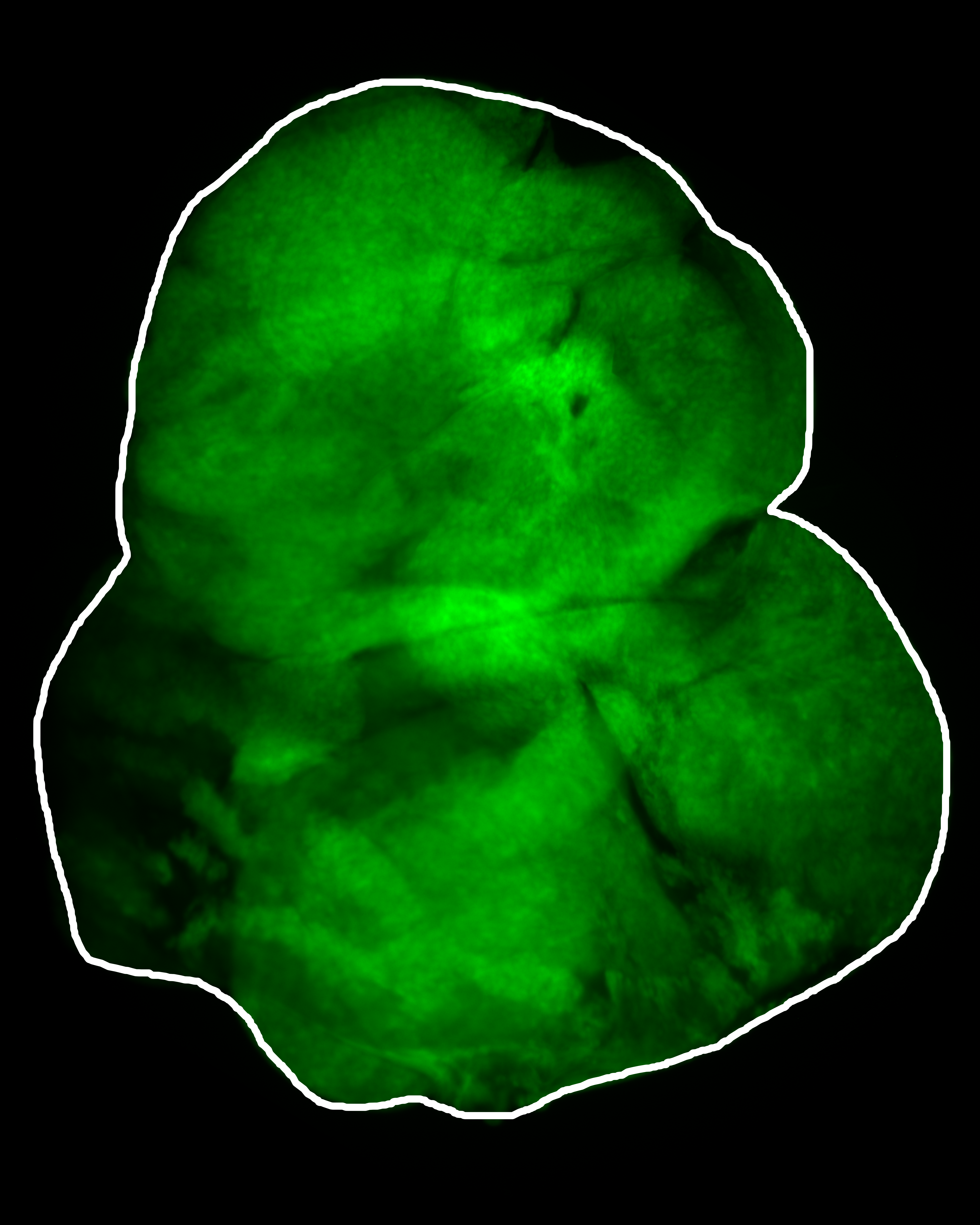

Supplement: Supplementary file 8 — Source data Fig. 4 [file 44318_2025_547_MOESM8_ESM.zip › Figure 4F/3-1 rotated and cut image with border line.tif]

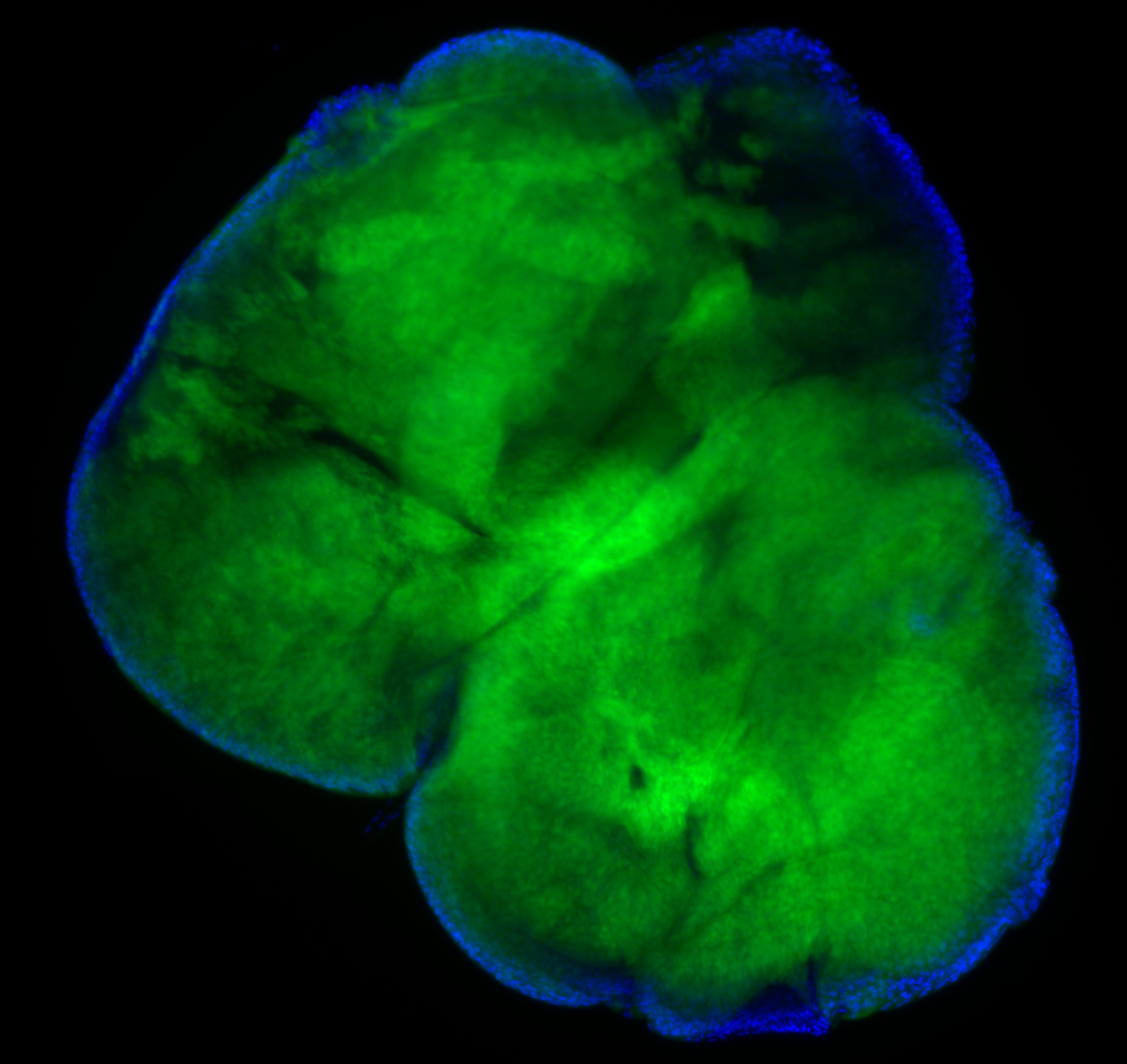

Supplement: Supplementary file 8 — Source data Fig. 4 [file 44318_2025_547_MOESM8_ESM.zip › Figure 4F/3-2 original image.tif]

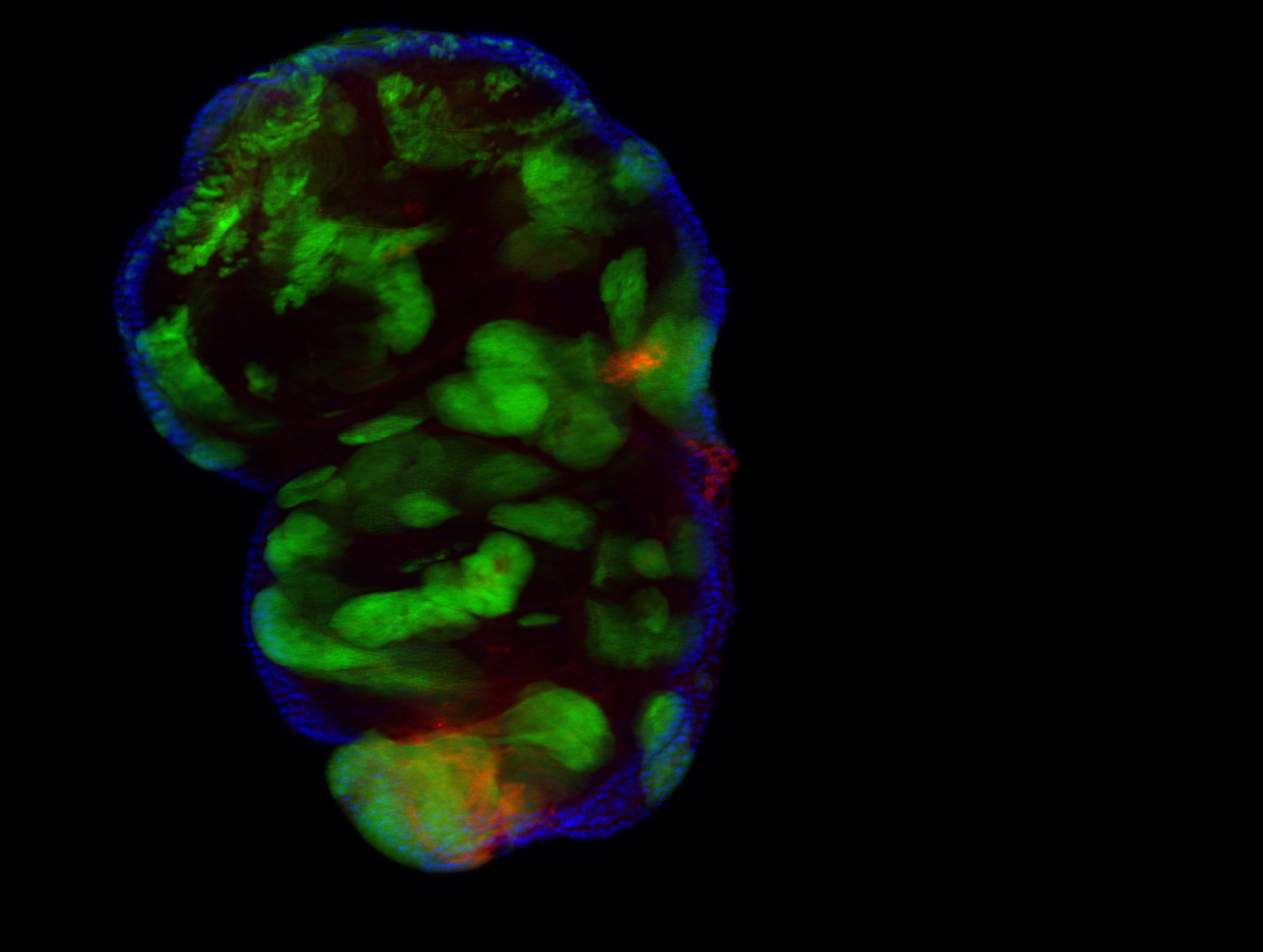

Supplement: Supplementary file 8 — Source data Fig. 4 [file 44318_2025_547_MOESM8_ESM.zip › Figure 4H/1 original image.tif]

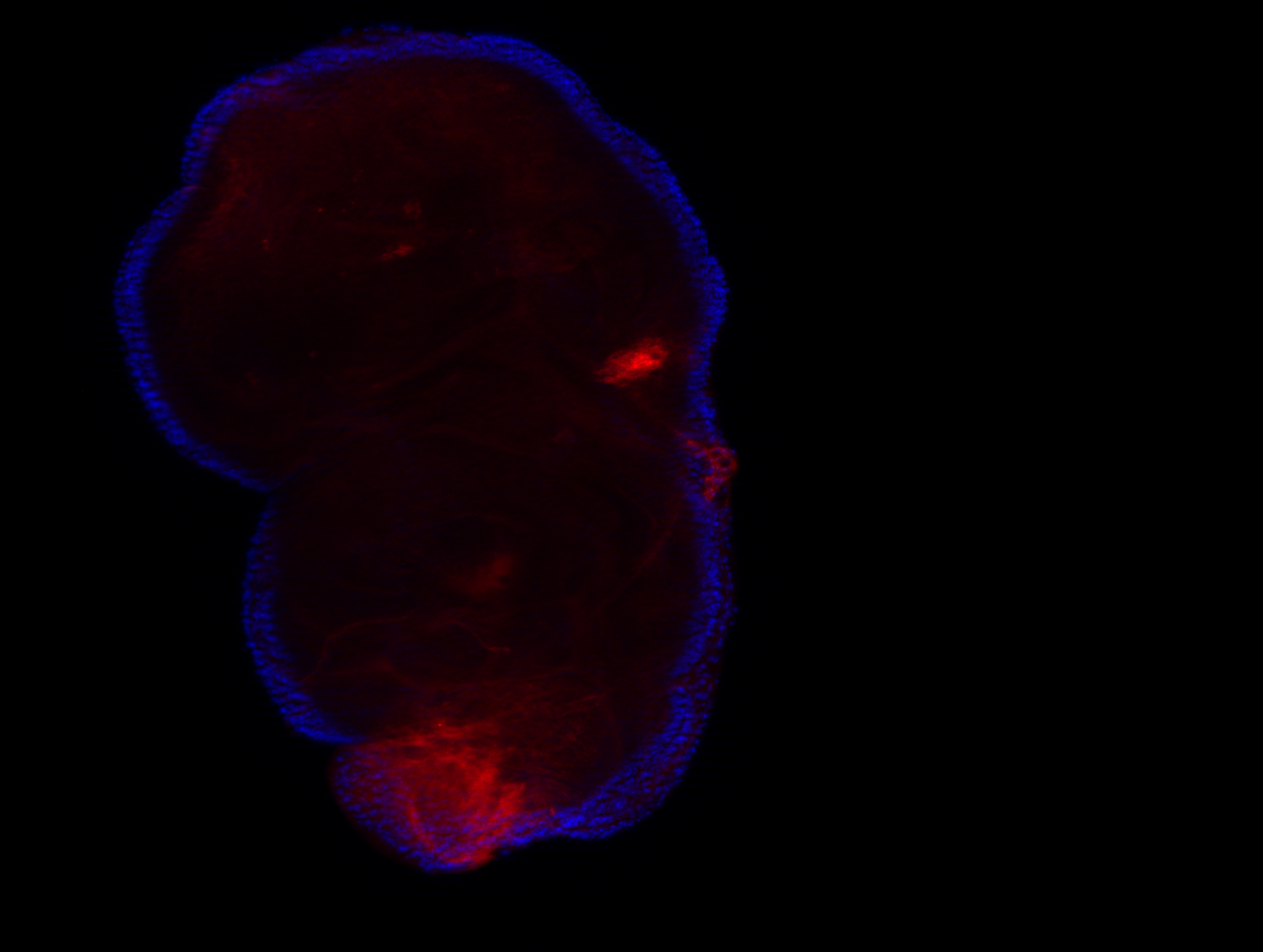

Supplement: Supplementary file 8 — Source data Fig. 4 [file 44318_2025_547_MOESM8_ESM.zip › Figure 4H/2 original image.tif]

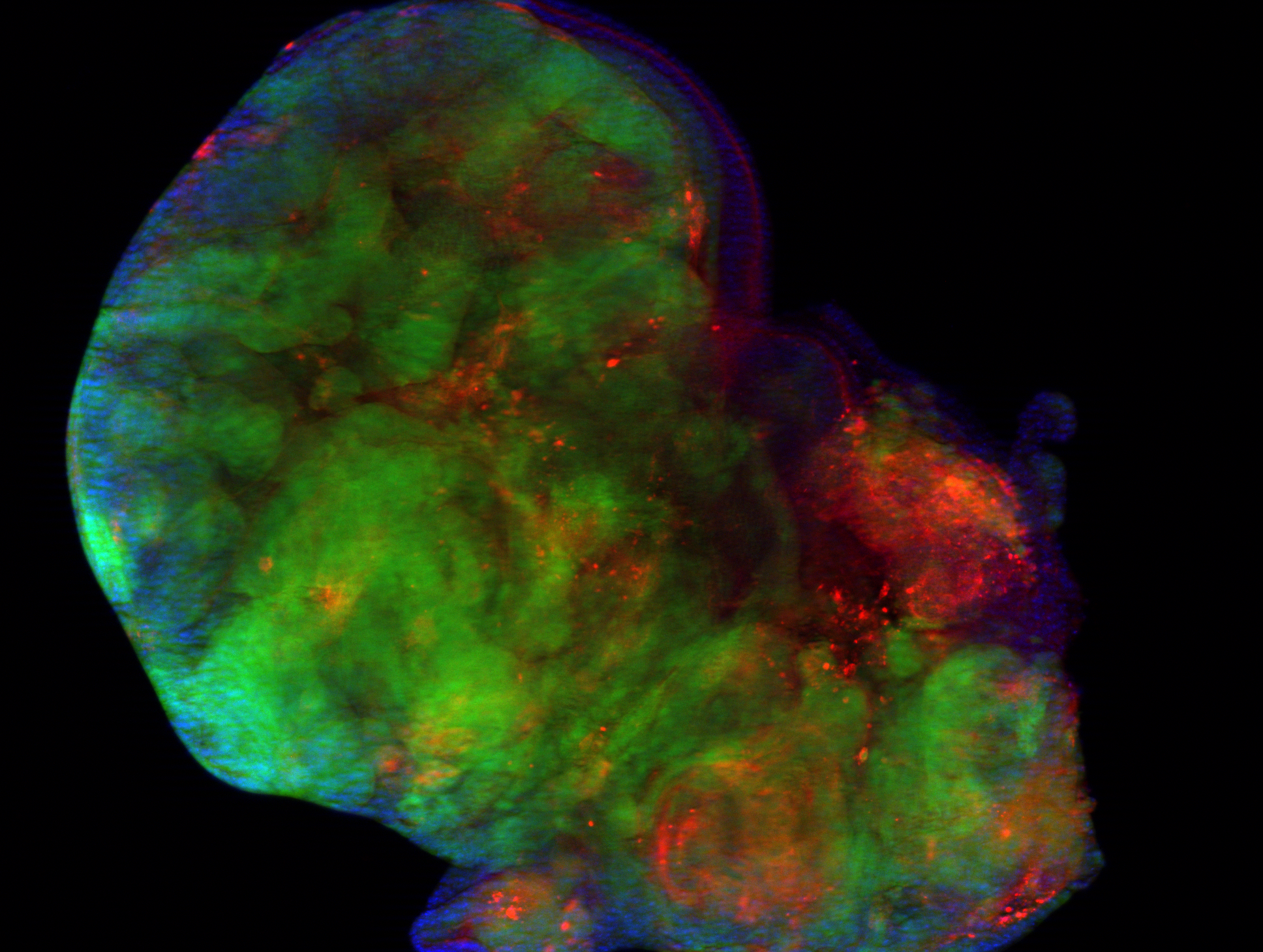

Supplement: Supplementary file 8 — Source data Fig. 4 [file 44318_2025_547_MOESM8_ESM.zip › Figure 4H/3 original image.tif]

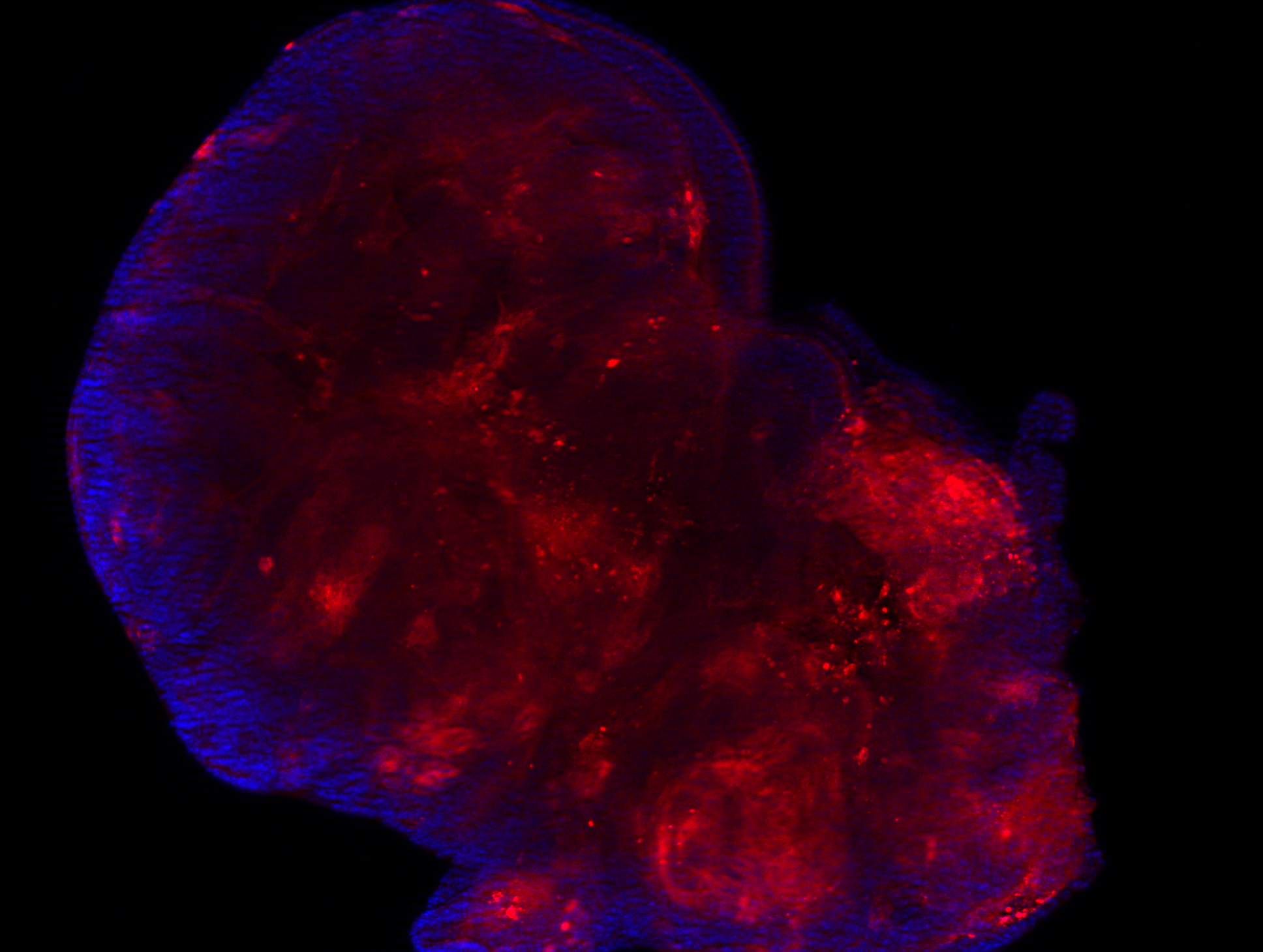

Supplement: Supplementary file 8 — Source data Fig. 4 [file 44318_2025_547_MOESM8_ESM.zip › Figure 4H/4 original image.tif]

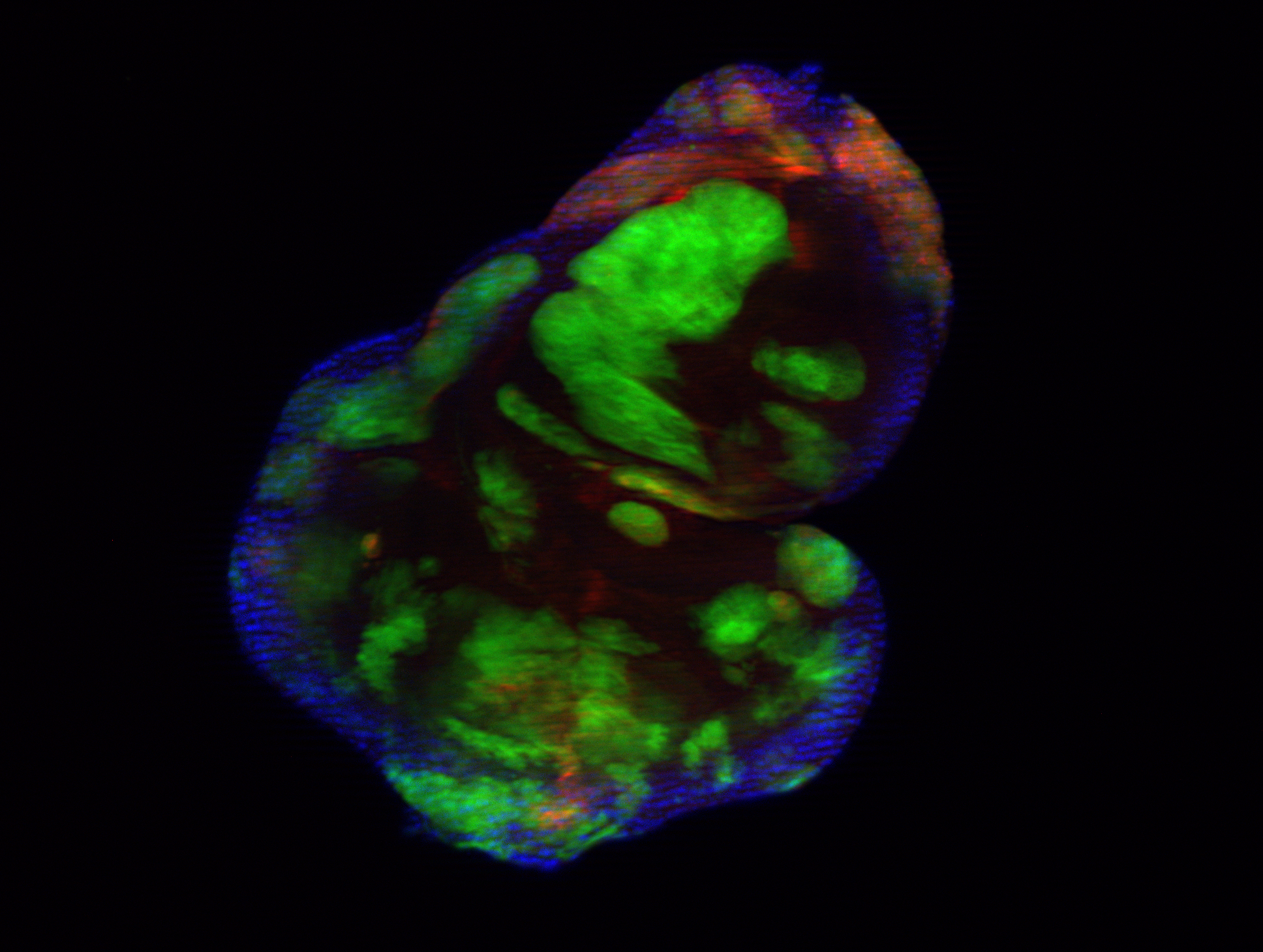

Supplement: Supplementary file 8 — Source data Fig. 4 [file 44318_2025_547_MOESM8_ESM.zip › Figure 4H/5 original image.tif]

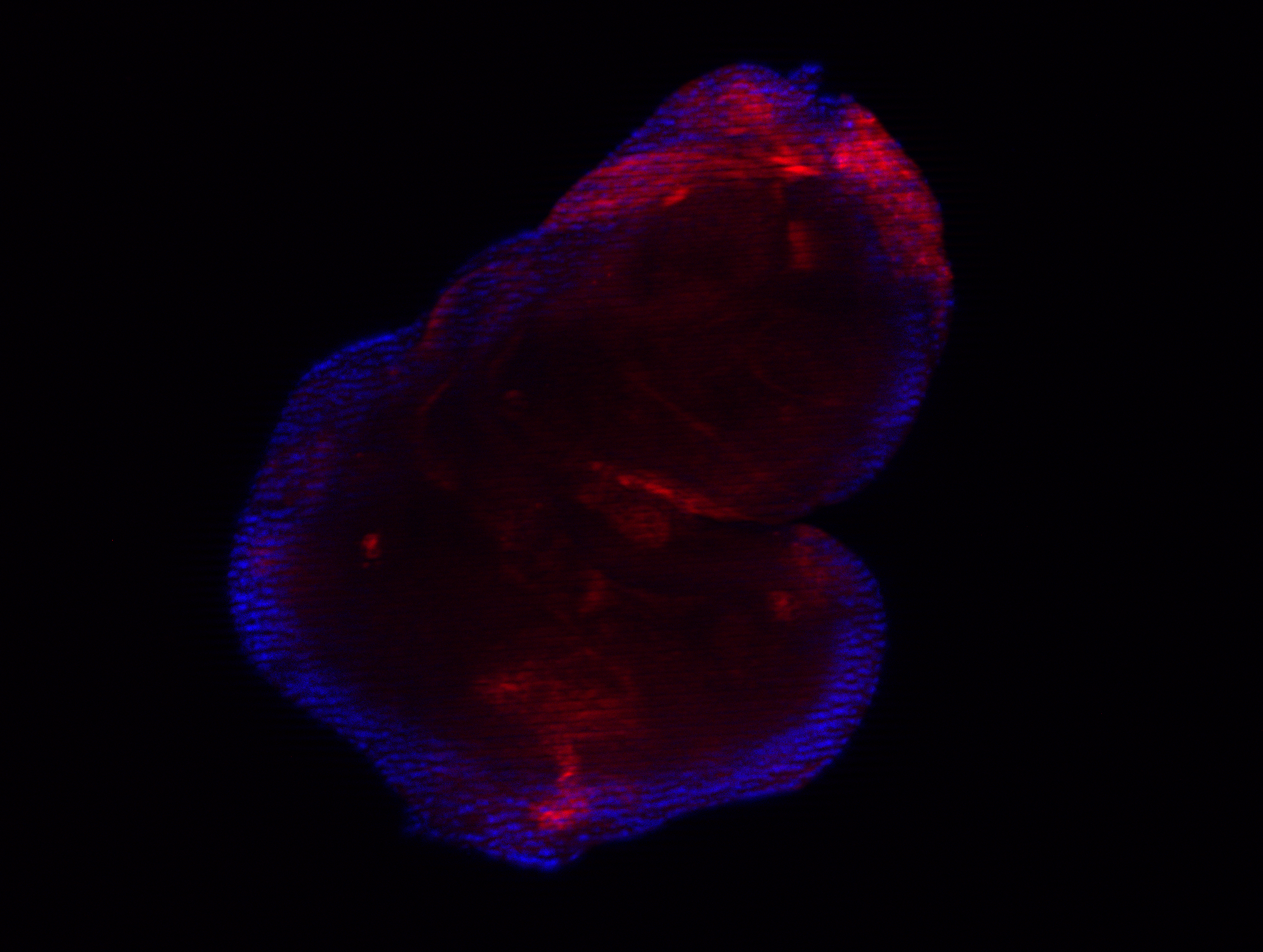

Supplement: Supplementary file 8 — Source data Fig. 4 [file 44318_2025_547_MOESM8_ESM.zip › Figure 4H/6 original image.tif]

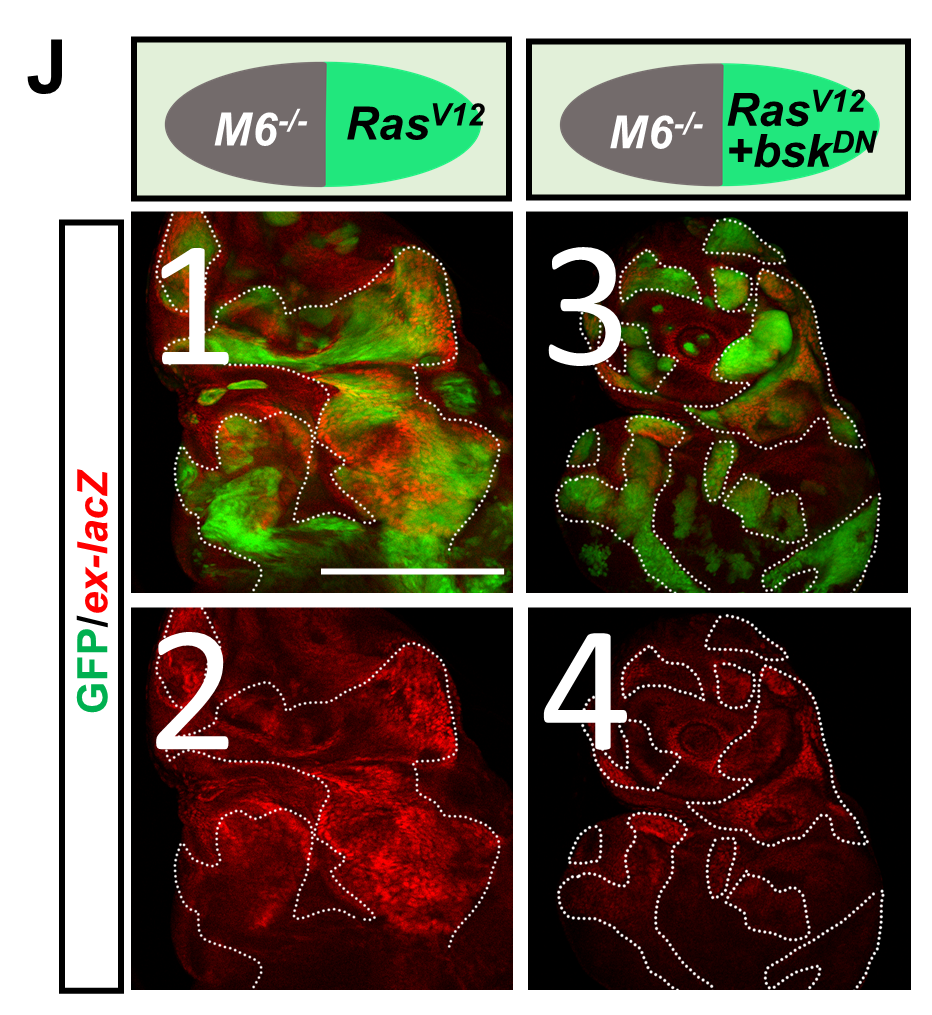

Supplement: Supplementary file 8 — Source data Fig. 4 [file 44318_2025_547_MOESM8_ESM.zip › Figure 4J/0 paper Figure 4J with provided image sequence.tif]

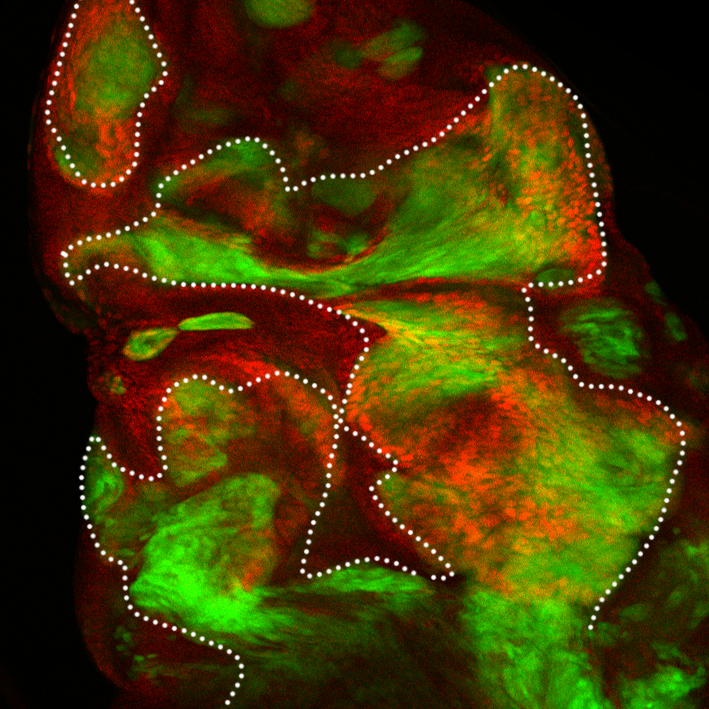

Supplement: Supplementary file 8 — Source data Fig. 4 [file 44318_2025_547_MOESM8_ESM.zip › Figure 4J/1-1 rotated and cut image with border line.tif]

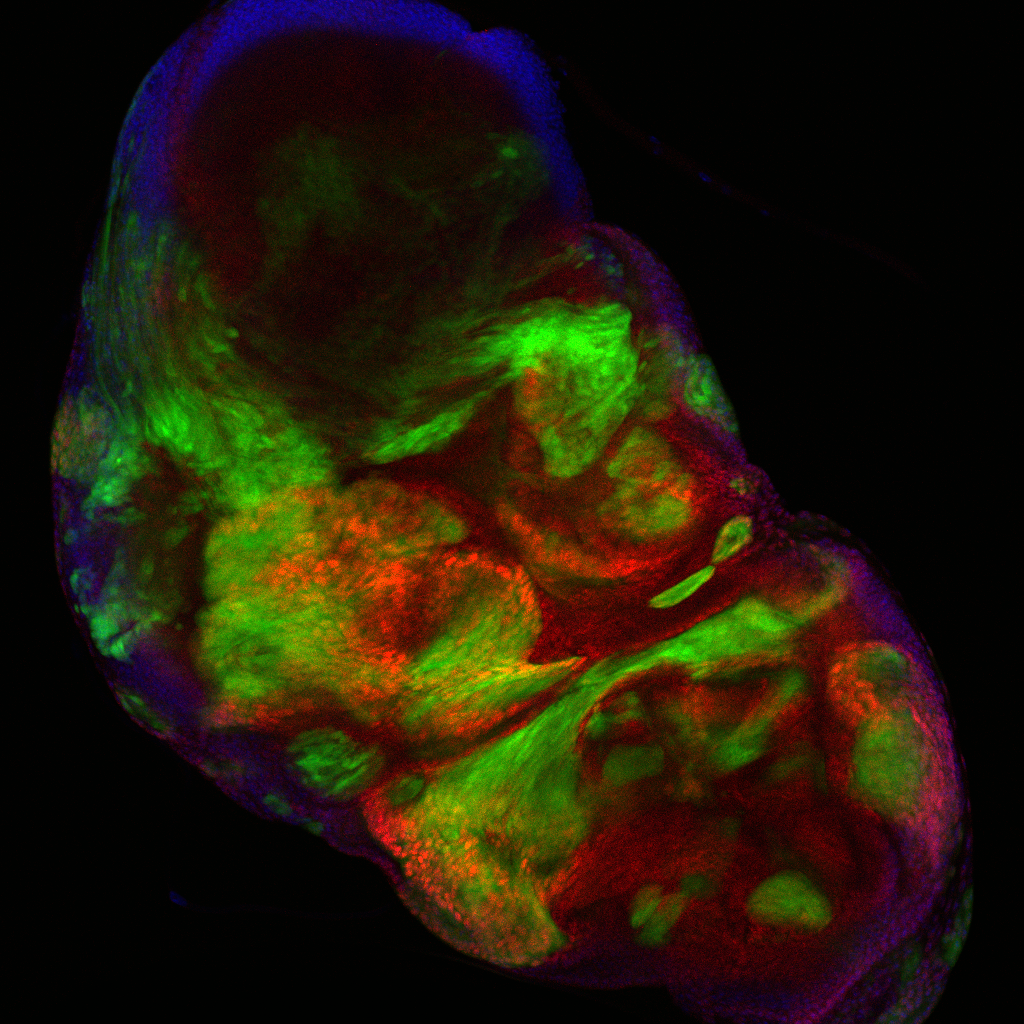

Supplement: Supplementary file 8 — Source data Fig. 4 [file 44318_2025_547_MOESM8_ESM.zip › Figure 4J/1-2 original image.tif]

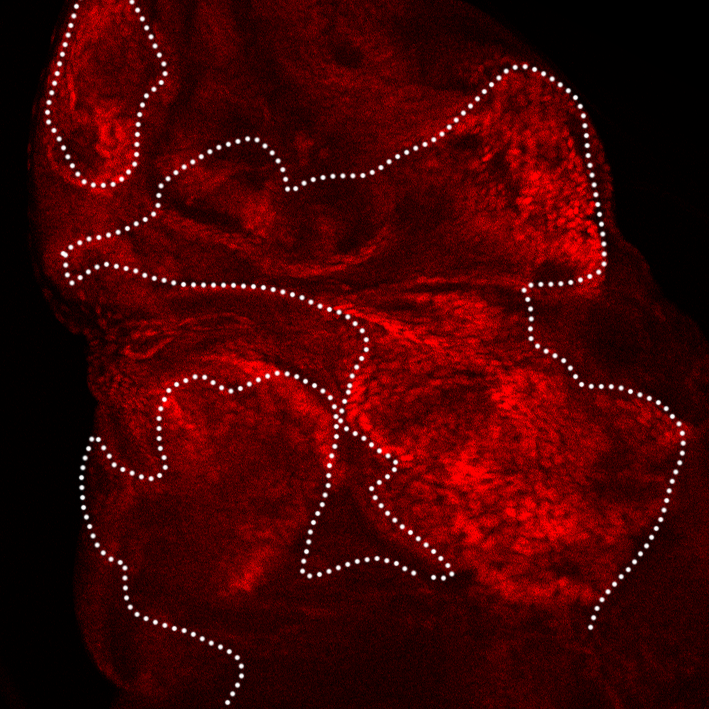

Supplement: Supplementary file 8 — Source data Fig. 4 [file 44318_2025_547_MOESM8_ESM.zip › Figure 4J/2-1 rotated and cut image with border line.tif]

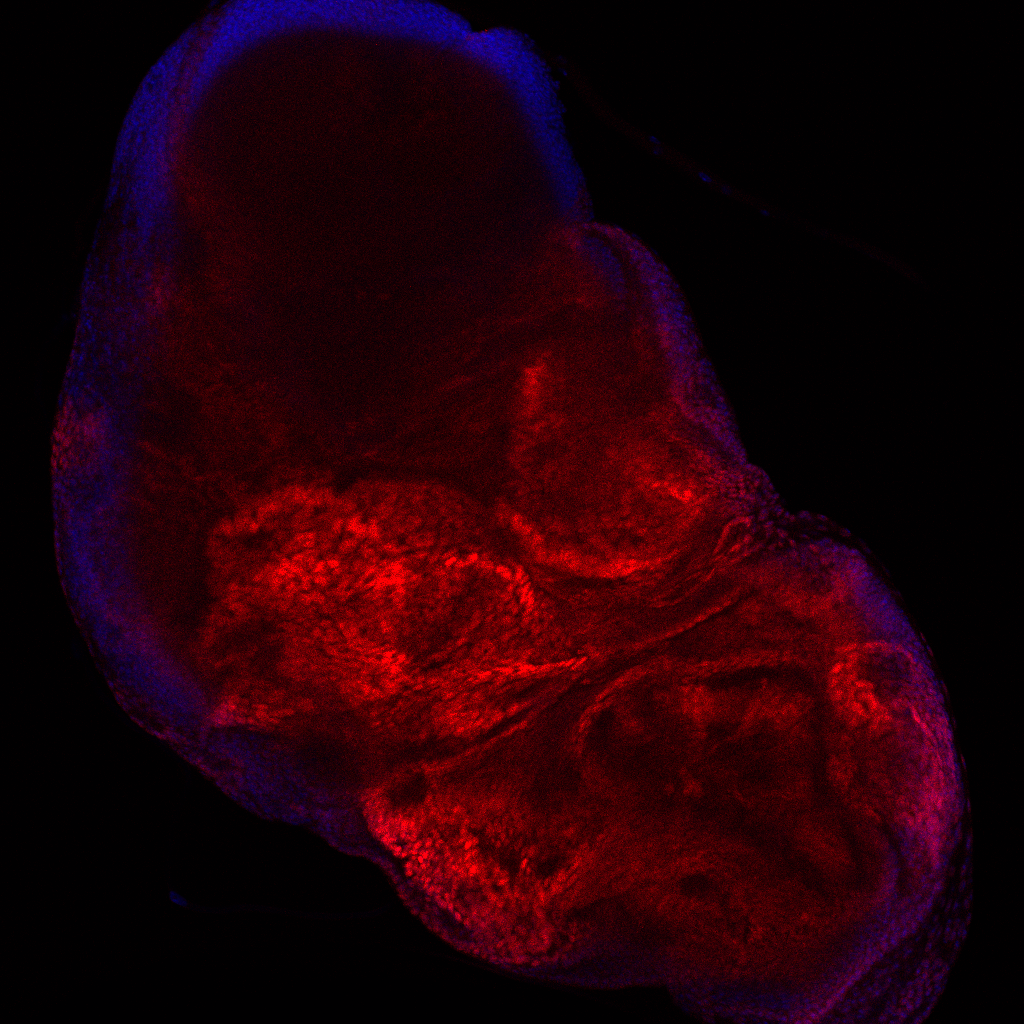

Supplement: Supplementary file 8 — Source data Fig. 4 [file 44318_2025_547_MOESM8_ESM.zip › Figure 4J/2-2 original image.tif]

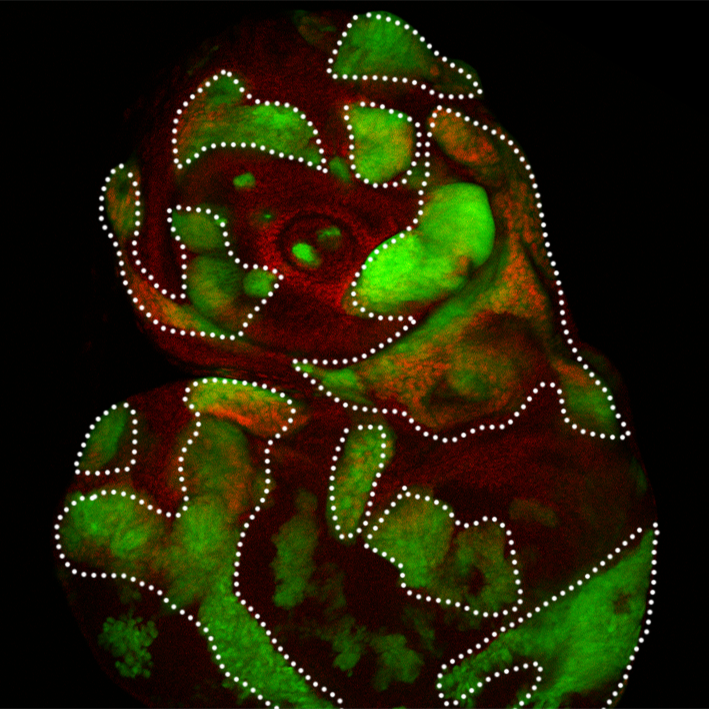

Supplement: Supplementary file 8 — Source data Fig. 4 [file 44318_2025_547_MOESM8_ESM.zip › Figure 4J/3-1 rotated and cut image with border line.tif]

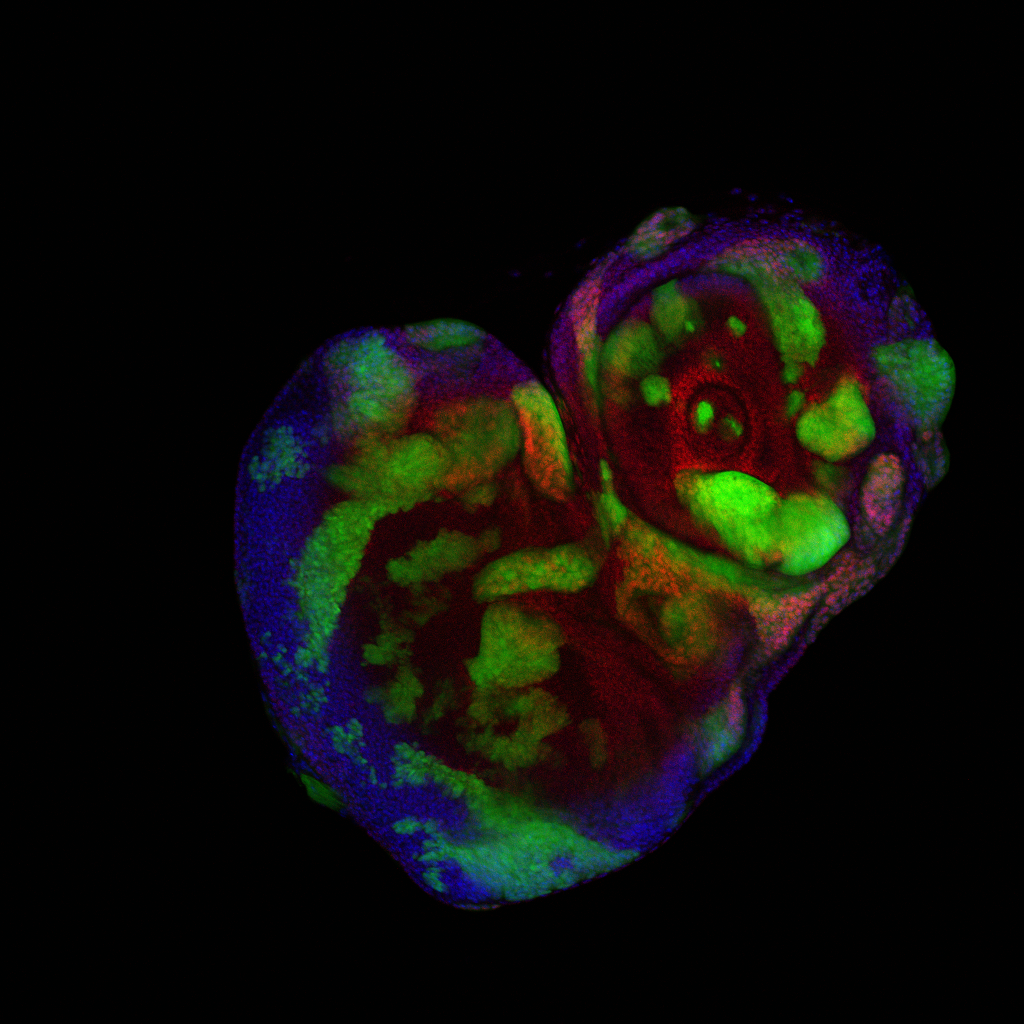

Supplement: Supplementary file 8 — Source data Fig. 4 [file 44318_2025_547_MOESM8_ESM.zip › Figure 4J/3-2 original image.tif]

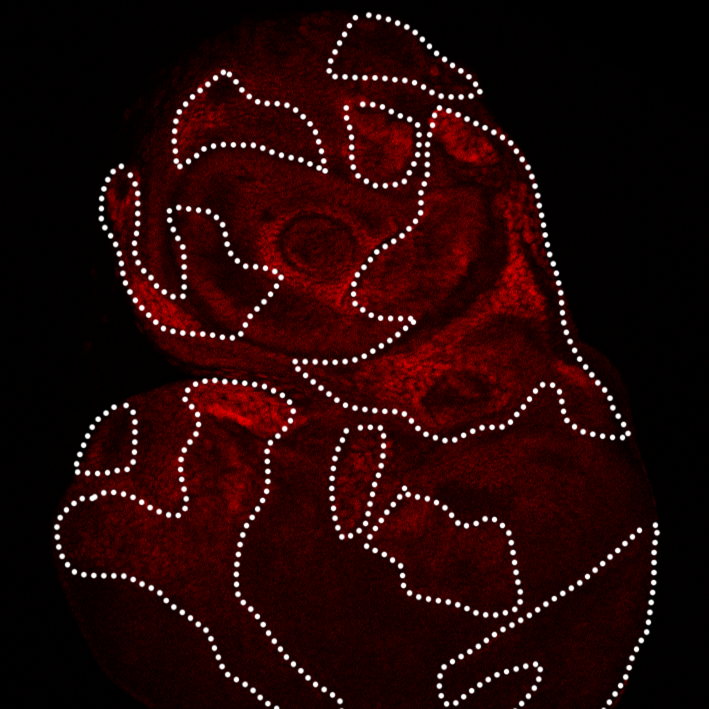

Supplement: Supplementary file 8 — Source data Fig. 4 [file 44318_2025_547_MOESM8_ESM.zip › Figure 4J/4-1 rotated and cut image with border line.tif]

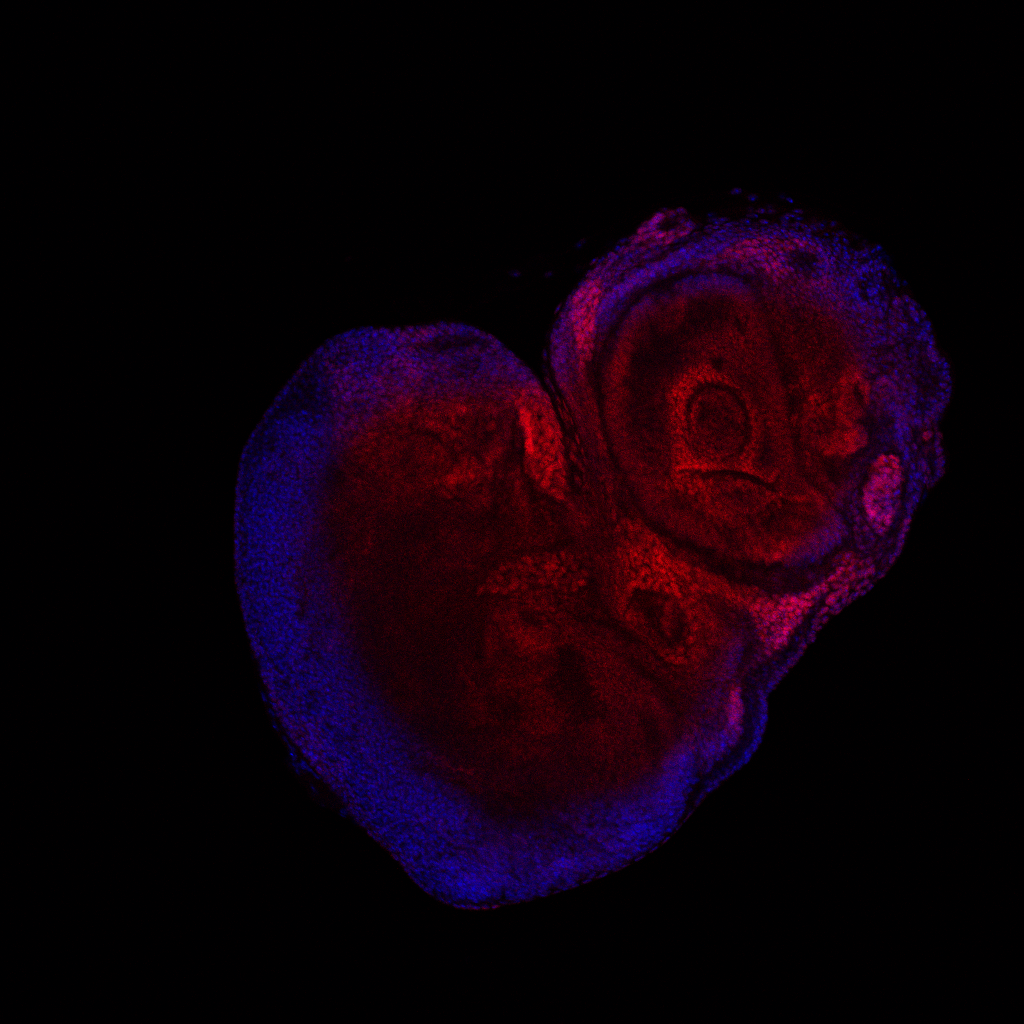

Supplement: Supplementary file 8 — Source data Fig. 4 [file 44318_2025_547_MOESM8_ESM.zip › Figure 4J/4-2 original image.tif]

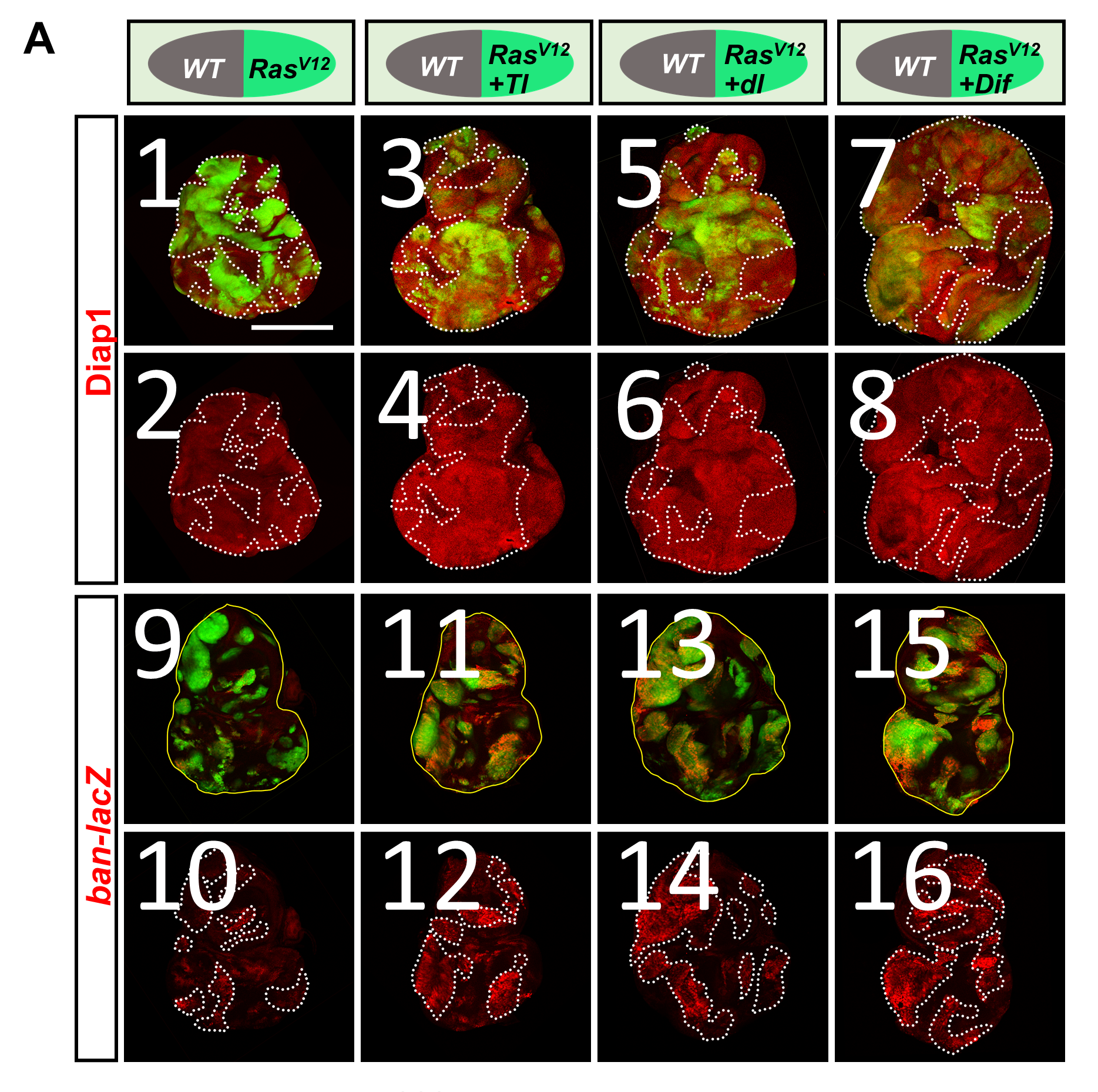

Supplement: Supplementary file 8 — Source data Fig. 4 [file 44318_2025_547_MOESM8_ESM.zip › Figure 4A/0 paper Figure 4A with provided image sequence.tif]

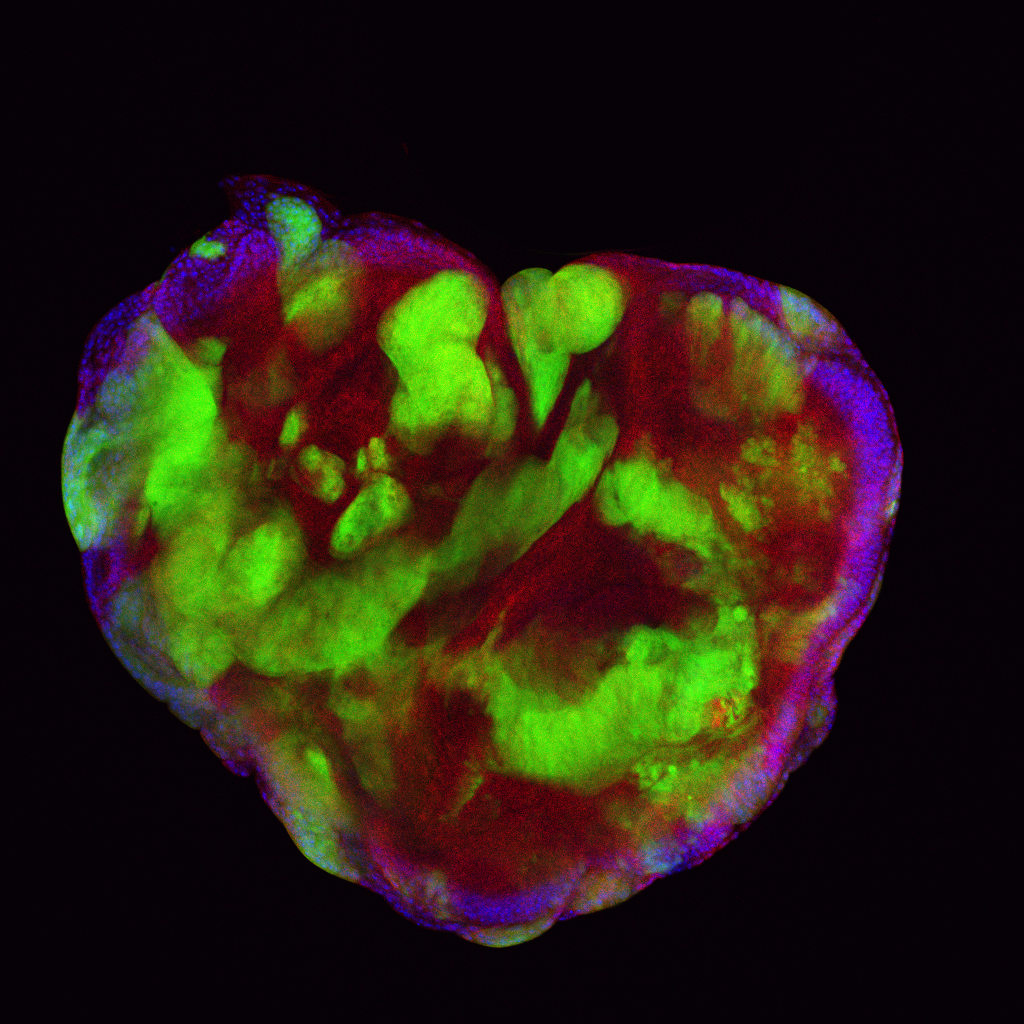

Supplement: Supplementary file 8 — Source data Fig. 4 [file 44318_2025_547_MOESM8_ESM.zip › Figure 4A/1 original image.tif]

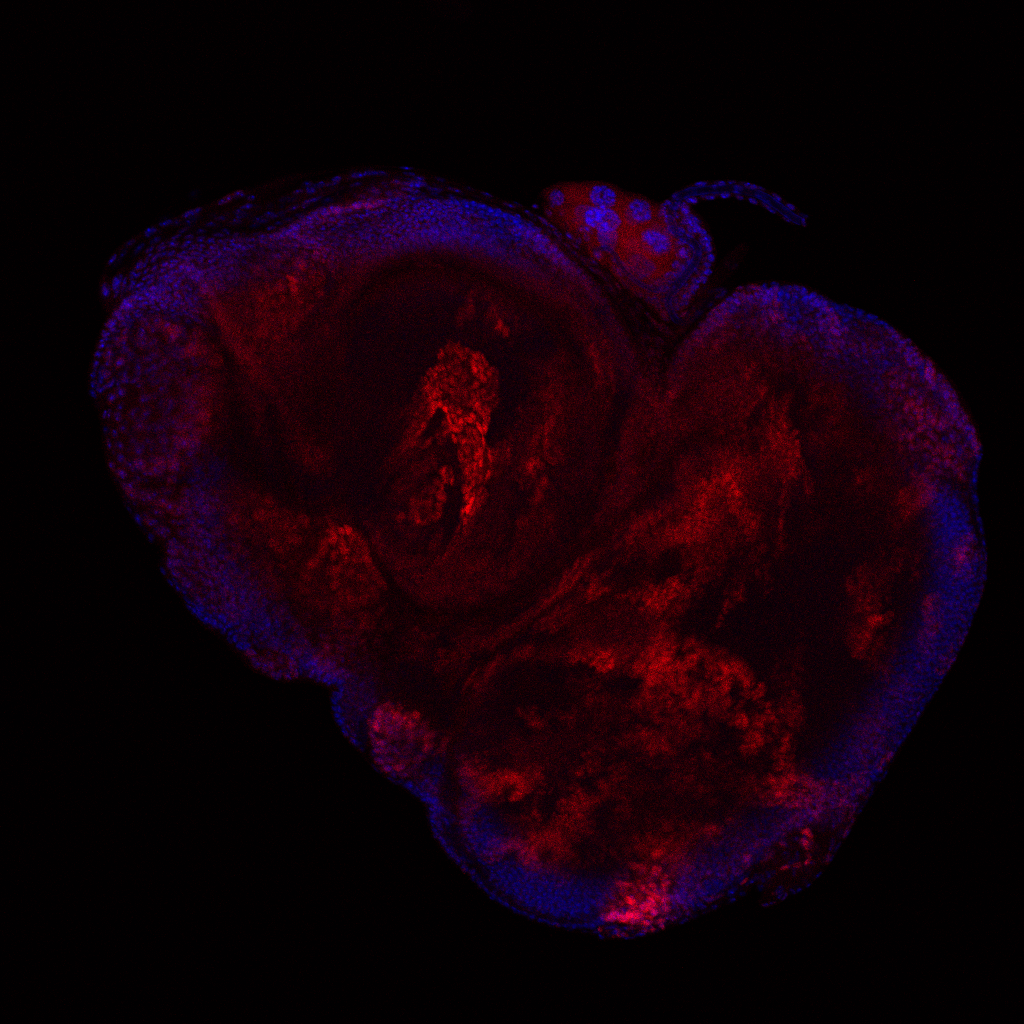

Supplement: Supplementary file 8 — Source data Fig. 4 [file 44318_2025_547_MOESM8_ESM.zip › Figure 4A/10 original image.tif]

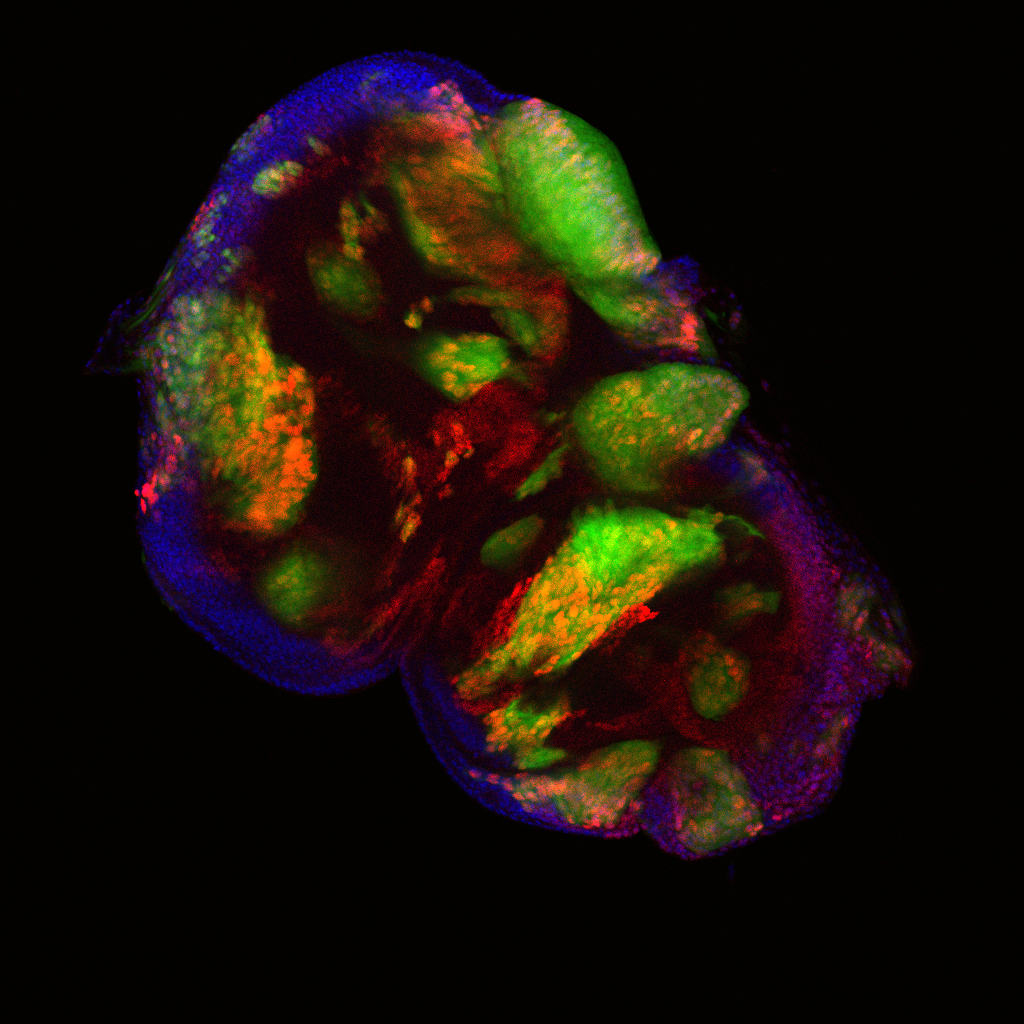

Supplement: Supplementary file 8 — Source data Fig. 4 [file 44318_2025_547_MOESM8_ESM.zip › Figure 4A/11 original image.tif]

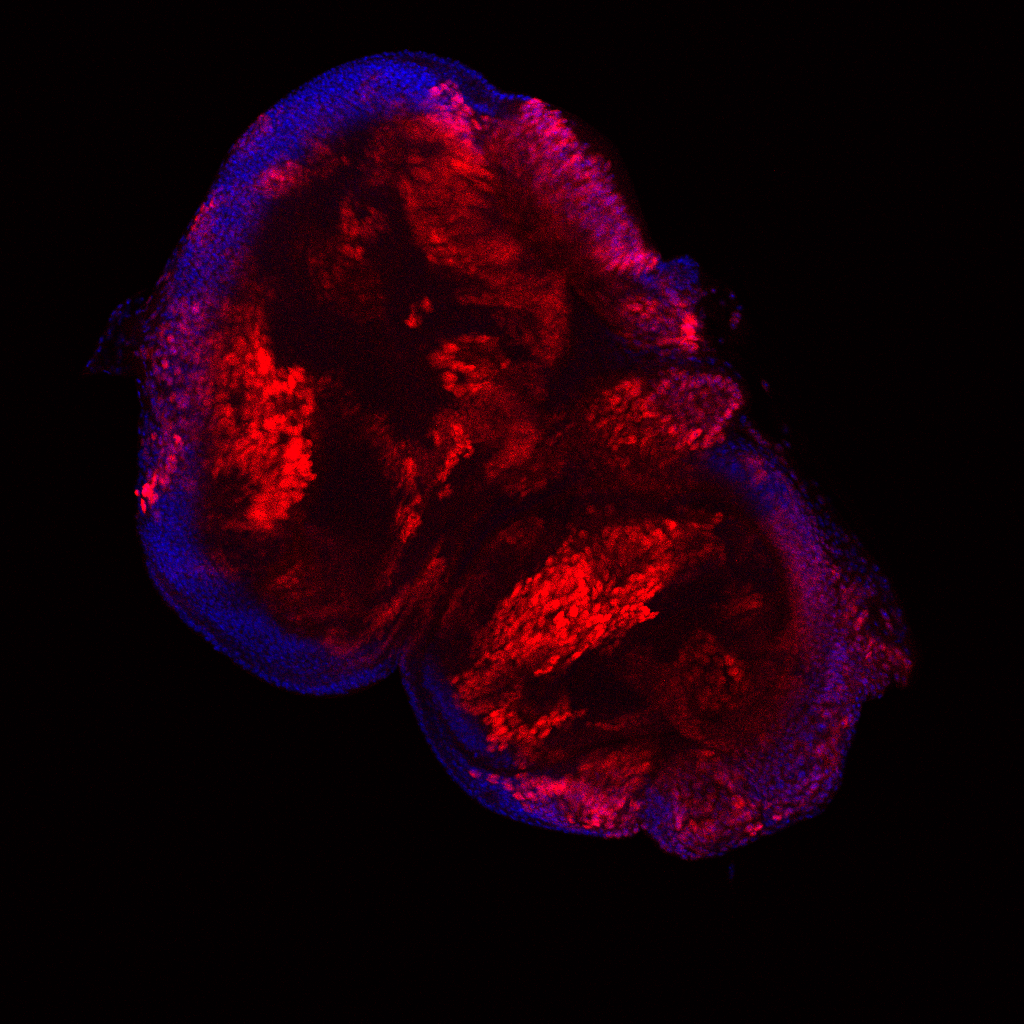

Supplement: Supplementary file 8 — Source data Fig. 4 [file 44318_2025_547_MOESM8_ESM.zip › Figure 4A/12 original image.tif]

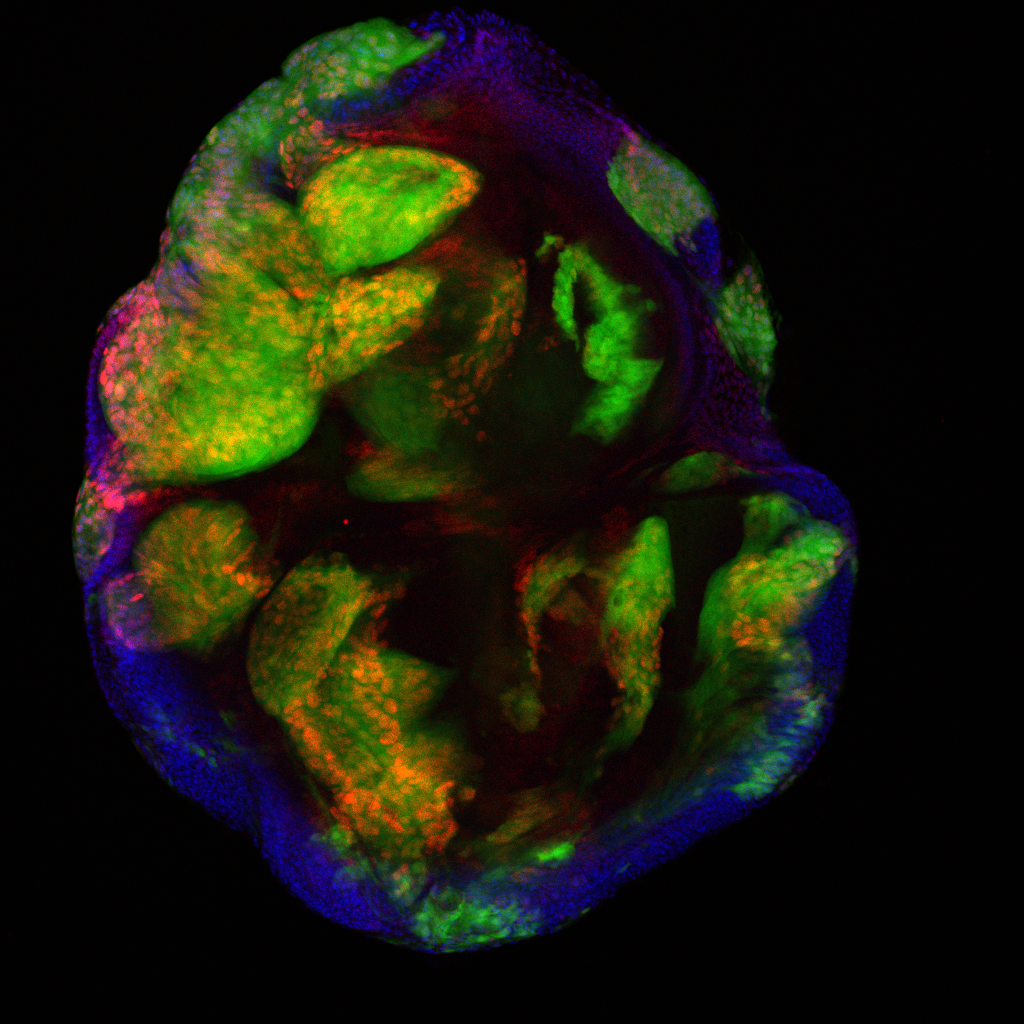

Supplement: Supplementary file 8 — Source data Fig. 4 [file 44318_2025_547_MOESM8_ESM.zip › Figure 4A/13 original image.tif]

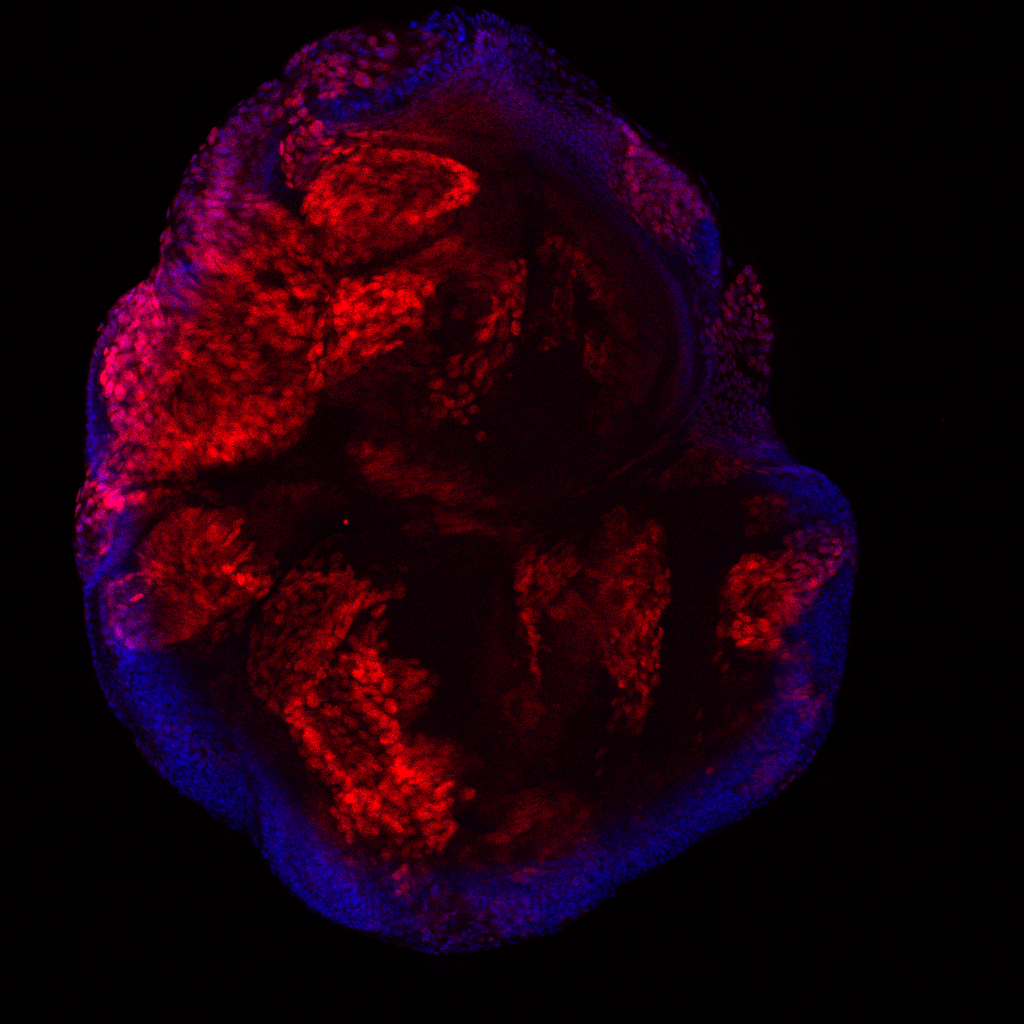

Supplement: Supplementary file 8 — Source data Fig. 4 [file 44318_2025_547_MOESM8_ESM.zip › Figure 4A/14 original image.tif]

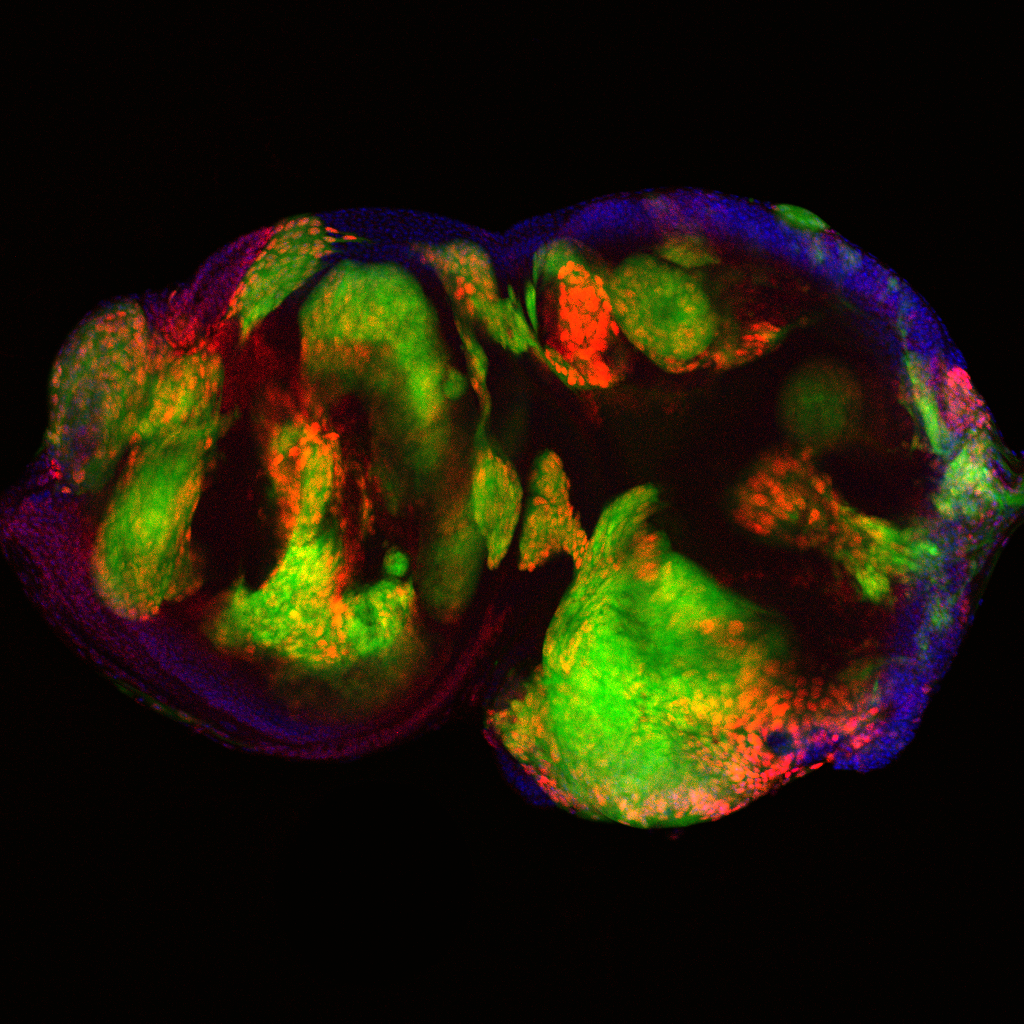

Supplement: Supplementary file 8 — Source data Fig. 4 [file 44318_2025_547_MOESM8_ESM.zip › Figure 4A/15 original image.tif]

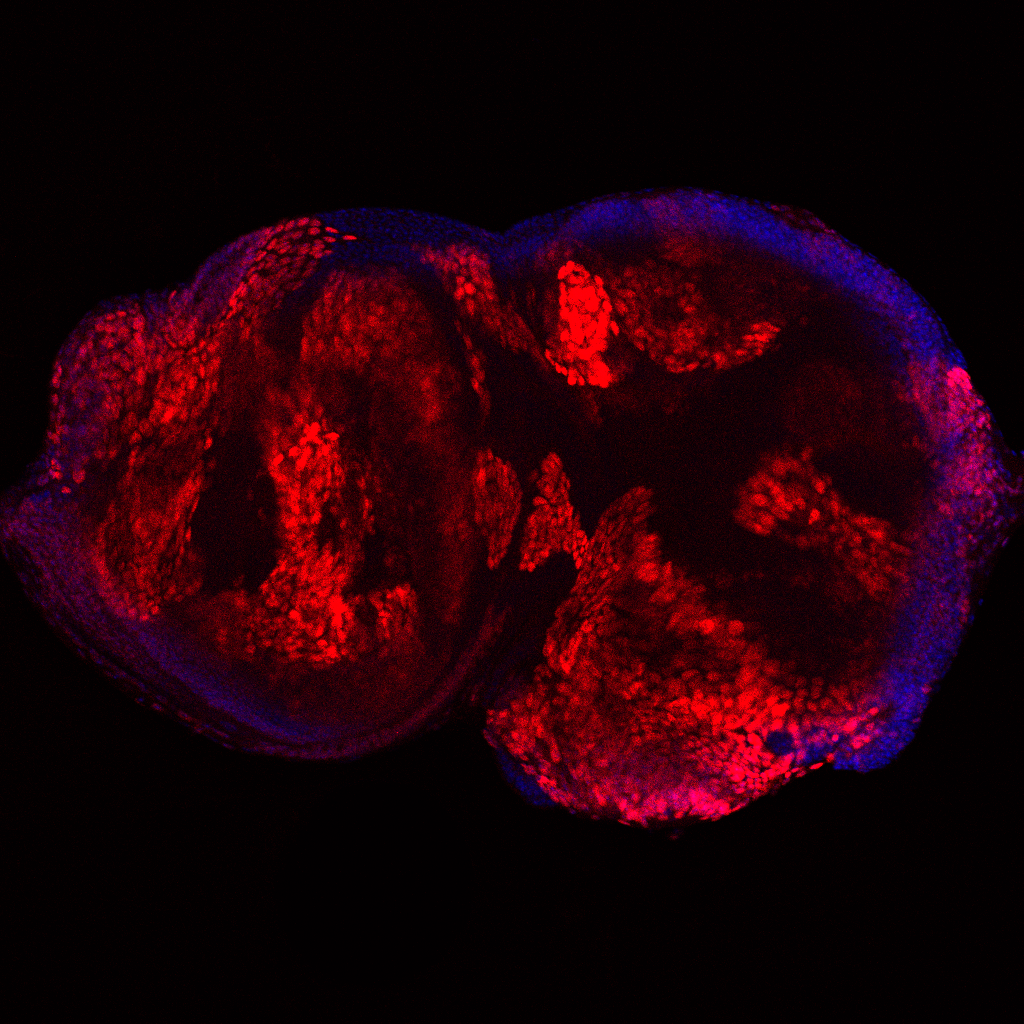

Supplement: Supplementary file 8 — Source data Fig. 4 [file 44318_2025_547_MOESM8_ESM.zip › Figure 4A/16 original image.tif]

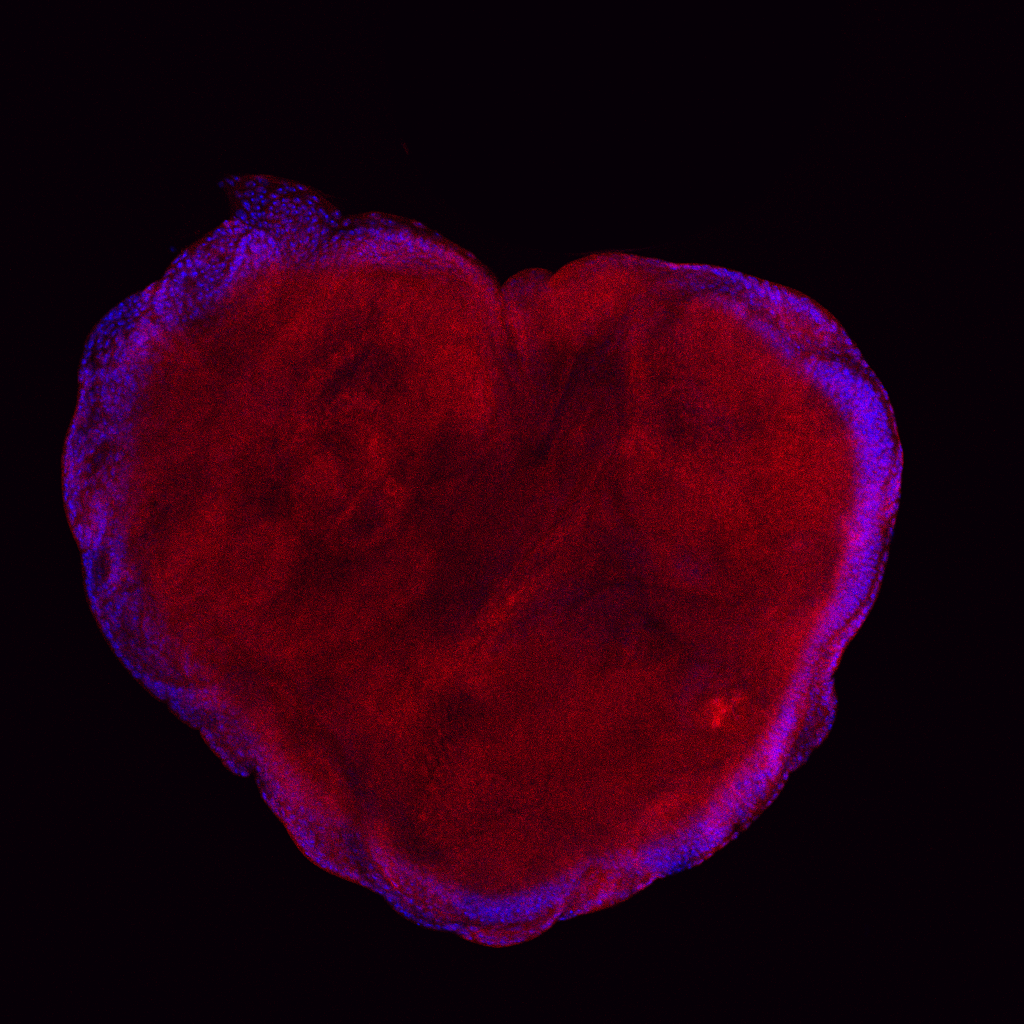

Supplement: Supplementary file 8 — Source data Fig. 4 [file 44318_2025_547_MOESM8_ESM.zip › Figure 4A/2 original image.tif]

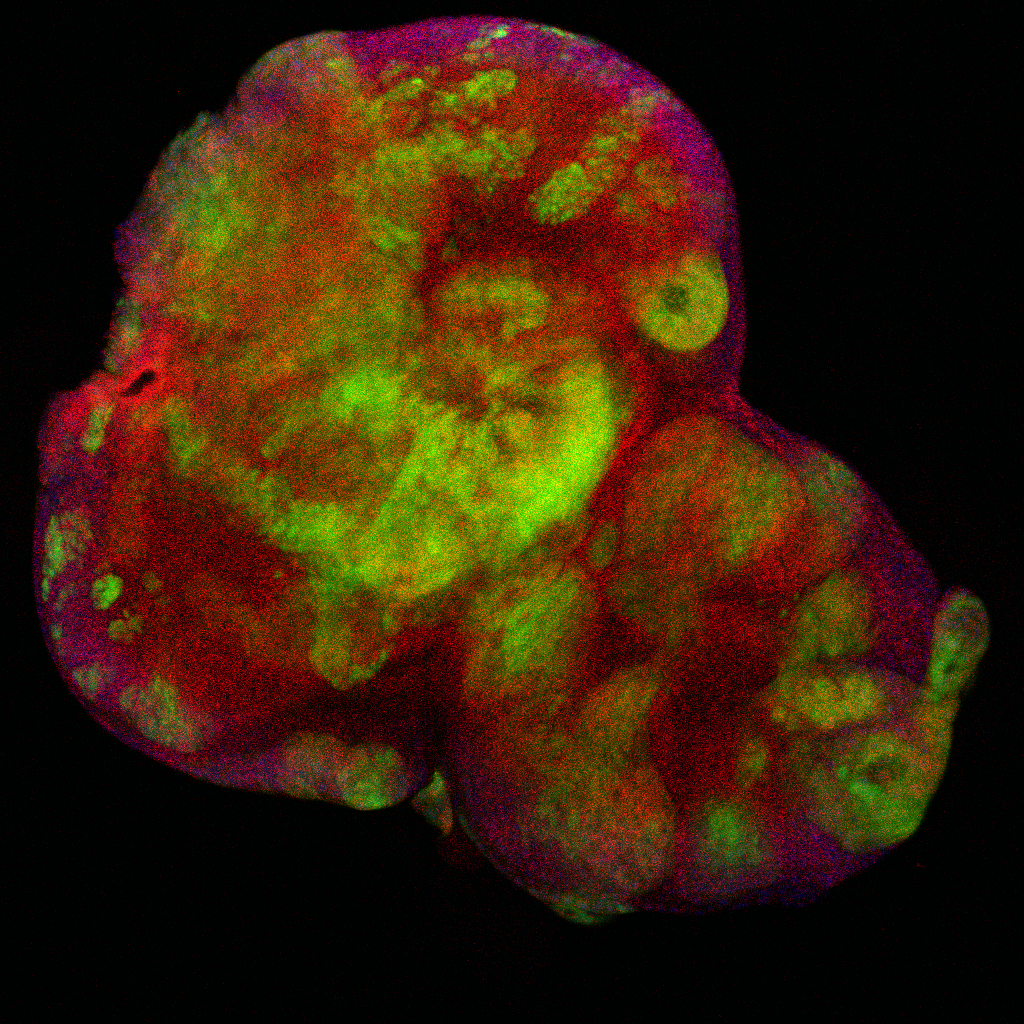

Supplement: Supplementary file 8 — Source data Fig. 4 [file 44318_2025_547_MOESM8_ESM.zip › Figure 4A/3 original image.tif]

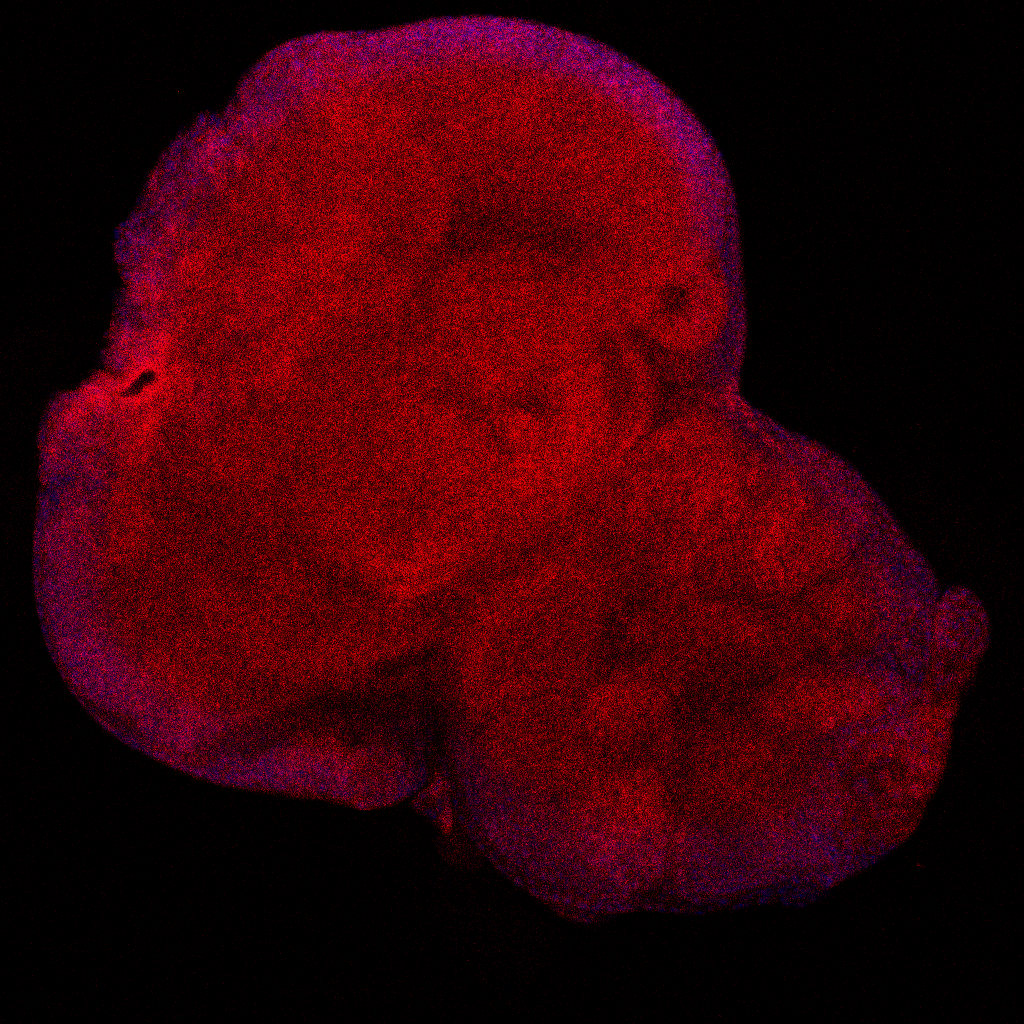

Supplement: Supplementary file 8 — Source data Fig. 4 [file 44318_2025_547_MOESM8_ESM.zip › Figure 4A/4 original image.tif]

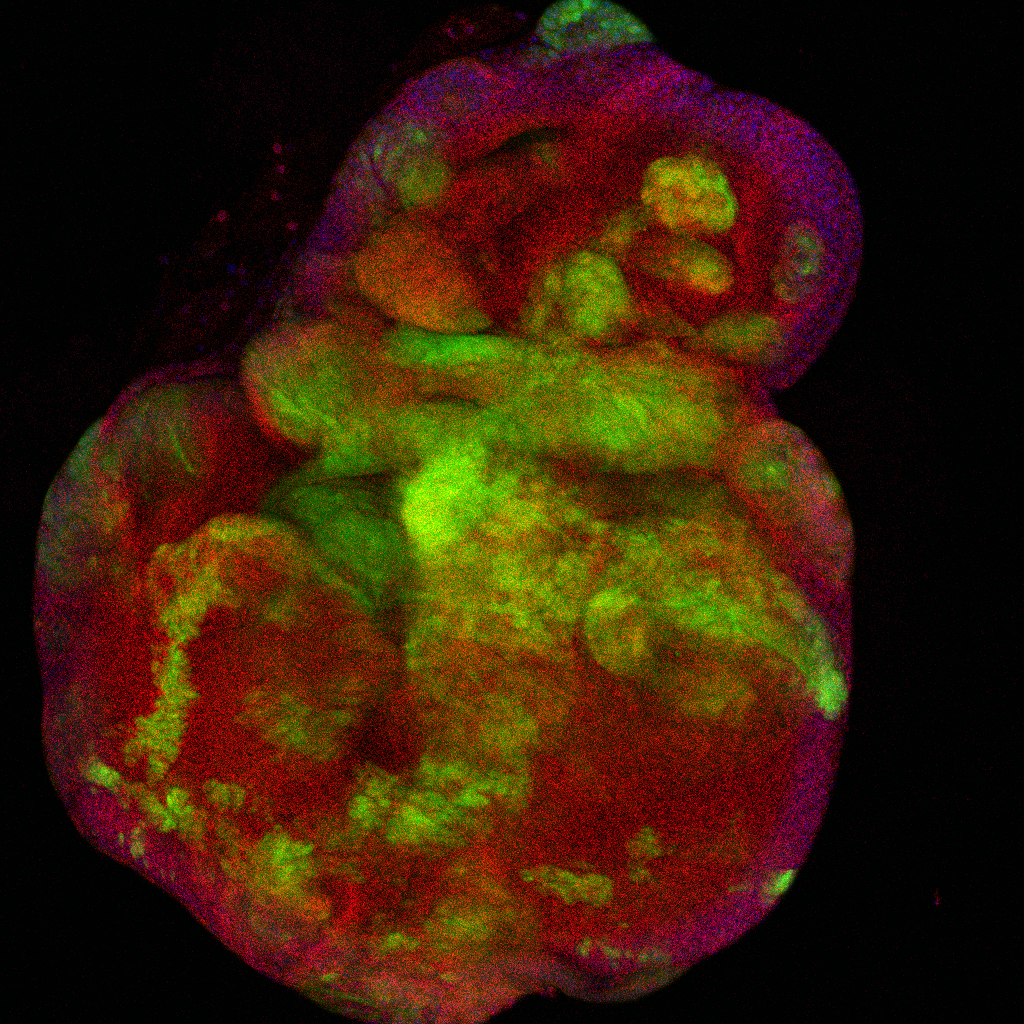

Supplement: Supplementary file 8 — Source data Fig. 4 [file 44318_2025_547_MOESM8_ESM.zip › Figure 4A/5 original image.tif]

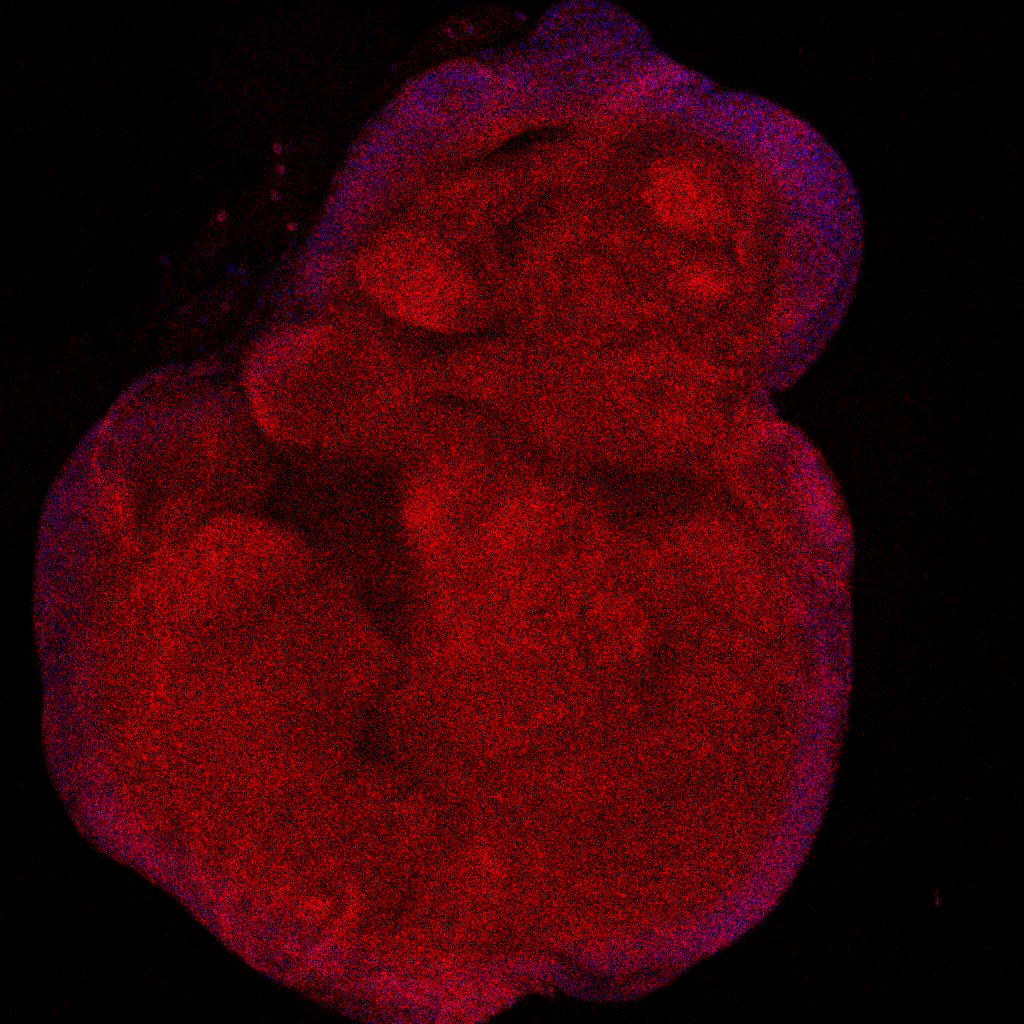

Supplement: Supplementary file 8 — Source data Fig. 4 [file 44318_2025_547_MOESM8_ESM.zip › Figure 4A/6 original image.tif]

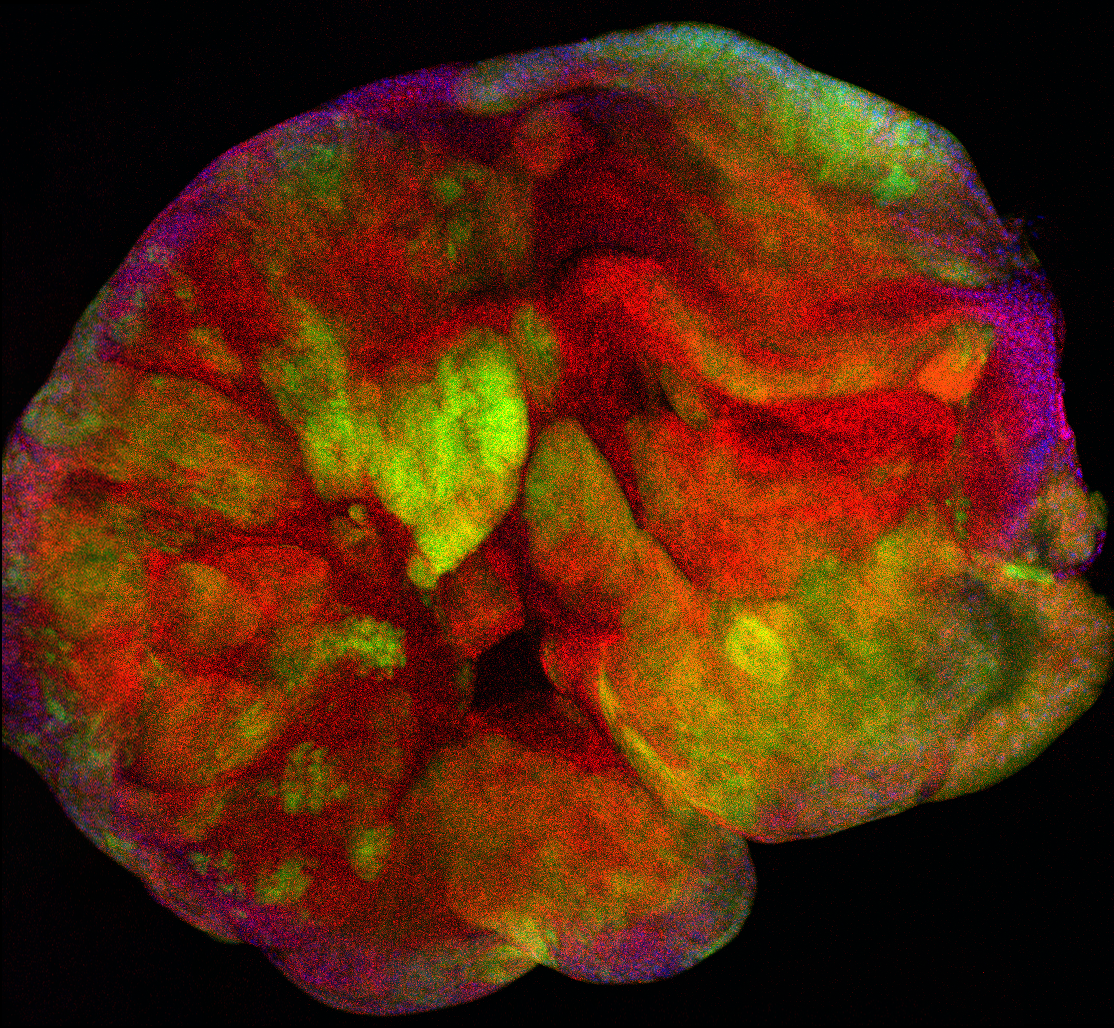

Supplement: Supplementary file 8 — Source data Fig. 4 [file 44318_2025_547_MOESM8_ESM.zip › Figure 4A/7 original image.tif]

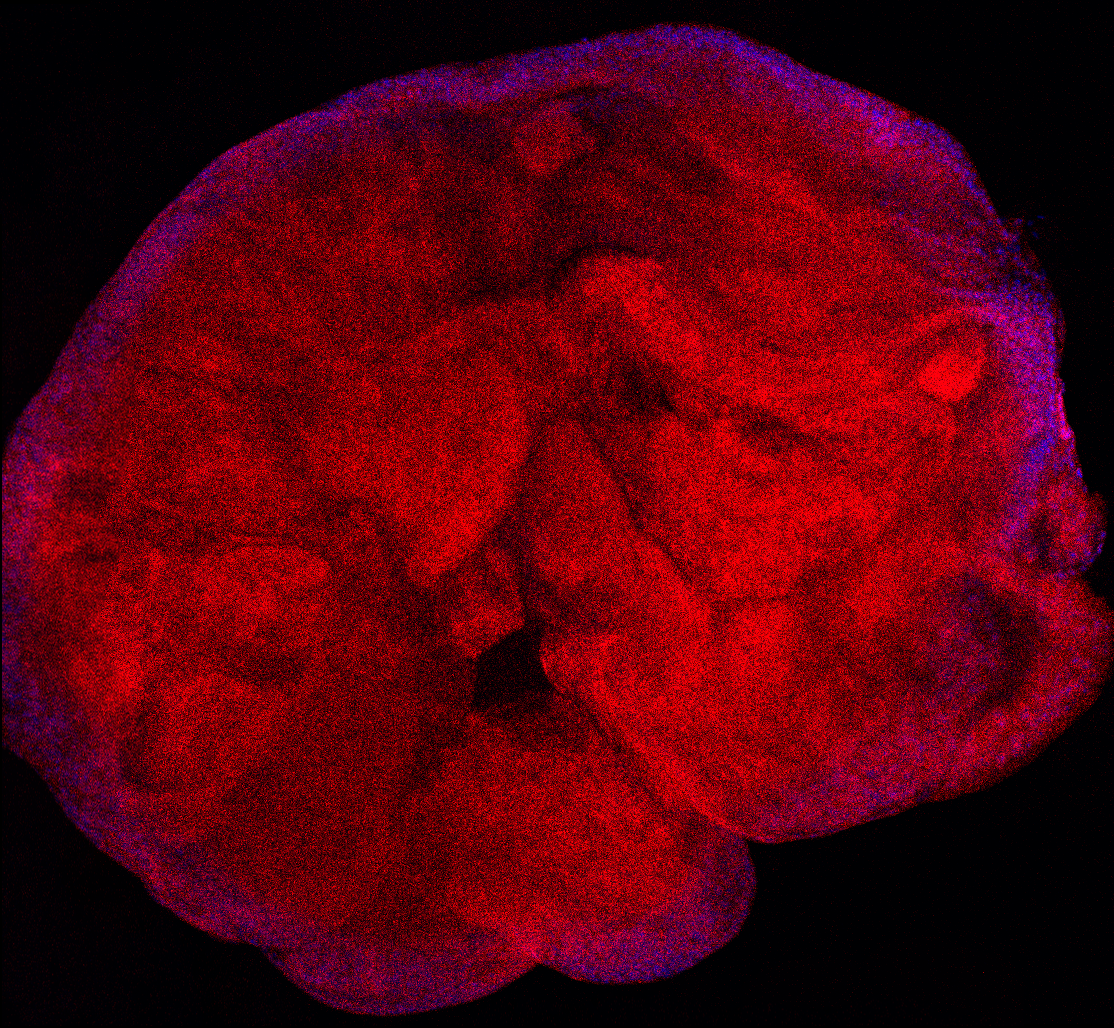

Supplement: Supplementary file 8 — Source data Fig. 4 [file 44318_2025_547_MOESM8_ESM.zip › Figure 4A/8 original image.tif]

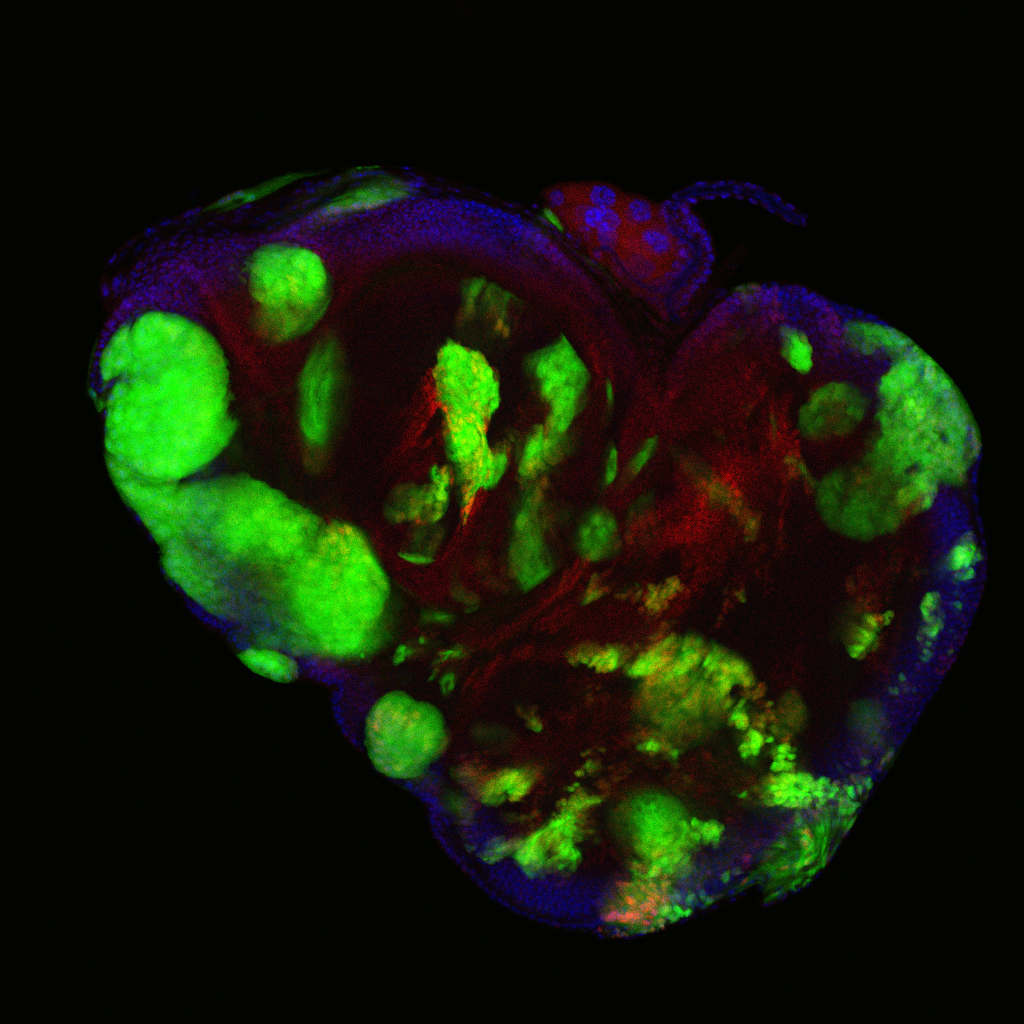

Supplement: Supplementary file 8 — Source data Fig. 4 [file 44318_2025_547_MOESM8_ESM.zip › Figure 4A/9 original image.tif]

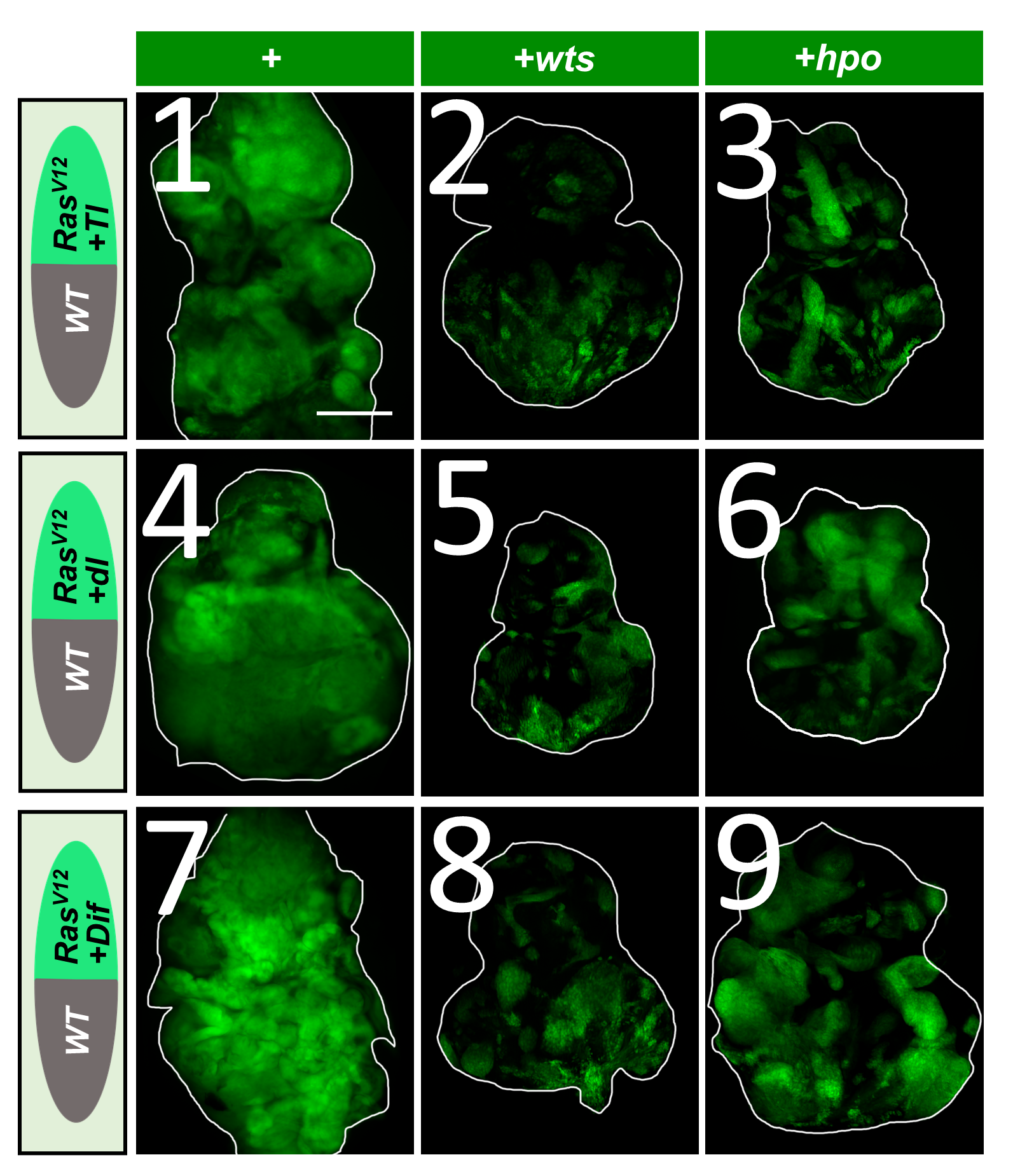

Supplement: Supplementary file 8 — Source data Fig. 4 [file 44318_2025_547_MOESM8_ESM.zip › Figure 4C/0 paper Figure 4C with provided image sequence.tif]

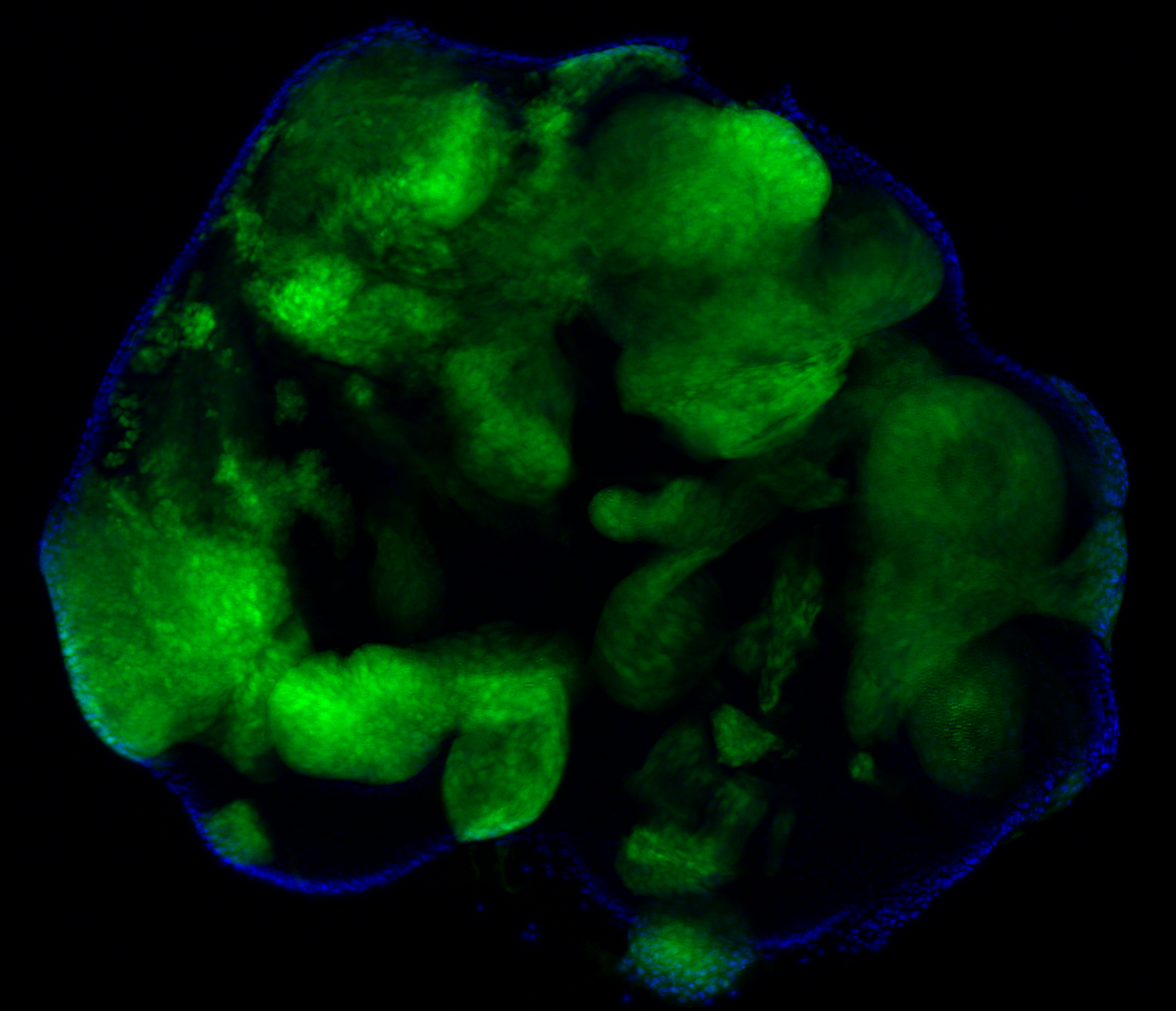

Supplement: Supplementary file 8 — Source data Fig. 4 [file 44318_2025_547_MOESM8_ESM.zip › Figure 4C/9 original image.tif]

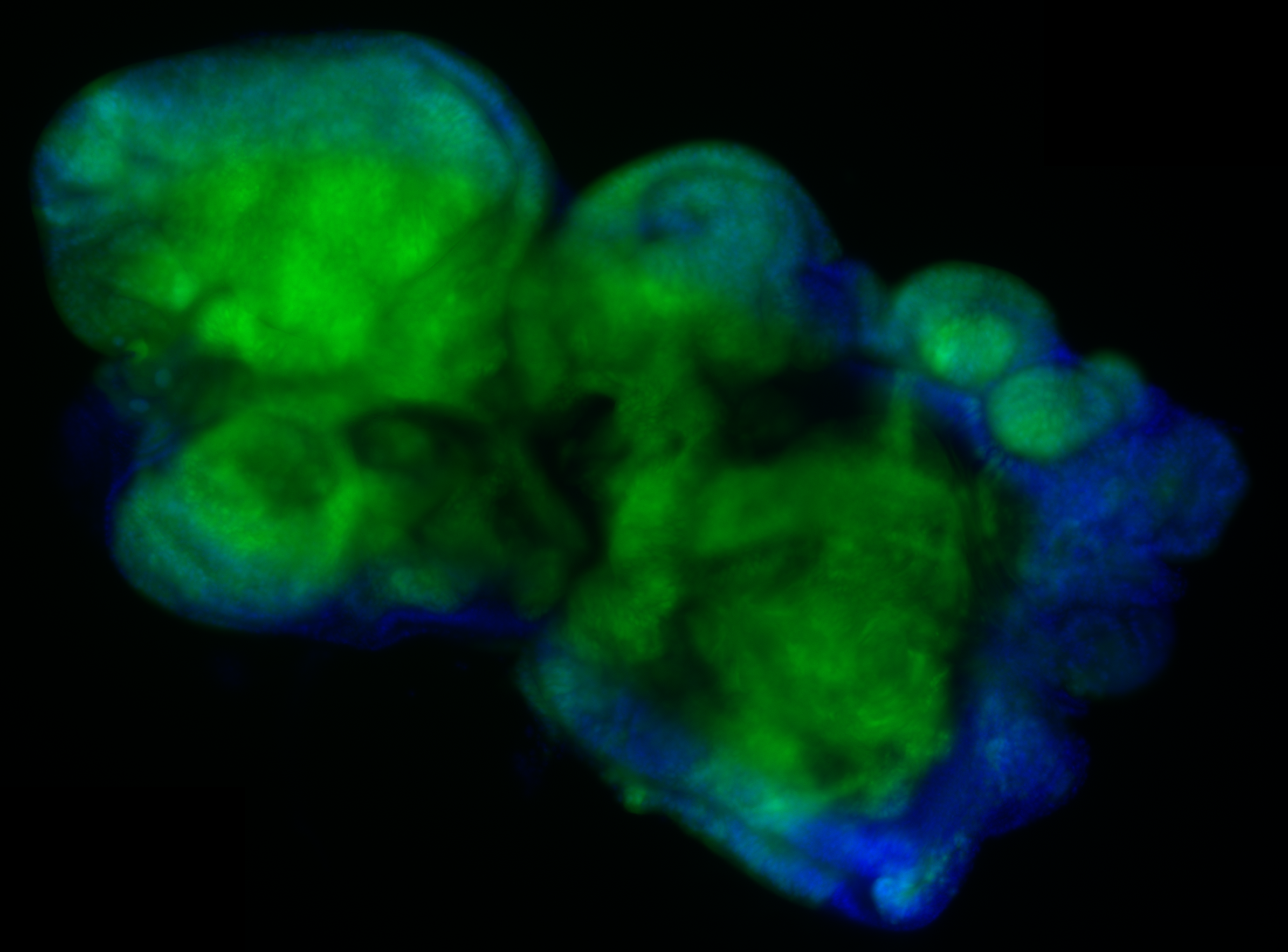

Supplement: Supplementary file 8 — Source data Fig. 4 [file 44318_2025_547_MOESM8_ESM.zip › Figure 4C/1 original image.tif]

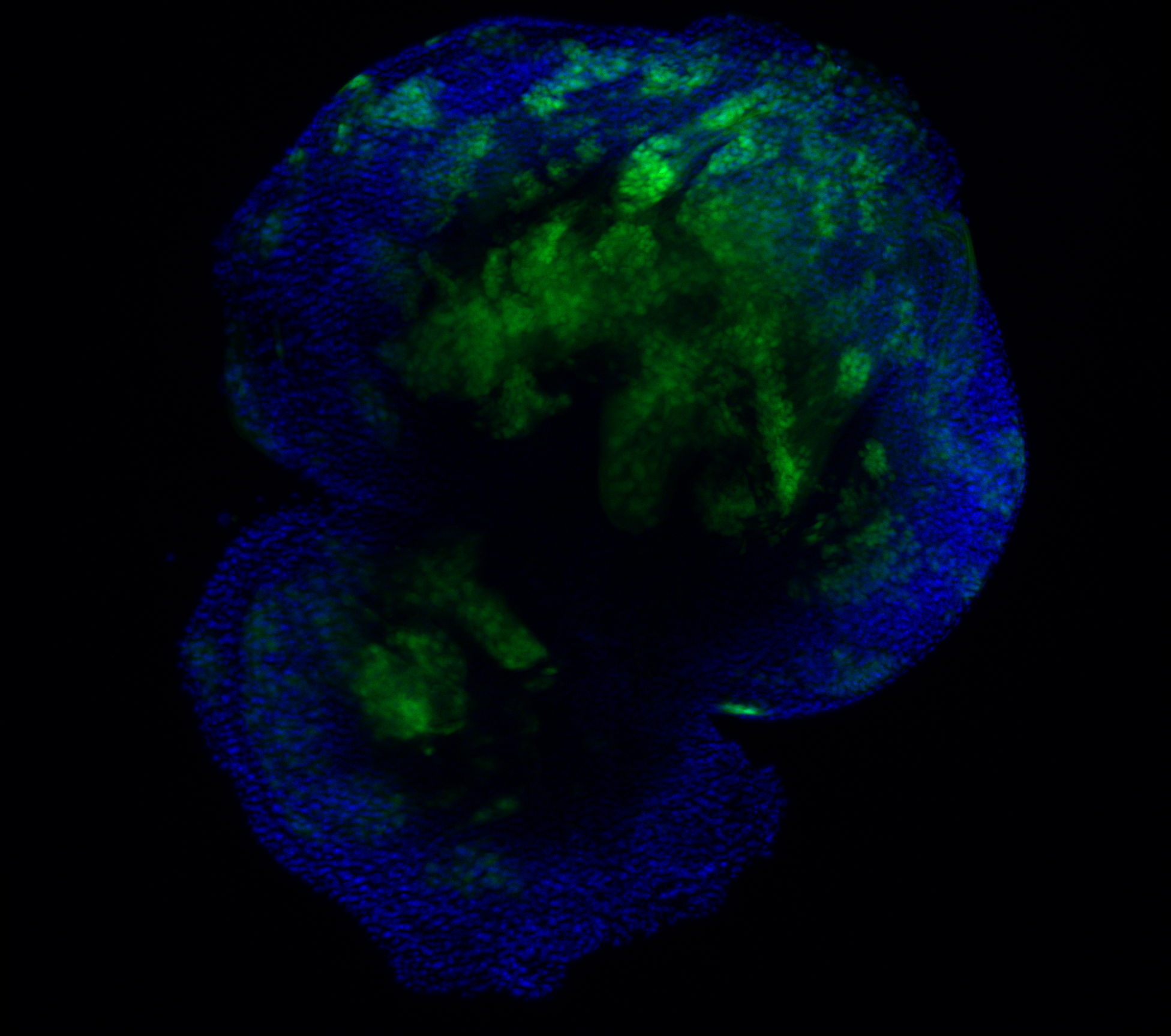

Supplement: Supplementary file 8 — Source data Fig. 4 [file 44318_2025_547_MOESM8_ESM.zip › Figure 4C/2 original image.tif]

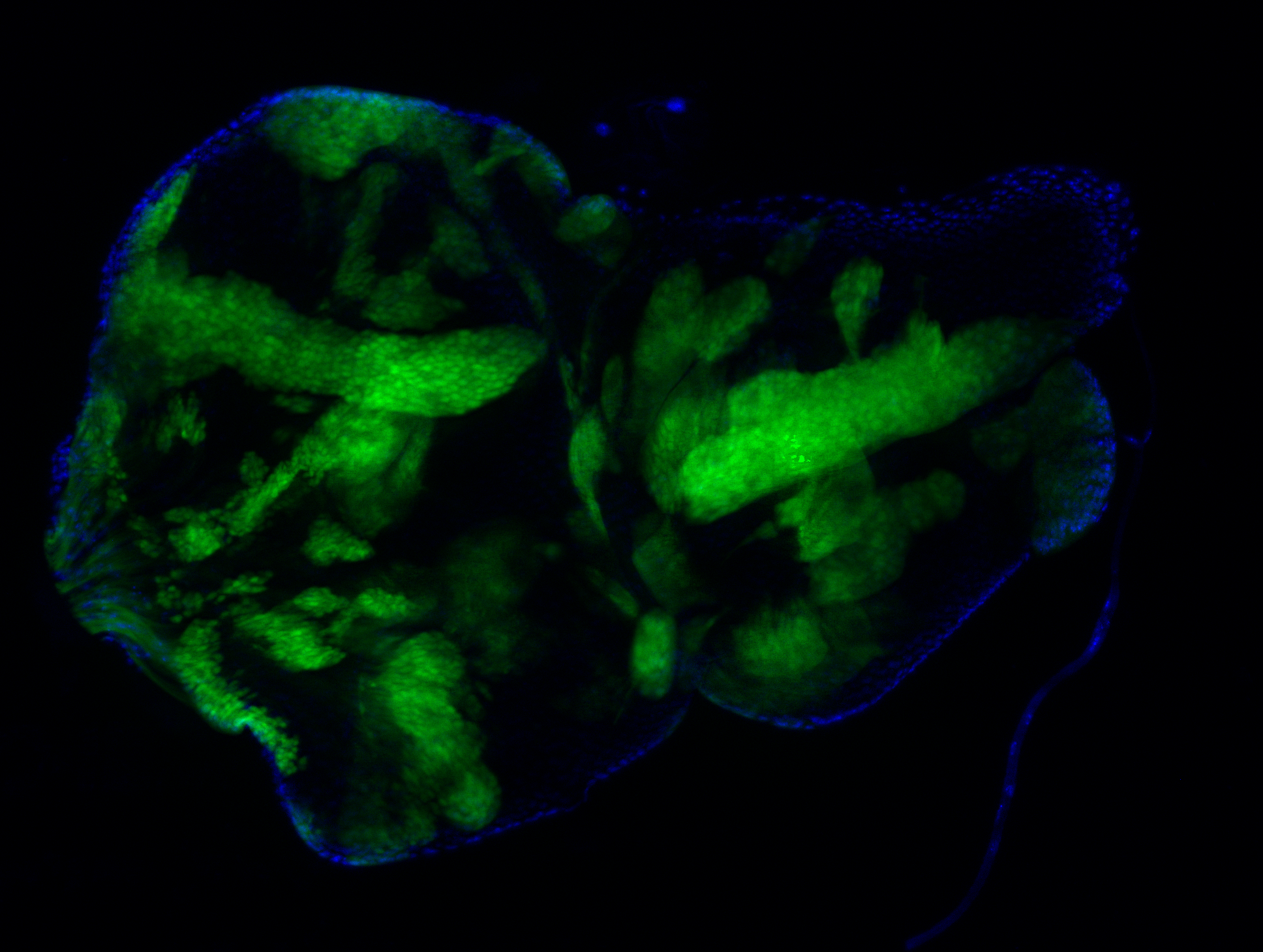

Supplement: Supplementary file 8 — Source data Fig. 4 [file 44318_2025_547_MOESM8_ESM.zip › Figure 4C/3 original image.tif]

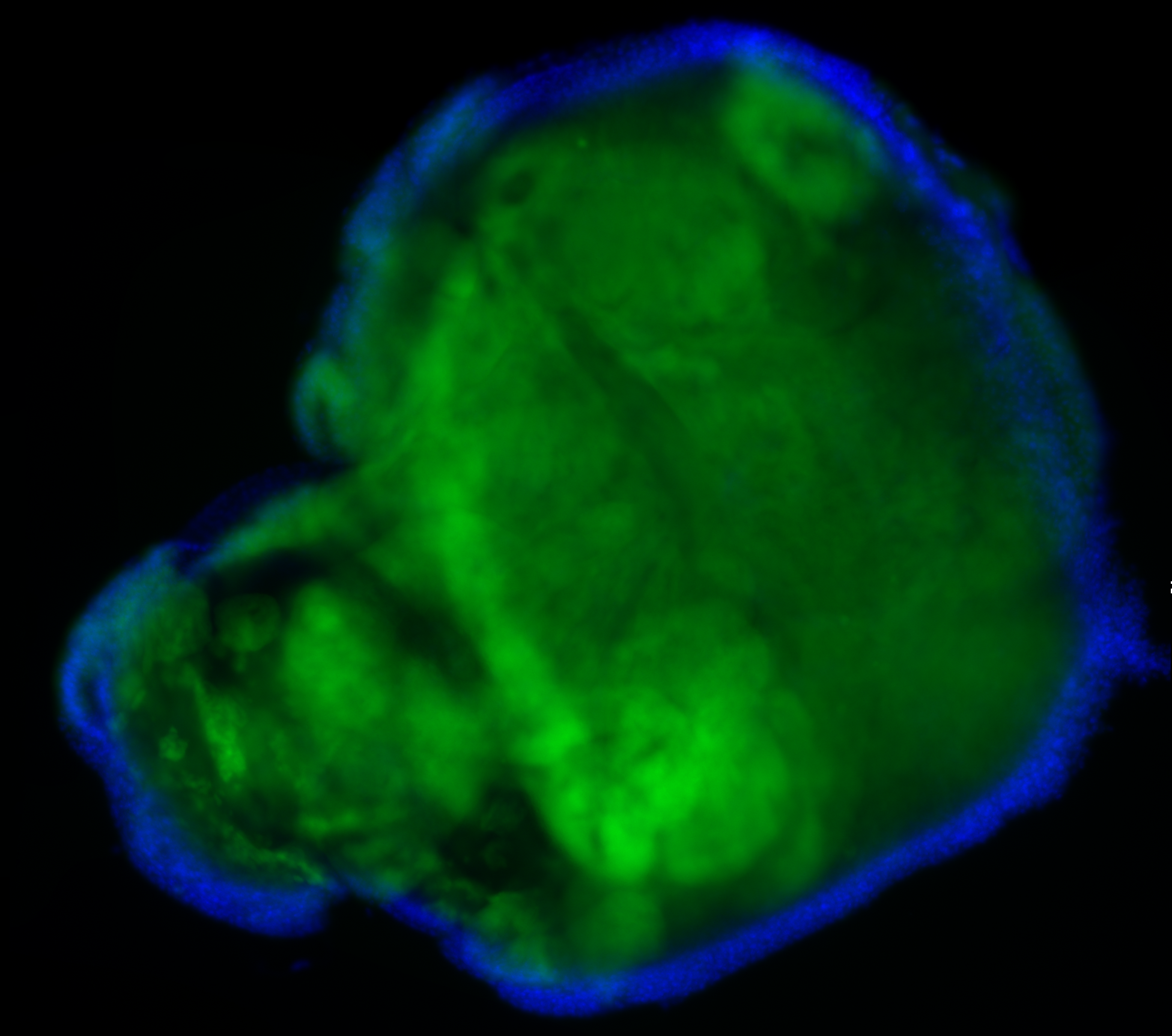

Supplement: Supplementary file 8 — Source data Fig. 4 [file 44318_2025_547_MOESM8_ESM.zip › Figure 4C/4 original image.tif]

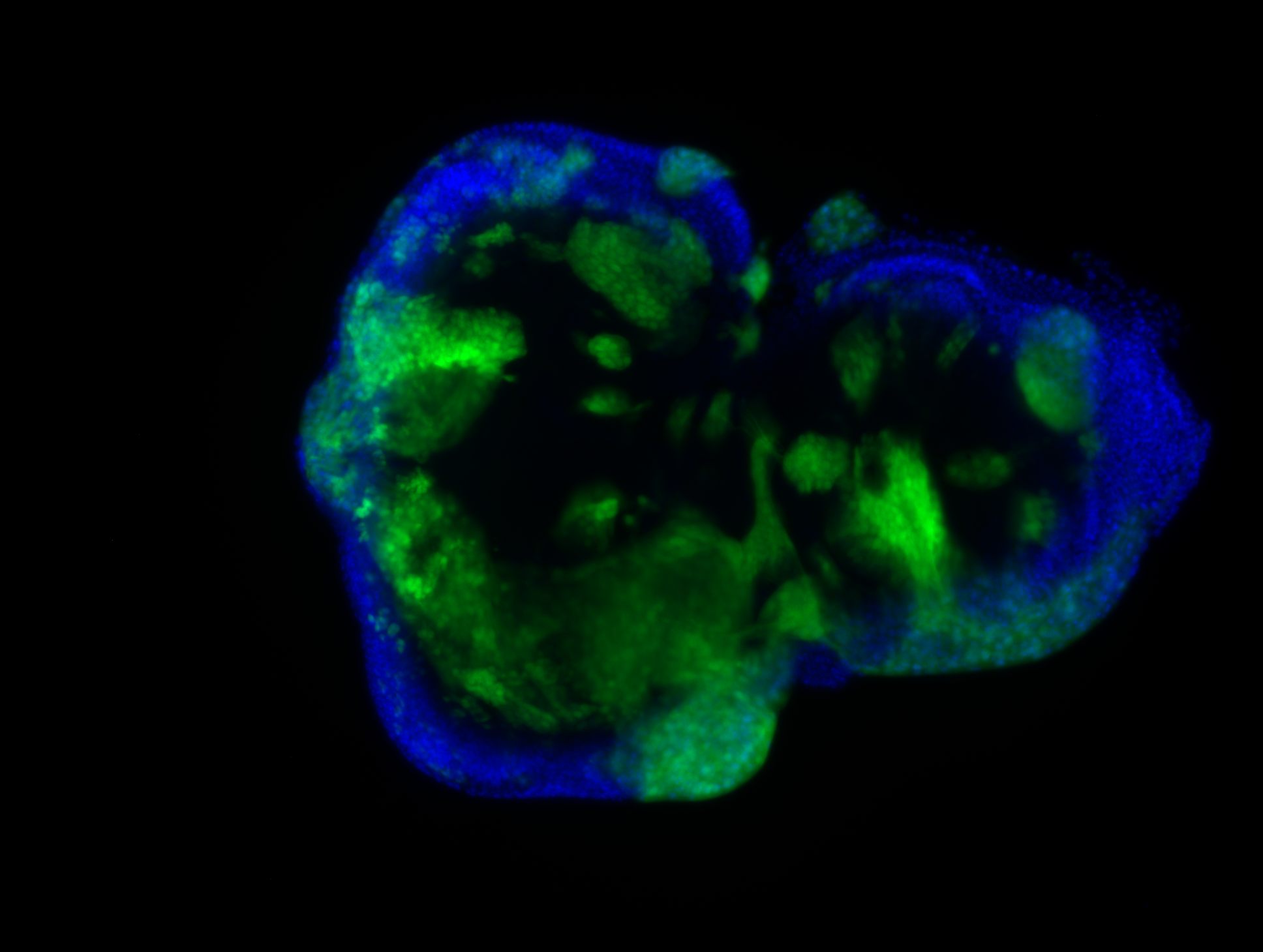

Supplement: Supplementary file 8 — Source data Fig. 4 [file 44318_2025_547_MOESM8_ESM.zip › Figure 4C/5 original image.tif]

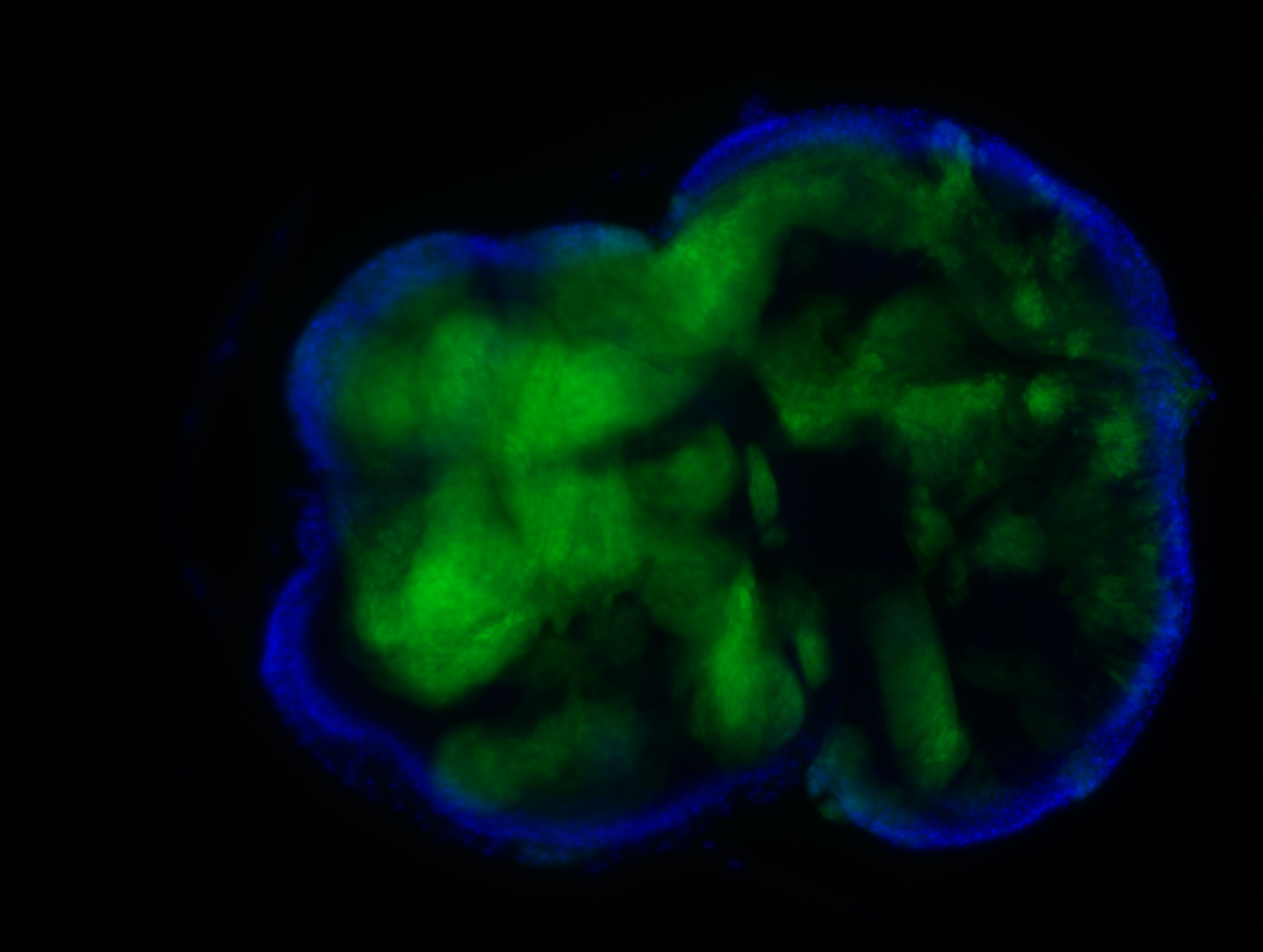

Supplement: Supplementary file 8 — Source data Fig. 4 [file 44318_2025_547_MOESM8_ESM.zip › Figure 4C/6 original image.tif]

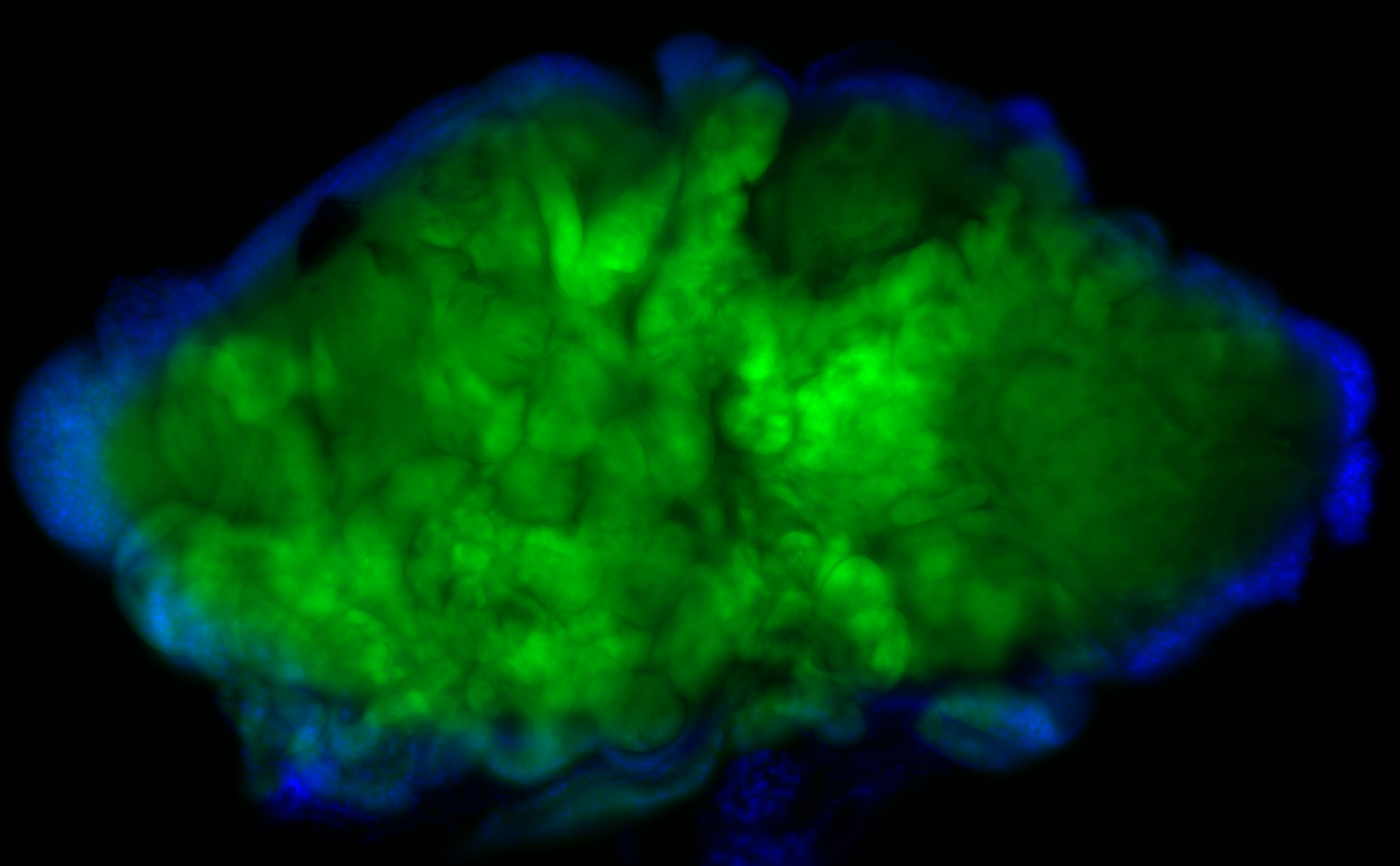

Supplement: Supplementary file 8 — Source data Fig. 4 [file 44318_2025_547_MOESM8_ESM.zip › Figure 4C/7 original image.tif]

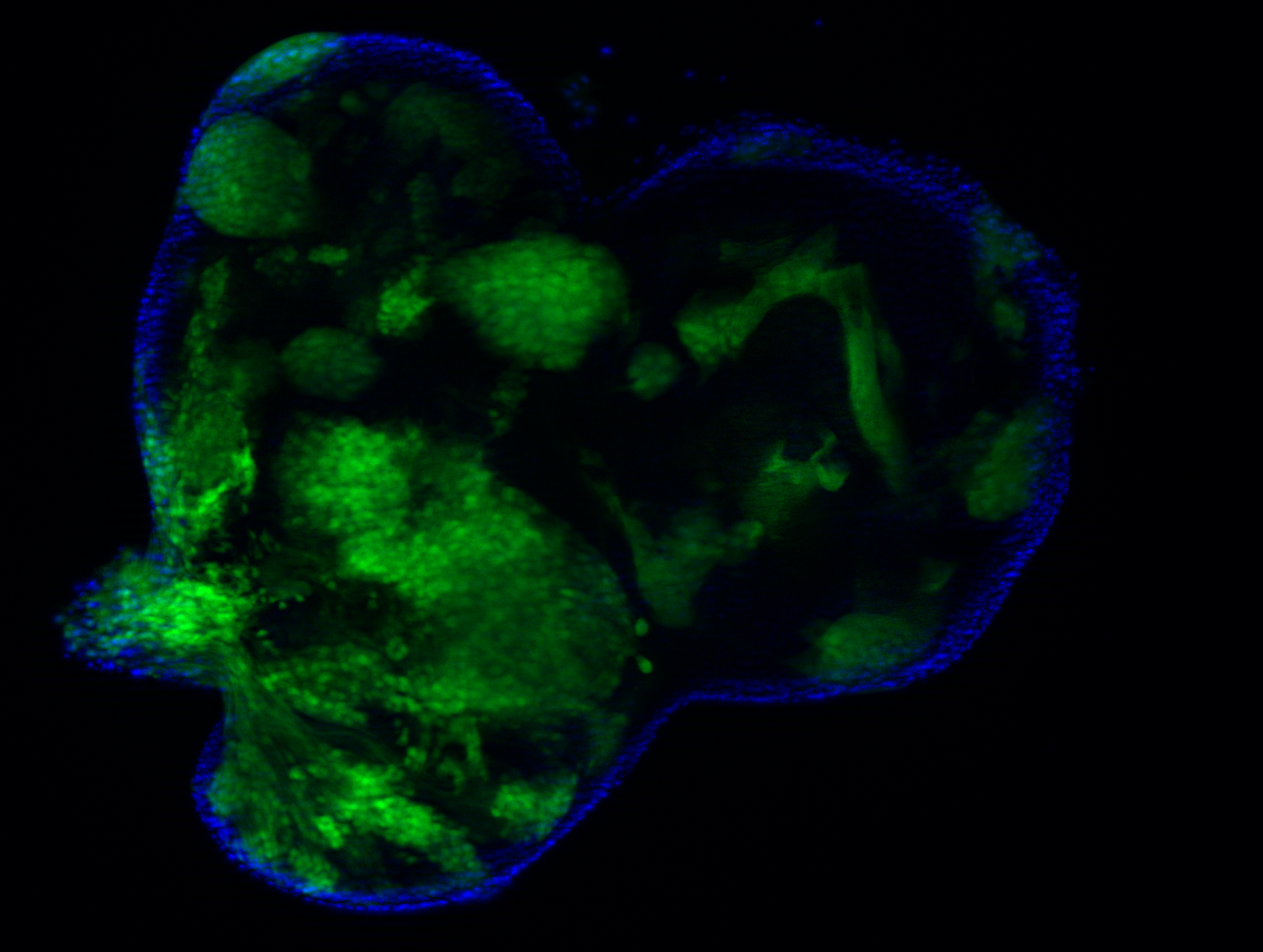

Supplement: Supplementary file 8 — Source data Fig. 4 [file 44318_2025_547_MOESM8_ESM.zip › Figure 4C/8 original image.tif]

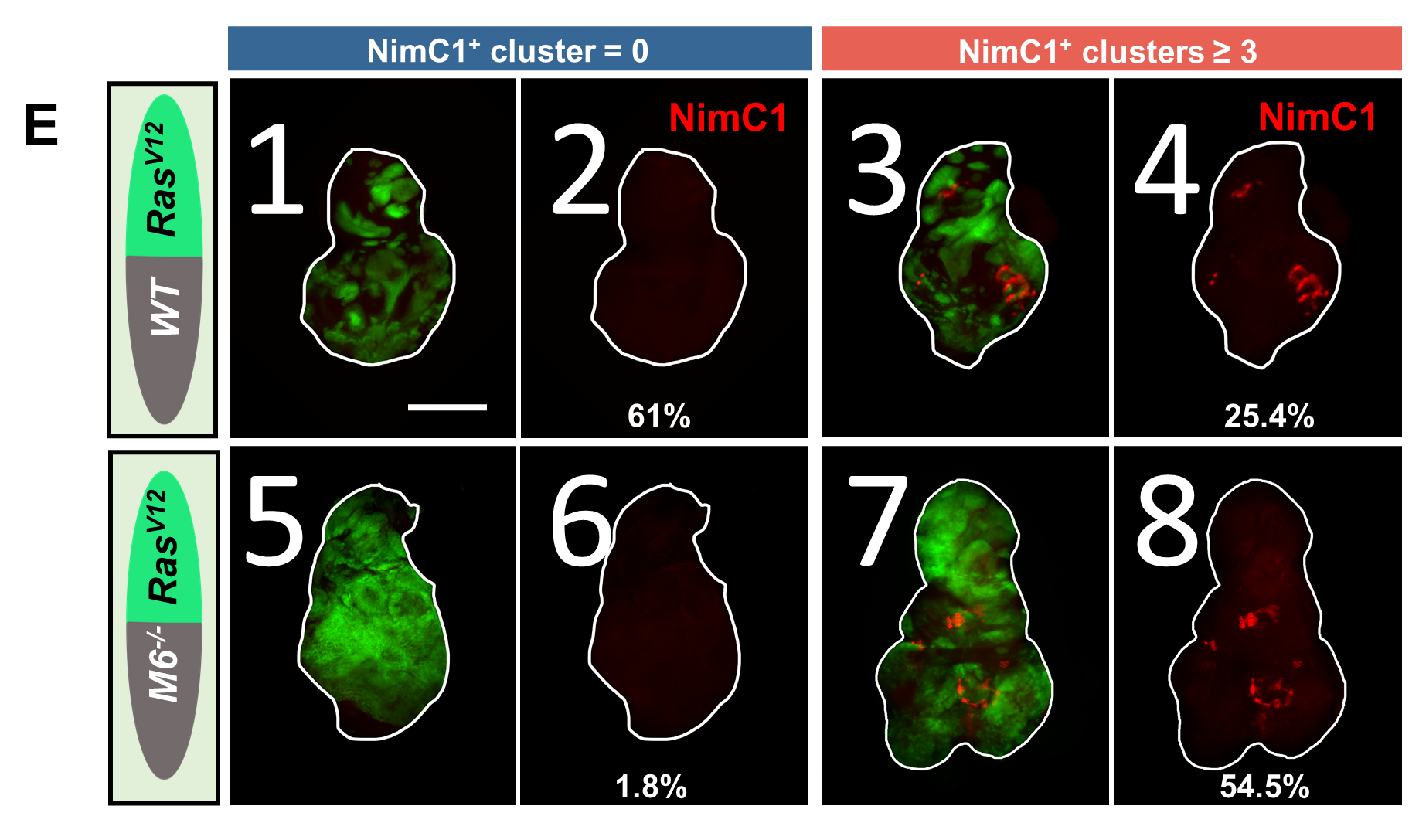

Supplement: Supplementary file 9 — Source data Fig. 5 [file 44318_2025_547_MOESM9_ESM.zip › Figure 5E/0 paper Figure 5E with provided image sequence.tif]

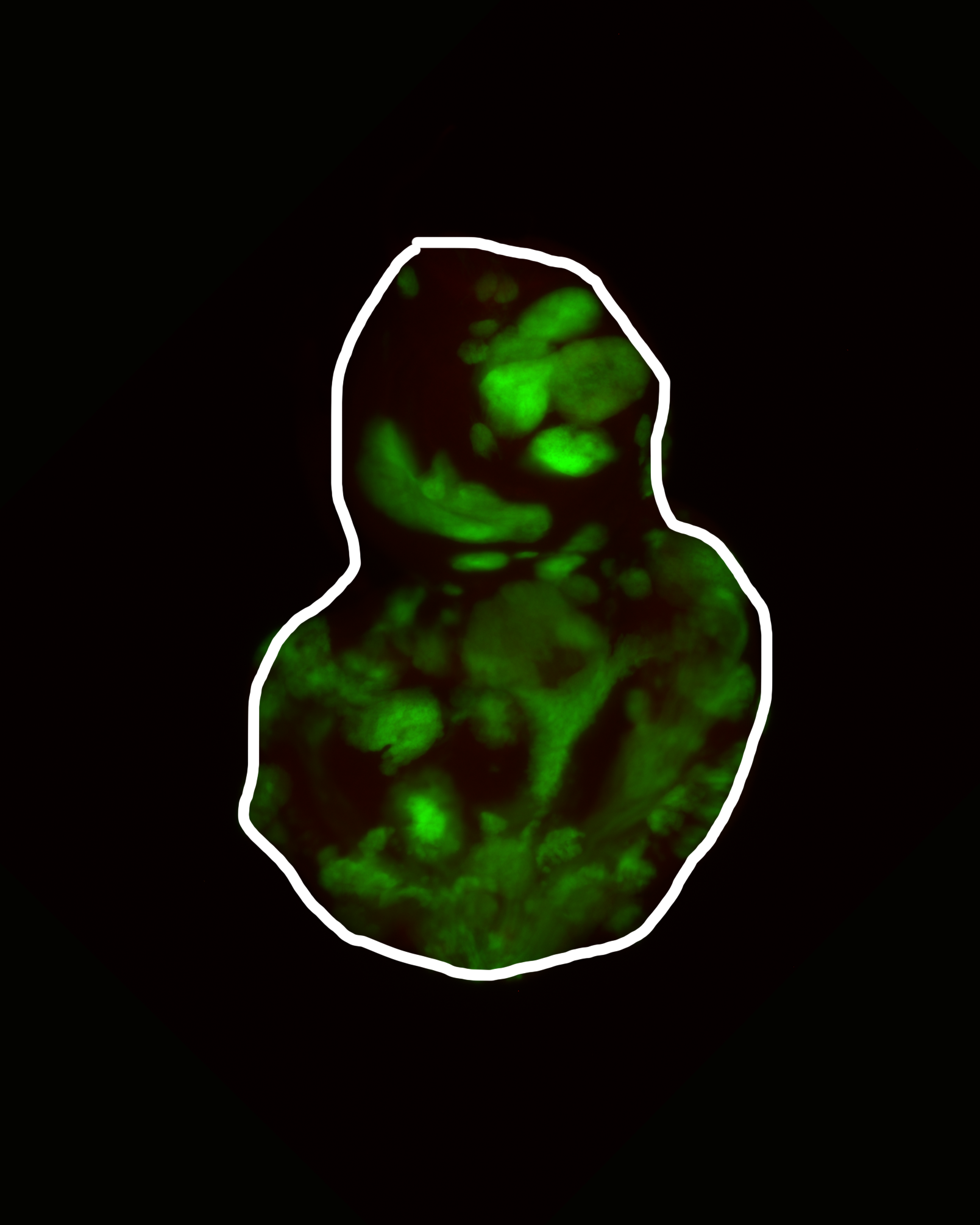

Supplement: Supplementary file 9 — Source data Fig. 5 [file 44318_2025_547_MOESM9_ESM.zip › Figure 5E/1-1- rotated and cut image with border line.tif]

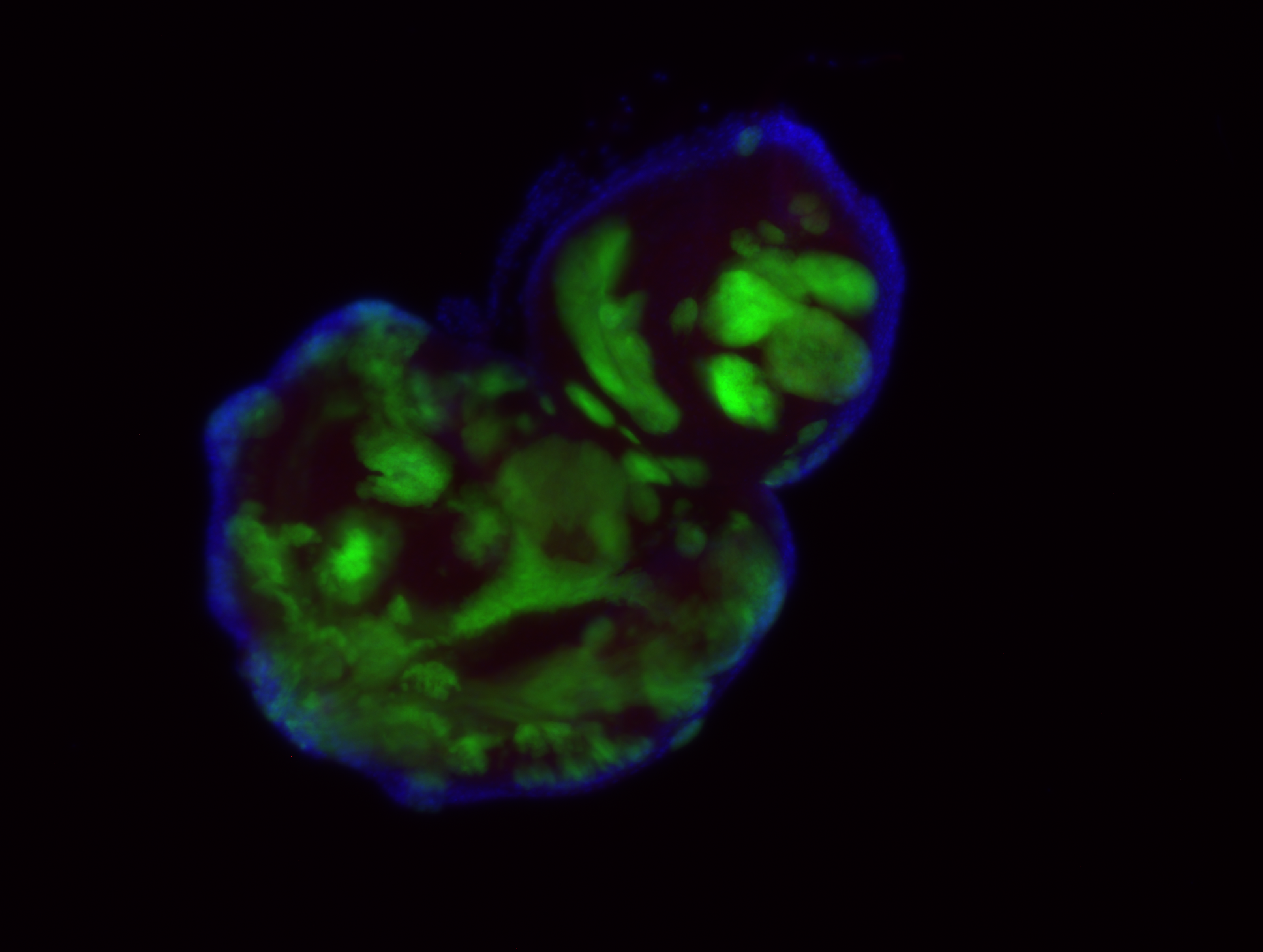

Supplement: Supplementary file 9 — Source data Fig. 5 [file 44318_2025_547_MOESM9_ESM.zip › Figure 5E/1-2 original image.tif]
