# Supplementary figures and images for: Hemocytes facilitate interclonal cooperation-induced tumor malignancy by hijacking the innate immune system in Drosophila (part 3 of 4)
Source: EMBO J. 2025 Aug 22;44(19):5394–428. doi: 10.1038/s44318-025-00547-5 (PMC12489090; doi:10.1038/s44318-025-00547-5)

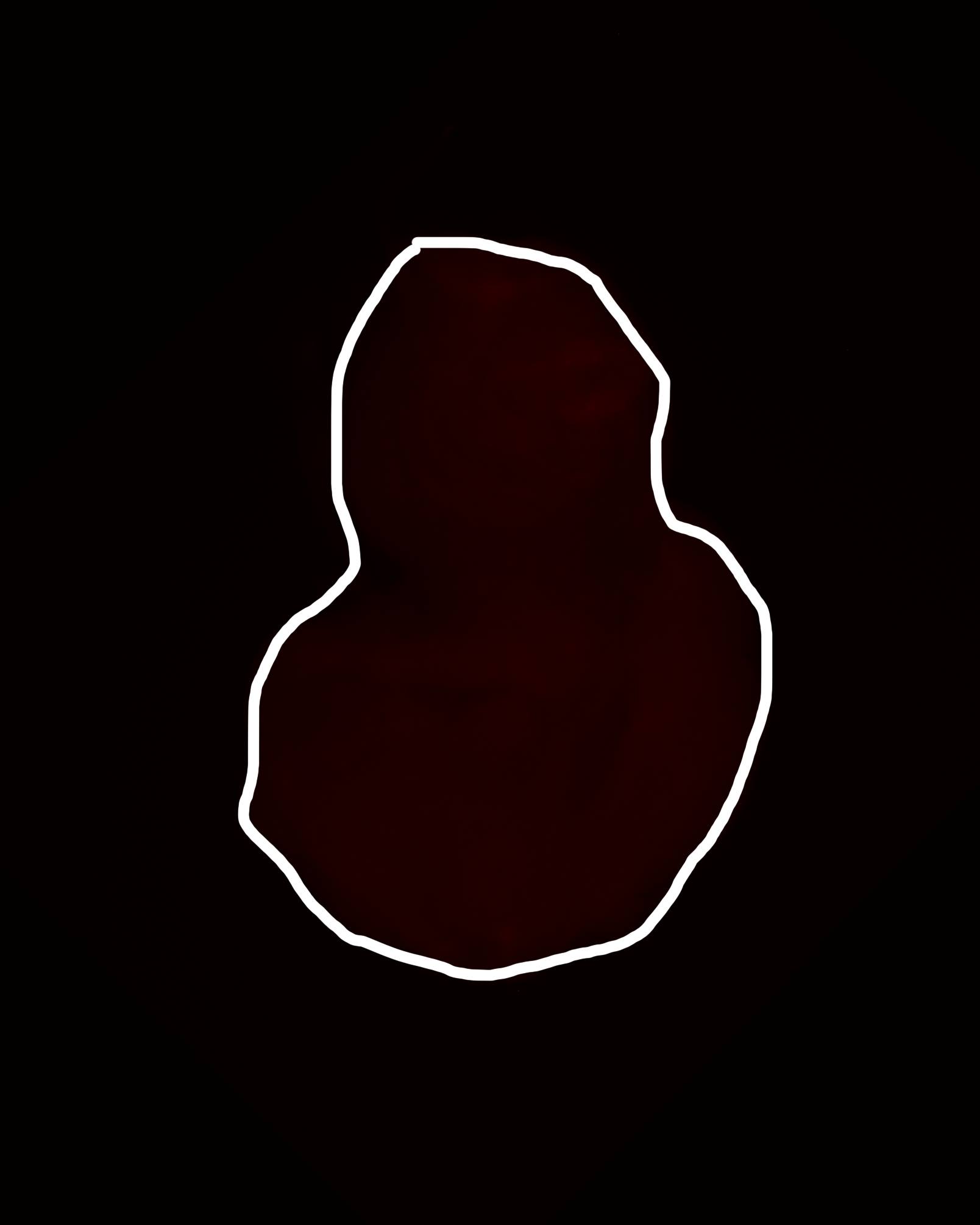

Supplement: Supplementary file 9 — Source data Fig. 5 [file 44318_2025_547_MOESM9_ESM.zip › Figure 5E/2-1- rotated and cut image with border line.tif]

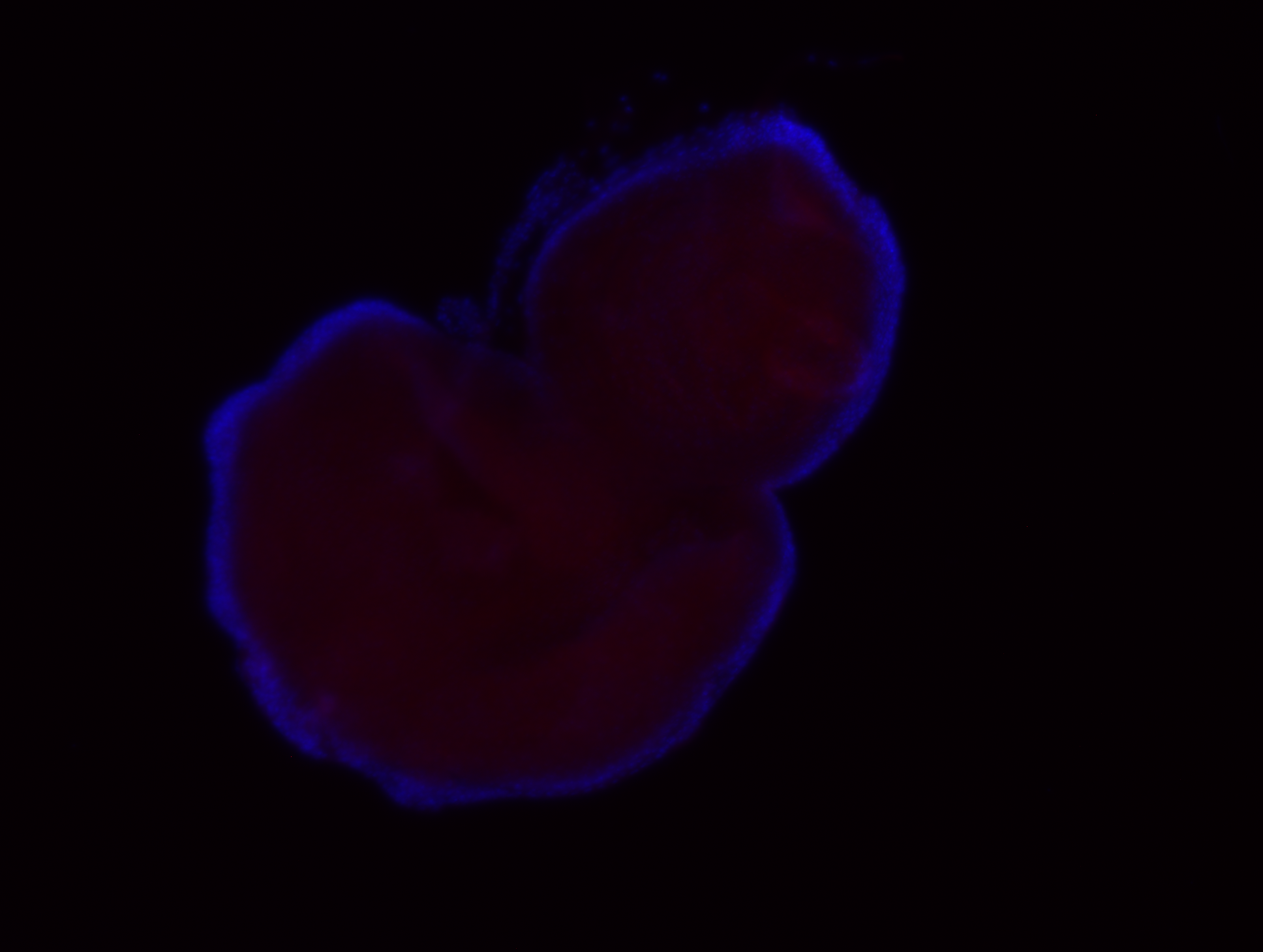

Supplement: Supplementary file 9 — Source data Fig. 5 [file 44318_2025_547_MOESM9_ESM.zip › Figure 5E/2-2 original image.tif]

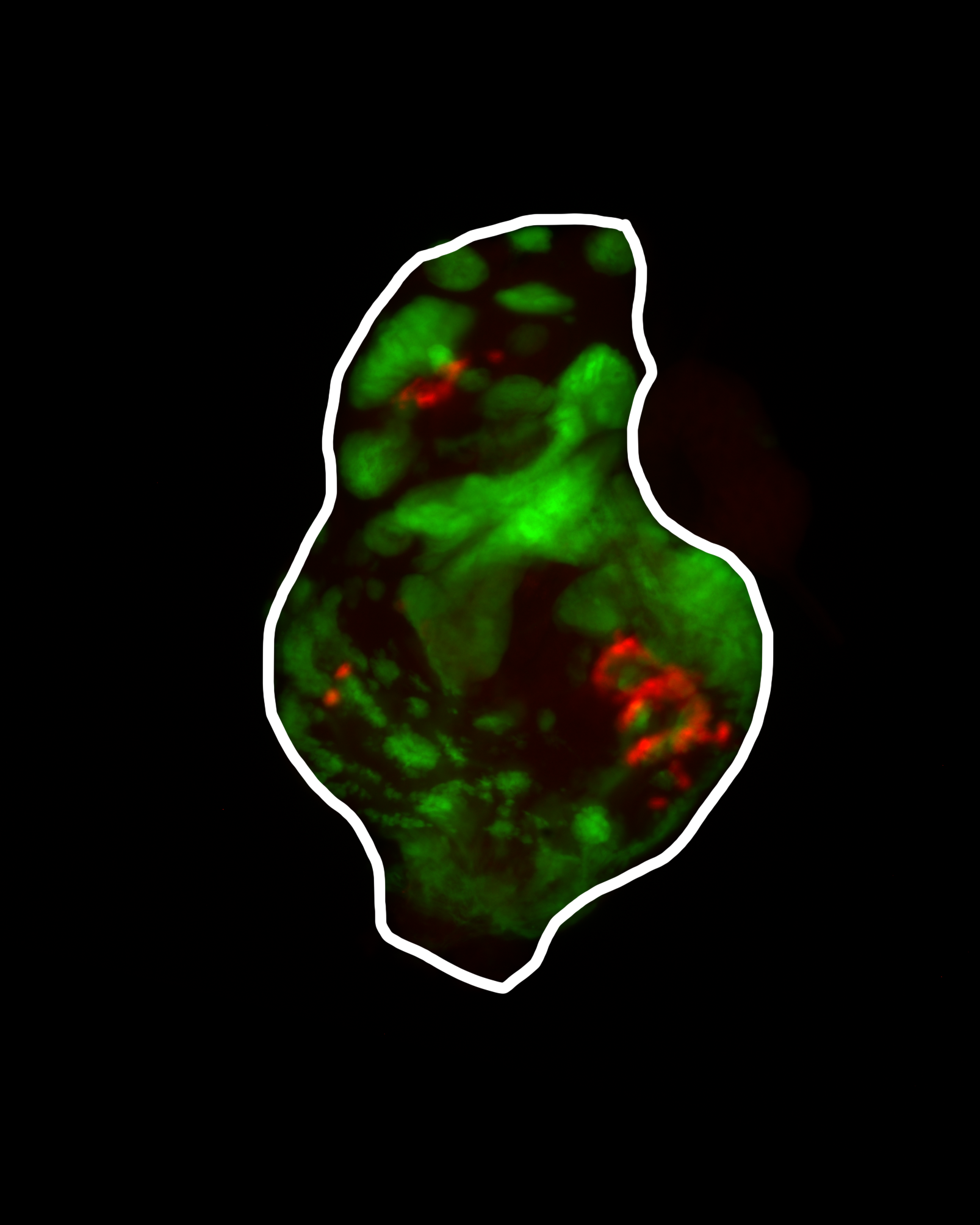

Supplement: Supplementary file 9 — Source data Fig. 5 [file 44318_2025_547_MOESM9_ESM.zip › Figure 5E/3-1 rotated and cut image with border line.tif]

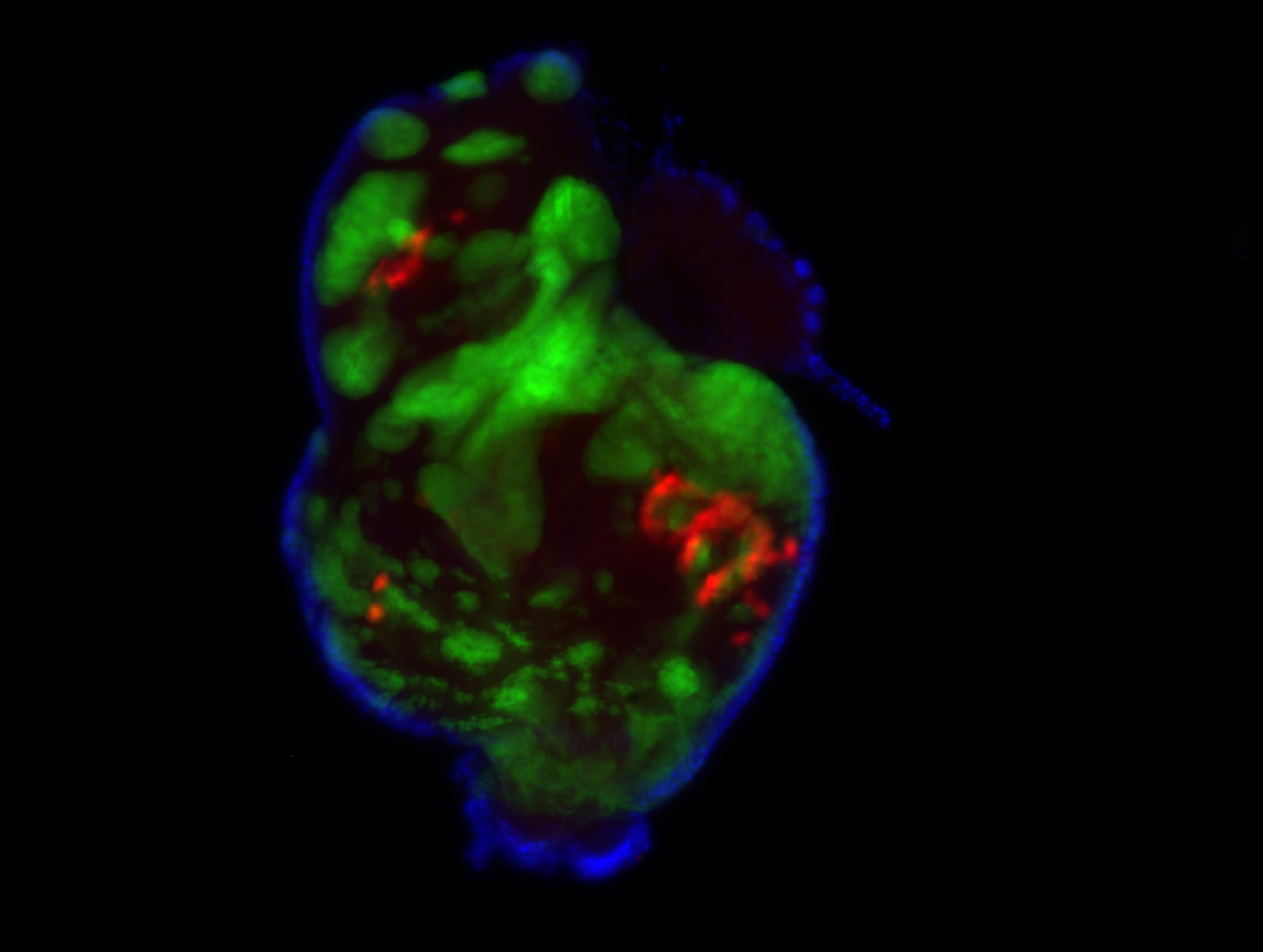

Supplement: Supplementary file 9 — Source data Fig. 5 [file 44318_2025_547_MOESM9_ESM.zip › Figure 5E/3-2 original image.tif]

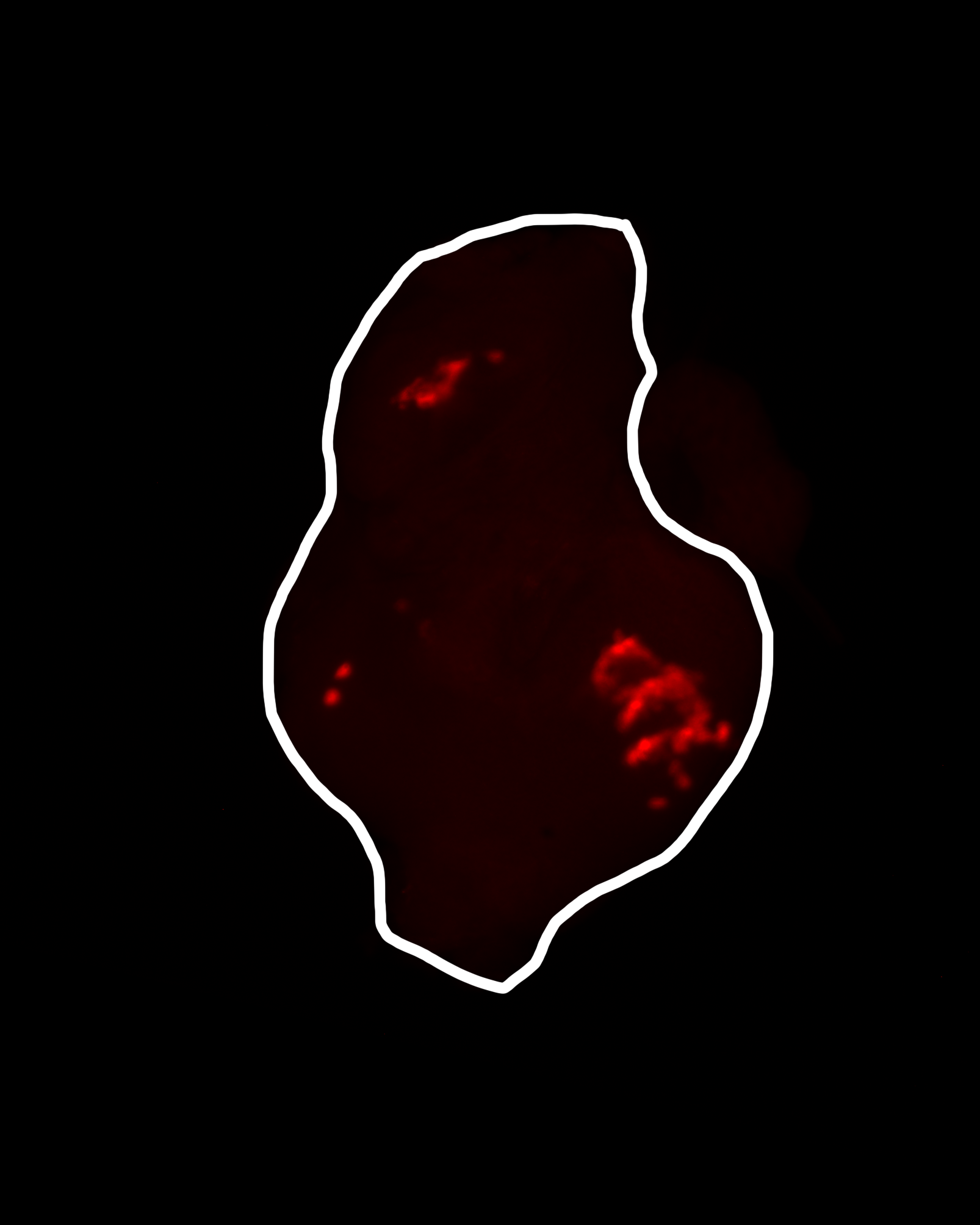

Supplement: Supplementary file 9 — Source data Fig. 5 [file 44318_2025_547_MOESM9_ESM.zip › Figure 5E/4-1 rotated and cut image with border line.tif]

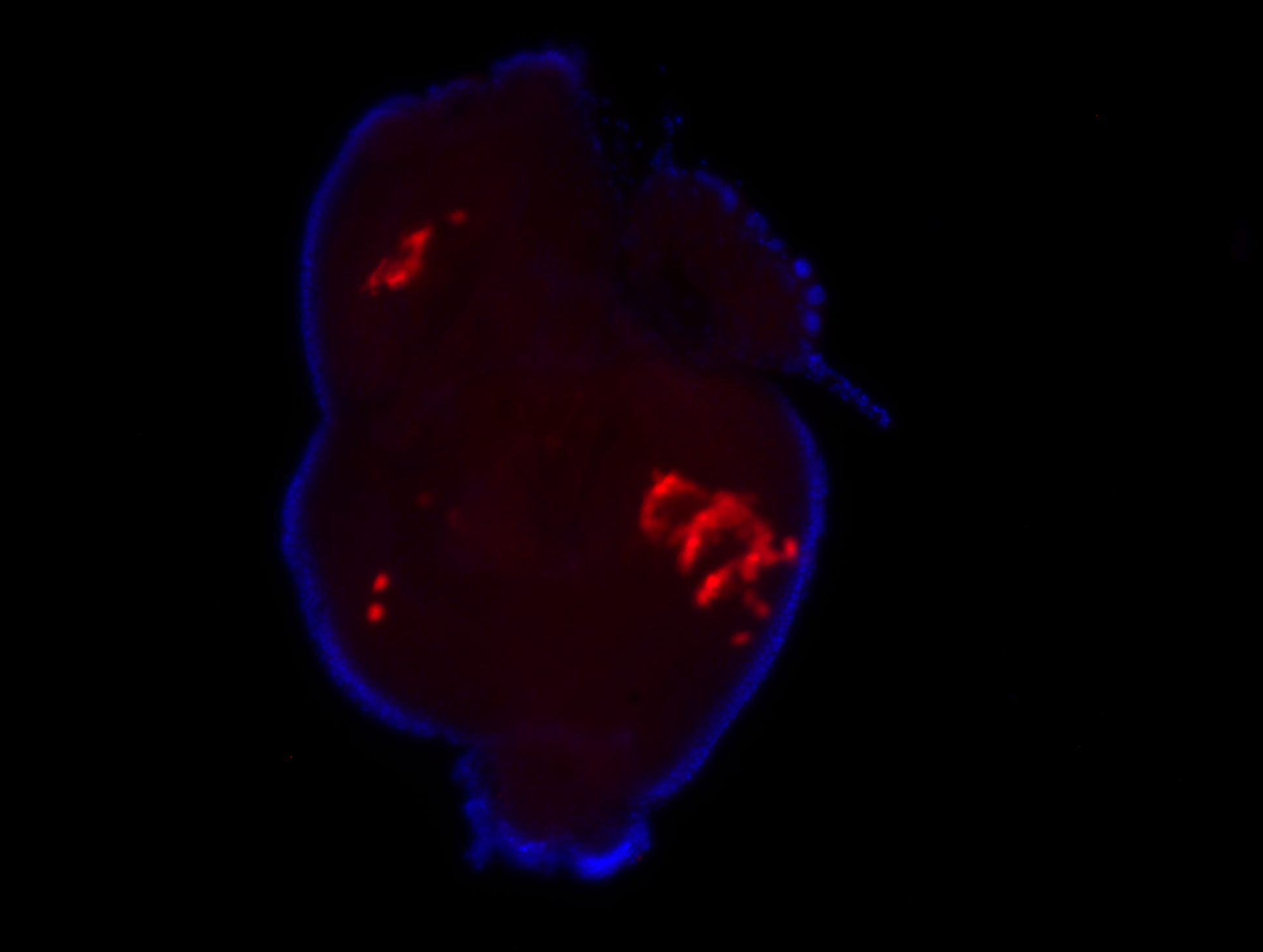

Supplement: Supplementary file 9 — Source data Fig. 5 [file 44318_2025_547_MOESM9_ESM.zip › Figure 5E/4-2 original image.tif]

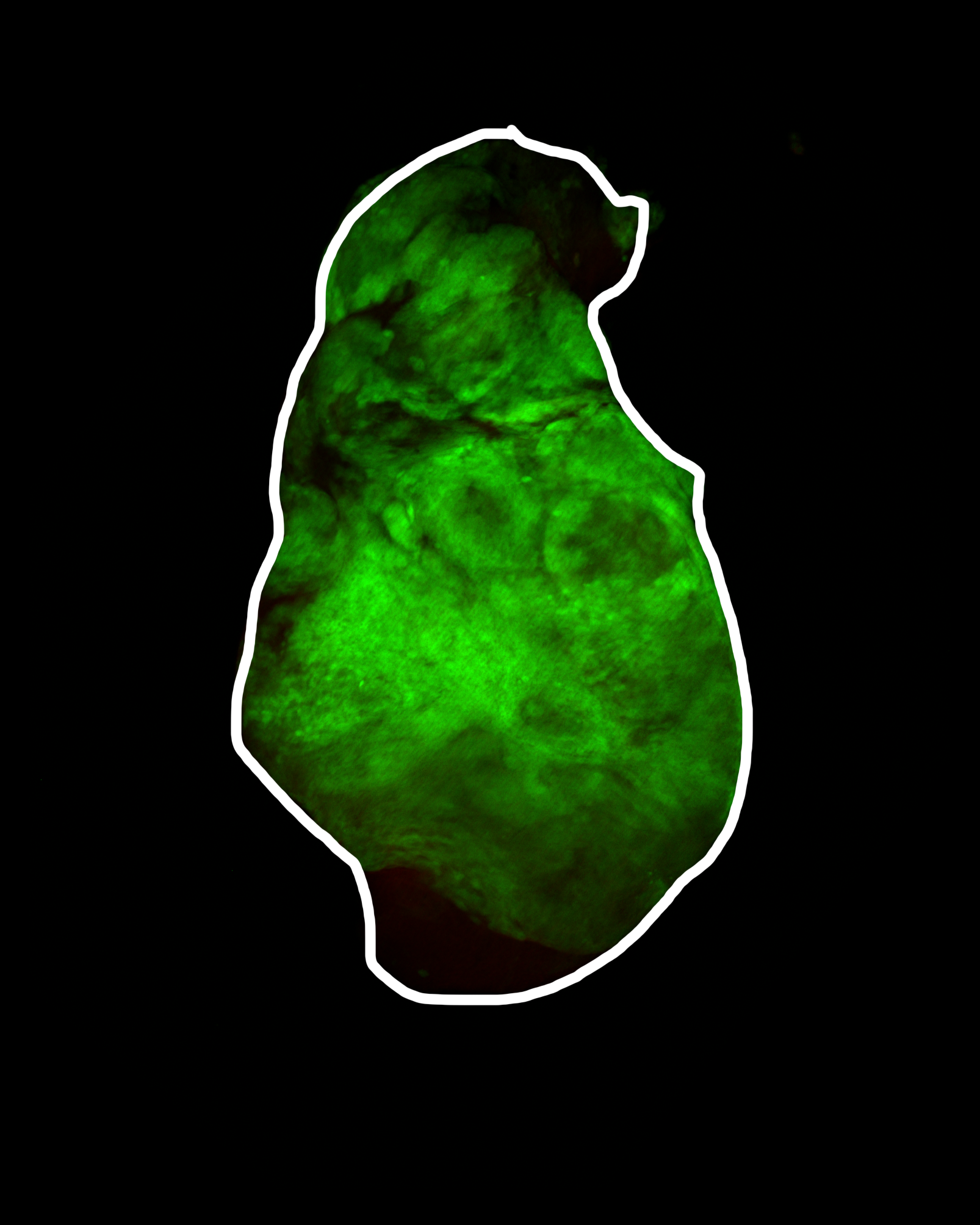

Supplement: Supplementary file 9 — Source data Fig. 5 [file 44318_2025_547_MOESM9_ESM.zip › Figure 5E/5-1 rotated and cut image with border line.tif]

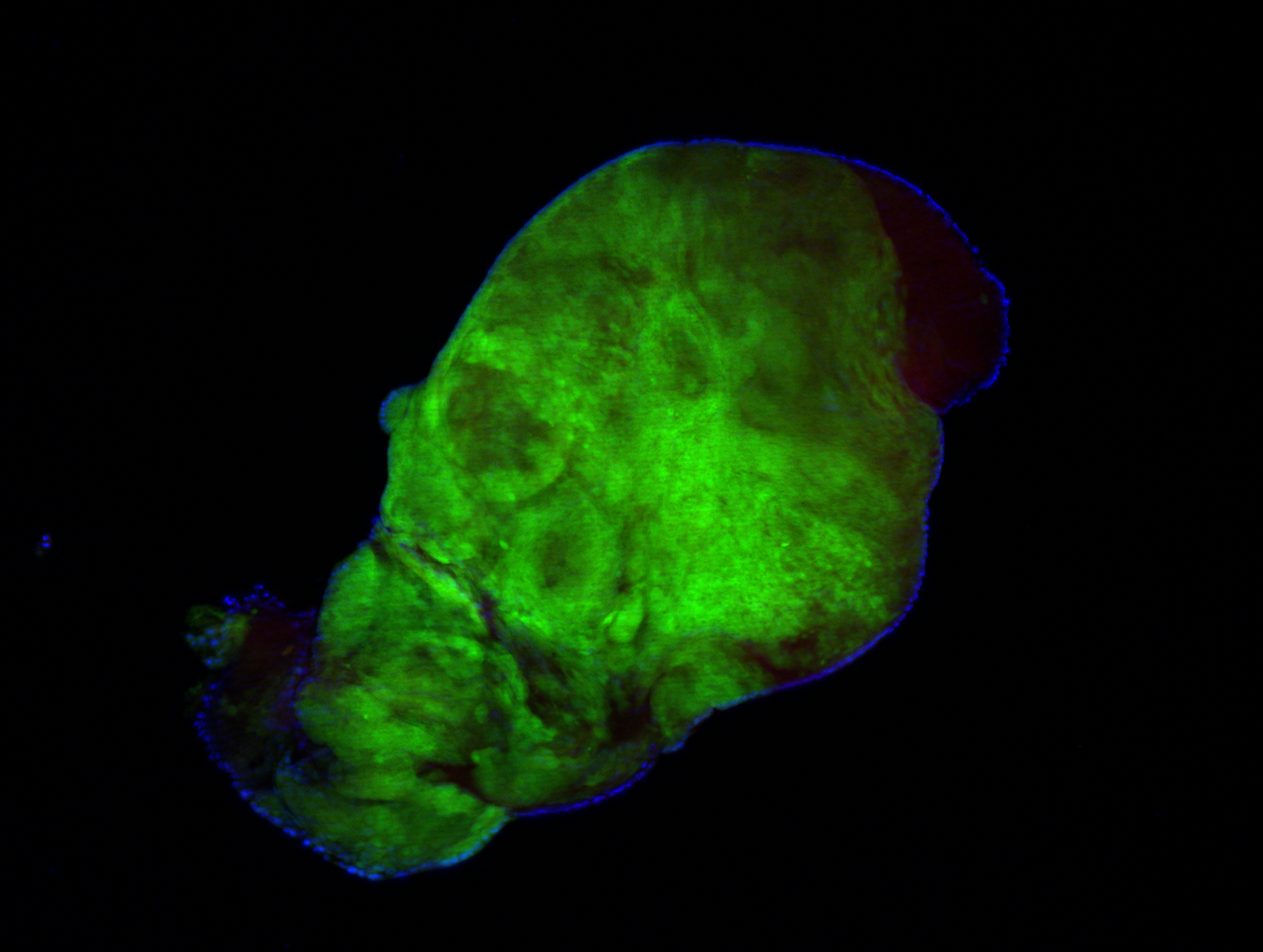

Supplement: Supplementary file 9 — Source data Fig. 5 [file 44318_2025_547_MOESM9_ESM.zip › Figure 5E/5-2 original image.tif]

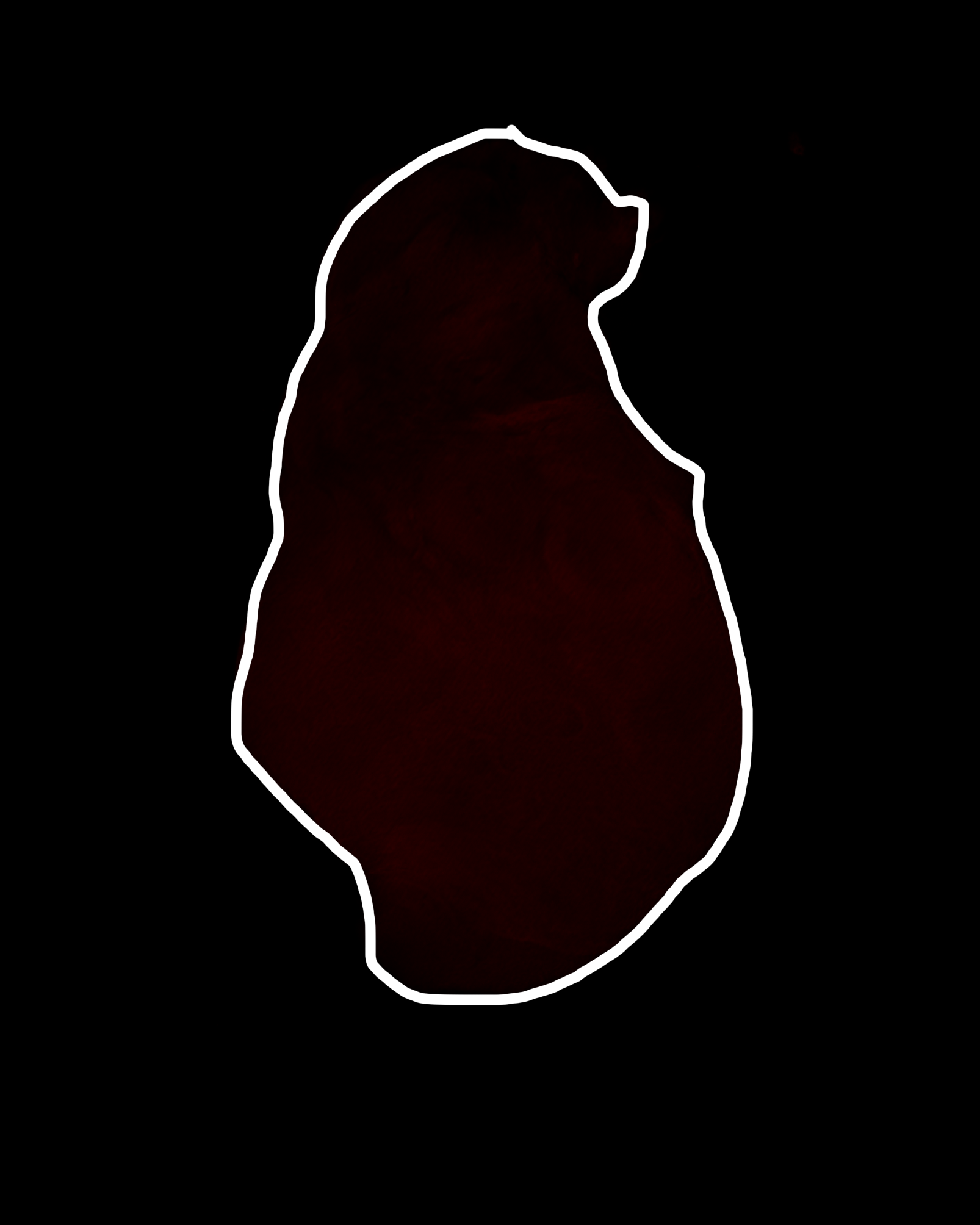

Supplement: Supplementary file 9 — Source data Fig. 5 [file 44318_2025_547_MOESM9_ESM.zip › Figure 5E/6-1 rotated and cut image with border line.tif]

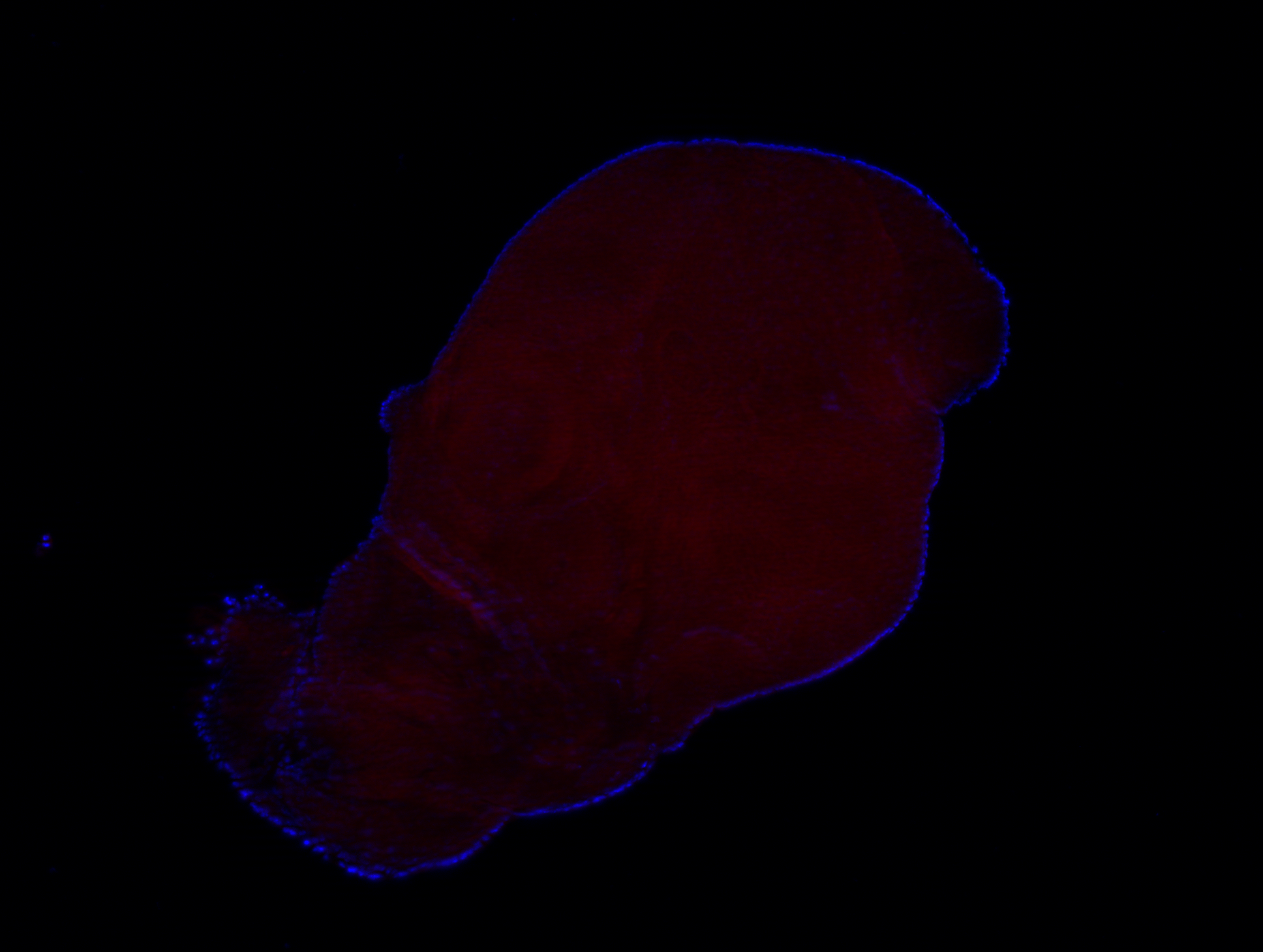

Supplement: Supplementary file 9 — Source data Fig. 5 [file 44318_2025_547_MOESM9_ESM.zip › Figure 5E/6-2 original image.tif]

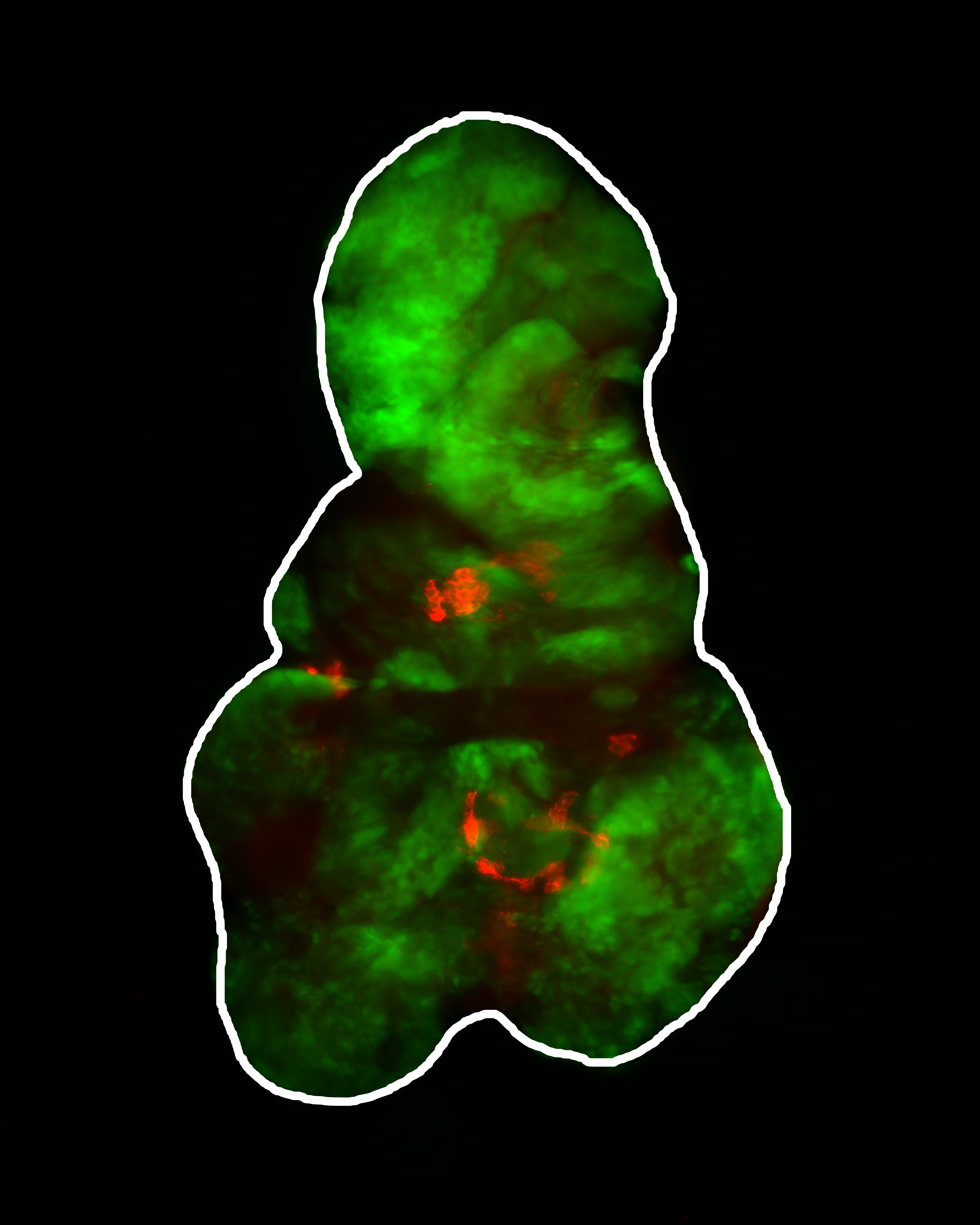

Supplement: Supplementary file 9 — Source data Fig. 5 [file 44318_2025_547_MOESM9_ESM.zip › Figure 5E/7-1 rotated and cut image with border line.tif]

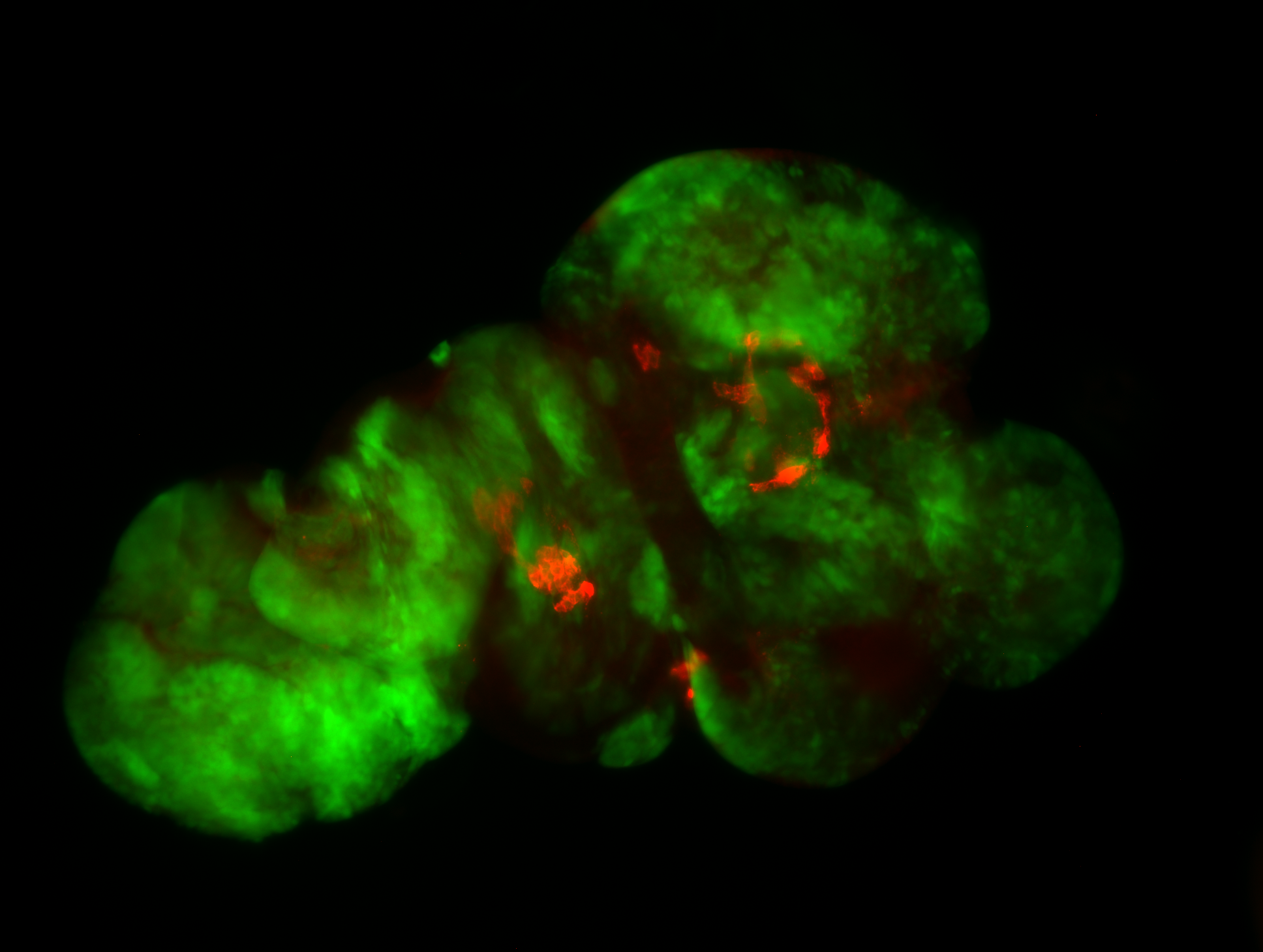

Supplement: Supplementary file 9 — Source data Fig. 5 [file 44318_2025_547_MOESM9_ESM.zip › Figure 5E/7-2 original image.tif]

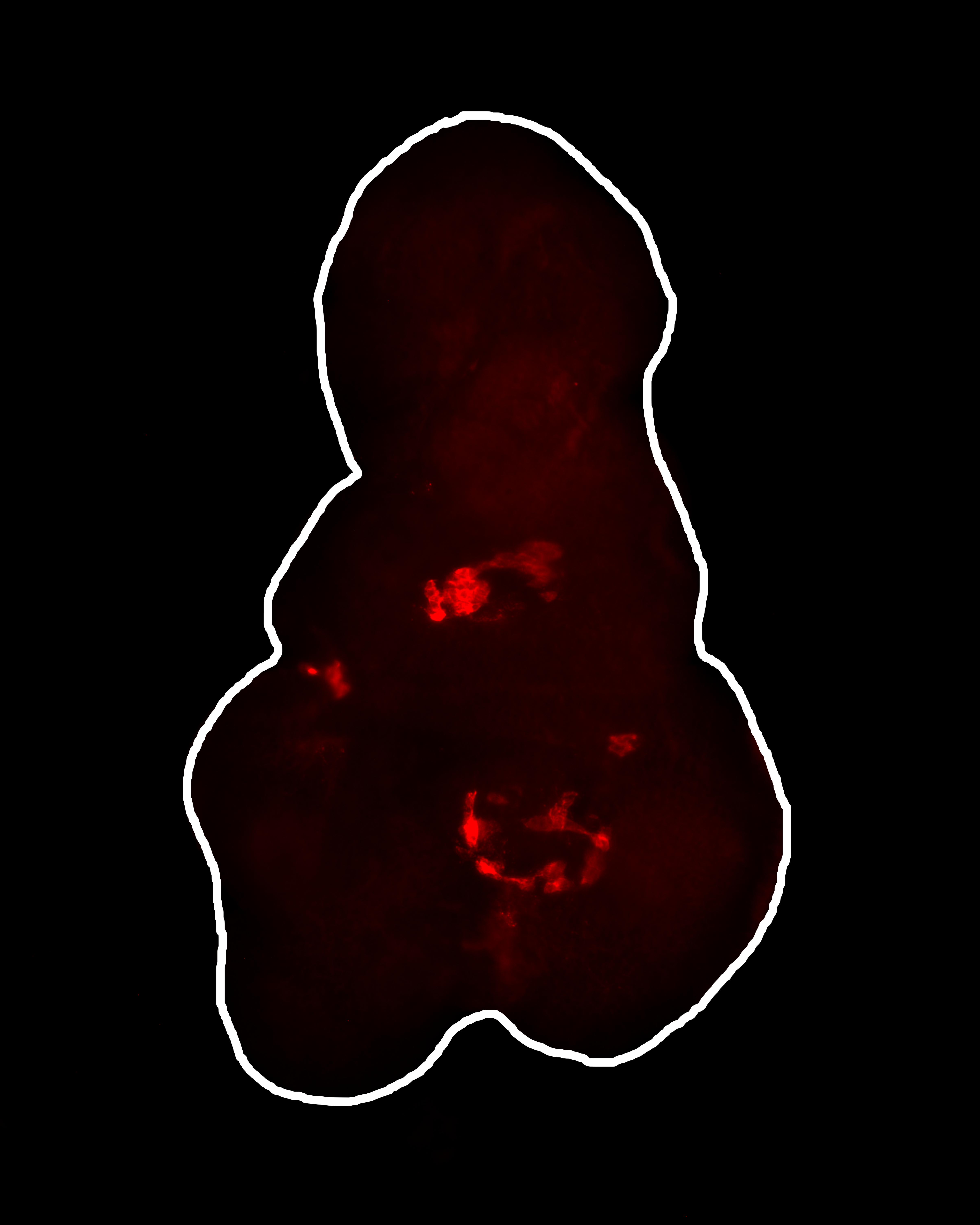

Supplement: Supplementary file 9 — Source data Fig. 5 [file 44318_2025_547_MOESM9_ESM.zip › Figure 5E/8-1 rotated and cut image with border line.tif]

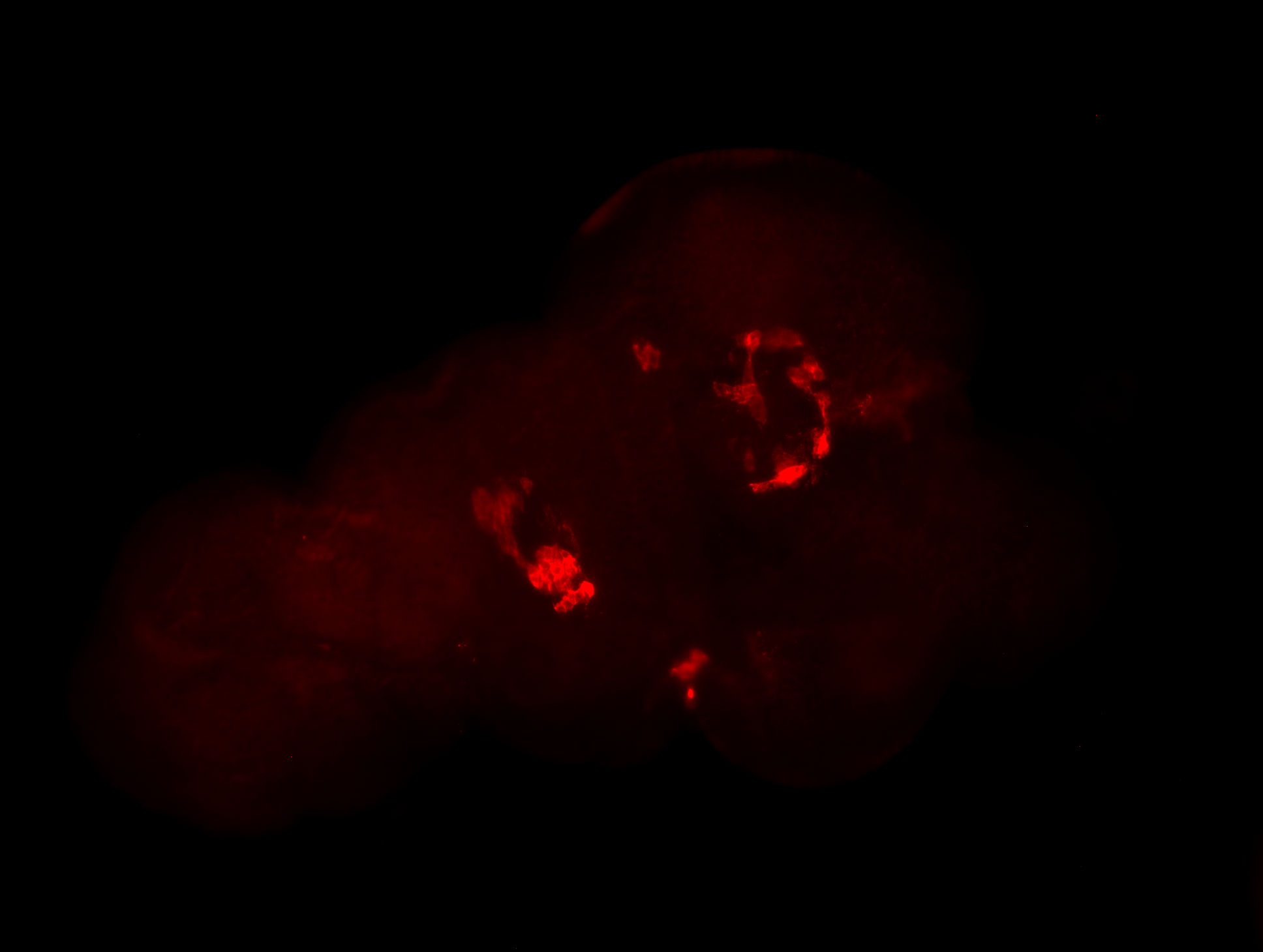

Supplement: Supplementary file 9 — Source data Fig. 5 [file 44318_2025_547_MOESM9_ESM.zip › Figure 5E/8-2 original image.tif]

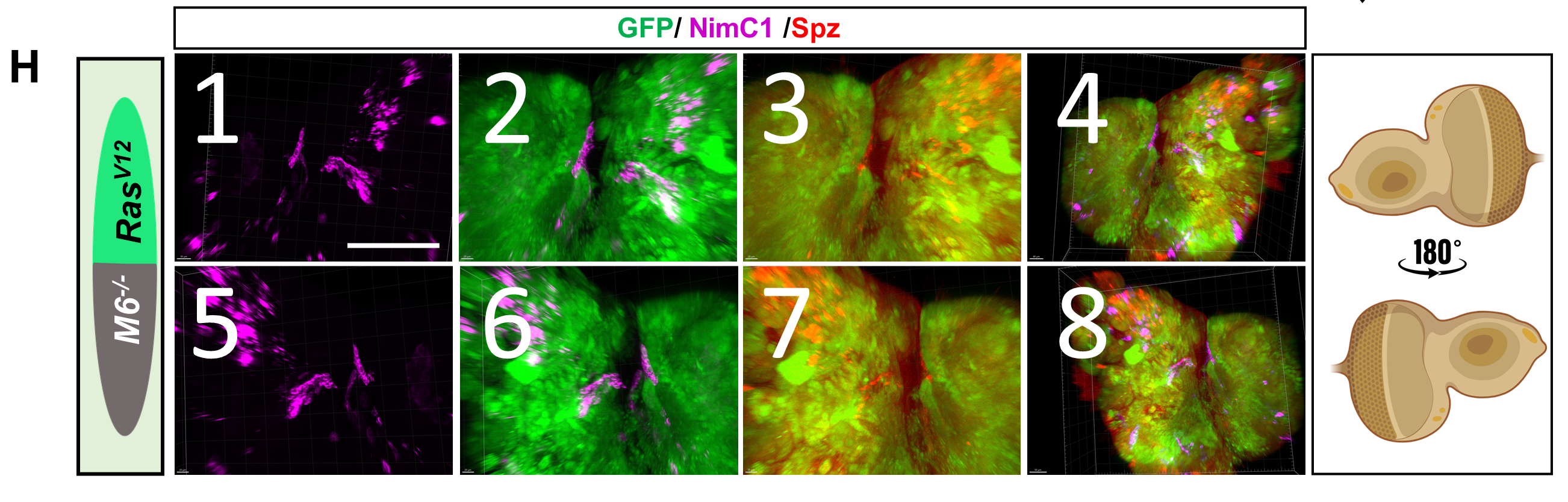

Supplement: Supplementary file 9 — Source data Fig. 5 [file 44318_2025_547_MOESM9_ESM.zip › Figure 5H/0 paper Figure 5H with provided image sequence.tif]

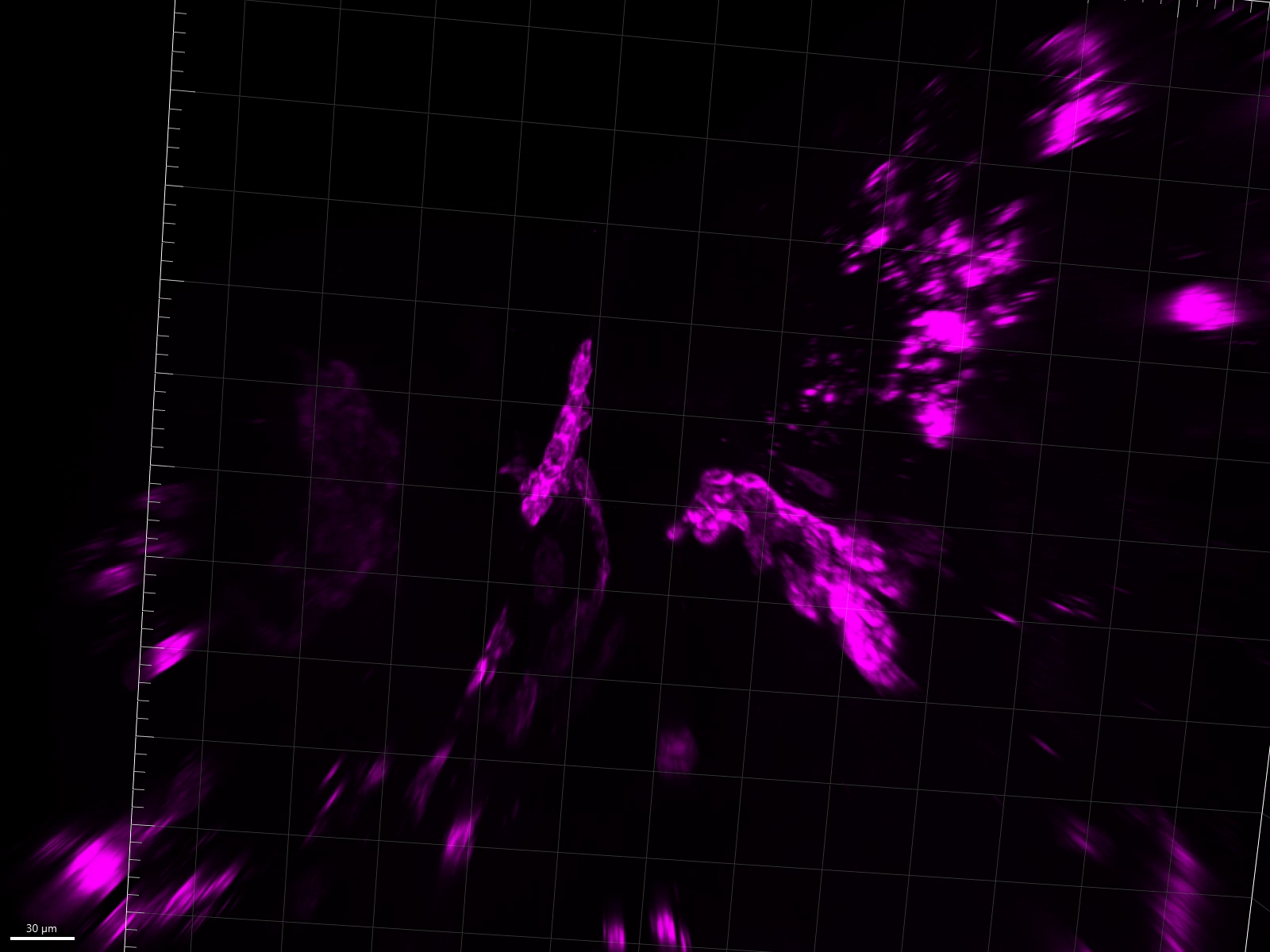

Supplement: Supplementary file 9 — Source data Fig. 5 [file 44318_2025_547_MOESM9_ESM.zip › Figure 5H/1 original image.tif]

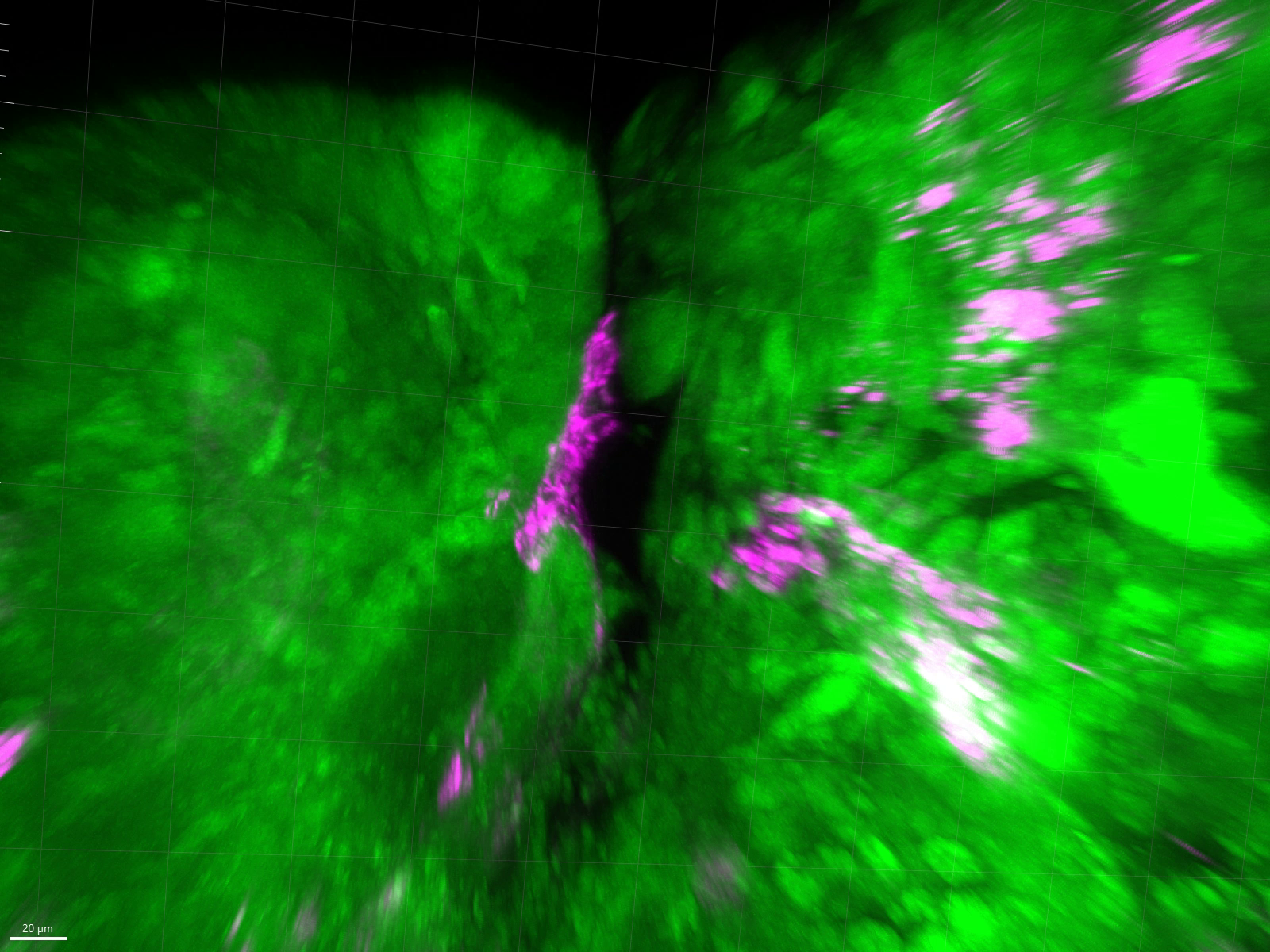

Supplement: Supplementary file 9 — Source data Fig. 5 [file 44318_2025_547_MOESM9_ESM.zip › Figure 5H/2 original image.tif]

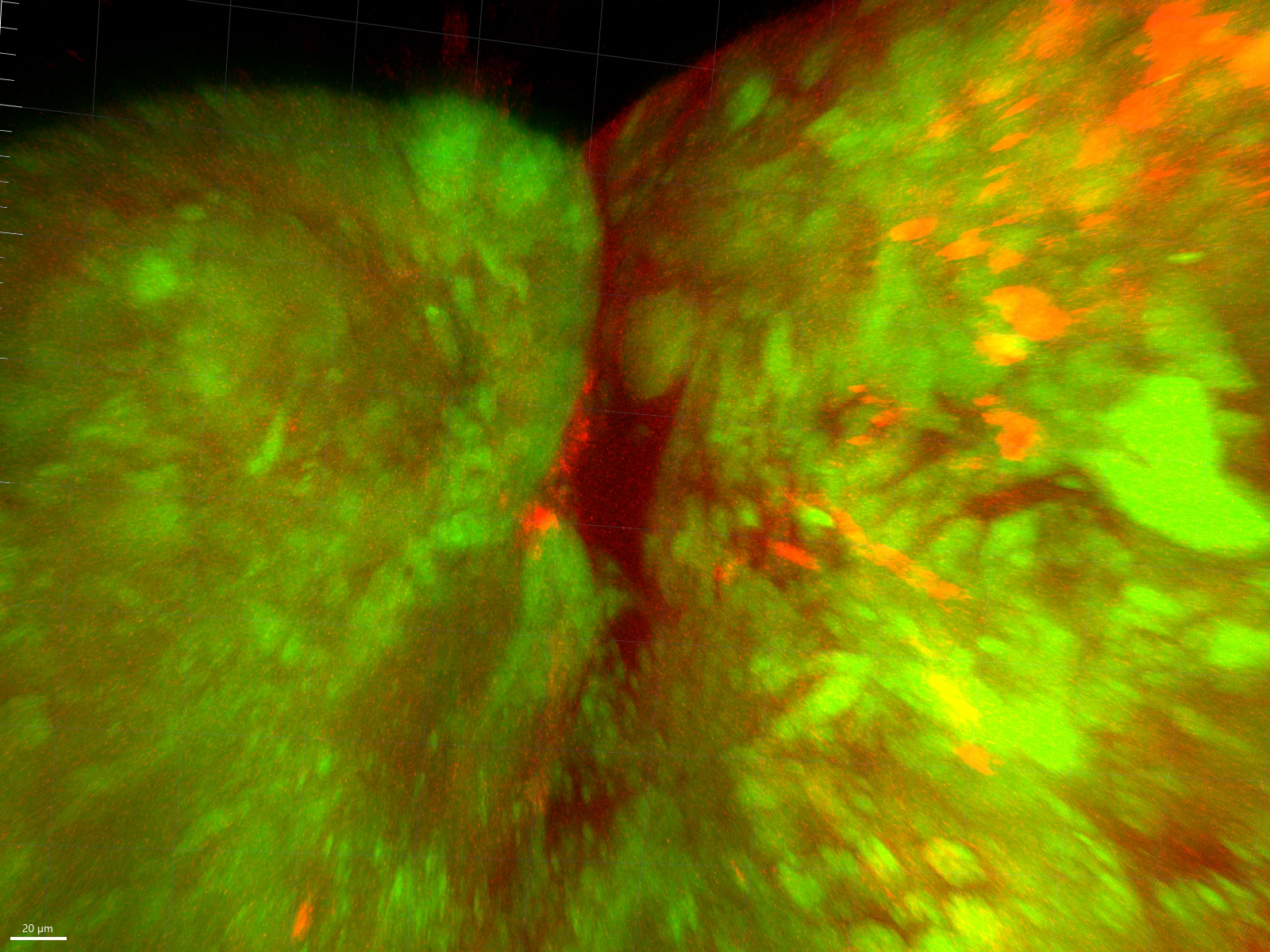

Supplement: Supplementary file 9 — Source data Fig. 5 [file 44318_2025_547_MOESM9_ESM.zip › Figure 5H/3 original image.tif]

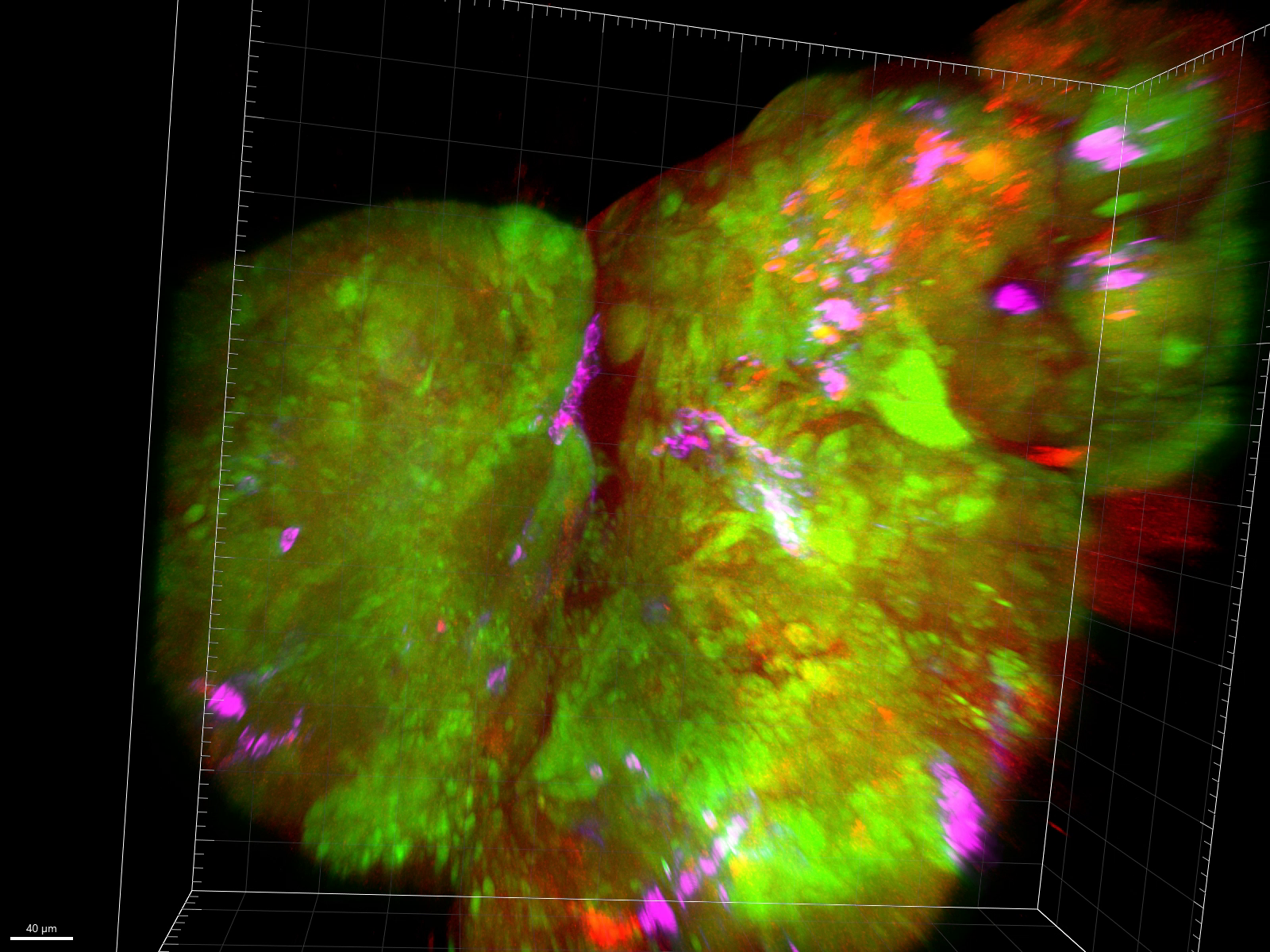

Supplement: Supplementary file 9 — Source data Fig. 5 [file 44318_2025_547_MOESM9_ESM.zip › Figure 5H/4 original image.tif]

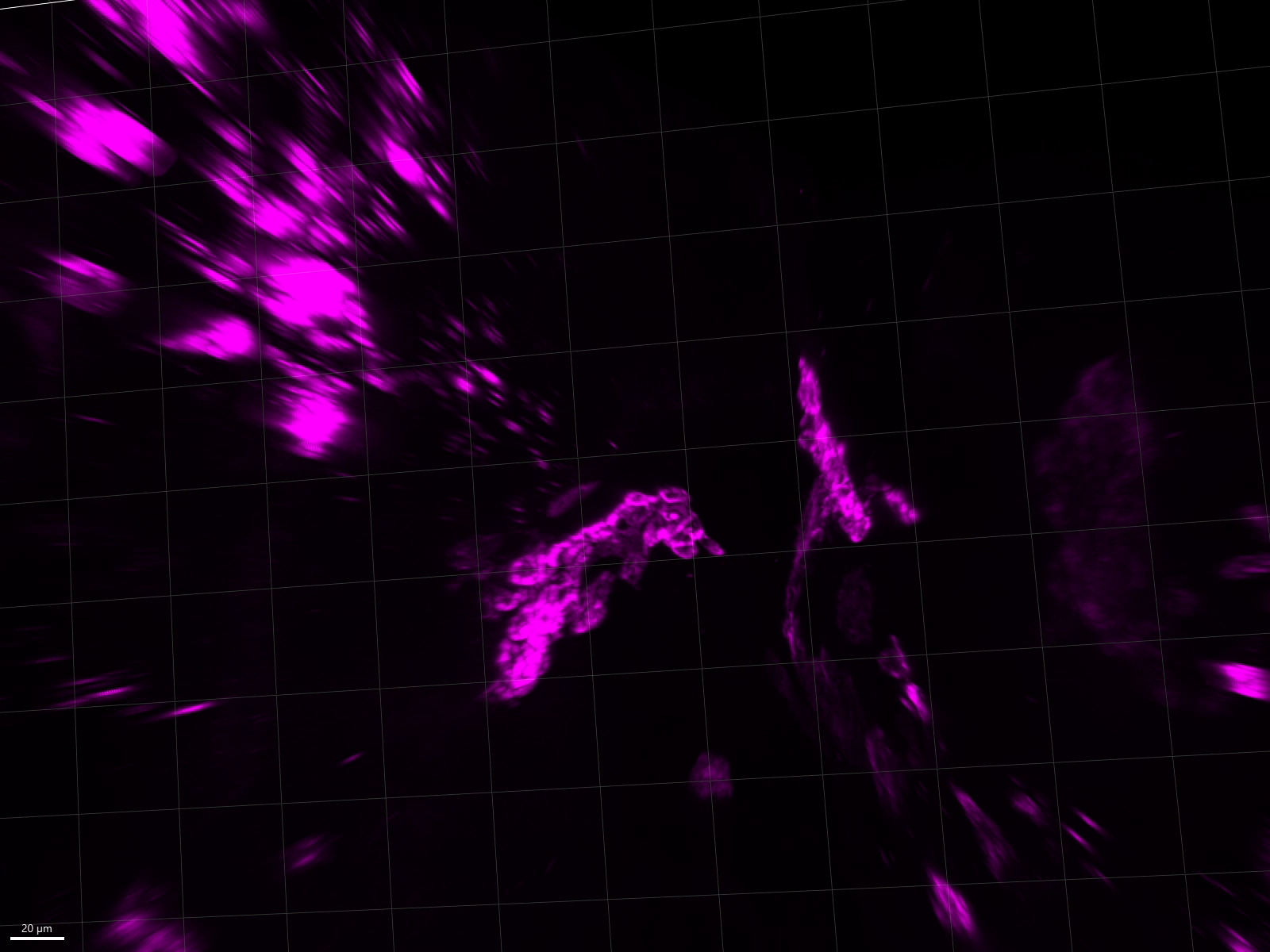

Supplement: Supplementary file 9 — Source data Fig. 5 [file 44318_2025_547_MOESM9_ESM.zip › Figure 5H/5 original image.tif]

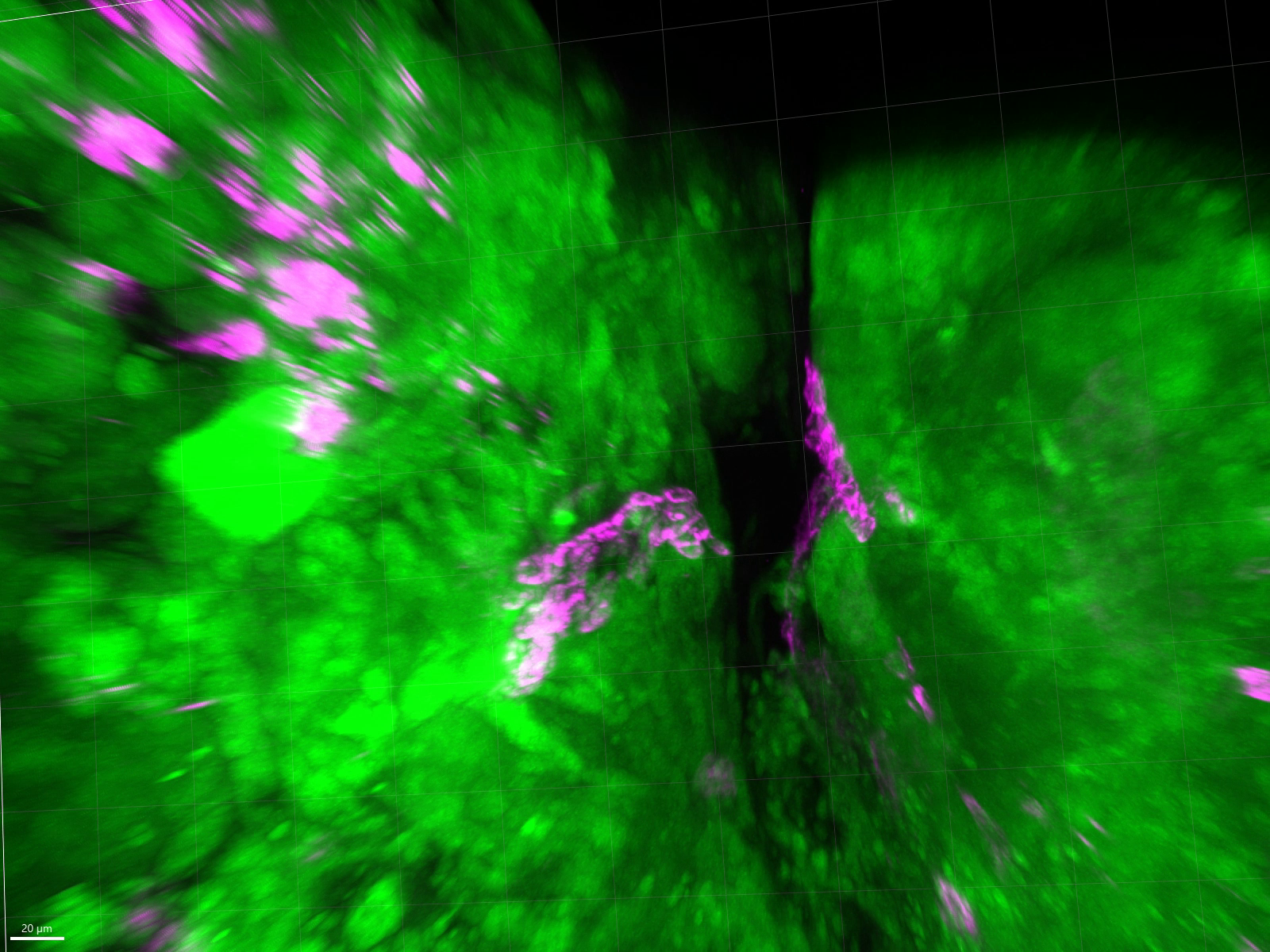

Supplement: Supplementary file 9 — Source data Fig. 5 [file 44318_2025_547_MOESM9_ESM.zip › Figure 5H/6 original image.tif]

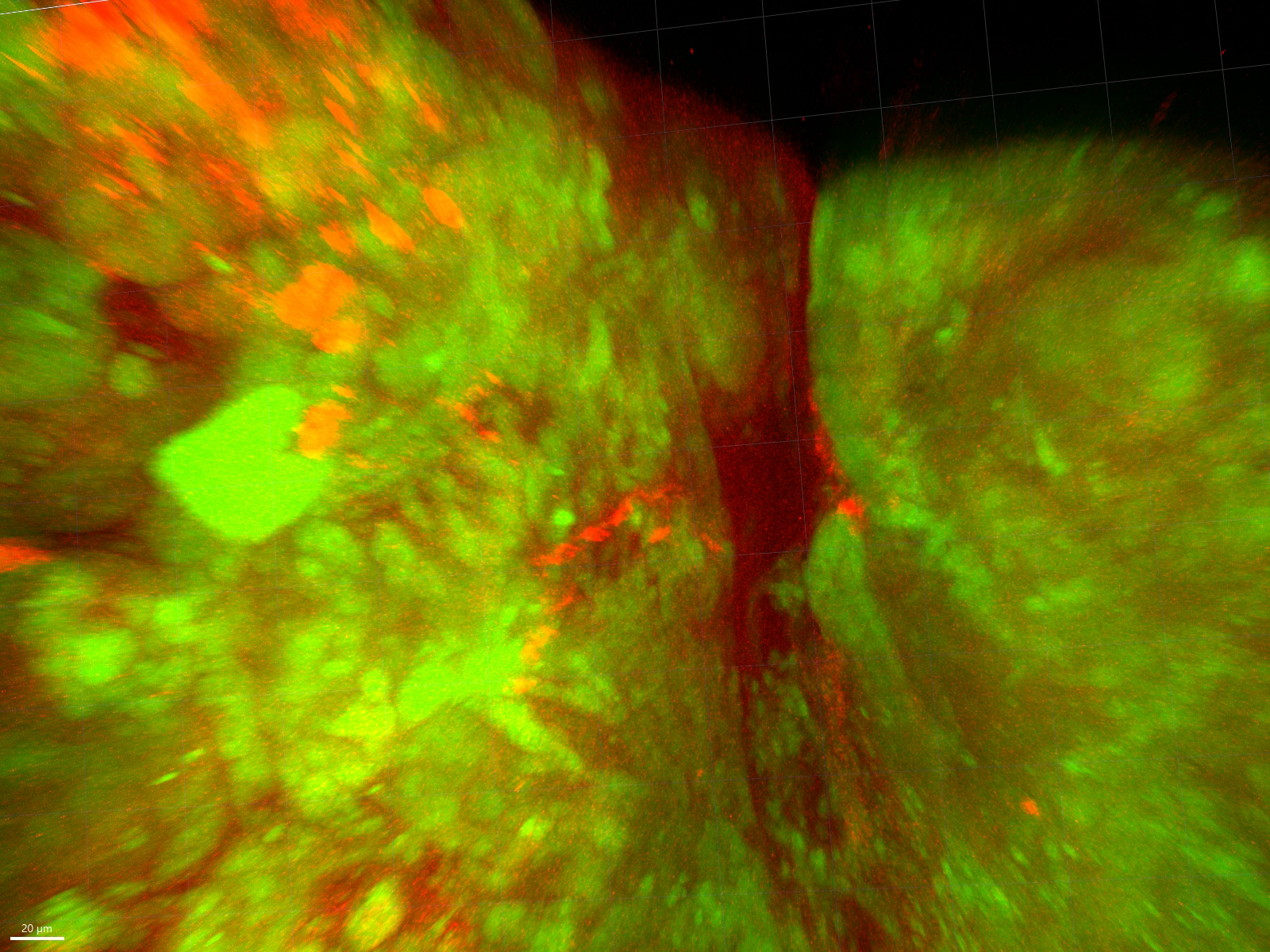

Supplement: Supplementary file 9 — Source data Fig. 5 [file 44318_2025_547_MOESM9_ESM.zip › Figure 5H/7 original image.tif]

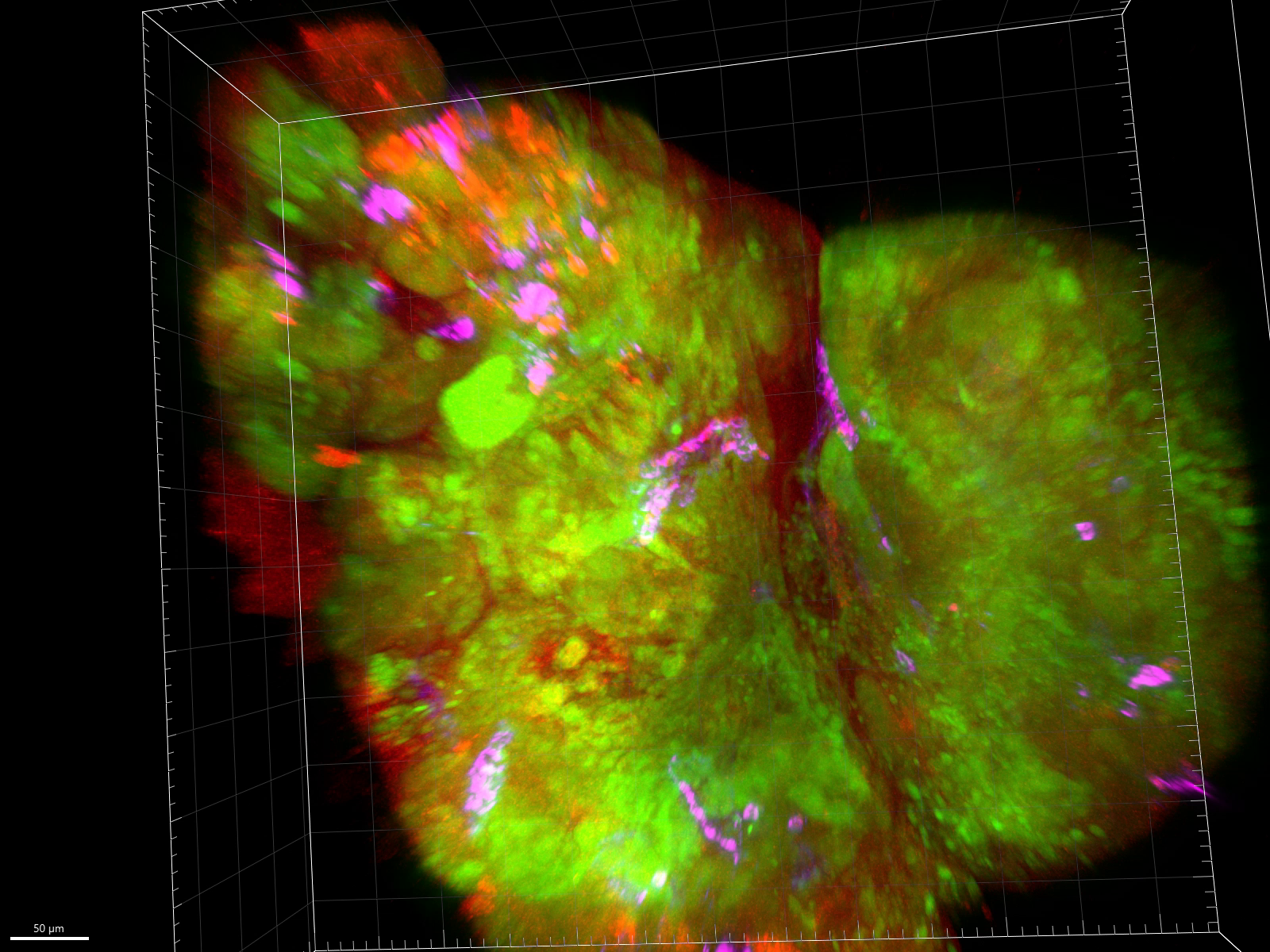

Supplement: Supplementary file 9 — Source data Fig. 5 [file 44318_2025_547_MOESM9_ESM.zip › Figure 5H/8 original image.tif]

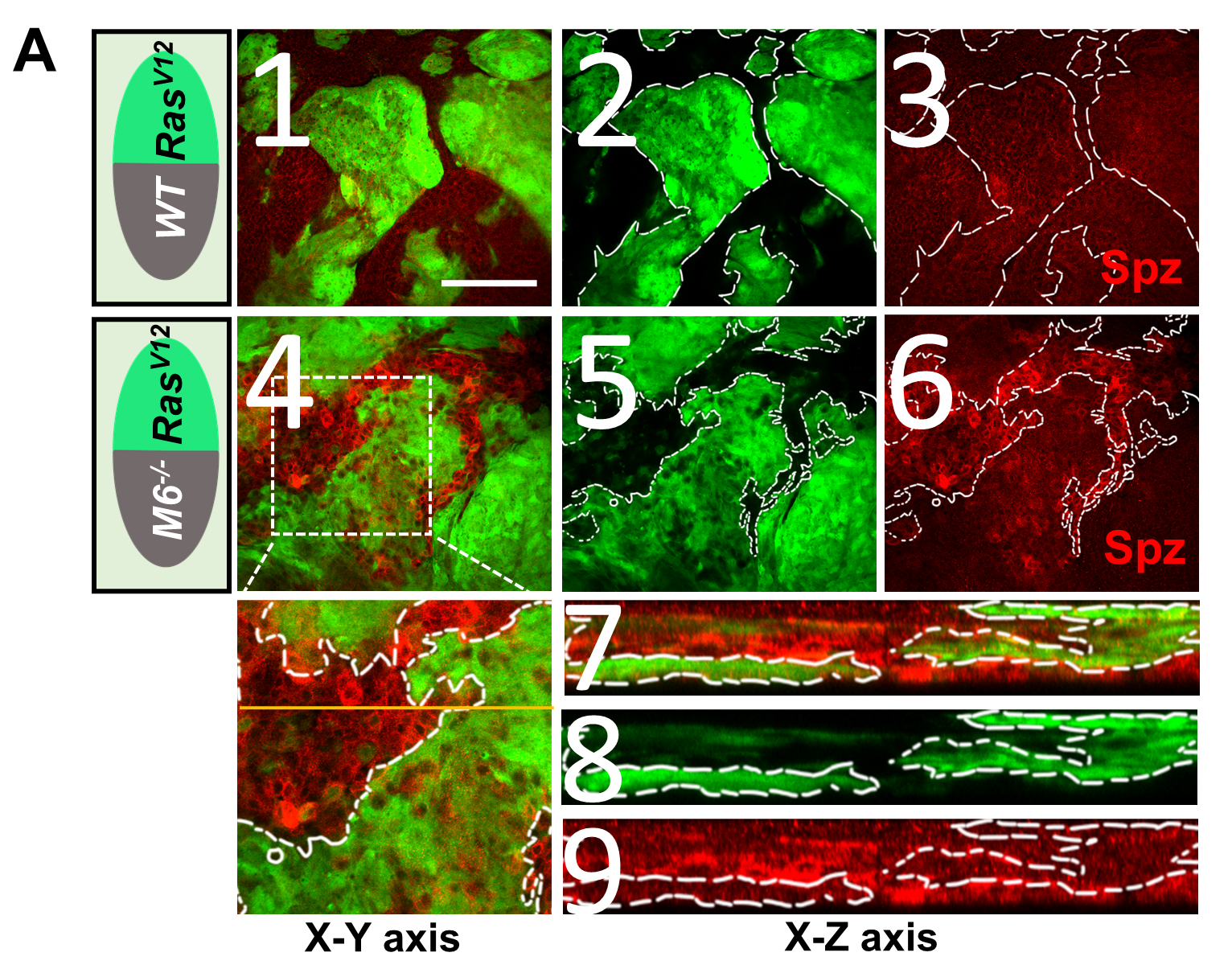

Supplement: Supplementary file 9 — Source data Fig. 5 [file 44318_2025_547_MOESM9_ESM.zip › Figure 5A/0 paper Figure 5A with provided image sequence.tif]

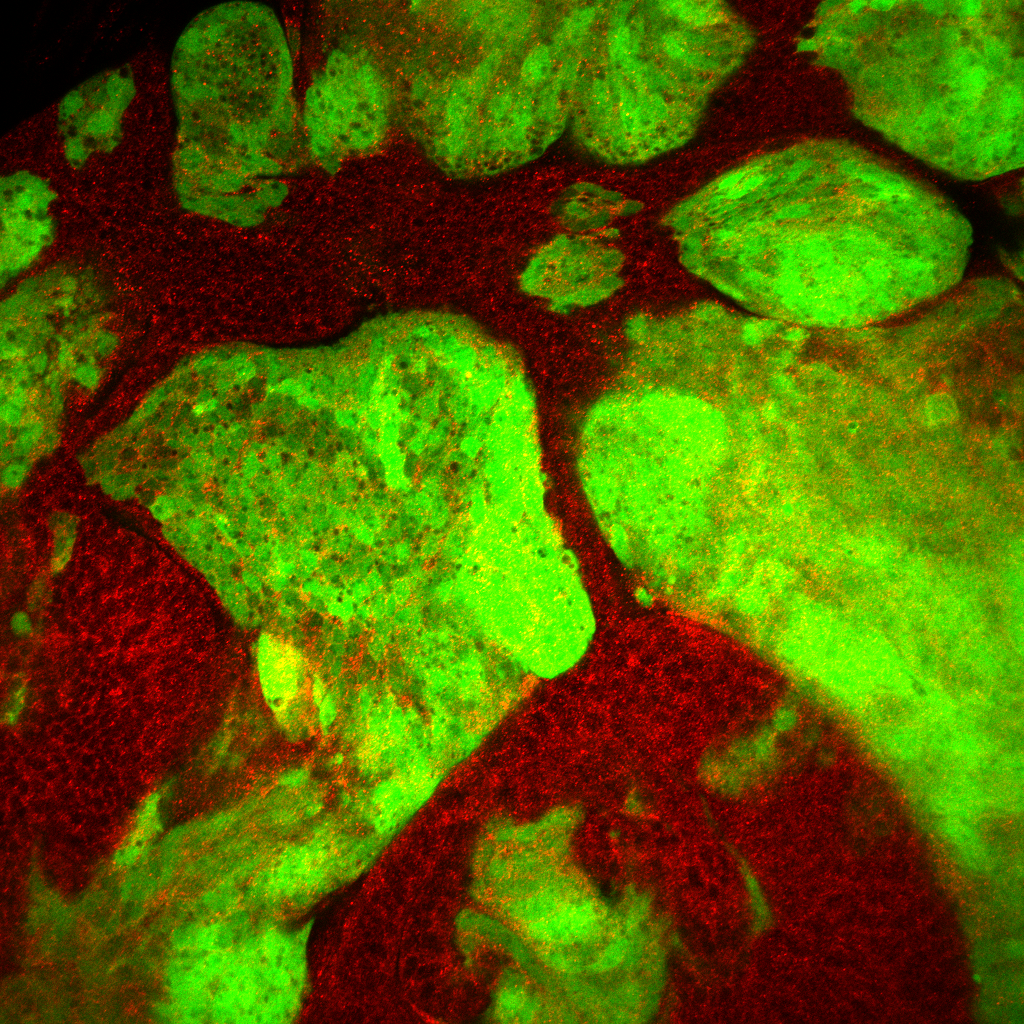

Supplement: Supplementary file 9 — Source data Fig. 5 [file 44318_2025_547_MOESM9_ESM.zip › Figure 5A/1 original image.tif]

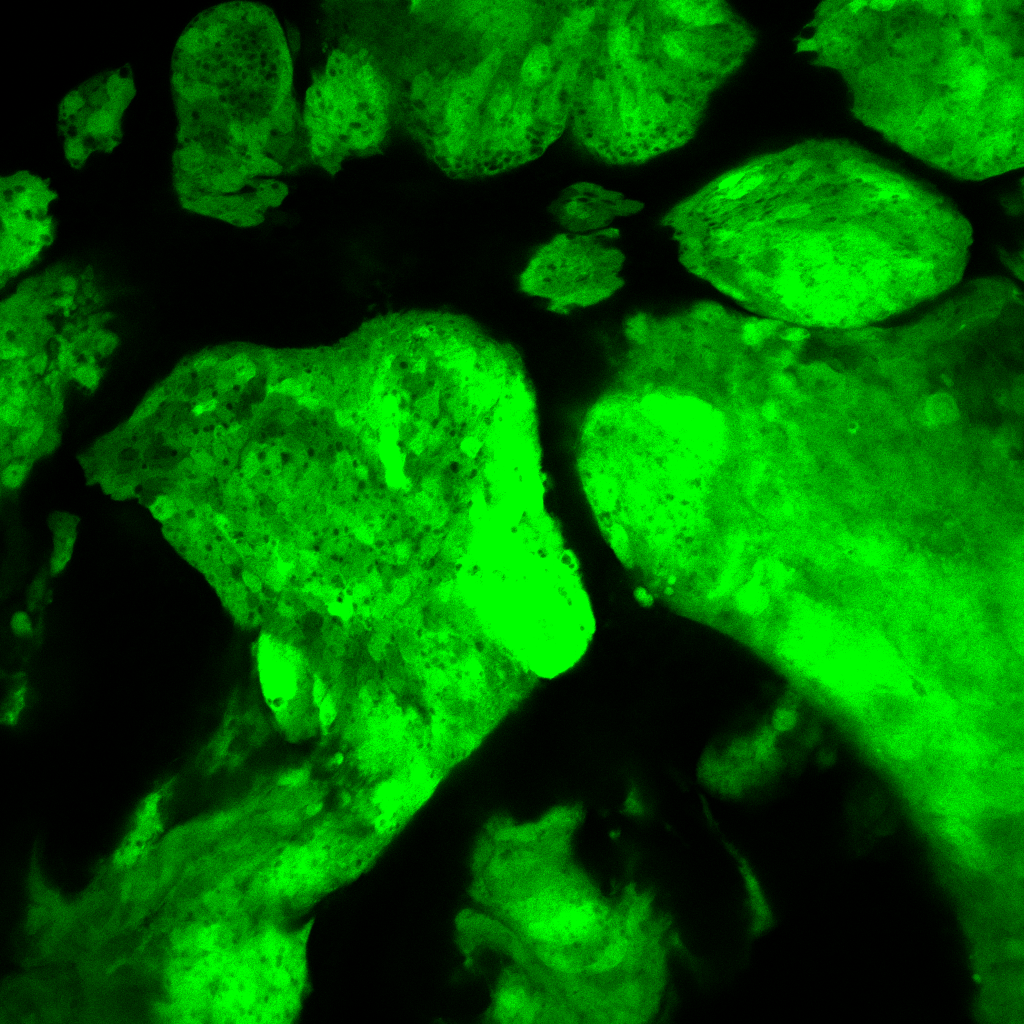

Supplement: Supplementary file 9 — Source data Fig. 5 [file 44318_2025_547_MOESM9_ESM.zip › Figure 5A/2 original image.tif]

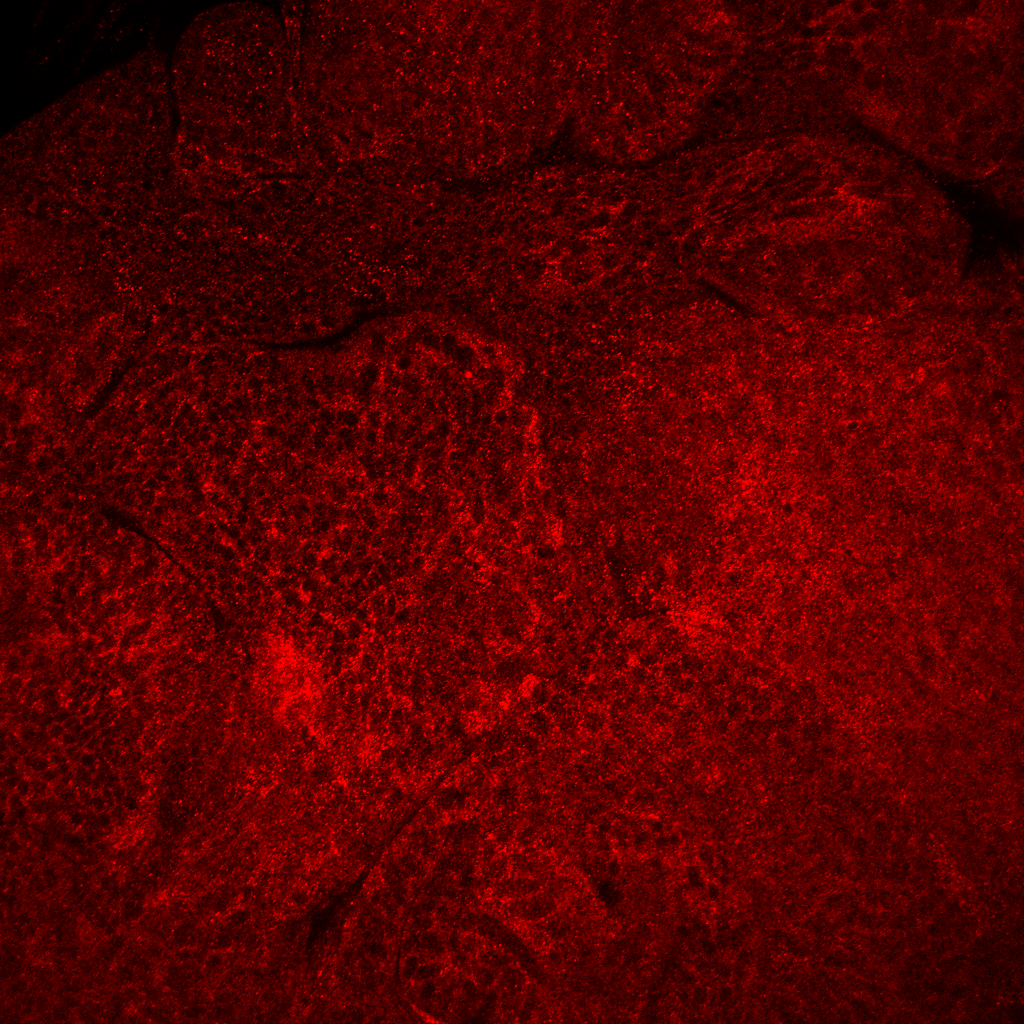

Supplement: Supplementary file 9 — Source data Fig. 5 [file 44318_2025_547_MOESM9_ESM.zip › Figure 5A/3 original image.tif]

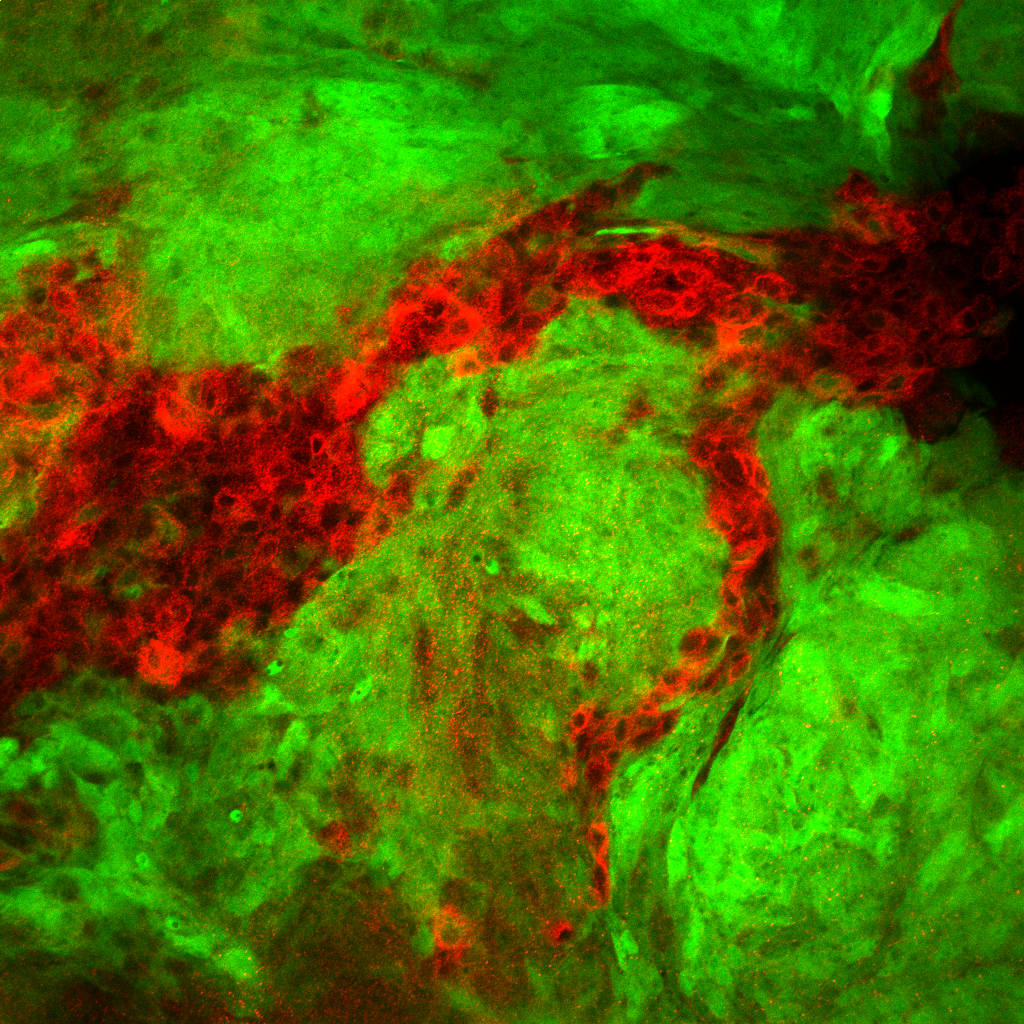

Supplement: Supplementary file 9 — Source data Fig. 5 [file 44318_2025_547_MOESM9_ESM.zip › Figure 5A/4 original image.tif]

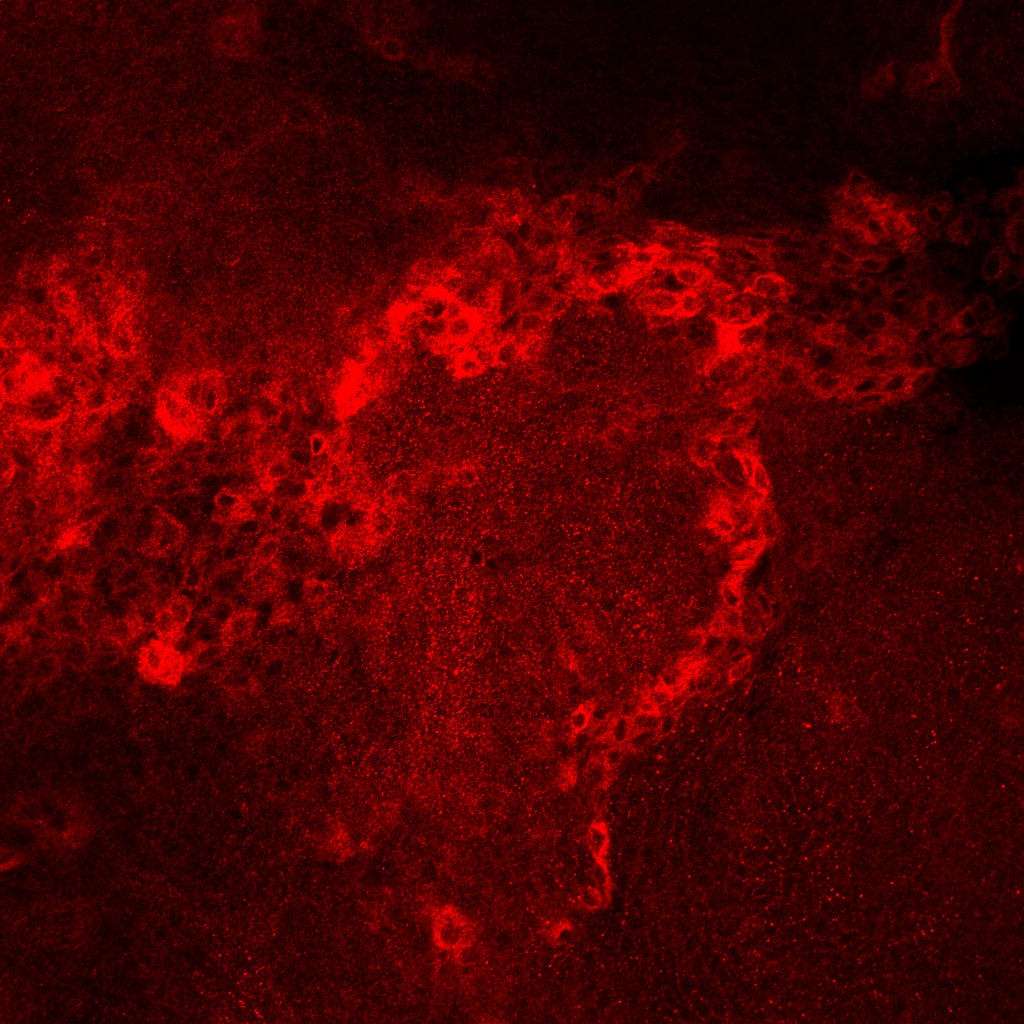

Supplement: Supplementary file 9 — Source data Fig. 5 [file 44318_2025_547_MOESM9_ESM.zip › Figure 5A/6 original image.tif]

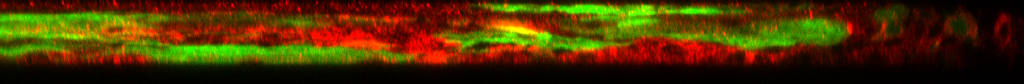

Supplement: Supplementary file 9 — Source data Fig. 5 [file 44318_2025_547_MOESM9_ESM.zip › Figure 5A/7 original image.tif]

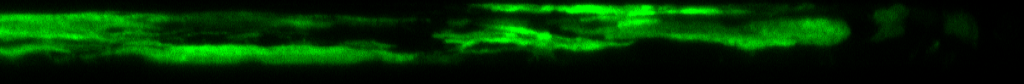

Supplement: Supplementary file 9 — Source data Fig. 5 [file 44318_2025_547_MOESM9_ESM.zip › Figure 5A/8 original image.tif]

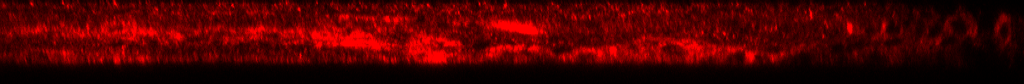

Supplement: Supplementary file 9 — Source data Fig. 5 [file 44318_2025_547_MOESM9_ESM.zip › Figure 5A/9 original image.tif]

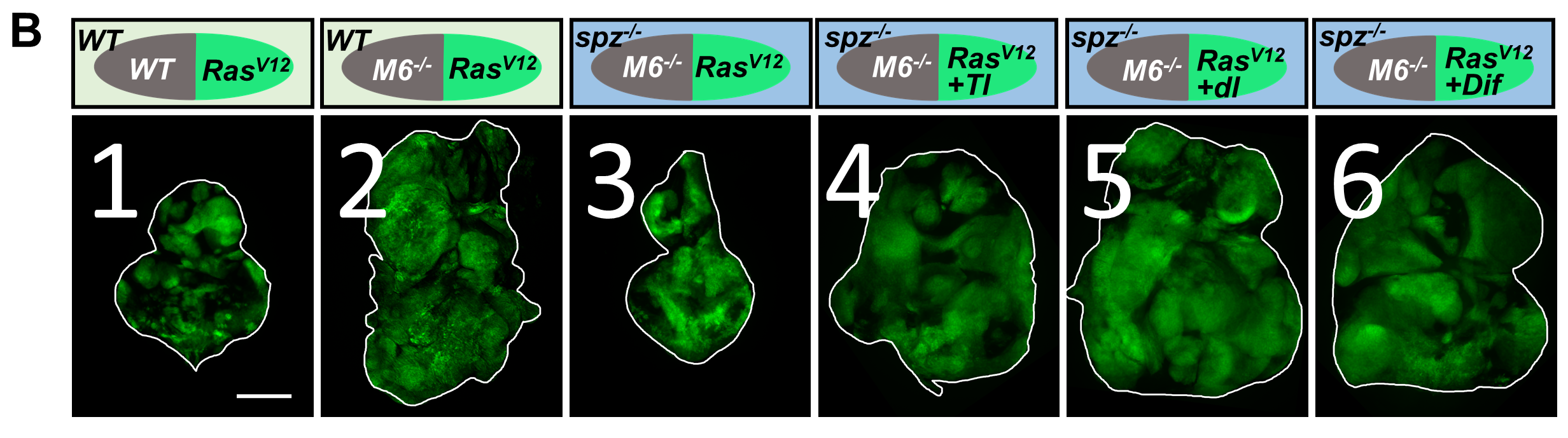

Supplement: Supplementary file 9 — Source data Fig. 5 [file 44318_2025_547_MOESM9_ESM.zip › Figure 5B/0 paper Figure 5B with provided image sequence.tif]

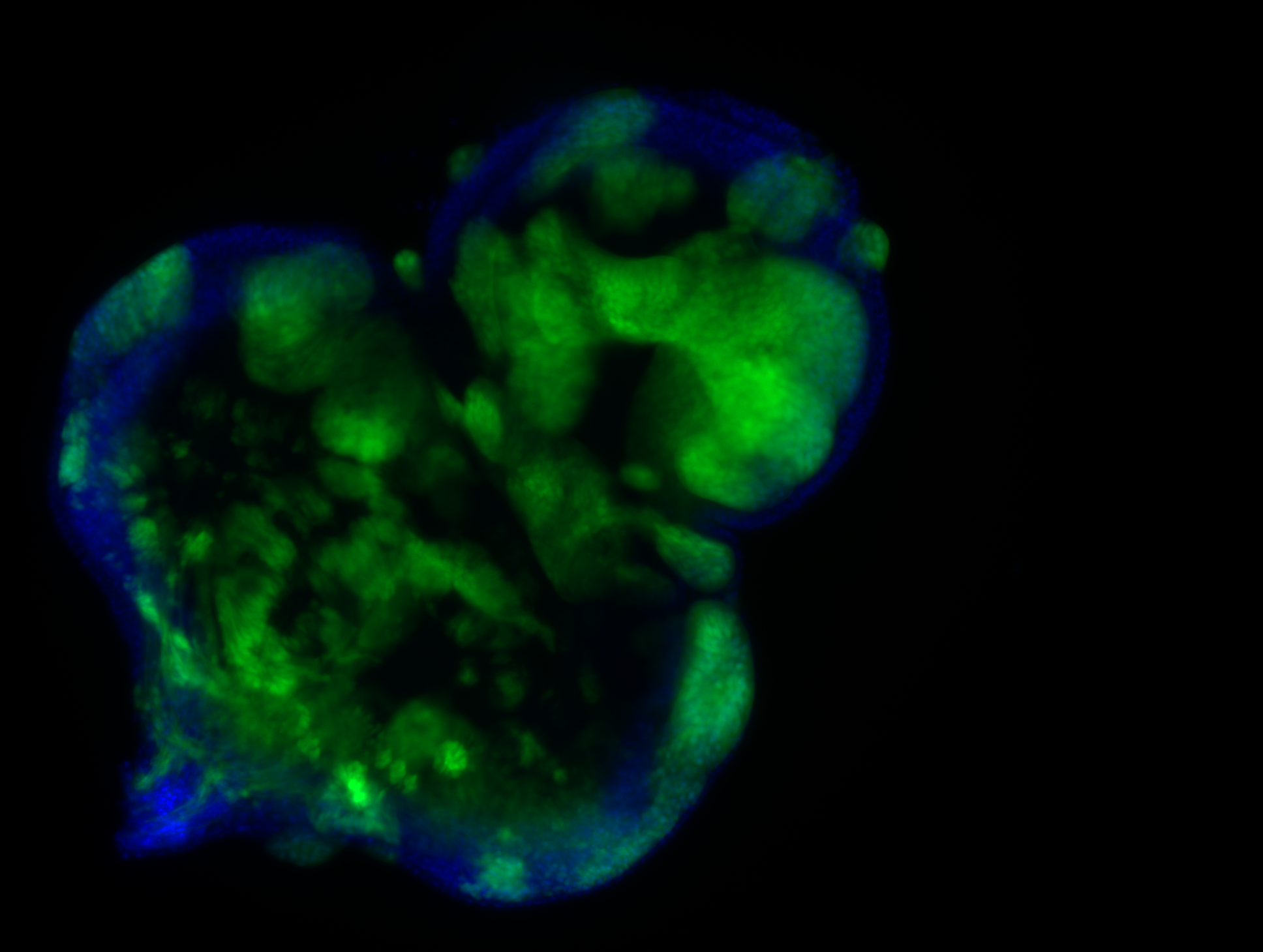

Supplement: Supplementary file 9 — Source data Fig. 5 [file 44318_2025_547_MOESM9_ESM.zip › Figure 5B/1 original image.tif]

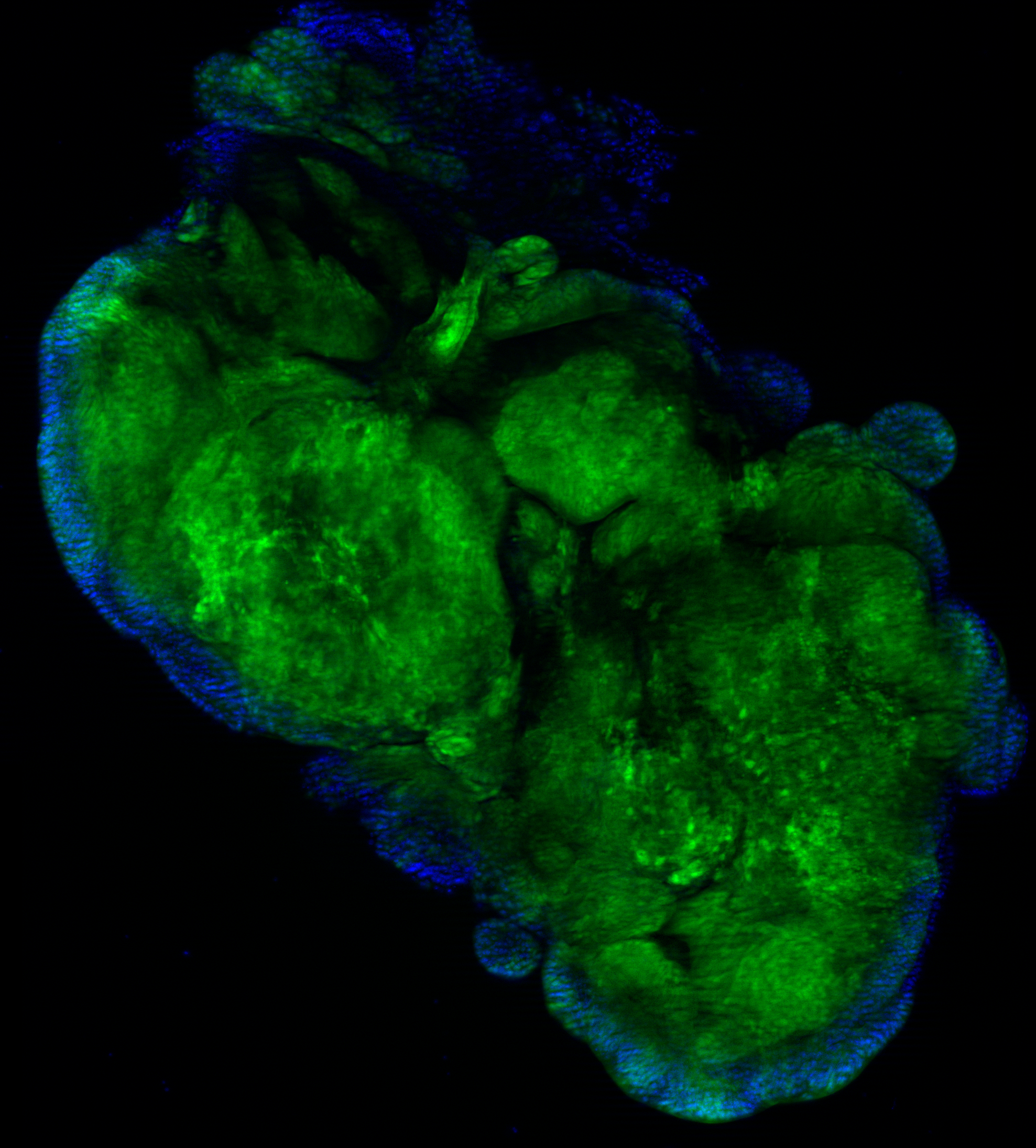

Supplement: Supplementary file 9 — Source data Fig. 5 [file 44318_2025_547_MOESM9_ESM.zip › Figure 5B/2 original image.tif]

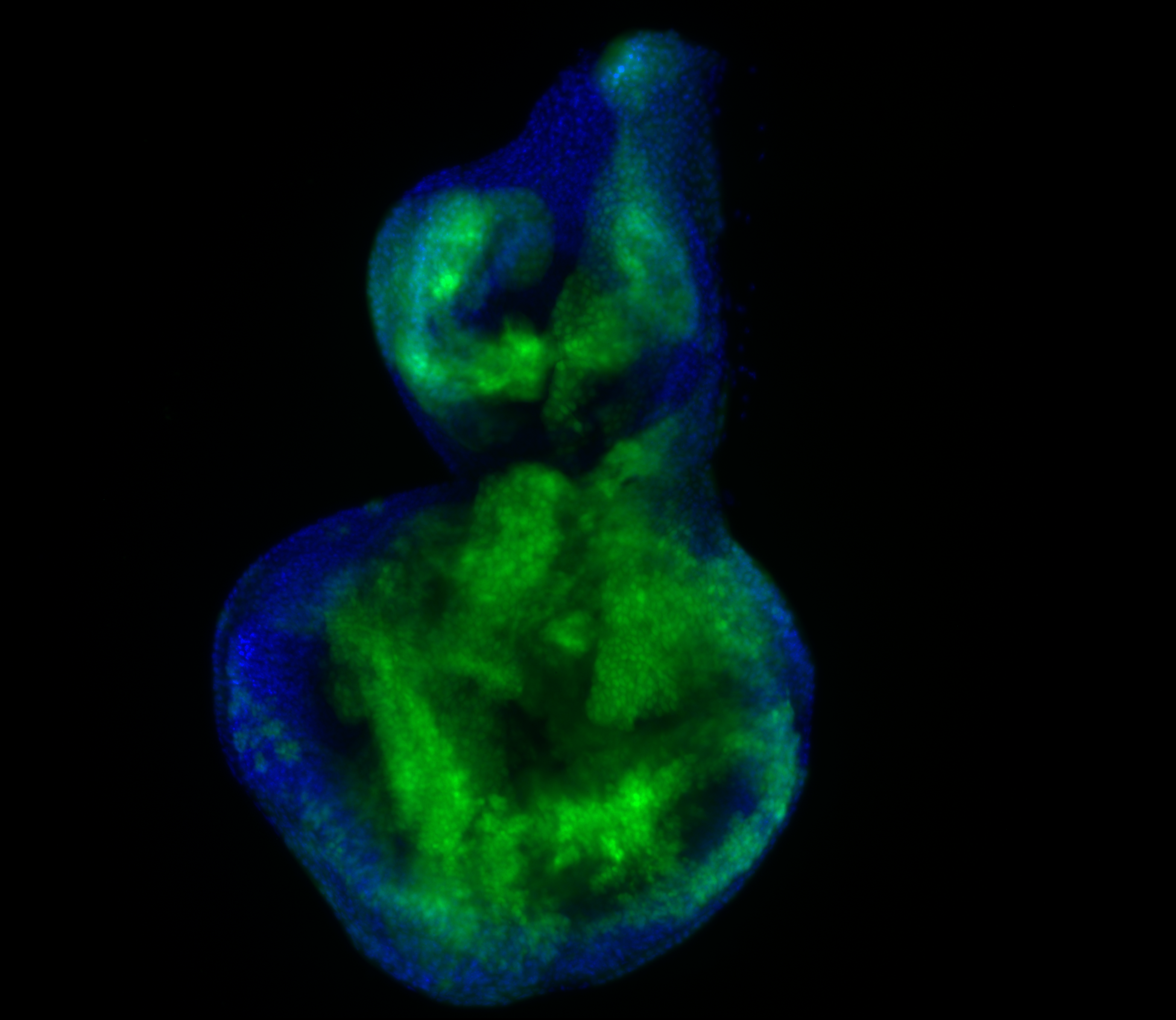

Supplement: Supplementary file 9 — Source data Fig. 5 [file 44318_2025_547_MOESM9_ESM.zip › Figure 5B/3 original image.tif]

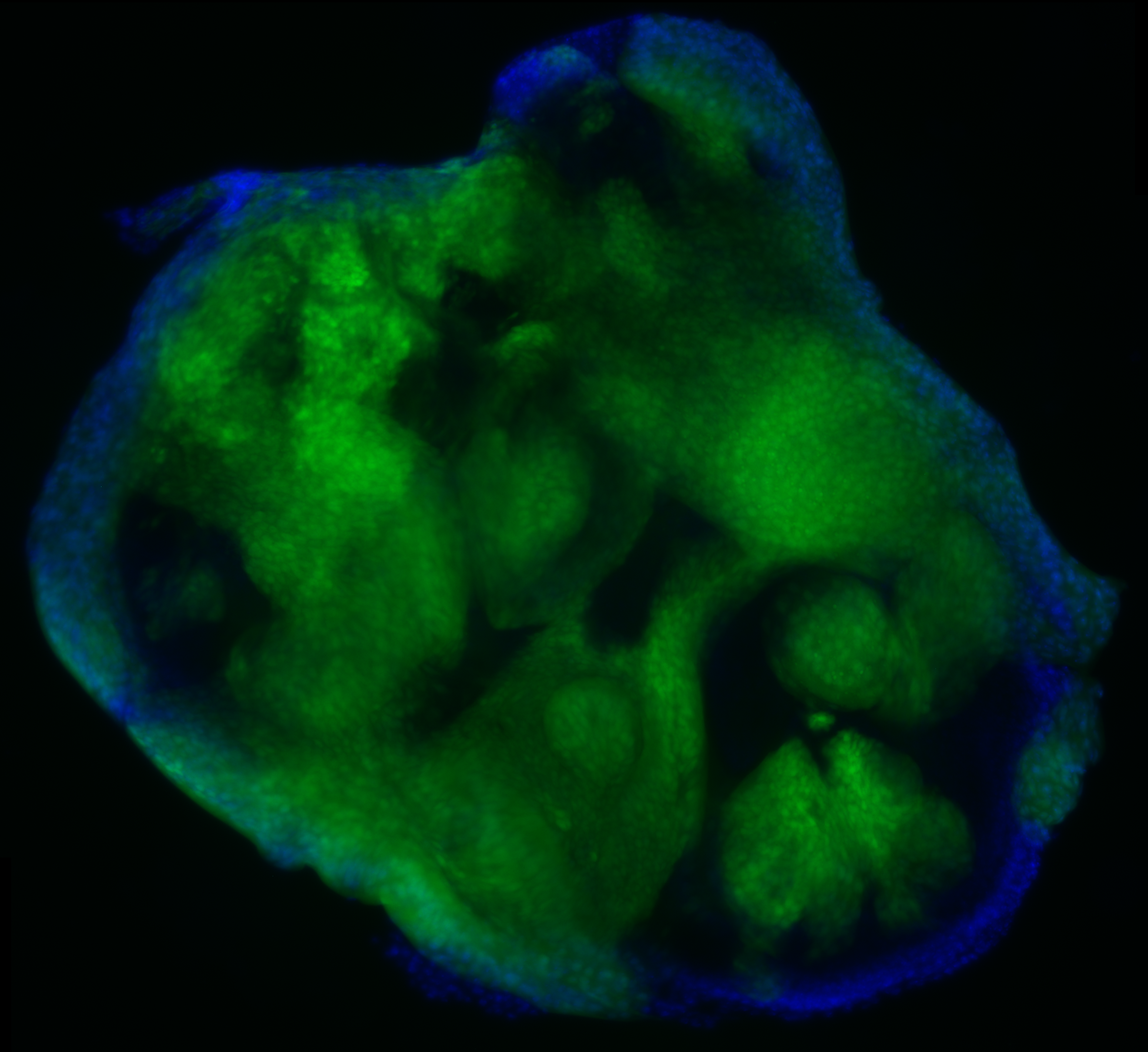

Supplement: Supplementary file 9 — Source data Fig. 5 [file 44318_2025_547_MOESM9_ESM.zip › Figure 5B/4 original image.tif]

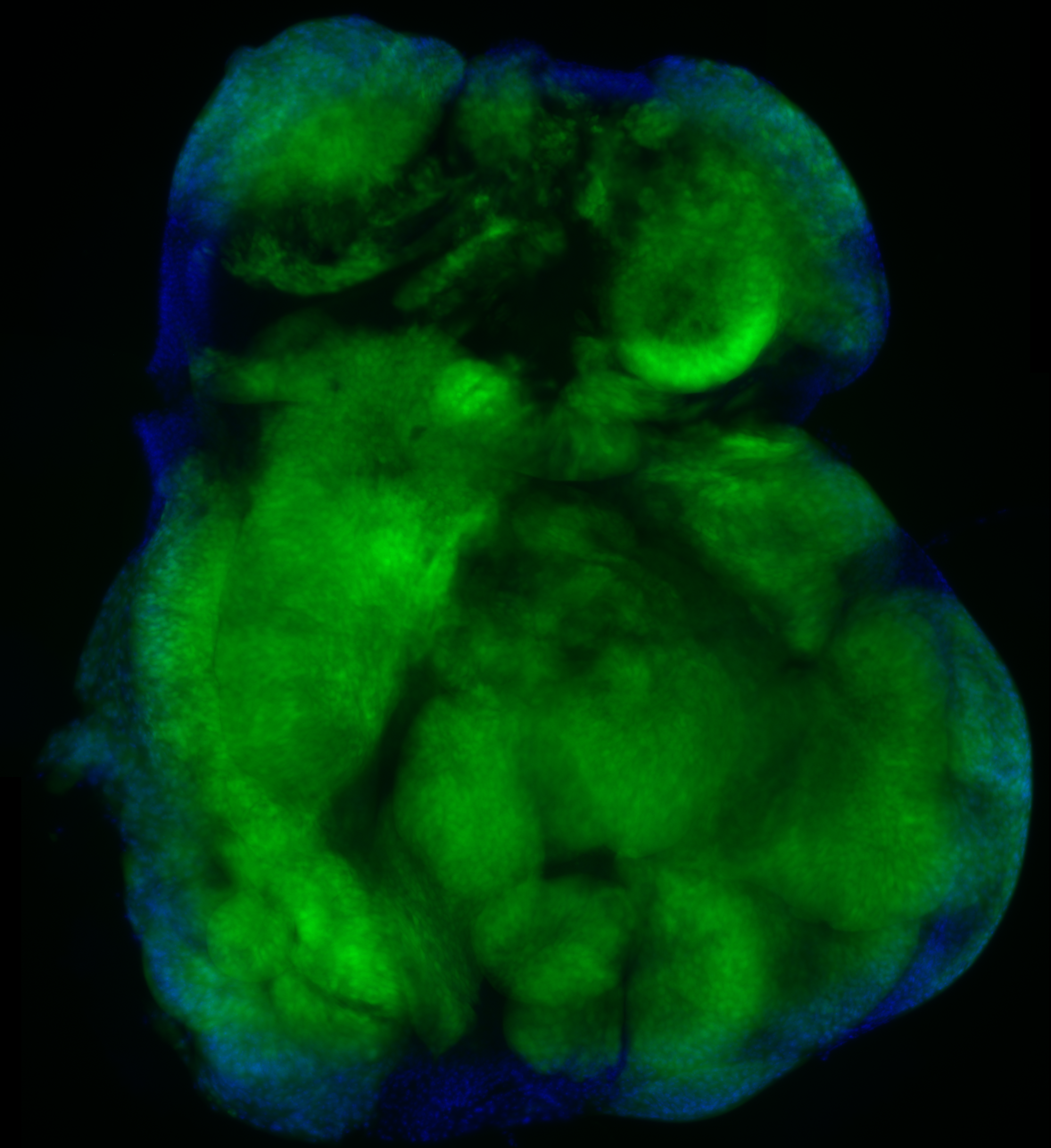

Supplement: Supplementary file 9 — Source data Fig. 5 [file 44318_2025_547_MOESM9_ESM.zip › Figure 5B/5 original image.tif]

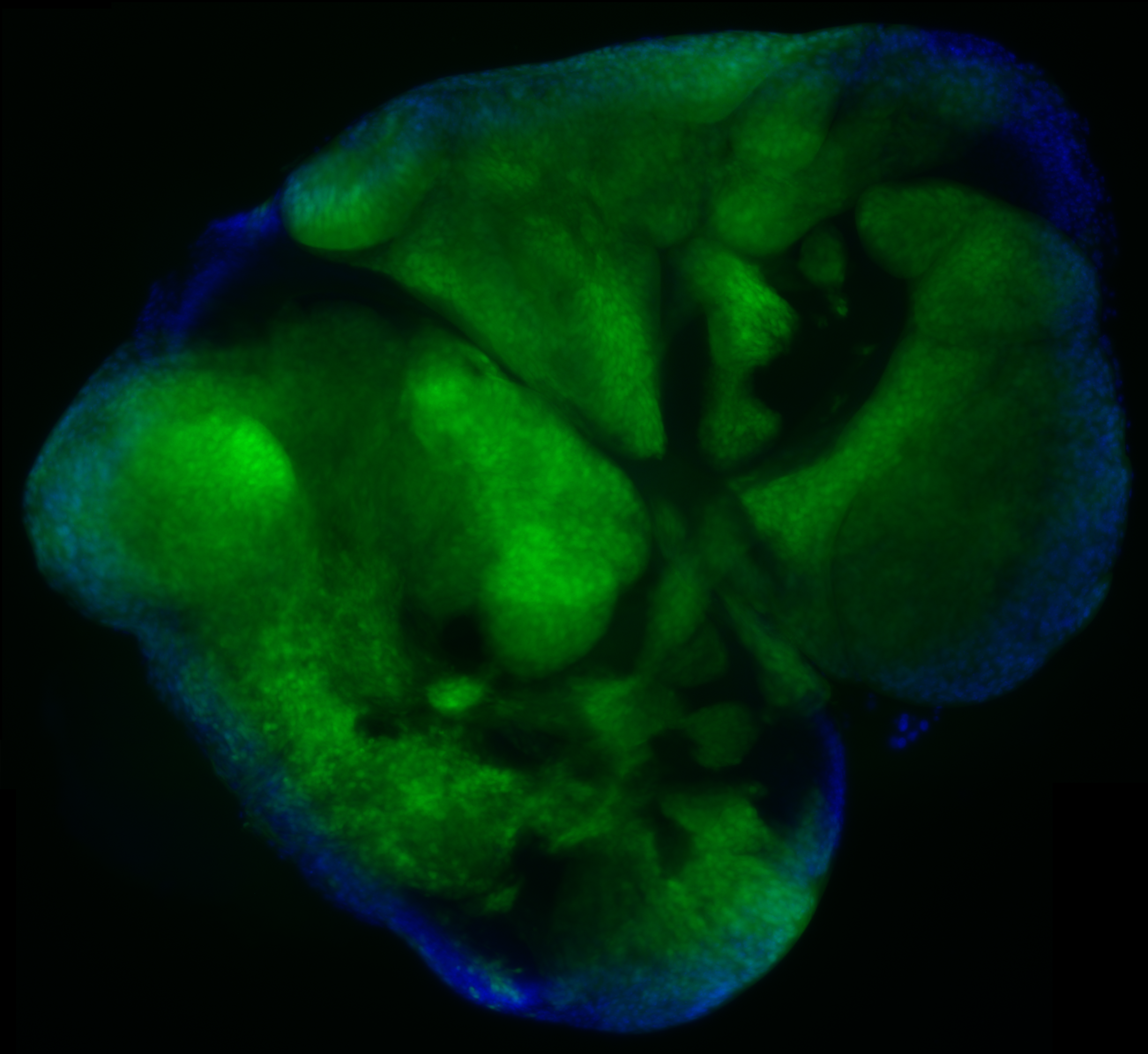

Supplement: Supplementary file 9 — Source data Fig. 5 [file 44318_2025_547_MOESM9_ESM.zip › Figure 5B/6 original image.tif]

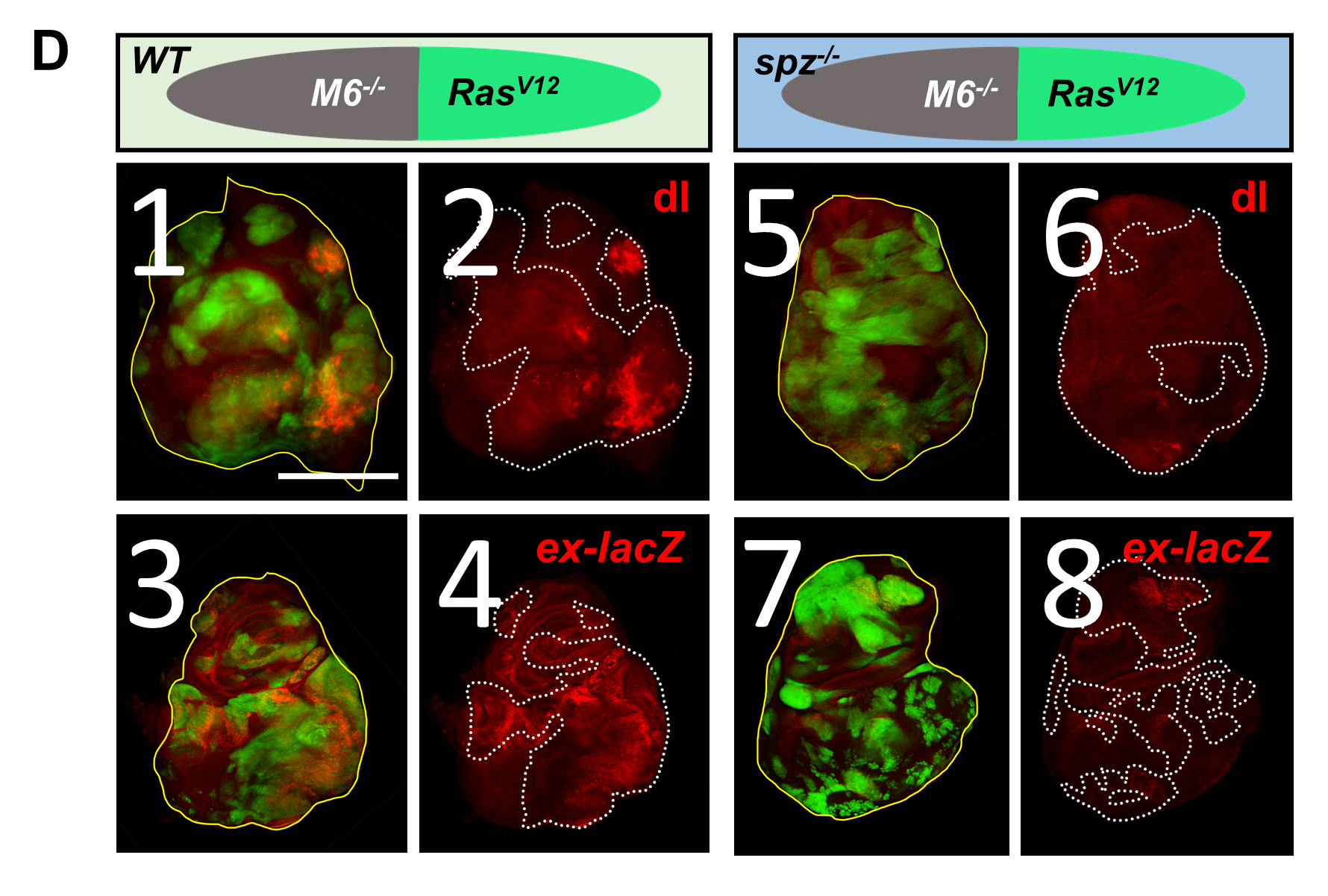

Supplement: Supplementary file 9 — Source data Fig. 5 [file 44318_2025_547_MOESM9_ESM.zip › Figure 5D/0 paper Figure 5D with provided image sequence.tif]

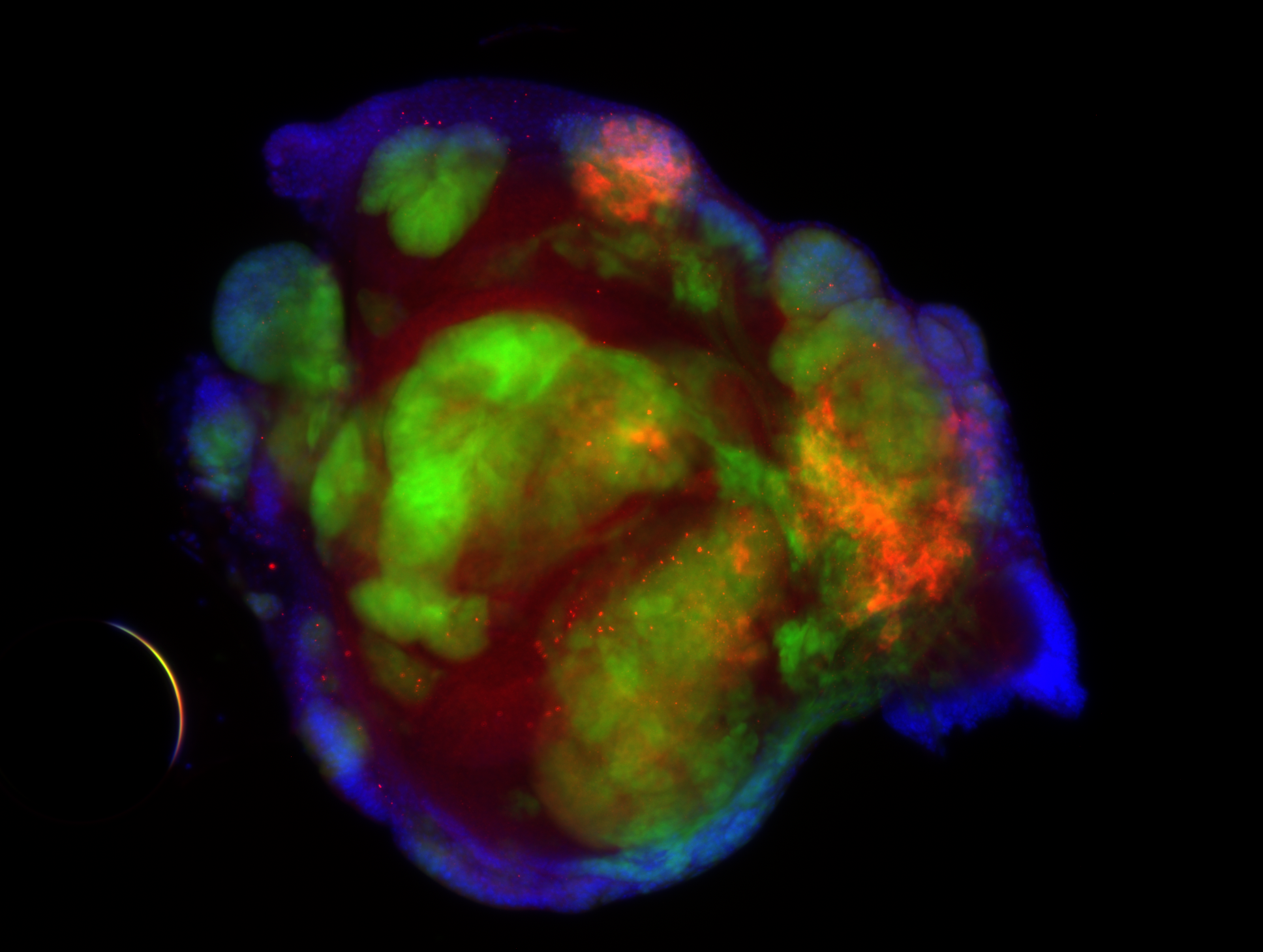

Supplement: Supplementary file 9 — Source data Fig. 5 [file 44318_2025_547_MOESM9_ESM.zip › Figure 5D/1 original image.tif]

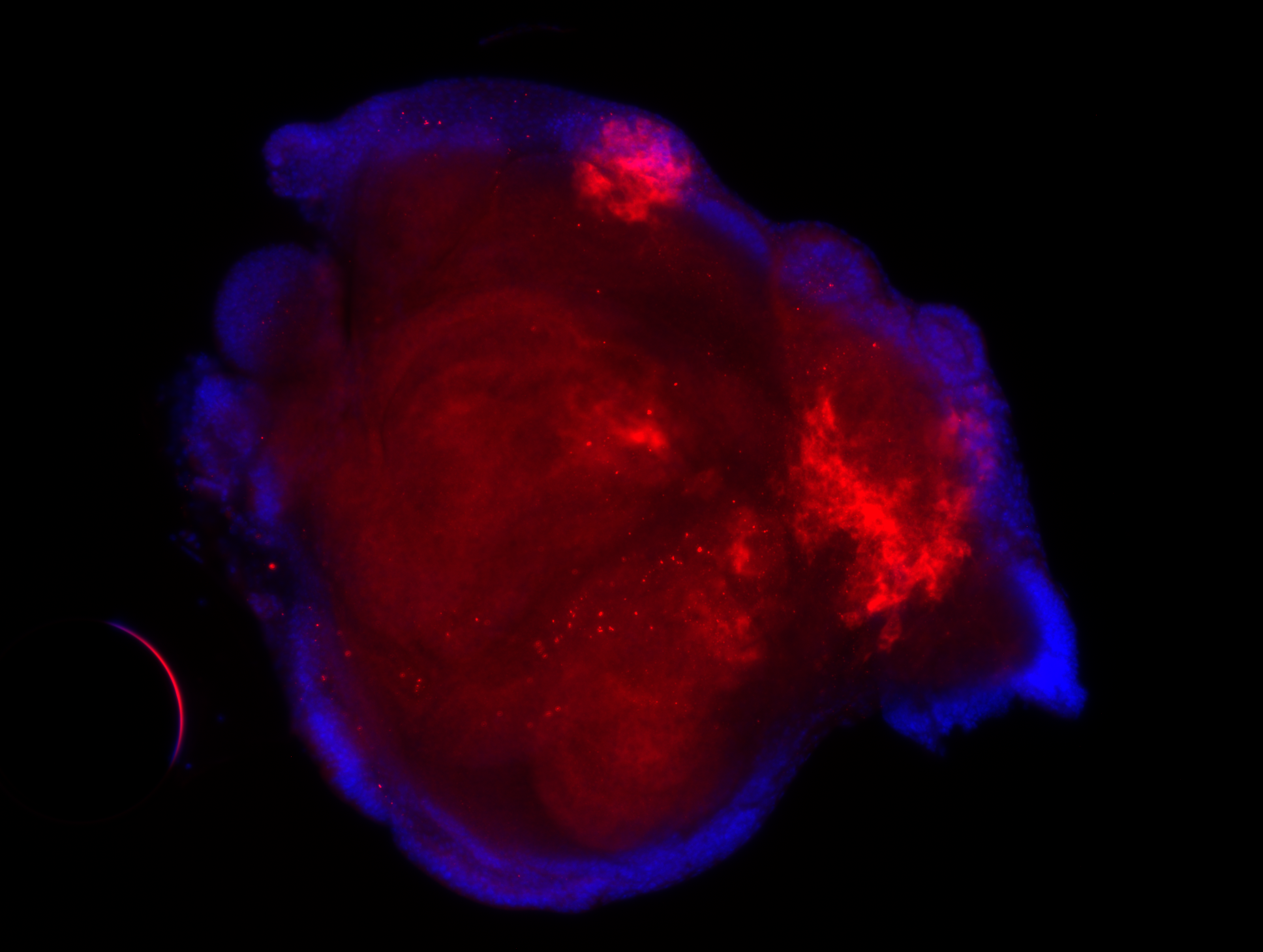

Supplement: Supplementary file 9 — Source data Fig. 5 [file 44318_2025_547_MOESM9_ESM.zip › Figure 5D/2 original image.tif]

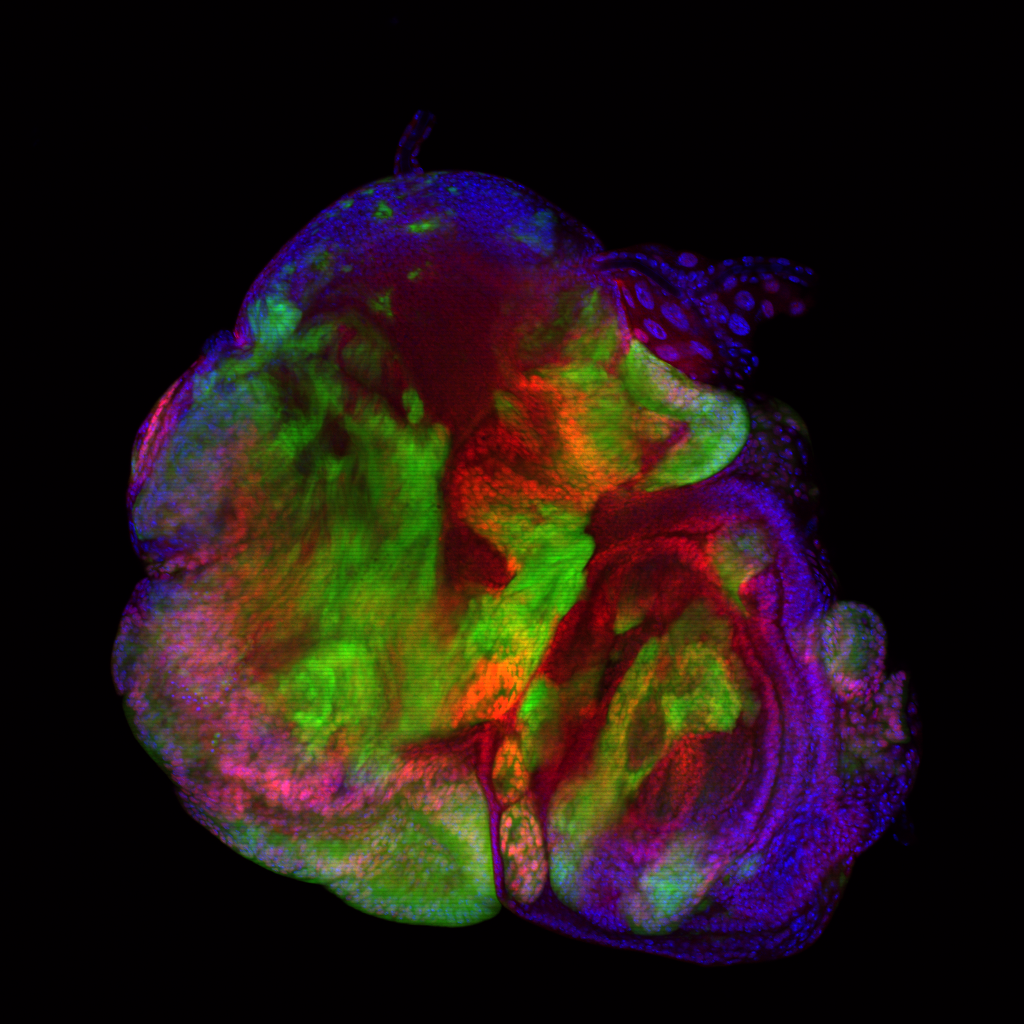

Supplement: Supplementary file 9 — Source data Fig. 5 [file 44318_2025_547_MOESM9_ESM.zip › Figure 5D/3 original image.tif]

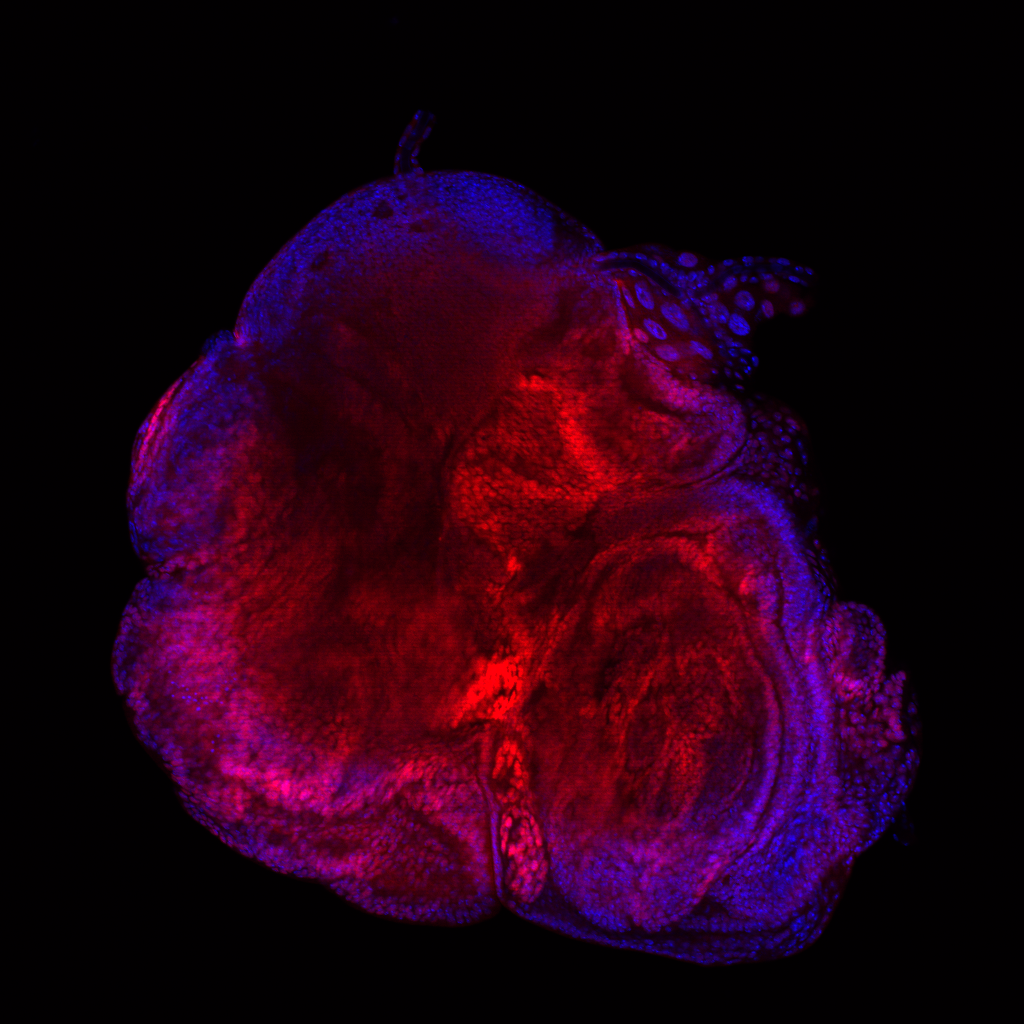

Supplement: Supplementary file 9 — Source data Fig. 5 [file 44318_2025_547_MOESM9_ESM.zip › Figure 5D/4 original image.tif]

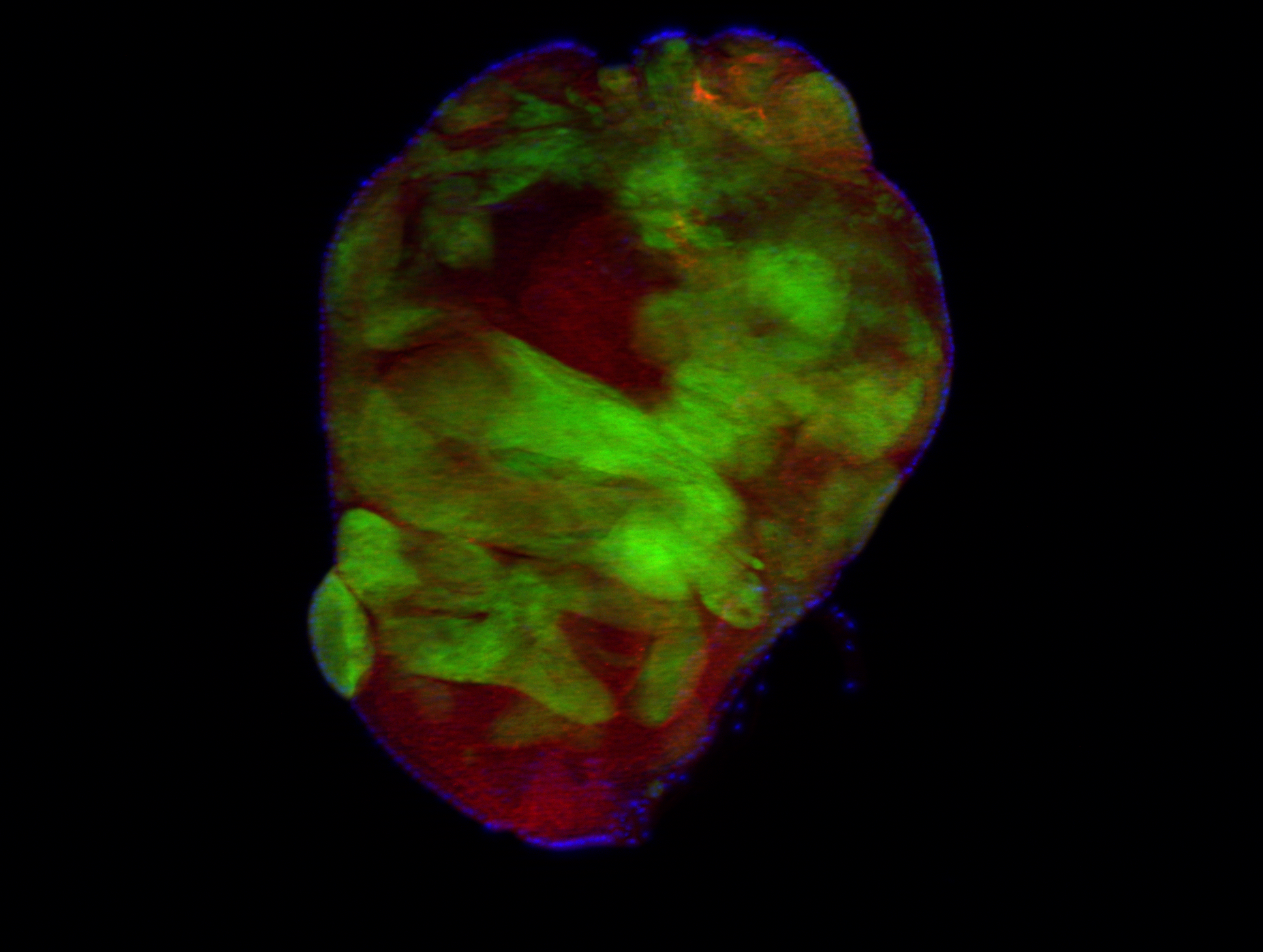

Supplement: Supplementary file 9 — Source data Fig. 5 [file 44318_2025_547_MOESM9_ESM.zip › Figure 5D/5 original image.tif]

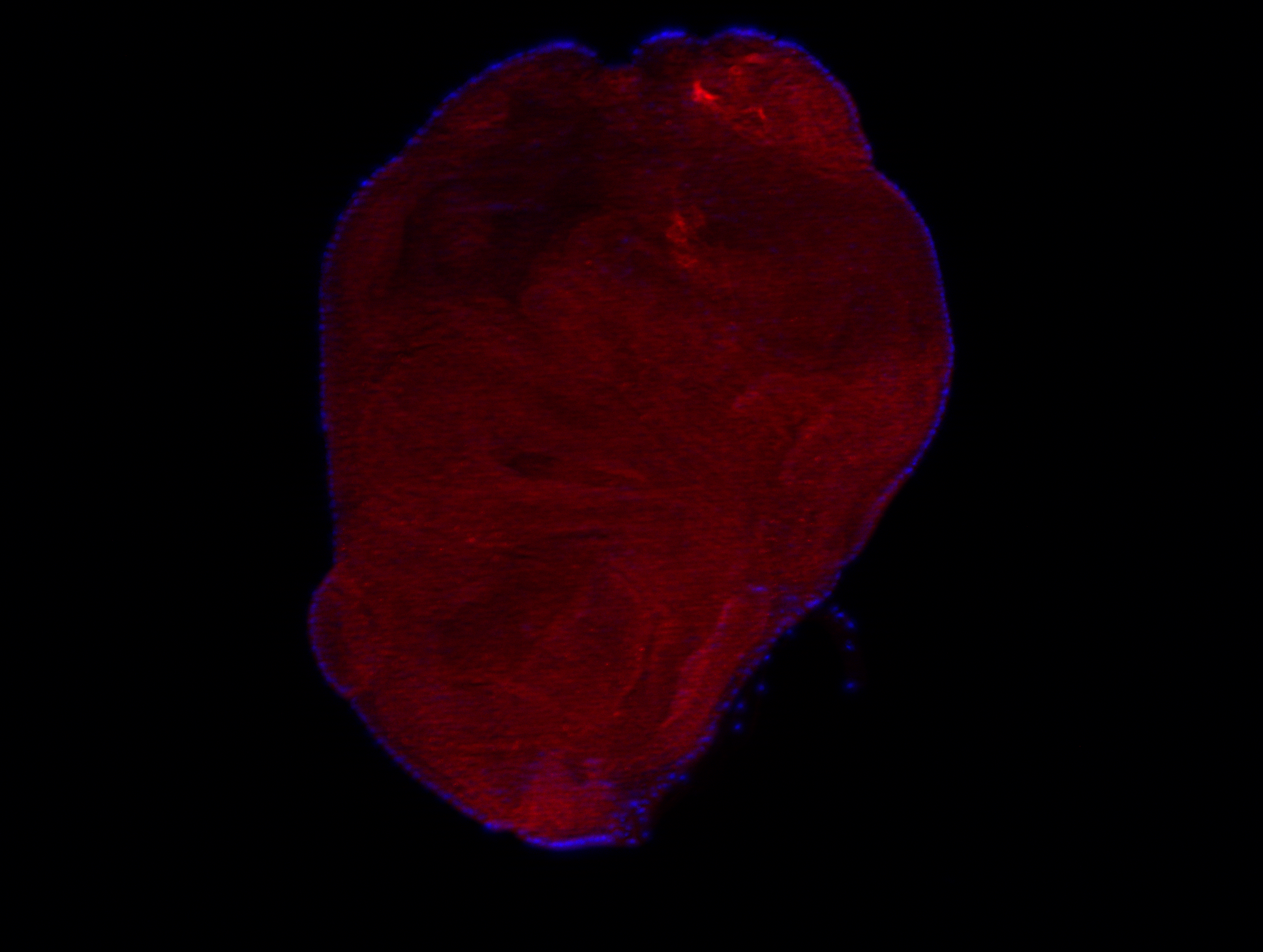

Supplement: Supplementary file 9 — Source data Fig. 5 [file 44318_2025_547_MOESM9_ESM.zip › Figure 5D/6 original image.tif]

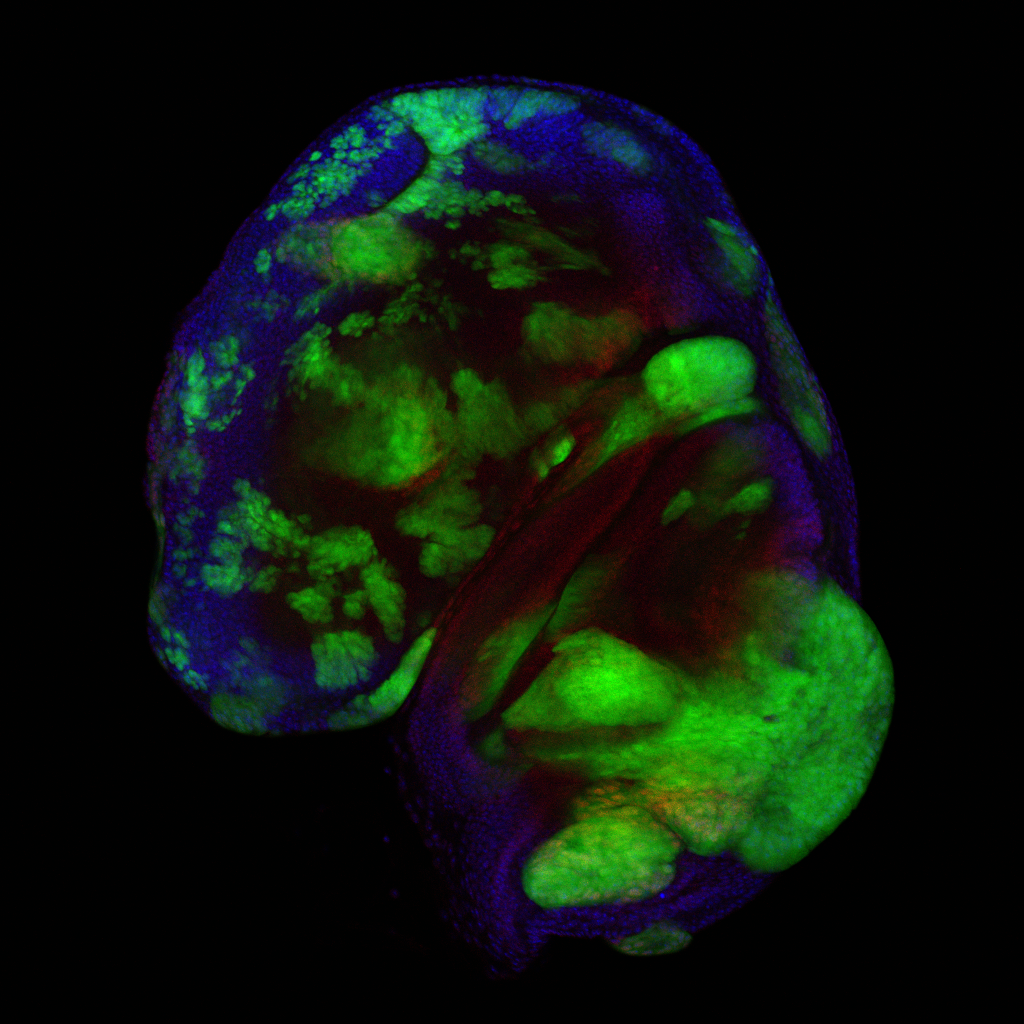

Supplement: Supplementary file 9 — Source data Fig. 5 [file 44318_2025_547_MOESM9_ESM.zip › Figure 5D/7 original image.tif]

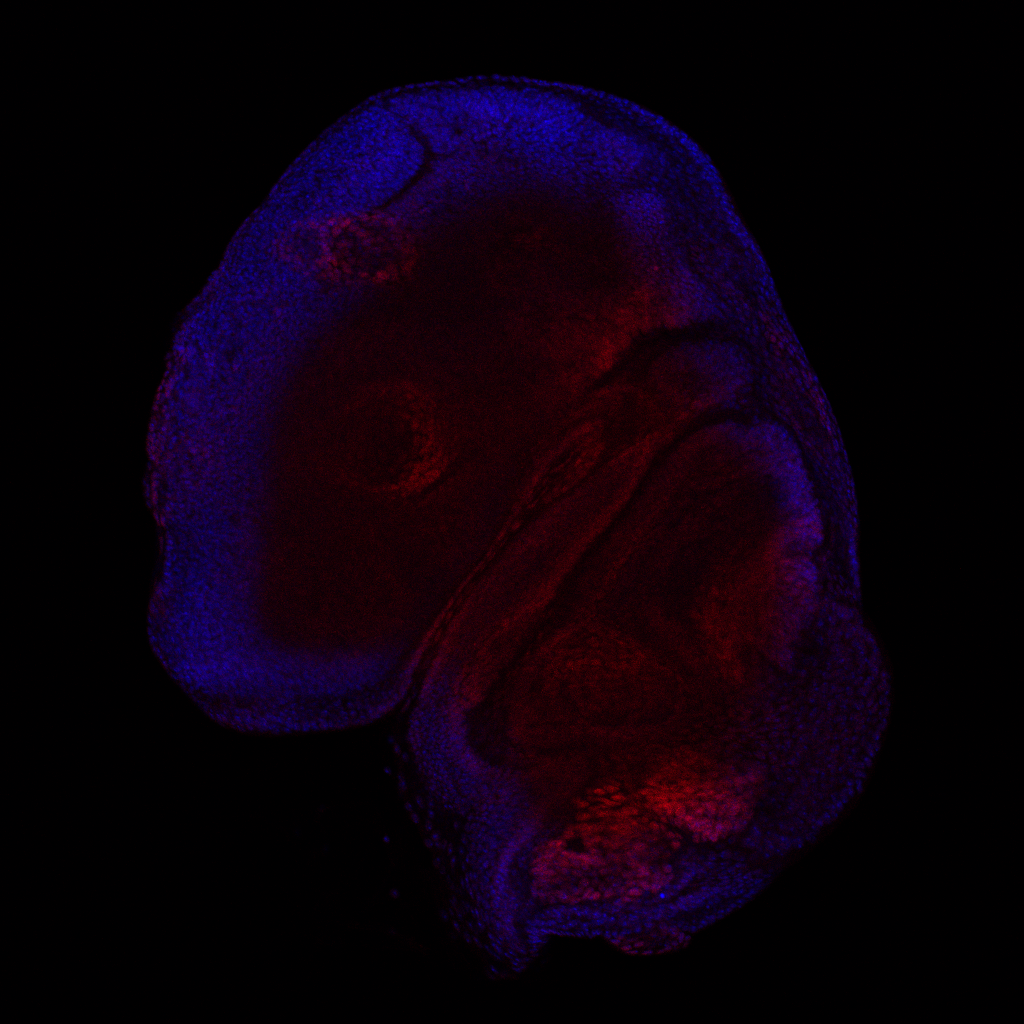

Supplement: Supplementary file 9 — Source data Fig. 5 [file 44318_2025_547_MOESM9_ESM.zip › Figure 5D/8 original image.tif]

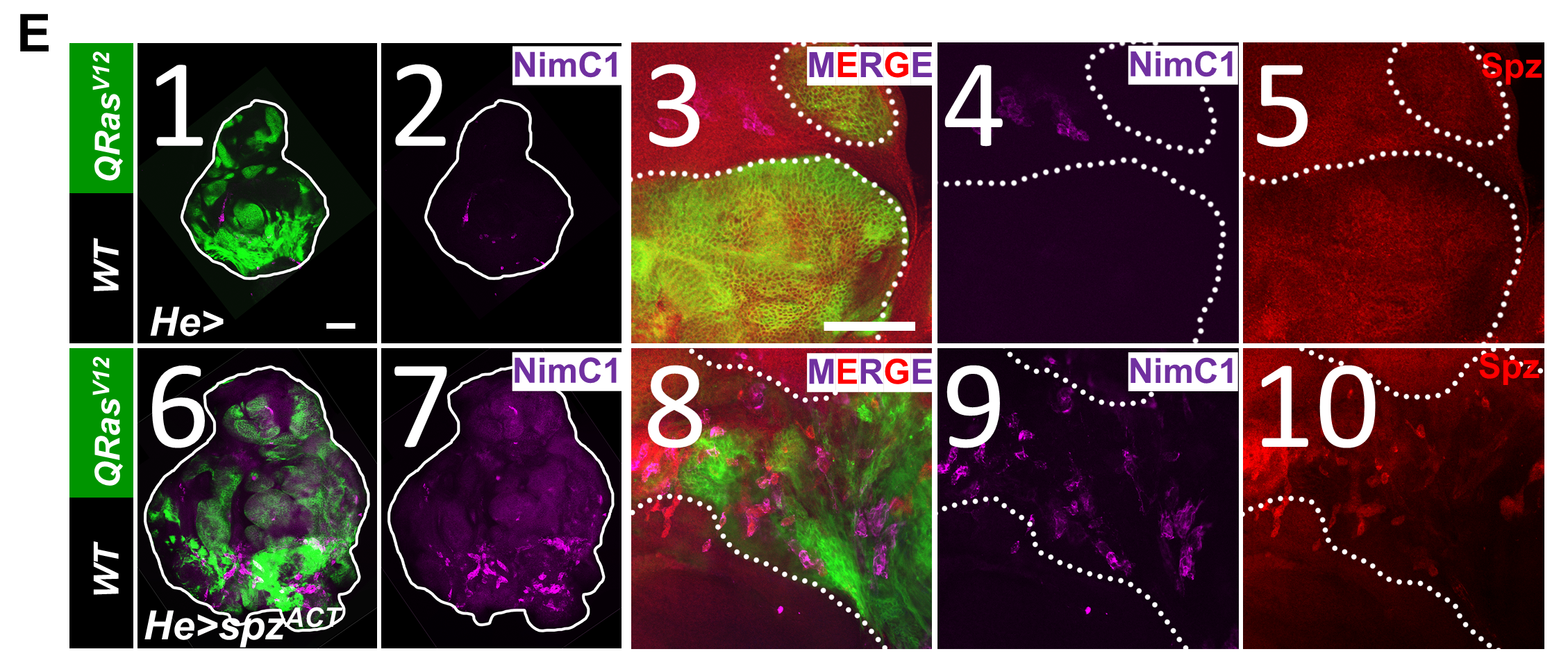

Supplement: Supplementary file 10 — Source data Fig. 6 [file 44318_2025_547_MOESM10_ESM.zip › Figure 6E/0 paper Figure 6E with provided image sequence.tif]

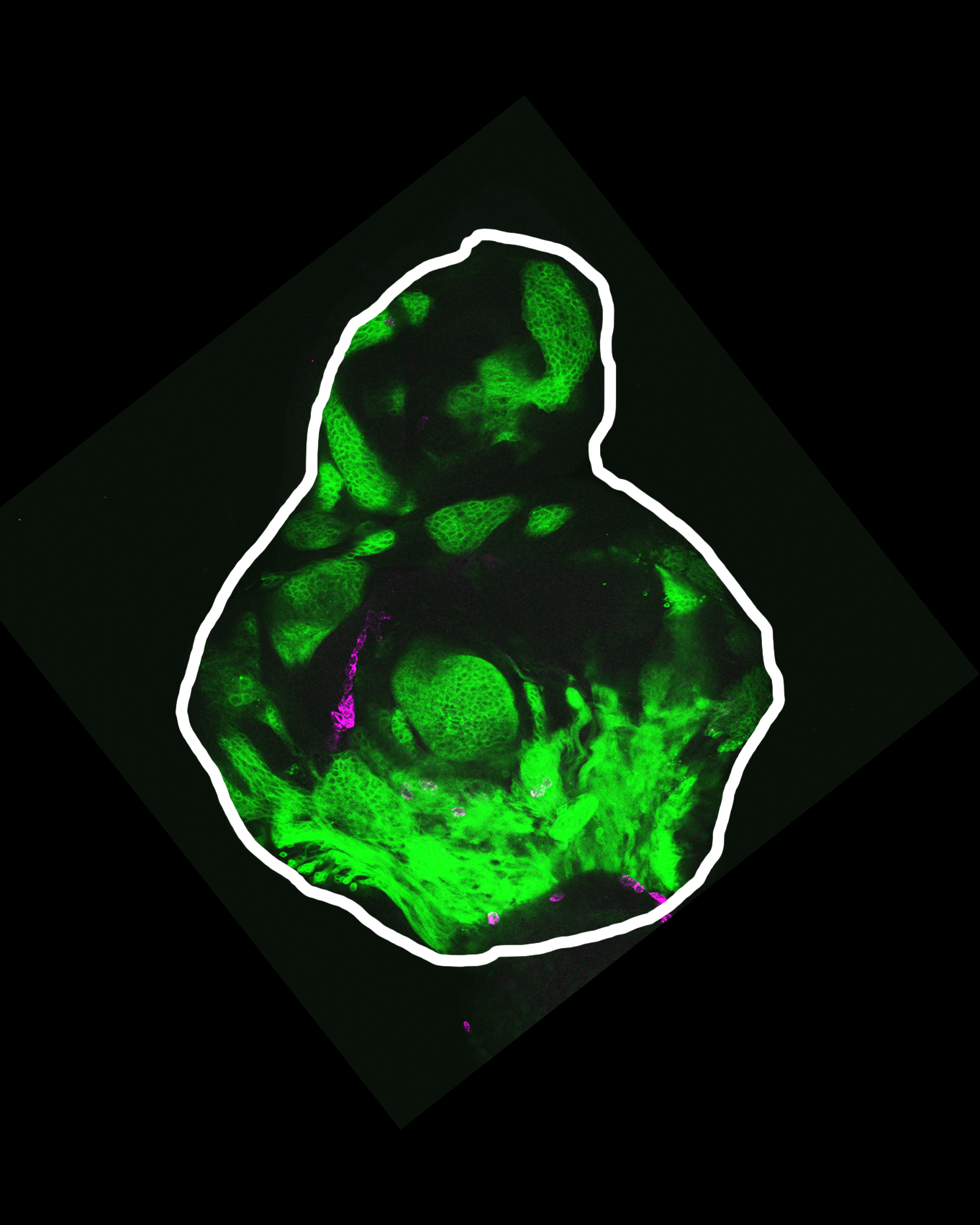

Supplement: Supplementary file 10 — Source data Fig. 6 [file 44318_2025_547_MOESM10_ESM.zip › Figure 6E/1-1 rotated and cut image.tif]

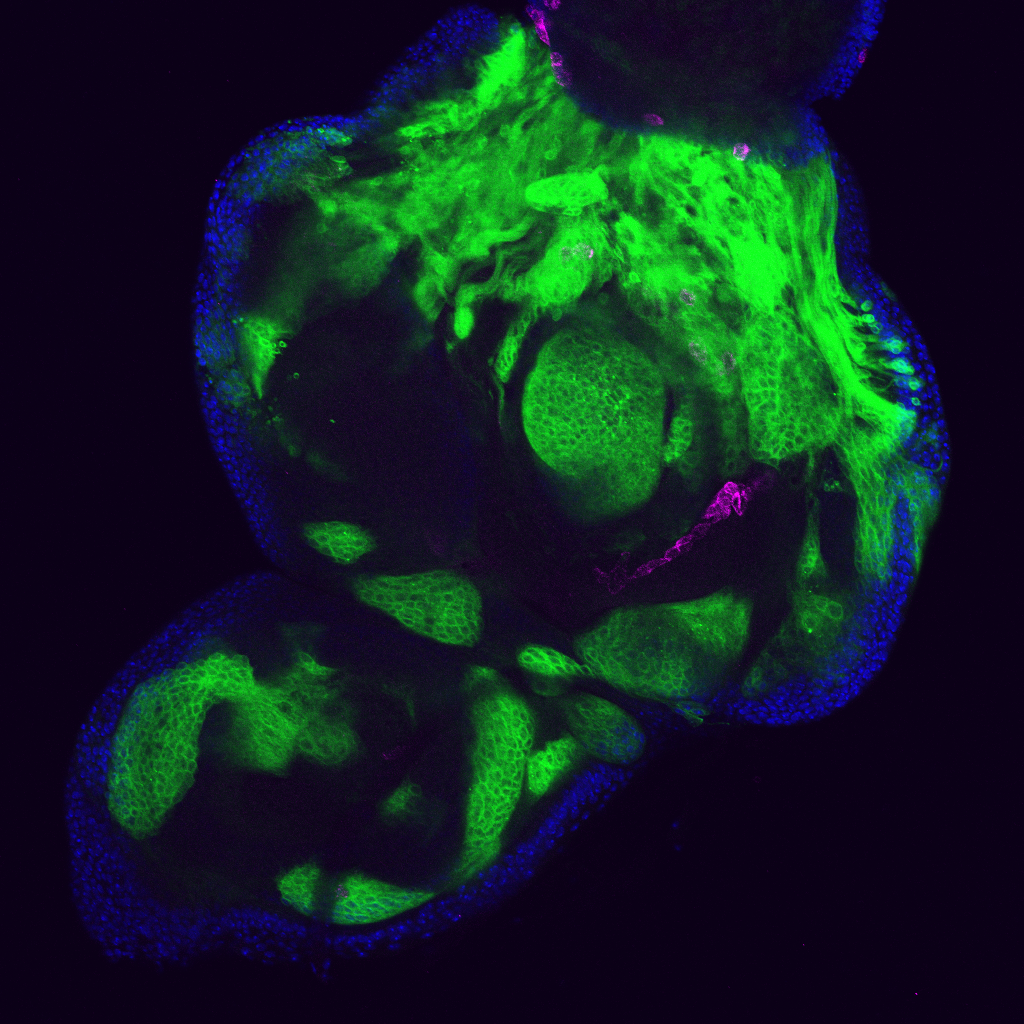

Supplement: Supplementary file 10 — Source data Fig. 6 [file 44318_2025_547_MOESM10_ESM.zip › Figure 6E/1-2 original image.tif]

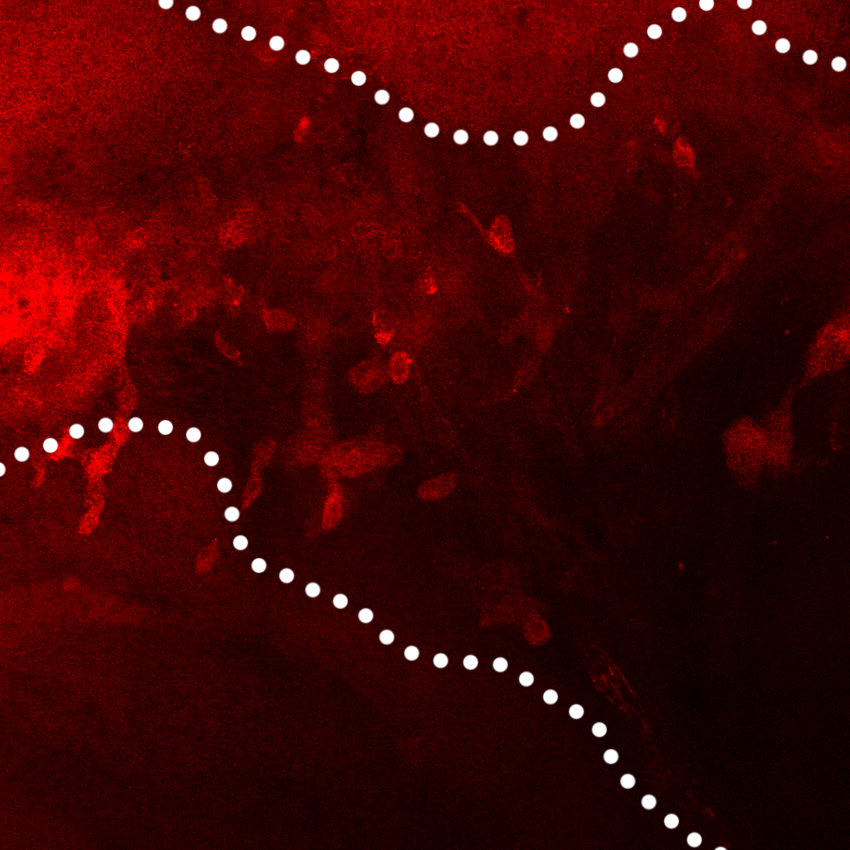

Supplement: Supplementary file 10 — Source data Fig. 6 [file 44318_2025_547_MOESM10_ESM.zip › Figure 6E/10-1 rotated and cut image.tif]

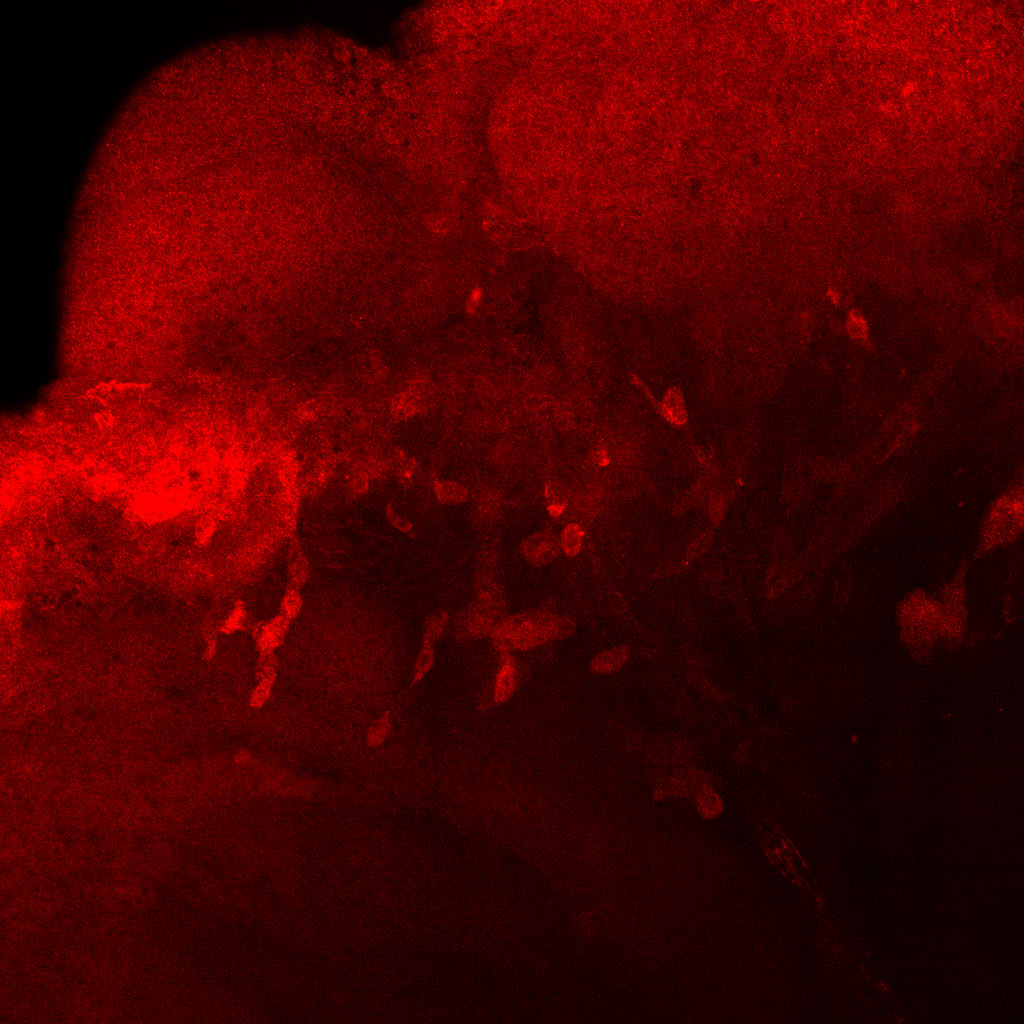

Supplement: Supplementary file 10 — Source data Fig. 6 [file 44318_2025_547_MOESM10_ESM.zip › Figure 6E/10-2 original image.tif]

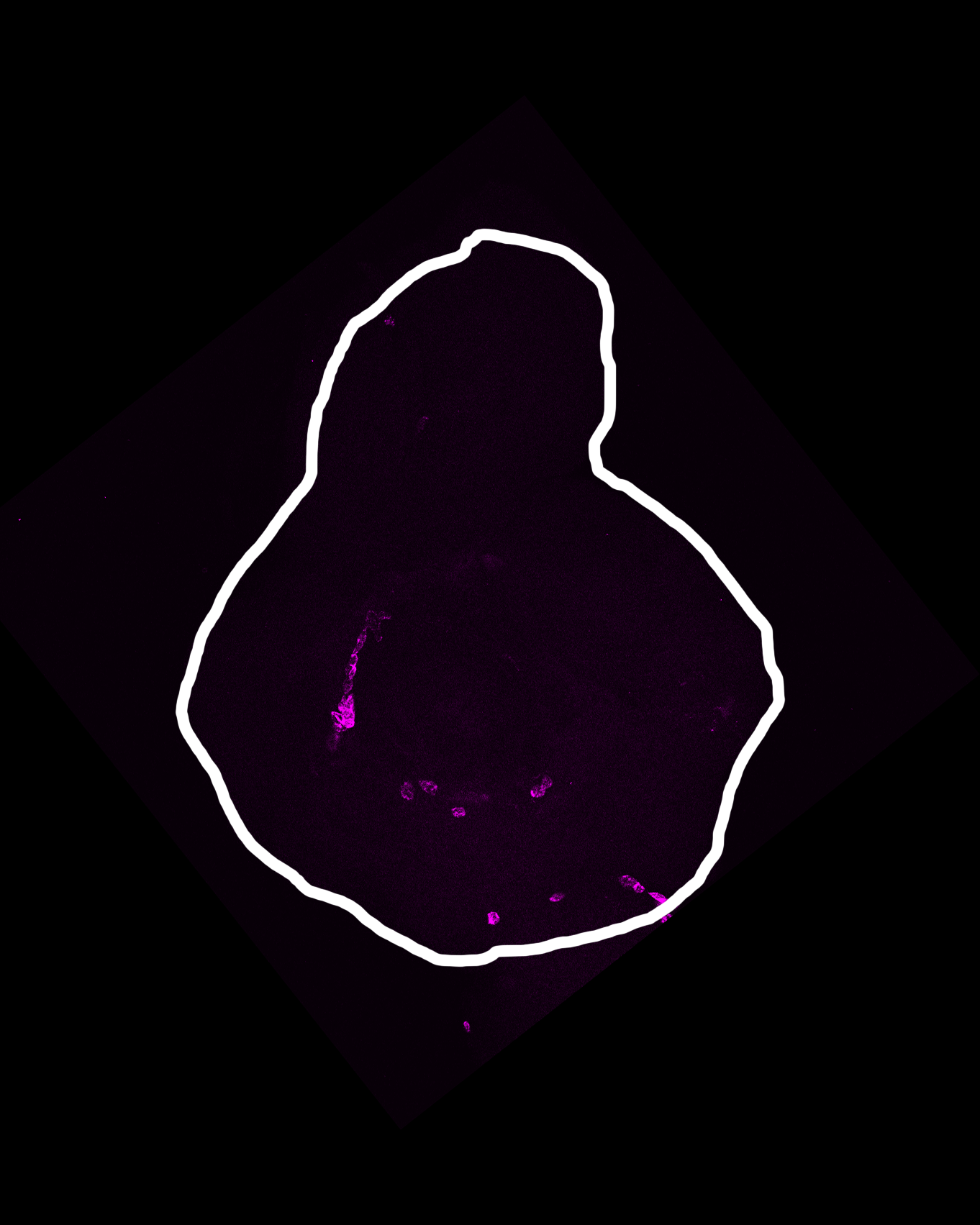

Supplement: Supplementary file 10 — Source data Fig. 6 [file 44318_2025_547_MOESM10_ESM.zip › Figure 6E/2-1 rotated and cut image.tif]

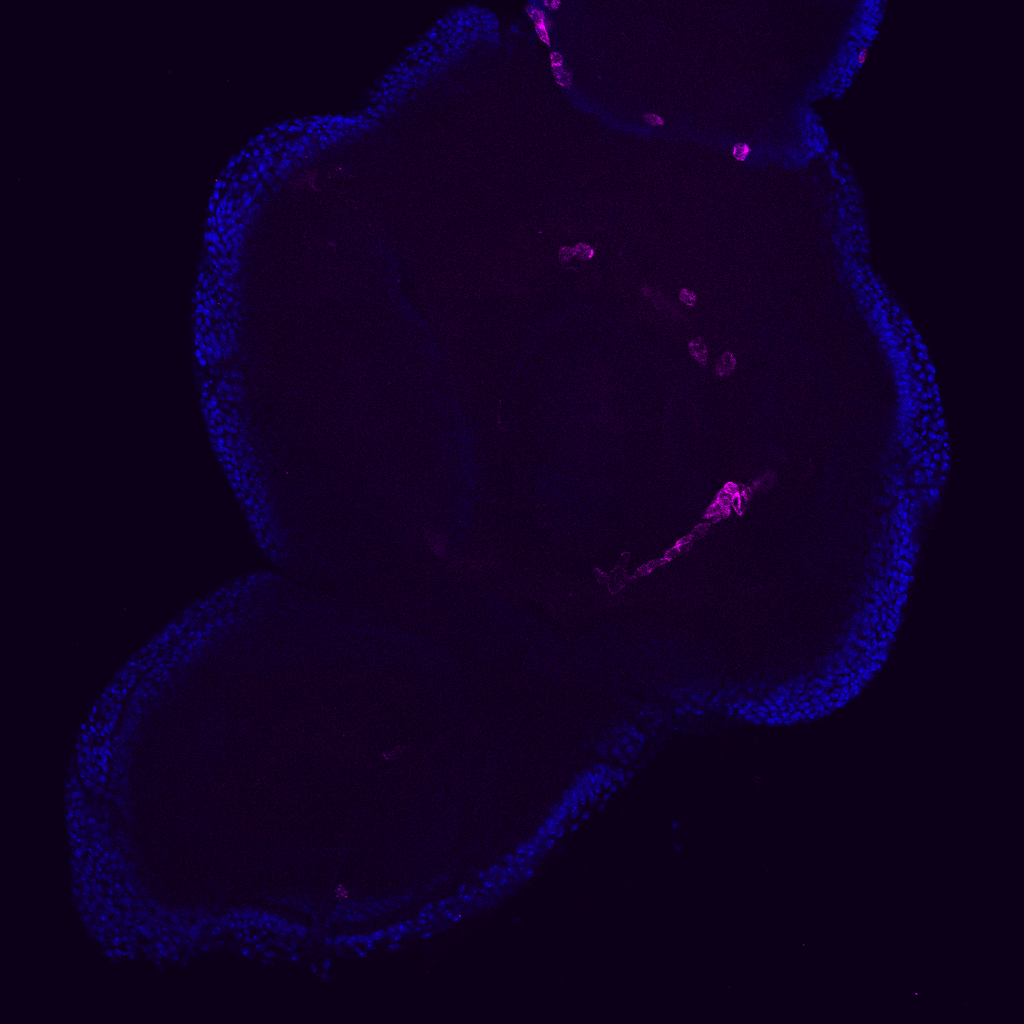

Supplement: Supplementary file 10 — Source data Fig. 6 [file 44318_2025_547_MOESM10_ESM.zip › Figure 6E/2-2 original image.tif]

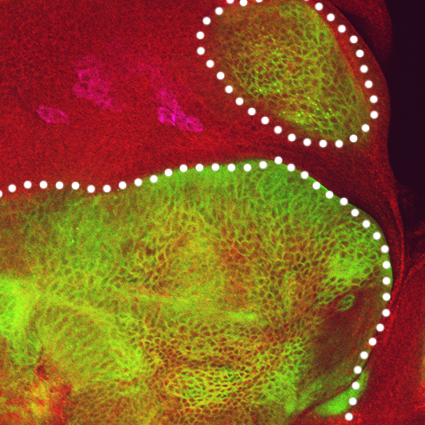

Supplement: Supplementary file 10 — Source data Fig. 6 [file 44318_2025_547_MOESM10_ESM.zip › Figure 6E/3-1 rotated and cut image.tif]

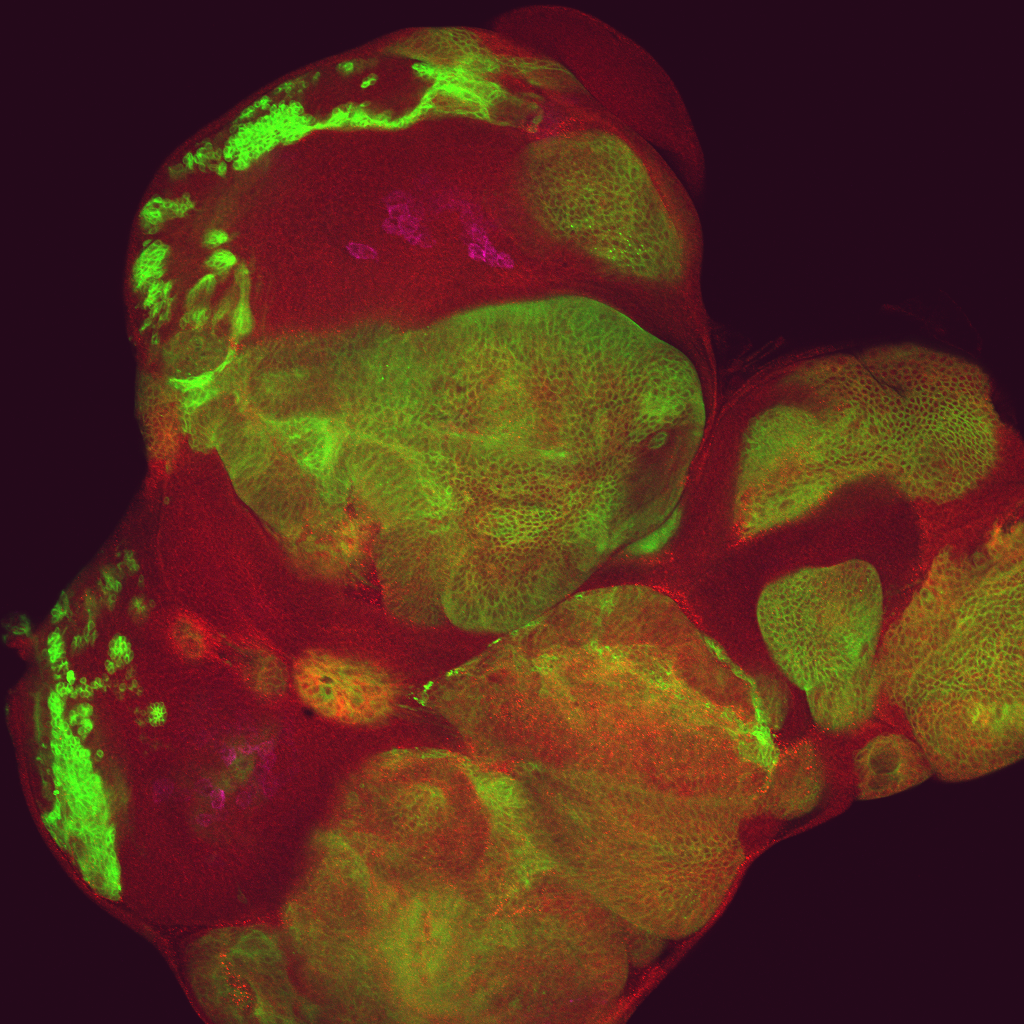

Supplement: Supplementary file 10 — Source data Fig. 6 [file 44318_2025_547_MOESM10_ESM.zip › Figure 6E/3-2 original image.tif]

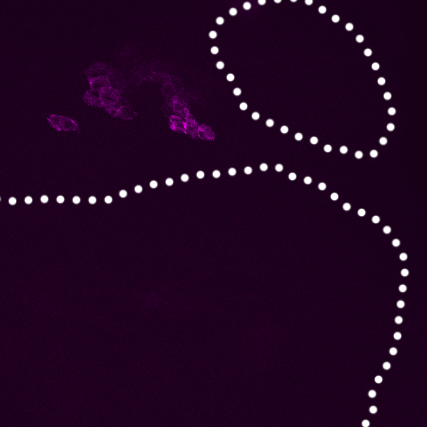

Supplement: Supplementary file 10 — Source data Fig. 6 [file 44318_2025_547_MOESM10_ESM.zip › Figure 6E/4-1 rotated and cut image.tif]

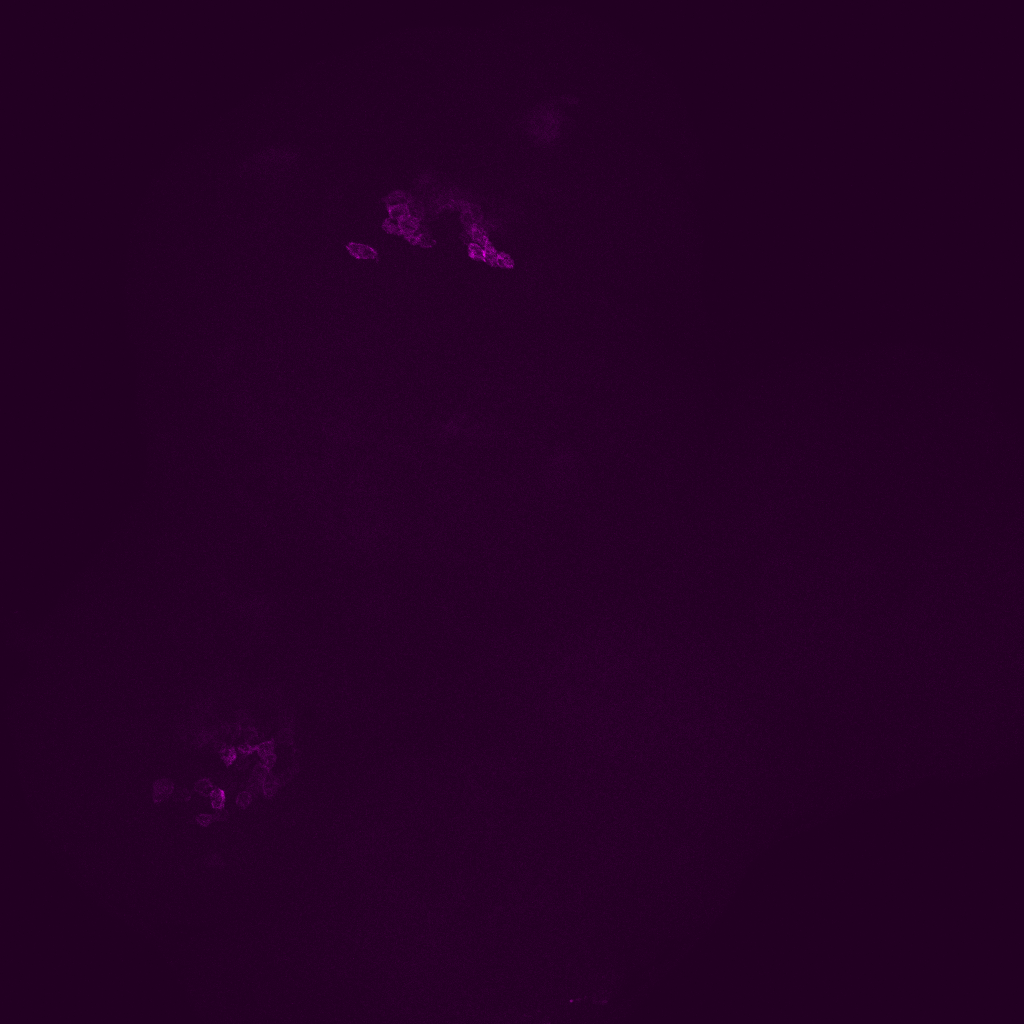

Supplement: Supplementary file 10 — Source data Fig. 6 [file 44318_2025_547_MOESM10_ESM.zip › Figure 6E/4-2 original image.tif]

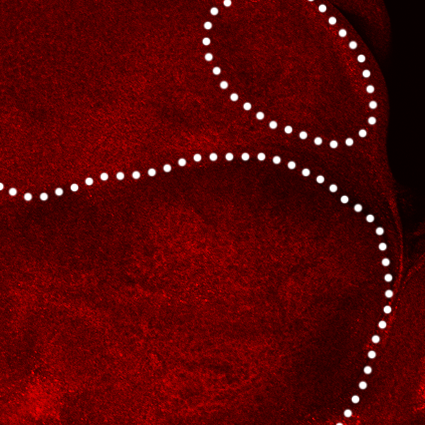

Supplement: Supplementary file 10 — Source data Fig. 6 [file 44318_2025_547_MOESM10_ESM.zip › Figure 6E/5-1 rotated and cut image.tif]

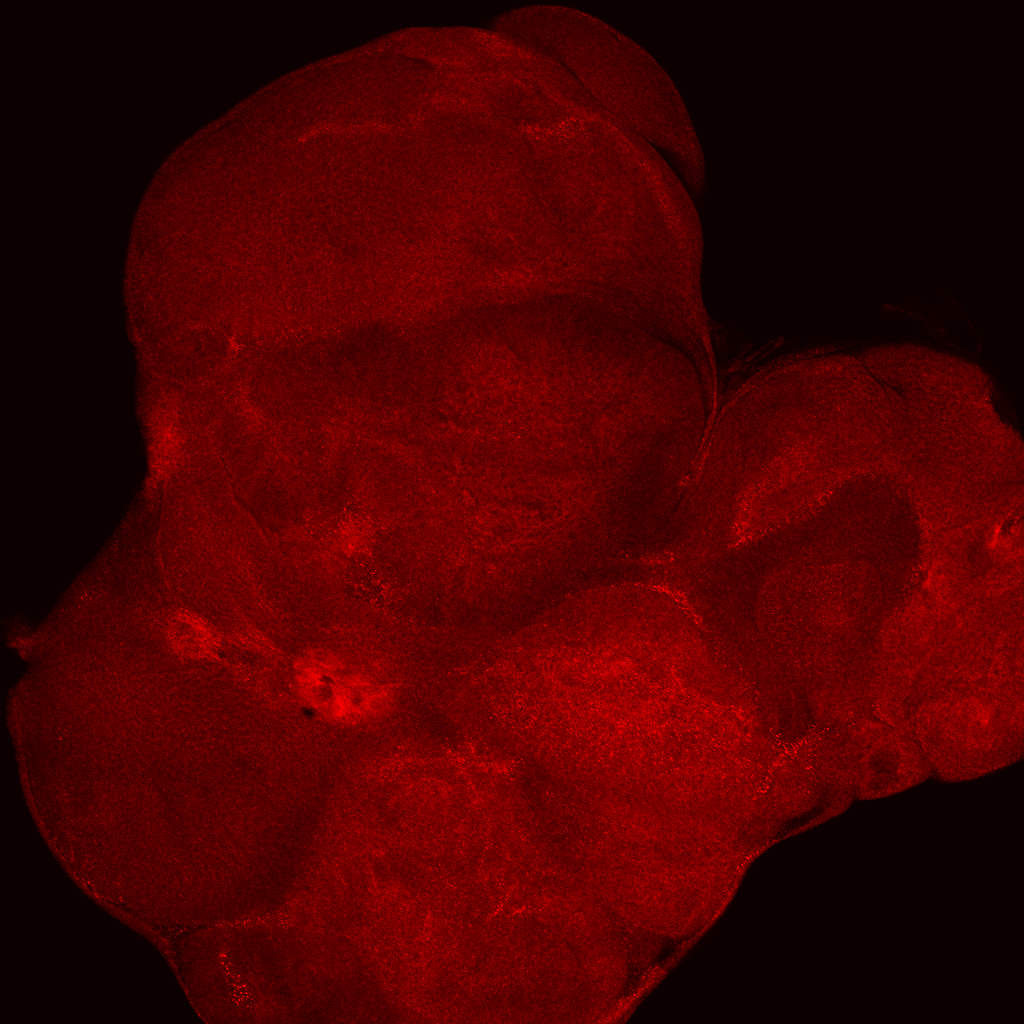

Supplement: Supplementary file 10 — Source data Fig. 6 [file 44318_2025_547_MOESM10_ESM.zip › Figure 6E/5-2 original image.tif]

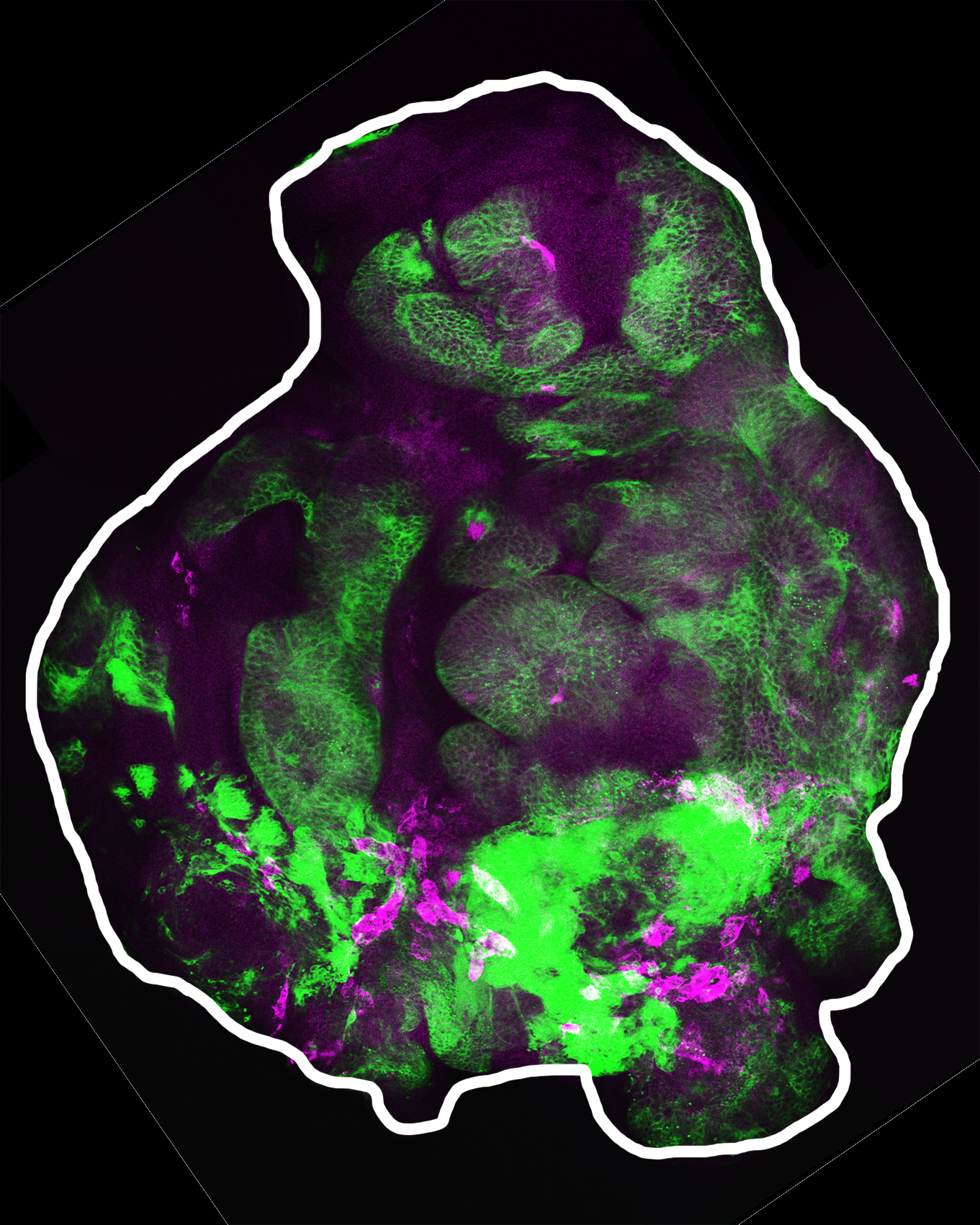

Supplement: Supplementary file 10 — Source data Fig. 6 [file 44318_2025_547_MOESM10_ESM.zip › Figure 6E/6-1 rotated and cut image.tif]

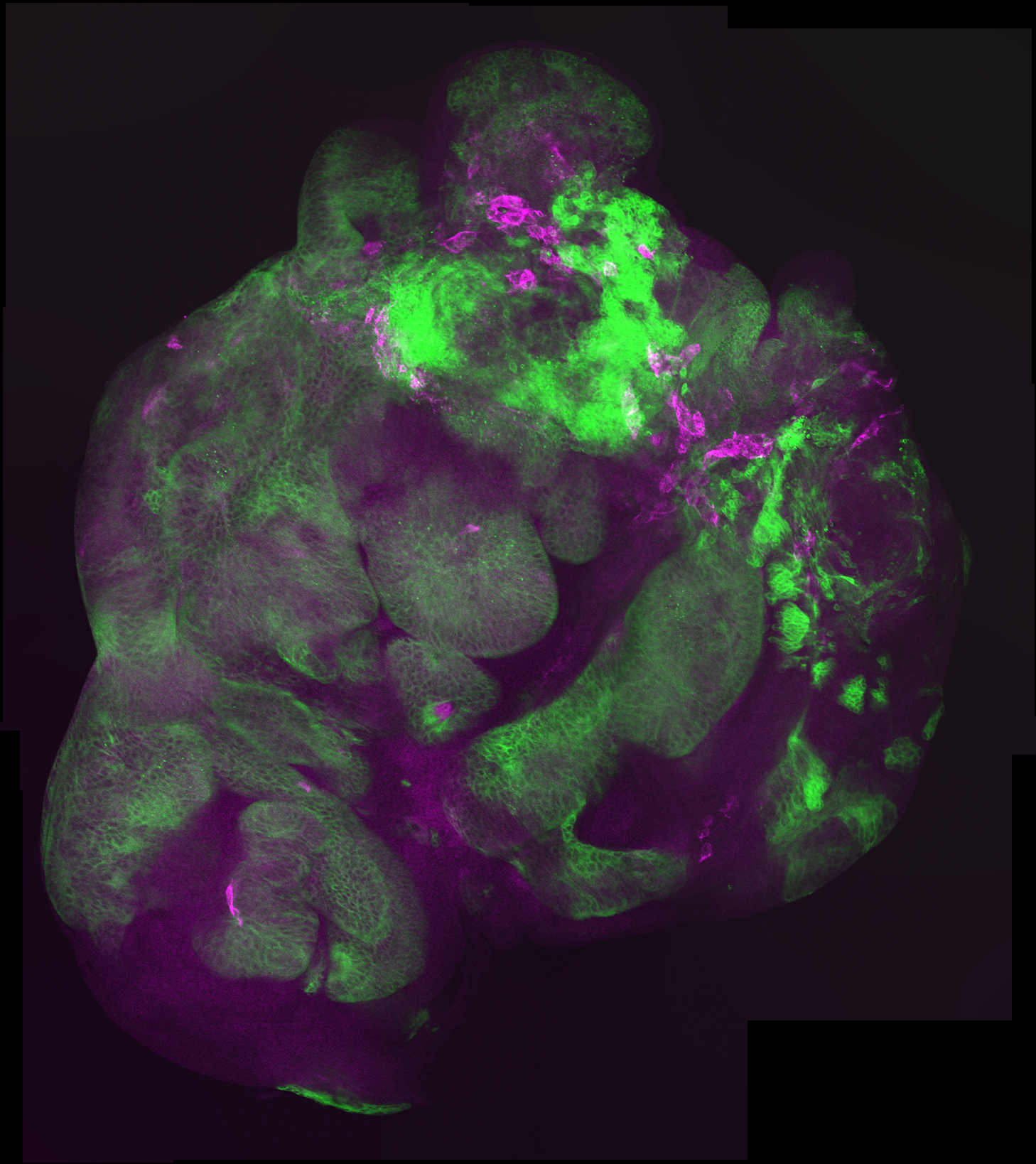

Supplement: Supplementary file 10 — Source data Fig. 6 [file 44318_2025_547_MOESM10_ESM.zip › Figure 6E/6-2 original image.tif]

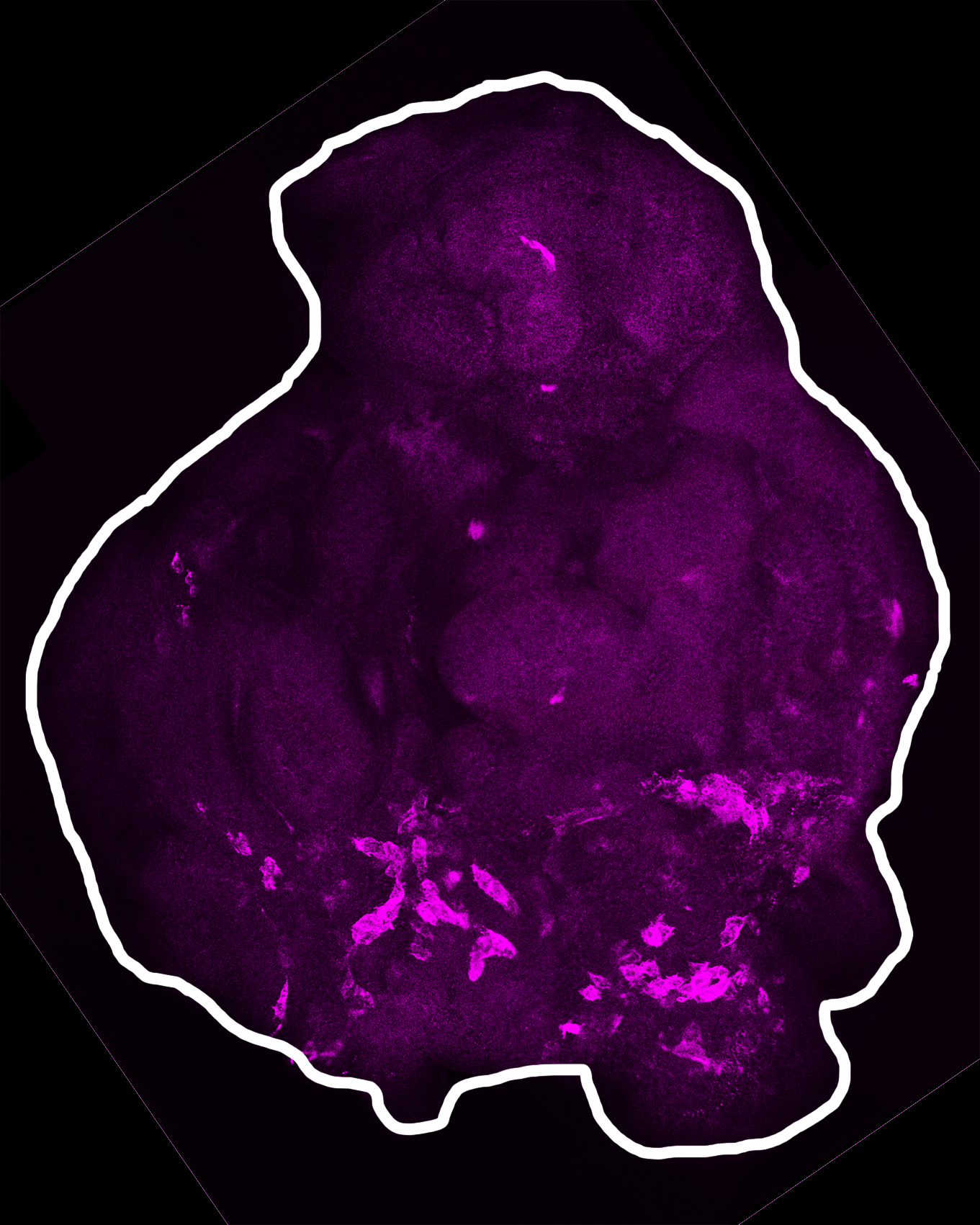

Supplement: Supplementary file 10 — Source data Fig. 6 [file 44318_2025_547_MOESM10_ESM.zip › Figure 6E/7-1 rotated and cut image.tif]

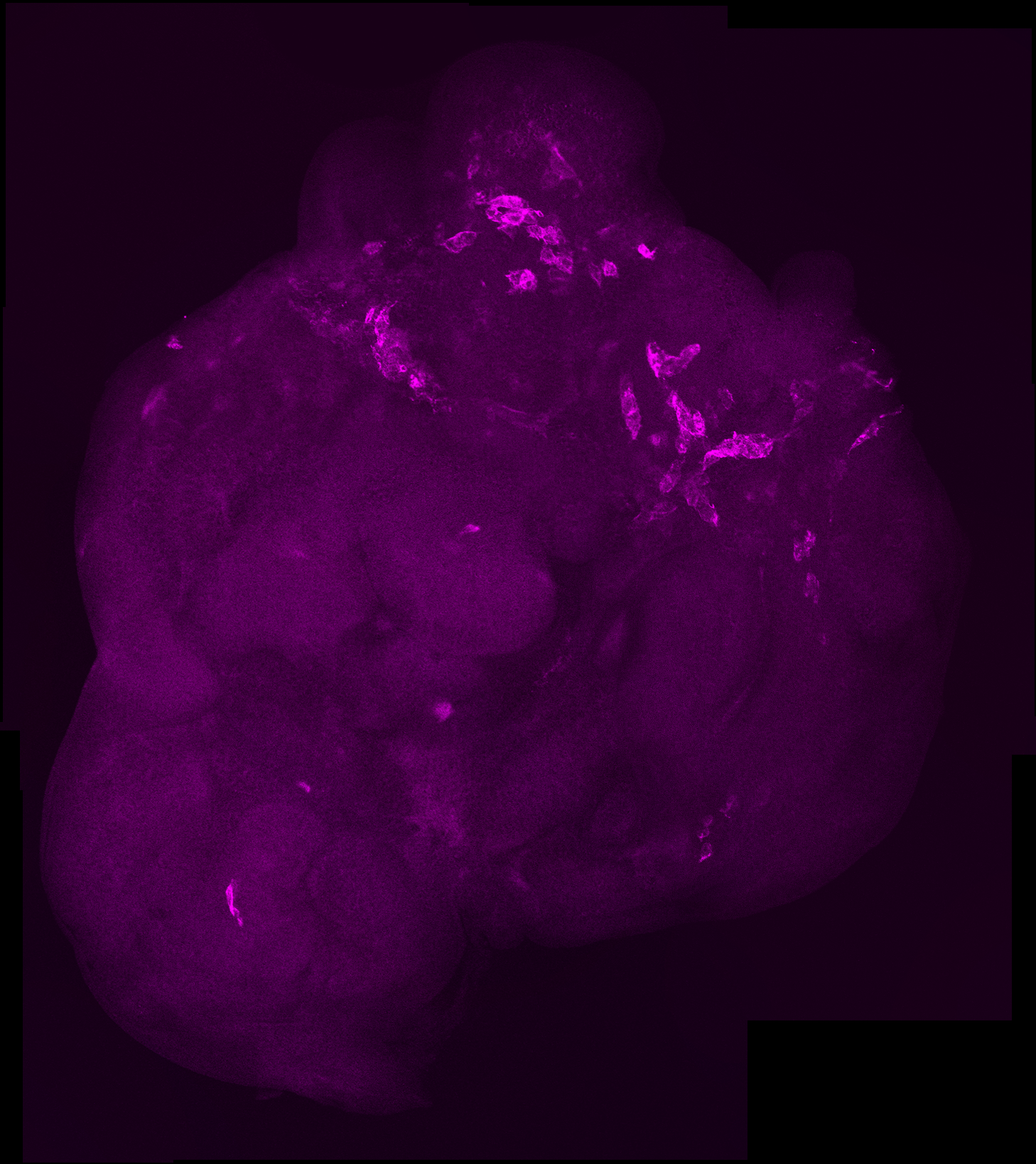

Supplement: Supplementary file 10 — Source data Fig. 6 [file 44318_2025_547_MOESM10_ESM.zip › Figure 6E/7-2 original image.tif]

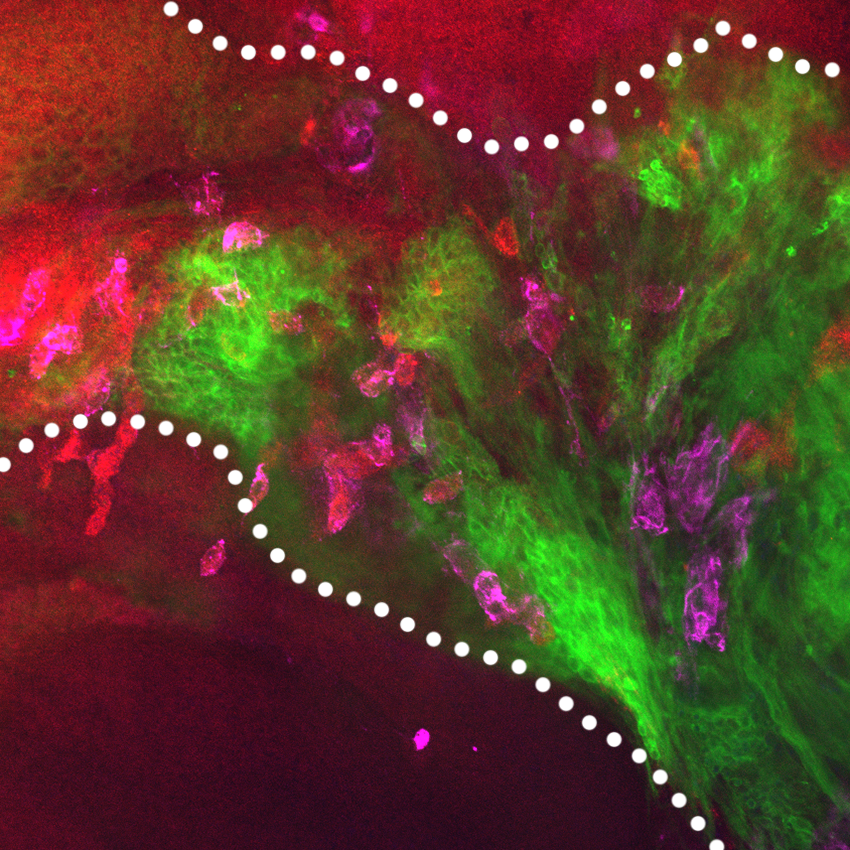

Supplement: Supplementary file 10 — Source data Fig. 6 [file 44318_2025_547_MOESM10_ESM.zip › Figure 6E/8-1 rotated and cut image.tif]

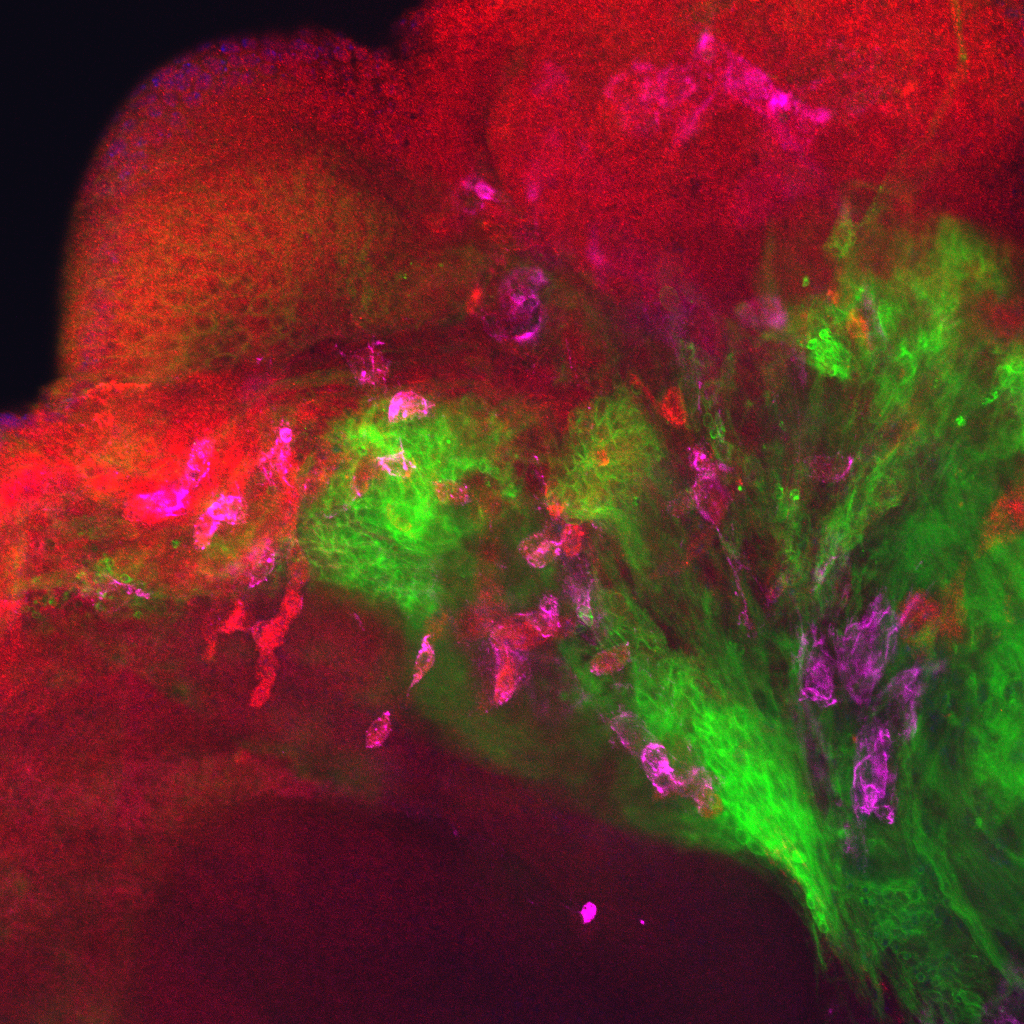

Supplement: Supplementary file 10 — Source data Fig. 6 [file 44318_2025_547_MOESM10_ESM.zip › Figure 6E/8-2 original image.tif]

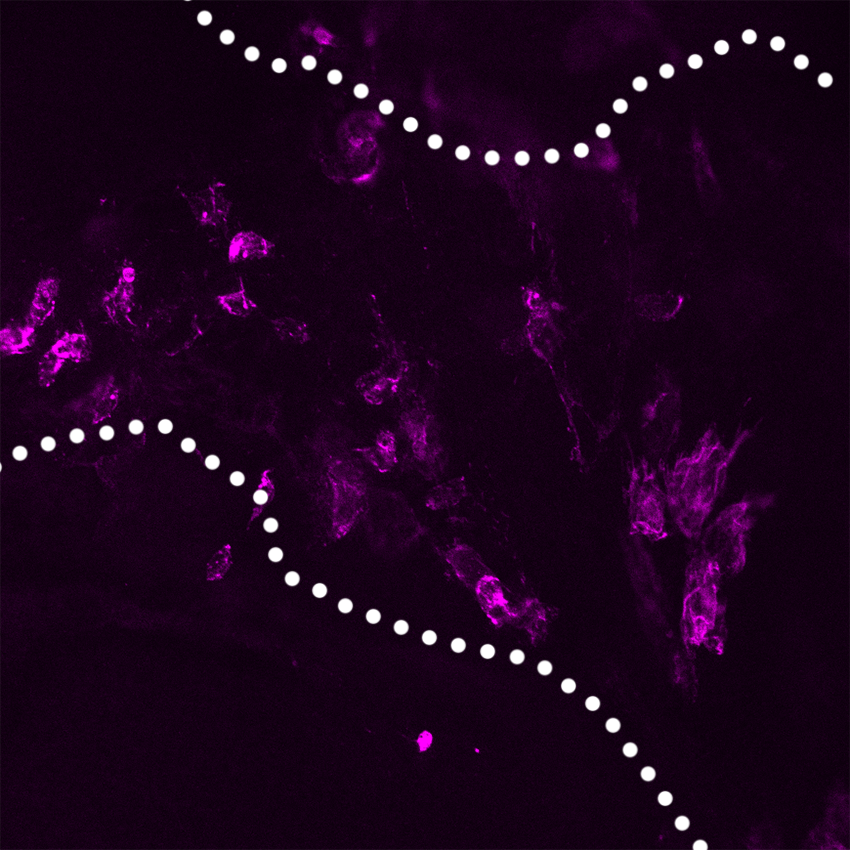

Supplement: Supplementary file 10 — Source data Fig. 6 [file 44318_2025_547_MOESM10_ESM.zip › Figure 6E/9-1 rotated and cut image.tif]

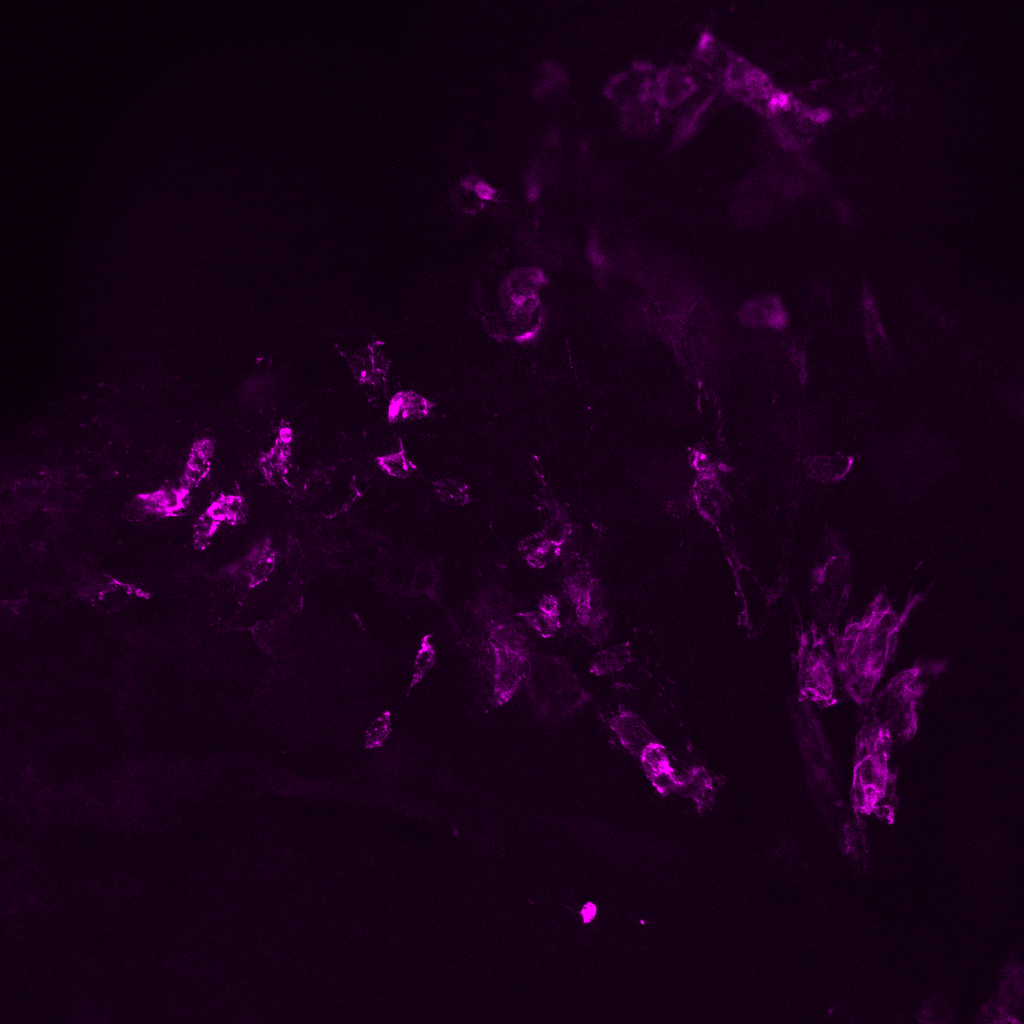

Supplement: Supplementary file 10 — Source data Fig. 6 [file 44318_2025_547_MOESM10_ESM.zip › Figure 6E/9-2 original image.tif]

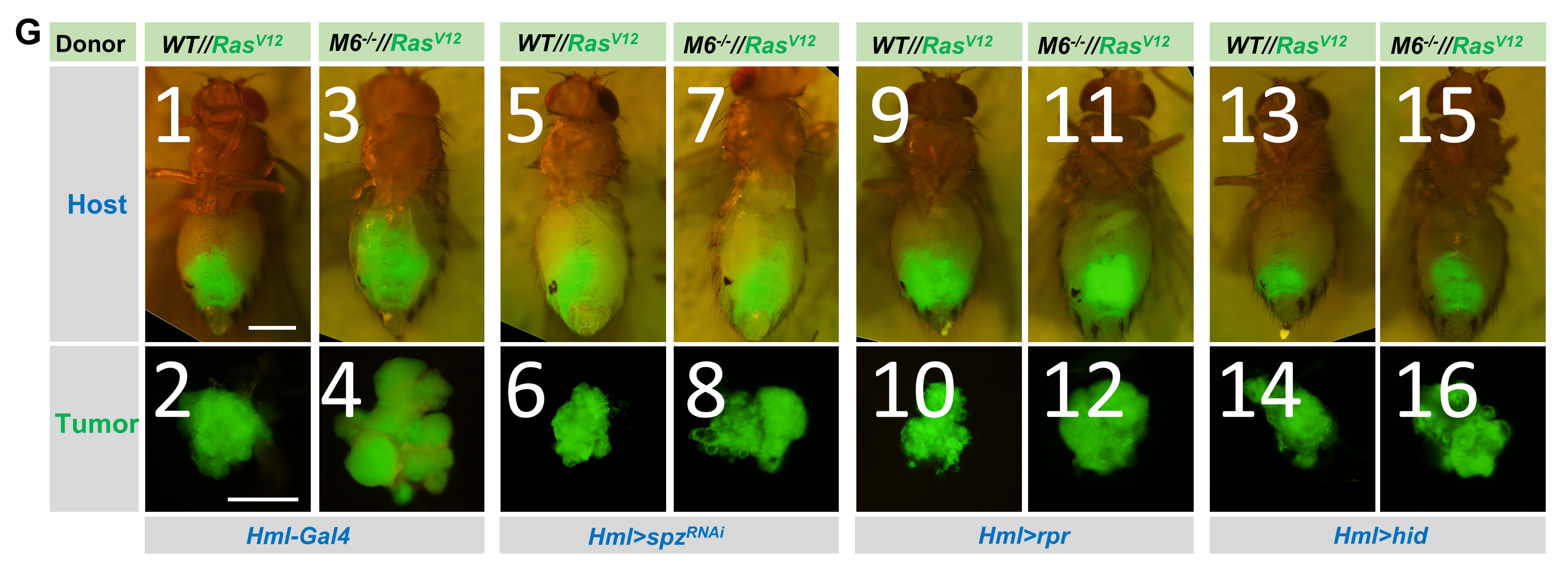

Supplement: Supplementary file 10 — Source data Fig. 6 [file 44318_2025_547_MOESM10_ESM.zip › Figure 6G/0 paper Figure 6G with provided image sequence.tif]

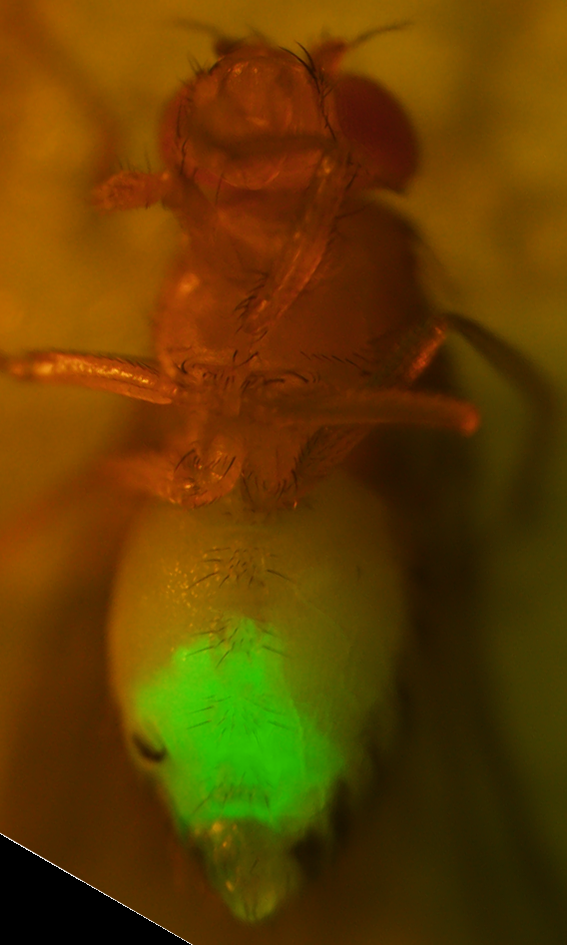

Supplement: Supplementary file 10 — Source data Fig. 6 [file 44318_2025_547_MOESM10_ESM.zip › Figure 6G/1-1 rotated and cut image.tif]

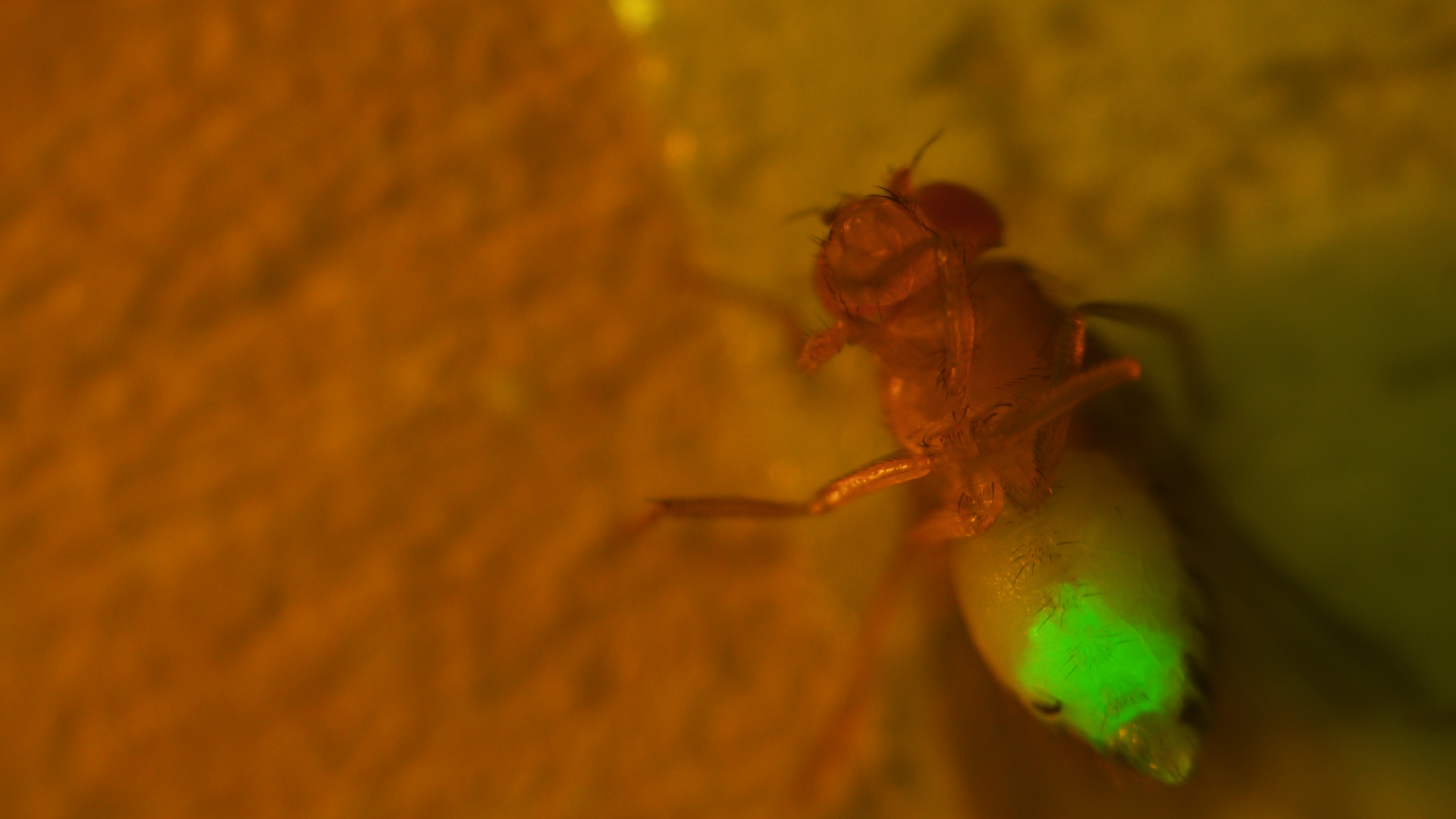

Supplement: Supplementary file 10 — Source data Fig. 6 [file 44318_2025_547_MOESM10_ESM.zip › Figure 6G/1-2 original image.tif]

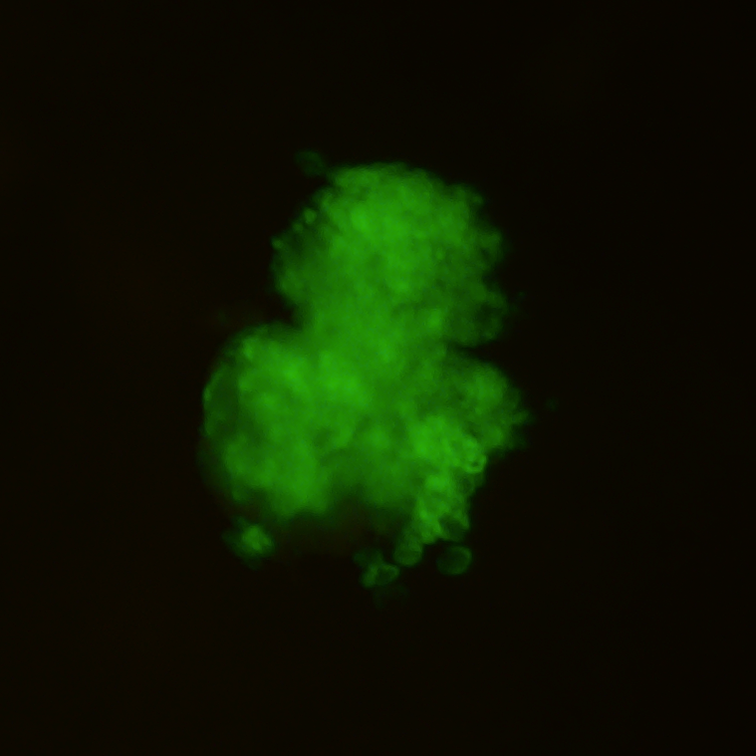

Supplement: Supplementary file 10 — Source data Fig. 6 [file 44318_2025_547_MOESM10_ESM.zip › Figure 6G/10-1 rotated and cut image.tif]

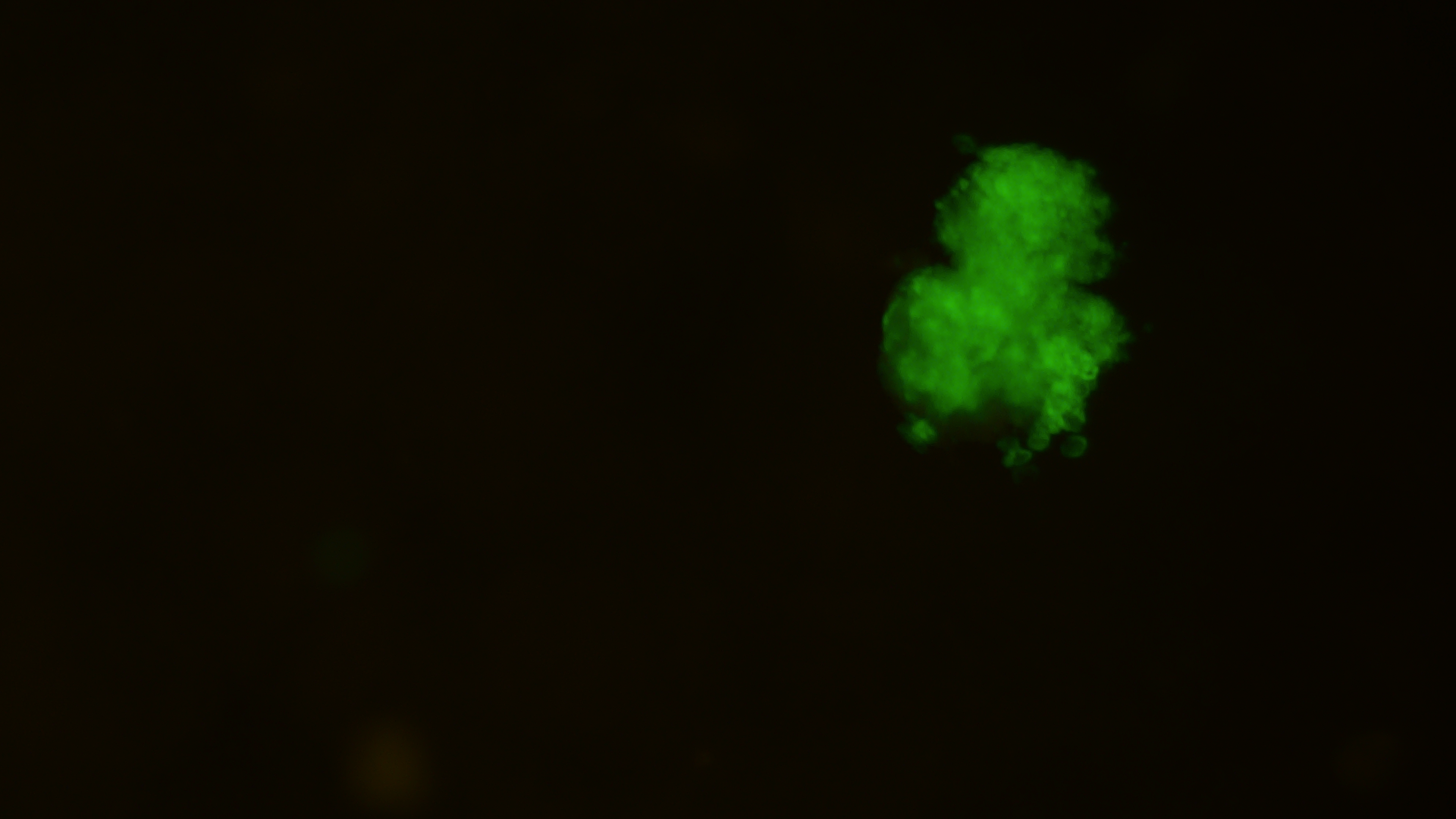

Supplement: Supplementary file 10 — Source data Fig. 6 [file 44318_2025_547_MOESM10_ESM.zip › Figure 6G/10-2 original image.tif]

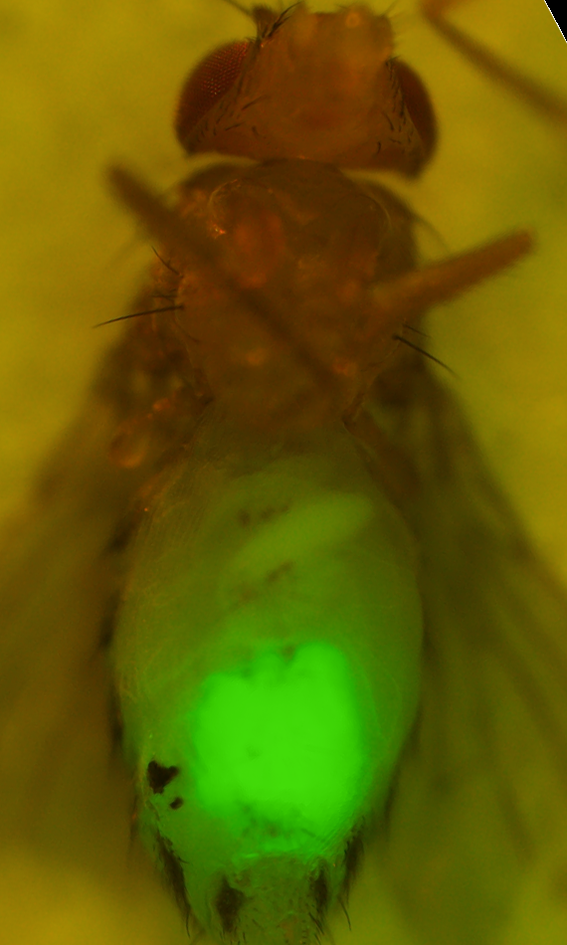

Supplement: Supplementary file 10 — Source data Fig. 6 [file 44318_2025_547_MOESM10_ESM.zip › Figure 6G/11-1 rotated and cut image.tif]

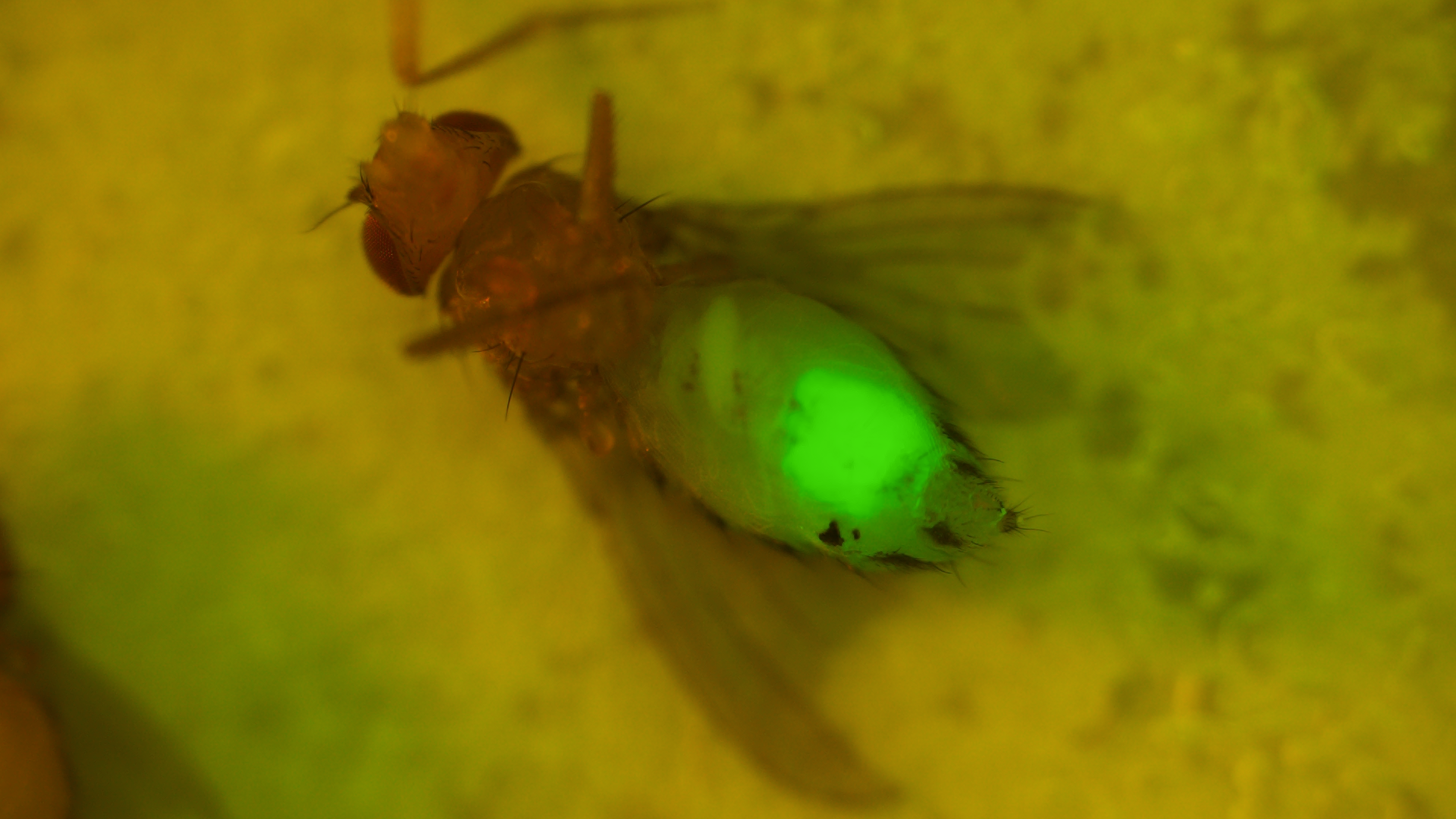

Supplement: Supplementary file 10 — Source data Fig. 6 [file 44318_2025_547_MOESM10_ESM.zip › Figure 6G/11-2 original image.tif]

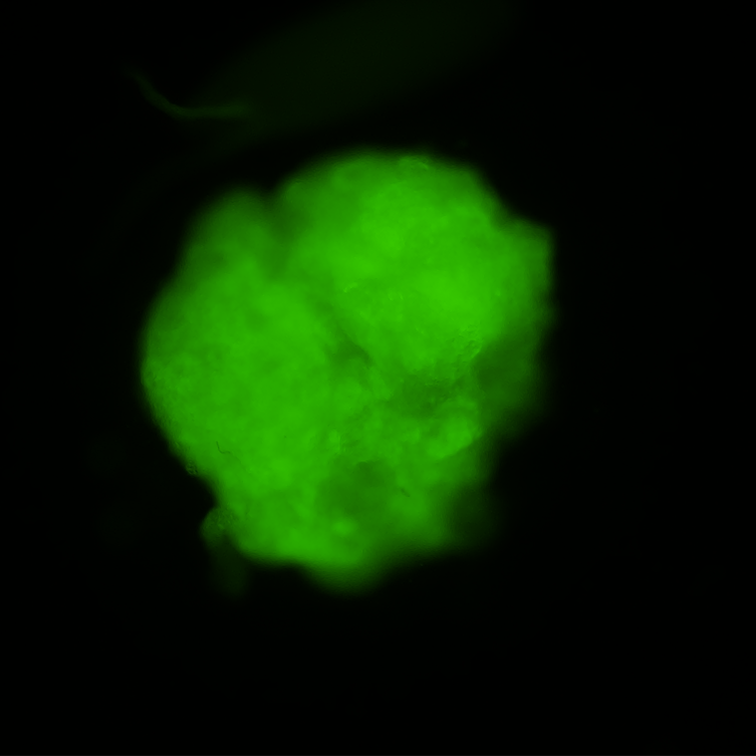

Supplement: Supplementary file 10 — Source data Fig. 6 [file 44318_2025_547_MOESM10_ESM.zip › Figure 6G/12-1 rotated and cut image.tif]

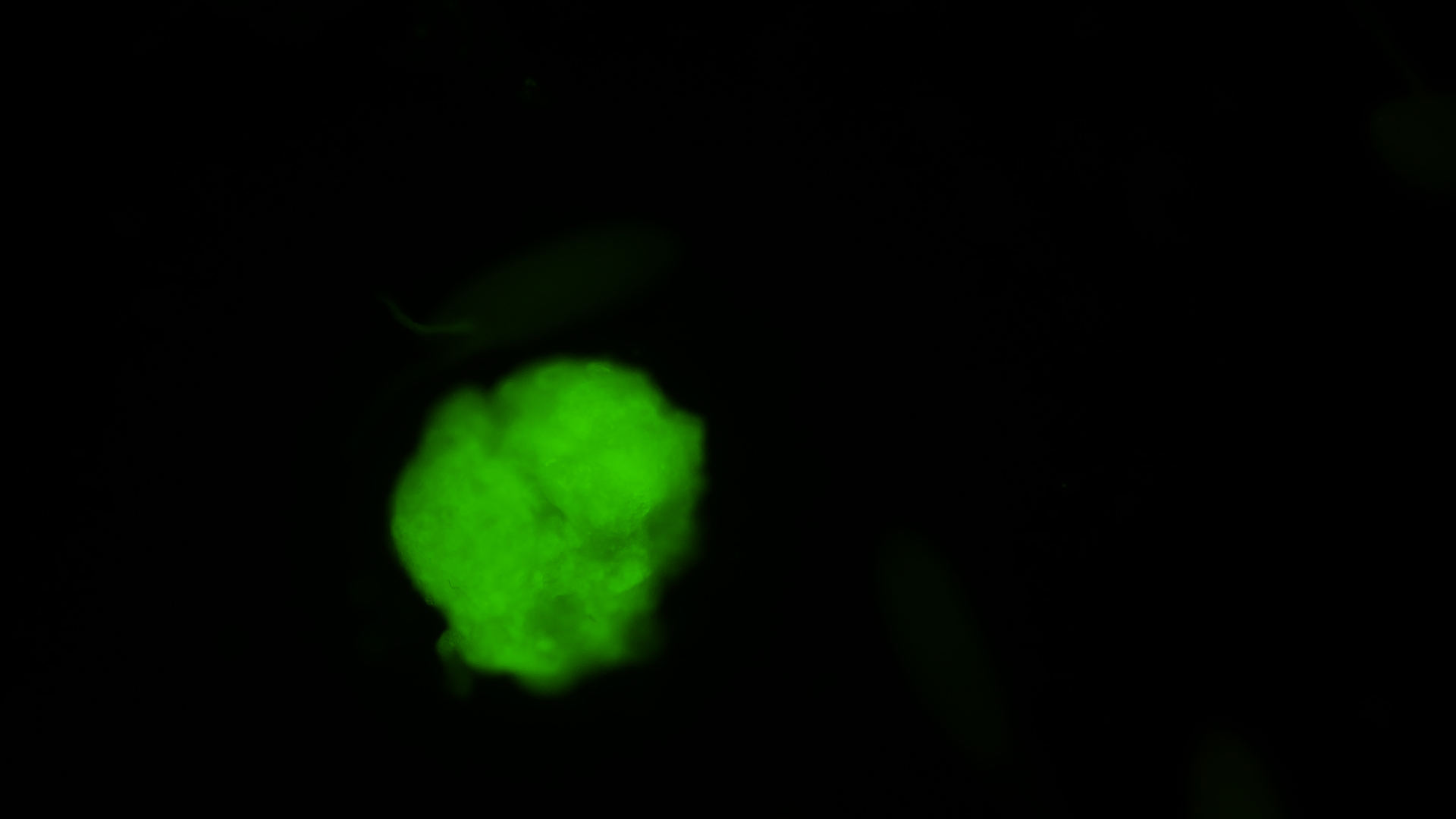

Supplement: Supplementary file 10 — Source data Fig. 6 [file 44318_2025_547_MOESM10_ESM.zip › Figure 6G/12-2 original image.tif]

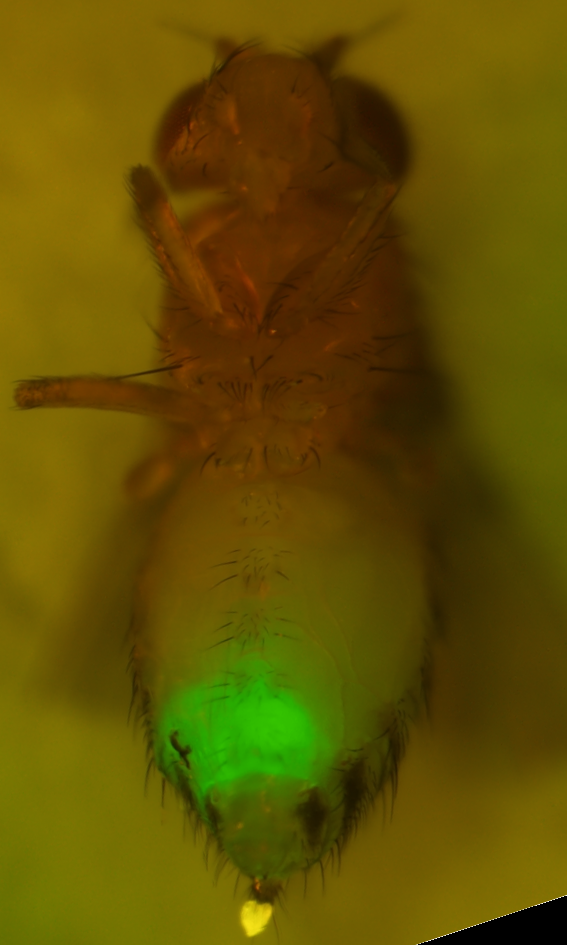

Supplement: Supplementary file 10 — Source data Fig. 6 [file 44318_2025_547_MOESM10_ESM.zip › Figure 6G/13-1 rotated and cut image.tif]

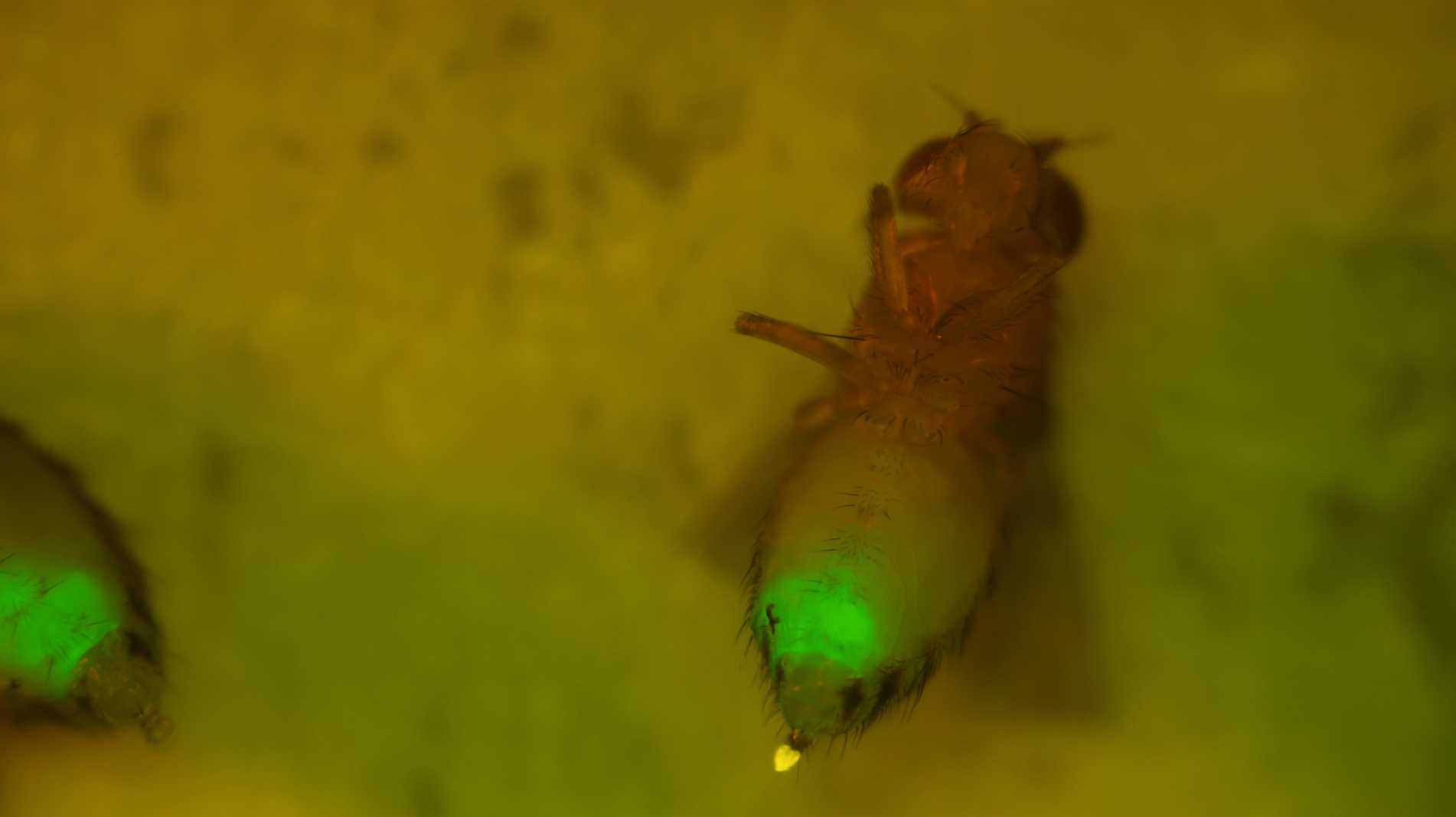

Supplement: Supplementary file 10 — Source data Fig. 6 [file 44318_2025_547_MOESM10_ESM.zip › Figure 6G/13-2 original image.tif]

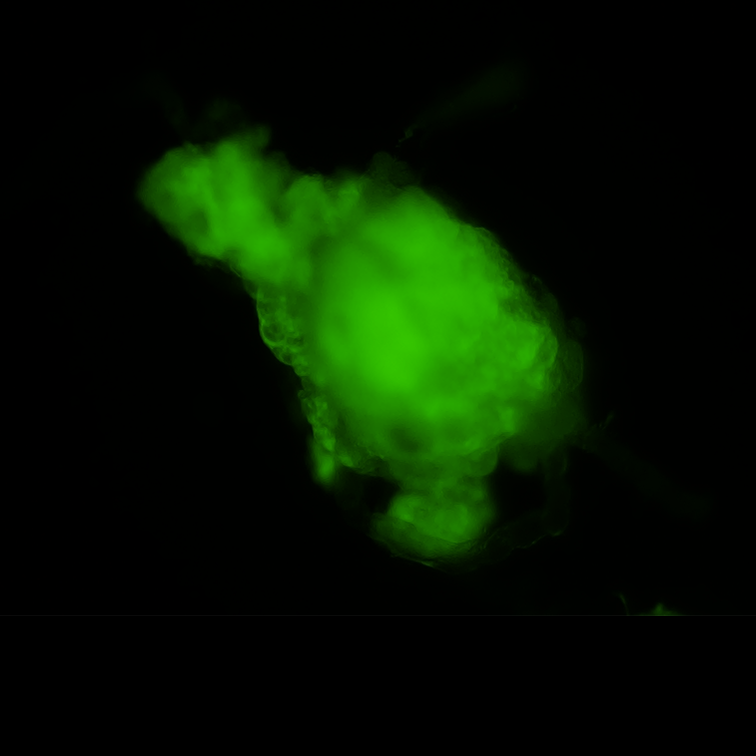

Supplement: Supplementary file 10 — Source data Fig. 6 [file 44318_2025_547_MOESM10_ESM.zip › Figure 6G/14-1 rotated and cut image.tif]

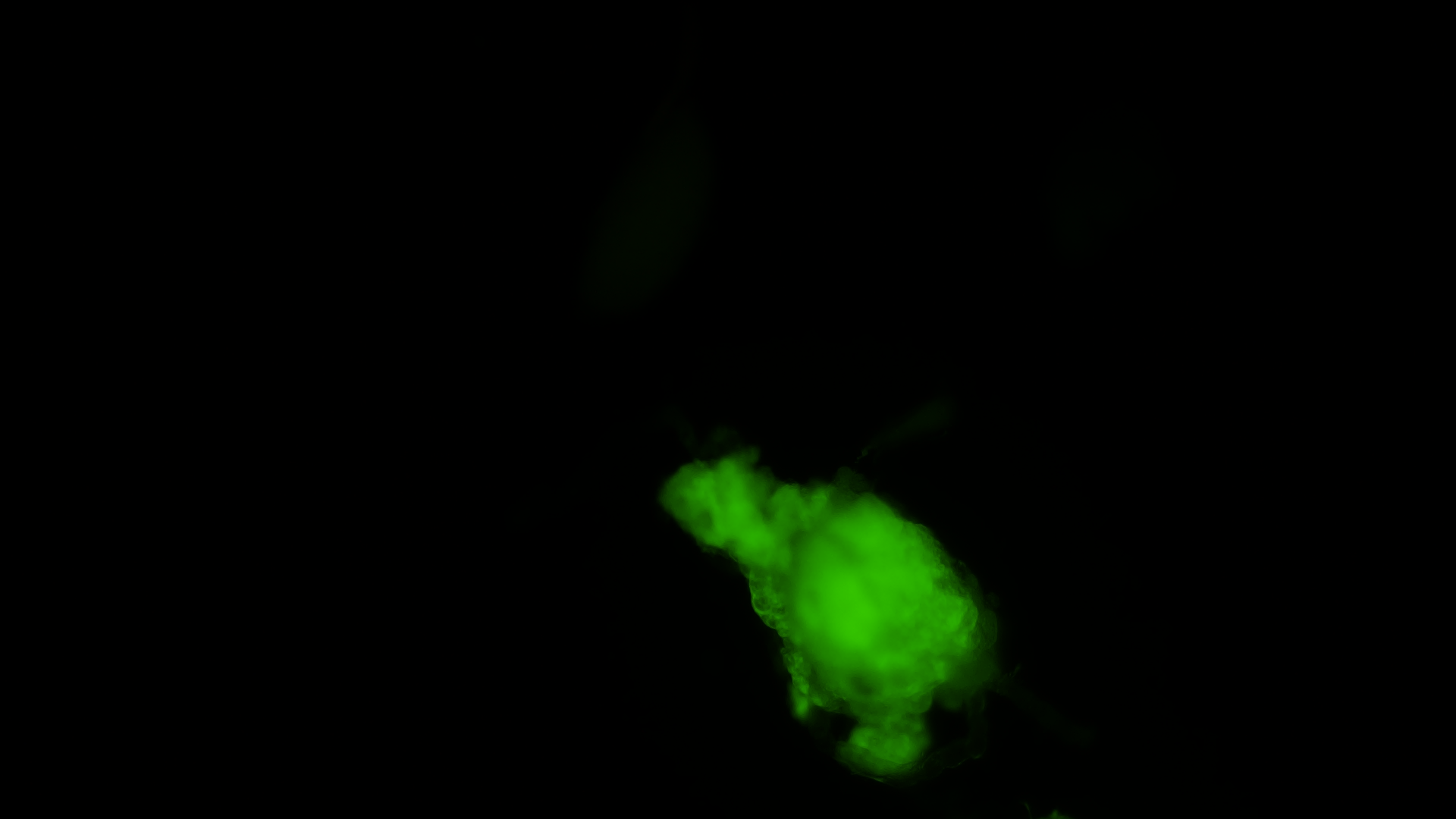

Supplement: Supplementary file 10 — Source data Fig. 6 [file 44318_2025_547_MOESM10_ESM.zip › Figure 6G/14-2 original image.tif]

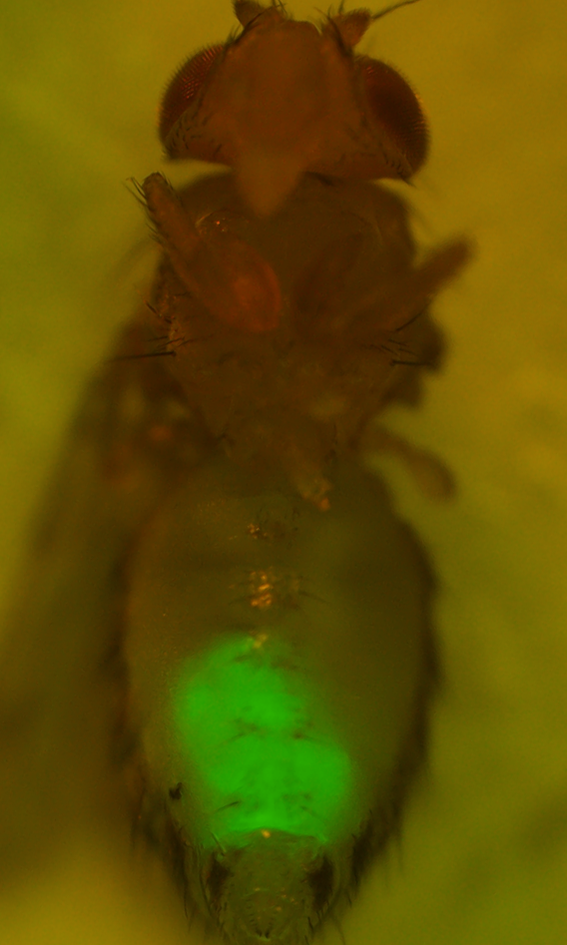

Supplement: Supplementary file 10 — Source data Fig. 6 [file 44318_2025_547_MOESM10_ESM.zip › Figure 6G/15-1 rotated and cut image.tif]

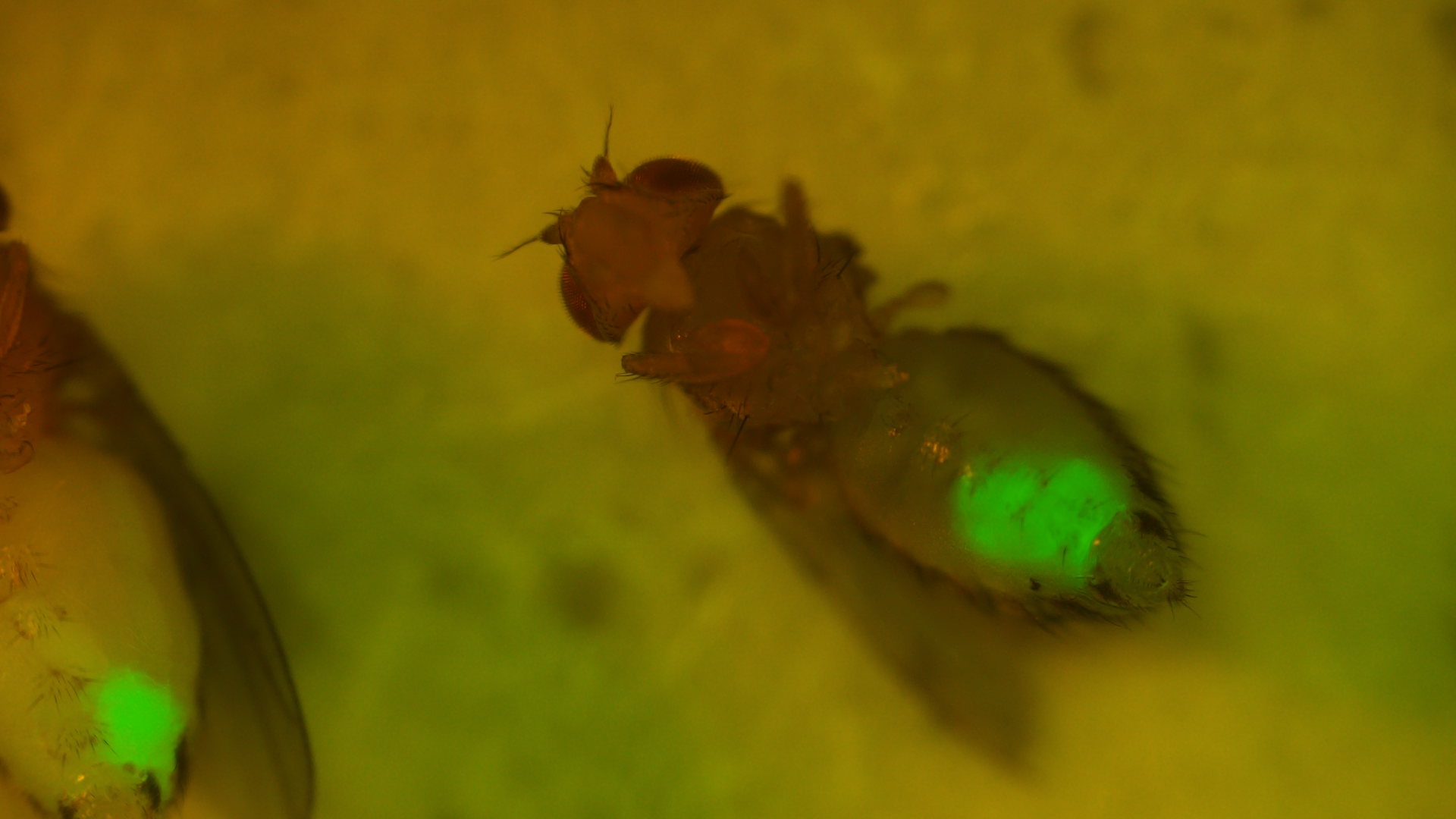

Supplement: Supplementary file 10 — Source data Fig. 6 [file 44318_2025_547_MOESM10_ESM.zip › Figure 6G/15-2 original image.tif]

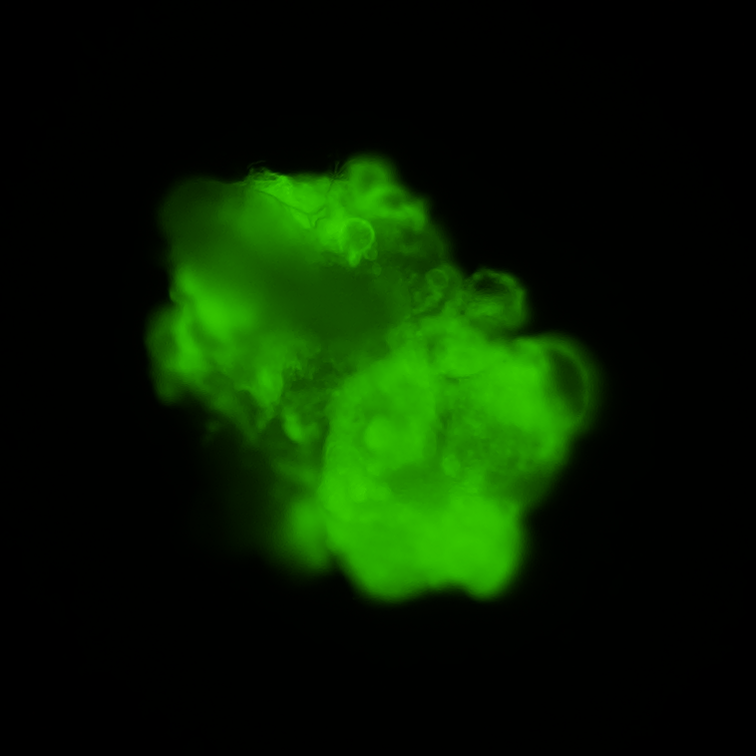

Supplement: Supplementary file 10 — Source data Fig. 6 [file 44318_2025_547_MOESM10_ESM.zip › Figure 6G/16-1 rotated and cut image.tif]

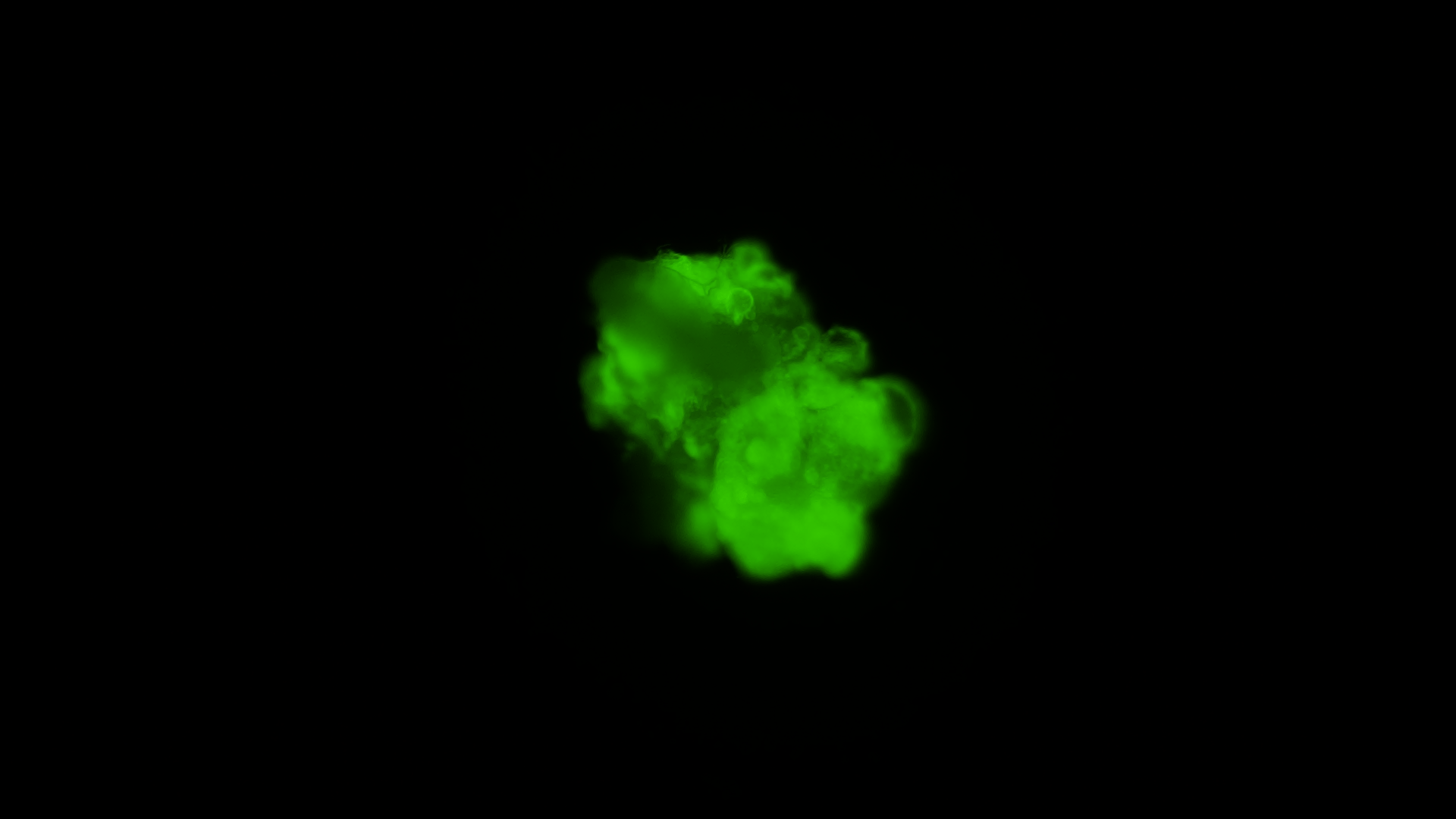

Supplement: Supplementary file 10 — Source data Fig. 6 [file 44318_2025_547_MOESM10_ESM.zip › Figure 6G/16-2 original image.tif]

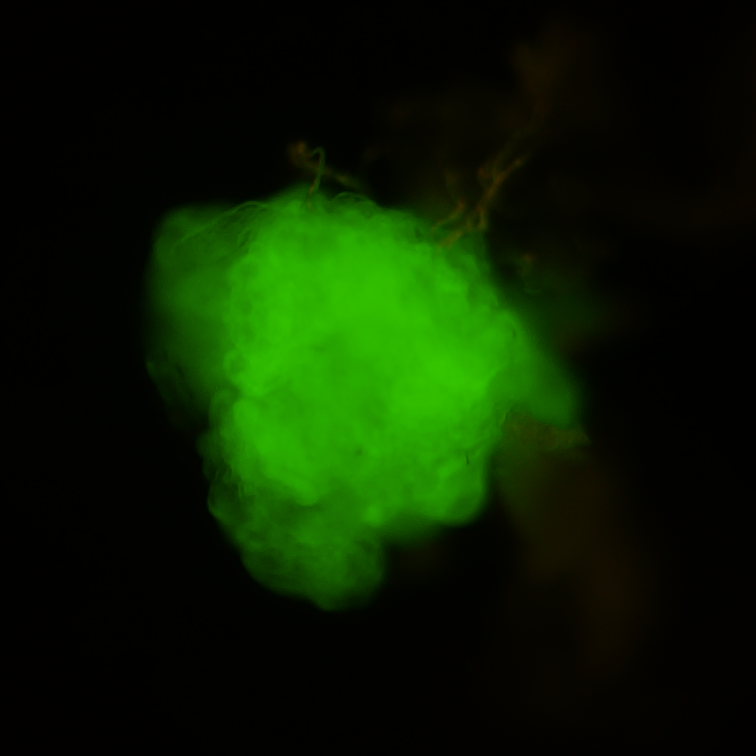

Supplement: Supplementary file 10 — Source data Fig. 6 [file 44318_2025_547_MOESM10_ESM.zip › Figure 6G/2-1 rotated and cut image.tif]

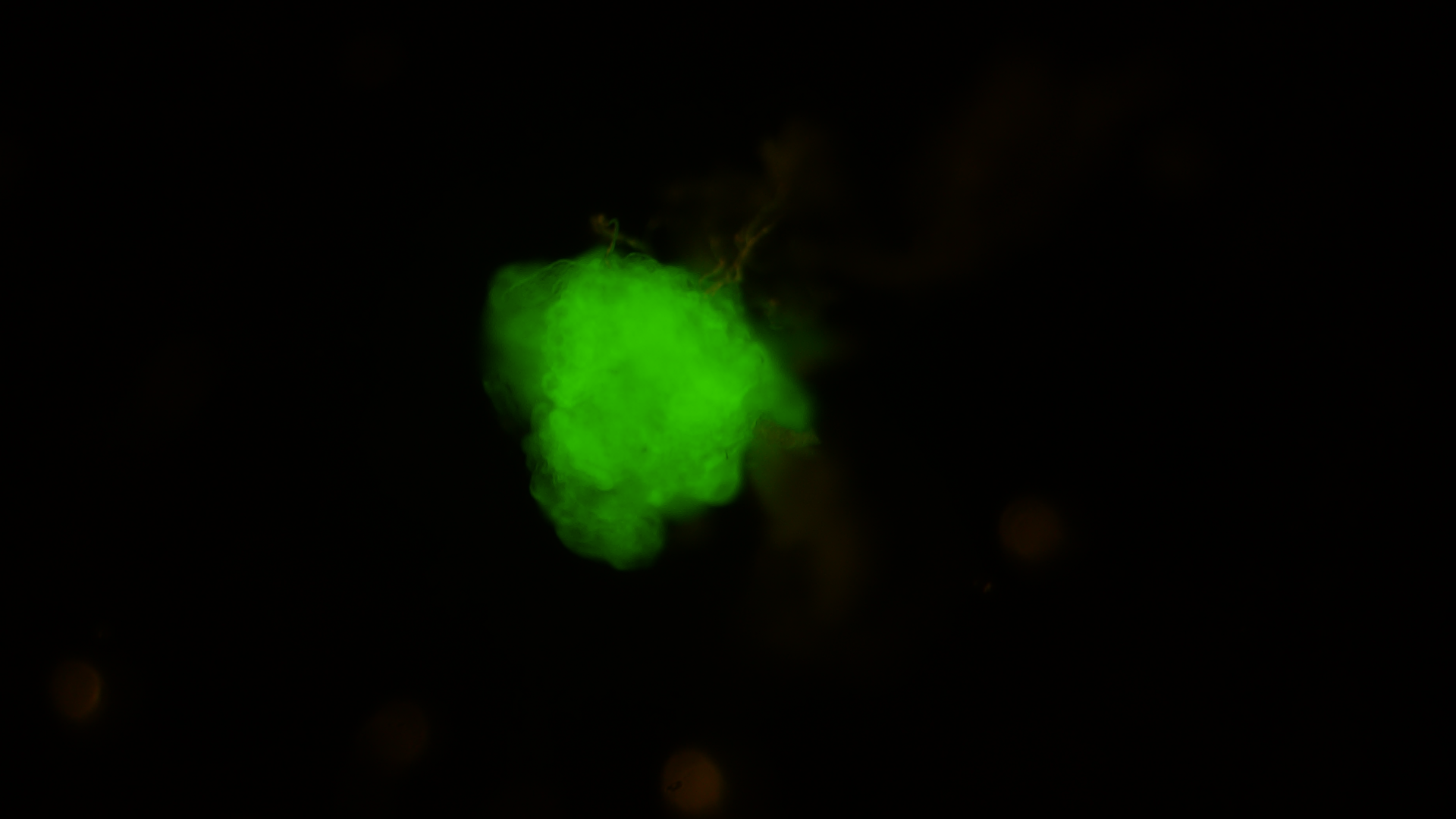

Supplement: Supplementary file 10 — Source data Fig. 6 [file 44318_2025_547_MOESM10_ESM.zip › Figure 6G/2-2 original image.tif]

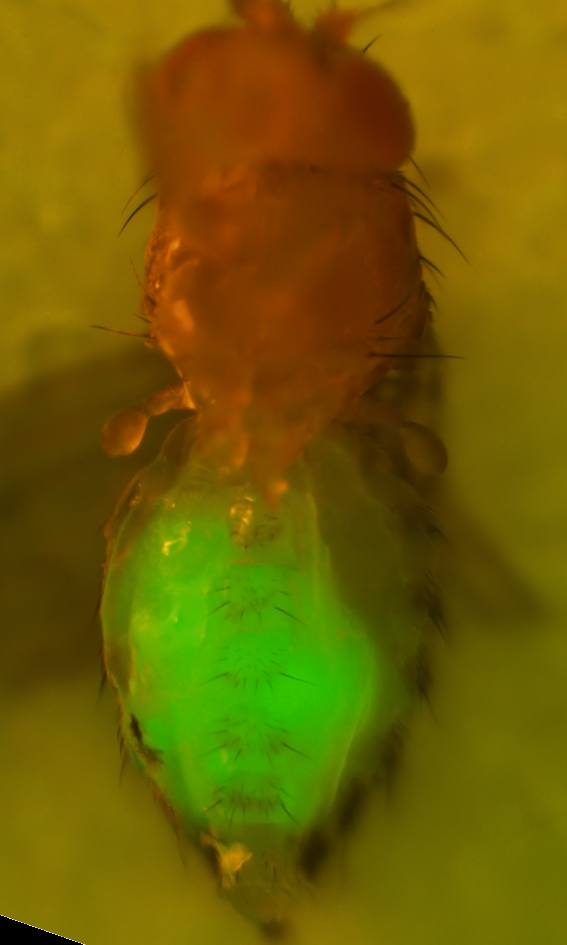

Supplement: Supplementary file 10 — Source data Fig. 6 [file 44318_2025_547_MOESM10_ESM.zip › Figure 6G/3-1 rotated and cut image.tif]

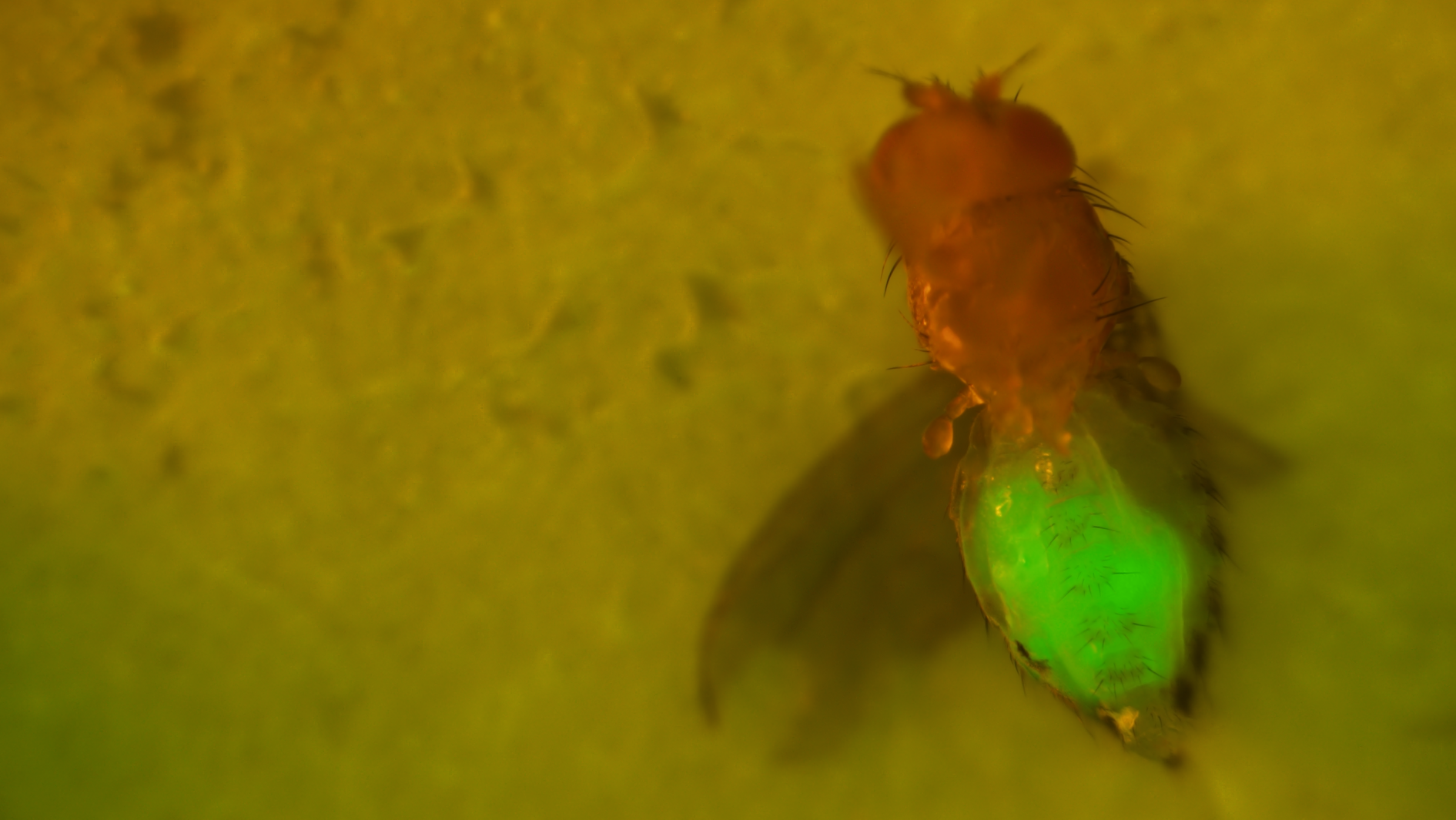

Supplement: Supplementary file 10 — Source data Fig. 6 [file 44318_2025_547_MOESM10_ESM.zip › Figure 6G/3-2 original image.tif]

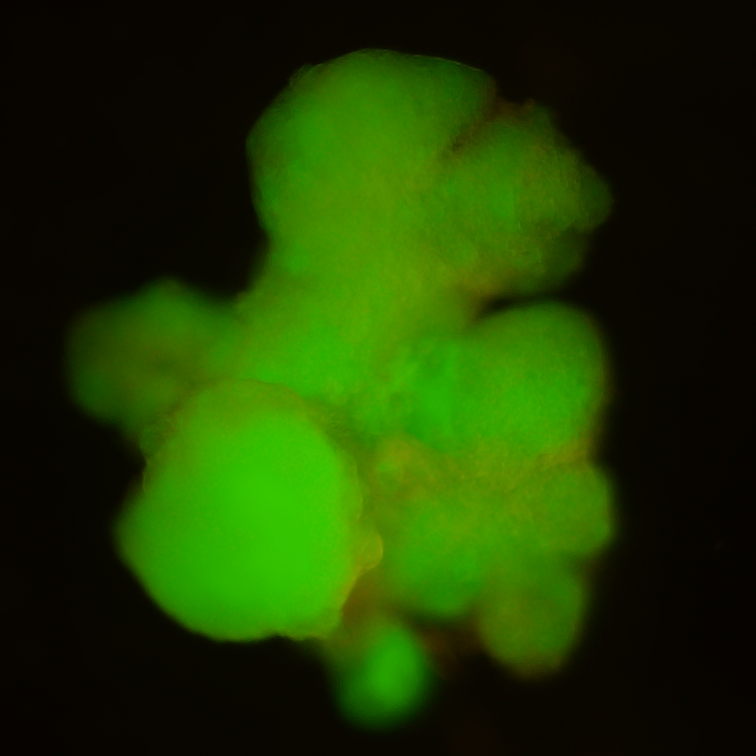

Supplement: Supplementary file 10 — Source data Fig. 6 [file 44318_2025_547_MOESM10_ESM.zip › Figure 6G/4-1 rotated and cut image.tif]

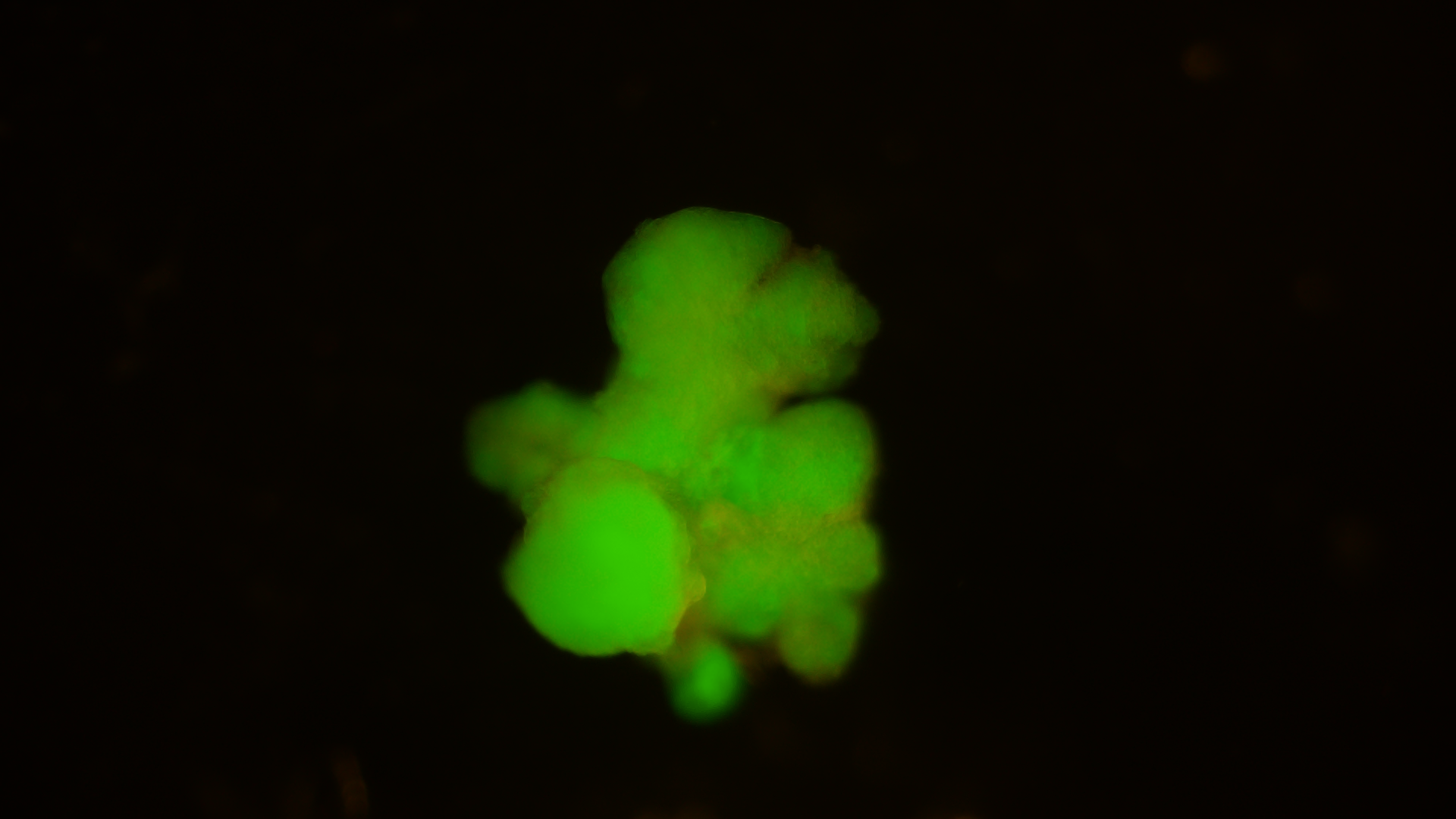

Supplement: Supplementary file 10 — Source data Fig. 6 [file 44318_2025_547_MOESM10_ESM.zip › Figure 6G/4-2 original image.tif]

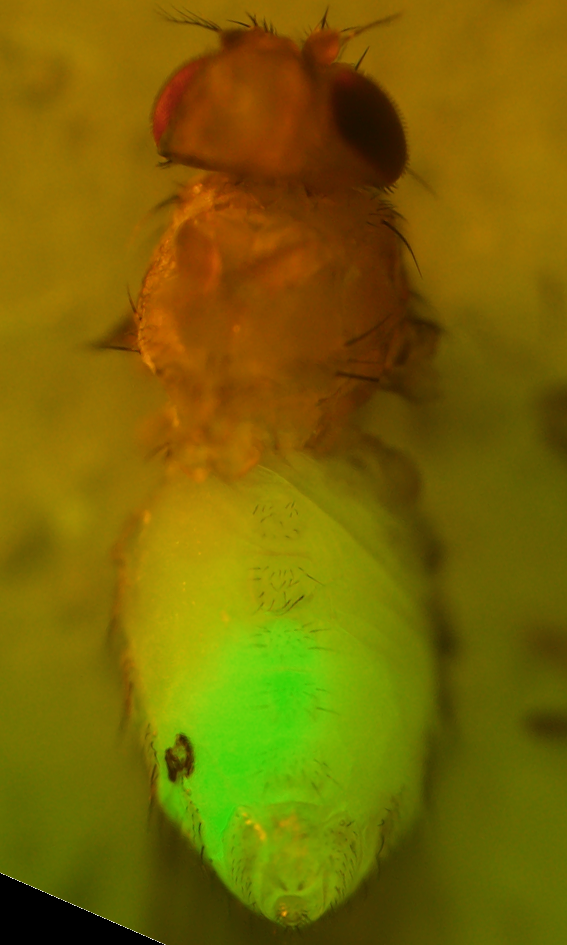

Supplement: Supplementary file 10 — Source data Fig. 6 [file 44318_2025_547_MOESM10_ESM.zip › Figure 6G/5-1 rotated and cut image.tif]

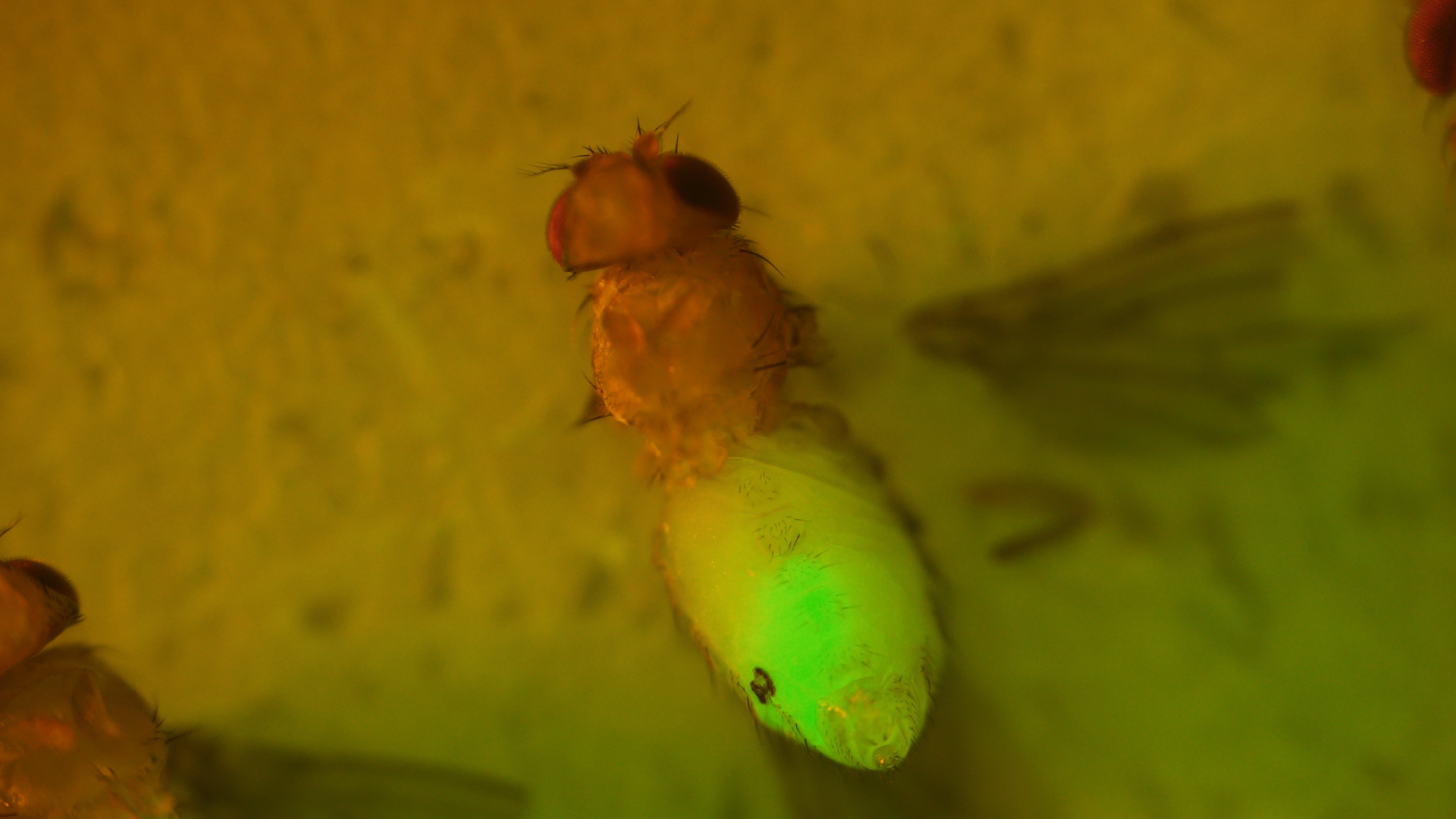

Supplement: Supplementary file 10 — Source data Fig. 6 [file 44318_2025_547_MOESM10_ESM.zip › Figure 6G/5-2 original image.tif]

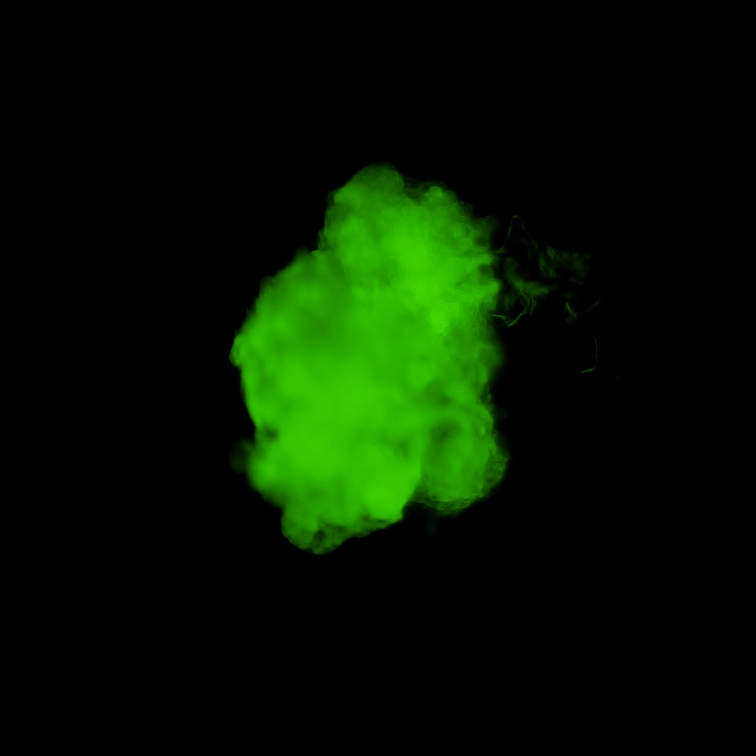

Supplement: Supplementary file 10 — Source data Fig. 6 [file 44318_2025_547_MOESM10_ESM.zip › Figure 6G/6-1 rotated and cut image with border line.tif]

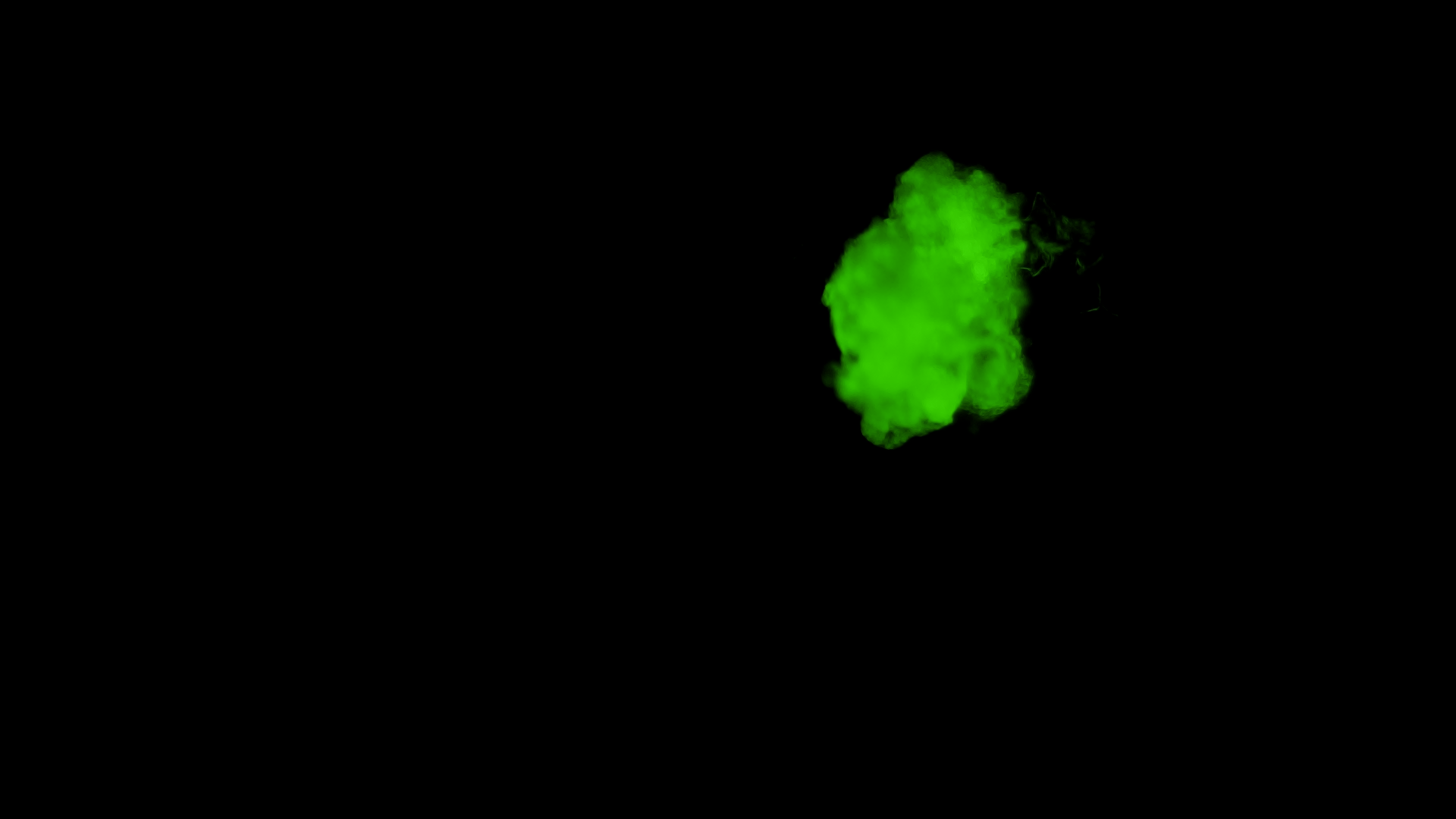

Supplement: Supplementary file 10 — Source data Fig. 6 [file 44318_2025_547_MOESM10_ESM.zip › Figure 6G/6-2 original image.tif]

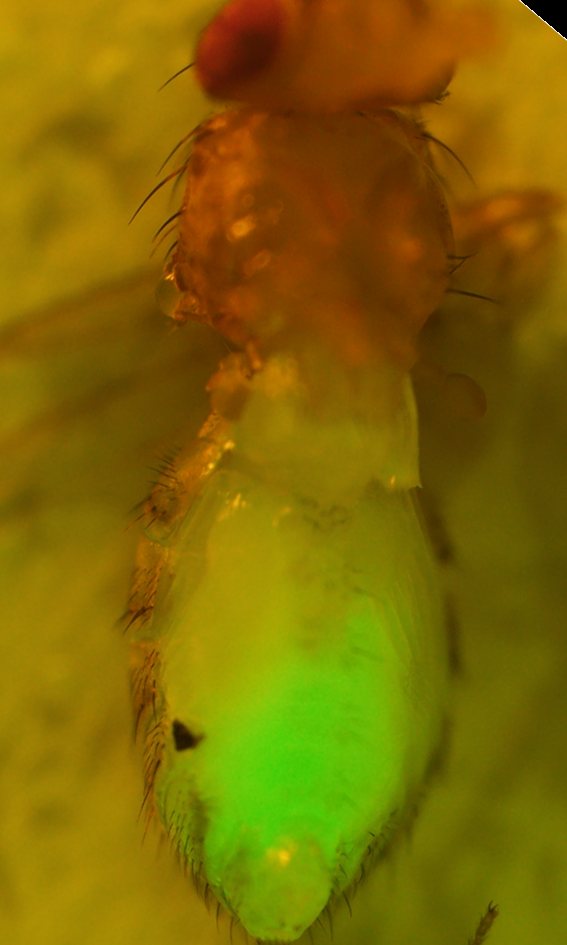

Supplement: Supplementary file 10 — Source data Fig. 6 [file 44318_2025_547_MOESM10_ESM.zip › Figure 6G/7-1 rotated and cut image.tif]

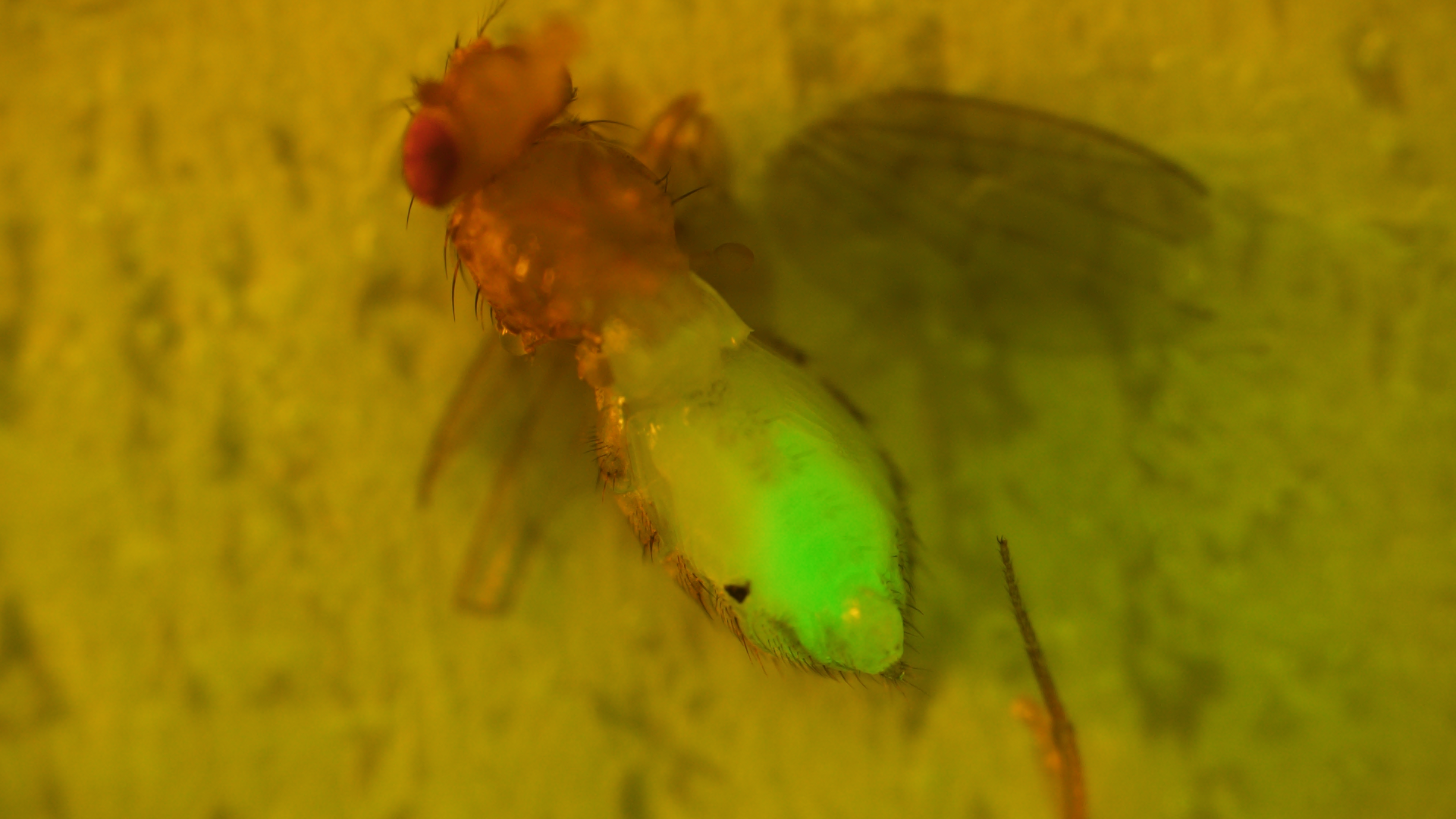

Supplement: Supplementary file 10 — Source data Fig. 6 [file 44318_2025_547_MOESM10_ESM.zip › Figure 6G/7-2 original image.tif]

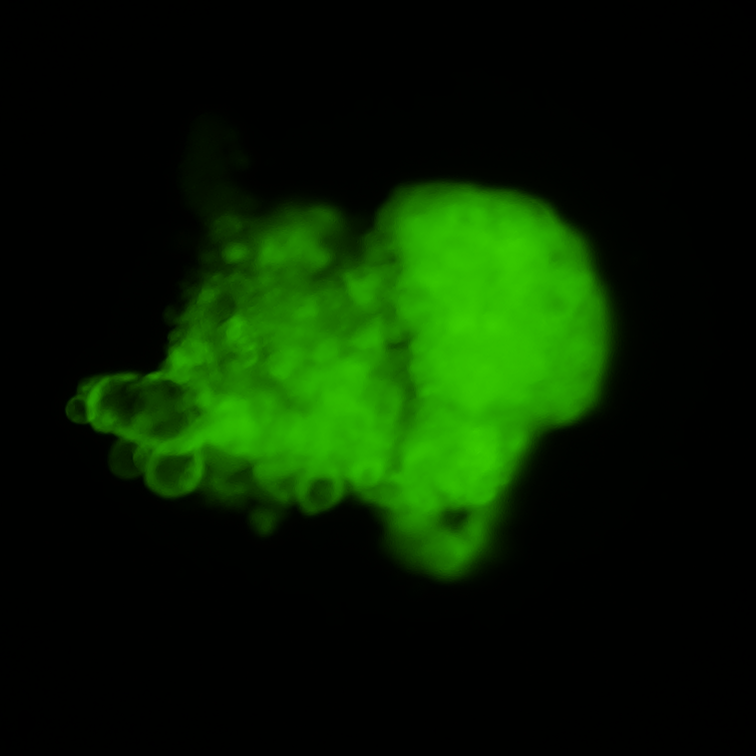

Supplement: Supplementary file 10 — Source data Fig. 6 [file 44318_2025_547_MOESM10_ESM.zip › Figure 6G/8-1 rotated and cut image.tif]

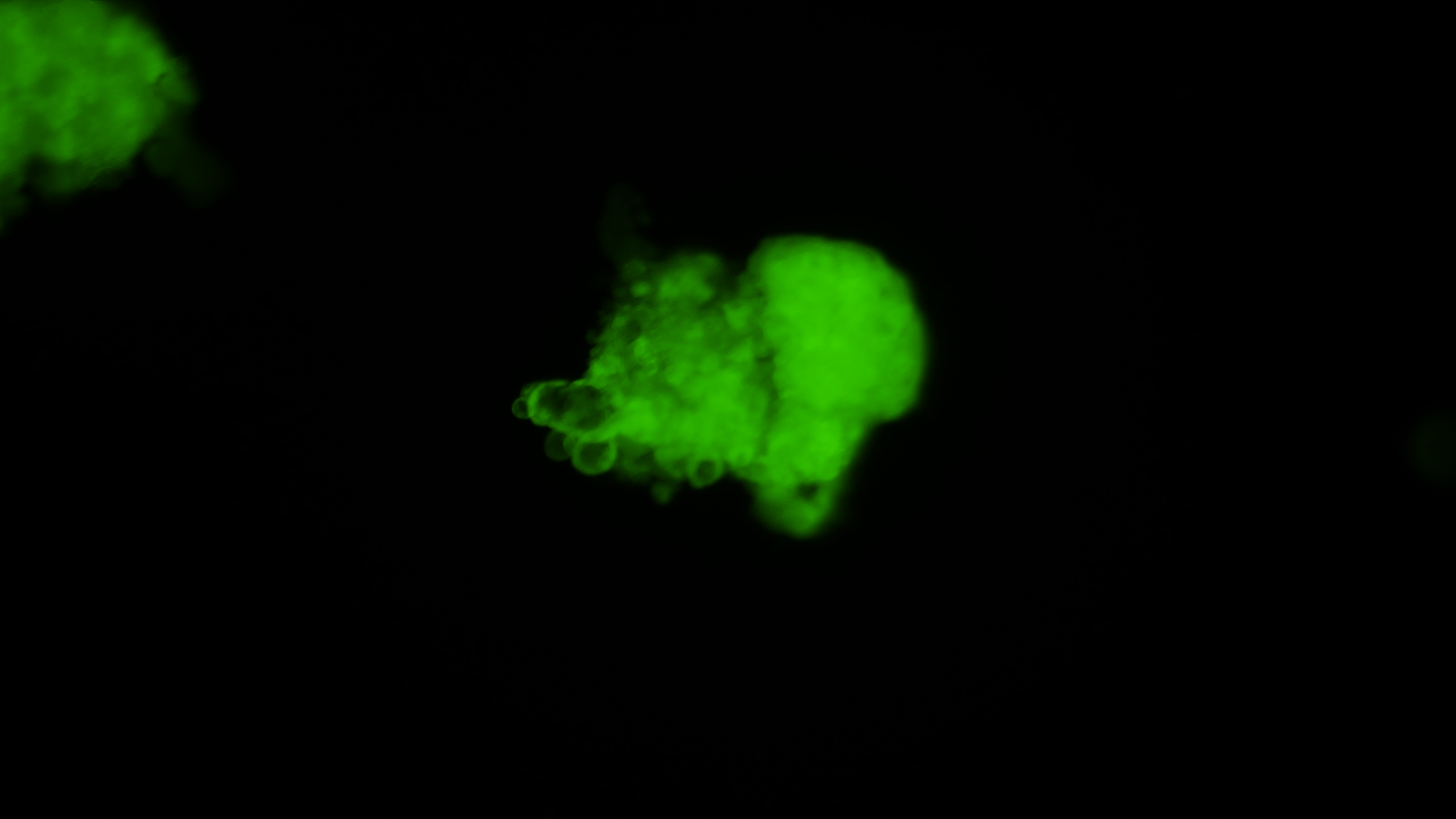

Supplement: Supplementary file 10 — Source data Fig. 6 [file 44318_2025_547_MOESM10_ESM.zip › Figure 6G/8-2 original image.tif]
