# Supplementary figures and images for: Hemocytes facilitate interclonal cooperation-induced tumor malignancy by hijacking the innate immune system in Drosophila (part 4 of 4)
Source: EMBO J. 2025 Aug 22;44(19):5394–428. doi: 10.1038/s44318-025-00547-5 (PMC12489090; doi:10.1038/s44318-025-00547-5)

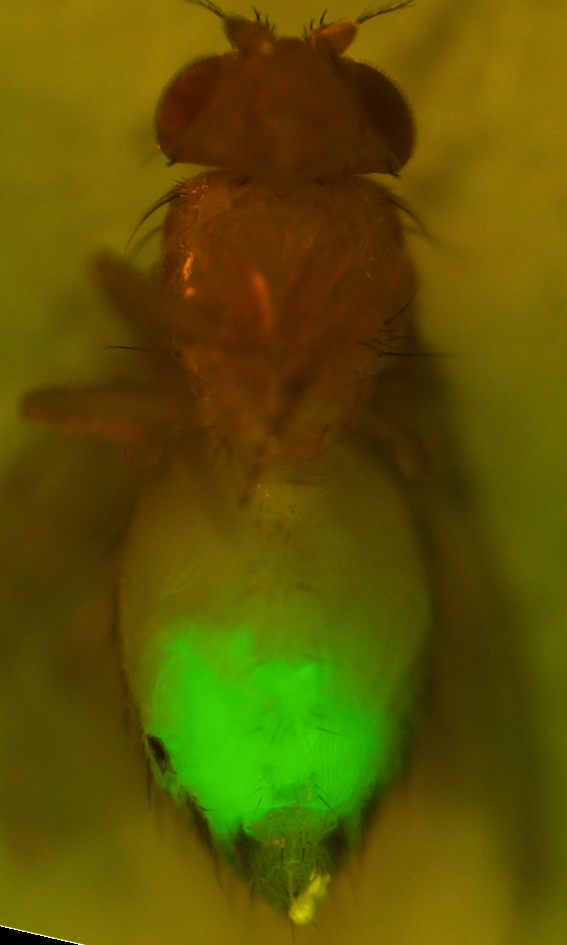

Supplement: Supplementary file 10 — Source data Fig. 6 [file 44318_2025_547_MOESM10_ESM.zip › Figure 6G/9-1 rotated and cut image.tif]

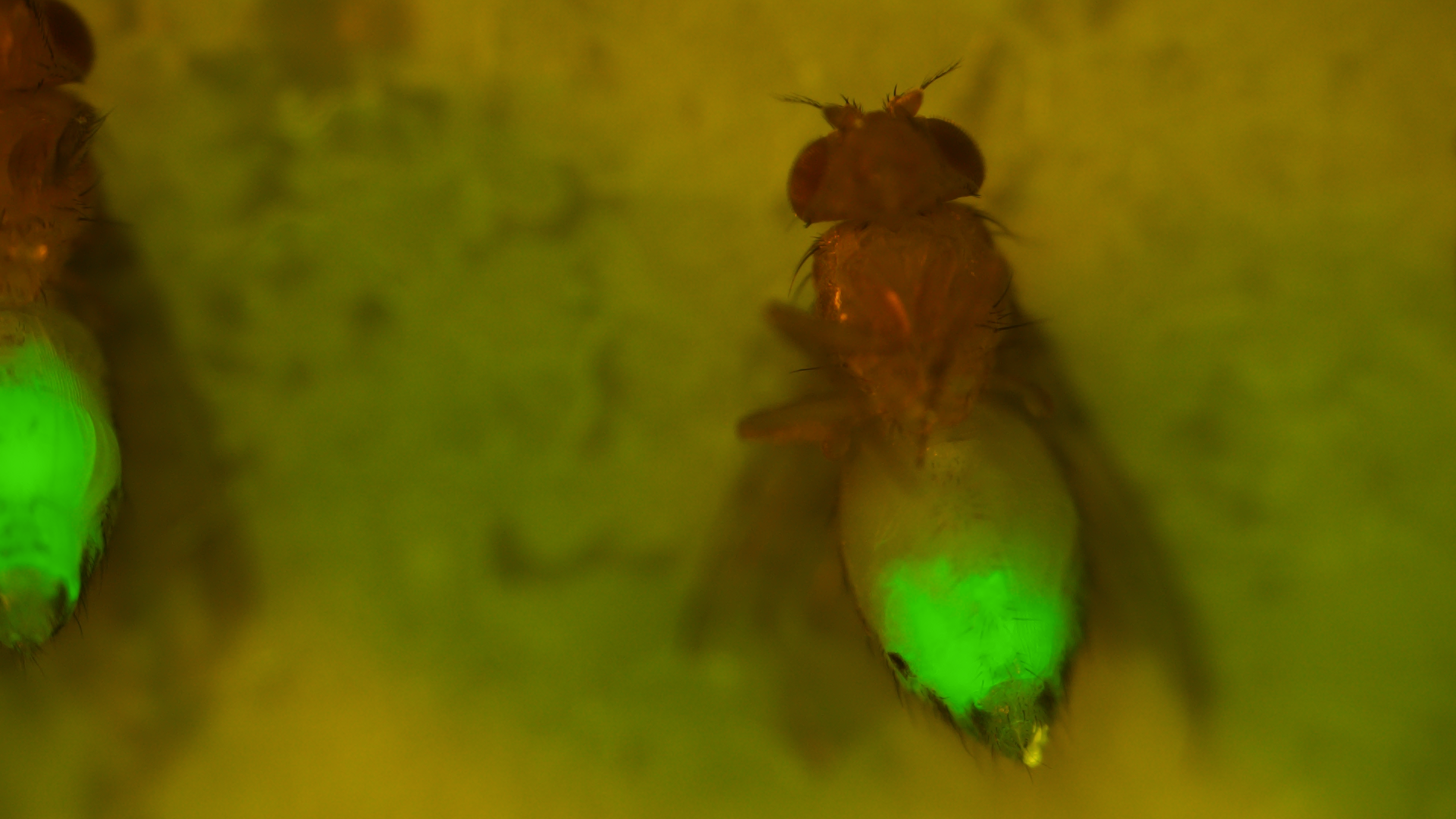

Supplement: Supplementary file 10 — Source data Fig. 6 [file 44318_2025_547_MOESM10_ESM.zip › Figure 6G/9-2 original image.tif]

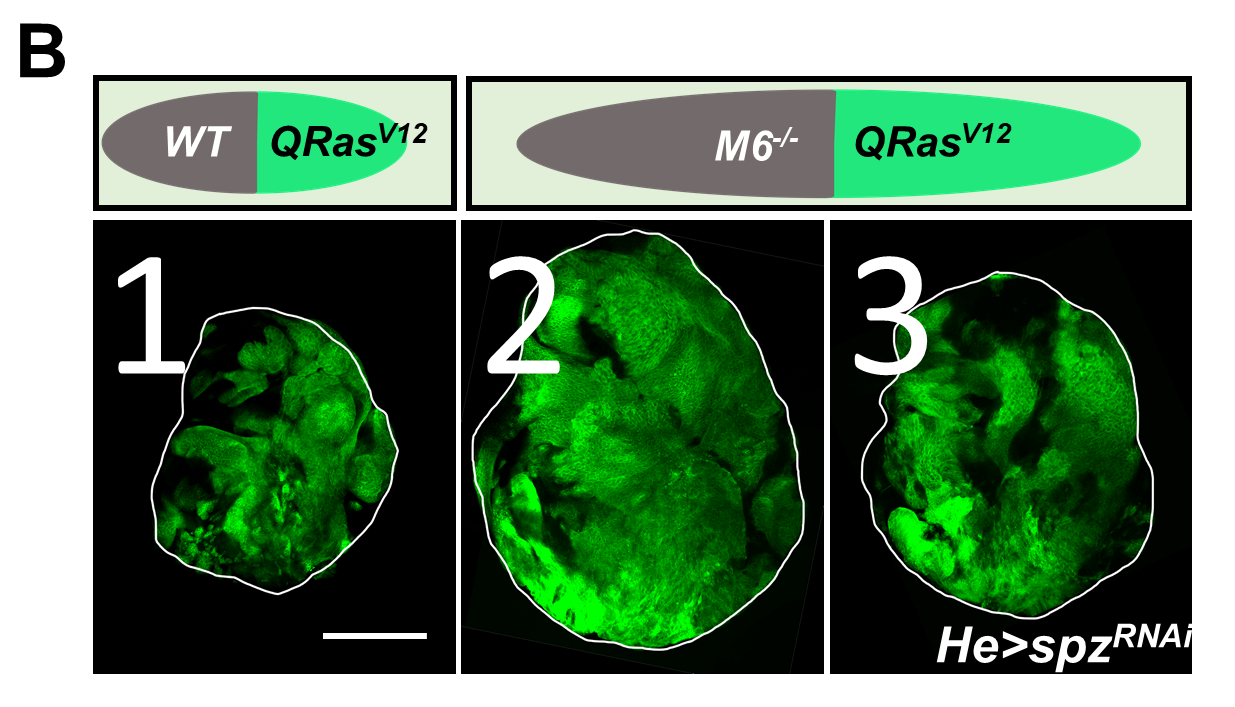

Supplement: Supplementary file 10 — Source data Fig. 6 [file 44318_2025_547_MOESM10_ESM.zip › Figure 6B/0 paper Figure 6B with provided image sequence.tif]

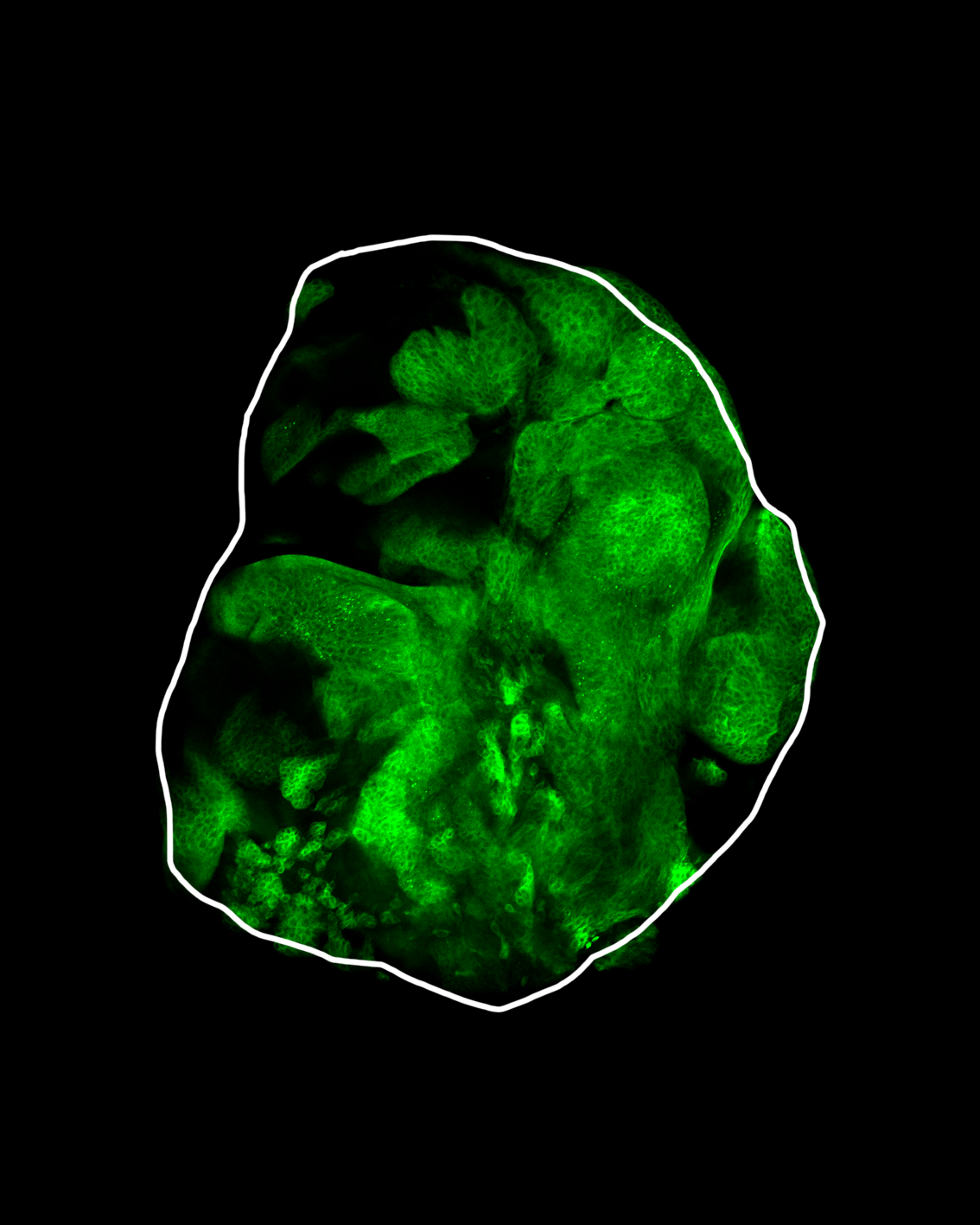

Supplement: Supplementary file 10 — Source data Fig. 6 [file 44318_2025_547_MOESM10_ESM.zip › Figure 6B/1-1 rotated and cut image with border line.tif]

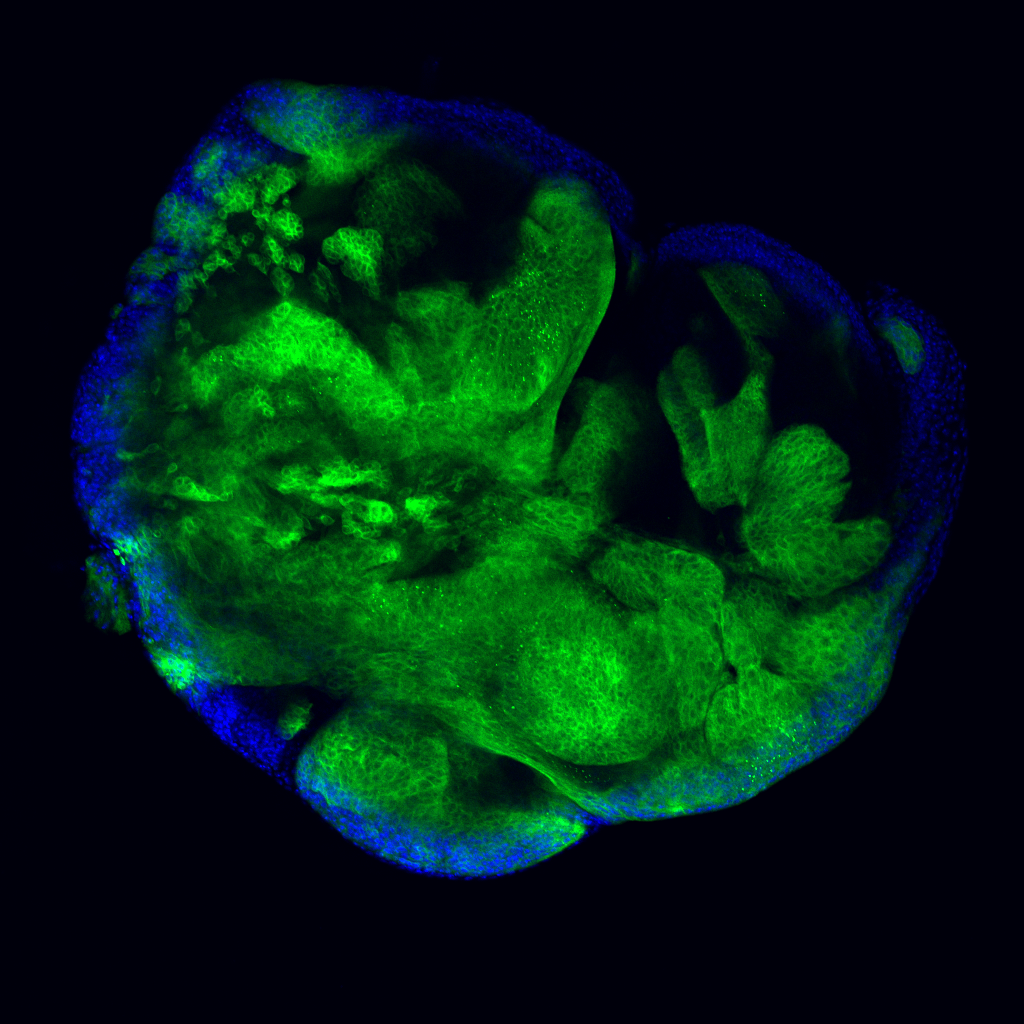

Supplement: Supplementary file 10 — Source data Fig. 6 [file 44318_2025_547_MOESM10_ESM.zip › Figure 6B/1-2 original image.tif]

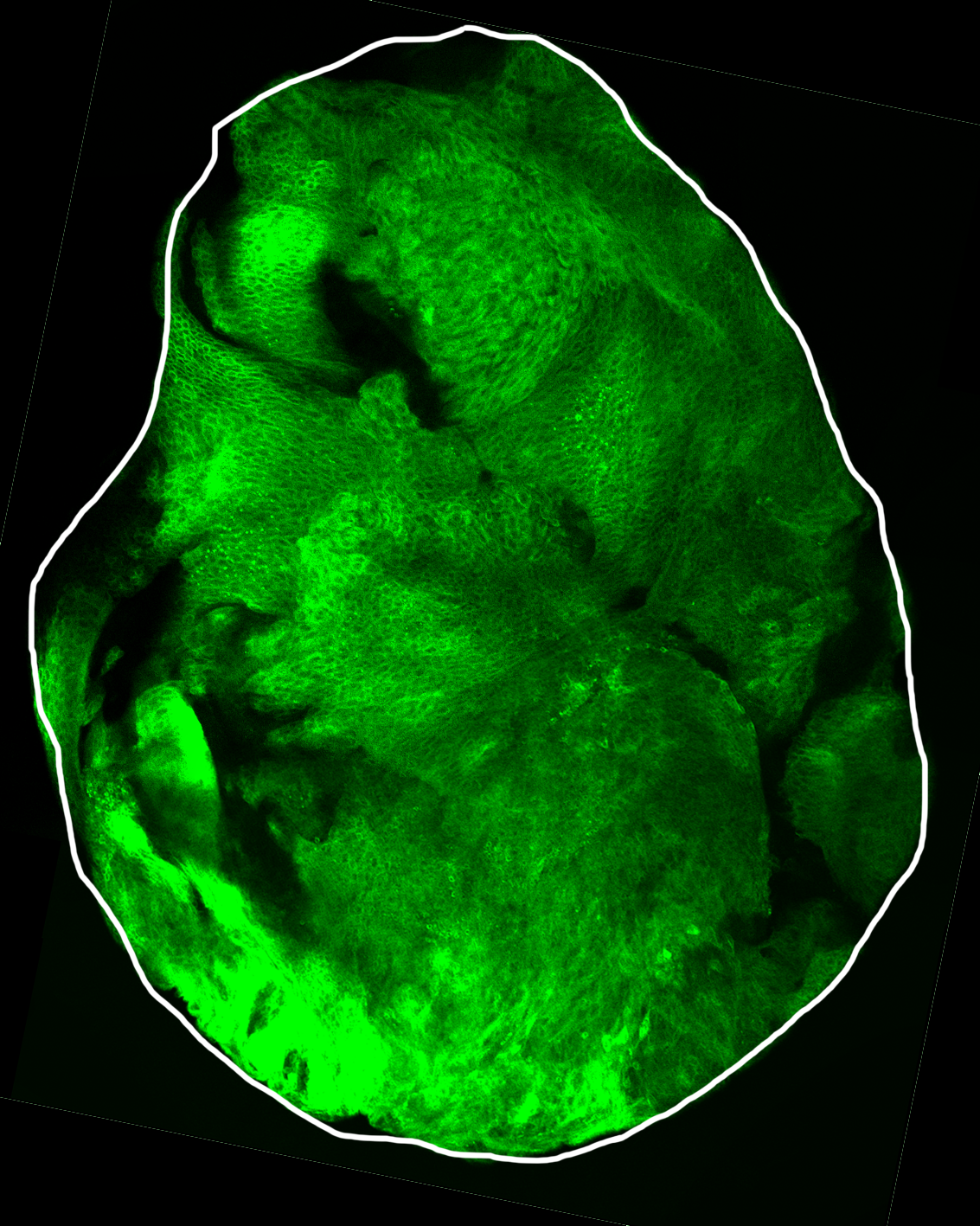

Supplement: Supplementary file 10 — Source data Fig. 6 [file 44318_2025_547_MOESM10_ESM.zip › Figure 6B/2-1 rotated and cut image with border line.tif]

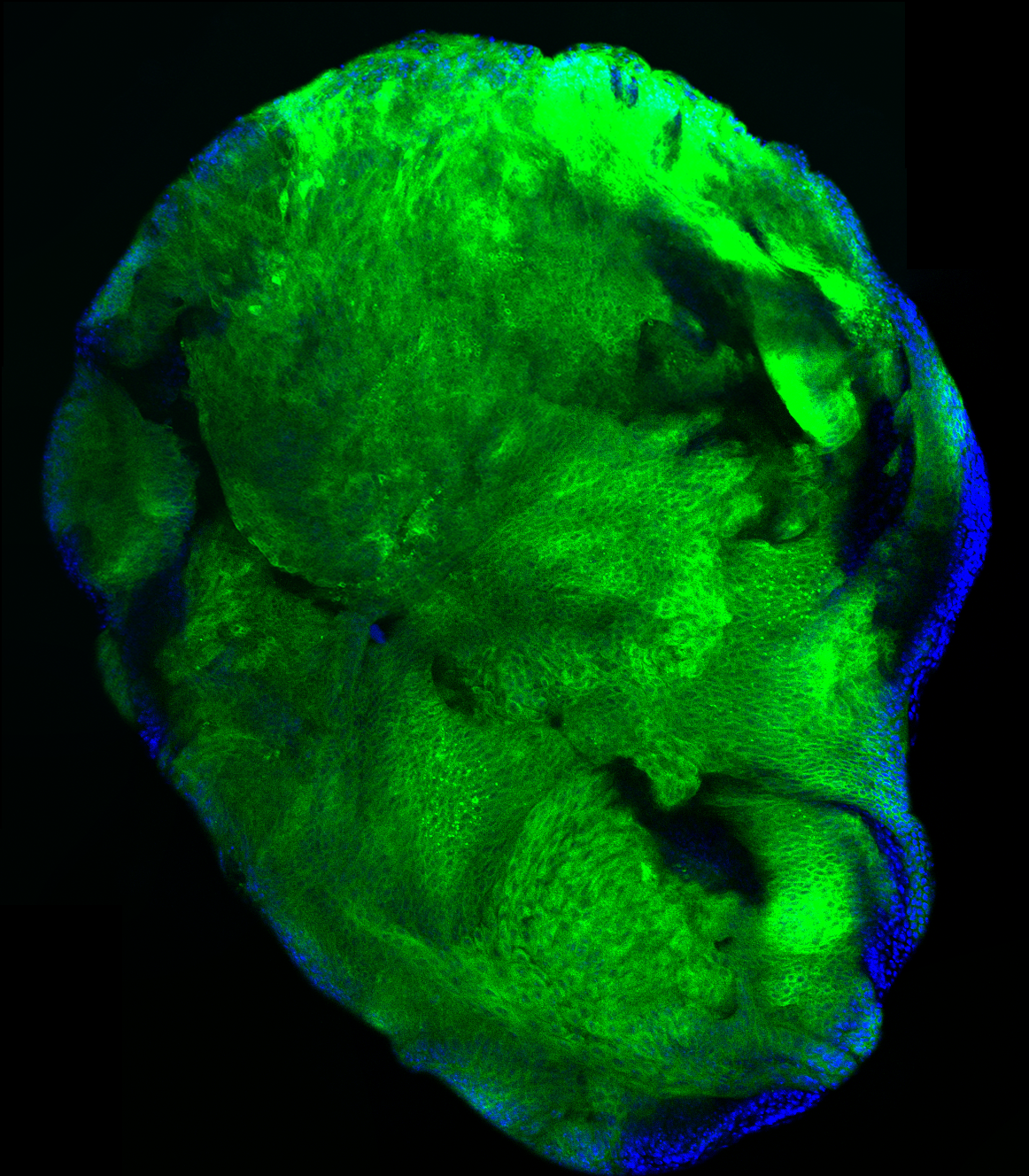

Supplement: Supplementary file 10 — Source data Fig. 6 [file 44318_2025_547_MOESM10_ESM.zip › Figure 6B/2-2 original image.tif]

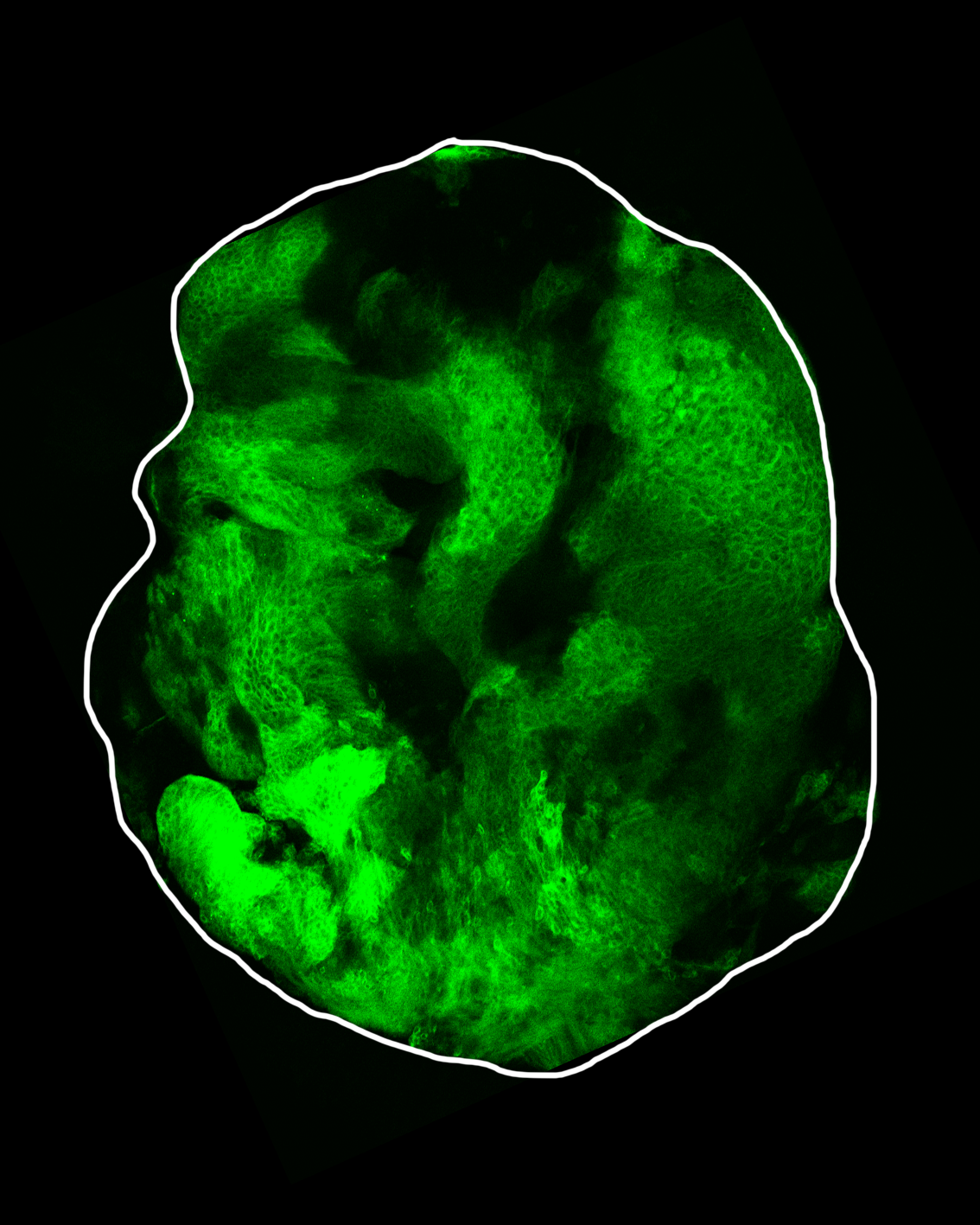

Supplement: Supplementary file 10 — Source data Fig. 6 [file 44318_2025_547_MOESM10_ESM.zip › Figure 6B/3-1 rotated and cut image with border line.tif]

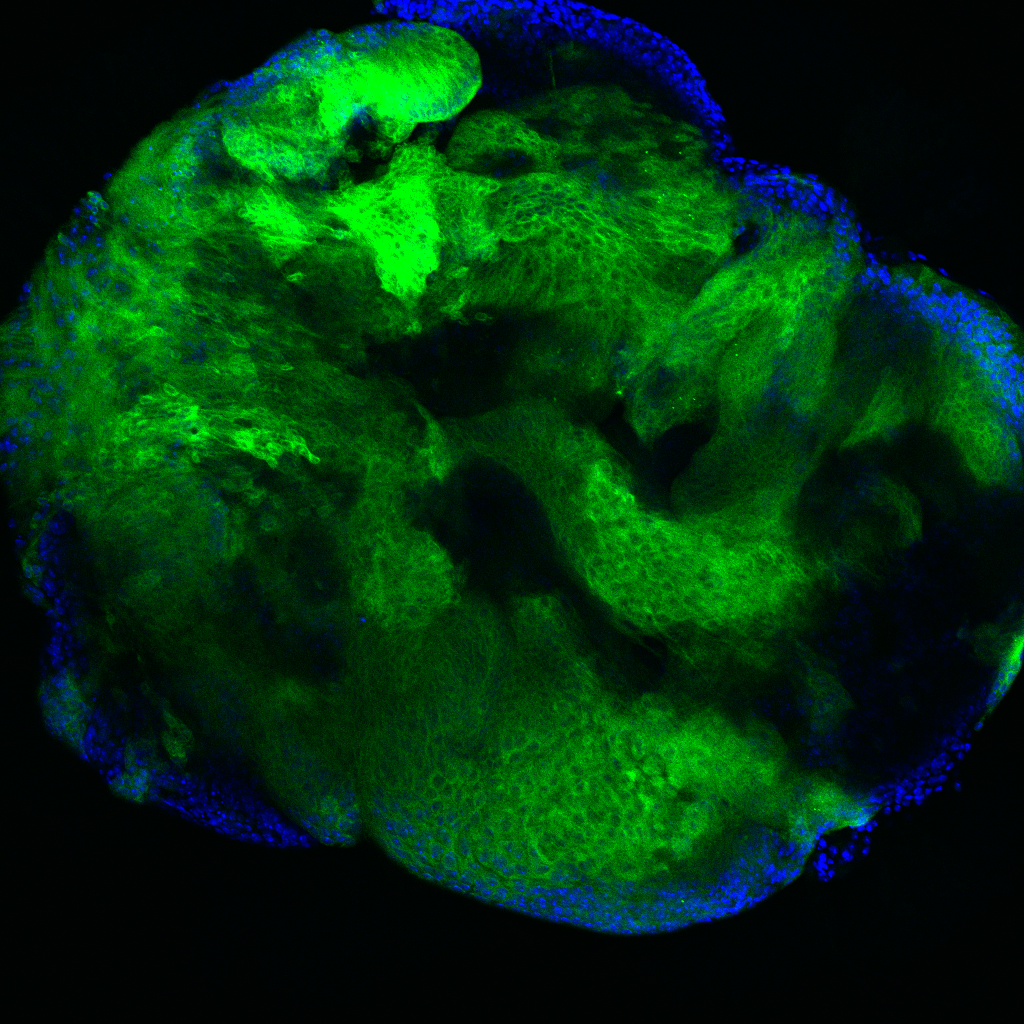

Supplement: Supplementary file 10 — Source data Fig. 6 [file 44318_2025_547_MOESM10_ESM.zip › Figure 6B/3-2 original image.tif]

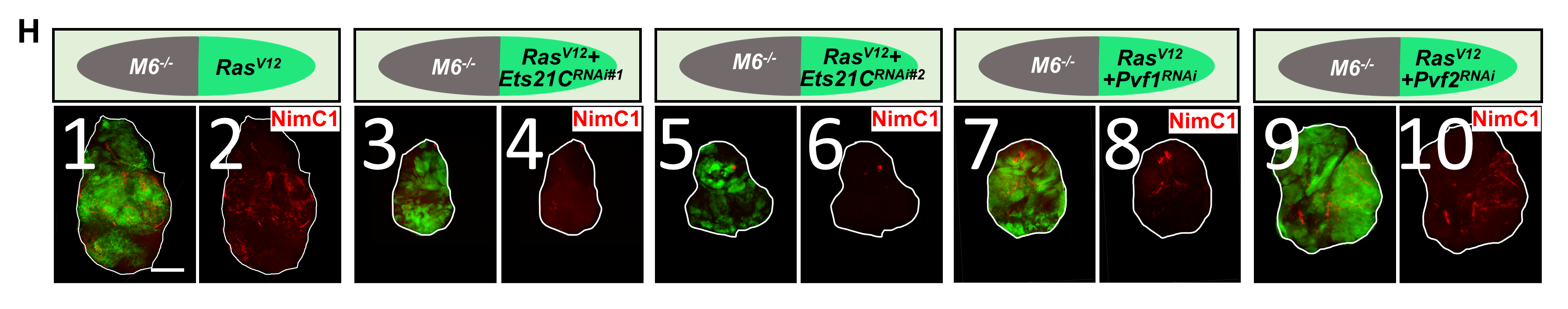

Supplement: Supplementary file 11 — Source data Fig. 7 [file 44318_2025_547_MOESM11_ESM.zip › Figure 7H/0 paper Figure 7H with provided image sequence.tif]

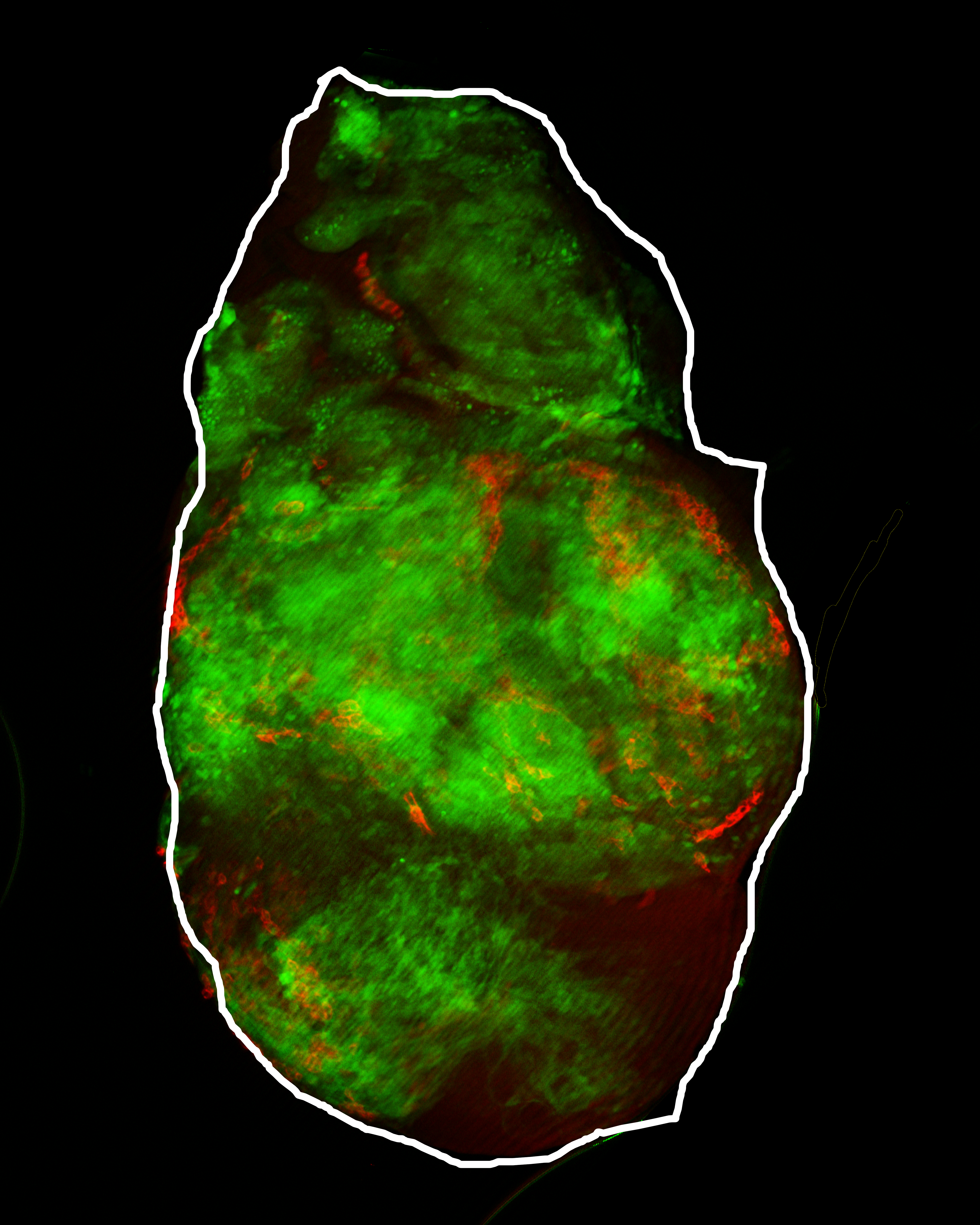

Supplement: Supplementary file 11 — Source data Fig. 7 [file 44318_2025_547_MOESM11_ESM.zip › Figure 7H/1-1 rotated and cut image with border line.tif]

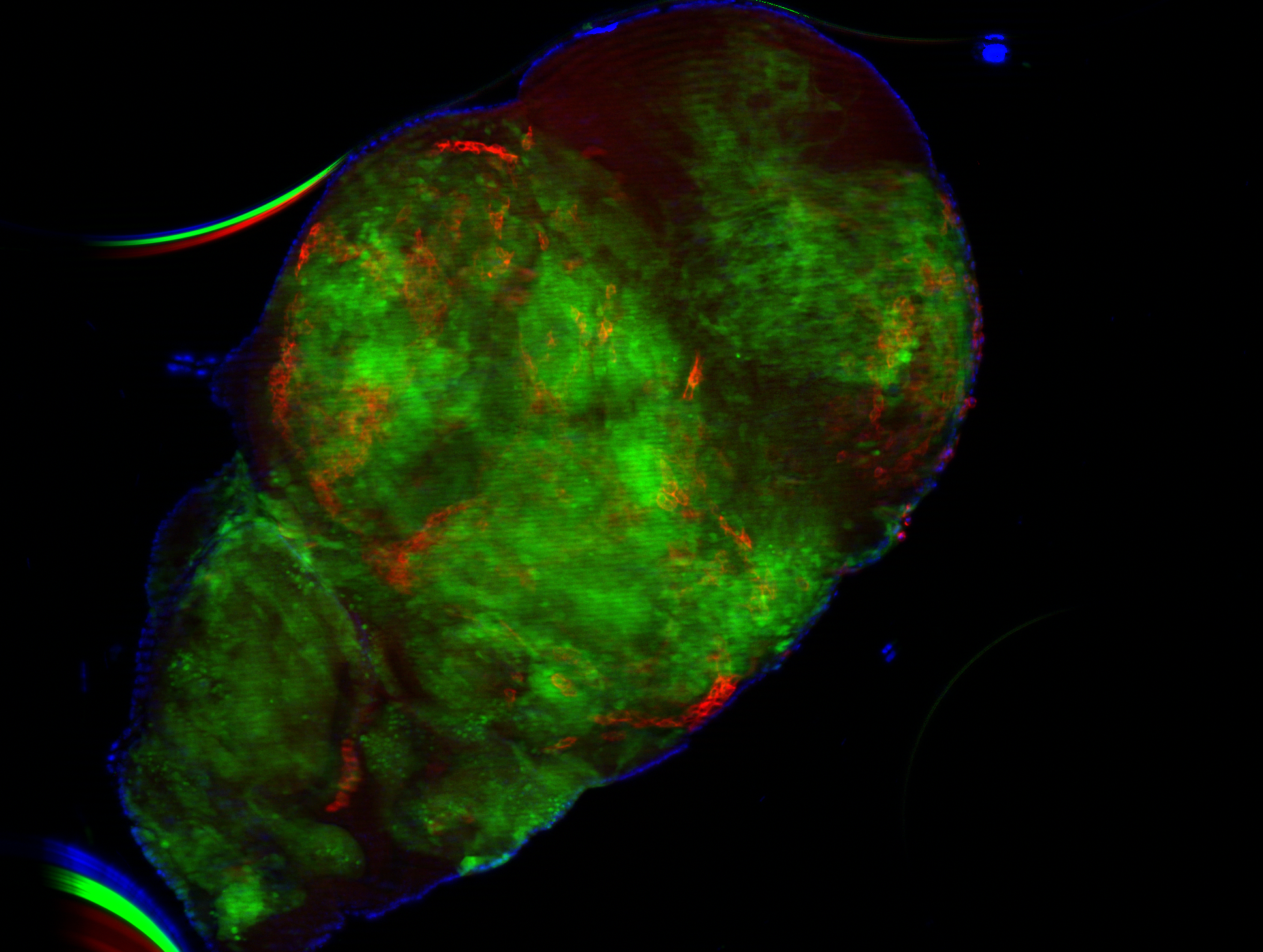

Supplement: Supplementary file 11 — Source data Fig. 7 [file 44318_2025_547_MOESM11_ESM.zip › Figure 7H/1-2 original image.tif]

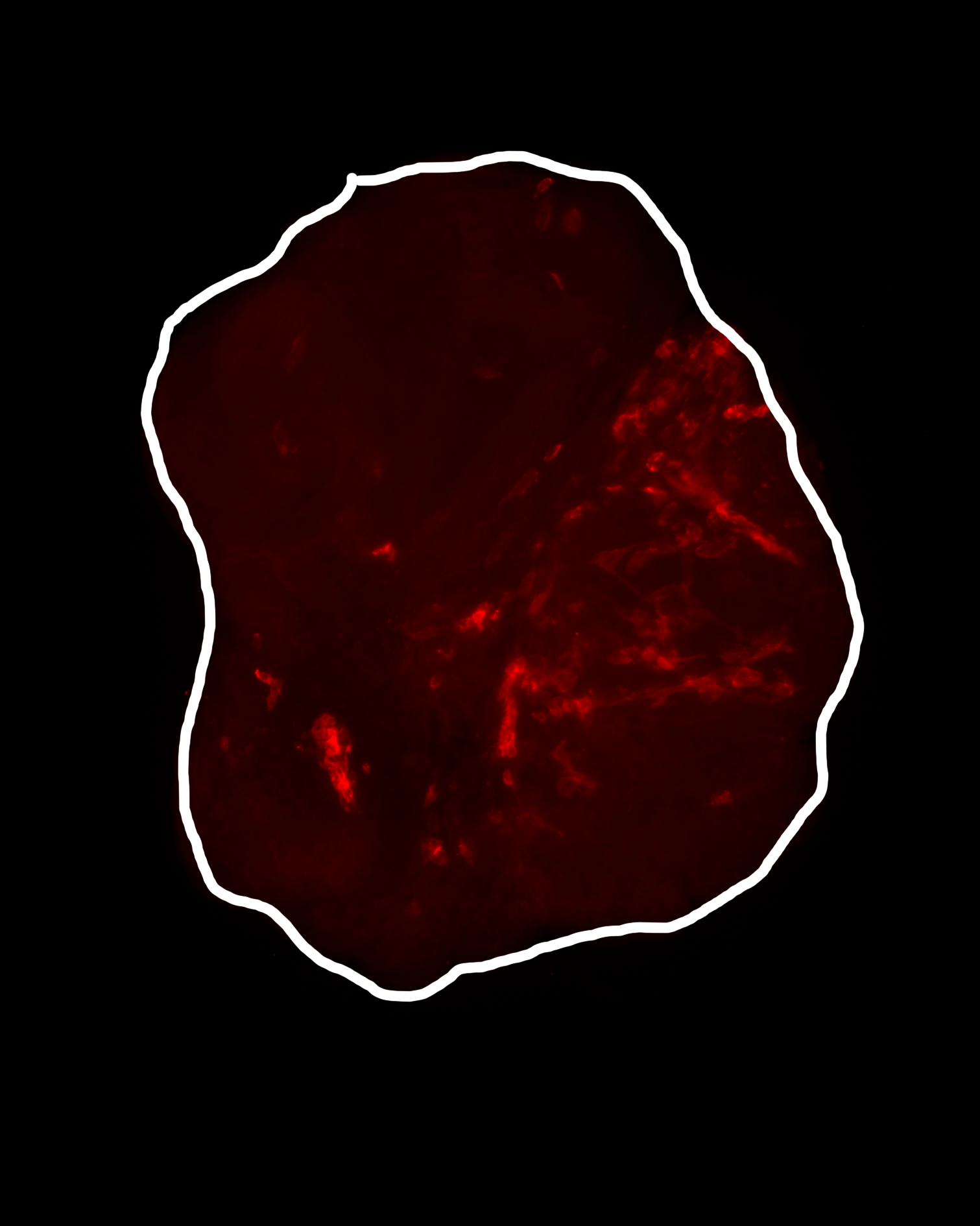

Supplement: Supplementary file 11 — Source data Fig. 7 [file 44318_2025_547_MOESM11_ESM.zip › Figure 7H/10-1 rotated and cut image with border line.tif]

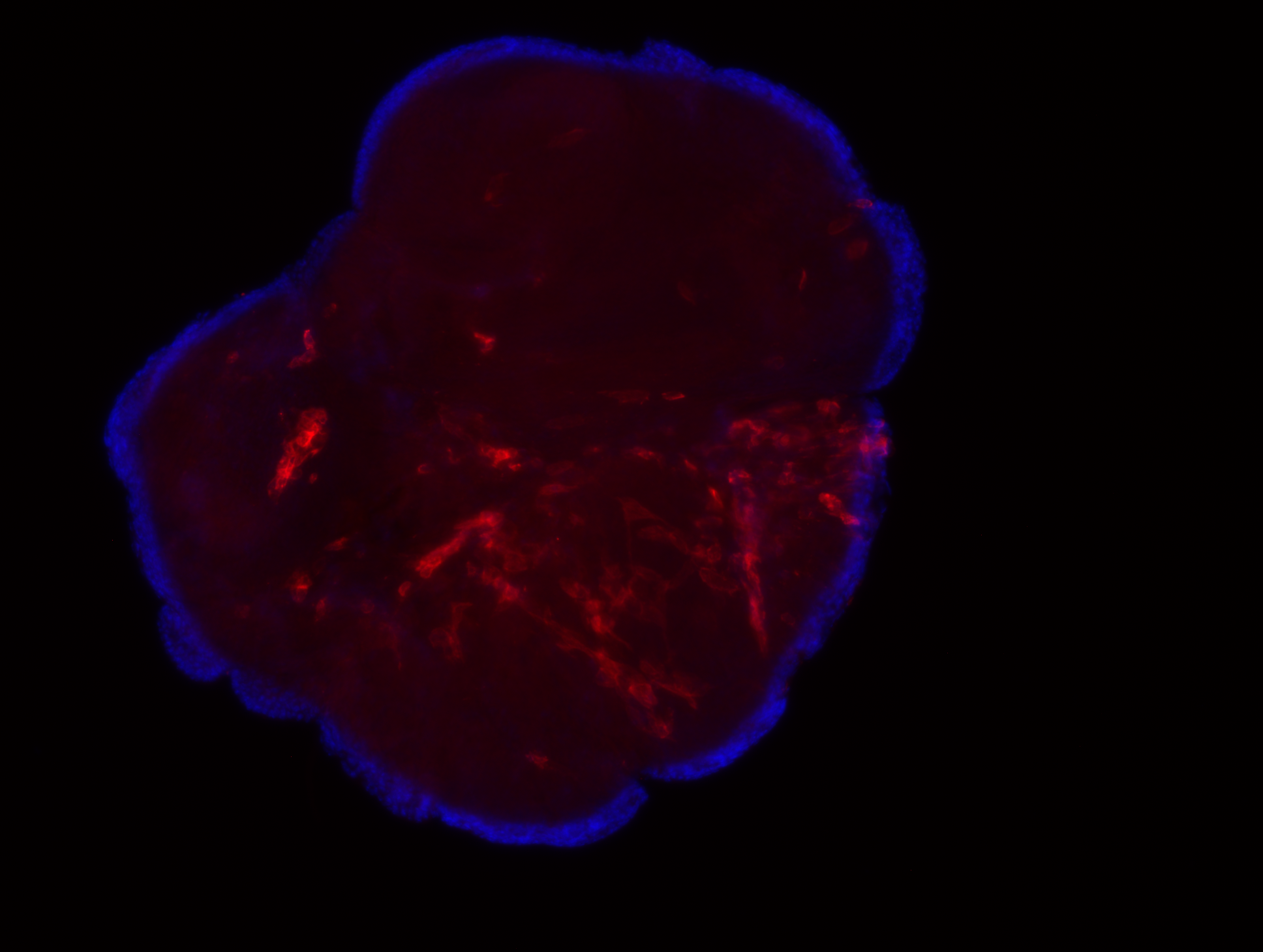

Supplement: Supplementary file 11 — Source data Fig. 7 [file 44318_2025_547_MOESM11_ESM.zip › Figure 7H/10-2 original image.tif]

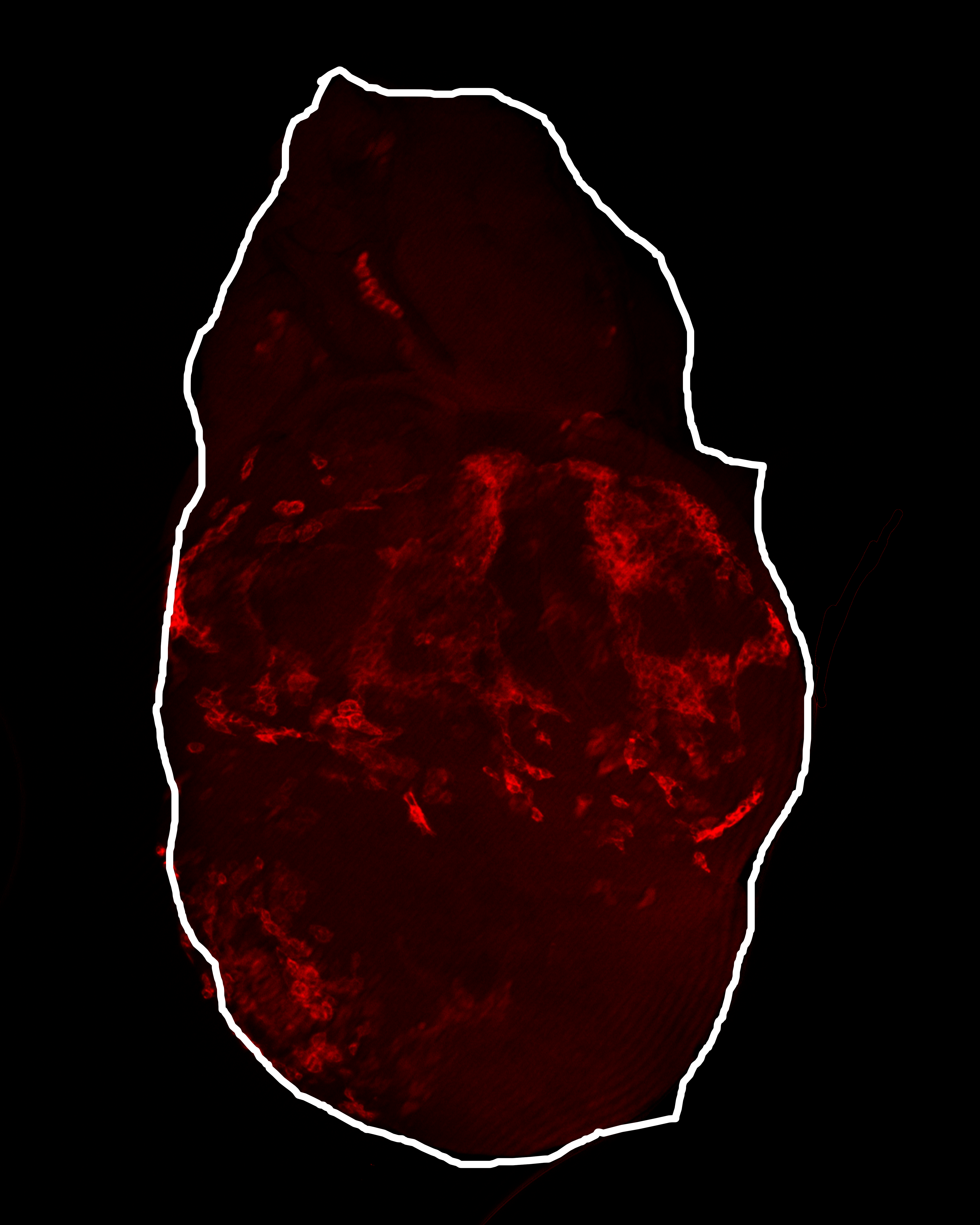

Supplement: Supplementary file 11 — Source data Fig. 7 [file 44318_2025_547_MOESM11_ESM.zip › Figure 7H/2-1 rotated and cut image with border line.tif]

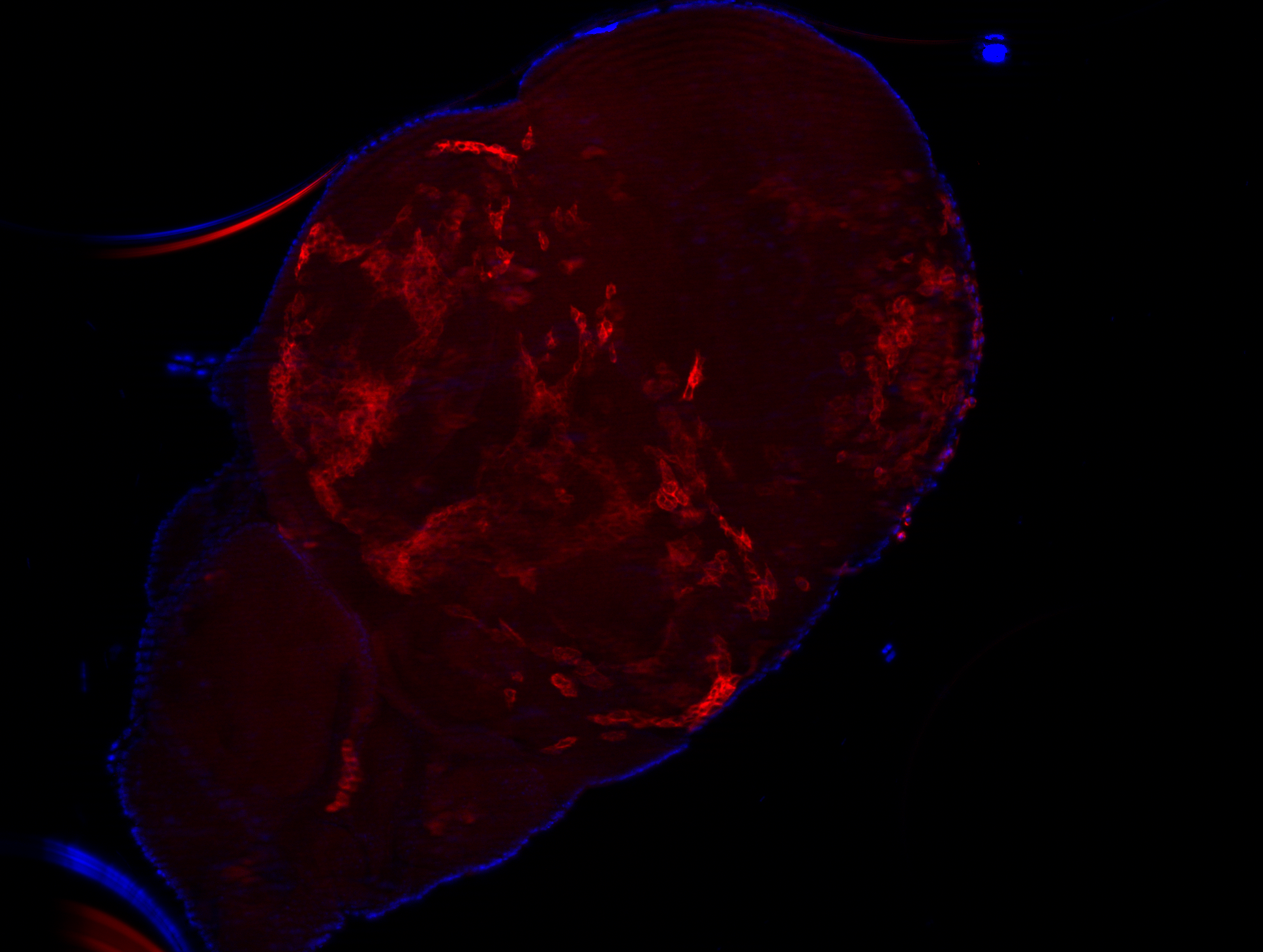

Supplement: Supplementary file 11 — Source data Fig. 7 [file 44318_2025_547_MOESM11_ESM.zip › Figure 7H/2-2 original image.tif]

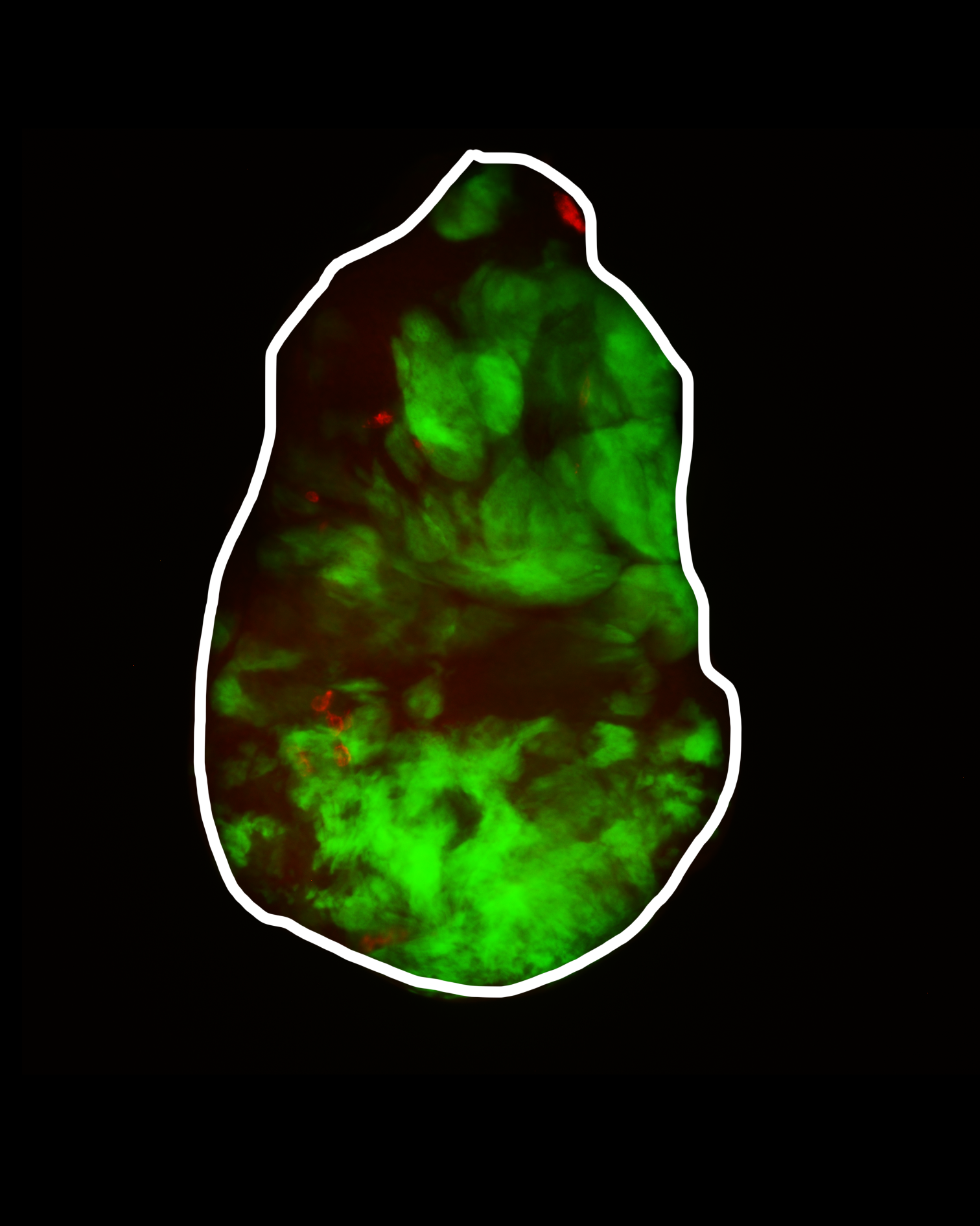

Supplement: Supplementary file 11 — Source data Fig. 7 [file 44318_2025_547_MOESM11_ESM.zip › Figure 7H/3-1 rotated and cut image with border line.tif]

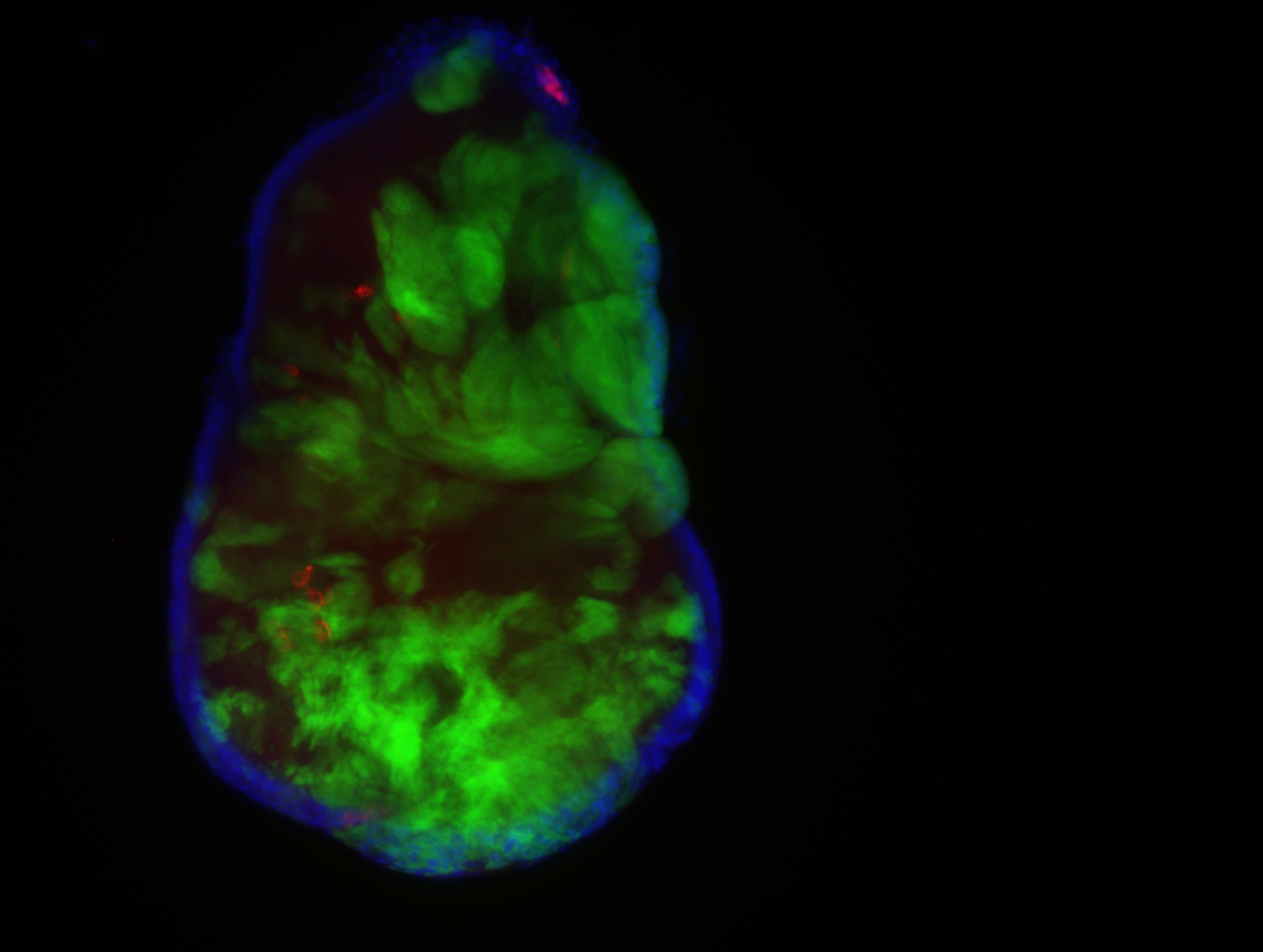

Supplement: Supplementary file 11 — Source data Fig. 7 [file 44318_2025_547_MOESM11_ESM.zip › Figure 7H/3-2 original image.tif]

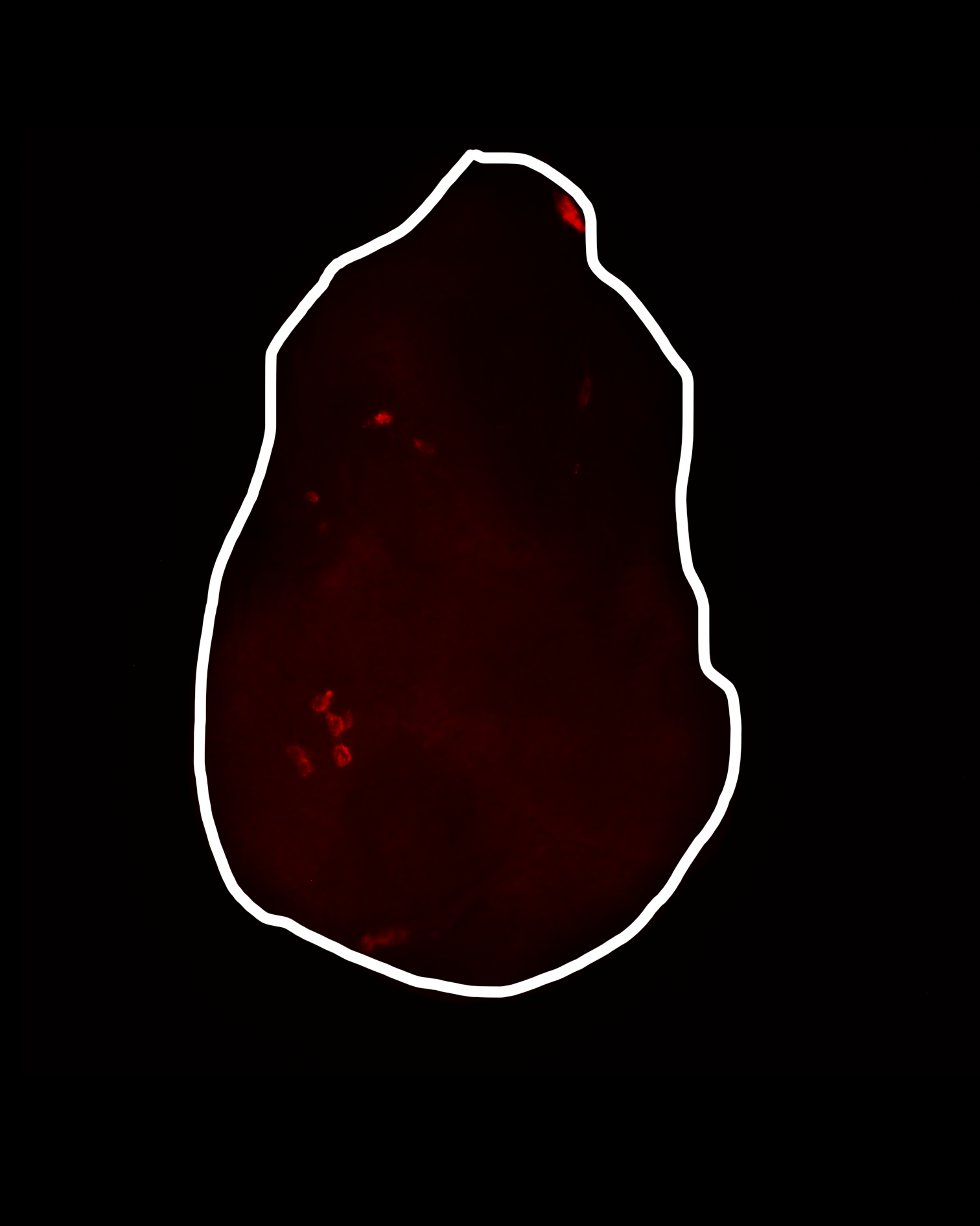

Supplement: Supplementary file 11 — Source data Fig. 7 [file 44318_2025_547_MOESM11_ESM.zip › Figure 7H/4-1 rotated and cut image with border line.tif]

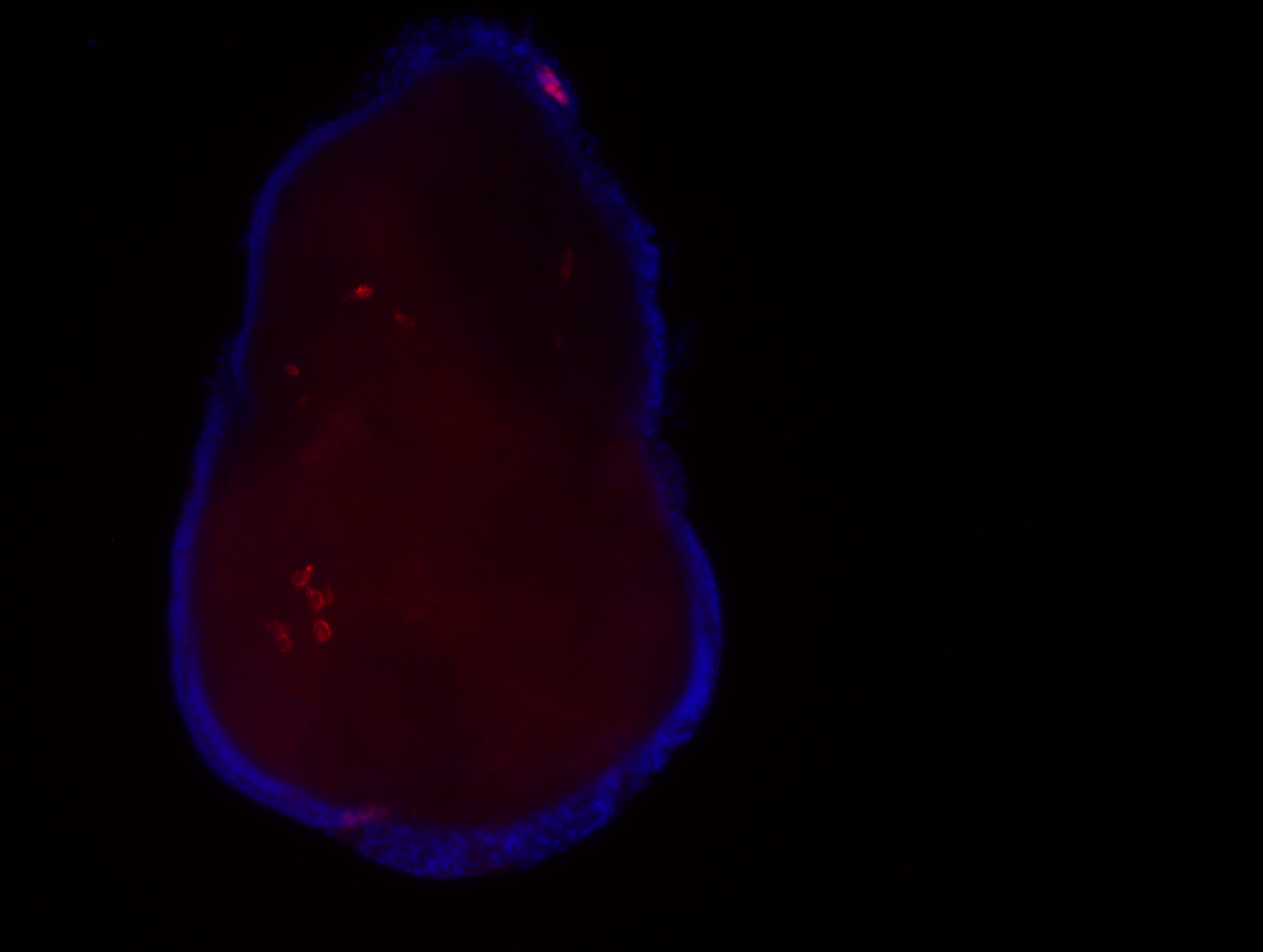

Supplement: Supplementary file 11 — Source data Fig. 7 [file 44318_2025_547_MOESM11_ESM.zip › Figure 7H/4-2 original image.tif]

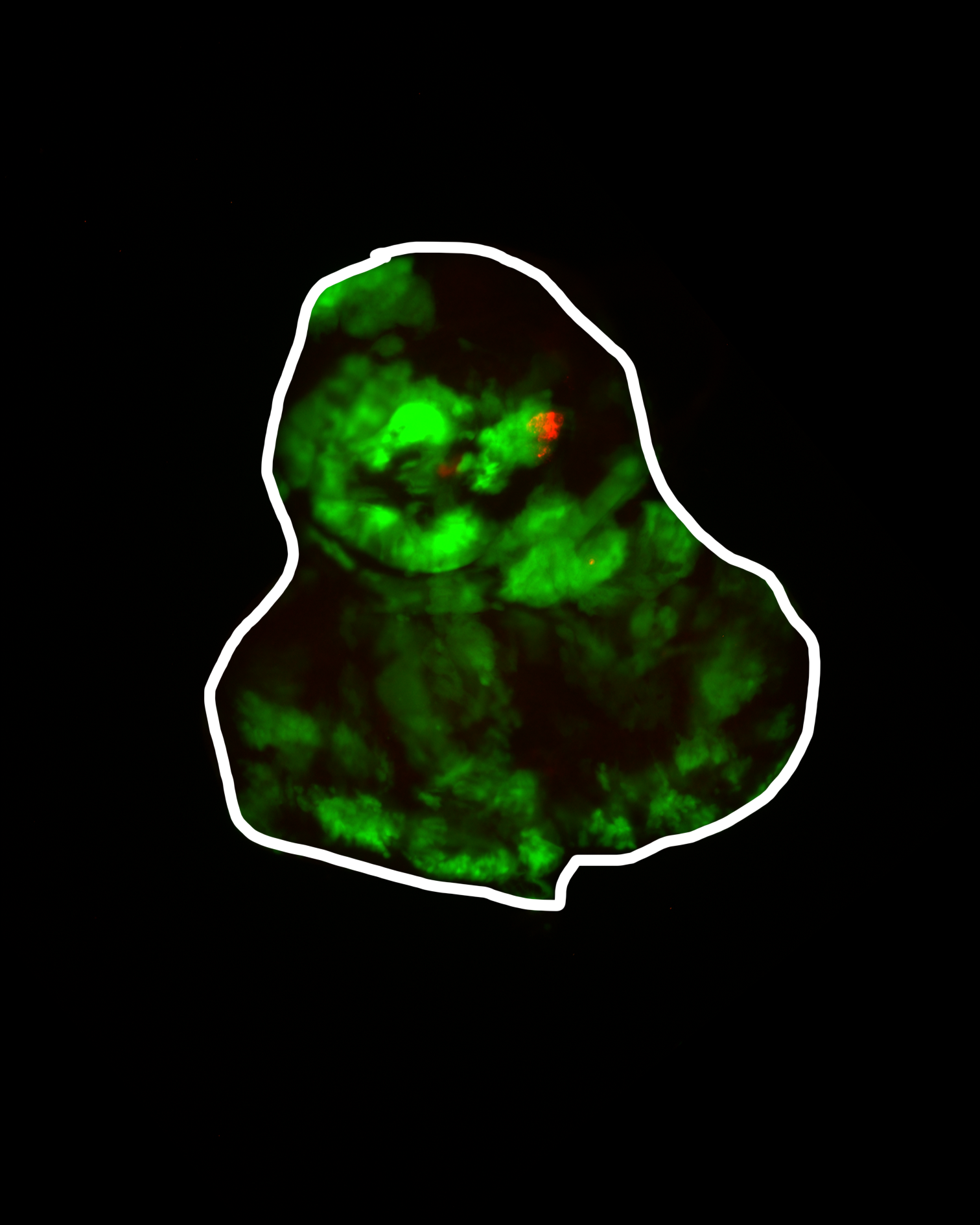

Supplement: Supplementary file 11 — Source data Fig. 7 [file 44318_2025_547_MOESM11_ESM.zip › Figure 7H/5-1 rotated and cut image with border line.tif]

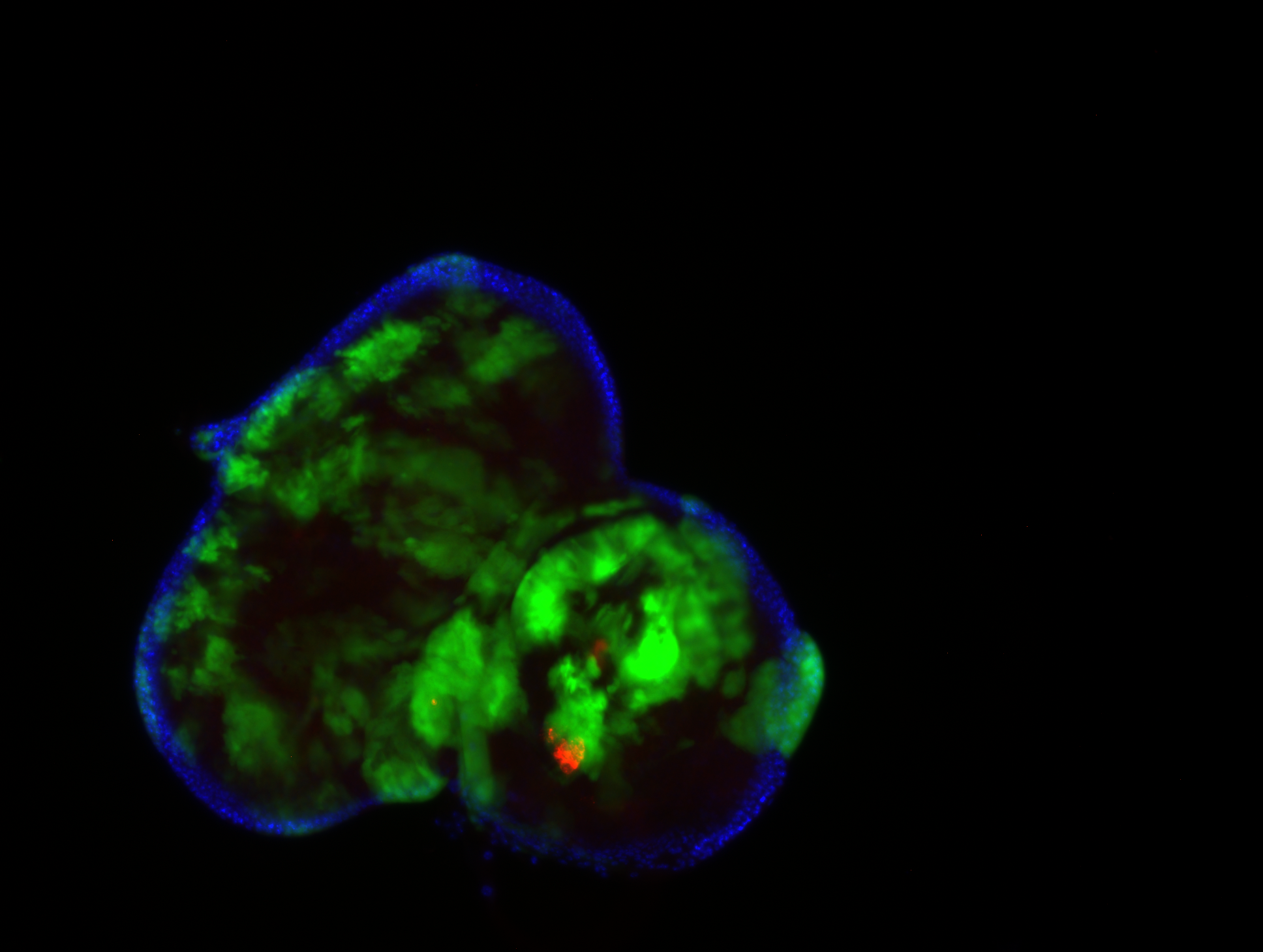

Supplement: Supplementary file 11 — Source data Fig. 7 [file 44318_2025_547_MOESM11_ESM.zip › Figure 7H/5-2 original image.tif]

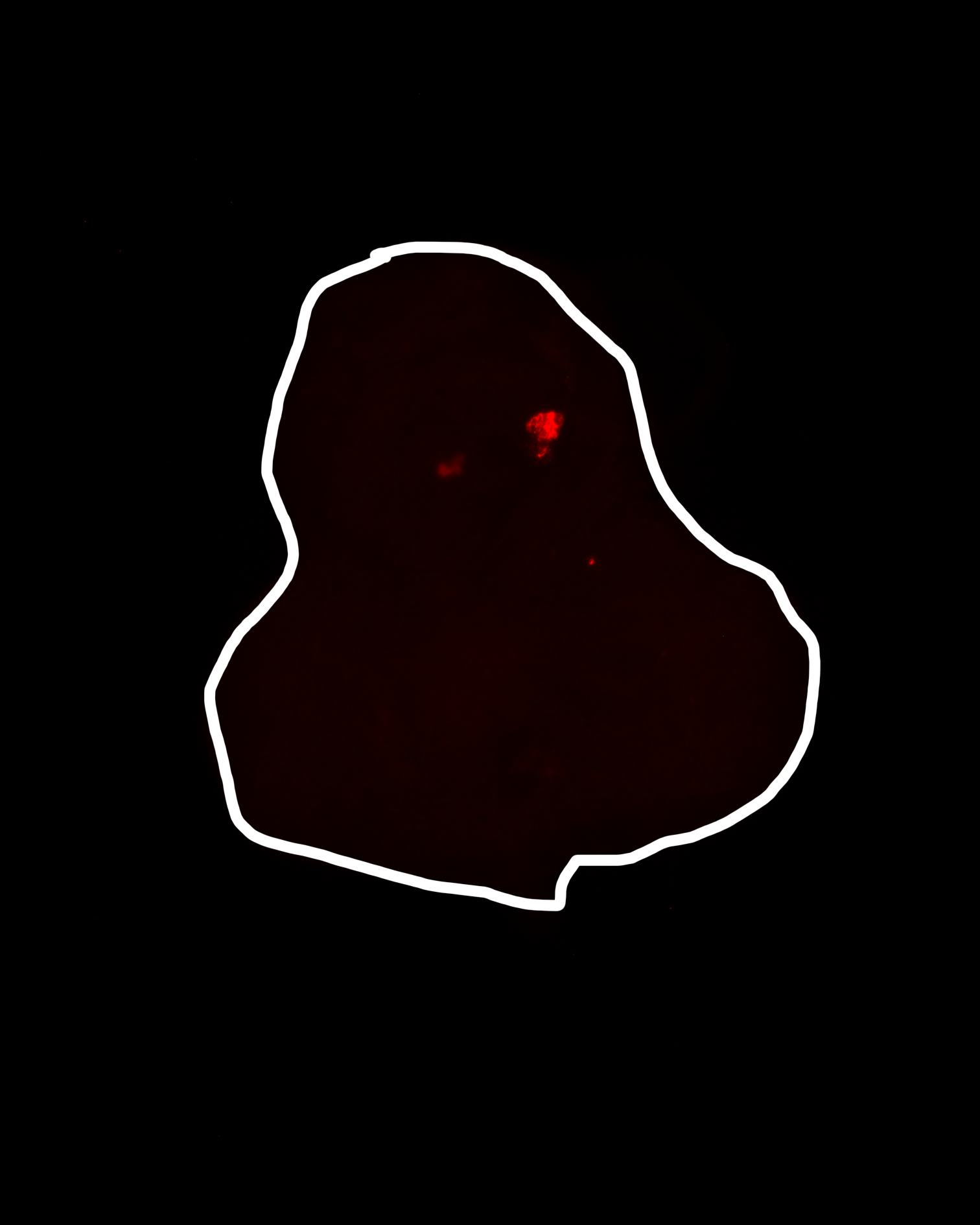

Supplement: Supplementary file 11 — Source data Fig. 7 [file 44318_2025_547_MOESM11_ESM.zip › Figure 7H/6-1 rotated and cut image with border line.tif]

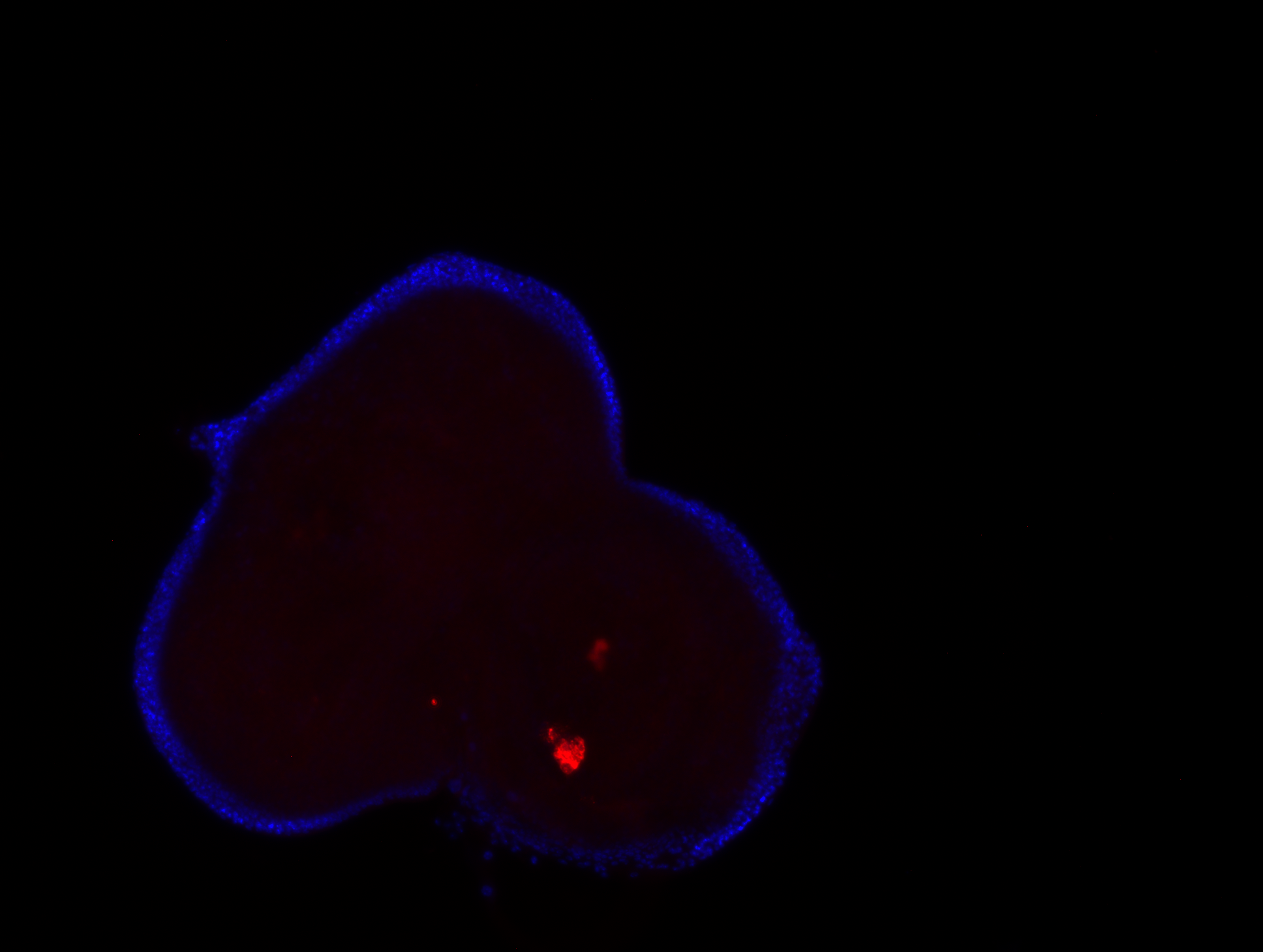

Supplement: Supplementary file 11 — Source data Fig. 7 [file 44318_2025_547_MOESM11_ESM.zip › Figure 7H/6-2 original image.tif]

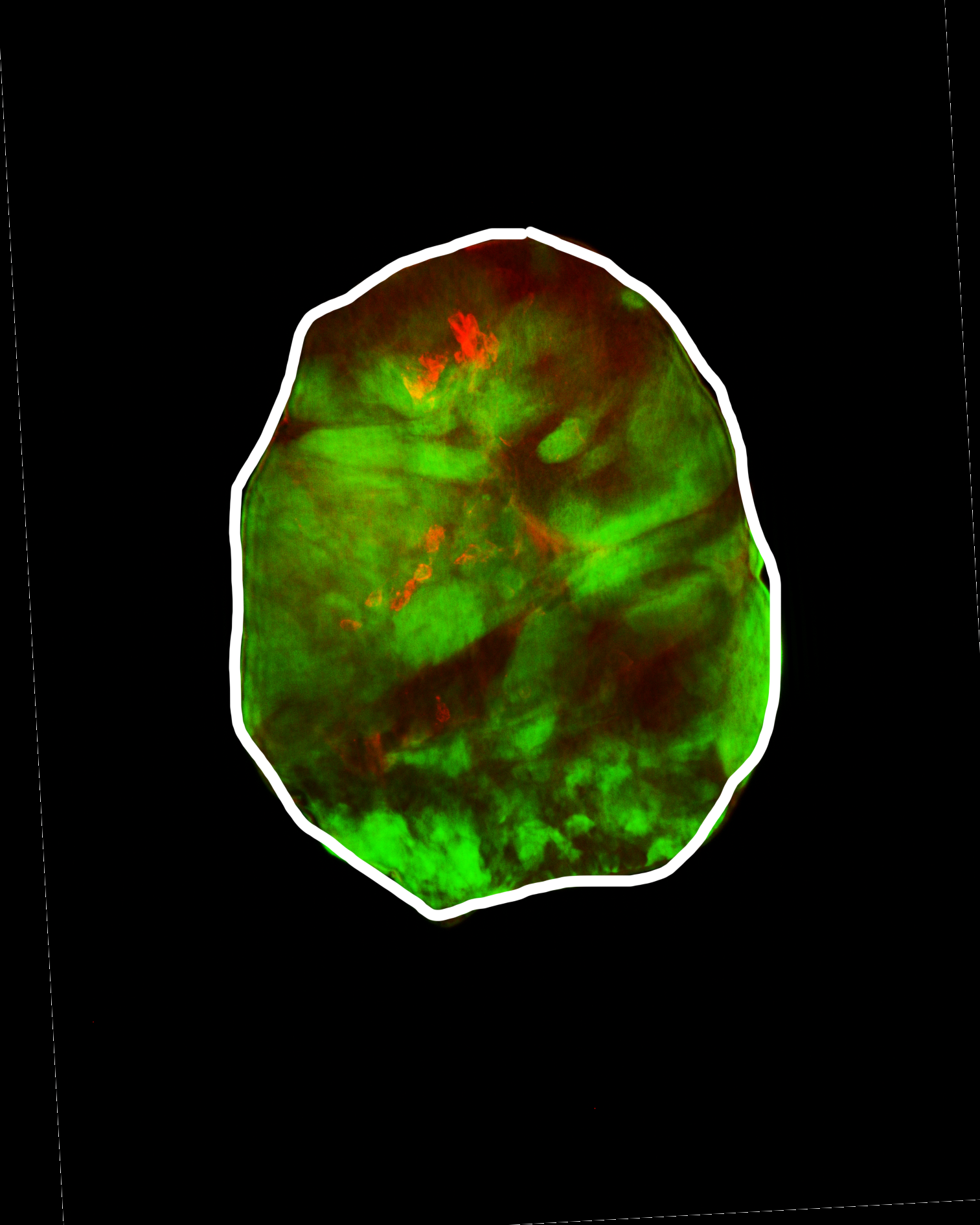

Supplement: Supplementary file 11 — Source data Fig. 7 [file 44318_2025_547_MOESM11_ESM.zip › Figure 7H/7-1 rotated and cut image with border line.tif]

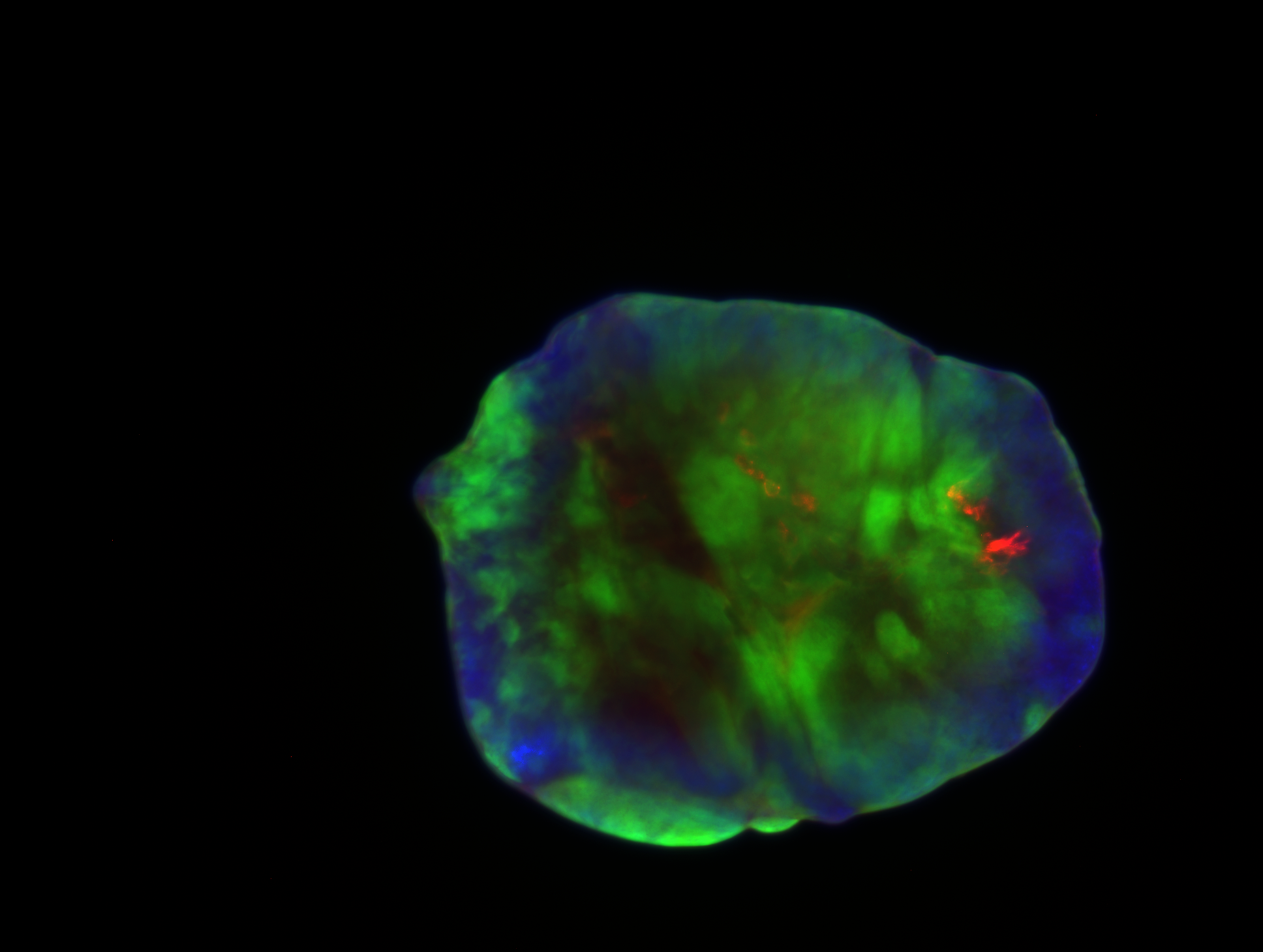

Supplement: Supplementary file 11 — Source data Fig. 7 [file 44318_2025_547_MOESM11_ESM.zip › Figure 7H/7-2 original image.tif]

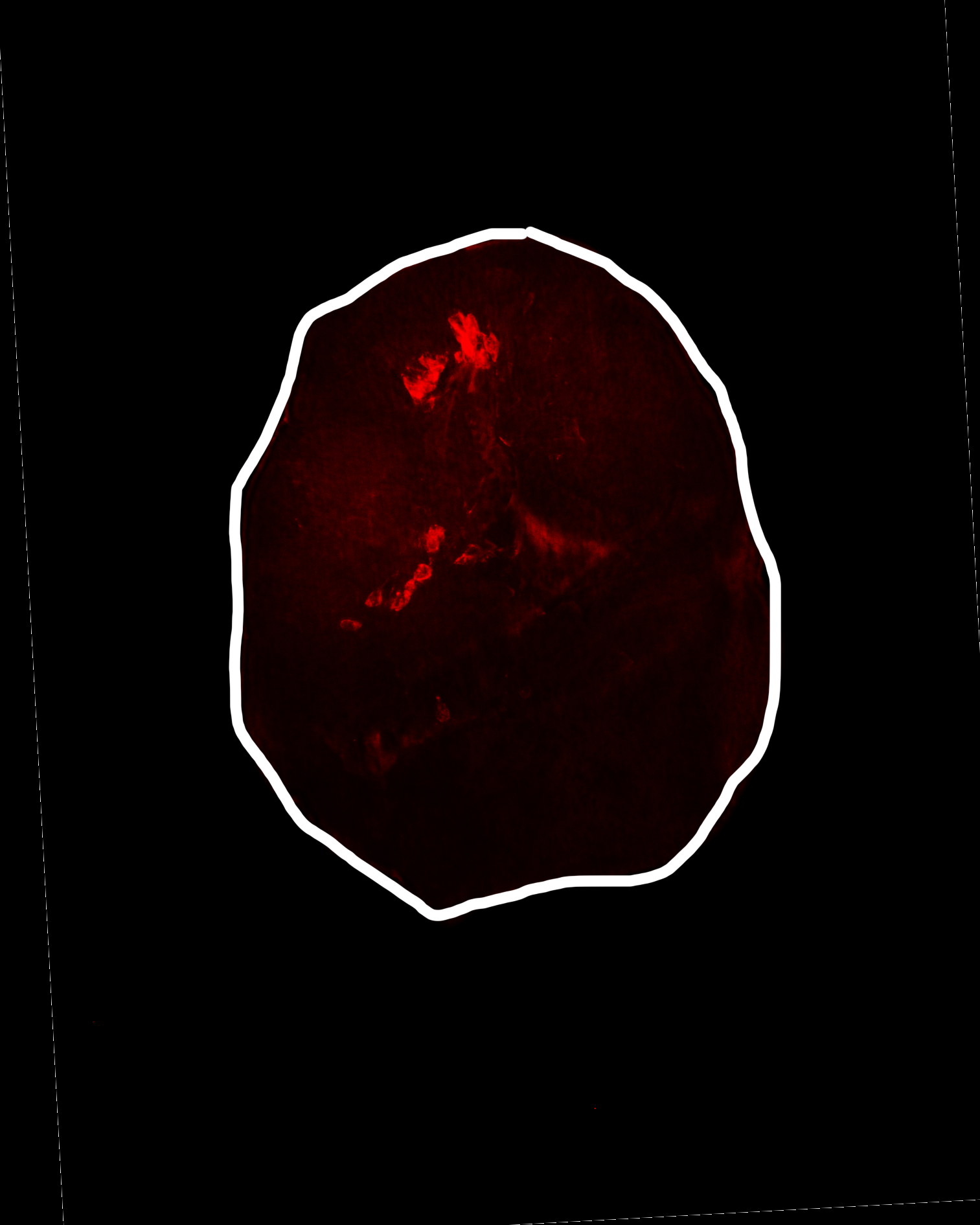

Supplement: Supplementary file 11 — Source data Fig. 7 [file 44318_2025_547_MOESM11_ESM.zip › Figure 7H/8-1 rotated and cut image with border line.tif]

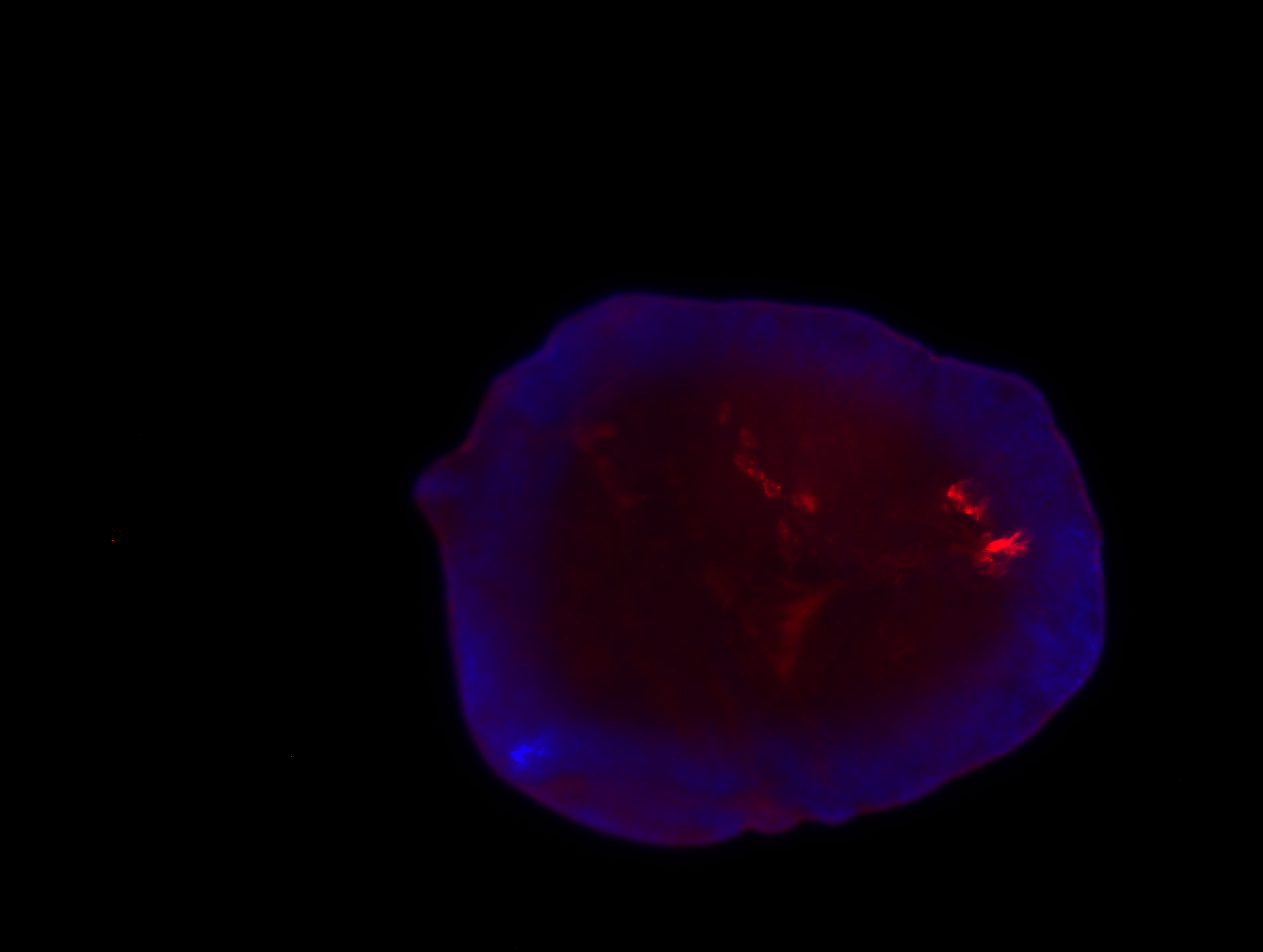

Supplement: Supplementary file 11 — Source data Fig. 7 [file 44318_2025_547_MOESM11_ESM.zip › Figure 7H/8-2 original image.tif]

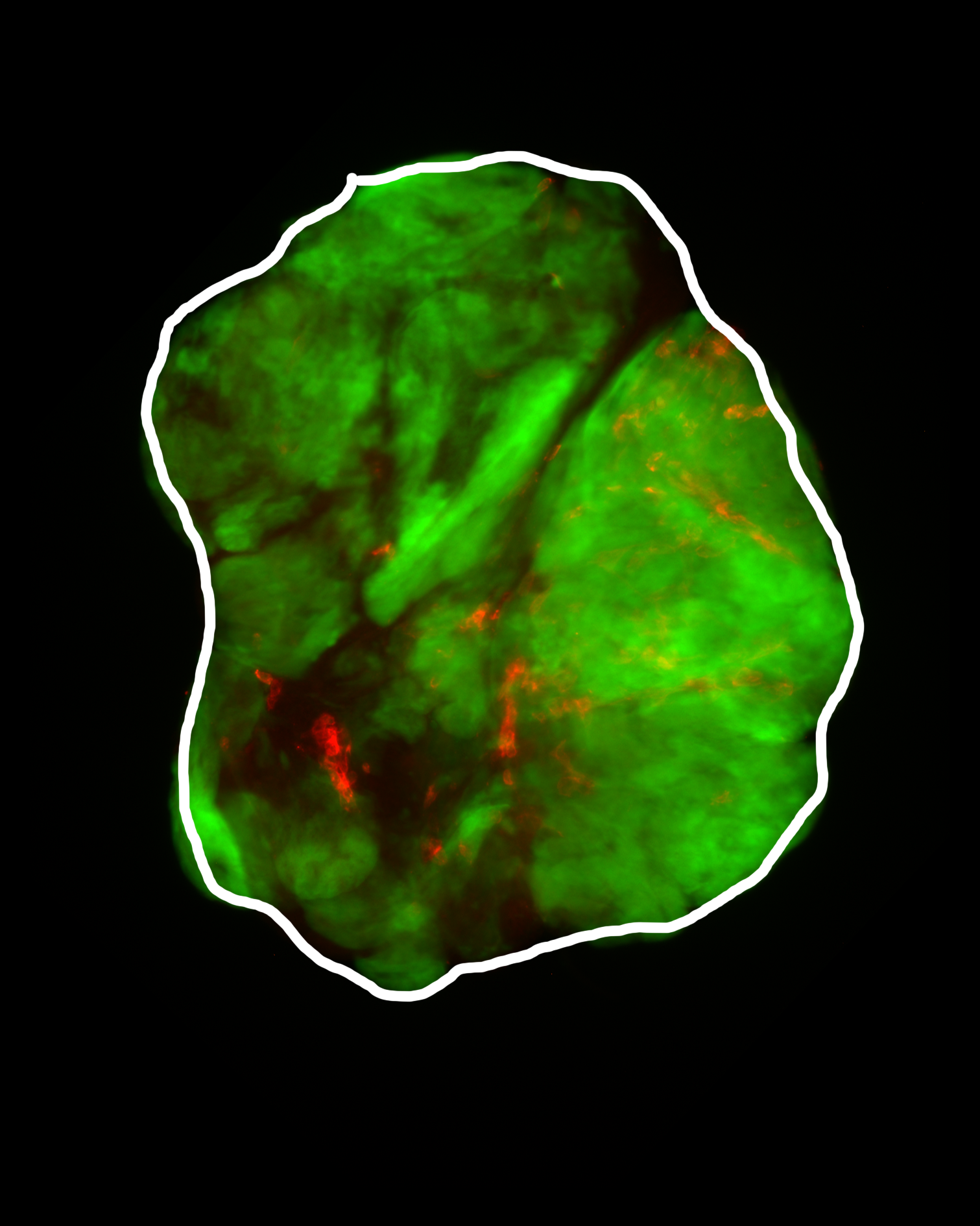

Supplement: Supplementary file 11 — Source data Fig. 7 [file 44318_2025_547_MOESM11_ESM.zip › Figure 7H/9-1 rotated and cut image with border line.tif]

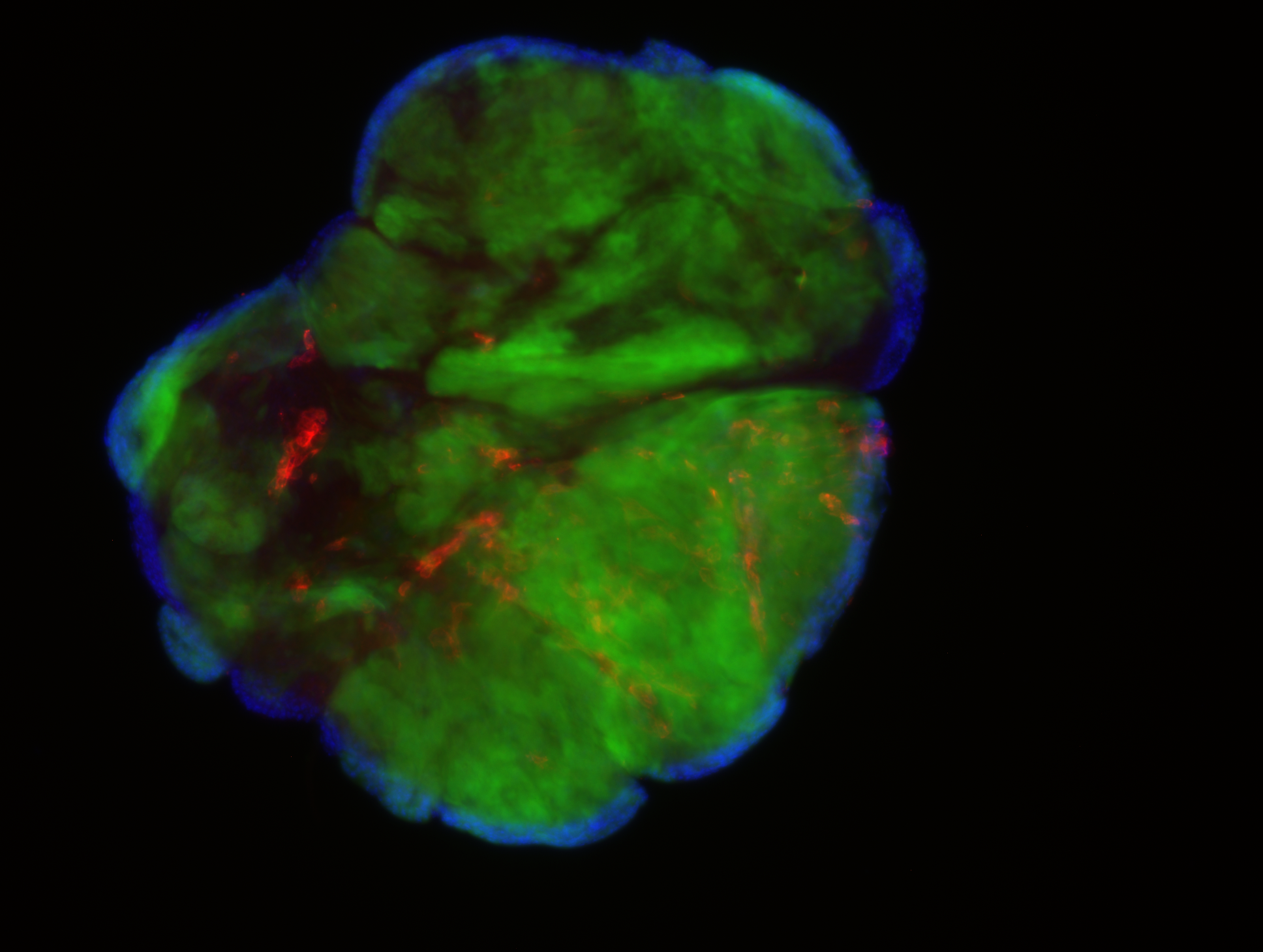

Supplement: Supplementary file 11 — Source data Fig. 7 [file 44318_2025_547_MOESM11_ESM.zip › Figure 7H/9-2 original image.tif]

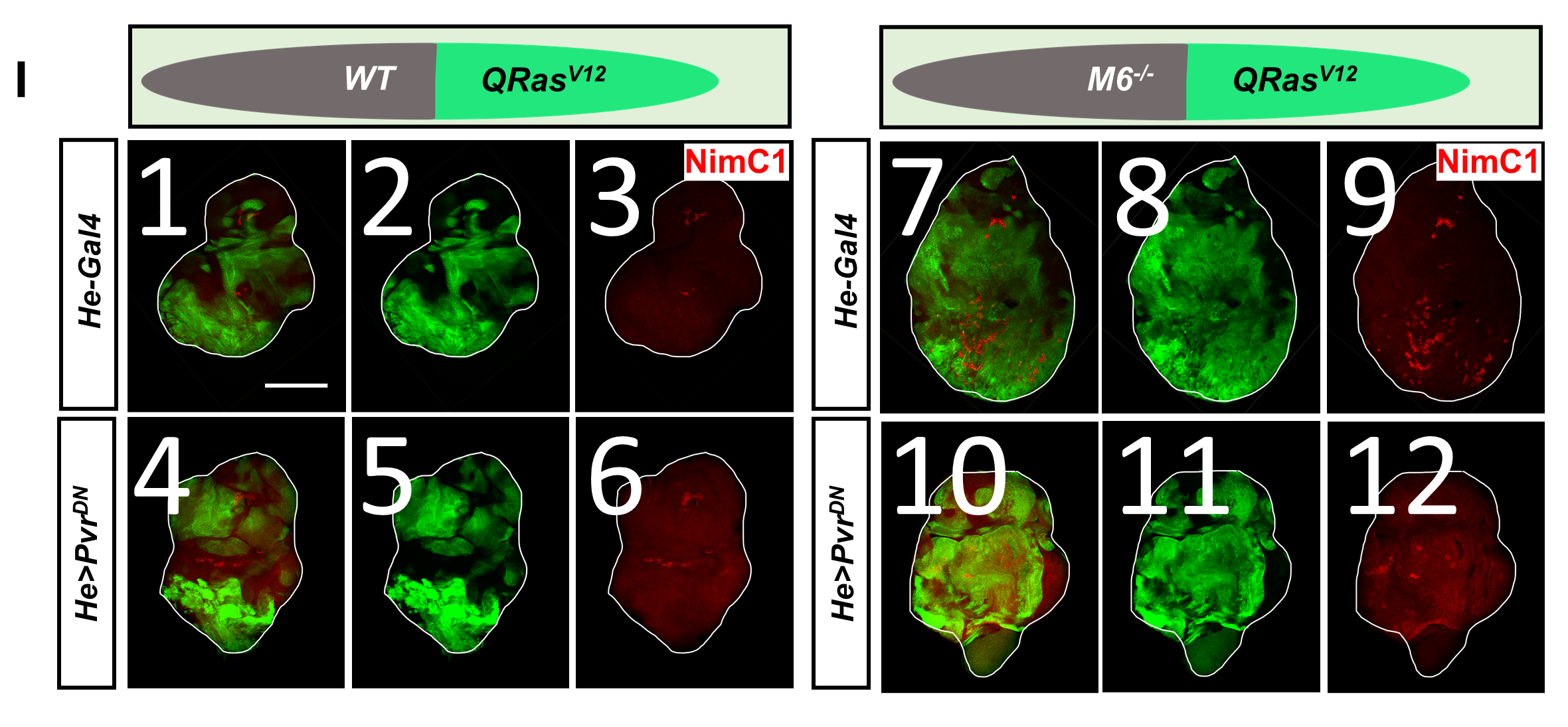

Supplement: Supplementary file 11 — Source data Fig. 7 [file 44318_2025_547_MOESM11_ESM.zip › Figure 7I/0 paper Figure 7I with provided image sequence.tif]

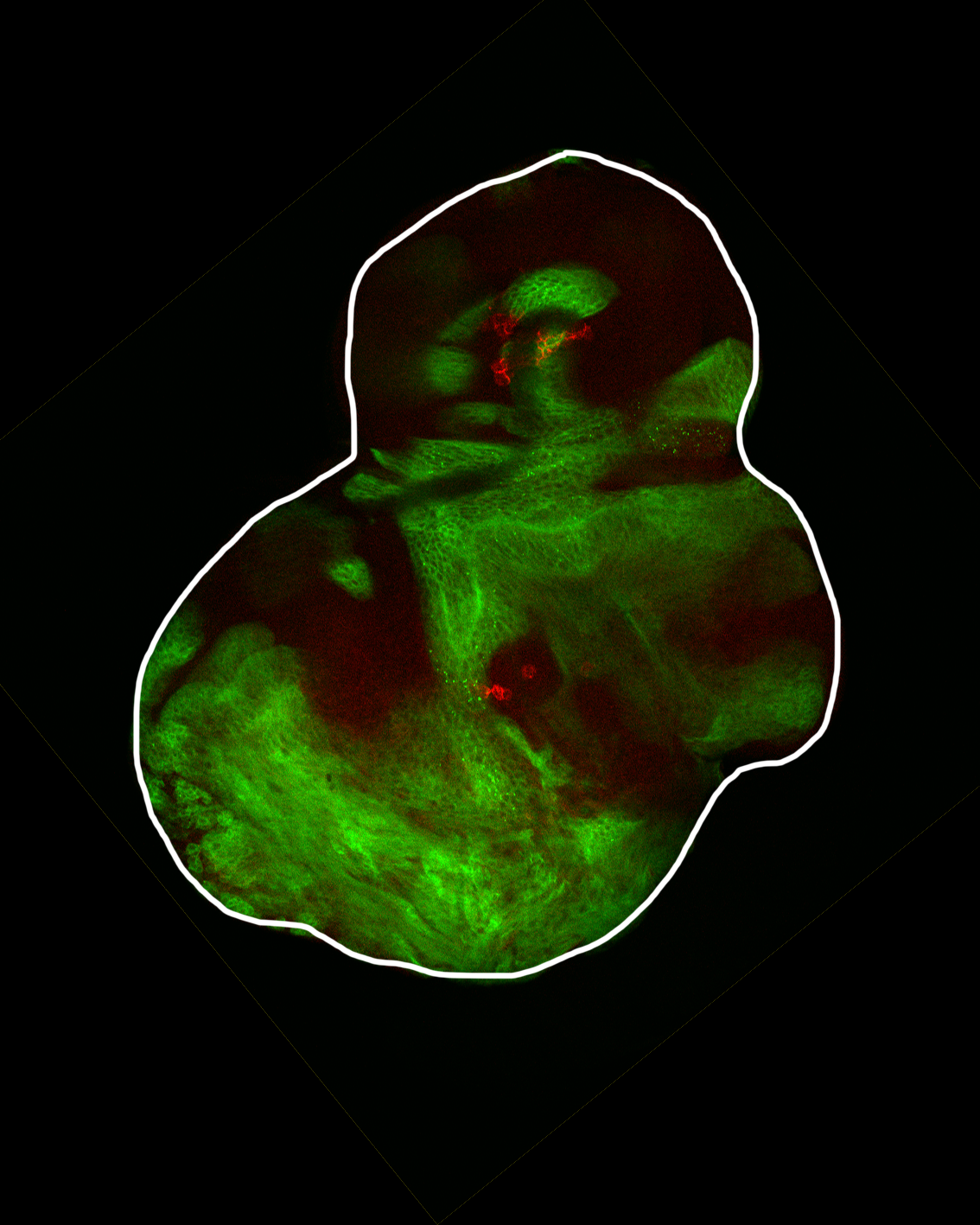

Supplement: Supplementary file 11 — Source data Fig. 7 [file 44318_2025_547_MOESM11_ESM.zip › Figure 7I/1-1 rotated and cut image with border line.tif]

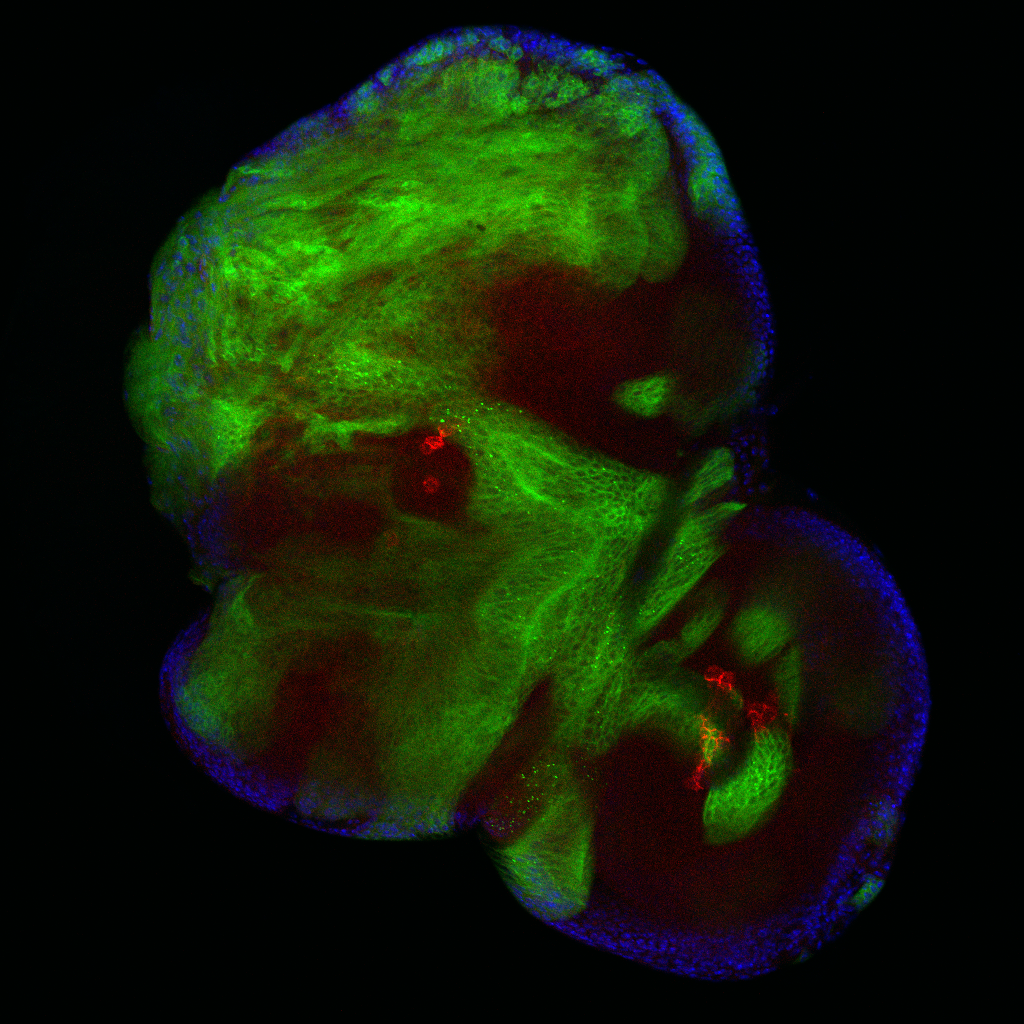

Supplement: Supplementary file 11 — Source data Fig. 7 [file 44318_2025_547_MOESM11_ESM.zip › Figure 7I/1-2 original image.tif]

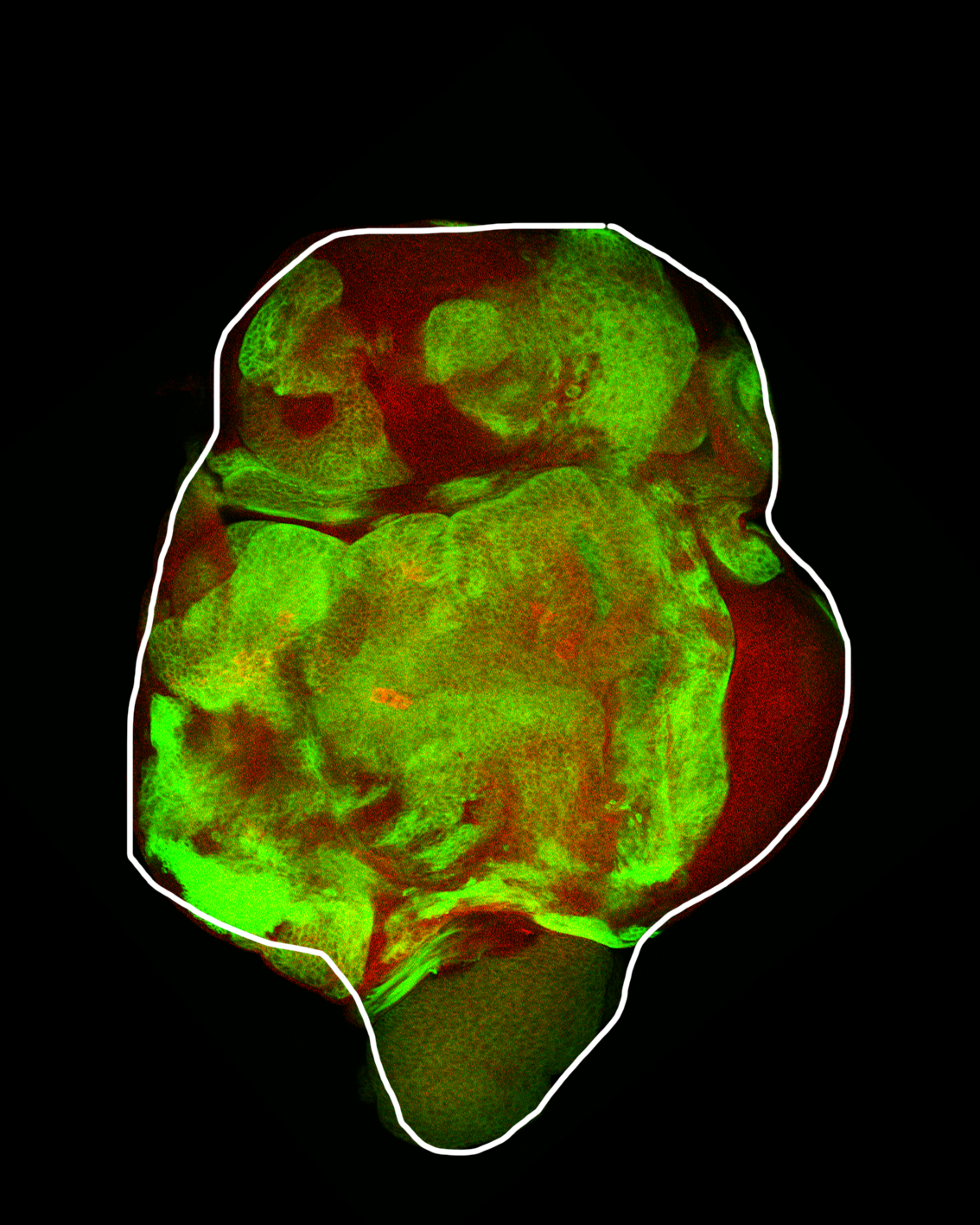

Supplement: Supplementary file 11 — Source data Fig. 7 [file 44318_2025_547_MOESM11_ESM.zip › Figure 7I/10-1 rotated and cut image with border line.tif]

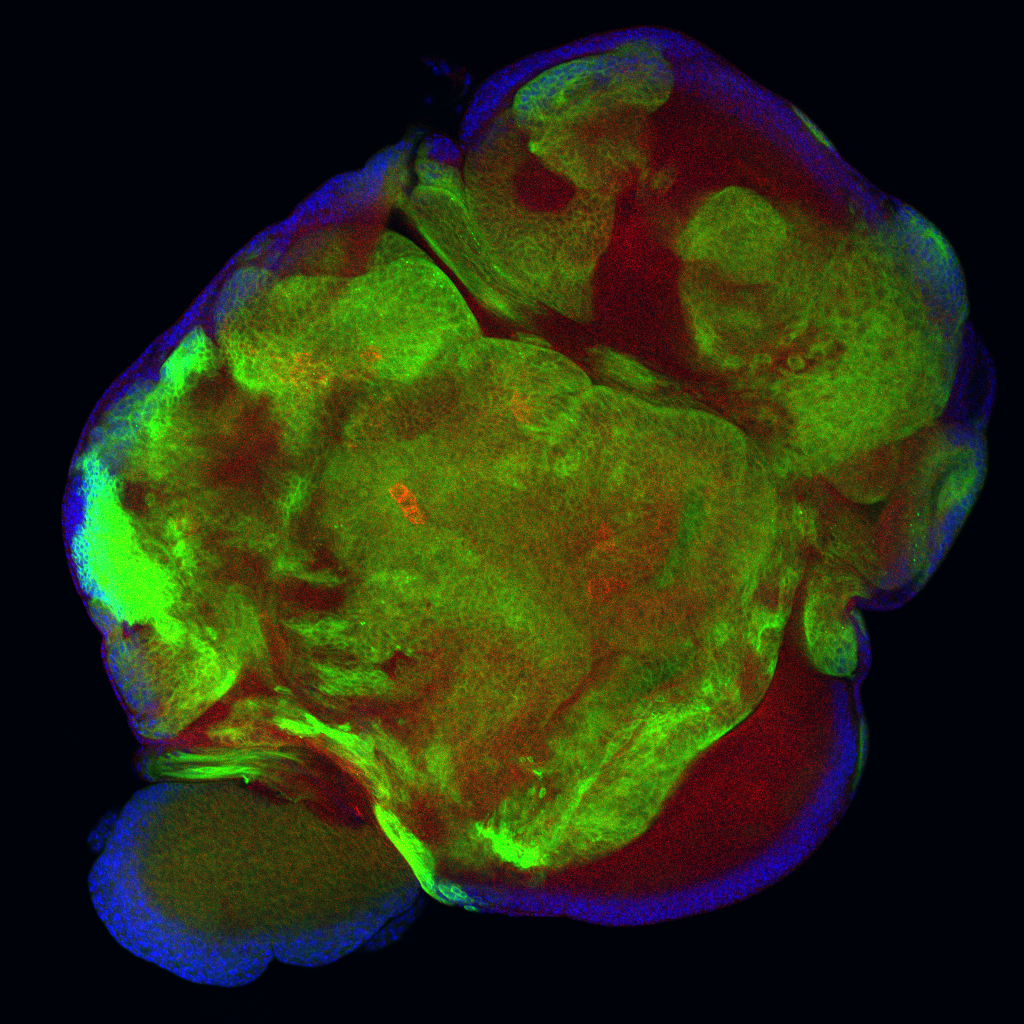

Supplement: Supplementary file 11 — Source data Fig. 7 [file 44318_2025_547_MOESM11_ESM.zip › Figure 7I/10-2 original image.tif]

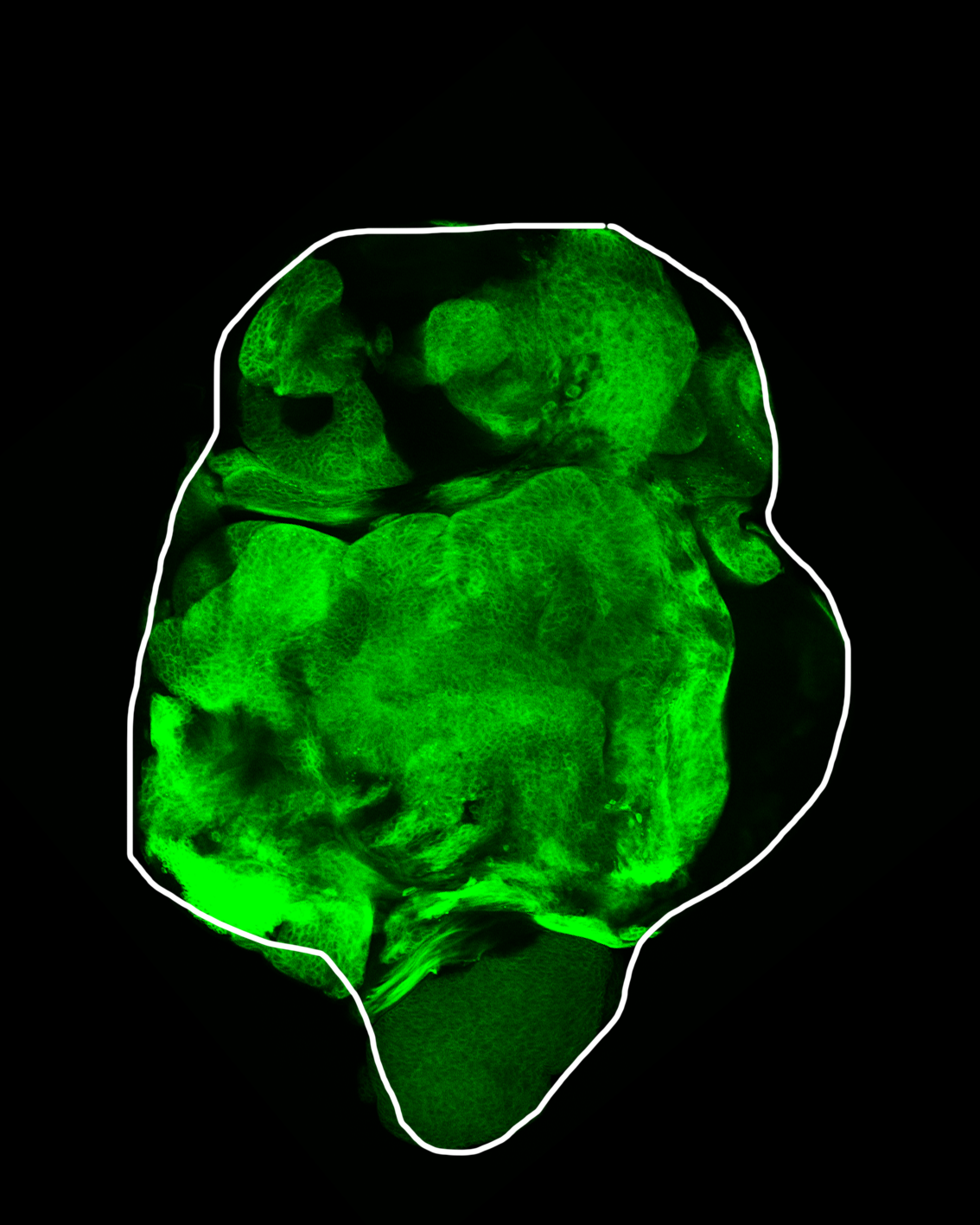

Supplement: Supplementary file 11 — Source data Fig. 7 [file 44318_2025_547_MOESM11_ESM.zip › Figure 7I/11-1 rotated and cut image with border line.tif]

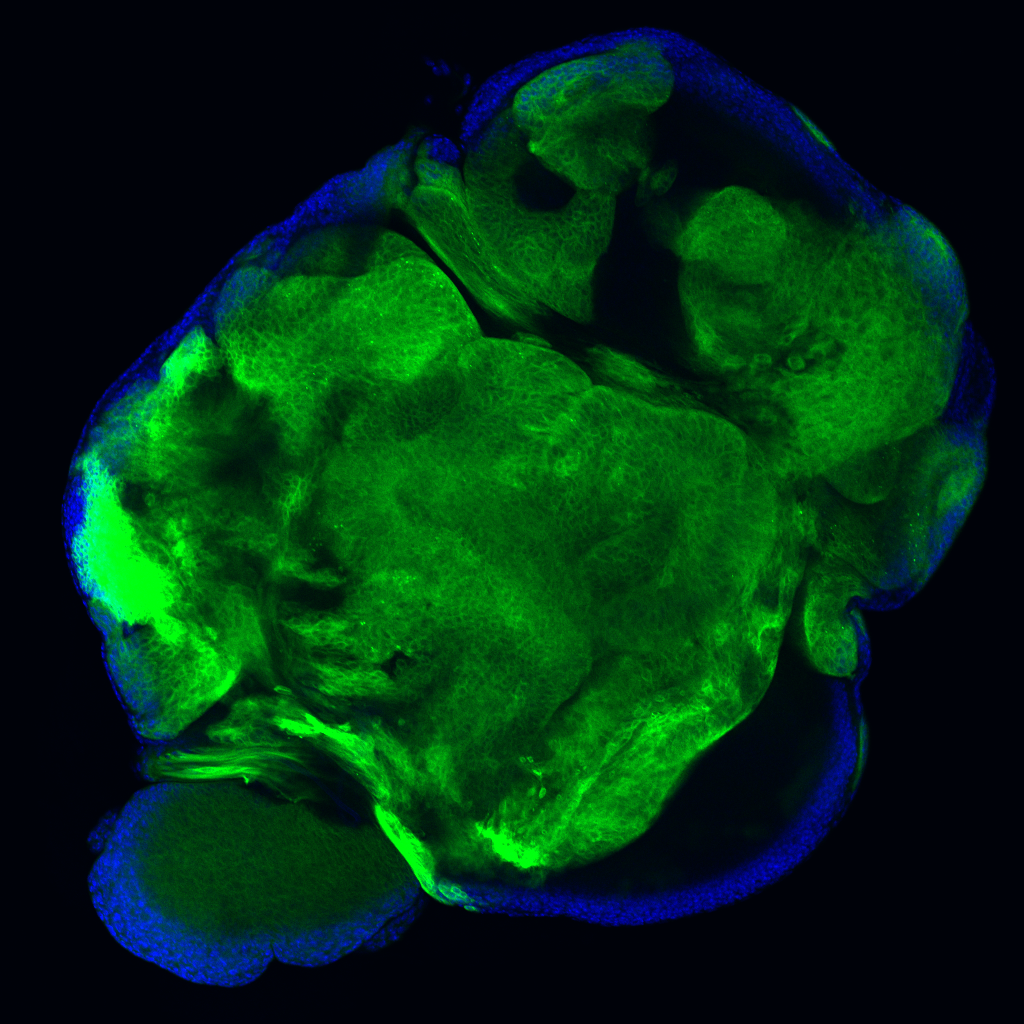

Supplement: Supplementary file 11 — Source data Fig. 7 [file 44318_2025_547_MOESM11_ESM.zip › Figure 7I/11-2 original image.tif]

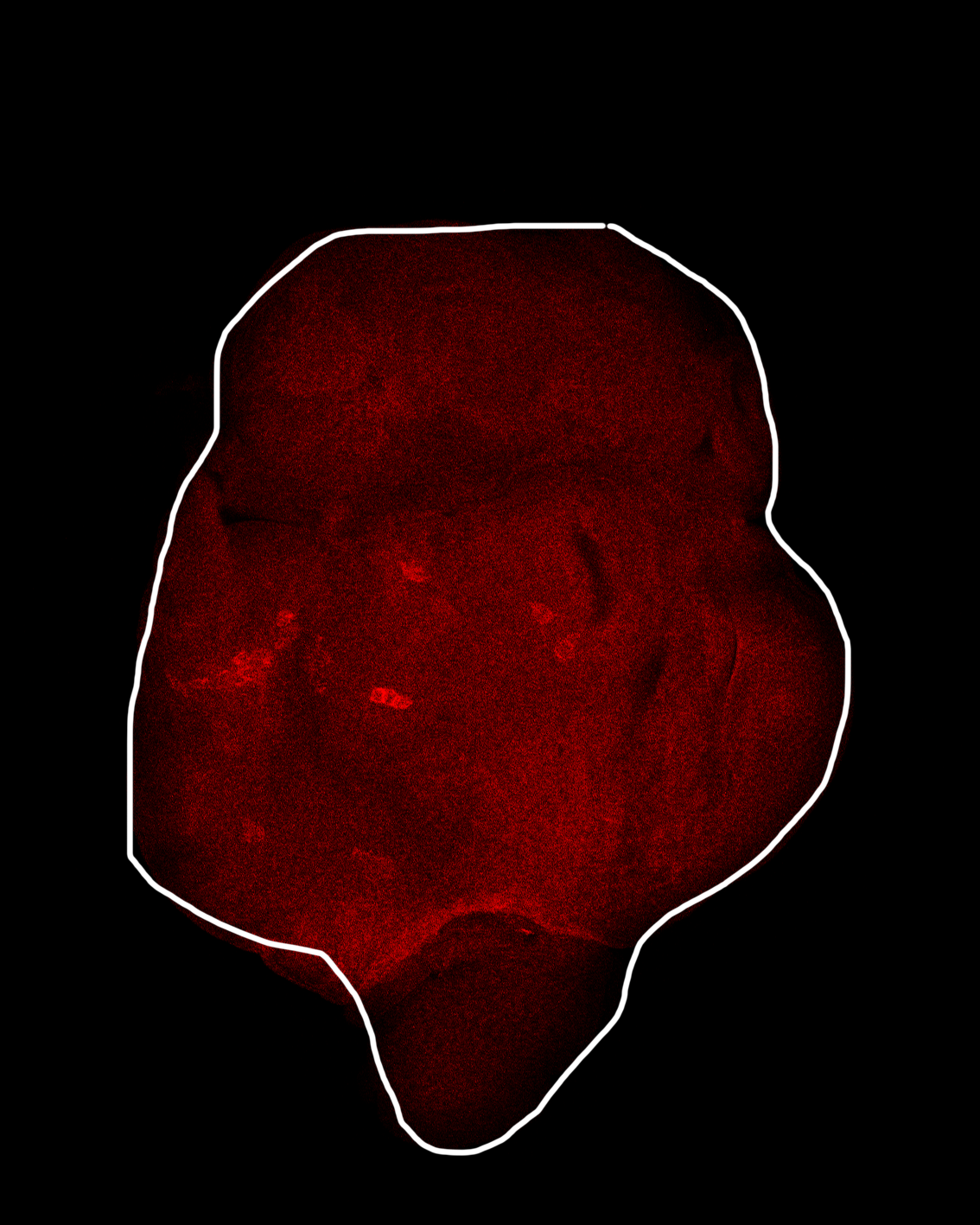

Supplement: Supplementary file 11 — Source data Fig. 7 [file 44318_2025_547_MOESM11_ESM.zip › Figure 7I/12-1 rotated and cut image with border line.tif]

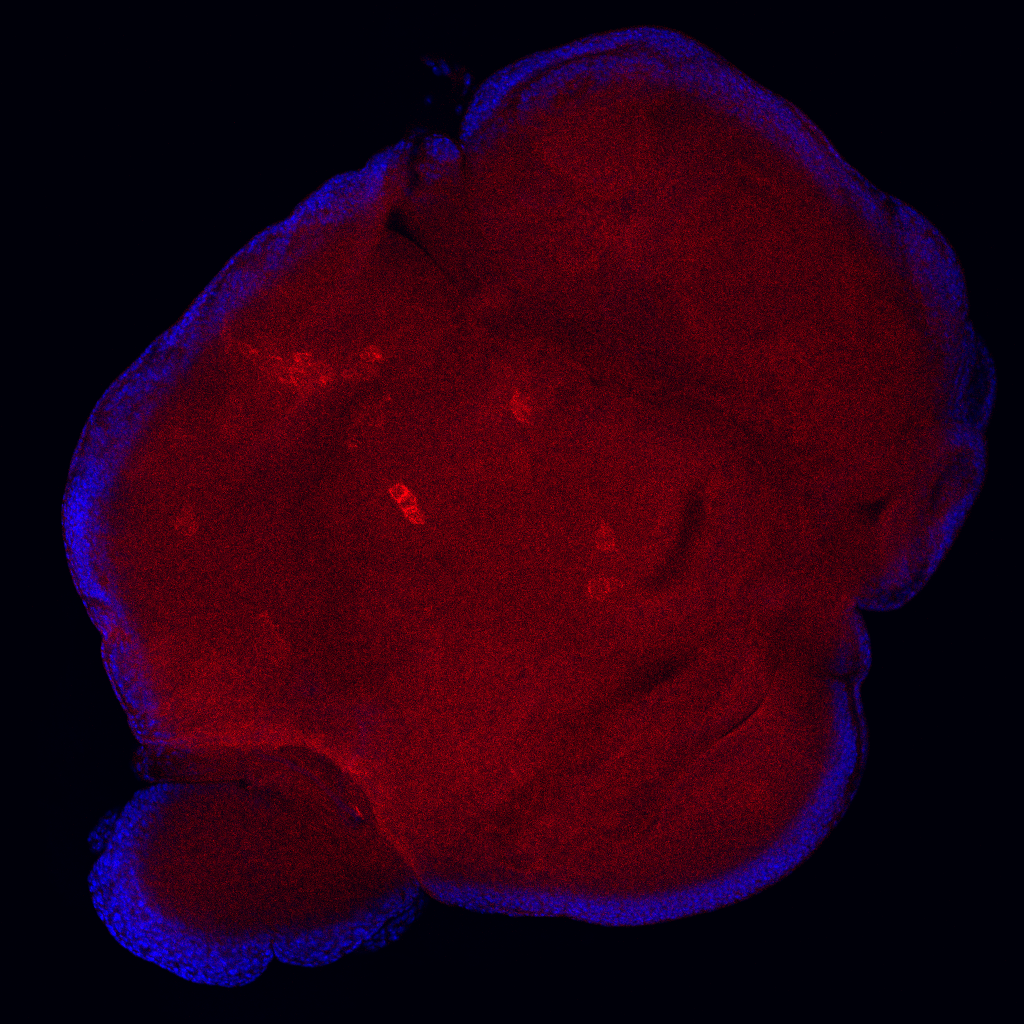

Supplement: Supplementary file 11 — Source data Fig. 7 [file 44318_2025_547_MOESM11_ESM.zip › Figure 7I/12-2 original image.tif]

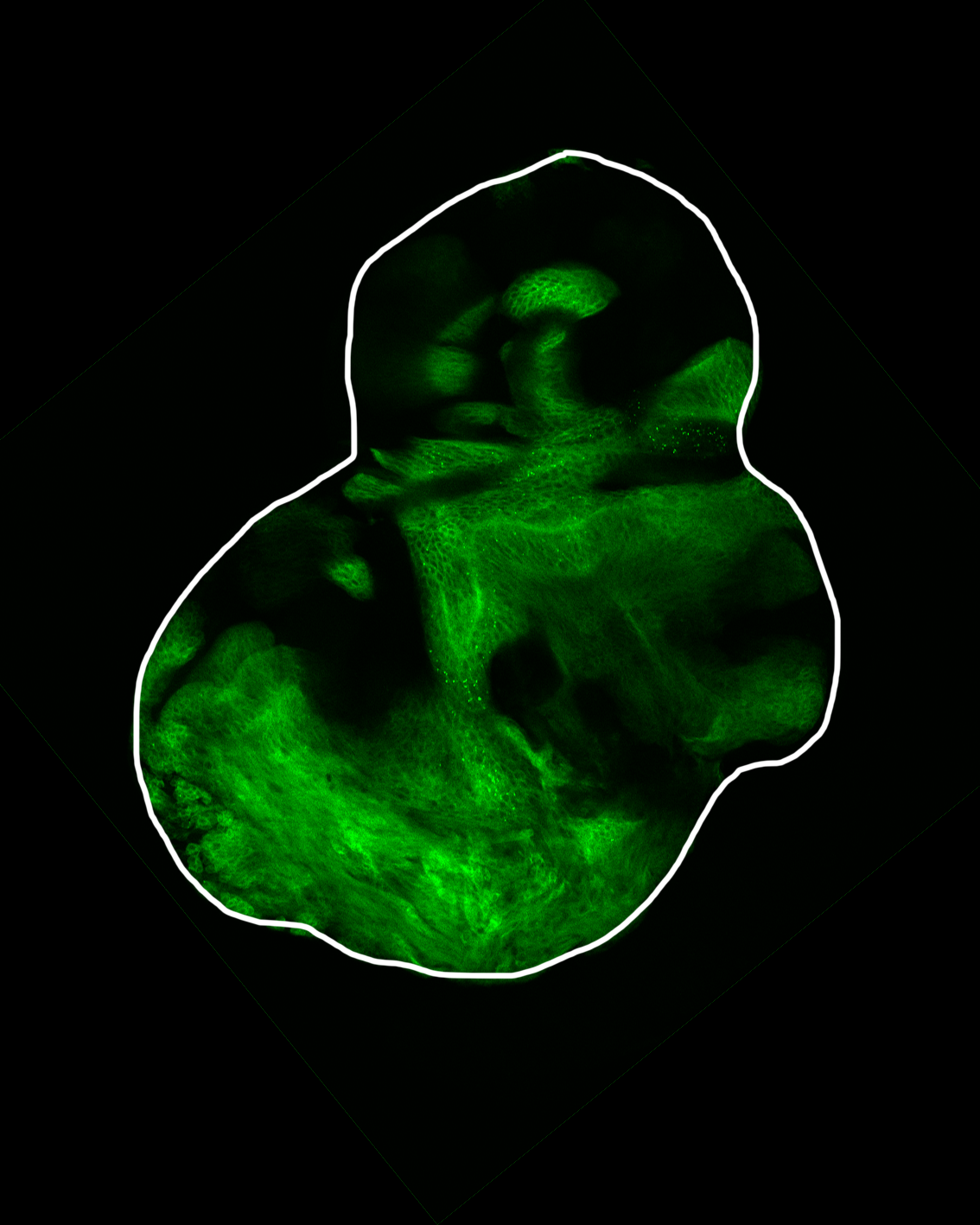

Supplement: Supplementary file 11 — Source data Fig. 7 [file 44318_2025_547_MOESM11_ESM.zip › Figure 7I/2-1 rotated and cut image with border line.tif]

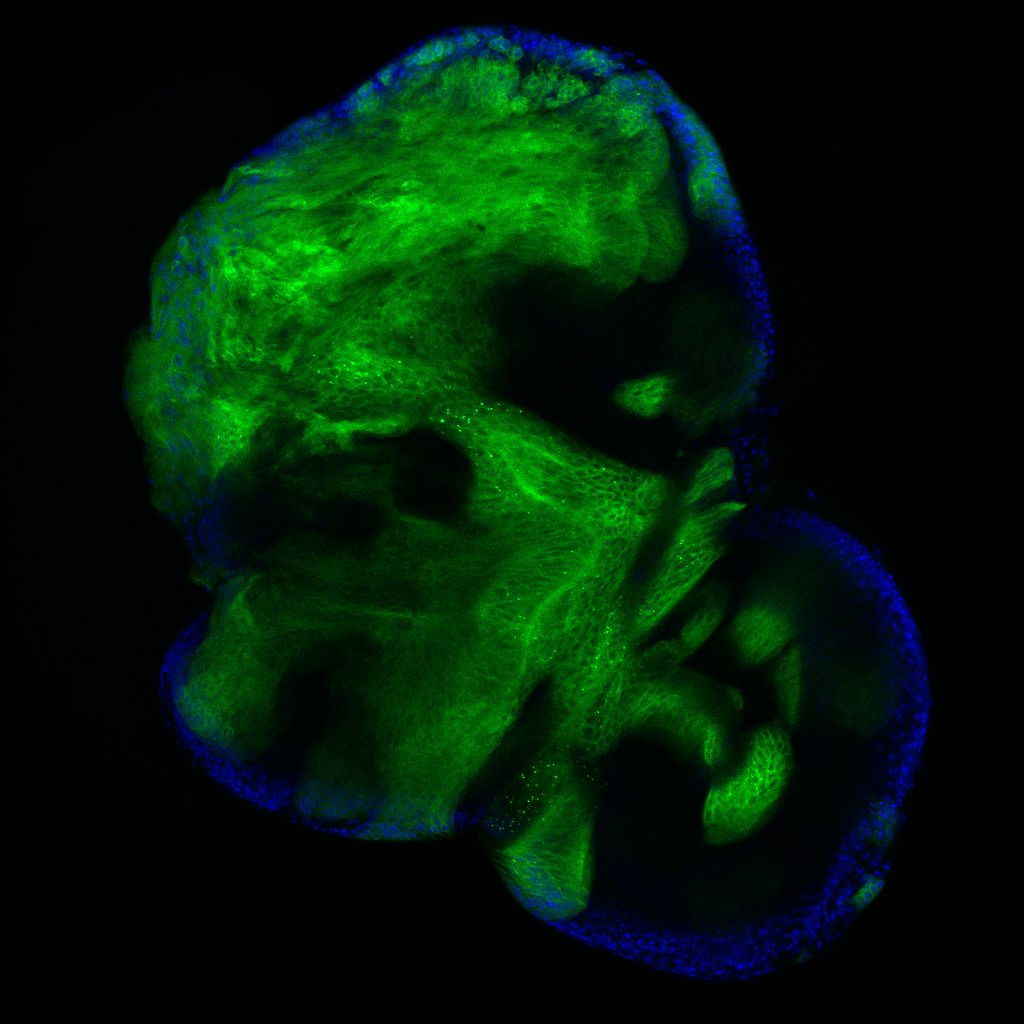

Supplement: Supplementary file 11 — Source data Fig. 7 [file 44318_2025_547_MOESM11_ESM.zip › Figure 7I/2-2 original image.tif]

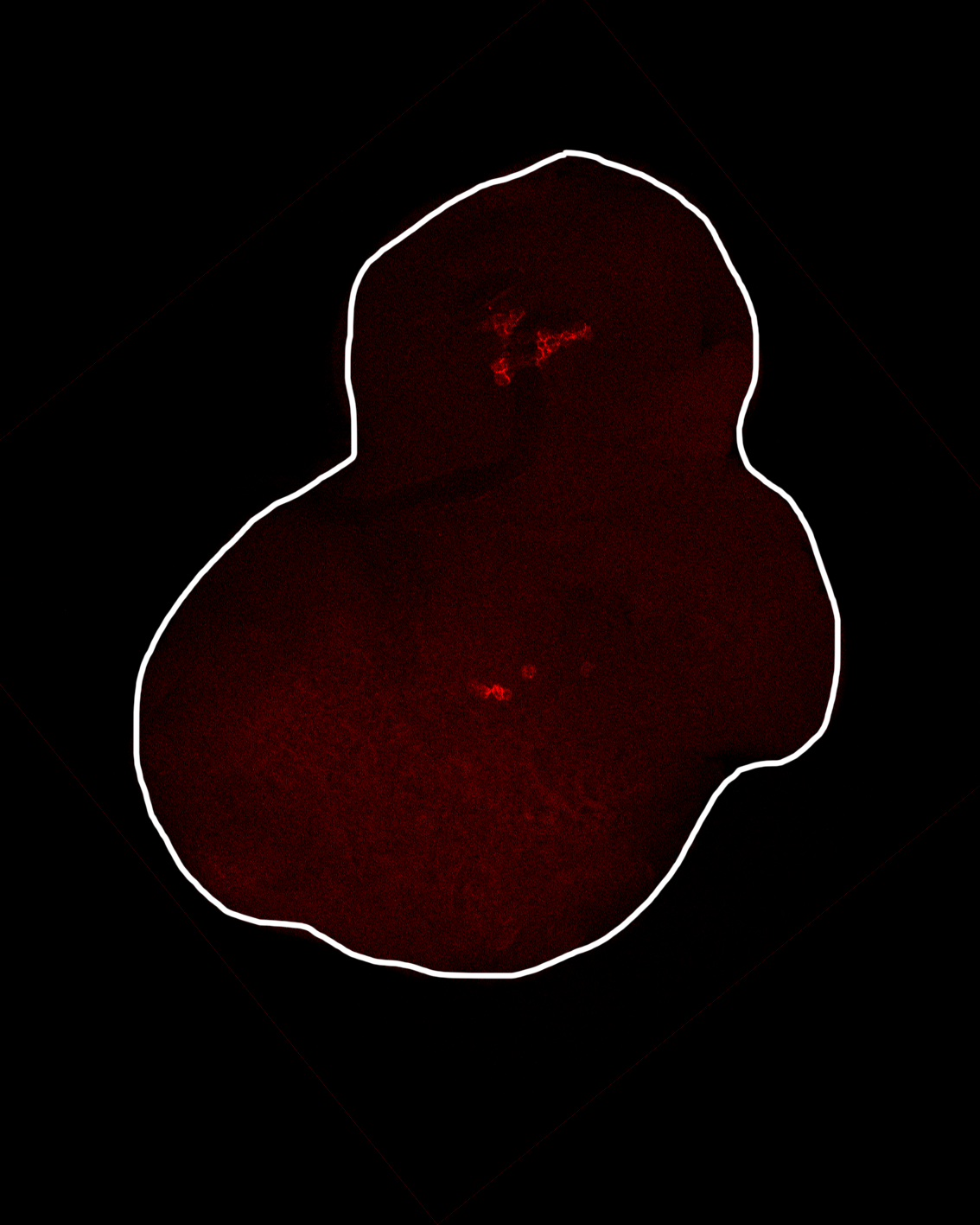

Supplement: Supplementary file 11 — Source data Fig. 7 [file 44318_2025_547_MOESM11_ESM.zip › Figure 7I/3-1 rotated and cut image with border line.tif]

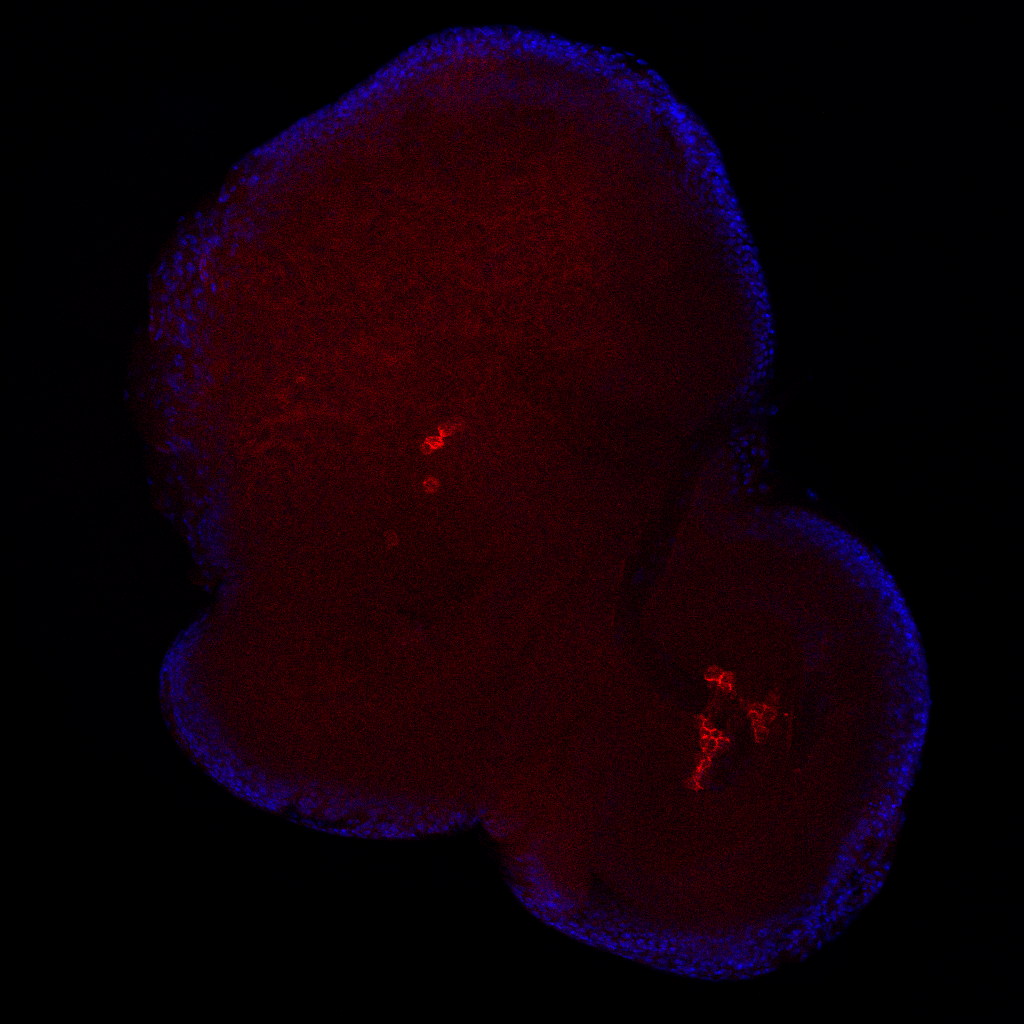

Supplement: Supplementary file 11 — Source data Fig. 7 [file 44318_2025_547_MOESM11_ESM.zip › Figure 7I/3-2 original image.tif]

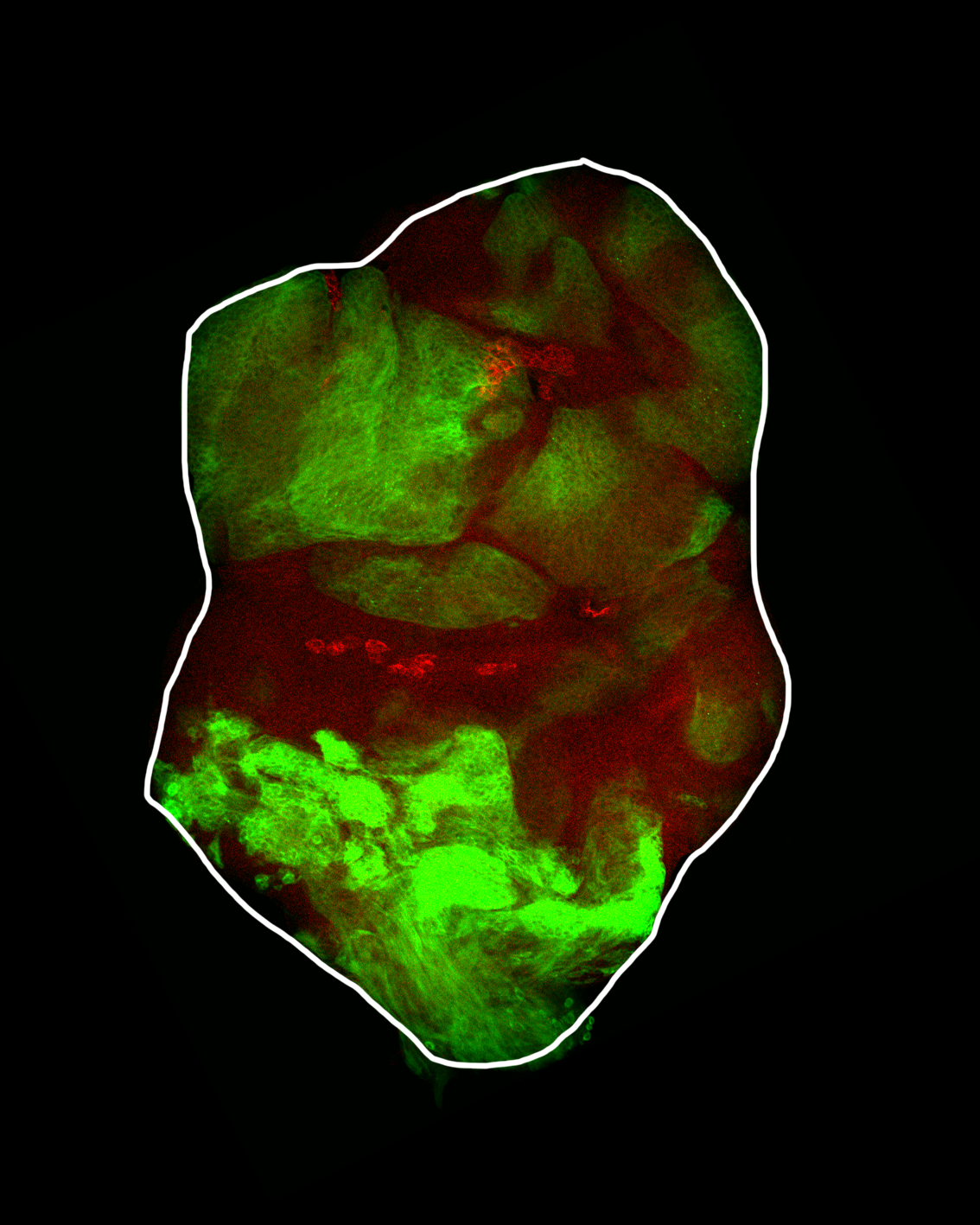

Supplement: Supplementary file 11 — Source data Fig. 7 [file 44318_2025_547_MOESM11_ESM.zip › Figure 7I/4-1 rotated and cut image with border line.tif]

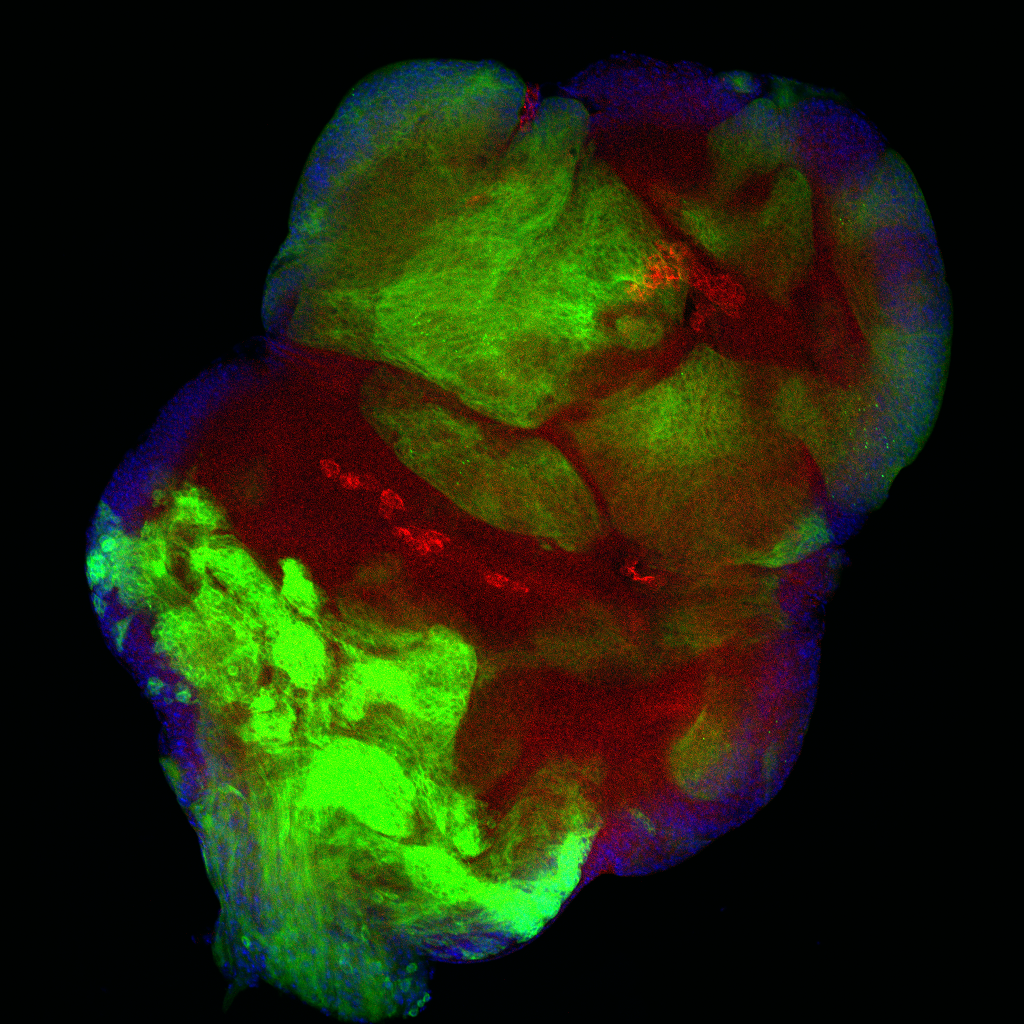

Supplement: Supplementary file 11 — Source data Fig. 7 [file 44318_2025_547_MOESM11_ESM.zip › Figure 7I/4-2 original image.tif]

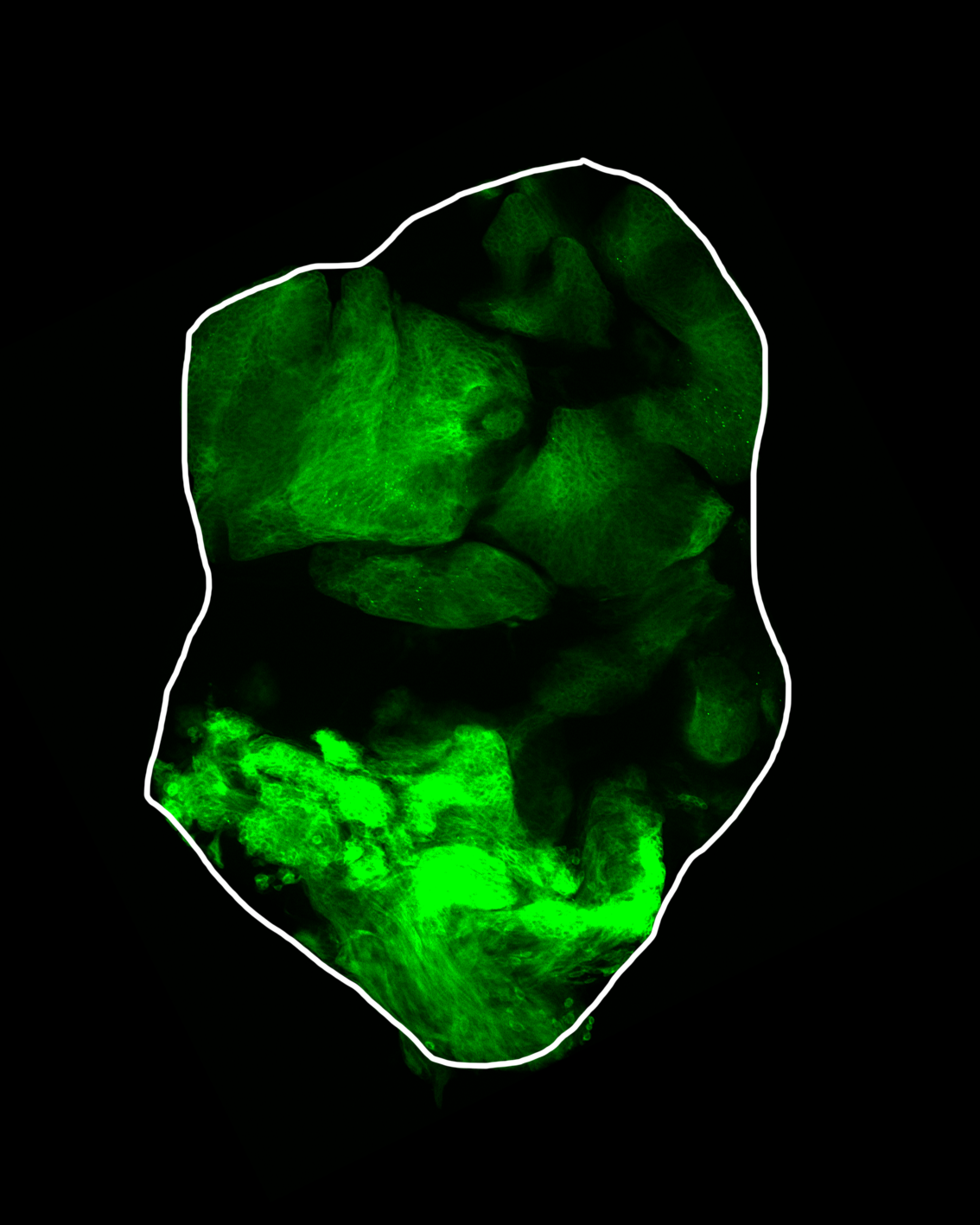

Supplement: Supplementary file 11 — Source data Fig. 7 [file 44318_2025_547_MOESM11_ESM.zip › Figure 7I/5-1 rotated and cut image with border line.tif]

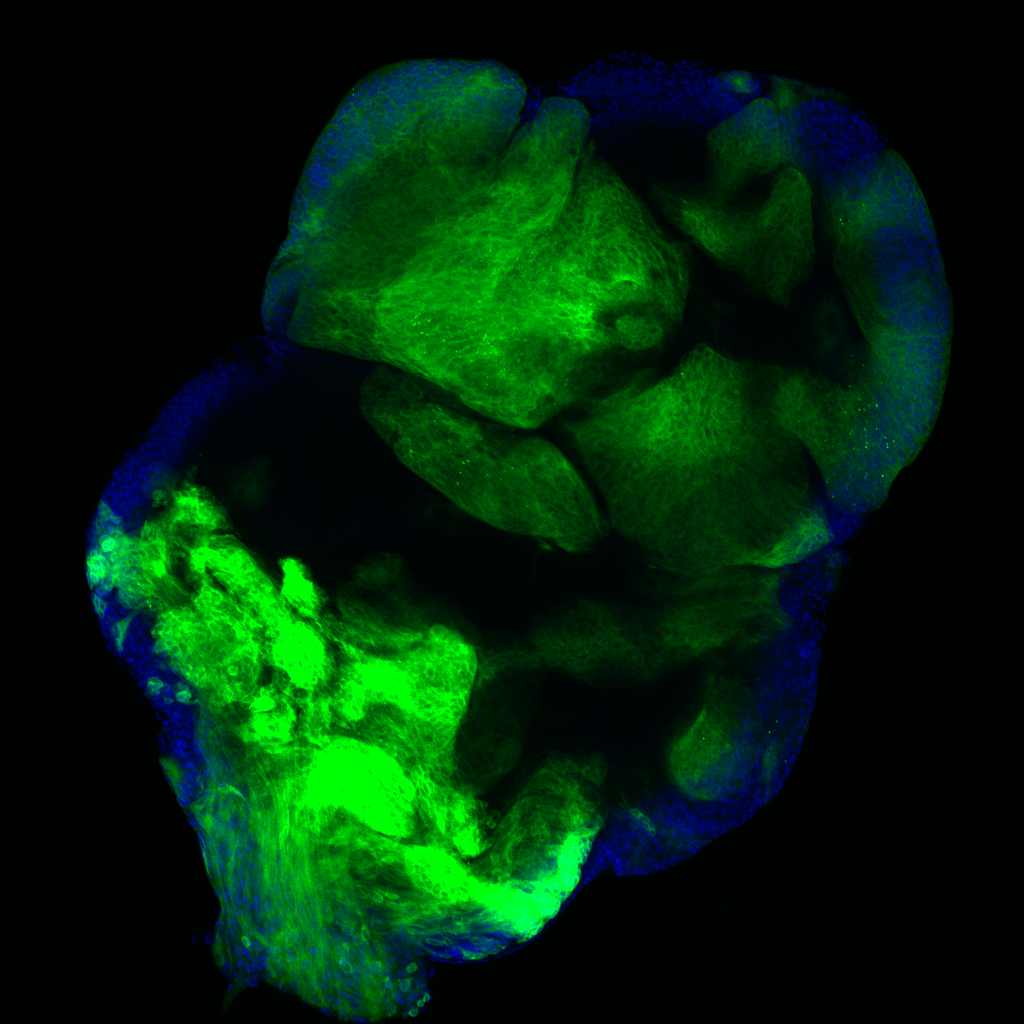

Supplement: Supplementary file 11 — Source data Fig. 7 [file 44318_2025_547_MOESM11_ESM.zip › Figure 7I/5-2 original image.tif]

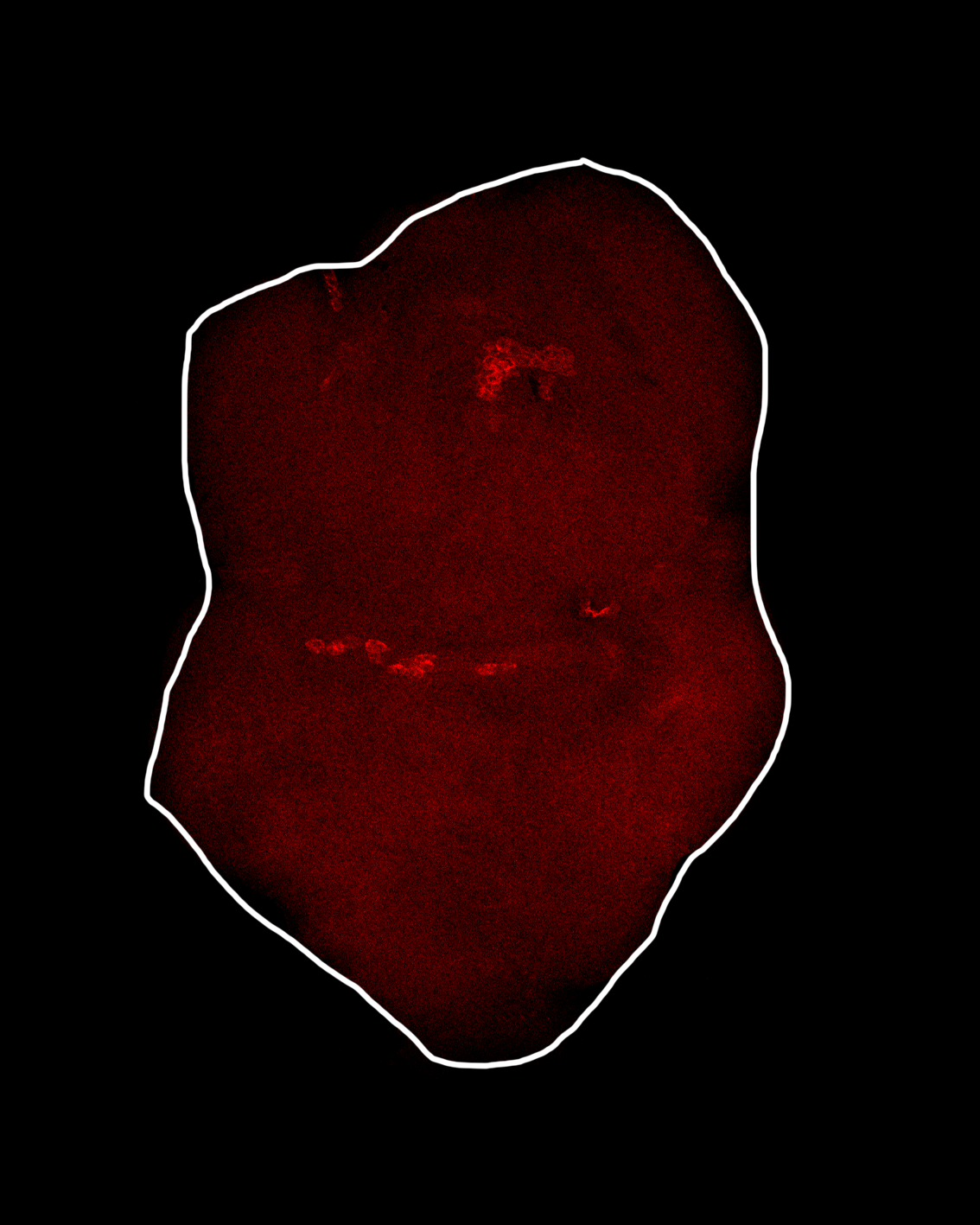

Supplement: Supplementary file 11 — Source data Fig. 7 [file 44318_2025_547_MOESM11_ESM.zip › Figure 7I/6-1 rotated and cut image with border line.tif]

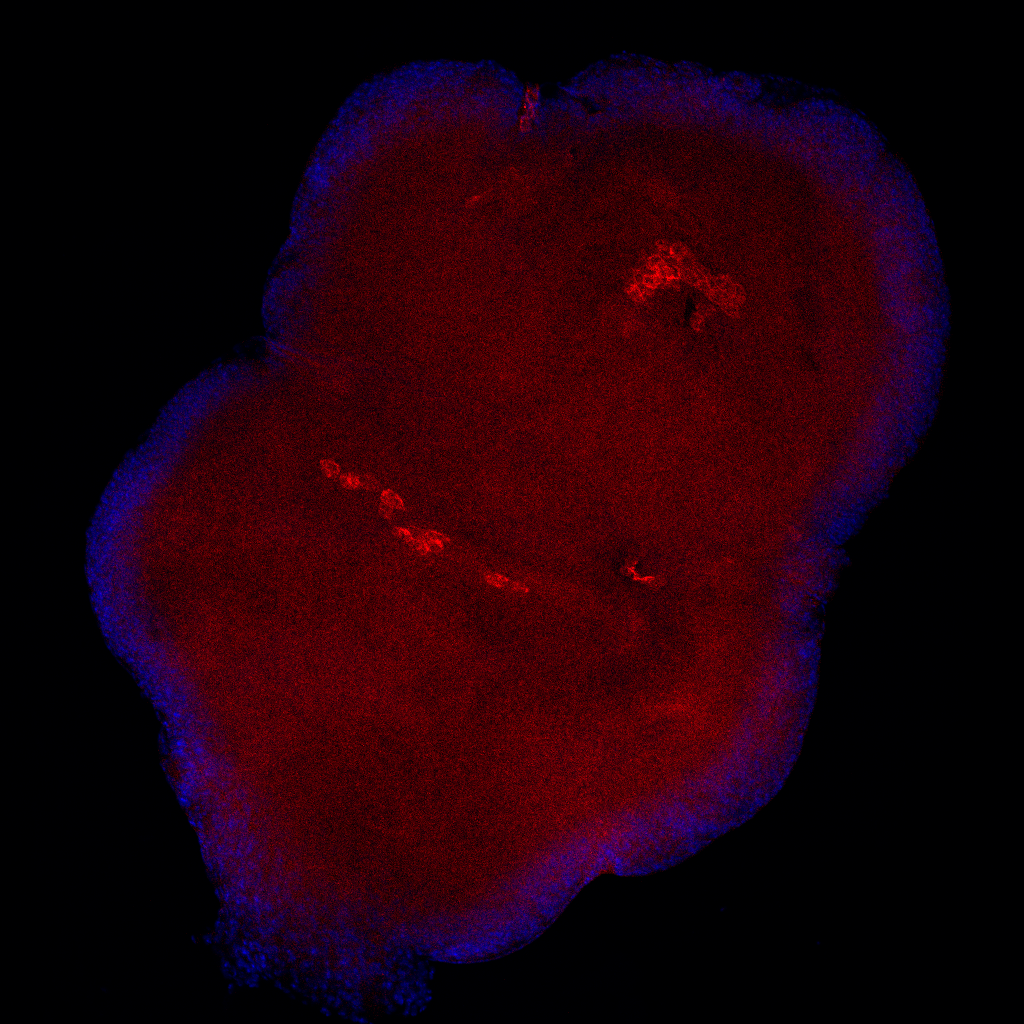

Supplement: Supplementary file 11 — Source data Fig. 7 [file 44318_2025_547_MOESM11_ESM.zip › Figure 7I/6-2 original image.tif]

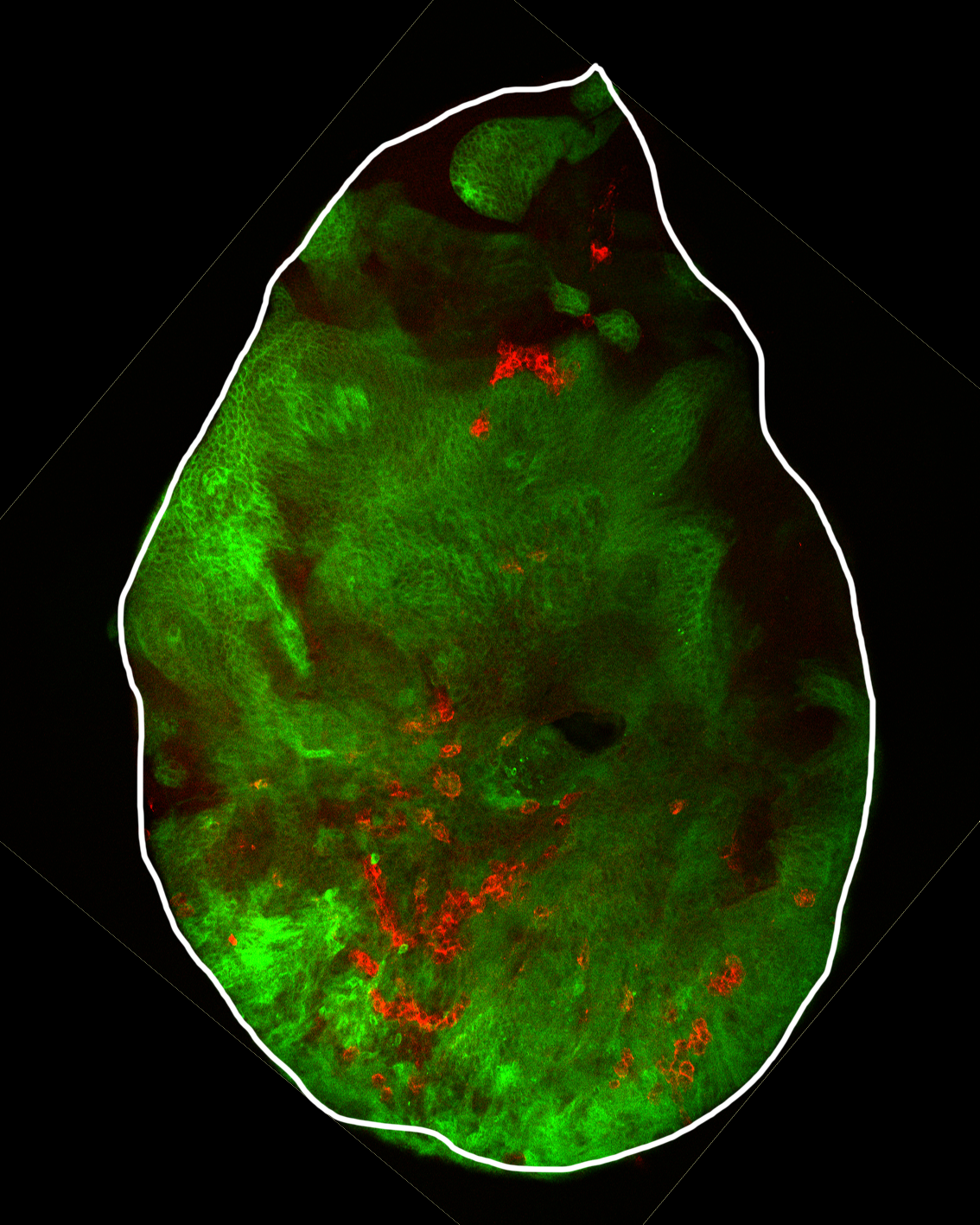

Supplement: Supplementary file 11 — Source data Fig. 7 [file 44318_2025_547_MOESM11_ESM.zip › Figure 7I/7-1 rotated and cut image with border line.tif]

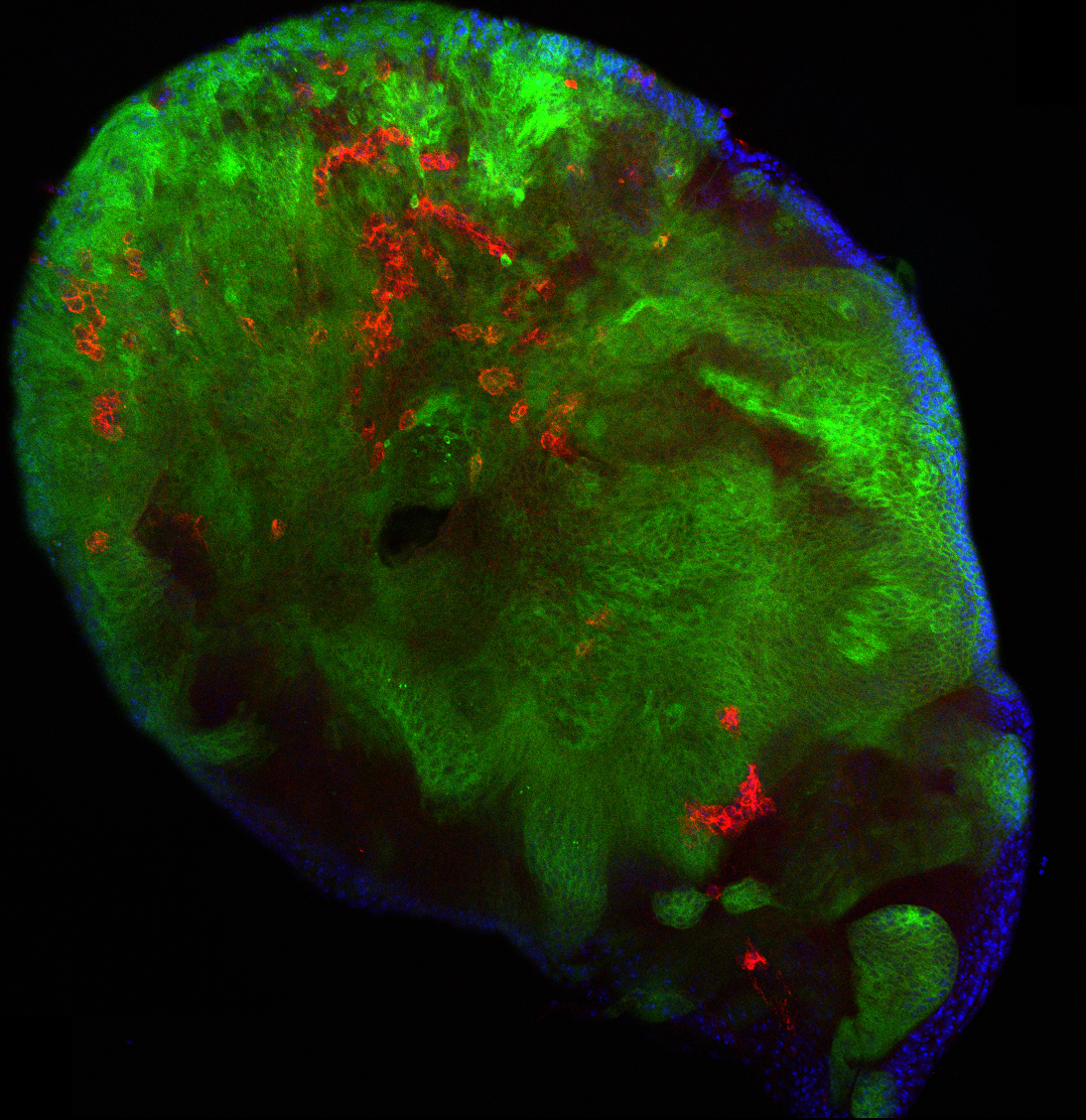

Supplement: Supplementary file 11 — Source data Fig. 7 [file 44318_2025_547_MOESM11_ESM.zip › Figure 7I/7-2 original image.tif]

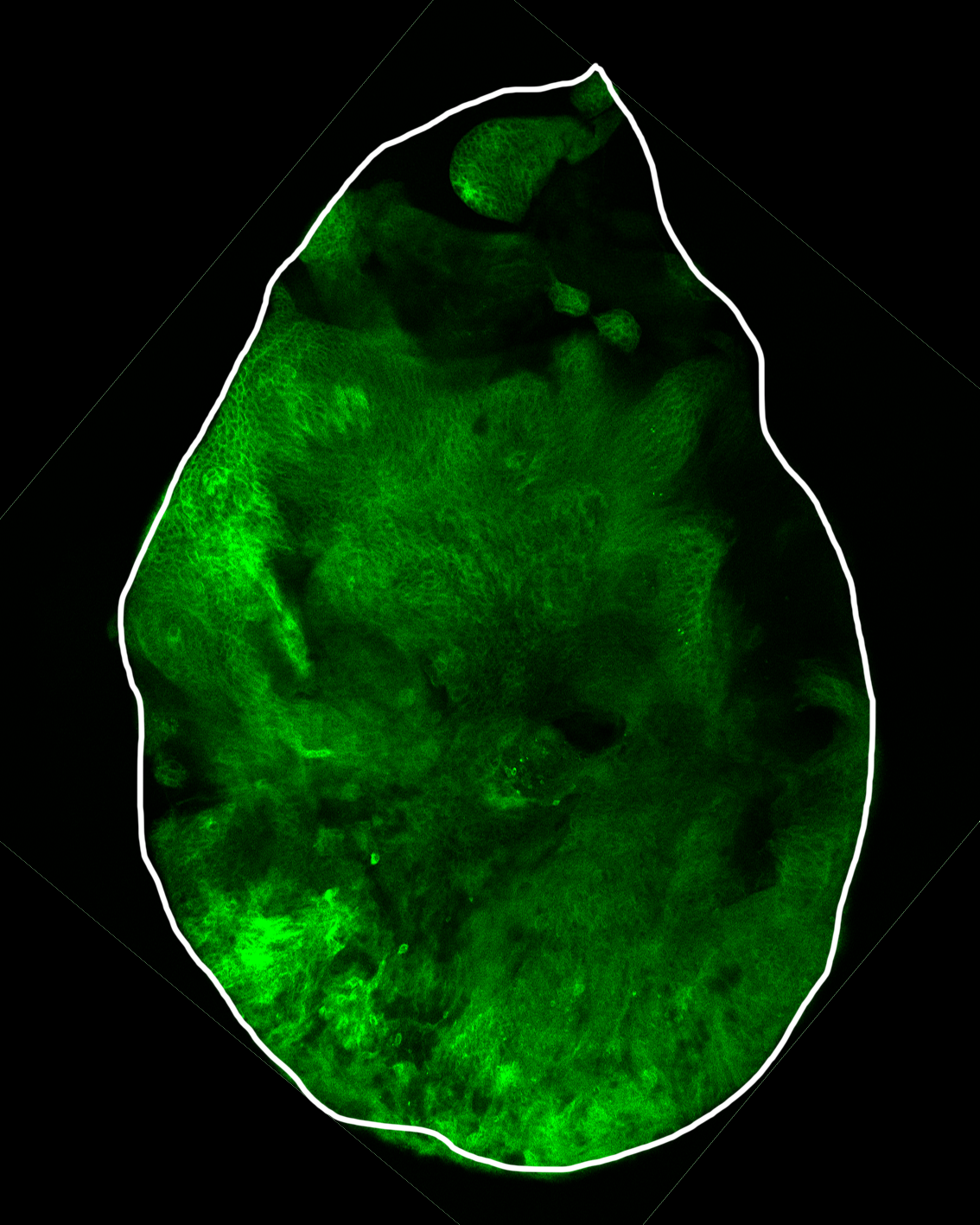

Supplement: Supplementary file 11 — Source data Fig. 7 [file 44318_2025_547_MOESM11_ESM.zip › Figure 7I/8-1 rotated and cut image with border line.tif]

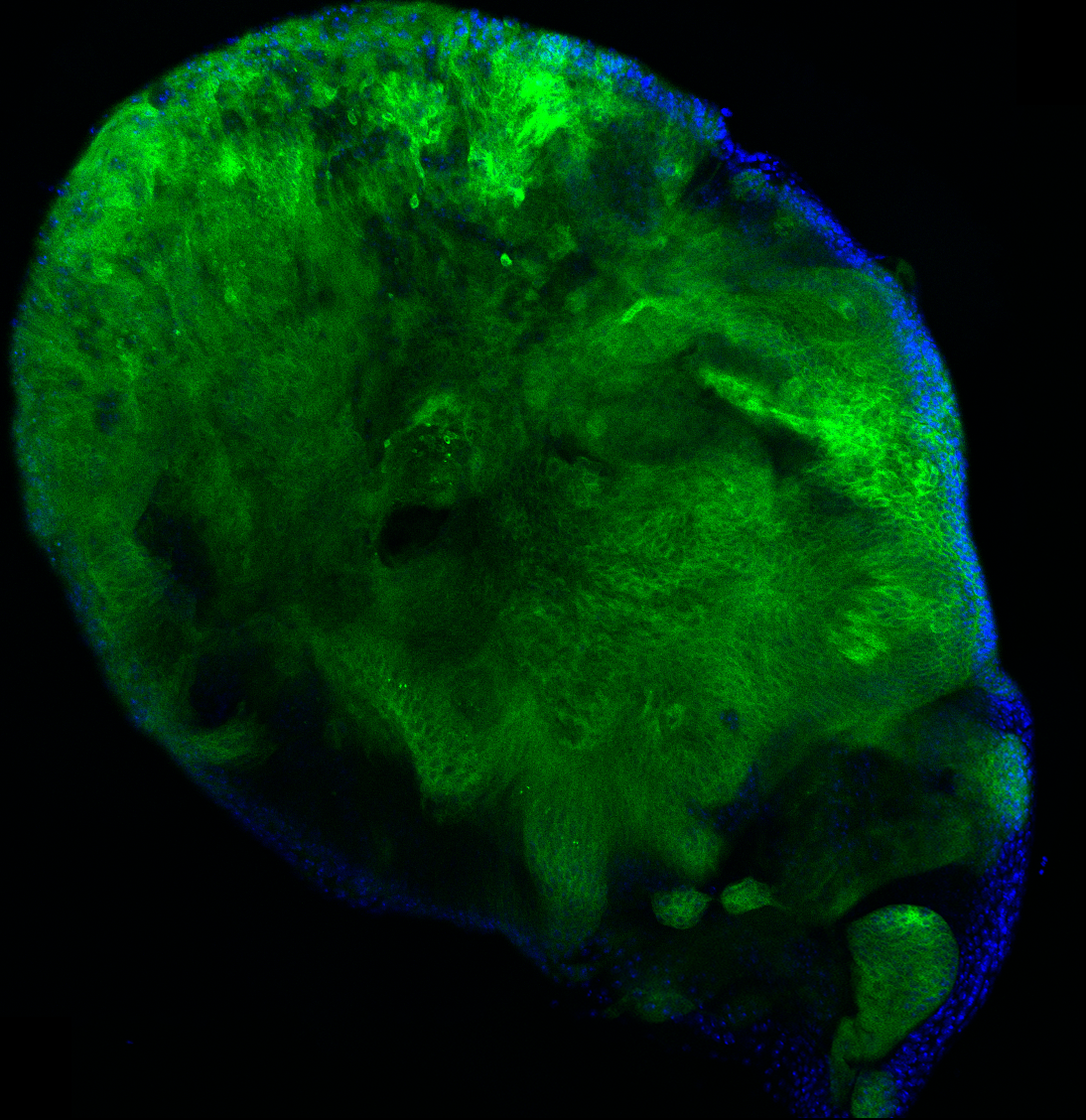

Supplement: Supplementary file 11 — Source data Fig. 7 [file 44318_2025_547_MOESM11_ESM.zip › Figure 7I/8-2 original image.tif]

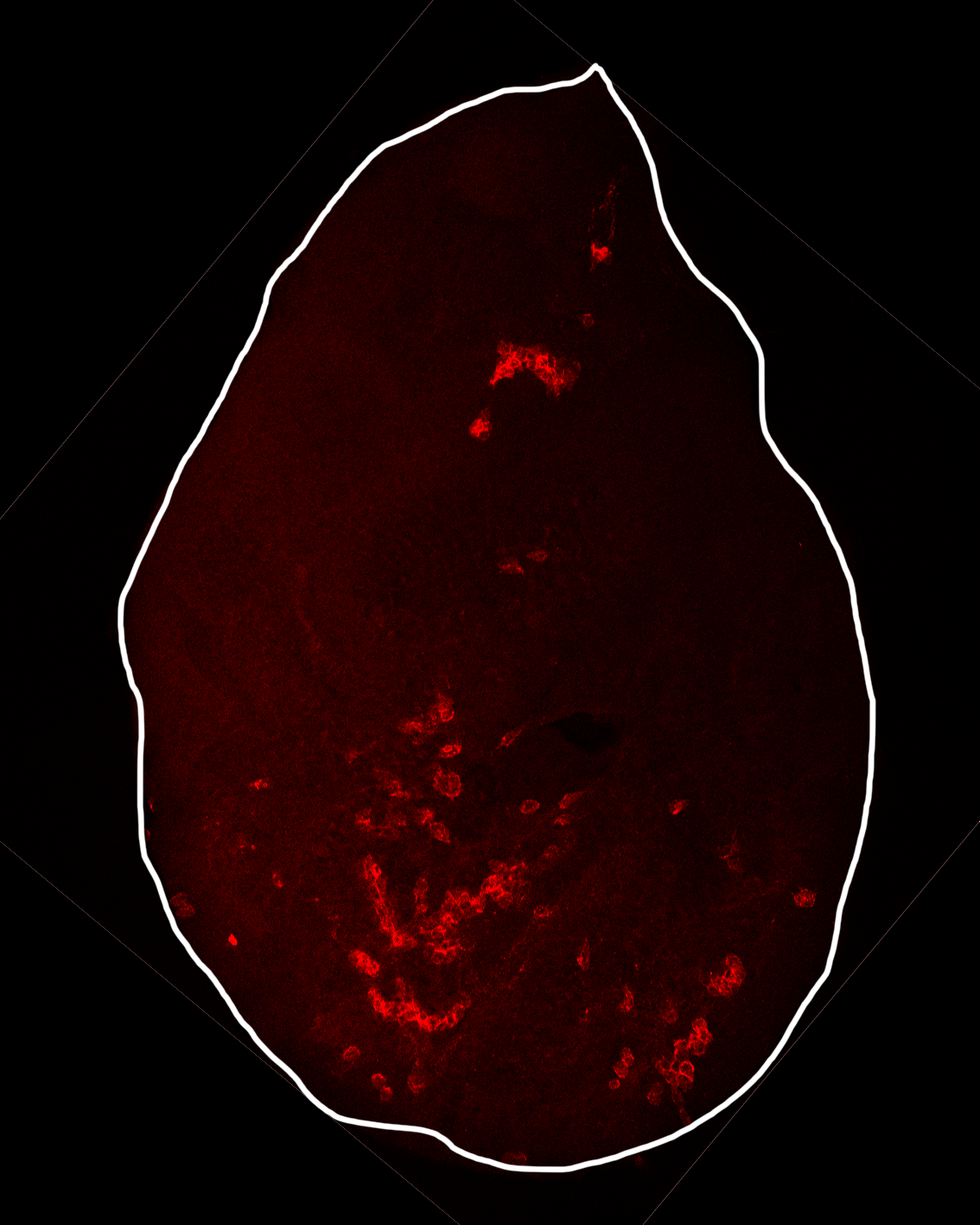

Supplement: Supplementary file 11 — Source data Fig. 7 [file 44318_2025_547_MOESM11_ESM.zip › Figure 7I/9-1 rotated and cut image with border line.tif]

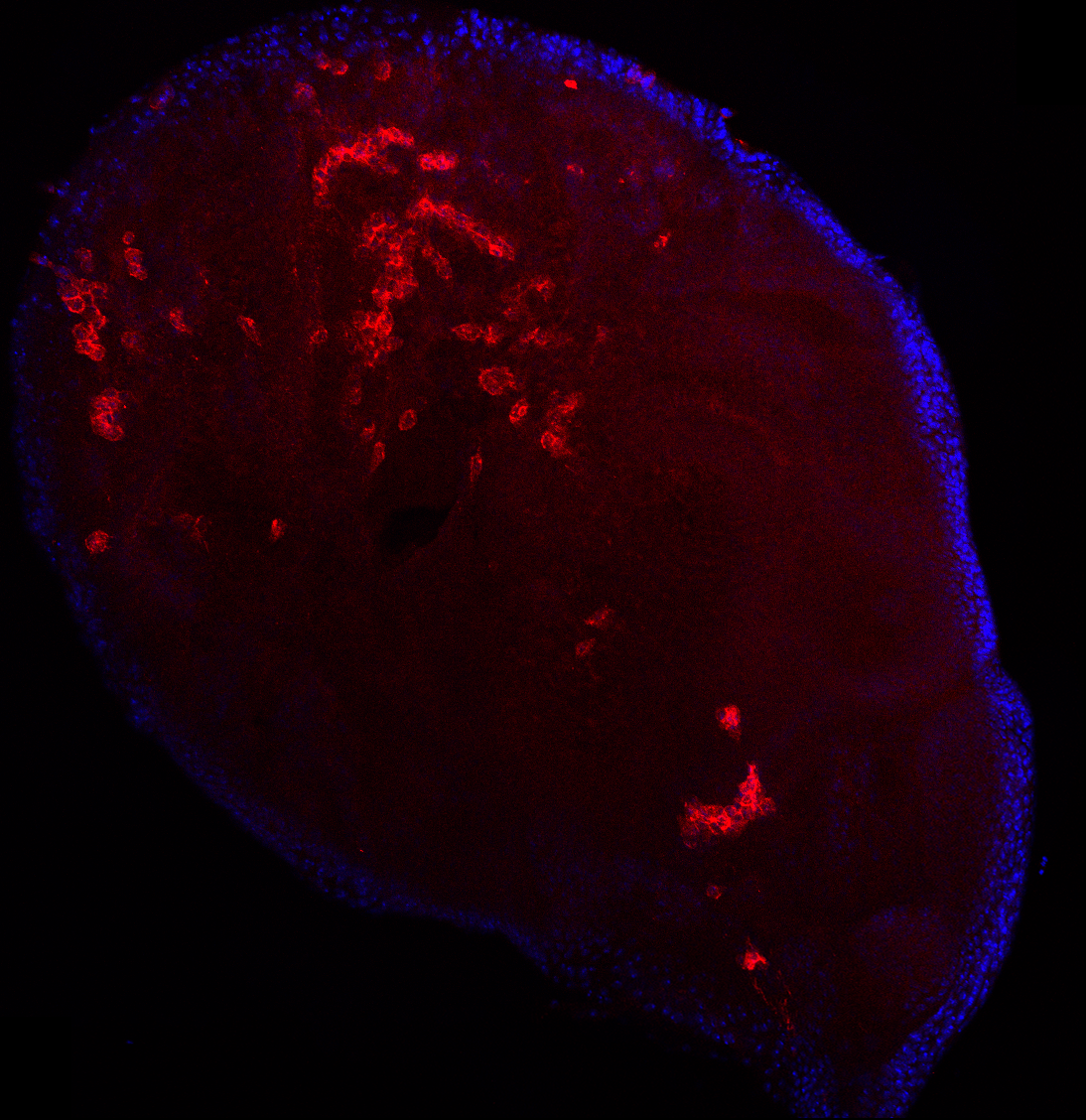

Supplement: Supplementary file 11 — Source data Fig. 7 [file 44318_2025_547_MOESM11_ESM.zip › Figure 7I/9-2 original image.tif]
