# Supplementary material for: Hemocytes facilitate interclonal cooperation-induced tumor malignancy by hijacking the innate immune system in Drosophila
Source: EMBO J. 2025 Aug 22;44(19):5394–428. doi: 10.1038/s44318-025-00547-5 (PMC12489090; doi:10.1038/s44318-025-00547-5)
Supplement: Supplementary file 12 — Expanded View Figures [file 44318_2025_547_MOESM12_ESM.pdf]

Expanded View Figures

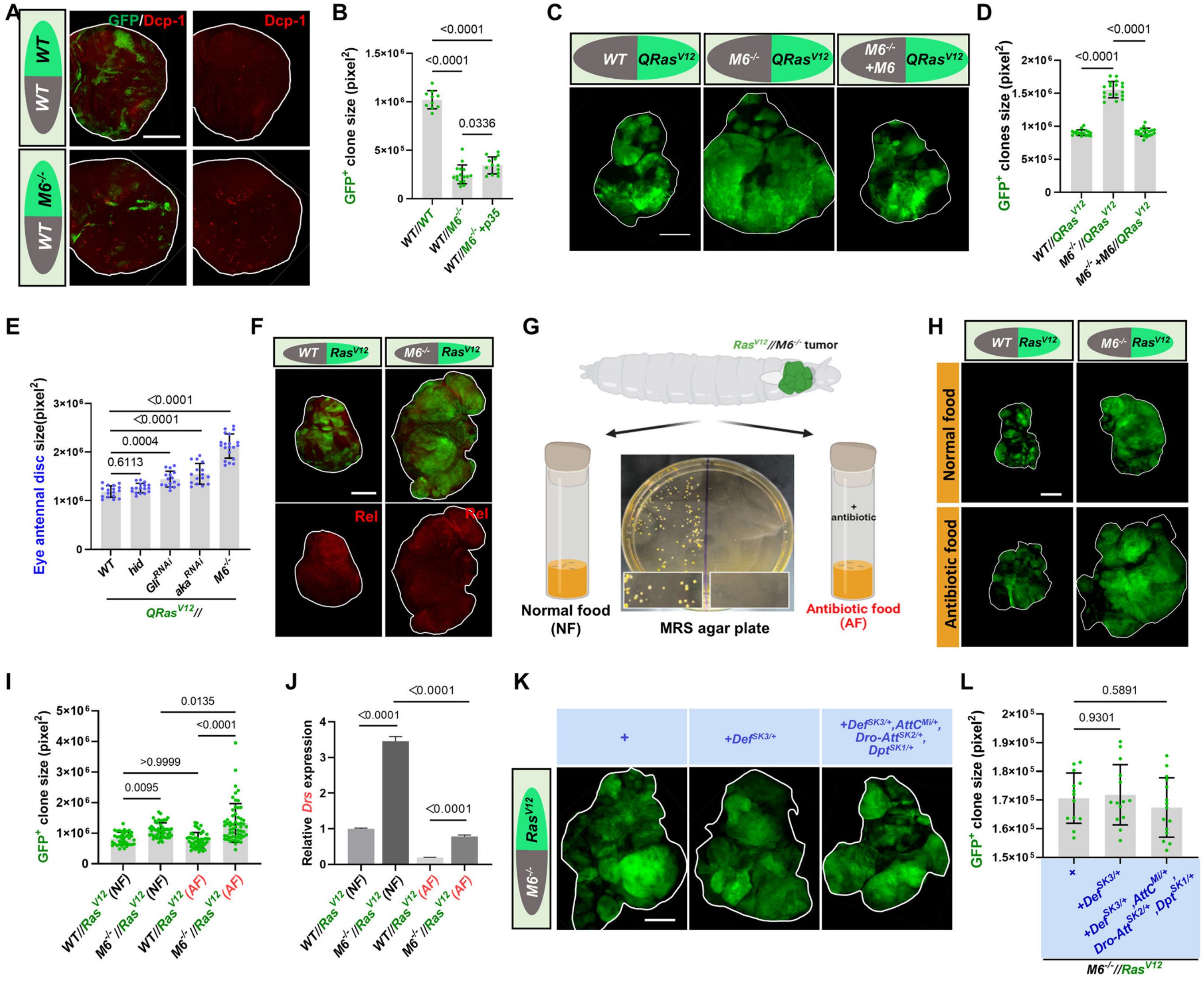

Figure EV1. Ras^{V12}//$M6^{-/-}$ tumor-induced Toll activation is independent of microbial infection.

(**A**) Eye discs bearing GFP-labeled clones of WT and $M6^{-/-}$ were stained with anti-Dcp-1 antibody at AEL-6. The white lines outline the borders of the eye discs. (**B**) Quantification of GFP+ clones' size (from left to right, $n = 10, 16, 13$) with indicated genotypes. Statistical analysis by ordinary one-way ANOVA test; mean ± SD. (**C**) Eye-antennal discs bearing clones of *WT*//$QRas^{V12}$ (AEL-6), and $M6^{-/-}$//$QRas^{V12}$, *M6* + $M6^{-/-}$//$QRas^{V12}$ (AEL-7). The white lines outline the borders of the eye-antennal discs. (**D**) Quantification of GFP+ clones' size (from left to right, $n = 17, 18, 20$) with indicated genotypes. Statistical analysis by ordinary one-way ANOVA test; mean ± SD. (**E**) Quantification of eye-antennal discs' size (from left to right, $n = 17, 17, 16, 16, 20$) with indicated genotypes. Statistical analysis by ordinary one-way ANOVA test; mean ± SD. (**F**) Eye-antennal discs bearing clones of Ras^{V12}//*WT* (AEL-6) and Ras^{V12}//$M6^{-/-}$ (AEL-7) were stained with Relish antibody. The white lines outline the borders of the eye-antennal discs. (**G**) Ras^{V12}//$M6^{-/-}$ tumor-bearing larvae were cultured on normal food (NF) and food supplemented with antibiotics (AF), respectively. Representative bacterial culture images were shown with larvae grown either on normal food or antibiotic food on MRS agar plates. (**H**) Eye-antennal discs bearing clones of Ras^{V12}//*WT* (AEL-6) or Ras^{V12}//$M6^{-/-}$ (AEL-7) cultured with or without antibiotic treatment. The white lines outline the borders of the eye-antennal discs. (**I**) Quantification of GFP^{+} clones' size with the indicated genotypes (from left to right, $n = 38, 50, 47, 58$). Statistical analysis by ordinary one-way ANOVA test; mean ± SD. (**J**) qPCR analysis to determine relative *Drs* mRNA levels of eye-antennal disc dissected from Ras^{V12}//*WT* (AF and NF) or Ras^{V12}//$M6^{-/-}$ (AF and NF) ($n = 4$ independent experiments). Statistical analysis by ordinary one-way ANOVA test; mean ± SD. (**K**) Eye-antennal discs bearing clones of Ras^{V12}//*WT*, Ras^{V12}+$Def^{SK3/+}$//$M6^{-/-}$+$Def^{SK3/+}$, Ras^{V12}+$Def^{SK3/+}$+$AttC^{MI/+}$+$Dro\text{-}Att^{SK2/+}$+$Dpt^{SK1/+}$//$M6^{-/-}$+$Def^{SK3/+}$+$AttC^{MI/+}$+$Dro\text{-}Att^{SK2/+}$+$Dpt^{SK1/+}$ (AEL-7). The white lines outline the borders of the eye-antennal discs. (**L**) Quantification of GFP^{+} clones' size with the indicated genotypes (from left to right, $n = 13, 14, 15$). Statistical analysis by ordinary one-way ANOVA test; mean ± SD. Exact *P* values are shown in the corresponding panels. Scale bars: 100 µm (**A**), 200 µm (**C**, **F**, **H**, **K**).

© The Author(s)

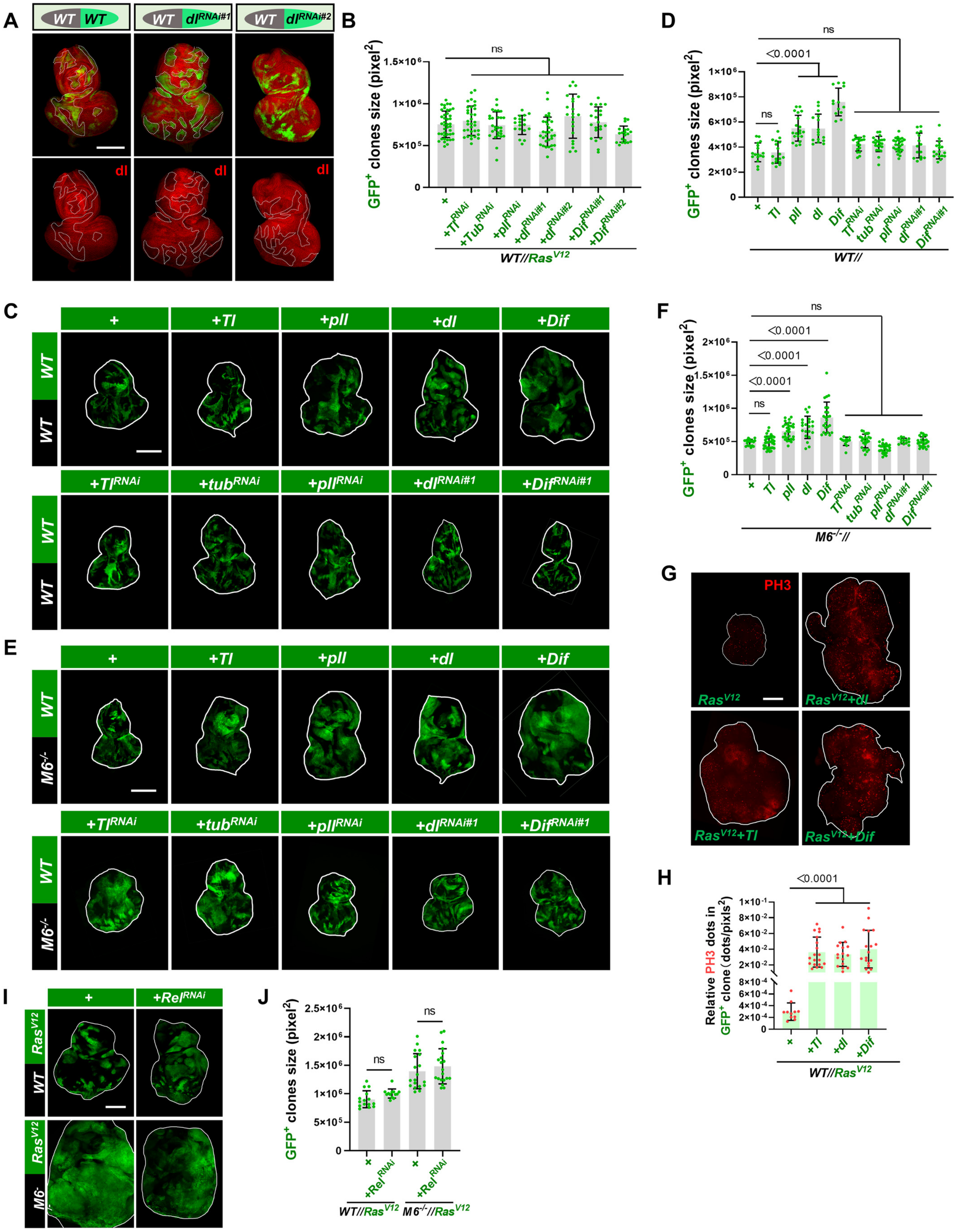
A
WT WT
WT dlRNAi#1
WT dlRNAi#2
dl
B
GFP+ clones size (pixel2)
ns
+TlRNAi
+Tub RNAi
+pllRNAi
+dlRNAi#1
+dlRNAi#2
+DifRNAi#1
+DifRNAi#2
WT//RasV12
C
+
+Tl
+pll
+dl
+Dif
WT
+TlRNAi
+tubRNAi
+pllRNAi
+dlRNAi#1
+DifRNAi#1
D
ns
<0.0001
Tl
pll
dl
Dif
TlRNAi
tub RNAi
pllRNAi
dlRNAi#1
DifRNAi#1
WT//
E
M6-/-
F
M6-/-//
G
PH3
RasV12
RasV12+dl
RasV12+Tl
RasV12+Dif
H
Relative PH3 dots in GFP+ clone (dots/pixls2)
WT//RasV12
I
+RelRNAi
J
WT//RasV12
M6-/-//RasV12

Figure EV2. Inhibition of the Imd pathway does not affect *Ras^{V12}//M6$^{-/-}$* tumors.

(**A**) Eye-antennal discs bearing clones of *WT//WT*, *dl$^{RNAi\#1}$//WT*, *dl$^{RNAi\#2}$//WT* were stained with anti-dl antibody (AEL-6). The white lines outline the borders of the eye-antennal discs. (**B**) Quantification of GFP^{+} clones' size with indicated genotypes (from left to right, $n = 42, 33, 28, 18, 31, 21, 22, 20$). Statistical analysis by ordinary one-way ANOVA test; mean ± SD; ns., not significant. (**C**) Eye-antennal discs bearing *WT//WT*, *Tl//WT*, *pll//WT*, *dl//WT*, *Dif//WT*, *Tl^{RNAi}//WT*, *tub^{RNAi}//WT*, *pll^{RNAi}//WT*, *dl$^{RNAi\#1}$//WT*, *Dif$^{RNAi\#1}$//WT* (AEL-6). The white lines outline the borders of the eye-antennal discs. (**D**) Quantification of GFP^{+} clones' size for the indicated genotypes (from left to right, $n = 17, 17, 20, 16, 13, 18, 22, 25, 15, 17$). Statistical analysis by ordinary one-way ANOVA test; mean ± SD; ns., not significant. (**E**) Eye-antennal discs bearing *WT//M6$^{-/-}$*, *Tl//M6$^{-/-}$*, *pll//M6$^{-/-}$*, *dl//M6$^{-/-}$*, *Dif//M6$^{-/-}$*, *Tl^{RNAi}//M6$^{-/-}$*, *tub^{RNAi}//M6$^{-/-}$*, *pll^{RNAi}//M6$^{-/-}$*, *dl$^{RNAi\#1}$//M6$^{-/-}$*, *Dif$^{RNAi\#1}$//M6$^{-/-}$* (AEL-6). The white lines outline the borders of the eye-antennal discs. (**F**) Quantification of GFP^{+} clones' size for the indicated genotypes (from left to right, $n = 18, 34, 29, 21, 24, 13, 24, 25, 13, 25$). Statistical analysis by ordinary one-way ANOVA test; mean ± SD; ns., not significant. (**G**) Eye-antennal discs with *Ras^{V12}//WT* clones (AEL-6), and with *Ras^{V12} + Tl//WT*, *Ras^{V12}+dl//WT*, *Ras^{V12}+ Dif//WT* clones (AEL-8), were stained with anti-PH3 antibody. The white lines outline the borders of the eye-antennal discs. (**H**) Quantification of PH3 dots per GFP^{+} clones' area (from left to right, $n = 11, 19, 16, 18$). Statistical analysis by ordinary one-way ANOVA test; mean ± SD. (**I**) Eye-antennal discs with *Ras^{V12}//WT*, *Ras^{V12}+Rel^{RNAi}//WT* clones (AEL-6), and with *Ras^{V12}//M6$^{-/-}$*, *Ras^{V12}+Rel^{RNAi}// M6$^{-/-}$* clones (AEL-7). The white lines outline the borders of the eye-antennal discs. (**J**) Quantification of GFP^{+} clones' size for the indicated genotypes (from left to right, $n = 15, 13, 19, 20$). Statistical analysis by ordinary one-way ANOVA test; mean ± SD. Exact *P* values are shown in the corresponding panels. Scale bars: 200 µm (**A**, **C**, **E**, **G**, **I**).

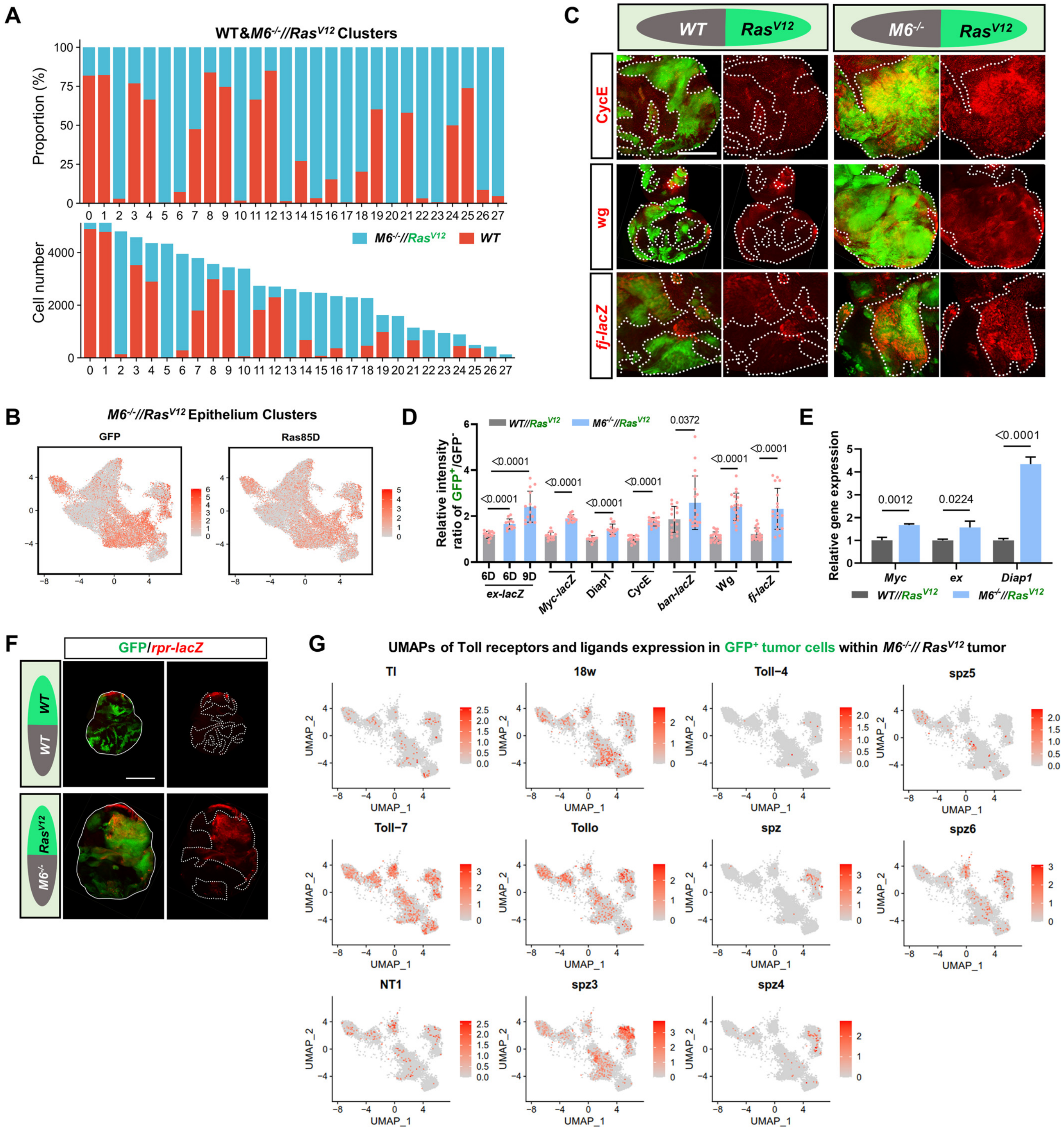
A
WT&M6-/-//RasV12 Clusters
Proportion (%)
Cell number
M6-/-//RasV12 WT
B
M6-/-//RasV12 Epithelium Clusters
GFP
Ras85D
C
WT RasV12
M6-/- RasV12
CycE
wg
fj-lacZ
D
WT//RasV12 M6-/-//RasV12
Relative intensity ratio of GFP+/GFP-
ex-lacZ
Myc-lacZ
Diap1
CycE
ban-lacZ
Wg
fj-lacZ
E
Relative gene expression
Myc
ex
Diap1
WT//RasV12 M6-/-//RasV12
F
GFP/rpr-lacZ
G
UMAPs of Toll receptors and ligands expression in GFP+ tumor cells within M6-/-// RasV12 tumor
Tl
18w
Toll−4
spz5
Toll−7
Tollo
spz
spz6
NT1
spz3
spz4
UMAP_1
UMAP_2

 © The Author(s)

Figure EV3. Both *Ras*^{V12}*//M6*$^{-/-}$ and *Ras*^{V12}+Toll activation-induced tumors display enhanced expression of *yki* target genes.

(**A**) The proportion (upper) and number (bottom) of cells of each cluster from *WT* or *Ras*^{V12}*//M6*$^{-/-}$ discs are shown (data from Fig. 3B). (**B**) UMAP plots showing the marker genes expression of *GFP* and *Ras85D* for the *Ras*^{V12}*//M6*$^{-/-}$ epithelial cells. (**C**) Eye-antennal discs harboring *Ras*^{V12}*//WT* (AEL-6) and *Ras*^{V12}*//M6*$^{-/-}$ (AEL-7) clones were stained for CycE, Wg, and *fj-lacZ*. The white dashed lines demarcate the boundaries of GFP-positive clones. (**D**) Quantification of relative staining intensity across different experimental groups (from left to right, $n = 15$, 12, 14, 14, 14, 13, 15, 16, 17, 15, 18, 20, 20,18, 17). Statistical analysis by unpaired two-tailed Student's t-test; mean ± SD. (**E**) qPCR analysis of mRNA levels for *yki* target genes (*Myc*, *ex*, and *Diap1*) in eye-antennal discs from the indicated genotypes ($n = 3$ independent experiments). Statistical analysis by unpaired two-tailed Student's *t* test; mean ± SD. (**F**) Eye-antennal discs bearing clones of *WT//WT* (AEL-6) and *Ras*^{V12}*//M6*$^{-/-}$ (AEL-7) were stained with anti-β-galactosidase antibodies to visualize the *rpr-lacZ* reporter. White solid lines outline the borders of the eye-antennal discs, while white dashed lines indicate the boundaries of GFP-positive clones. (**G**) UMAP plots showing the expression of Toll receptors and ligands in the GFP+ epithelial cells of *Ras*^{V12}*//M6*$^{-/-}$ tumors. Exact *P* values are shown in the corresponding panels. Scale bars: 100 μm (**C**) and 200 μm (**F**).

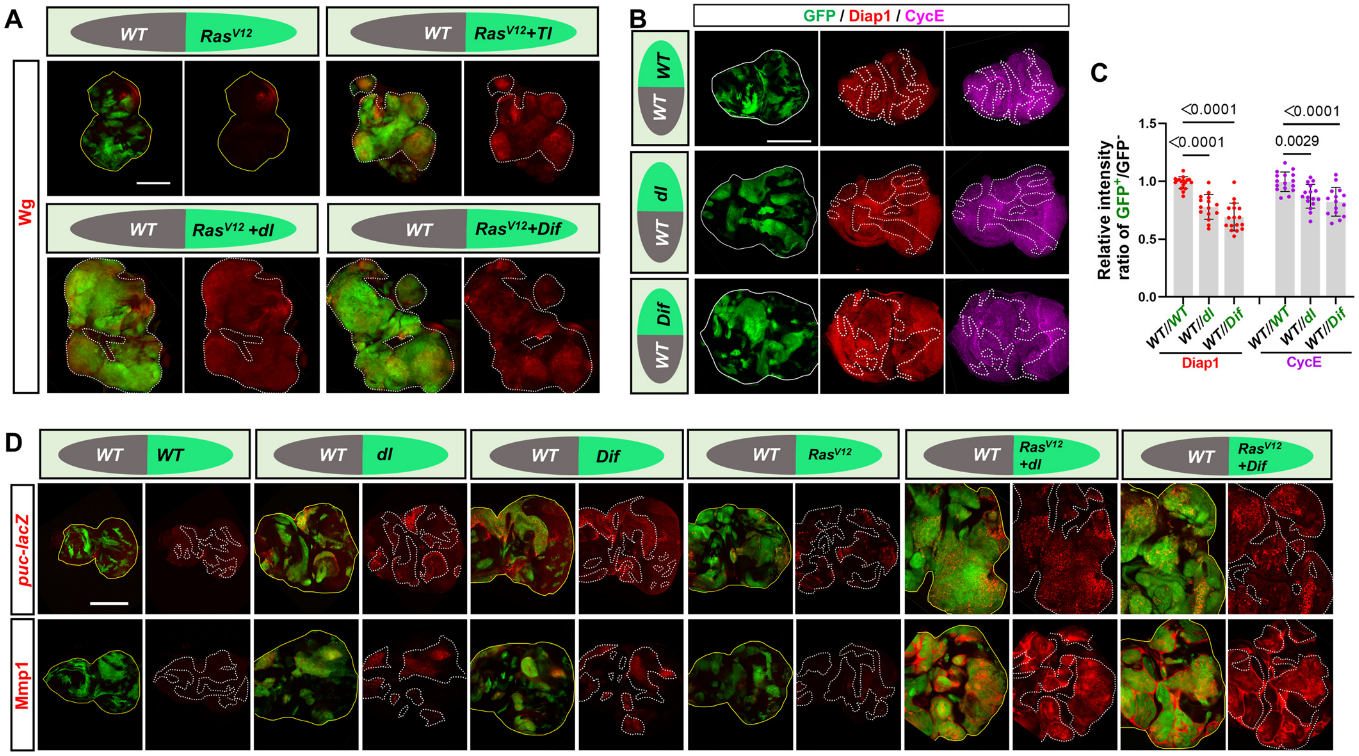

Figure EV4. Toll pathway genetically acts upstream of JNK pathway.

(**A**) Eye-antennal discs with *Ras*^{V12}*//WT* clones (AEL-6), and *Ras*^{V12} + *Tl//WT*, *Ras*^{V12}+ *dl//WT*, *Ras*^{V12}+ *Dif//WT* clones (AEL-7) were stained with anti-Wg antibody. The yellow lines outline the borders of the eye-antennal discs, the white dashed lines mark the boundaries of the GFP-positive clones. (**B**) Eye-antennal discs bearing clones of *WT//WT*, *dl//WT*, *Dif//WT* were stained with anti-Diap1 and anti-CycE antibodies (AEL-6). The white lines outline the borders of the eye-antennal discs, the white dashed lines mark the boundaries of the GFP-positive clones. (**C**) Quantification of relative staining intensity (from left to right, $n = 17, 15, 17, 16, 16, 15$). Statistical analysis by ordinary one-way ANOVA test; mean ± SD. (**D**) Eye-antennal discs with *WT//WT*, *dl//WT*, *Dif//WT*, *Ras*^{V12}*//WT* clones (AEL-6), as well as *Ras*^{V12}+ *dl//WT* and *Ras*^{V12}+ *Dif //WT* clones (AEL-7), were stained with antibodies to detect *puc-lacZ* and Mmp1 expression. Yellow lines outline the borders of the eye-antennal discs, while white dashed lines indicate the boundaries of GFP-positive clones. Exact *P* values are shown in the corresponding panels. Scale bars: 200 μm (**A**, **B**, **D**).

 © The Author(s)

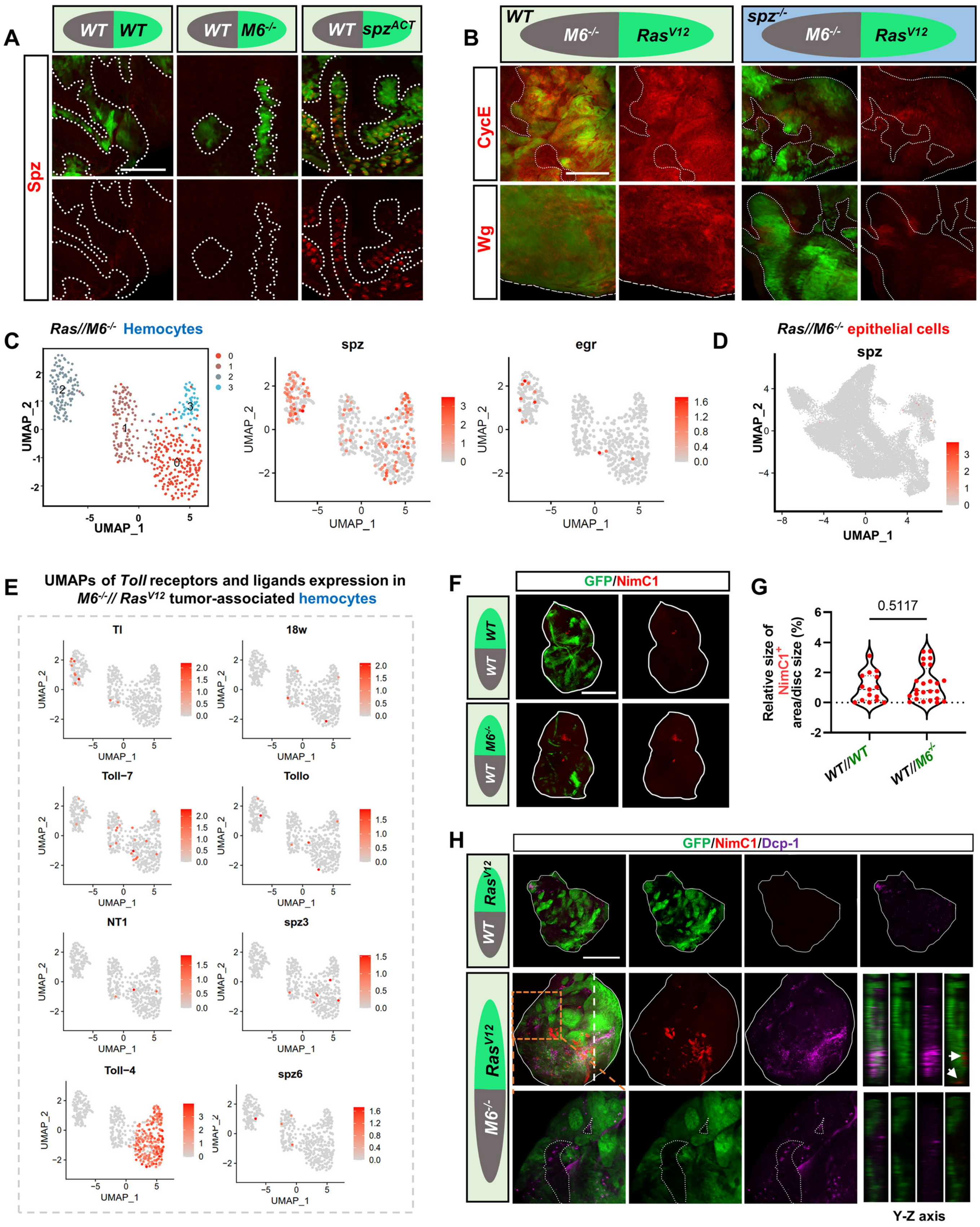
A
WT WT
WT M6-/-
WT spzACT
Spz
B
WT
M6-/- RasV12
spz-/-
M6-/- RasV12
CycE
Wg
C
Ras//M6-/- Hemocytes
spz
egr
UMAP_1
UMAP_2
D
Ras//M6-/- epithelial cells
spz
E
UMAPs of Toll receptors and ligands expression in
M6-/-// RasV12 tumor-associated hemocytes
Tl
18w
Toll−7
Tollo
NT1
spz3
Toll−4
spz6
F
GFP/NimC1
WT WT
WT M6-/-
G
0.5117
Relative size of NimC1+ area/disc size (%)
WT//WT
WT//M6-/-
H
GFP/NimC1/Dcp-1
WT RasV12
RasV12 M6-/-
Y-Z axis

Figure EV5. scRNA-seq analysis reveals the specific upregulation of *spz* in *Ras^{V12}//M6$^{-/-}$* attached hemocytes, rather than epithelial cells.

(**A**) Eye-antennal discs bearing clones of *WT//WT*, *M6$^{-/-}$//WT* and *spz^{ACT}//WT* were stained with anti-Spz antibody (AEL-6). White dashed lines mark the boundaries of the GFP-positive clones. (**B**) Eye-antennal discs bearing clones of *Ras^{V12}//M6$^{-/-}$*, *Ras^{V12}+spz$^{-/-}$//M6$^{-/-}$+spz$^{-/-}$* were stained with anti-cycE and anti-Wg antibodies (AEL-7). White dashed lines mark the boundaries of the GFP-positive clones. (**C**) UMAP plot showing the re-clustered *Ras^{V12}//M6$^{-/-}$*-attached hemocytes. A total of 516 hemocytes were classified into four subclusters (left). UMAP plot showing the *spz* and *egr* expression in *Ras^{V12}//M6$^{-/-}$*-attached hemocytes. (**D**) UMAP plot showing the *spz* expression in *Ras^{V12}//M6$^{-/-}$* epithelial cells. (**E**) UMAP plot showing the expression of Toll receptors and ligands in *Ras^{V12}//M6$^{-/-}$*-attached hemocytes. (**F**) Eye-antennal discs bearing clones of *WT//WT*, *M6$^{-/-}$//WT* were stained with anti-NimC1 antibody (AEL-6). White lines outline the borders of the eye-antennal discs. (**G**) Quantification of relative total NimC1^{+} area compared to the area of disc size for the indicated genotypes (from left to right, $n = 15$, 24). Violin plots represent kernel density estimation of data distribution, with the width proportionate to the number of points at each Y value. Scattered red dots represent individual data points. Statistical analysis by unpaired two-tailed Student's t-test. (**H**) Eye-antennal discs bearing clones of *Ras^{V12}//WT* (upper row, AEL-6) and *Ras^{V12}//M6$^{-/-}$* (lower two rows, AEL-7) were stained with anti-NimC1 and anti-Dcp-1 antibodies. In *Ras^{V12}//M6$^{-/-}$* disc, bottom images show xy cross-section, right images show yz across-section. Straight white dashed line indicates the position of vertical section images, the white lines outline the borders of the eye-antennal discs, while the white dashed lines mark the boundaries of the GFP-positive clones. Arrows highlight NimC1-positive hemocytes adhering to GFP-positive cells. Exact *P* values are shown in the corresponding panels. Scale bars: 50 µm (**A**), 100 µm (**B**), and 200 µm (**F**, **H**).

 © The Author(s)

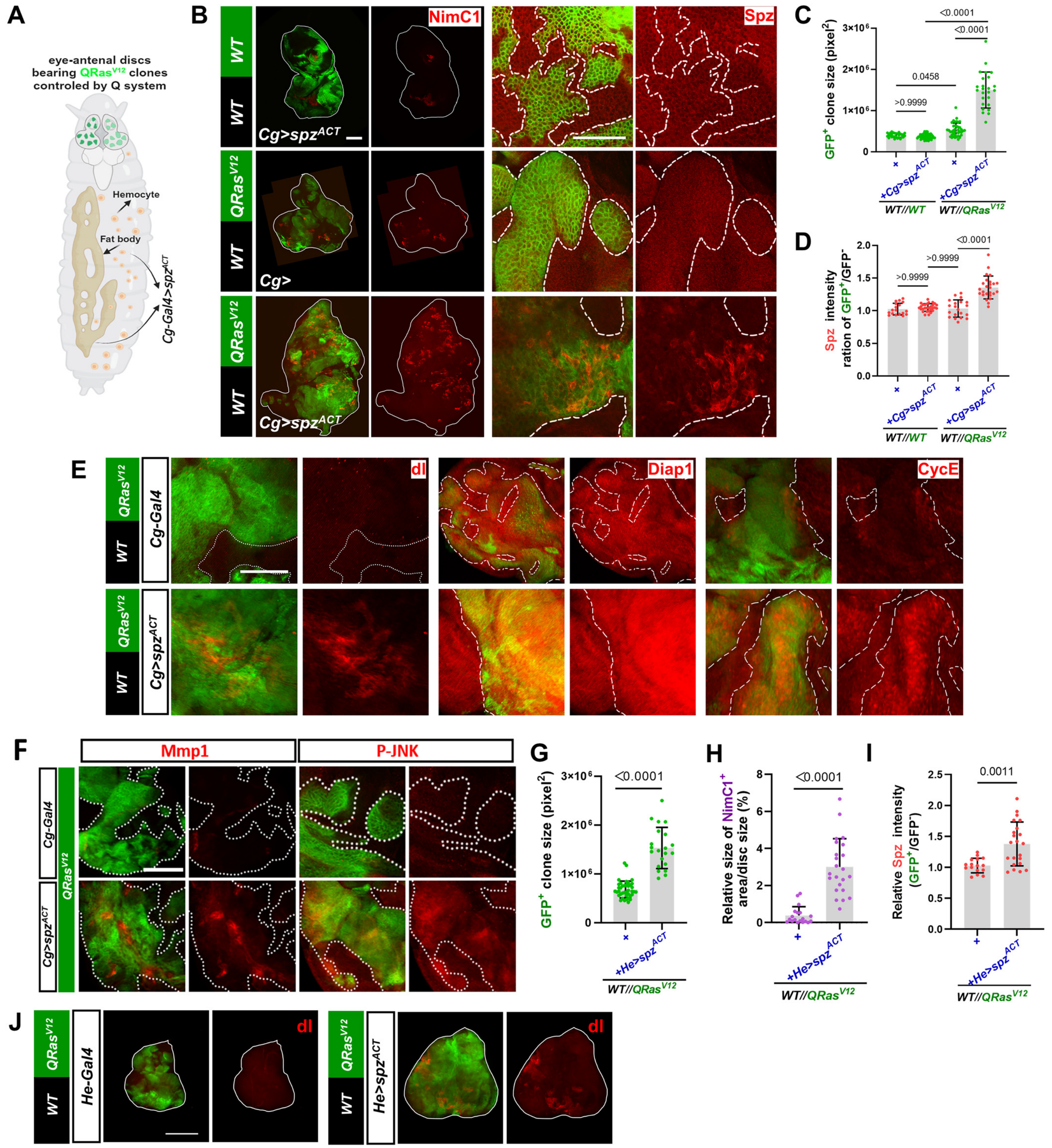
A
eye-antenal discs
bearing QRas^{V12} clones
controled by Q system
Hemocyte
Fat body
Cg-Gal4>spz^{ACT}
B
NimC1
Spz
Cg>spz^{ACT}
Cg>
C
GFP^{+} clone size (pixel^{2})
<0.0001
0.0458
>0.9999
WT//WT
WT//QRas^{V12}
D
Spz intensity ration of GFP^{+}/GFP^{-}
E
dl
Diap1
CycE
Cg-Gal4
F
Mmp1
P-JNK
G
H
Relative size of NimC1^{+} area/disc size (%)
I
Relative Spz intensity (GFP^{+}/GFP^{-})
0.0011
+He>spz^{ACT}
J
He-Gal4
He>spz^{ACT}

Figure EV6. Overexpression of activated *spz* driven by *Cg-gal4* and *He-gal4* promotes the overgrowth of *QRas*^{V12} clones in the distal eye-antennal disc.

(**A**) Schematic representation of the dual-expression system designed to overexpress activated *spz* (*spz*^{ACT}) in hemocytes and fat bodies under the control of *Cg* promoter and simultaneously inducing GFP-labeled *QRas*^{V12} clones in the distal eye-antennal disc controlled by the Q system. (**B**) Eye-antennal discs bearing GFP-labeled clones of wild-type cells (upper row, AEL-6), *QRas*^{V12}-overexpression clones without (middle row, AEL-6), or with *spz*^{ACT} overexpression driven by the *Cg* promoter (lower row, AEL-7) were stained with anti-NimC1 and anti-Spz antibodies. White solid lines mark the borders of the eye-antennal discs, while white dashed lines delineate the boundaries of the GFP-positive clones. (**C**) Quantification of GFP^{+} clones' size of indicated flies (from left to right, $n = 29, 48, 39, 25$). Statistical analysis by ordinary one-way ANOVA test; mean ± SD. (**D**) Quantification of relative Spz signal intensity in the indicated genotypes (from left to right, $n = 18, 29, 20, 25$). Statistical analysis by ordinary one-way ANOVA test; mean ± SD. (**E**) Eye-antennal discs harboring GFP-labeled *QRas*^{V12} clones, either without (upper row, AEL-6), or with *spz*^{ACT} expression under the control of the *Cg* promoter (lower row, AEL-7) were stained with anti-dl, anti-Diap1, and anti-CycE antibodies. White dashed lines mark the boundaries of the GFP-positive clones. (**F**) Eye-antennal discs harboring GFP-labeled *QRas*^{V12} clones, either without (upper row, AEL-6), or with *spz*^{ACT} expression under the control of the *Cg* promoter (lower row, AEL-7) were stained with anti-Mmp1, and anti-P-JNK antibodies. White dashed lines mark the boundaries of the GFP-positive clones. (**G**) Quantification of GFP-labeled *QRas*^{V12} clones without or with *spz*^{ACT} expression under the *He* promoter (from left to right, $n = 40, 21$). Statistical analysis by unpaired non-parametric Mann-Whitney test; mean ± SD. (**H**) Quantification of relative total NimC1^{+} area compared to the area of disc size with indicated genotypes (from left to right, $n = 19, 23$). Statistical analysis by unpaired non-parametric Mann–Whitney test; mean ± SD. (**I**) Quantification of relative Spz signal intensity of indicated flies (from left to right, $n = 16, 22$). Statistical analysis by unpaired non-parametric Mann–Whitney test; mean ± SD. (**J**) Eye-antennal discs harboring GFP-labeled *QRas*^{V12} clones, either without (AEL-6), or with *spz*^{ACT} expression under the control of *He* promoter (AEL-7) were stained with anti-dl antibody. White lines outline the borders of the eye-antennal discs. Exact *P* values are shown in the corresponding panels. Scale bars: 100 µm (**B**, **E**, **F**), 200 µm (**J**).

 © The Author(s)

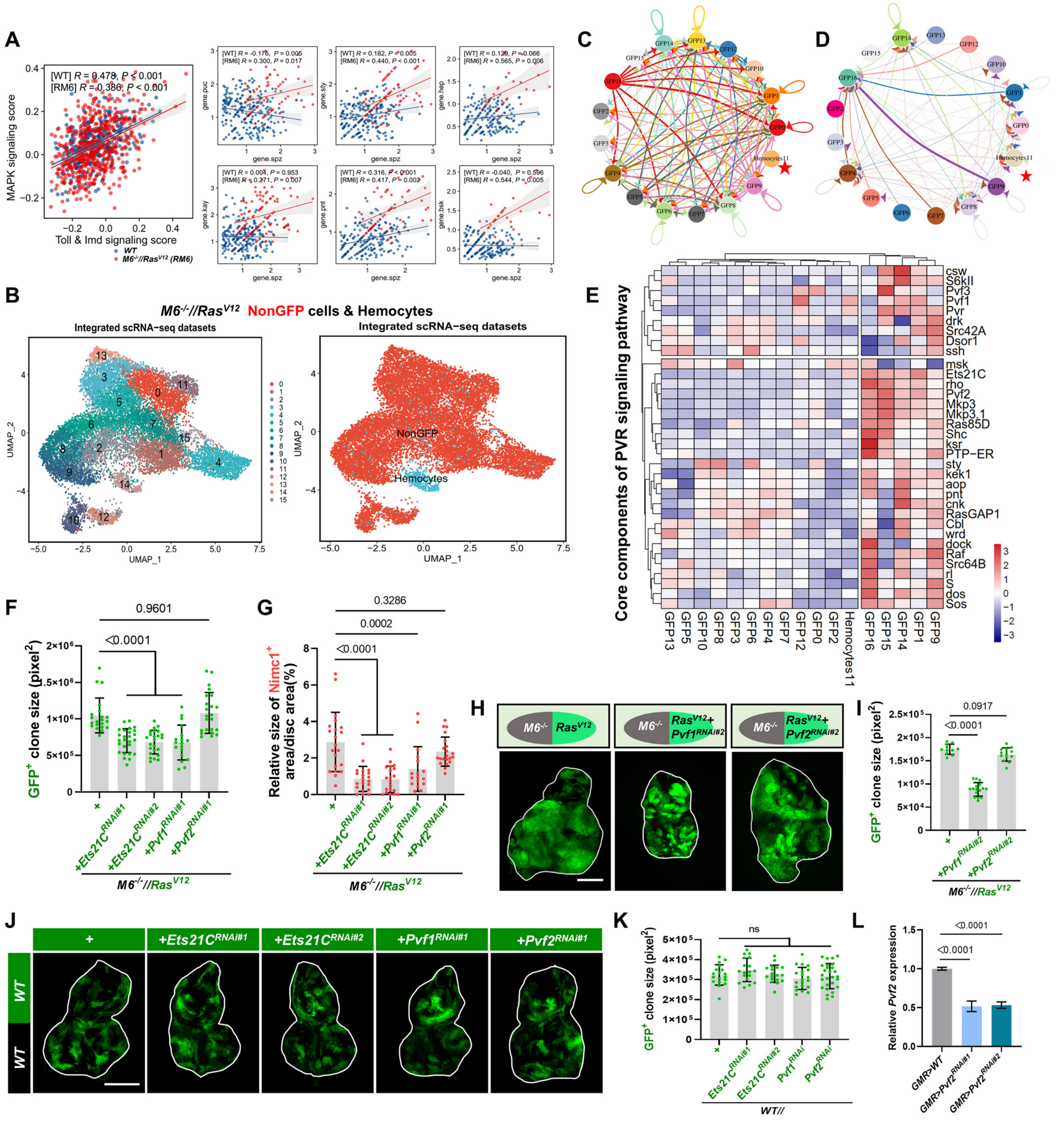
A
MAPK signaling score
Toll & Imd signaling score
WT
M6-/-//RasV12 (RM6)
B
M6-/-//RasV12 NonGFP cells & Hemocytes
Integrated scRNA-seq datasets
NonGFP
Hemocytes
UMAP_1
UMAP_2
C
D
E
Core components of PVR signaling pathway
F
GFP+ clone size (pixel2)
M6-/-//RasV12
G
Relative size of Nimc1+ area/disc area(%)
H
I
J
K
L
Relative Pvf2 expression

Figure EV7. In *Ras*^{V12}*//M6*$^{-/-}$ tumor-associated hemocytes, the expression level of *spz* is positively correlated with the expression of key genes in the JNK pathway.

(**A**) Expression correlations analyses of WT hemocytes and *Ras*^{V12}*//M6*$^{-/-}$ tumor-associated hemocytes. Toll & Imd signaling score and MAPK signaling are positively correlated (left), the expression of *spz* showed pan-positive correlations with MAPK signaling critical members, including *puc*, *sty*, *hep*, *kay*, *pnt*, and *bsk* (right). These correlations were determined by using Spearman's correlation analysis, with coefficients R and *P*-values reported, where $P < 0.05$ indicates statistical significance. (**B**) UMAP plots showing integrated hemocytes and non-GFP cells derived from *Ras*^{V12}*//M6*$^{-/-}$ samples. A total of 16 clusters were identified (left, resolution = 1.2). (**C**) Circular plot showing a significant enrichment in the EGFR signaling pathway between multiple *Ras*^{V12}*//M6*$^{-/-}$ GFP^{+} tumor cell clusters and *Ras*^{V12}*//M6*$^{-/-}$ tumor-associated hemocytes (cluster 11, marked with a red asterisk). Notably, *Ras*^{V12}*//M6*$^{-/-}$ GFP^{+} tumor cells do not communicate with hemocytes via the EGFR pathway. (**D**) Circular plot showing a significant enrichment in the FGFR signaling pathway between multiple *Ras*^{V12}*//M6*$^{-/-}$ GFP^{+} tumor cell clusters and *Ras*^{V12}*//M6*$^{-/}$ tumor-associated hemocytes (cluster 11, marked with a red asterisk). The analysis reveals weak intercellular communication between *Ras*^{V12}*//M6*$^{-/-}$ GFP^{+} tumor cells and hemocytes via the FGFR pathway. (**E**) Heatmap displaying the expression patterns of PVR signaling pathway members across different clusters (as shown in Fig. 7F). Each row represents the average expression level of a gene, normalized by z-score. Clusters 1, 9, 14, and 15 from GFP^{+} tumors exhibit high expression levels of ligands (*Pvf1*, *Pvf2*, and *Pvf3*), whereas hemocytes show elevated expression of the receptor *Pvr*. (**F**) Quantification of GFP^{+} clone sizes for the indicated genotypes (from left to right, $n = 21$, 26, 24, 18, 23). Statistical analysis by ordinary one-way ANOVA test; mean ± SD. (**G**) Quantification of the relative NimC1^{+} area for the indicated genotypes (from left to right, $n = 21$, 20, 20, 17, 21). Statistical analysis by ordinary one-way ANOVA test; mean ± SD. (**H**) Eye-antennal discs bearing clones of *Ras*^{V12}*//M6*$^{-/-}$, *Ras*^{V12}*+Pvf1*$^{RNAi\#2}$*//M6*$^{-/-}$, and *Ras*^{V12}*+Pvf2*$^{RNAi\#2}$*//M6*$^{-/-}$ were stained with anti-NimC1 antibody (AEL-7). White lines delineate the borders of the eye-antennal discs. (**I**) Quantification of GFP^{+} clone sizes for the indicated genotypes (from left to right, $n = 11$, 16, 13). Statistical analysis by ordinary one-way ANOVA test; mean ± SD. (**J**) Fluorescent eye-antennal discs bearing the following genotypes: *WT//WT, Ets21C*$^{RNAi\#1}$*//WT, Ets21C*$^{RNAi\#2}$*//WT, Pvf1*$^{RNAi\#1}$*//WT, Pvf2*$^{RNAi\#1}$*//WT* (AEL-6). White lines delineate the borders of the eye-antennal discs. (**K**) Quantification of GFP^{+} clone sizes for the indicated genotypes (from left to right, $n = 18$, 18, 18, 20, 27). Statistical analysis by ordinary one-way ANOVA test; mean ± SD. (**L**) qPCR analysis of *Pvf2* mRNA levels in the adult heads with the indicated genotypes ($n = 3$ independent experiments). Statistical analysis by ordinary one-way ANOVA test; mean ± SD. Exact *P* values are shown in the corresponding panels. Scale bars: 200 µm (**H**, **J**).

 © The Author(s)

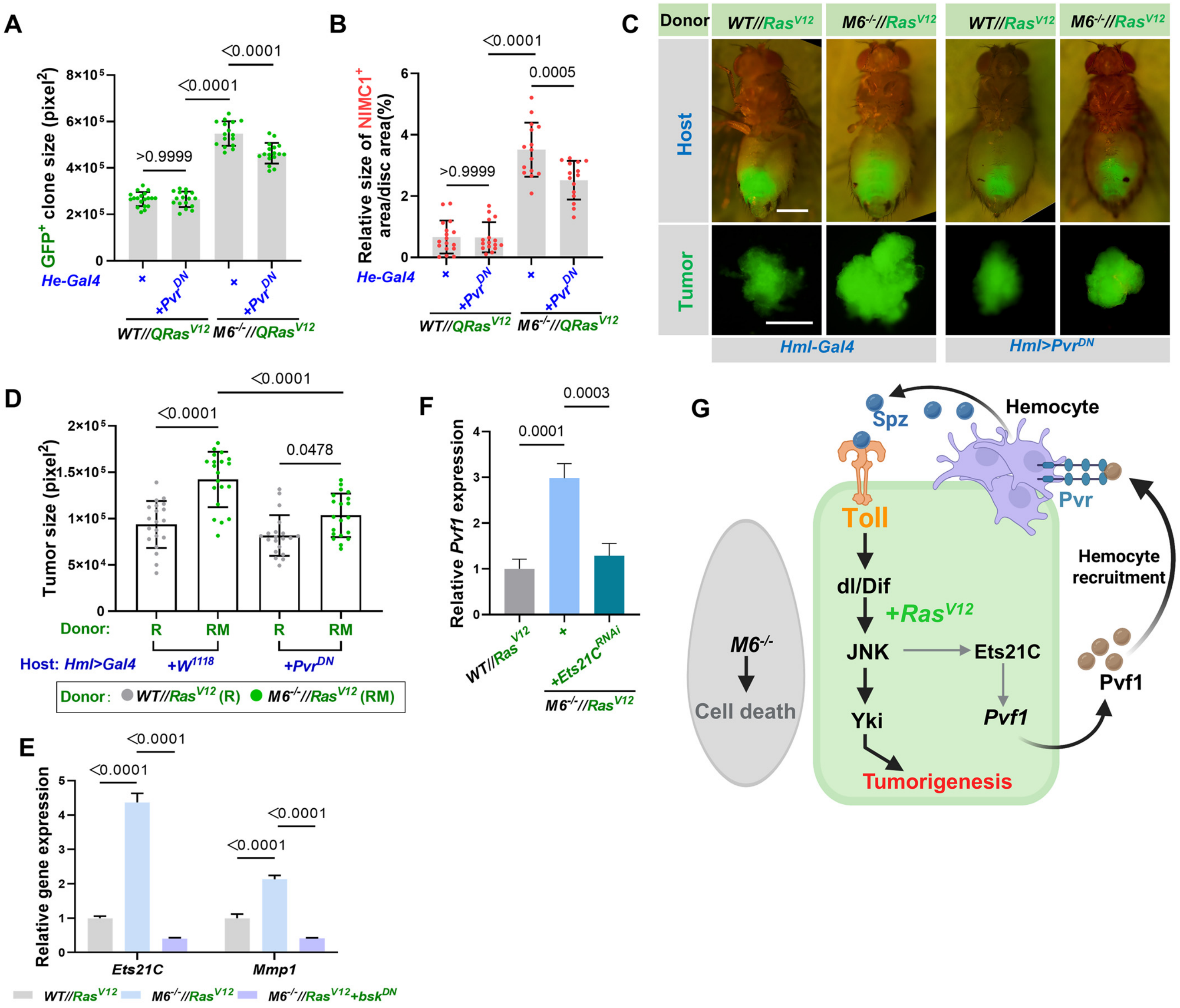

Figure EV8. Pvr in hemocytes promotes the proliferation of Ras^{V12}//$M6^{-/-}$ tumors and hemocyte adhesion.

(**A**) Quantification of GFP+ clone sizes for indicated genotypes (from left to right, $n = 19, 17, 16, 17$). Statistical analysis by ordinary one-way ANOVA test; mean ± SD. (**B**) Quantification of the relative NimC1+ area for the indicated genotypes (from left to right, $n = 17, 16, 15, 15$). Statistical analysis by ordinary one-way ANOVA test; mean ± SD. (**C**) *Ras^{V12}//WT* and *Ras^{V12}//$M6^{-/-}$* eye discs were transplanted into female adults with the following genotypes: *Hml-Gal4* and *Hml>Pvr^{DN}*. Transplants were dissected 8 days post-transplantation for quantification. (**D**) Quantification of GFP+ transplanted tumors with indicated genotypes (from left to right, $n = 20, 20, 20, 20$). Statistical analysis by ordinary one-way ANOVA test; mean ± SD. (**E**) qPCR analysis of mRNA levels for *Ets21C* and *Mmp1* in eye-antennal discs of indicated flies ($n = 3$ independent experiments). Statistical analysis by ordinary one-way ANOVA test; mean ± SD. (**F**) qPCR analysis of mRNA levels of *Pvf1* in eye-antennal discs of indicated flies ($n = 3$ independent experiments). Statistical analysis by ordinary one-way ANOVA test; mean ± SD. (**G**) Proposed working model illustrating the progression of *Ras^{V12}//$M6^{-/-}$* tumors: Loss of the tricellular junction protein M6 promotes tumor malignancy in neighboring *Ras^{V12}* clones by inducing secretion of Pvf1. Pvf1 acts as a chemoattractant, recruiting hemocytes through the Pvr receptor. These recruited hemocytes, in turn, activate a paracrine Spz-Toll signaling axis within *Ras^{V12}* clones. Activation of the Toll pathway synergizes with oncogenic *Ras* to promote tumorigenesis through a JNK-Hippo signaling cascade. Exact *P* values are shown in the corresponding panels. Scale bar: 500 µm (**C**).
